# Actinomycin Analogs From *Streptomyces polyantibioticus* S4D258: Structural Elucidation and Antiproliferative Activity Against Triple‐Negative Breast Cancer

**DOI:** 10.1002/cbdv.71791

**Published:** 2026-09-30

**Authors:** He Pang, Xiao‐Min Huang, Usama Ramadan Abdelmohsen, Li Shen, Jun Wu

**Affiliations:** ^1^ Guangdong Provincial Engineering Technology Research Center for Innovative Drugs and Bioproducts School of Pharmacy Guangdong Medical University Dongguan P. R. China; ^2^ Department of Pharmacognosy Faculty of Pharmacy Minia University Minia Egypt; ^3^ Dongguan Key Laboratory For Marine Innovative Drugs and Bioproducts Guangdong Medical University Dongguan P. R. China

**Keywords:** actinomycins, ferroptosis, *streptomyces polyantibioticus*, triple‐negative breast cancer

## Abstract

Fifteen new actinomycin analogs were obtained from the marine‐derived *Streptomyces polyantibioticus* S4D258. Structural elucidation of these compounds was successfully achieved by comprehensive analysis of high‐resolution electrospray ionization mass spectrometry (HR‐ESIMS), nuclear magnetic resonance spectroscopy (NMR), and Marfey's method. Notably, actinomycins H_1_–H_5_ (**1**–**5**) contain *L*‐serine (*L*‐Ser), *D*‐2‐aminobutyric acid (*D*‐2‐Aba), and *L*‐2‐methylaminobutyric acid (*L*‐2‐MeAba) residues, whereas actinomycin H_6_ (**6**) comprises a 5*H*‐oxazolo[4,5‐*b*]phenoxazine chromophore. Actinomycins H_7_–H_15_ (**7**–**15**) are nine truncated actinomycin analogs only containing one pentapeptidolactone unit. Actinomycins H_1_ (**1**) and H_9_ (**9**) exhibited inhibitory activities against the proliferation of triple‐negative breast cancer MDA‐MB‐231 cells with IC50 values of 28.00 nM and 4.34 µM, respectively. Mechanistic investigation revealed that the above two actinomycin analogs not only induce S‐phase arrest, mitochondrial membrane potential depolarization, and apoptosis, but also trigger ferroptosis, as revealed by an increase in the level of reactive oxygen species, lipid peroxidation, and malondialdehyde. Collectively, our findings enriched the structural diversity of natural actinomycins and inspired the re‐examination of actinomycin analogs as anticancer leads against triple‐negative breast cancer.

## Graphical abstract

Fifteen new actinomycin analogs, named actinomycins H_1_–H_15_, are identified from *S. polyantibioticus* S4D258. Actinomycins H_7_–H_15_ are truncated actinomycin analogs. Actinomycins H_1_–H_6_ and H_9_ exhibit significant antiproliferative activities against both MDA‐MB‐231 and 4T1 TNBC cells. Mechanistic investigations reveal that actinomycins H_1_ and H_9_ induce S‐phase arrest, apoptosis, and mitochondrial membrane potential disruption.

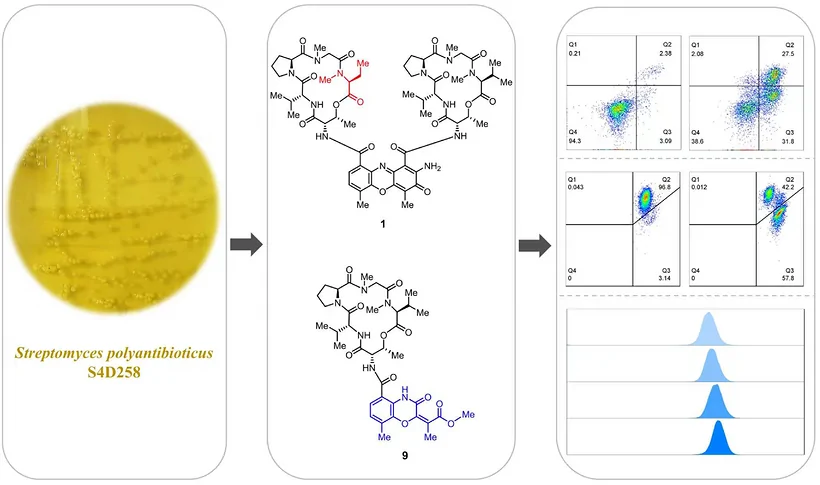

## 1 Introduction

Triple‐negative breast cancer (TNBC) accounts for 15% to 20% of breast cancers [1]. Both aggressive pathology and metastatic tendency indicate the worse prognosis of TNBC, compared with other subtypes of breast cancer [2]. Due to the absence of reliable biomarkers, the clinical treatment of TNBC still relies on cytotoxic agents, including anthracyclines, taxanes, and platinum compounds [3, 4]. However, the clinical benefits of cytotoxic drugs are often curtailed by the emergence of drug resistance, underscoring the urgent need for the development of new drugs with high efficacy, low toxicity, and new mechanism of action [5]. During the recent years, 4‐thiazolidinone, azo‐ether and azo‐sulfone compounds, and thiazolidin‐4‐one derivatives have been synthesized for the development of new anticancer drug leads [6, 7, 8].

Actinomycins, predominantly produced by *Streptomyces*, are a family of chromopeptide antibiotics, of which the molecule consists of three parts, viz., a conserved tricyclic phenoxazinone core and two pentapeptidolactone loops (α‐ and β‐ring), connected by two amide bonds [9, 10]. These chromopeptide antibiotics exhibited potent antitumor and antibacterial activities. In double‐stranded DNA, the chromophore core of actinomycin inserts between G–C and C–G base pairs, whereas the two pentapeptidolactone loops occupy the minor groove. Collectively, the above modes of action form a stable DNA–ligand complex to block transcription [11]. Actinomycin D, a representative anticancer member of the actinomycin family, remains the cornerstone of combination chemotherapy for malignancies, such as gestational trophoblastic neoplasia, rhabdomyosarcoma, Wilms’ tumor, and Ewing's sarcoma [12, 13, 14, 15]. However, despite its clinical efficacy, dose–limiting toxicity and drug resistance restrict the widespread clinical application of actinomycin D [16, 17]. The structural diversity–oriented natural discovery of actinomycin analogs, combined with rational structural redesign, provides a feasible approach to discover highly effective and low‐toxic anticancer drugs [18].

Since the discovery of the first actinomycin member in 1940, nearly 40 members, all sharing a conserved phenoxazinone chromophore, have been reported. The structural diversity of these members originates from difference in amino acid residue composition of the two pentapeptidolactone rings [19, 20]. Notably, hemiactinomycin, an unusual truncated actinomycin analog containing only a single pentapeptidolactone unit, is an exception, indicating the nonconservation of the canonical framework comprising two pentapeptidolactone units [21]. However, the architectural plasticity of truncated actinomycins remains underexplored.

To find new anti‐TNBC compounds from marine actinomycetes, 15 new actinomycin analogs, named actinomycins H_1_–H_15_ (**1**–**15**, Figure 1), were obtained from a marine‐derived *Streptomyces polyantibioticus* S4D258. These newly obtained actinomycin analogs feature pentapeptidolactone loops incorporating atypical amino acid residues (e.g., 4‐hydroxyproline (Hyp), Ser, 2‐Aba, and 2‐MeAba) or truncated actinomycins containing only a pentapeptidolactone unit. Notably, actinomycins H_1_ (**1**) and H_9_ (**9**) inhibit MDA‐MB‐231 cell proliferation and induce S‐phase arrest. Further mechanistic investigation revealed that the above two actinomycin analogs induce both apoptosis and ferroptosis, highlighting the potential of **1** and **9** as lead structures for novel anti‐TNBC agents. The structure elucidation and anti‐TNBC activities of fifteen actinomycin analogs (**1**–**15**) are reported, along with the anticancer mechanistic investigation of actinomycins H_1_ (**1**) and H_9_ (**9**).

**FIGURE 1:**
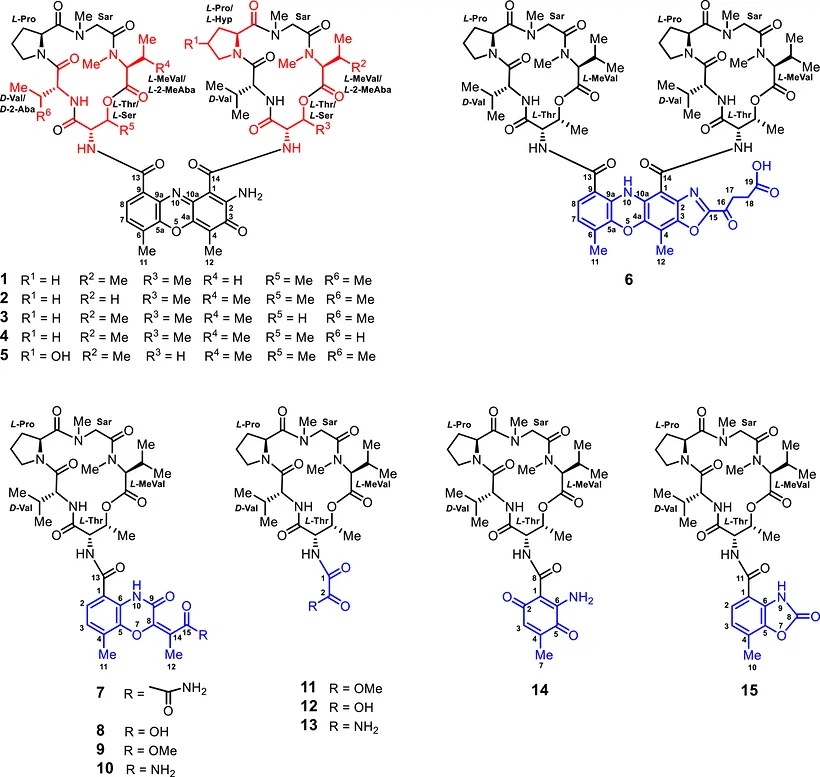
Chemical structures of compounds **1**–**15**.

## 2 Results and Discussion

Compound **1**, a red amorphous powder, had the molecular formula C_61_H_84_N_12_O_16_ as deduced by analysis of the positive HR‐ESIMS ion at *m/z* 1241.6220 (calcd for [M + H]^+^, 1241.6201) (Figure S2). The NMR data of **1** indicated the presence of a phenoxazinone chromophore and two pentapeptidolactone moieties (Tables 1 and 3; Figures S3–S9). The extended sequence of amino acid residues in the α‐pentapeptidolactone ring of **1** consists of valine (Val), proline (Pro), sarcosine (Sar), 2‐MeAba, and threonine (Thr), whereas the β‐pentapeptidolactone ring comprises Val, Pro, Sar, *N*‑methylvaline (MeVal), and Thr. ^1^H–^1^H COSY correlations between H‐2/H_2_‐3 and H_2_‐3/H_3_‐4 within the 2‐MeAba residue, along with HMBC correlations between H‐2/N‐Me, H‐2/C‐1, H‐2/C‐3, H_3_‐4/C‐2, H_3_‐4/C‐3, and N‐Me/C‐2, established the presence of the 2‐MeAba residue unit in the α‐ring (Figure 2). The residues were assigned as *D*‐Val, *L*‐MeVal, *L*‐2‐MeAba, *L*‐Thr, *L*‐Pro, and non‐chiral Sar by Marfey's analyses (Figures S10–S13). The characteristic UV_max_ absorption of **1** closely resembled that of actinomycin D (UV_max_ 239 and 442 nm in **1 **vs. UV_max_ 241 and 442 nm in actinomycin D), indicating the preservation of the phenoxazinone chromophore scaffold [22]. ^1^H–^1^H COSY correlations between H‐7/H‐8, along with HMBC correlations between H‐7/C‐5a, H‐7/C‐9, H‐8/C‐6, H‐8/C‐9a, H‐8/C‐13, H_3_‐11/C‐5a, H_3_‐11/C‐6, H_3_‐11/C‐7, H_3_‐12/C‐3, H_3_‐12/C‐4, and H_3_‐12/C‐4a, established a 2‐amino‐4,6‐dimethyl‐3*H*‐phenoxazin‐3‐one chromophore (Figure 2), consistent with that of actinomycin D [22]. The connection of α‐ and β‐pentapeptidolactone rings to the phenoxazinone chromophore of **1** was confirmed by HMBC cross‐peaks from NHs of two *L*‐Thr residues to C‐13 and C‐14, respectively. Collectively, the above findings established the structure of **1**, named actinomycin H_1_, which represents a new actinomycin analog incorporating an *L*‐2‐MeAba residue in the α‐ring.

**TABLE 1:** ^13^C NMR data for compounds **1–6** (150 MHz, *δ*_C_ in ppm).

NO.	Pentapeptidolactone (α‐ring, *δ*_C_)						NO.	Pentapeptidolactone (β‐ring, *δ*_C_)					
	1^a^	2^a^	3^a^	4^a^	5^a^	6^b^		1^a^	2^a^	3^a^	4^a^	5^a^	6^b^
Thr/ 1	168.4 qC	168.6 qC	168.7 qC	168.6 qC	169.2 qC	169.0 qC	Thr/ 1	169.1 qC	168.9 qC	169.2 qC	169.0 qC	168.7 qC	169.1 qC
Ser 2	55.4 CH	55.4 CH	53.3 CH	55.4 CH	55.5 CH	53.8 CH	Ser 2	55.0 CH	55.0 CH	55.0 CH	55.0 CH	52.9 CH	55.0 CH
3	75.6 CH	75.2 CH	65.2 CH_2_	75.3 CH	75.3 CH	71.8 CH	3	75.2 CH	75.6 CH	75.1 CH	75.1 CH	65.5 CH_2_	72.1 CH
4	18.0 CH_3_	17.6 CH_3_		18.0 CH_3_	17.8 CH_3_	16.8 CH_3_	4	17.3 CH_3_	17.7 CH_3_	17.9 CH_3_	17.6 CH_3_		16.3 CH_3_
Val/ 1	173.5 qC	173.4 qC	173.2 qC	173.3 qC	172.9 qC	170.5 qC	Val 1	173.4 qC	173.4 qC	173.3 qC	173.5 qC	172.9 qC	170.6 qC
2‐Aba 2	59.0 CH	59.0 CH	58.9 CH	54.2 CH	58.9 CH	57.3 CH	2	58.9 CH	58.8 CH	58.9 CH	58.8 CH	58.0 CH	57.3 CH
3	31.6 CH	31.7 CH	31.7 CH	26.2 CH	31.7 CH	30.0 CH	3	32.0 CH	31.9 CH	31.6 CH	32.0 CH	31.3 CH	30.1 CH
4	19.4 CH_3_	19.4 CH_3_	19.4 CH_3_	10.1 CH_3_	19.4 CH_3_	19.0 CH_3_	4	19.5 CH_3_	19.4 CH_3_	19.5 CH_3_	19.4 CH_3_	19.4 CH_3_	19.0 CH_3_
5	19.1 CH_3_	19.2 CH_3_	19.1 CH_3_		19.0 CH_3_	18.5 CH_3_	5	19.2 CH_3_	19.2 CH_3_	19.1 CH_3_	19.2 CH_3_	19.0 CH_3_	18.5 CH_3_
Pro 1	173.9 qC	173.6 qC	173.5 qC	173.6 qC	173.2 qC	172.9 qC	Pro/ 1	173.5 qC	173.9 qC	173.8 qC	173.9 qC	173.8 qC	172.9 qC
2	56.4 CH	56.3 CH	56.3 CH	56.3 CH	56.6 CH	54.6 CH	Hyp 2	56.4 CH	56.6 CH	56.3 CH	56.5 CH	56.3 CH	54.7 CH
3	31.4 CH_2_	31.1 CH_2_	31.2 CH_2_	31.4 CH_2_	31.1 CH_2_	30.9 CH_2_	3	31.1 CH_2_	31.4 CH_2_	31.3 CH_2_	31.3 CH_2_	41.0 CH_2_	31.1 CH_2_
4	23.0 CH_2_	23.2 CH_2_	23.1 CH_2_	23.1 CH_2_	23.1 CH_2_	22.6 CH_2_	4	23.2 CH_2_	23.0 CH_2_	23.4 CH_2_	23.3 CH_2_	69.6 CH	22.7 CH_2_
5	47.8 CH_2_	47.5 CH_2_	47.8 CH_2_	47.8 CH_2_	47.7 CH_2_	46.3 CH_2_	5	47.5 CH_2_	47.7 CH_2_	47.5 CH_2_	47.5 CH_2_	55.2 CH_2_	46.3 CH_2_
Sar 1	166.6 qC	166.5 qC	166.5 qC	167.7 qC	166.5 qC	166.9 qC	Sar 1	166.7 qC	166.6 qC	166.4 qC	167.8 qC	166.1 qC	167.0 qC
2	51.6 CH_2_	51.6 CH_2_	51.6 CH_2_	51.5 CH_2_	51.4 CH_2_	51.1 CH_2_	2	51.6 CH_2_	51.5 CH_2_	51.5 CH_2_	51.5 CH_2_	51.5 CH_2_	51.1 CH_2_
N‐Me	34.9 CH_3_	35.0 CH_3_	35.1 CH_3_	35.2 CH_3_	35.1 CH_3_	34.2 CH_3_	N‐Me	35.1 CH_3_	35.0 CH_3_	34.9 CH_3_	35.4 CH_3_	35.3 CH_3_	34.3 CH_3_
MeVal/ 1	168.7 qC	167.7 qC	168.4 qC	166.6 qC	167.6 qC	168.0 qC	MeVal/ 1	167.8 qC	168.8 qC	167.7 qC	166.7 qC	169.1 qC	168.0 qC
2‐MeAba 2	67.1 CH	71.6 CH	71.5 CH	71.5 CH	70.8 CH	69.5 CH	2‐MeAba 2	71.4 CH	66.9 CH	71.3 CH	71.5 CH	71.5 CH	69.5 CH
3	21.4 CH	27.0 CH	27.1 CH	27.1 CH	28.0 CH	26.3 CH	3	27.0 CH	21.5 CH	27.4 CH	27.0 CH	27.1 CH	26.4 CH
4	11.1 CH_3_	21.9 CH_3_	22.0 CH_3_	21.8 CH_3_	22.0 CH_3_	20.9 CH_3_	4	21.8 CH_3_	11.1 CH_3_	21.8 CH_3_	21.8 CH_3_	21.8 CH_3_	21.0 CH_3_
5		19.3 CH_3_	19.2 CH_3_	19.2 CH_3_	19.3 CH_3_	18.6 CH_3_	5	19.3 CH_3_		19.3 CH_3_	19.2 CH_3_	19.2 CH_3_	18.7 CH_3_
N‐Me	38.9 CH_3_	39.5 CH_3_	39.5 CH_3_	39.4 CH_3_	39.5 CH_3_	38.5 CH_3_	N‐Me	39.4 CH_3_	38.7 CH_3_	39.5 CH_3_	39.4 CH_3_	39.5 CH_3_	38.6 CH_3_
	Chromophore (*δ*_C_)												
1	101.9 qC	102.0 qC	102.2 qC	101.9 qC	99.5 qC	99.9 qC	9a	129.3 qC	129.3 qC	130.0 qC	129.3 qC	129.2 qC	129.8 qC
2	147.8 qC	147.8 qC	147.8 qC	147.8 qC	149.3 qC	132.4 qC	10a	146.1 qC	146.0 qC	146.0 qC	146.0 qC	146.5 qC	133.9 qC
3	179.3 qC	179.3 qC	179.1 qC	179.3 qC	179.1 qC	144.4 qC	11	15.2 CH_3_	15.2 CH_3_	15.4 CH_3_	15.2 CH_3_	15.2 CH_3_	14.5 CH_3_
4	113.6 qC	113.6 qC	113.7 qC	113.6 qC	113.8 qC	111.7 qC	12	7.9 CH_3_	8.0 CH_3_	8.0 CH_3_	8.0 CH_3_	8.0 CH_3_	8.6 CH_3_
4a	145.3 qC	145.2 qC	145.2 qC	145.3 qC	145.7 qC	142.9 qC	13	166.3 qC	166.2 qC	164.5 qC	166.1 qC	166.8 qC	166.4 qC
5a	140.6 qC	140.6 qC	140.7 qC	140.6 qC	140.6 qC	140.2 qC	14	166.3 qC	166.5 qC	166.3 qC	166.5 qC	166.7 qC	164.9 qC
6	127.7 qC	127.7 qC	128.6 qC	127.7 qC	128.5 qC	127.7 qC	15						155.7 qC
7	130.4 CH	130.4 CH	130.1 CH	130.4 CH	130.4 CH	122.3 CH	16						186.1 qC
8	126.1 CH	126.1 CH	126.9 CH	126.1 CH	126.4 CH	122.1 CH	17						33.5 CH_2_
9	132.8 qC	132.8 qC	130.1 qC	132.7 qC	131.3 qC	113.6 qC	18						27.1 CH_2_
							19						173.4 qC

^a^ Measured in CDCl_3_.

^b^ Measured in DMSO‐*d*_6._

**FIGURE 2:**
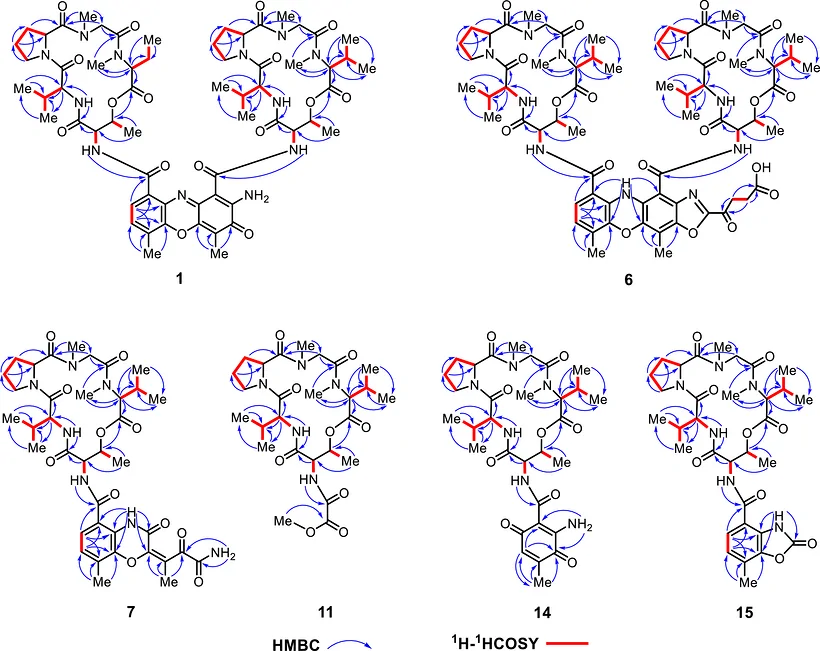
^1^H–^1^H COSY and key HMBC correlations of compounds **1**, **6**, **7**, **11**, **14**, and **15**.

Compound **2** provided the molecular formula C_61_H_84_N_12_O_16_ as determined by the HR‐ESIMS data (Figure S15). The NMR data of **2** indicated the presence of a phenoxazinone chromophore and two pentapeptidolactone moieties (Tables 1 and 3; Figures S16–S22). The extended sequence of amino acid residues in the α‐pentapeptidolactone ring of **2** consists of Val, Pro, Sar, MeVal, and Thr, whereas the β‐pentapeptidolactone ring comprises Val, Pro, Sar, 2‐MeAba, and Thr. ^1^H–^1^H COSY correlations between H‐2/H_2_‐3 and H_2_‐3/H_3_‐4 within the 2‐MeAba residue, along with HMBC correlations between H‐2/N‐Me, H‐2/C‐1, H‐2/C‐3, H_3_‐4/C‐2, H_3_‐4/C‐3, and N‐Me/C‐2, established the 2‐MeAba residue in the β‐ring. The residues were assigned as *D*‐Val, *L*‐MeVal, *L*‐2‐MeAba, *L*‐Thr, *L*‐Pro, and non‐chiral Sar by Marfey's analyses (Figure S23). The characteristic UV_max_ absorption and detailed NMR analyses established the chromophore of **2** as identical to that of **1**. Collectively, the above findings established the structure of **2**, named actinomycin H_2_, as a new actinomycin analog incorporating an *L*‐2‐MeAba residue in the β‐ring. The positioning of the *L*‐2‐MeAba residue within the α‐ versus β‐pentapeptidolactone ring might be derived from the substrate promiscuity‌ of nonribosomal peptide synthetase (NRPS).

Compound **3** provided the molecular formula C_61_H_84_N_12_O_16_ as determined by the HR‐ESIMS data (Figure S25). The NMR data of **3** indicated the presence of a phenoxazinone chromophore and two pentapeptidolactone moieties (Tables 1 and 3; Figures S26–S32). The extended sequence of amino acid residues in the α‐pentapeptidolactone ring of **3** consists of Val, Pro, Sar, MeVal, and Ser, whereas the β‐pentapeptidolactone ring comprises Val, Pro, Sar, MeVal, and Thr. ^1^H–^1^H COSY correlations between NH‐2/H‐2 within the Ser residue, together with HMBC correlations between H‐2/C‐1 and H_2_‐3/C‐2, indicated the presence of the Ser residue unit in the α‐ring. The residues were assigned as *D*‐Val, *L*‐MeVal, *L*‐Ser, *L*‐Thr, *L*‐Pro, and non‐chiral Sar by Marfey's analyses (Figures S33–S34). Collectively, the above findings established the structure of **3**, named actinomycin H_3_, as a new actinomycin analog incorporating an *L*‐Ser residue in the α‐ring.

Compound **4** afforded the molecular formula C_61_H_84_N_12_O_16_ as determined by the analysis of the HR‐ESIMS data (Figure S36). The NMR data of **4** indicated the presence of a phenoxazinone chromophore and two pentapeptidolactone moieties (Tables 1 and 3; Figures S37–S43). The extended sequence of amino acid residues in the α‐pentapeptidolactone ring of **4** consists of 2‐Aba, Pro, Sar, MeVal, and Thr, whereas the β‐pentapeptidolactone ring comprises Val, Pro, Sar, MeVal, and Thr. ^1^H–^1^H COSY correlations between NH‐2/H‐2, H‐2/H_2_‐3, and H_2_‐3/H_3_‐4 within the 2‐Aba residue, along with HMBC correlations between NH‐2/C‐2, NH‐2/C‐3, H‐2/C‐1, H‐2/C‐3, H‐2/C‐4, H_2_‐3/C‐1, H_2_‐3/C‐2, H_2_‑3/C‐4, H_3_‐4/C‑2, and H_3_‑4/C‐3, indicated the presence of the 2‐Aba residue unit in the α‐ring. The residues were assigned as *D*‐2‐Aba, *D*‐Val, *L*‐MeVal, *L*‐Thr, *L*‐Pro, and non‐chiral Sar by Marfey's analyses (Figures S44–S45). Collectively, the above findings established the structure of **4**, named actinomycin H_4_, as a new actinomycin analog incorporating a *D*‐2‐Aba residue in the α‐ring.

Compound **5** afforded the molecular formula C_61_H_84_N_12_O_17_ as determined by the HR‐ESIMS data (Figure S47). The NMR data of **5** indicated the presence of a phenoxazinone chromophore and two pentapeptidolactone moieties (Tables 1 and 3; Figures S48–S54). The extended sequence of amino acid residues in the α‐pentapeptidolactone ring of **5** consists of Val, Pro, Sar, MeVal, and Thr, whereas the β‐pentapeptidolactone ring comprises Val, Hyp, Sar, MeVal, and Ser. ^1^H–^1^H COSY correlations between H‐2/H_2_‐3, H_2_‐3/H‐4, and H‐4/H_2_‐5 within the Hyp residue, along with HMBC correlations between H‐2/C‐3, H‐2/C‐4, H_2_‐3/C‐2, H_2_‐3/C‐4, H_2_‐5/C‐3, and H_2_‐5/C‐4, indicated the Hyp residue unit in the β‐ring. Likewise, ^1^H–^1^H COSY correlations between NH‐2/H‐2 within the Ser residue, along with HMBC correlations between H‐2/C‐1 and H_2_‐3/C‐2, corroborated the Ser residue unit in the β‐ring. The residues were assigned as *D*‐Val, *L*‐MeVal, *L*‐Ser, *L*‐Thr, *L*‐Hyp, *L*‐Pro, and non‐chiral Sar by Marfey's analyses (Figures S55–S56). Collectively, the above findings established the structure of **5**, named actinomycin H_5_, as a new actinomycin analog incorporating *L*‐Ser and *L*‐Hyp residues in the β‐ring.

Compound **6** had the molecular formula C_67_H_90_N_12_O_19_ as deduced by analysis of the HR‐ESIMS data (Figure S58). The NMR data of **6** indicated the presence of a tetracyclic chromophore and two pentapeptidolactone moieties (Tables 1 and 3; Figures S59–S65). The extended sequence of amino acid residues in both α‐ and β‐pentapeptidolactone rings of **6** consists of Val, Pro, Sar, MeVal, and Thr. The residues were assigned as *D*‐Val, *L*‐MeVal, *L*‐Thr, *L*‐Pro, and non‐chiral Sar by Marfey's analyses (Figure S66). ^1^H–^1^H COSY correlations between H‐7/H‐8, along with HMBC correlations between H‐7/C‐5a, H‐7/C‐9, H‐8/C‐6, H‐8/C‐9a, H‐8/C‐13, H_3_‐11/C‐5a, H_3_‐11/C‐6, and H_3_‐11/C‐7, indicated a monomethyl‐substituted benzene ring. HMBC cross‐peaks from an exchangeable proton (*δ*_H_ 12.04 s) to C‐1, C‐4a, C‐5a, and C‐9 revealed the 10‐NH group. Those from the methyl protons (*δ*_H_ 2.25 s) to C‐3, C‐4, and C‐4a corroborated the 12‐Me assignment (Figure 2). The downfield‐shifted C‐15 (*δ*_C_ 155.7 ppm) revealed the presence of a C‐15 imine group. Thus, the tetracyclic chromophore of **6** was concluded to be 5*H*‐oxazolo[4,5‐*b*]phenoxazine. This scaffold is analogous to that of the previously reported actinomycin D_1_ [23]. Notably, the UV_max_ absorption of **6** differed from that of actinomycin D_1_ (UV_max_ 255 and 450 nm in **6** vs. 252 and 409 nm in actinomycin D_1_) and a molecular weight increase of 100 Da in **6** compared to that of actinomycin D_1_ suggested an additional side chain on the oxazolo‐phenoxazine scaffold. ^1^H–^1^H COSY correlations between H_2_‐17/H_2_‐18, along with HMBC cross‐peaks between H_2_‐17/C‐16, H_2_‐17/C‐19, H_2_‐18/C‐16, and H_2_‐18/C‐19, supported a 4‐oxobutanoic acid unit (Figure 2). Collectively, the above findings established the structure of **6**, named actinomycin H_6_, as a new actinomycin analog featuring a 5*H*‐oxazolo[4,5‐*b*]phenoxazine backbone and bearing a 4‐oxobutanoic acid side chain.

Compound **7** had the molecular formula C_37_H_49_N_7_O_11_ as established by the HR‐ESIMS data (Figure S68). The NMR data of **7** indicated the presence of a bicyclic chromophore and a pentapeptidolactone moiety (Tables 2 and 4; Figures S69–S75). ^1^H–^1^H COSY correlations between H‐2/H‐3, along with HMBC cross‐peaks between H‐2/C‐4, H‐2/C‐6, H‐2/C‐13, H‐3/C‐1, H‐3/C‐5, H_3_‐11/C‐3, H_3_‐11/C‐4, H_3_‐11/C‐5, indicated a monomethyl‐substituted benzene ring. HMBC cross‐peaks from an exchangeable proton (*δ*_H_ 11.91 s) to C‐1, C‐5, C‐6, C‐8, and C‐9 revealed the 10‐NH group. The downfield‐shifted C‐9 (*δ*_C_ 156.2 ppm) revealed a C‐9 carbonyl group. Collectively, the above findings corroborated the presence of a morpholin‐3‐one motif. HMBC correlations between H_3_‐12/C‐8, H_3_‐12/C‐14, H_3_‐12/C‐15, NH_2_‐16/C‐15, and NH_2_‐16/C‐16 corroborated the presence of a 3‐methyl‐2‐oxobut‐3‐enamide side chain attached at C‐8 of the morpholin‐3‐one motif (Figure 2). Thus, the chromophore of **7** was concluded to be an 8‐methyl‐2*H*‐benzo[*b*][1,4]oxazin‐3(4*H*)‐one scaffold bearing a 3‐methyl‐2‐oxobut‐3‐enamide side chain. The extended sequence of amino acid residues in the pentapeptidolactone ring of **7** consists of Val, Pro, Sar, MeVal, and Thr. The residues were assigned as *D*‐Val, *L*‐MeVal, *L*‐Thr, *L*‐Pro, and non‐chiral Sar by Marfey's analyses (Figure S76). Collectively, the above findings established the structure of **7**, named actinomycin H_7_, as a new actinomycin analog featuring only one pentapeptidolactone moiety and a benzoxazinone chromophore scaffold.

**TABLE 2:** ^13^C NMR data for compounds **7–15** (150 MHz, *δ*_C_ in ppm).

NO.	Pentapeptidolactone (δ_C_)								
	7^a^	8^a^	9^a^	10^a^	11^a^	12^a^	13^a^	14^a^	15^a^
Thr 1	171.8 qC	171.3 qC	171.4 qC	170.9 qC	167.9 qC	168.0 qC	168.0 qC	169.1 qC	169.5 qC
2	56.6 CH	56.4 CH	56.3 CH	56.1 CH	54.1 CH	54.1 CH	54.0 CH	53.4 CH	54.5 CH
3	70.7 CH	71.0 CH	70.9 CH	70.9 CH	72.1 CH	72.1 CH	72.1 CH	72.4 CH	71.9 CH
4	16.2 CH_3_	16.4 CH_3_	16.2 CH_3_	16.3 CH_3_	16.3 CH_3_	16.4 CH_3_	16.4 CH_3_	16.6 CH_3_	16.3 CH_3_
Val 1	170.2 qC	170.4 qC	170.3 qC	170.3 qC	170.4 qC	170.4 qC	170.4 qC	170.7 qC	170.4 qC
2	57.9 CH	57.9 CH	57.8 CH	57.7 CH	57.2 CH	57.2 CH	57.2 CH	57.3 CH	57.4 CH
3	30.0 CH	30.1 CH	30.0 CH	30.0 CH	30.0 CH	30.0 CH	30.0 CH	30.0 CH	30.0 CH
4	18.9 CH_3_	19.1 CH_3_	18.9 CH_3_	18.9 CH_3_	18.9 CH_3_	18.9 CH_3_	18.9 CH_3_	19.0 CH_3_	19.0 CH_3_
5	18.5 CH_3_	18.6 CH_3_	18.5 CH_3_	18.5 CH_3_	18.4 CH_3_	18.4 CH_3_	18.4 CH_3_	18.5 CH_3_	18.5 CH_3_
Pro 1	173.1 qC	173.4 qC	173.2 qC	173.3 qC	172.8 qC	172.9 qC	172.9 qC	172.9 qC	172.9 qC
2	55.4 CH	55.4 CH	55.4 CH	55.2 CH	54.8 CH	54.8 CH	54.8 CH	54.5 CH	54.7 CH
3	31.1 CH_2_	31.5 CH_2_	31.2 CH_2_	31.4 CH_2_	31.2 CH_2_	31.2 CH_2_	31.2 CH_2_	31.3 CH_2_	31.1 CH_2_
4	22.5 CH_2_	22.8 CH_2_	22.6 CH_2_	22.7 CH_2_	22.7 CH_2_	22.8 CH_2_	22.8 CH_2_	22.7 CH_2_	22.7 CH_2_
5	46.5 CH_2_	46.6 CH_2_	46.5 CH_2_	46.5 CH_2_	46.4 CH_2_	46.4 CH_2_	46.4 CH_2_	46.4 CH_2_	46.3 CH_2_
Sar 1	167.2 qC	167.1 qC	167.0 qC	167.0 qC	167.0 qC	167.1 qC	167.1 qC	167.1 qC	166.8 qC
2	50.9 CH_2_	51.1 CH_2_	50.6 CH_2_	51.0 CH_2_	51.0 CH_2_	51.1 CH_2_	51.1 CH_2_	51.2 CH_2_	51.0 CH_2_
N‐Me	34.2 CH_3_	34.4 CH_3_	34.2 CH_3_	34.3 CH_3_	34.3 CH_3_	34.3 CH_3_	34.3 CH_3_	34.3 CH_3_	34.3 CH_3_
MeVal 1	168.1 qC	168.1 qC	168.0 qC	168.0 qC	167.8 qC	167.9 qC	168.0 qC	168.1 qC	168.0 qC
2	69.3 CH	69.4 CH	69.3 CH	69.3 CH	69.2 CH	69.2 CH	69.1 CH	69.4 CH	69.4 CH
3	26.4 CH	26.6 CH	26.6 CH	26.4 CH	26.5 CH	26.5 CH	26.5 CH	26.1 CH	26.4 CH
4	21.2 CH_3_	21.2 CH_3_	21.1 CH_3_	21.1 CH_3_	20.9 CH_3_	20.9 CH_3_	20.8 CH_3_	20.8 CH_3_	21.0 CH_3_
5	18.6 CH_3_	18.7 CH_3_	18.6 CH_3_	18.6 CH_3_	18.7 CH_3_	18.7 CH_3_	18.7 CH_3_	18.6 CH_3_	18.7 CH_3_
N‐Me	38.2 CH_3_	38.1 CH_3_	37.9 CH_3_	38.1 CH_3_	38.4 CH_3_	38.4 CH_3_	38.3 CH_3_	38.4 CH_3_	38.4 CH_3_
Chromophore (*δ*_C_)									
1	122.3 qC	121.0 qC	121.3 qC	120.4 qC	156.9 qC	157.9 qC	159.7 qC	97.5 qC	114.3 qC
2	121.5 CH	121.4 CH	121.5 CH	121.1 CH	160.8 qC	161.5 qC	161.2 qC	183.8 qC	121.2 CH
3	124.6 CH	124.3 CH	124.3 CH	123.9 CH	53.1 CH_3_			137.4 CH	122.6 CH
4	126.7 qC	127.0 qC	126.8 qC	126.8 qC				141.0 qC	122.7 qC
5	138.6 qC	139.1 qC	138.7 qC	139.5 qC				182.4 qC	142.1 qC
6	122.6 qC	123.7 qC	123.3 qC	124.1 qC				153.2 qC	130.0 qC
7								14.5 CH_3_	
8	139.2 qC	137.8 qC	138.8 qC	136.8 qC				167.5 qC	154.1 qC
9	156.2 qC	155.3 qC	155.0 qC	155.7 qC					
10									14.3 CH_3_
11	15.0 CH_3_	15.1 CH_3_	15.0 CH_3_	15.1 CH_3_					164.8 qC
12	13.8 CH_3_	13.9 CH_3_	13.5 CH_3_	14.8 CH_3_					
13	166.4 qC	166.7 qC	166.5 qC	166.7 qC					
14	121.3 qC	119.4 qC	117.4 qC	123.1 qC					
15	191.5 qC	169.6 qC	168.8 qC	169.9 qC					
16	163.0 qC		52.1 qC						

^a^ Measured in DMSO‐*d*_6__._

**TABLE 3:** ^1^H NMR data for compounds **1–6** (600 MHz, *δ*_H_ in ppm, *J* in Hz).

Position	1^a^	2^a^	3^a^	4^a^	5^a^	6^b^
Pentapeptidolactone (α‐ring, *δ*_H_)						
Thr/ 2	4.48 dd (6.3, 2.5)	4.51 d (4.6)	4.25 br s	4.48 d (4.3)	4.95 dd (7.2, 2.3)	4.99 d (9.6)
Ser 3	5.13 m	5.22 m	4.76 d (11.0) 4.32 d (11.2)	5.19 m	5.28 m	5.28 m
4	1.26 d (5.7)	1.26 d (4.3)		1.26 d (5.8)	1.30 d (6.1)	1.29 d (6.2)
NH	7.17 d (6.5)	7.18 d (6.4)	8.28 d (3.3)	7.27 d (6.7)	7.24 d (7.4)	7.27 d (8.7)
Val/ 2	3.58 m	3.53 m	3.52 m	3.81 m	3.61 m	3.52 m
2‐Aba 3	2.22 m	2.20 m	2.17 m	1.84 m	2.12 m	1.89 m
4	0.91 d (6.5)	0.88 d (6.2)	0.90 d (5.3)	0.98 overlapped	0.95 d (7.0)	0.71 d (6.2)
5	1.13 d (6.7)	1.12 d (5.4)	1.14 d (6.5)		1.17 d (6.7)	0.95 d (6.7)
NH	8.10 d (5.5)	8.13 d (5.7)	8.18 d (5.1)	8.04 d (5.7)	7.75 d (6.3)	8.46 d (5.0)
Pro 2	5.98 d (9.1)	6.03 d (9.0)	6.01 d (9.1)	5.92 d (9.0)	6.08 d (9.1)	6.26 d (7.5)
3	2.96, 1.86 m	2.94, 1.80 m	2.72, 2.16 m	2.99, 2.67 m	2.57, 1.82 m	2.09, 1.74 m
4	2.28, 2.08 m	2.29, 2.04 m	2.24, 2.16 m	2.30, 2.11 m	2.22, 2.07 m	1.91, 1.66 m
5	3.82, 3.73 m	3.95, 3.73 m	3.77, 3.72 m	3.85, 3.77 m	3.78, 3.76 m	3.48, 3.27 m
Sar 2	4.68 d (17.2) 3.66 d (17.1)	4.71 d (16.9) 3.63 d (16.8)	4.67 d (17.5) 3.61 d (17.7)	4.76 d (17.1) 3.64 d (17.2)	4.68 d (17.4) 3.64 d (17.0)	4.81 d (17.9) 4.11 d (17.7)
N‐Me	2.88 s	2.86 s	2.87 s	2.90 s	2.91 s	2.77 s
MeVal/ 2	2.98 m	2.66 overlapped	2.69 overlapped	2.69 overlapped	2.85 d (8.8)	3.23 d (9.4)
2‐MeAba 3	1.97 m	2.67 m	2.68 m	2.67 m	2.64 m	2.55 m
4	0.83 t (7.3)	0.97 d (5.0)	0.96 d (5.8)	0.97 overlapped	1.02 d (6.5)	0.98 d (6.4)
5		0.74 d (5.3)	0.74 d (6.3)	0.76 d (5.6)	0.78 d (6.8)	0.80 d (6.7)
N‐Me	2.95 s	2.93 s	2.91 s	2.95 s	2.95 s	3.22 s
Pentapeptidolactone (β‐ring, *δ*_H_)						
Thr 2	4.62 dd (6.2, 2.2)	4.60 d (3.8)	4.62 dd (6.5, 2.2)	4.61 d (4.0)	4.18 br s	4.90 d (8.8)
Ser/ 3	5.17 m	5.11 m	5.20 m	5.19 m	4.64 dd (11.8, 2.2) 4.46 d (11.1)	5.17 m
4	1.26 d (5.7)	1.25 d (4.7)	1.25 d (6.4)	1.27 d (6.0)		1.20 d (6.2)
NH	7.79 d (6.0)	7.75 d (5.9)	7.51 d (6.4)	7.81 d (6.2)	8.45 d (3.0)	9.38 d (8.0)
Val 2	3.55 m	3.61 m	3.58 m	3.58 m	3.57 m	3.52 m
3	2.15 m	2.16 m	2.17 m	2.21 m	2.12 m	1.89 m
4	0.89 d (6.9)	0.92 d (6.5)	0.90 d (5.3)	0.94 overlapped	0.89 d (6.4)	0.71 d (6.2)
5	1.11 d (6.4)	1.13 d (4.4)	1.11 d (6.6)	1.14 d (6.2)	1.10 d (6.7)	0.94 d (6.6)
NH	7.95 d (6.0)	7.95 d (5.7)	7.96 d (4.5)	8.07 d (6.0)	8.12 d (4.7)	8.33 d (5.0)
Pro/ 2	6.04 d (9.1)	5.97 d (9.0)	6.05 d (9.1)	6.01 d (9.0)	5.87 t (7.5)	6.26 d (7.5)
Hyp 3	2.65, 1.80 m	2.68, 1.85 m	2.65, 1.83 m	2.99, 2.67 m	2.47, 2.22 m	2.09, 1.74 m
4	2.28, 2.08 m	2.08, 2.27 m	2.30, 2.08 m	2.30, 2.11 m	4.62 m	1.91, 1.66 m
5	3.99, 3.73 m	3.85, 3.73 m	3.96, 3.75 m	3.96, 3.77 m	4.34, 3.86 m	3.48, 3.27 m
Sar 2	4.77 d (17.5) 3.61 d (17.1)	4.74 d (16.9) 3.63 d (16.8)	4.74 d (16.9) 3.65 d (17.4)	4.79 d (17.1) 3.68 d (17.2)	4.73 d (17.2) 3.65 d (17.0)	4.76 d (17.9) 4.08 d (17.7)
N‐Me	2.86 s	2.88 s	2.89 s	2.90 s	2.88 s	2.76 s
MeVal/ 2	2.67 overlapped	2.99 m	2.75 d (9.7)	2.69 overlapped	2.68 overlapped	3.17 d (9.4)
2‐MeAba 3	2.65 m	1.95 m	2.68 m	2.67 m	2.64 m	2.55 m
4	0.95 d (5.9)	0.83 t (7.3)	1.00 d (6.1)	0.98 overlapped	0.97 d (5.8)	0.98 d (6.4)
5	0.73 d (6.0)		0.75 d (6.5)	0.77 d (5.6)	0.75 d (6.3)	0.81 d (6.7)
N‐Me	2.89 s	2.92 s	2.92 s	2.91 s	2.95 s	3.14 s
Chromophore (*δ*_H_)						
7	7.36 d (7.5)	7.37 d (7.7)	7.39 d (7.6)	7.39 d (7.7)	7.35 d (7.6)	6.63 d (8.0)
8	7.64 d (7.7)	7.65 d (7.6)	7.94 d (7.7)	7.68 d (7.7)	7.64 d (7.8)	7.08 d (7.9)
11‐Me	2.55 s	2.55 s	2.57 s	2.58 s	2.54 s	2.08 s
12‐Me	2.25 s	2.25 s	2.26 s	2.28 s	2.24 s	2.25 s
17						3.40 m
18						2.71 m
NH_2_‐2					6.77 s, 9.31 s	
NH‐10						12.04 s
OH‐19						12.39 s

^a^ Measured in CDCl_3_.

^b^ Measured in DMSO‐*d*_6._

**TABLE 4:** ^1^H NMR data for compounds **7–15** (600 MHz, *δ*_H_ in ppm, *J* in Hz).

Position	7^a^	8^a^	9^a^	10^a^	11^a^	12^a^	13^a^	14^a^	15^a^
Pentapeptidolactone (*δ*_H_)									
Thr 2	4.74 dd (8.3, 2.1)	4.76 dd (8.4, 2.4)	4.76 dd (8.4, 2.3)	4.76 dd (8.2, 1.6)	4.72 dd (9.4, 2.1)	4.70 d (9.2)	4.67 dd (9.6, 2.0)	4.90 d (9.6)	4.94 d (8.7)
3	5.21 m	5.20 m	5.20 m	5.18 m	5.09 m	5.10 m	5.10 m	5.09 m	5.18 m
4	1.21 d (6.3)	1.21 d (6.3)	1.21 d (6.4)	1.21 d (6.2)	1.10 d (6.3)	1.11 d (6.0)	1.09 d (6.4)	1.10 d (6.1)	1.20 d (5.7)
NH	8.71 d (8.2)	8.59 d (8.3)	8.64 d (8.3)	8.54 d (8.3)	7.98 d (9.3)	7.87 d (9.3)	7.98 d (9.6)	9.94 d (9.4)	7.70 d (8.7)
Val 2	3.54 m	3.56 m	3.56 m	3.56 m	3.51 m	3.52 m	3.51 m	3.45 m	3.52 m
3	1.85 m	1.88 m	1.86 m	1.88 m	1.85 m	1.86 m	1.86 m	1.87 m	1.88 m
4	0.70 d (6.7)	0.71 d (6.7)	0.71 d (6.6)	0.70 d (6.6)	0.69 d (6.7)	0.69 d (6.5)	0.69 d (6.7)	0.68 d (6.6)	0.69 d (6.6)
5	0.92 d (6.7)	0.93 d (6.6)	0.93 d (6.7)	0.93 d (6.5)	0.90 d (6.5)	0.90 d (6.5)	0.90 d (6.7)	0.89 d (6.5)	0.93 d (6.5)
NH	8.62 d (4.8)	8.61 d (5.1)	8.61 d (4.8)	8.60 d (5.0)	8.45 d (5.7)	8.46 d (5.3)	8.44 d (5.8)	8.38 d (5.8)	8.50 d (4.4)
Pro 2	6.25 dd (8.6, 2.6)	6.30 dd (8.6, 3.5)	6.28 dd (8.6, 2.9)	6.30 dd (8.4, 2.8)	6.16 dd (8.6, 3.1)	6.17 dd (8.3, 2.4)	6.16 dd (8.6, 3.5)	6.24 dd (8.5, 2.7)	6.21 d (6.1)
3	1.99, 1.79 m	2.16, 1.71 m	2.11, 1.70 m	2.19, 1.73 m	2.18, 1.78 m	2.20, 1.77 m	2.22, 1.77 m	2.16, 1.77 m	2.12, 1.76 m
4	1.86, 1.60 m	1.88, 1.61 m	1.88, 1.63 m	1.89, 1.64 m	1.93, 1.70 m	1.94, 1.71 m	1.93, 1.70 m	1.94, 1.69 m	1.92, 1.68 m
5	3.44, 3.23 m	3.51, 3.23 m	3.48, 3.23 m	3.48, 3.24 m	3.48, 3.27 m	3.49, 3.28 m	3.50, 3.26 m	3.47, 3.26 m	3.46, 3.27 m
Sar 2	5.23 d (18.1) 3.98 d (18.3)	5.27 d (18.1) 3.94 d (18.1)	5.20 overlapped 4.01 d (18.1)	5.28 d (18.0) 3.98 d (18.1)	4.78 d (18.0) 4.10 d (17.9)	4.82 d (17.8) 4.10 d (17.9)	4.83 d (17.8) 4.09 d (17.9)	4.83 d (17.8) 4.08 d (17.9)	4.79 d (17.8) 4.12 d (17.8)
N‐Me	2.74 s	2.76 s	2.76 s	2.76 s	2.75 s	2.75 s	2.75 s	2.75 s	2.75 s
MeVal 2	2.92 d (9.3)	2.99 d (9.3)	2.97 d (9.2)	2.99 d (9.3)	3.24 d (9.3)	3.27 d (9.2)	3.29 d (9.3)	3.25 d (9.4)	3.18 overlapped
3	2.49 m	2.50 m	2.48 m	2.50 m	2.49 m	2.50 m	2.49 m	2.50 m	2.52 m
4	0.97 d (6.5)	0.96 d (6.4)	0.97 d (6.5)	0.96 d (6.5)	0.94 d (6.5)	0.95 d (6.2)	0.94 d (6.5)	0.95 d (6.4)	0.97 d (6.5)
5	0.79 d (6.8)	0.78 d (6.8)	0.79 d (6.9)	0.78 d (6.8)	0.78 d (6.9)	0.78 d (6.1)	0.77 d (6.8)	0.78 d (6.7)	0.79 d (6.8)
N‐Me	2.98 s	3.06 s	3.04 s	3.08 s	3.16 s	3.16 s	3.16 s	3.15 s	3.20 s
Chromophore (*δ*_H_)									
2	7.04 d (7.8)	7.08 d (7.8)	7.07 d (7.9)	7.08 d (7.8)					7.50 d (7.9)
3	7.01 d (7.9)	6.99 d (8.0)	6.99 d (7.7)	6.97 d (8.0)	3.80 s			6.61 s	7.05 d (7.9)
7								1.96 s	
10									2.36 s
11	2.34 s	2.32 s	2.32 s	2.31 s					
12	1.99 s	2.03 s	2.03 s	1.99 s					
16			3.72 s						
NH‐9									11.70 s
NH‐10	11.91 s	11.55 s	11.68 s	11.32 s					
NH_2_‐2							8.04 s, 8.30 s		
NH_2_‐6								8.56 s, 10.30 s	
NH_2_‐15				7.09 s, 7.36 s					
NH_2_‐16	7.91 s, 7.59 s								
OH‐15		12.74 s							

^a^ Measured in DMSO‐*d*_6_.

Compound **8** provided the molecular formula C_36_H_48_N_6_O_11_ as determined by the HR‐ESIMS data (Figure S78). The NMR data of **8** indicated the presence of a benzoxazinone chromophore and a pentapeptidolactone moiety (Tables 2 and 4; Figures S79–S85). HMBC cross‐peaks between H_3_‐12/C‐8, H_3_‐12/C‐14, and H_3_‐12/C‐15 corroborated the acrylic acid substituent attached at C‐8 of the morpholin‐3‐one motif. Thus, the chromophore of **8** was concluded to be a benzoxazinone scaffold bearing an acrylic acid side chain. The extended sequence of amino acid residues in the pentapeptidolactone ring of **8** consists of Val, Pro, Sar, MeVal, and Thr. The residues were assigned as *D*‐Val, *L*‐MeVal, *L*‐Thr, *L*‐Pro, and non‐chiral Sar by Marfey's analyses (Figure S86). Collectively, the above findings established the structure of **8**, named actinomycin H_8_, as a new actinomycin analog featuring a benzoxazinone chromophore scaffold bearing an acrylic acid side chain.

Compound **9** afforded the molecular formula C_37_H_50_N_6_O_11_ as determined by the HR‐ESIMS data (Figure S88). The NMR data of **9** indicated the presence of a benzoxazinone chromophore and a pentapeptidolactone moiety (Tables 2 and 4; Figures S89–S95). HMBC cross‐peaks between H_3_‐12/C‐8, H_3_‐12/C‐14, H_3_‐12/C‐15, and H_3_‐16/C‐15 corroborated a methyl acrylate moiety attached at C‐8 of the morpholin‐3‐one motif. Thus, the chromophore of **9** was concluded to be a benzoxazinone scaffold bearing a methyl acrylate side chain. The extended sequence of amino acid residues in the pentapeptidolactone ring of **9** consists of Val, Pro, Sar, MeVal, and Thr. The residues were assigned as *D*‐Val, *L*‐MeVal, *L*‐Thr, *L*‐Pro, and non‐chiral Sar by Marfey's analyses (Figure S96). Collectively, the above findings established the structure of **9**, named actinomycin H_9_, as a new actinomycin analog featuring a benzoxazinone chromophore scaffold bearing a methyl acrylate side chain.

Compound **10** provided the molecular formula C_36_H_49_N_7_O_10_ as determined by analysis of the HR‐ESIMS data (Figure S98). The NMR data of **10** indicated the presence of a benzoxazinone chromophore and a pentapeptidolactone moiety (Tables 2 and 4; Figures S99–S105). HMBC cross‐peaks between H_3_‐12/C‐8, H_3_‐12/C‐14, H_3_‐12/C‐15, and NH_2_‐15/C‐15 corroborated an acrylamide moiety attached at C‐8 of the morpholin‐3‐one motif. Thus, the chromophore of **10** was concluded to be a benzoxazinone scaffold bearing an acrylamide side chain. The extended sequence of amino acid residues in the pentapeptidolactone ring of **10** consists of Val, Pro, Sar, MeVal, and Thr. The residues were assigned as *D*‐Val, *L*‐MeVal, *L*‐Thr, *L*‐Pro, and non‐chiral Sar by Marfey's analyses (Figure S106). Collectively, the above findings established the structure of **10**, named actinomycin H_10_, as a new actinomycin analog featuring a benzoxazinone chromophore scaffold bearing an acrylamide side chain.

Compound **11** had the molecular formula C_26_H_41_N_5_O_9_ as deduced by analysis of the HR‐ESIMS data (Figure S108). The NMR data of **11** indicated a pentapeptidolactone moiety (Tables 2 and 4; Figures S109–S115). The extended sequence of amino acid residues in the pentapeptidolactone ring of **11** consists of Val, Pro, Sar, MeVal, and Thr. HMBC correlation between the Thr NH and C‐1 of the 2‐methoxy‐2‐oxoacetyl moiety and that between the H_3_‐3 and C‐2 revealed the modification of the Thr residue by a 2‐methoxy‐2‐oxoacetyl group (Figure 2). The residues were assigned as *D*‐Val, *L*‐MeVal, *L*‐Thr, *L*‐Pro, and non‐chiral Sar by Marfey's analyses (Figure S116). Collectively, the structure of **11**, named actinomycin H_11_, was concluded to be a truncated actinomycin analog featuring the attachment of an N‐terminal 2‐methoxy‐2‐oxoacetyl group at the Thr residue.

Compound **12** provided the molecular formula C_25_H_39_N_5_O_9_ as determined by the HR‐ESIMS data (Figure S118). The NMR data of **12** indicated the presence of a pentapeptidolactone moiety (Tables 2 and 4; Figures S119–S125). The extended sequence of amino acid residues in the pentapeptidolactone ring of **12** consists of Val, Pro, Sar, MeVal, and Thr. HMBC cross‐peak from the Thr NH to C‐1 of the oxalyl group revealed the modification of the Thr residue by an oxalyl group. The residues were assigned as *D*‐Val, *L*‐MeVal, *L*‐Thr, *L*‐Pro, and non‐chiral Sar by Marfey's analyses (Figure S126). Collectively, the structure of **12**, named actinomycin H_12_, was concluded to be a truncated actinomycin analog featuring the attachment of an N‐terminal oxalyl group at the Thr residue.

Compound **13** afforded the molecular formula C_25_H_40_N_6_O_8_ as determined by the HR‐ESIMS data (Figure S128). The NMR data of **13** indicated the presence of a pentapeptidolactone moiety (Tables 2 and 4; Figures S129–S135). The extended sequence of amino acid residues in the pentapeptidolactone ring of **13** consists of Val, Pro, Sar, MeVal, and Thr. HMBC cross‐peaks between the Thr NH and C‐1 of the 2‐amino‐2‐oxoacetyl moiety and those between NH_2_‐2/C‐1 and NH_2_‐2/C‐2 indicated an additional 2‐amino‐2‐oxoacetyl group connected to the N‐terminus of the Thr residue. The residues were assigned as *D*‐Val, *L*‐MeVal, *L*‐Thr, *L*‐Pro, and non‐chiral Sar by Marfey's analyses (Figure S136). Collectively, the structure of **13**, named actinomycin H_13_, was concluded to be a truncated actinomycin analog featuring the presence of an N‐terminal 2‐amino‐2‐oxoacetyl group connected at the Thr residue. The same truncated scaffold of **11**–**13**, along with the different *N*‐terminal modification patterns, indicated the possible degradation or transformation during the process of fermentation or isolation.

Compound **14** had the molecular formula C_31_H_44_N_6_O_9_ as deduced by analysis of the HR‐ESIMS data (Figure S138). The NMR data of **14** indicated the presence of a monocyclic chromophore and a pentapeptidolactone moiety (Tables 2 and 4; Figures S139–S144). HMBC cross‐peak from a methine proton (*δ*_H_ 6.61 s) to C‐1, C‐5, and C‐7 revealed a methine at C‐3. HMBC correlations from two exchangeable amino protons (*δ*_H_ 8.56 s, 10.30 s) to C‐1 and C‐5 indicated the 6‐NH_2_ group. In addition, HMBC correlations from the methyl protons (*δ*_H_ 1.96 s) to C‐3, C‐4, and C‐5 corroborated the presence of the 7‐Me group (Figure 2). The C‐2 (*δ*_C_ 183.8 ppm) and C‐5 (*δ*_C_ 182.4 ppm) were consistent with two quinone carbonyl carbons. Thus, the chromophore of **14** was concluded to be a monocyclic 2‐amino‐6‐methylcyclohexa‐2,5‐diene‐1,4‐dione. The extended sequence of amino acid residues in the pentapeptidolactone ring of **14** consists of Val, Pro, Sar, MeVal, and Thr. The residues were assigned as *D*‐Val, *L*‐MeVal, *L*‐Thr, *L*‐Pro, and non‐chiral Sar by Marfey's analyses (Figure S145). Collectively, the above findings established the structure of **14**, named actinomycin H_14_.

Compound **15** afforded the molecular formula C_32_H_44_N_6_O_9_ as deduced by analysis of the HR‐ESIMS data (Figure S147). The NMR data of **15** indicated the presence of a bicyclic chromophore and a pentapeptidolactone moiety (Tables 2 and 4; Figures S148–S154). ^1^H–^1^H COSY correlations between H‐2/H‐3, along with HMBC correlations between H‐2/C‐4, H‐2/C‐6, H‐2/C‐11, H‐3/C‐1, H‐3/C‐5, H_3_‐10/C‐3, H_3_‐10/C‐4, and H_3_‐10/C‐5, indicated the presence of a monomethyl‐substituted benzene ring. HMBC cross‐peaks from an exchangeable proton (*δ*_H_ 11.70 s) to C‐5, C‐6, and C‐8 revealed the presence of the 9‐NH group (Figure 2). The downfield‐shifted C‐8 (*δ*_C_ 154.1 ppm) was identified as a carbonyl carbon. Collectively, the above findings corroborated the presence of an oxazolidin‐2‐one motif. Thus, the bicyclic chromophore of **15** was concluded to be 7‐methylbenzo[*d*]oxazol‐2(3*H*)‐one. The extended sequence of amino acid residues in the pentapeptidolactone ring of **15** consists of Val, Pro, Sar, MeVal, and Thr. The residues were assigned as *D*‐Val, *L*‐MeVal, *L*‐Thr, *L*‐Pro, and non‐chiral Sar by Marfey's analyses (Figure S155). Collectively, the above findings established the structure of **15**, named actinomycin H_15_.

Human MDA‐MB‐231 and murine 4T1 TNBC cancer cell lines were employed to assess the antiproliferative‌ effects of all the compounds. Compounds **1**–**6** and **9** exhibited antiproliferative activity against both cell lines in the CCK‐8 assay (Figure S156). In MDA‐MB‐231 cells, **1**–**6** showed more potent antiproliferative activity than that of the truncated analog **9**. Compound **4** was the most active (IC_50_ = 11.89 nM) (Figure 3A). Preliminary structure–activity relationship (SAR) investigations indicated that **1**–**6**, containing two pentapeptidolactone moieties, exhibited marked antiproliferative activities, whereas among the truncated analogs **7**–**15**, only **9** retained notable activity. Based on the above SAR analyses, **1** and **9** were selected for further mechanistic investigation, representing an actinomycin analog containing two pentapeptidolactone moieties and a truncated actinomycin analog, respectively. Although the inhibitory activity of **1** (IC_50_ = 28.00 vs. 11.89 nM) was weaker than that of **4**, it was also selected for further mechanistic investigation because **1** and **2** are positional isomers, only differing in the position of the *L*‐2‐MeAba residue. Cell cycle analyses revealed that the treatment of **1** and **9** induced marked S‐phase accumulation and decreased the G0/G1‐phase population in MDA‐MB‐231 cells (Figure 3B). This reduction in cell viability was accompanied by S‐phase arrest, suggesting that cell cycle disruption may contribute to the antiproliferative effects of **1** and **9**.

**FIGURE 3:**
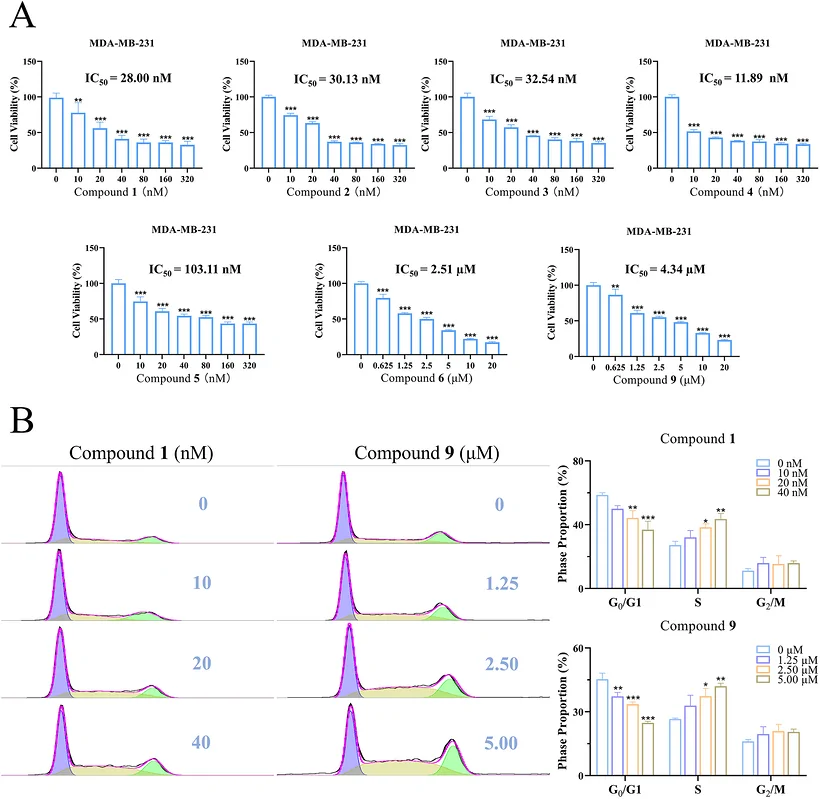
Antiproliferative effects in MDA‐MB‐231 cells. (A) Cell viability determined by the CCK‐8 assay. (B) Flow cytometric analysis of cell cycle arrest. Data are presented as mean ± SD (*n* = 3); *p < 0.05, **p < 0.01, ***p < 0.001 compared to the control group.

Apoptosis is a major mechanism underlying the antitumor activity of many anticancer agents [24]. To investigate the effects of **1** and **9** on apoptosis in MDA‐MB‐231 cells, flow cytometry was employed. The proportion of apoptotic MDA‐MB‐231 cells increased upon treatment with both compounds (Figure 4A). Mitochondrial depolarization is an early hallmark of apoptosis [25]. Accordingly, MDA‐MB‐231 cells were treated with **1** and **9** and subsequently stained with the JC‐1 probe. Enhanced green fluorescence indicated mitochondrial membrane potential dissipation and early apoptotic changes (Figure 4B). The above findings suggested that mitochondrial dysfunction might contribute to the apoptosis induced by **1** and **9**.

**FIGURE 4:**
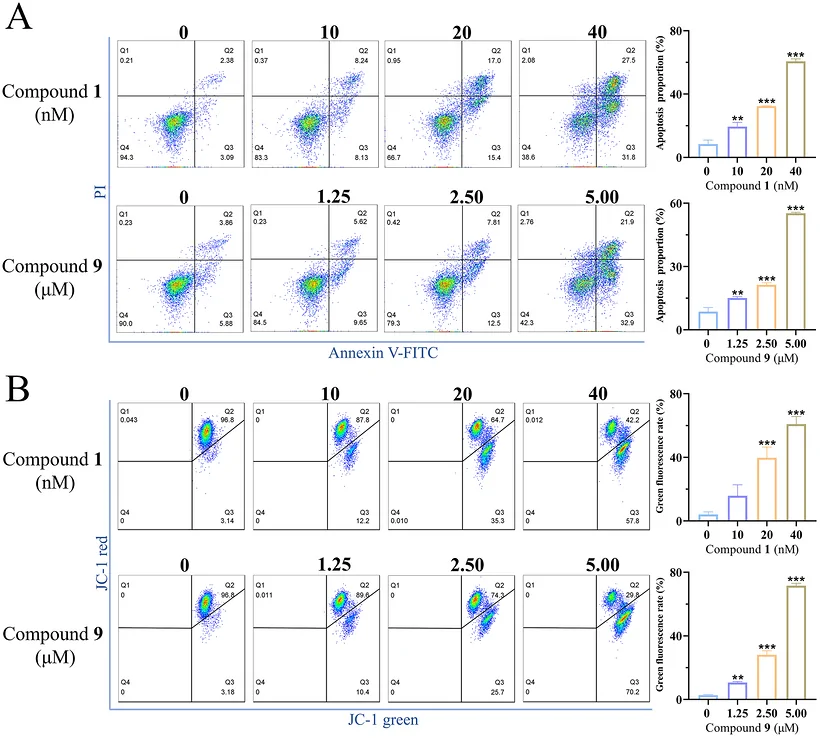
Apoptosis and mitochondrial membrane potential alterations in MDA‐MB‐231 cells. (A) Apoptosis was quantified by Annexin V‐FITC/PI staining. (B) Mitochondrial membrane potential dissipation was assessed using JC‐1 staining. Data are presented as mean ± SD (*n* = 3); *p < 0.05, **p < 0.01, ***p < 0.001 compared to the control group.

Ferroptosis is driven by iron‐dependent lipid peroxidation of membrane phospholipids [26, 27]. To determine whether **1** and **9** induce ferroptosis, intracellular lipid peroxidation, reactive oxygen species (ROS), and malondialdehyde were evaluated. BODIPY 581/591 C11 staining demonstrated that both compounds markedly increased lipid peroxidation (Figure 5A). In agreement with the above findings, DCFH‐DA staining revealed a concentration‐dependent elevation of ROS (Figure 5B). Furthermore, treatment with **1** and **9** significantly increased intracellular MDA levels (Figure 5C). Ferrostatin‐1 (Fer‐1), a ferroptosis inhibitor, significantly restored cell viability following the treatment with **1** and **9** (Figure 5D). The coordinated increases in ROS, lipid peroxidation, and MDA, together with the restoration of cell viability by Fer‐1, supported the involvement of ferroptosis in the cell death induced by **1** and **9**. Taken together, these findings suggested that **1** and **9** suppressed TNBC cell growth through S‐phase arrest and induced cell death through apoptosis and ferroptosis.

**FIGURE 5:**
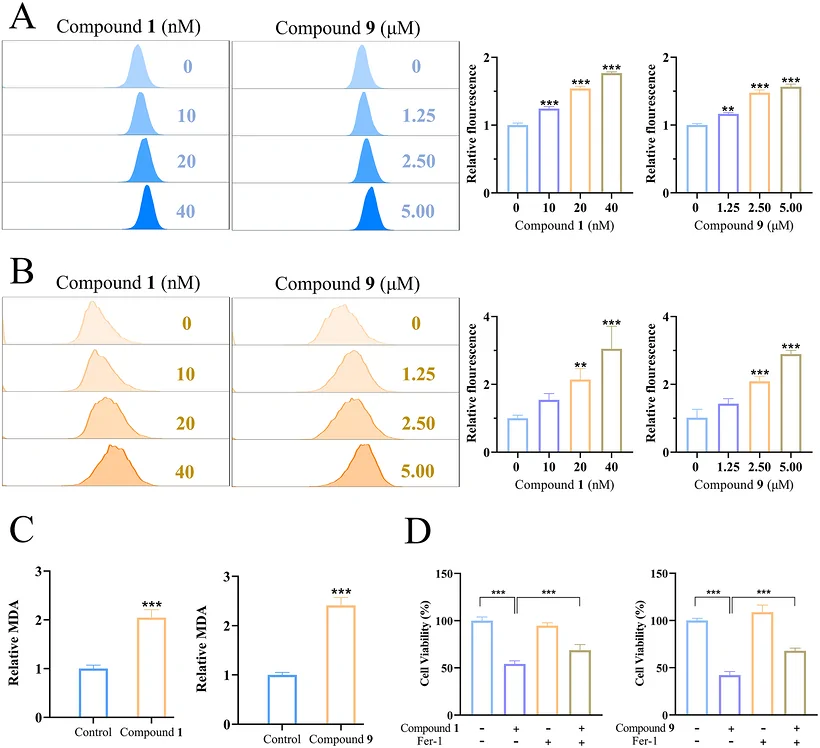
Ferroptosis‐related effects in MDA‐MB‐231 cells. (A) Lipid peroxidation assessed by BODIPY 581/591 C11 staining. (B) Intracellular ROS assessed by DCFH‐DA staining. (C) Malondialdehyde levels assessed by the TBARS assay. (D) Fer‐1‐mediated rescue assessed by the CCK‐8 assay. Data are presented as mean ± SD (*n* = 3); *p < 0.05, **p < 0.01, ***p < 0.001 compared with the control or corresponding compound‐treated group.

## 3 Conclusions

In summary, fifteen new actinomycin analogs, named actinomycins H_1_–H_15_, were identified from *S. polyantibioticus* S4D258. The structures of these compounds differ in the pentapeptidolactone moiety. Actinomycins H_7_–H_15_ are truncated actinomycin analogs. Actinomycins H_1_–H_6_ (**1–6**) and H_9_ (**9**) exhibited significant antiproliferative activities against both MDA‐MB‐231 and 4T1 TNBC cells. Mechanistic investigations revealed that actinomycins H_1_ (**1**) and H_9_ (**9**) induced S‐phase arrest, apoptosis, and mitochondrial membrane potential disruption. In addition, treatment with both compounds increased intracellular ROS, promoted lipid peroxidation, and elevated malondialdehyde levels, whereas ferrostatin‐1 reversed the cytotoxic effects, suggesting that ferroptosis contributes to anticancer effects of these compounds. Collectively, these compounds broaden the chemical diversity of natural actinomycins and support their further exploration as potential anti‐TNBC leads.

## 4 Experimental Section

### 4.1 General Experimental Procedures

UV spectra were recorded on a Shimadzu UV‐2600i double‐beam spectrophotometer (Kyoto, Japan). LC–MS measurements were carried out using a Vanquish Auto‐liquid chromatography system coupled with a TSQ Quantis Plus mass spectrometer (Thermo Fisher Scientific, Waltham, MA, USA). HR‐ESIMS spectra were obtained with a Bruker maXis Q‐TOF instrument (Karlsruhe, Germany). NMR spectra were recorded on a Bruker AVANCE 600 MHz spectrometer (Bruker, Karlsruhe, Germany). A Vanquish LC system (Thermo Scientific) was used for semi‐preparative and analytical HPLC with YMC‐Pack ODS‐A columns (5 µm; Kyoto, Japan; 250 × 10 mm i.d. and 250 × 4.6 mm i.d., respectively). Optical rotation values were recorded using a Model 341 polarimeter (PerkinElmer, Waltham, MA, USA).

### 4.2 Actinomycete Material

The marine actinomycete strain S4D258 was isolated from the South China Sea sediments by Prof. Gaiyun Zhang at the Third Institute of Oceanography, Ministry of Natural Resources, China. 16S rRNA gene sequence analysis and comparison with the NCBI database assigned strain S4D258 to *S. polyantibioticus*.

### 4.3 Extraction and Isolation

*S. polyantibioticus* S4D258 was cultivated in 100 mL of ISP2 seed medium for 5 days with shaking (28°C, 180 rpm). The seed cultures were dispensed into 100 Erlenmeyer flasks containing 250 mL of F2 production medium each (g/L: beef extract 1; soluble starch 10; yeast extract 10; corn steep liquor 3; glucose 20; CaCO_3_ 2; MgSO_4_·7H2O 0.5; and KH_2_PO4 0.5; sea salt 2.5% w/v) and cultured for 14 days. Upon completion of fermentation, cultures were pooled and filtered under vacuum. The supernatant was concentrated and extracted with EtOAc, and the extract was evaporated to dryness, yielding 13.5 g of crude extract. Compound yields (%) were calculated on a w/w basis relative to the crude extract.

The resulting extract was fractionated on a YMC‐GEL ODS‐A‐HG column with a MeOH/H_2_O gradient (30%–100% MeOH), yielding 58 fractions. Fraction 31, purified with MeCN/H_2_O (47:53), yielded compound **5** (4.0 mg, 0.0296% yield, *t*_R_ = 74.3 min). Pooled fractions 32–33 (MeCN/H_2_O, 50:50) afforded compounds **1** (5.2 mg, 0.0385% yield, *t*_R_ = 50.5 min) and **2** (5.0 mg, 0.0370% yield, *t*_R_ = 52.6 min). Pooled fractions 34–35 (MeCN/H_2_O, 55:45) gave compounds **4** (4.8 mg, 0.0356% yield, *t*_R_ = 35.6 min) and **3** (6.3 mg, 0.0467% yield, *t*_R_ = 40.1 min). Fraction 50, purified with MeCN/H_2_O containing 0.1% formic acid (52:48), provided compound **6** (12.0 mg, 0.0889% yield, *t*_R_ = 32.4 min).

Fractions 5–27 (4.5 g) were pooled for further separation on an ODS‐A column with a MeOH/H_2_O gradient (25%–70% MeOH), yielding 36 subfractions. Subfraction 1 was purified with MeCN/H_2_O (20:80), yielding compound **12** (16.3 mg, 0.1207% yield, *t*_R_ = 24.8 min). Subfraction 2, purified with MeCN/H_2_O (20:80), afforded compounds **13** (8.4 mg, 0.0622% yield, *t*_R_ = 46.2 min) and **11** (10.2 mg, 0.0756% yield, *t*_R_ = 60.0 min). Subfraction 4, purified with MeCN/H_2_O (30:70), gave compounds **7** (7.8 mg, 0.0578% yield, *t*_R_ = 36.2 min), **10** (11.6 mg, 0.0859% yield, *t*_R_ = 42.3 min), and **14** (2.1 mg, 0.0156% yield, *t*_R_ = 56.6 min). Subfraction 9, purified with MeCN/H_2_O (35:65), yielded compound **15** (2.0 mg, 0.0148% yield, *t*_R_ = 34.5 min). Pooled subfractions 10–11, purified with MeCN/H_2_O (35:65), afforded compound **8** (5.6 mg, 0.0415% yield, *t*_R_ = 24.1 min). Subfraction 18, purified with MeCN/H_2_O (65:35), provided compound **9** (13.6 mg, 0.1007% yield, *t*_R_ = 31.5 min).

**1**: Red amorphous powder; ${\left[a\right]}_{D}^{25}$ = −317.5 (*c* 0.1, MeOH); UV (MeOH) *λ*_max_ (log *ε*) 207 (4.78), 239 (4.52), 442 (4.36) nm (Figure S1); HR‐ESIMS *m/z* 1241.6220 [M + H]^+^ (calcd for C_61_H_85_N_12_O_16_, 1241.6201).

**2**: Red amorphous powder; ${\left[a\right]}_{D}^{25}$ = −310.0 (*c* 0.15, MeOH); UV (MeOH) *λ*_max_ (log *ε*) 208 (4.85), 239 (4.61), 442 (4.45) nm (Figure S14); HR‐ESIMS *m/z* 1241.6205 [M + H]^+^ (calcd for C_61_H_85_N_12_O_16_, 1241.6201).

**3**: Red amorphous powder; ${\left[a\right]}_{D}^{25}$ = −375.0 (*c* 0.1, MeOH); UV (MeOH) *λ*_max_ (log *ε*) 205 (4.47), 240 (4.53), 448 (4.33) nm (Figure S24); HR‐ESIMS *m/z* 1241.6203 [M + H]^+^ (calcd for C_61_H_85_N_12_O_16_, 1241.6201).

**4**: Red amorphous powder; ${\left[a\right]}_{D}^{25}$ = −388.8 (*c* 0.1, MeOH); UV (MeOH) *λ*_max_ (log *ε*) 207 (4.83), 238 (4.58), 442 (4.40) nm (Figure S35); HR‐ESIMS *m/z* 1241.6202 [M + H]^+^ (calcd for C_61_H_85_N_12_O_16_, 1241.6201).

**5**: Red amorphous powder; ${\left[a\right]}_{D}^{25}$ = −313.3 (c 0.1, MeOH); UV (MeOH) λmax (log ε) 206 (4.77), 239 (4.56), 444 (4.37) nm (Figure S46); HR‐ESIMS m/z 1257.6141 [M + H]^+^ (calcd for C_61_H_85_N_12_O_17_, 1257.6150).

**6**: Red amorphous powder; ${\left[a\right]}_{D}^{25}$ = −191.3 (*c* 0.2, MeOH); UV (MeOH) *λ*_max_ (log *ε*) 211 (4.72), 255 (4.56), 450 (4.25) nm (Figure S57); HR‐ESIMS *m/z* 1384.6774 [M + NH_4_]^+^ (calcd for C_67_H_94_N_13_O_19_, 1384.6783).

**7**: Yellow amorphous powder; ${\left[a\right]}_{D}^{25}$ = −110.0 (*c* 0.2, MeOH); UV (MeOH) *λ*_max_ (log *ε*) 212 (4.40), 316 (3.67) nm (Figure S67); HR‐ESIMS *m/z* 768.3566 [M + H]^+^ (calcd for C_37_H_50_N_7_O_11_, 768.3563).

**8**: Yellow amorphous powder; ${\left[a\right]}_{D}^{25}$ = −98.5 (*c* 0.5, MeOH); UV (MeOH) *λ*_max_ (log *ε*) 214 (4.41), 316 (3.76) nm (Figure S77); HR‐ESIMS *m/z* 741.3447 [M + H]^+^ (calcd for C_36_H_49_N_6_O_11_, 741.3454).

**9**: Yellow amorphous powder; ${\left[a\right]}_{D}^{25}$ = −76.3 (*c* 0.2, MeOH); UV (MeOH) *λ*_max_ (log *ε*) 214 (4.43), 316 (3.79) nm (Figure S87); HR‐ESIMS *m/z* 755.3610 [M + H]^+^ (calcd for C_37_H_51_N_6_O_11_, 755.3610).

**10**: Yellow amorphous powder; ${\left[a\right]}_{D}^{25}$ = −92.5 (*c* 0.1, MeOH); UV (MeOH) *λ*_max_ (log *ε*) 212 (4.39), 316 (3.65) nm (Figure S97); HR‐ESIMS *m/z* 740.3623 [M + H]^+^ (calcd for C_36_H_50_N_7_O_10_, 740.3614).

**11**: White amorphous powder; ${\left[a\right]}_{D}^{25}$ = −57.5 (c 0.2, MeOH); UV (MeOH) λmax (log ε) 218 (3.94) nm (Figure S107); HR‐ESIMS m/z 568.2979 [M + H]^+^ (calcd for C_26_H_42_N_5_O_9_, 568.2977).

**12**: White amorphous powder; ${\left[a\right]}_{D}^{25}$ = −34.5 (*c* 1.0, MeOH); UV (MeOH) *λ*_max_ (log *ε*) 216 (3.67) nm (Figure S117); HR‐ESIMS *m/z* 554.2829 [M + H]^+^ (calcd for C_25_H_40_N_5_O_9_, 554.2821).

**13**: White amorphous powder; ${\left[a\right]}_{D}^{25}$ = −68.8 (*c* 0.2, MeOH); UV (MeOH) *λ*_max_ (log *ε*) 219 (3.94) nm (Figure S127); HR‐ESIMS *m/z* 553.2979 [M + H]^+^ (calcd for C_25_H_41_N_6_O_8_, 553.2980).

**14**: Red amorphous powder; ${\left[a\right]}_{D}^{25}$ = −20.0 (*c* 0.2, MeOH); UV (MeOH) *λ*_max_ (log *ε*) 211 (4.64), 260 (4.33), 456 (3.40) nm (Figure S137); HR‐ESIMS *m/z* 645.3249 [M + H]^+^ (calcd for C_31_H_45_N_6_O_9_, 645.3243).

**15**: Yellow amorphous powder; ${\left[a\right]}_{D}^{25}$ = −102.5 (*c* 0.1, MeOH); UV (MeOH) *λ*_max_ (log *ε*) 218 (4.67), 249 (4.33), 298 (3.95) nm (Figure S146); HR‐ESIMS *m/z* 657.3255 [M + H]^+^ (calcd for C_32_H_45_N_6_O_9_, 657.3243).

### 4.4 Marfey's Analysis

The target compounds (0.5 mg each) were hydrolyzed in sealed vials under acidic conditions (6 M HCl, 110°C, 24 h). After hydrolysis, the samples were evaporated to dryness under a stream of N_2_, mixed with 1 M NaHCO_3_ solution (20 µL), and derivatized in the dark with 1% (w/v) Nα‐(2,4‐Dinitro‐5‐fluorophenyl)‐*L*‐alaninamide (FDAA, Marfey's reagent, Shanghai Macklin Biochemical Co., Ltd.) in acetone (50 µL) at 45°C for 60 min. The resulting derivatives were tested by LC–MS using a 20%–60% MeCN gradient in 0.1% formic acid (60 min; 1.0 mL/min). The absolute configurations were assigned by comparing the FDAA derivatives with authentic standards under the same conditions. The standards included the *L‐* and *D‐*forms of Val, Thr, Pro, Hyp, MeVal, Ser, 2‐Aba, 2‐MeAba, allo‐Thr, and cis‐Hyp, together with Sar (Shanghai Macklin Biochemical Co., Ltd.), all derivatized under the same conditions.

### 4.5 Cell Culture

MDA‐MB‐231 (RRID: CVCL_0062) and 4T1 cells (RRID: CVCL_0125) were obtained from the Cell Bank of the Chinese Academy of Sciences (Shanghai, China). Both cell lines were cultured in DMEM supplemented with 10% fetal bovine serum (Thermo Fisher Scientific) and 1% penicillin‐streptomycin (Solarbio Science & Technology Co., Ltd., Beijing, China), and maintained at 37°C in a humidified incubator with 5% CO_2_.

### 4.6 Cell Viability Assay

MDA‐MB‐231 and 4T1 cells were seeded at 4.0 × 10^3^ cells/well and cultured for 24 h before treatment with compounds **1**–**15** (0–20 µM) for 48 h under standard culture conditions. For the Fer‐1 rescue experiment, MDA‐MB‐231 cells with 1 h pretreatment of Fer‐1 (1.0 µM) were subsequently treated with compound **1** (20 nM) or compound **9** (2.50 µM) for 48 h. After adding 10 µL of CCK‐8 solution (Beyotime Institute of Biotechnology, Shanghai, China) and incubating for 2 h, the OD450 was measured with a Thermo Fisher Scientific Multiskan SkyHigh microplate reader.

### 4.7 Cell Cycle Analysis

Following treatment, cells were subjected to overnight fixation in 70% ethanol at 4°C. After removing ethanol, the samples were resuspended in 500 µL of PI/RNase staining solution (Beyotime) and maintained under light‐protected conditions for 30 min. Flow cytometric data were acquired on a FACSCalibur instrument (BD Biosciences, CA, USA).

### 4.8 Apoptosis Analysis

Following treatment, the adherent and nonadherent cell fractions were collected and stained with the Annexin V‐FITC/PI kit (Beyotime) for 30 min at room temperature in the dark. Flow‐cytometric acquisition was performed immediately on a FACSCalibur system, followed by quantification of apoptotic fractions in FlowJo software.

### 4.9 Measurement of Mitochondrial Membrane Potential

Following treatment, the adherent and nonadherent cells were combined and pelleted. The cells were then stained with a JC‐1 kit (Beyotime) for 20 min at 37°C, washed twice with 1× staining buffer, and resuspended in fresh buffer. Flow‐cytometric data were acquired immediately on a FACSCalibur system.

### 4.10 Measurement of Lipid Peroxidation

Following two washes with PBS, BODIPY 581/591 C11 (2 µM, Beyotime) was added to the cells, and the mixture was incubated for 30 min in the dark. After incubation, the cells were resuspended in fresh PBS and immediately analyzed by flow cytometry on a FACSCalibur instrument. Lipid peroxidation was evaluated according to the increase in green fluorescence intensity.

### 4.11 Measurement of Intracellular ROS Content

Following treatment, the adherent and nonadherent cell fractions were pooled and exposed to 10 µM DCFH‐DA (Beyotime) in serum‐free DMEM for 20 min under light‐protected conditions. Following three rinses in serum‐free DMEM, the samples were brought into suspension with 500 µL of fresh medium and immediately subjected to flow cytometric analysis on a FACSCalibur instrument. Intracellular ROS levels were evaluated according to the fluorescence intensity of DCF.

### 4.12 Measurement of Intracellular Malondialdehyde Levels

Malondialdehyde accumulation was assessed following exposure to compounds **1** (20 nM) or **9** (2.50 µM). The harvested cells were adjusted to 2 × 10^6^ cells per sample and resuspended in extraction buffer. After sonication on ice, the lysates were centrifuged (8,000 × g, 10 min). The supernatants were used for TBARS analysis following the manufacturer's protocol (Solarbio). The reaction mixtures were heated (100°C, 1 h) and then centrifuged again (10,000 × g, 10 min) under ambient conditions. The resulting supernatants were analyzed at 532 nm using a Multiskan SkyHigh microplate reader, with readings at 600 nm for background correction.

### 4.13 Statistical Analysis

Data are presented as the mean ± standard deviation (SD) from at least three independent experiments. Two‑group comparisons were assessed by unpaired *t*‑tests, and multiple‑group differences by one‑way ANOVA with Tukey's test, using SPSS 25.0 and GraphPad Prism 8.0. Differences were regarded as significant when *p* < 0.05.

## Author Contributions

The manuscript was written through contributions of all authors. All authors have given approval to the final version of the manuscript. **He Pang**: investigation, visualization, writing – original draft, writing – review and editing. **Xiao‐Min Huang**: investigation, visualization. **Usama Ramadan Abdelmohsen**: methodology, visualization, writing – review and editing. **Li Shen**: conceptualization, methodology, investigation, visualization, supervision, writing – original draft, writing – review and editing, funding acquisition, resources. **Jun Wu**: conceptualization, methodology, investigation, visualization, supervision, writing – original draft, writing – review and editing, funding acquisition, resources.

## Conflicts of Interest

The authors declare no conflicts of interest.

## Supporting information



UV, HR‐ESIMS, 1D and 2D NMR, and Marfey's analysis spectra for compounds **1–15**. **Supporting File**: cbdv71791‐sup‐0001‐SuppMat.pdf.

## Data Availability

The data that support the findings of this study are available in the Supporting Information of this article.

## References

[1] A. C. Garrido‐Castro , N. U. Lin , and K. Polyak , “Insights into Molecular Classifications of Triple‐Negative Breast Cancer: Improving Patient Selection for Treatment,” Cancer Discovery 9 (2019): 176–198, 10.1158/2159-8290.CD-18-1177. PMC6387871 30679171

[2] H. Kennecke , R. Yerushalmi , R. Woods , et al., “Metastatic Behavior of Breast Cancer Subtypes,” Journal of Clinical Oncology 28 (2010): 3271–3277, 10.1200/JCO.2009.25.9820. 20498394

[3] J. Cortes , H. S. Rugo , D. W. Cescon , et al., “Pembrolizumab plus Chemotherapy in Advanced Triple‐Negative Breast Cancer,” New England Journal of Medicine 387 (2022): 217–226, 10.1056/NEJMoa2202809. 35857659

[4] P. Zagami and L. A. Carey , “Triple Negative Breast Cancer: Pitfalls and Progress,” npj Breast Cancer 8 (2022): 95, 10.1038/s41523-022-00468-0. PMC9392735 35987766

[5] G. Bianchini , C. De Angelis , L. Licata , and L. Gianni , “Treatment Landscape of Triple‐Negative Breast Cancer—Expanded Options, Evolving Needs,” Nature Reviews Clinical Oncology 19 (2022): 91–113, 10.1038/s41571-021-00565-2. 34754128

[6] S. A. Hassan and M. N. Abdullah , “Synthesis of New 4‐Thiazolidinone Bearing Thiazole, Assess Anticancer, and Antimicrobial Activities: Insights from DFT, and Molecular Docking,” Journal of Molecular Structure 1346 (2025): 143181, 10.1016/j.molstruc.2025.143181.

[7] K. D. Issa , D. A. Abdul , S. O. Mamand , N. O. Ahmad , A. M. Salih , and S. A. Hassan , “Exploring the Anticancer, Antibacterial Potency, and Molecular Docking of Synthesized Azo‐Ether and Azo‐Sulfone Compounds,” ChemistrySelect 10 (2025): e02350, 10.1002/slct.202502350.

[8] S. A. Hassan , J. B. Ziwar , D. M. Aziz , and M. N. Abdullah , “Sonochemical Synthesis of New Thiazolidin‐4‐one Derivatives as Potent Anticancer and Antimicrobial Agents with Docking Design, and Energy Gap Estimation,” Journal of Molecular Structure 1301 (2024): 137282, 10.1016/j.molstruc.2023.137282.

[9] H. Lackner , I. Bahner , N. Shigematsu , L. K. Pannell , and A. B. Mauger , “Structures of Five Components of the Actinomycin Z Complex from *Streptomyces fradiae*, Two of Which Contain 4‐Chlorothreonine,” Journal of Natural Products 63 (2000): 352–356, 10.1021/np990416u. 10757717

[10] W. Cai , X. Wang , S. I. Elshahawi , et al., “Antibacterial and Cytotoxic Actinomycins Y6–Y9 and Zp from *Streptomyces* sp. Strain Gö‐GS12,” Journal of Natural Products 79 (2016): 2731–2739, 10.1021/acs.jnatprod.6b00742. PMC5217177 27736087

[11] R. Satange , C.‐C. Chang , L.‐Y. Li , S.‐H. Lin , S. Neidle , and M.‐H. Hou , “Synergistic Binding of Actinomycin D and Echinomycin to DNA Mismatch Sites and Their Combined Anti‐Tumour Effects,” Nucleic Acids Research 51 (2023): 3540–3555, 10.1093/nar/gkad156. PMC10164580 36919604

[12] R. J. Osborne , V. Filiaci , J. C. Schink , et al., “Phase III Trial of Weekly Methotrexate or Pulsed Dactinomycin for Low‐Risk Gestational Trophoblastic Neoplasia: A Gynecologic Oncology Group Study,” Journal of Clinical Oncology 29 (2011): 825–831, 10.1200/JCO.2010.30.4386. PMC3068058 21263100

[13] C. A. S. Arndt , J. A. Stoner , D. S. Hawkins , et al., “Vincristine, Actinomycin, and Cyclophosphamide Compared with Vincristine, Actinomycin, and Cyclophosphamide Alternating with Vincristine, Topotecan, and Cyclophosphamide for Intermediate‐Risk Rhabdomyosarcoma: Children's Oncology Group Study D9803,” Journal of Clinical Oncology 27 (2009): 5182–5188, 10.1200/JCO.2009.22.3768. PMC2773476 19770373

[14] J. S. Dome , E. A. Mullen , D. B. Dix , et al., “Impact of the First Generation of Children's Oncology Group Clinical Trials on Clinical Practice for Wilms Tumor,” Journal of the National Comprehensive Cancer Network 19 (2021): 978–985, 10.6004/jnccn.2021.7070. PMC8805512 34416705

[15] B. Brennan , L. Kirton , P. Marec‐Bérard , et al., “Comparison of Two Chemotherapy Regimens in Patients with Newly Diagnosed Ewing Sarcoma (EE2012): An Open‐Label, Randomised, Phase 3 Trial,” The Lancet 400 (2022): 1513–1521, 10.1016/S0140-6736(22)01790-1. 36522207

[16] M. C. Winter , “Treatment of Low‐Risk Gestational Trophoblastic Neoplasia,” Best Practice & Research Clinical Obstetrics & Gynaecology 74 (2021): 67–80, 10.1016/j.bpobgyn.2021.01.006. 33741258

[17] G. J. Veal , M. Cole , J. Errington , et al., “Pharmacokinetics of Dactinomycin in a Pediatric Patient Population: A United Kingdom Children's Cancer Study Group Study,” Clinical Cancer Research 11 (2005): 5893–5899, 10.1158/1078-0432.CCR-04-2546. 16115931

[18] A. L. Harvey , R. Edrada‐Ebel , and R. J. Quinn , “The Re‐Emergence of Natural Products for Drug Discovery in the Genomics Era,” Nature Reviews Drug Discovery 14 (2015): 111–129, 10.1038/nrd4510. 25614221

[19] J. Bitzer , M. Streibel , H. J. Langer , and S. Grond , “First Y‐Type Actinomycins from *Streptomyces* with Divergent Structure–Activity Relationships for Antibacterial and Cytotoxic Properties,” Organic & Biomolecular Chemistry 7 (2009): 444–450, 10.1039/b815689a. 19156308

[20] N. V. Machushynets , S. S. Elsayed , C. Du , et al., “Discovery of Actinomycin L, a New Member of the Actinomycin Family of Antibiotics,” Scientific Reports 12 (2022): 2813, 10.1038/s41598-022-06736-0. PMC8857259 35181725

[21] T. Liu , T. Xu , C. Hu , D. Sun , and G. Zhou , “Hemiactinomycin, an Undescribed Intermediate of Actinomycin Biosynthesis from an Actinomycetes Strain *Streptomyces antibioticus* H41‐55,” Natural Product Research 38 (2024): 1577–1582, 10.1080/14786419.2022.2161541. 36564053

[22] J. Bitzer , V. Gesheva , and A. Zeeck , “Actinomycins with Altered Threonine Units in the β‐Peptidolactone,” Journal of Natural Products 69 (2006): 1153–1157, 10.1021/np060063g. 16933866

[23] W.‐H. Jiao , W. Yuan , Z.‐Y. Li , et al., “Anti‐MRSA Actinomycins D1‐D4 from the Marine Sponge‐Associated Streptomyces sp. LHW52447,” Tetrahedron 74 (2018): 5914–5919, 10.1016/j.tet.2018.08.023.

[24] W. Liu , W. Jin , S. Zhu , Y. Chen , and B. Liu , “Targeting Regulated Cell Death (RCD) with Small‐Molecule Compounds in Cancer Therapy: A Revisited Review of Apoptosis, Autophagy‐Dependent Cell Death and Necroptosis,” Drug Discovery Today 27 (2022): 612–625, 10.1016/j.drudis.2021.10.011. 34718209

[25] A. Perelman , C. Wachtel , M. Cohen , S. Haupt , H. Shapiro , and A. Tzur , “JC‐1: Alternative Excitation Wavelengths Facilitate Mitochondrial Membrane Potential Cytometry,” Cell Death & Disease 3 (2012): e430, 10.1038/cddis.2012.171. PMC3542606 23171850

[26] Y. Yu , Y. Yan , F. Niu , et al., “Ferroptosis: A Cell Death Connecting Oxidative Stress, Inflammation and Cardiovascular Diseases,” Cell Death Discovery 7 (2021): 193, 10.1038/s41420-021-00579-w. PMC8313570 34312370

[27] S. J. Dixon , K. M. Lemberg , M. R. Lamprecht , et al., “Ferroptosis: An Iron‐Dependent Form of Nonapoptotic Cell Death,” Cell 149 (2012): 1060–1072, 10.1016/j.cell.2012.03.042. PMC3367386 22632970

