# Supplementary material for: Actinomycin Analogs From Streptomyces polyantibioticus S4D258: Structural Elucidation and Antiproliferative Activity Against Triple‐Negative Breast Cancer
Source: Chem Biodivers. 2026 Sep 30;23(10):e71791. doi: 10.1002/cbdv.71791 (PMC13626259; doi:10.1002/cbdv.71791)
Supplement: Supplementary file 1 [file CBDV-23-e71791-s001.pdf]

Supporting Information

Actinomycin Analogs from *Streptomyces polyantibioticus* S4D258: Structural Elucidation and Antiproliferative Activity against Triple-Negative Breast Cancer

He Pang,[a] Xiao-Min Huang,[a] Usama Ramadan Abdelmohsen,[c,*] Li Shen,[a,b,*] and Jun Wu,[a,b,*]

[a] Guangdong Provincial Engineering Technology Research Center for Innovative Drugs and Bioproducts, School of Pharmacy, Guangdong Medical University, Dongguan 523808, PR China.

[b] Dongguan Key Laboratory for Marine Innovative Drugs and Bioproducts, Guangdong Medical University, Dongguan 523808, PR China.

[c] Department of Pharmacognosy, Faculty of Pharmacy, Minia University, Minia 61519, Egypt.

*Corresponding authors: usama.ramadan@mu.edu.eg (U.R.A); shenli6052@sina.com (L.S.); wwujun68@163.com (J.W.)

Table of contents:

Figure S1. UV spectrum of compound **1** ········ S9
Figure S2. HR-ESIMS for compound **1** ········ S10
Figure S3. ^{1}H NMR (600 MHz) spectrum of compound **1** in $CDCl_3$ ········ S11–S13
Figure S4. ^{13}C NMR (150 MHz) spectrum of compound **1** in $CDCl_3$ ········ S14–S16
Figure S5. DEPT135 (150 MHz) spectrum of compound **1** in $CDCl_3$ ········ S17–S19
Figure S6. ^{1}H–^{1}H COSY (600 MHz) spectrum of compound **1** in $CDCl_3$ ········ S20–S22
Figure S7. HSQC (600 MHz) spectrum of compound **1** in $CDCl_3$ ········ S23–S25
Figure S8. HMBC (600 MHz) spectrum of compound **1** in $CDCl_3$ ········ S26–S30
Figure S9. NOESY (600 MHz) spectrum of compound **1** in $CDCl_3$ ········ S31–S33
Figure S10. Marfey's analysis of Sar, Val, and Pro standards ········ S34
Figure S11. Marfey's analysis of MeVal, and Thr standards ········ S35
Figure S12. Marfey's analysis of MeAba standard ········ S36
Figure S13. Marfey's analysis of compound **1** and standard amino acids ········ S37
Figure S14. UV spectrum of compound **2** ········ S38
Figure S15. HR-ESIMS for compound **2** ········ S39
Figure S16. ^{1}H NMR (600 MHz) spectrum of compound **2** in $CDCl_3$ ········ S40–S42
Figure S17. ^{13}C NMR (150 MHz) spectrum of compound **2** in $CDCl_3$ ········ S43–S45
Figure S18. DEPT135 (150 MHz) spectrum of compound **2** in $CDCl_3$ ········ S46–S48
Figure S19. ^{1}H–^{1}H COSY (600 MHz) spectrum of compound **2** in $CDCl_3$ ········ S49–S51
Figure S20. HSQC (600 MHz) spectrum of compound **2** in $CDCl_3$ ········ S52–S54
Figure S21. HMBC (600 MHz) spectrum of compound **2** in $CDCl_3$ ········ S55–S59

Figure S22. NOESY (600 MHz) spectrum of compound **2** in $CDCl_3$ ········ S60–S62
Figure S23. Marfey's analysis of compound **2** and standard amino acids ········ S63
Figure S24. UV spectrum of compound **3** ········ S64
Figure S25. HR-ESIMS for compound **3** ········ S65
Figure S26. ^{1}H NMR (600 MHz) spectrum of compound **3** in $CDCl_3$ ········ S66–S68
Figure S27. ^{13}C NMR (150 MHz) spectrum of compound **3** in $CDCl_3$ ········ S69–S71
Figure S28. DEPT135 (150 MHz) spectrum of compound **3** in $CDCl_3$ ········ S72–S74
Figure S29. ^{1}H–^{1}H COSY (600 MHz) spectrum of compound **3** in $CDCl_3$ ········ S75–S77
Figure S30. HSQC (600 MHz) spectrum of compound **3** in $CDCl_3$ ········ S78–S80
Figure S31. HMBC (600 MHz) spectrum of compound **3** in $CDCl_3$ ········ S81–S85
Figure S32. NOESY (600 MHz) spectrum of compound **3** in $CDCl_3$ ········ S86–S88
Figure S33. Marfey's analysis of Ser standard ········ S89
Figure S34. Marfey's analysis of compound **3** and standard amino acids ········ S90
Figure S35. UV spectrum of compound **4** ········ S91
Figure S36. HR-ESIMS for compound **4** ········ S92
Figure S37. ^{1}H NMR (600 MHz) spectrum of compound **4** in $CDCl_3$ ········ S93–S95
Figure S38. ^{13}C NMR (150 MHz) spectrum of compound **4** in $CDCl_3$ ········ S96–S98
Figure S39. DEPT135 (150 MHz) spectrum of compound **4** in $CDCl_3$ ········ S99–S101
Figure S40. ^{1}H–^{1}H COSY (600 MHz) spectrum of compound **4** in $CDCl_3$ ········ S102–S104
Figure S41. HSQC (600 MHz) spectrum of compound **4** in $CDCl_3$ ········ S105–S107
Figure S42. HMBC (600 MHz) spectrum of compound **4** in $CDCl_3$ ········ S108–S112
Figure S43. NOESY (600 MHz) spectrum of compound **4** in $CDCl_3$ ········ S113–S115
Figure S44. Marfey's analysis of 2-Aba standard ········ S116

Figure S45. Marfey's analysis of compound **4** and standard amino acids ··· S117
Figure S46. UV spectrum of compound **5** ··· S118
Figure S47. HR-ESIMS for compound **5** ··· S119
Figure S48. ^{1}H NMR (600 MHz) spectrum of compound **5** in $CDCl_3$ ··· S120–S122
Figure S49. ^{13}C NMR (150 MHz) spectrum of compound **5** in $CDCl_3$ ··· S123–S125
Figure S50. DEPT135 (150 MHz) spectrum of compound **5** in $CDCl_3$ ··· S126–S128
Figure S51. ^{1}H–^{1}H COSY (600 MHz) spectrum of compound **5** in $CDCl_3$ ··· S129–S131
Figure S52. HSQC (600 MHz) spectrum of compound **5** in $CDCl_3$ ··· S132–S134
Figure S53. HMBC (600 MHz) spectrum of compound **5** in $CDCl_3$ ··· S135–S139
Figure S54. NOESY (600 MHz) spectrum of compound **5** in $CDCl_3$ ··· S140–S142
Figure S55. Marfey's analysis of Hyp standard ··· S143
Figure S56. Marfey's analysis of compound **5** and standard amino acids ··· S144
Figure S57. UV spectrum of compound **6** ··· S145
Figure S58. HR-ESIMS for compound **6** ··· S146
Figure S59. ^{1}H NMR (600 MHz) spectrum of compound **6** in DMSO-d_6 ··· S147–S149
Figure S60. ^{13}C NMR (150 MHz) spectrum of compound **6** in DMSO-d_6 ··· S150–S152
Figure S61. DEPT135 (150 MHz) spectrum of compound **6** in DMSO-d_6 ··· S153–S155
Figure S62. ^{1}H–^{1}H COSY (600 MHz) spectrum of compound **6** in DMSO-d_6 ··· S156–S158
Figure S63. HSQC (600 MHz) spectrum of compound **6** in DMSO-d_6 ··· S159–S161
Figure S64. HMBC (600 MHz) spectrum of compound **6** in DMSO-d_6 ··· S162–S166
Figure S65. ROESY (600 MHz) spectrum of compound **6** in DMSO-d_6 ··· S167–S169
Figure S66. Marfey's analysis of compound **6** and standard amino acids ··· S170
Figure S67. UV spectrum of compound **7** ··· S171

Figure S68. HR-ESIMS for compound **7** ······ S172
Figure S69. ^{1}H NMR (600 MHz) spectrum of compound **7** in DMSO-d_6 ······ S173–S175
Figure S70. ^{13}C NMR (150 MHz) spectrum of compound **7** in DMSO-d_6 ······ S176–S178
Figure S71. DEPT135 (150 MHz) spectrum of compound **7** in DMSO-d_6 ······ S179–S181
Figure S72. ^{1}H–^{1}H COSY (600 MHz) spectrum of compound **7** in DMSO-d_6 ······ S182–S184
Figure S73. HSQC (600 MHz) spectrum of compound **7** in DMSO-d_6 ······ S185–S187
Figure S74. HMBC (600 MHz) spectrum of compound **7** in DMSO-d_6 ······ S188–S192
Figure S75. ROESY (600 MHz) spectrum of compound **7** in DMSO-d_6 ······ S193–S195
Figure S76. Marfey's analysis of compound **7** and standard amino acids ······ S196
Figure S77. UV spectrum of compound **8** ······ S197
Figure S78. HR-ESIMS for compound **8** ······ S198
Figure S79. ^{1}H NMR (600 MHz) spectrum of compound **8** in DMSO-d_6 ······ S199–S201
Figure S80. ^{13}C NMR (150 MHz) spectrum of compound **8** in DMSO-d_6 ······ S202–S204
Figure S81. DEPT135 (150 MHz) spectrum of compound **8** in DMSO-d_6 ······ S205–S207
Figure S82. ^{1}H–^{1}H COSY (600 MHz) spectrum of compound **8** in DMSO-d_6 ······ S208–S210
Figure S83. HSQC (600 MHz) spectrum of compound **8** in DMSO-d_6 ······ S211–S213
Figure S84. HMBC (600 MHz) spectrum of compound **8** in DMSO-d_6 ······ S214–S218
Figure S85. ROESY (600 MHz) spectrum of compound **8** in DMSO-d_6 ······ S219–S221
Figure S86. Marfey's analysis of compound **8** and standard amino acids ······ S222
Figure S87. UV spectrum of compound **9** ······ S223
Figure S88. HR-ESIMS for compound **9** ······ S224
Figure S89. ^{1}H NMR (600 MHz) spectrum of compound **9** in DMSO-d_6 ······ S225–S227
Figure S90. ^{13}C NMR (150 MHz) spectrum of compound **9** in DMSO-d_6 ······ S228–S230

Figure S91. DEPT135 (150 MHz) spectrum of compound **9** in DMSO-d_6 .... S231–S232
Figure S92. ^{1}H–^{1}H COSY (600 MHz) spectrum of compound **9** in DMSO-d_6 .... S233–S235
Figure S93. HSQC (600 MHz) spectrum of compound **9** in DMSO-d_6 .... S237–S239
Figure S94. HMBC (600 MHz) spectrum of compound **9** in DMSO-d_6 .... S240–S244
Figure S95. ROESY (600 MHz) spectrum of compound **9** in DMSO-d_6 .... S245–S247
Figure S96. Marfey's analysis of compound **9** and standard amino acids S248
Figure S97. UV spectrum of compound **10** S249
Figure S98. HR-ESIMS for compound **10** S250
Figure S99. ^{1}H NMR (600 MHz) spectrum of compound **10** in DMSO-d_6 .... S251–S253
Figure S100. ^{13}C NMR (150 MHz) spectrum of compound **10** in DMSO-d_6 .... S254–S256
Figure S101. DEPT135 (150 MHz) spectrum of compound **10** in DMSO-d_6 .... S257–S259
Figure S102. ^{1}H–^{1}H COSY (600 MHz) spectrum of compound **10** in DMSO-d_6 .... S260–S262
Figure S103. HSQC (600 MHz) spectrum of compound **10** in DMSO-d_6 .... S263–S265
Figure S104. HMBC (600 MHz) spectrum of compound **10** in DMSO-d_6 .... S266–S270
Figure S105. ROESY (600 MHz) spectrum of compound **10** in DMSO-d_6 .... S271–S273
Figure S106. Marfey's analysis of compound **10** and standard amino acids S274
Figure S107. UV spectrum of compound **11** S275
Figure S108. HR-ESIMS for compound **11** S276
Figure S109. ^{1}H NMR (600 MHz) spectrum of compound **11** in DMSO-d_6 .... S277–S279
Figure S110. ^{13}C NMR (150 MHz) spectrum of compound **11** in DMSO-d_6 .... S280–S282
Figure S111. DEPT135 (150 MHz) spectrum of compound **11** in DMSO-d_6 .... S283–S285
Figure S112. ^{1}H–^{1}H COSY (600 MHz) spectrum of compound **11** in DMSO-d_6 .... S286–S288
Figure S113. HSQC (600 MHz) spectrum of compound **11** in DMSO-d_6 .... S289–S291

Figure S114. HMBC (600 MHz) spectrum of compound **11** in DMSO-d_6 ·········· S292–S296
Figure S115. ROESY (600 MHz) spectrum of compound **11** in DMSO-d_6 ·········· S297–S299
Figure S116. Marfey's analysis of compound **11** and standard amino acids ·········· S300
Figure S117. UV spectrum of compound **12** ·········· S301
Figure S118. HR-ESIMS for compound **12** ·········· S302
Figure S119. ^{1}H NMR (600 MHz) spectrum of compound **12** in DMSO-d_6 ·········· S303–S305
Figure S120. ^{13}C NMR (150 MHz) spectrum of compound **12** in DMSO-d_6 ·········· S306–S308
Figure S121. DEPT135 (150 MHz) spectrum of compound **12** in DMSO-d_6 ·········· S309–S311
Figure S122. ^{1}H–^{1}H COSY (600 MHz) spectrum of compound **12** in DMSO-d_6 ·········· S312–S314
Figure S123. HSQC (600 MHz) spectrum of compound **12** in DMSO-d_6 ·········· S315–S317
Figure S124. HMBC (600 MHz) spectrum of compound **12** in DMSO-d_6 ·········· S318–S322
Figure S125. ROESY (600 MHz) spectrum of compound **12** in DMSO-d_6 ·········· S323–S325
Figure S126. Marfey's analysis of compound **12** and standard amino acids ·········· S326
Figure S127. UV spectrum of compound **13** ·········· S327
Figure S128. HR-ESIMS for compound **13** ·········· S328
Figure S129. ^{1}H NMR (600 MHz) spectrum of compound **13** in DMSO-d_6 ·········· S329–S331
Figure S130. ^{13}C NMR (150 MHz) spectrum of compound **13** in DMSO-d_6 ·········· S332–S334
Figure S131. DEPT135 (150 MHz) spectrum of compound **13** in DMSO-d_6 ·········· S335–S337
Figure S132. ^{1}H–^{1}H COSY (600 MHz) spectrum of compound **13** in DMSO-d_6 ·········· S338–S340
Figure S133. HSQC (600 MHz) spectrum of compound **13** in DMSO-d_6 ·········· S341–S343
Figure S134. HMBC (600 MHz) spectrum of compound **13** in DMSO-d_6 ·········· S344–S348
Figure S135. ROESY (600 MHz) spectrum of compound **13** in DMSO-d_6 ·········· S349–S351
Figure S136. Marfey's analysis of compound **13** and standard amino acids ·········· S352

Figure S137. UV spectrum of compound **14** ······ S353
Figure S138. HR-ESIMS for compound **14** ······ S354
Figure S139. ^{1}H NMR (600 MHz) spectrum of compound **14** in DMSO-d_6 ······ S355–S357
Figure S140. ^{13}C NMR (150 MHz) spectrum of compound **14** in DMSO-d_6 ······ S358–S360
Figure S141. DEPT135 (150 MHz) spectrum of compound **14** in DMSO-d_6 ······ S361–S363
Figure S142. ^{1}H–^{1}H COSY (600 MHz) spectrum of compound **14** in DMSO-d_6 ······ S364–S366
Figure S143. HSQC (600 MHz) spectrum of compound **14** in DMSO-d_6 ······ S367–S369
Figure S144. HMBC (600 MHz) spectrum of compound **14** in DMSO-d_6 ······ S370–S374
Figure S145. Marfey's analysis of compound **14** and standard amino acids ······ S375
Figure S146. UV spectrum of compound **15** ······ S376
Figure S147. HR-ESIMS for compound **15** ······ S377
Figure S148. ^{1}H NMR (600 MHz) spectrum of compound **15** in DMSO-d_6 ······ S378–S380
Figure S149. ^{13}C NMR (150 MHz) spectrum of compound **15** in DMSO-d_6 ······ S381–S383
Figure S150. DEPT135 (150 MHz) spectrum of compound **15** in DMSO-d_6 ······ S384–S386
Figure S151. ^{1}H–^{1}H COSY (600 MHz) spectrum of compound **15** in DMSO-d_6 ······ S387–S389
Figure S152. HSQC (600 MHz) spectrum of compound **15** in DMSO-d_6 ······ S390–S392
Figure S153. HMBC (600 MHz) spectrum of compound **15** in DMSO-d_6 ······ S393–S396
Figure S154. ROESY (600 MHz) spectrum of compound **15** in DMSO-d_6 ······ S397–S399
Figure S155. Marfey's analysis of compound **15** and standard amino acids ······ S400
Figure S156. IC_{50} values of compounds **1–15** in MDA-MB-231 and 4T1 TNBC Cells ······ S401

Figure S1. UV spectrum of compound 1

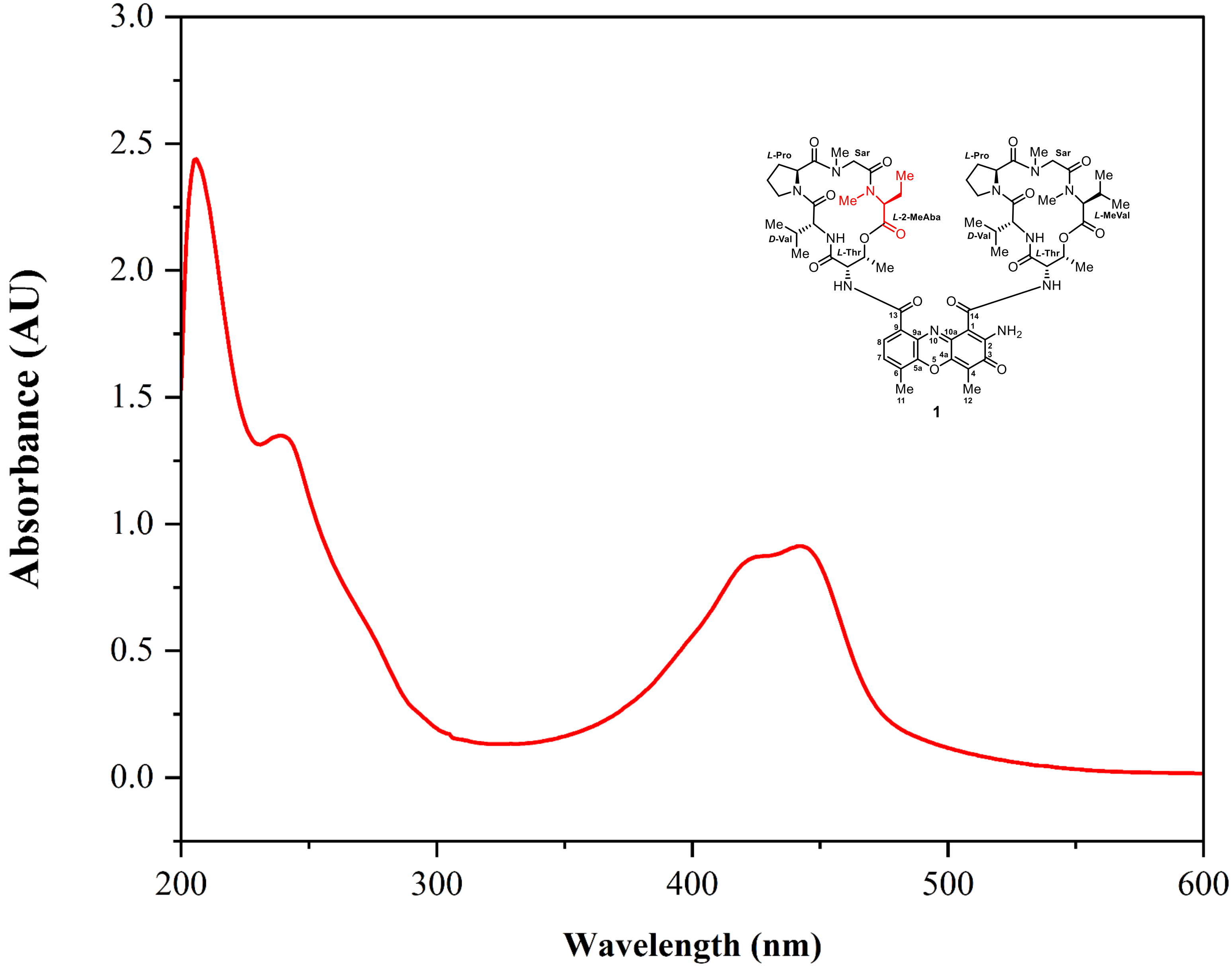

Figure S2. HR-ESIMS for compound **1**

Mass Spectrum SmartFormula Report

Analysis Info

Analysis Name	D:\Data\MS\data\202505\panghe_S-10-1_pos_31_01_19976.d	Acquisition Date	5/29/2025 5:33:05 PM
Method	LC_Direct Infusion_pos_100-1000mz.m	Operator	SCSIO
Sample Name	panghe_S-10-1_pos	Instrument	maXis 255552.00029
Comment			

Acquisition Parameter

Source Type	ESI	Ion Polarity	Positive	Set Nebulizer	0.4 Bar
Focus	Active	Set Capillary	4500 V	Set Dry Heater	180 °C
Scan Begin	100 m/z	Set End Plate Offset	-500 V	Set Dry Gas	4.0 l/min
Scan End	2000 m/z	Set Charging Voltage	0 V	Set Divert Valve	Waste
		Set Corona	0 nA	Set APCI Heater	0 °C

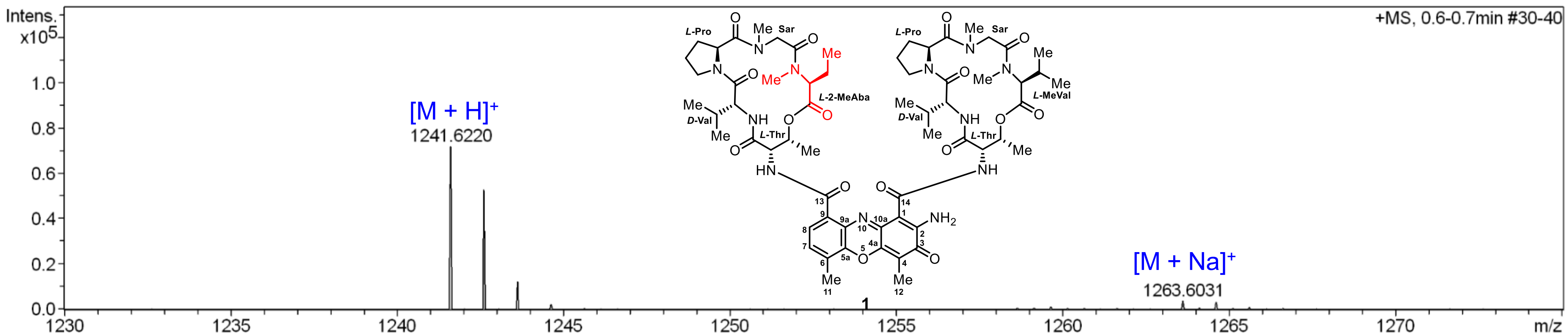

Meas. m/z	#	Ion Formula	Score	m/z	err [ppm]	err [mDa]	mSigma	rdb	e⁻ Conf	N-Rule
1241.6220	1	C61H85N12O16	100.00	1241.6201	1.5	1.9	52.2	25.5	even	ok
1263.6031	1	C61H84N12NaO16	100.00	1263.6020	0.8	1.0	39.7	25.5	even	ok

Figure S3. ^{1}H NMR (600 MHz) spectrum of compound **1** in $CDCl_3$

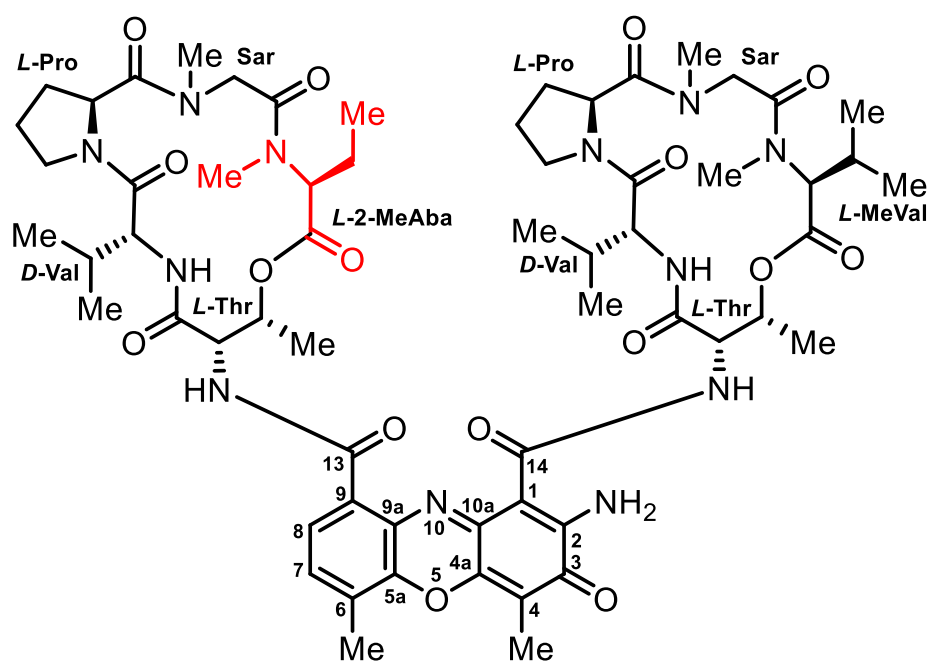

1

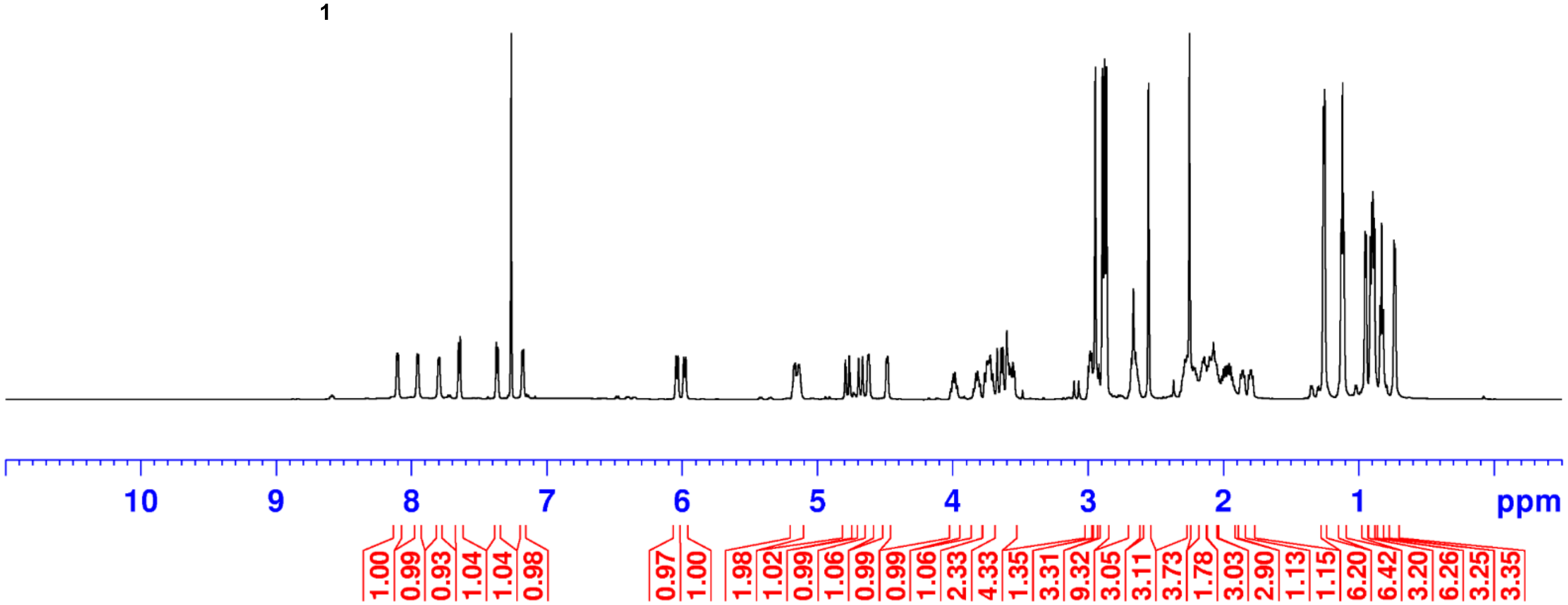

Figure S3-continue

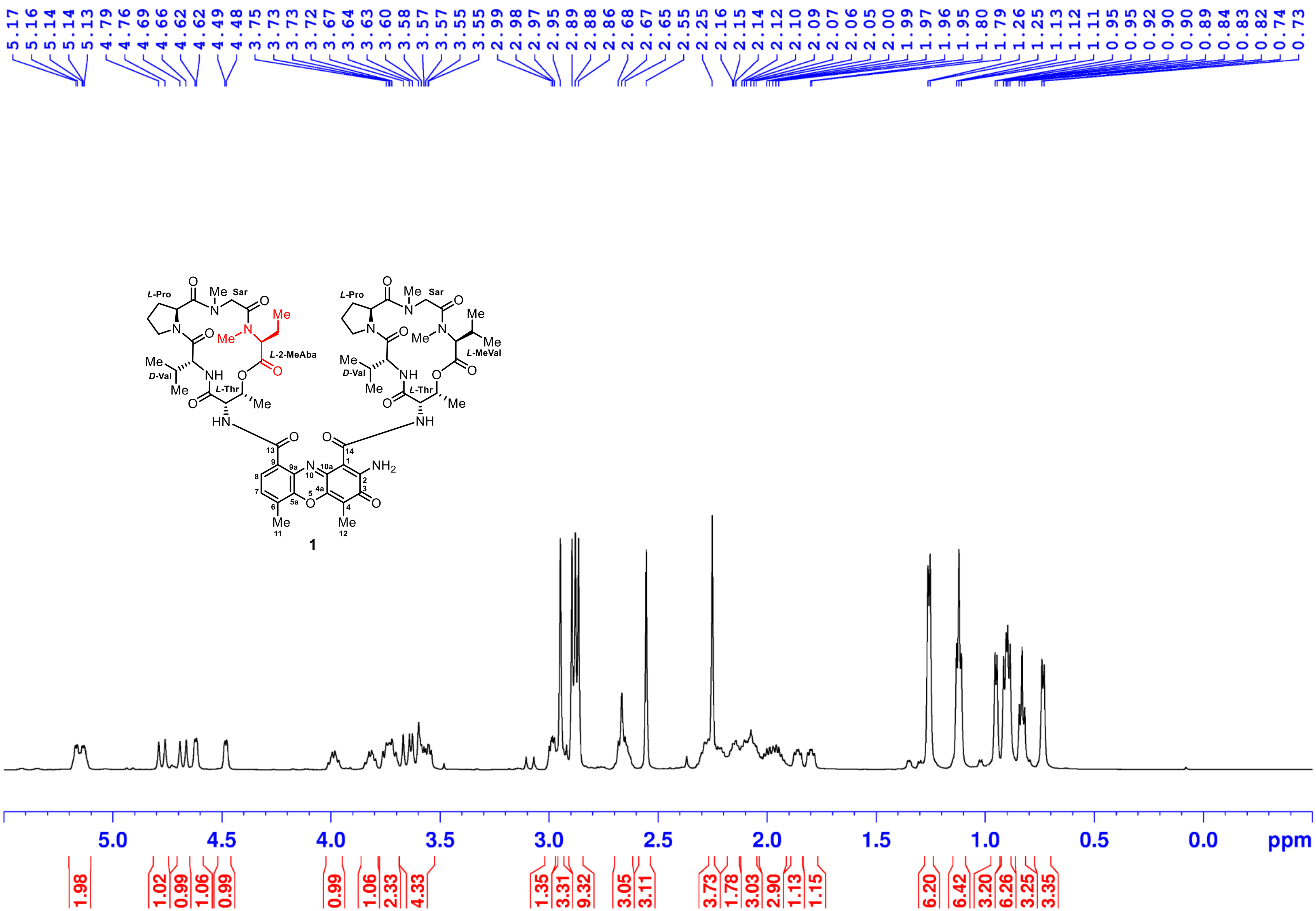

Figure S3-continue

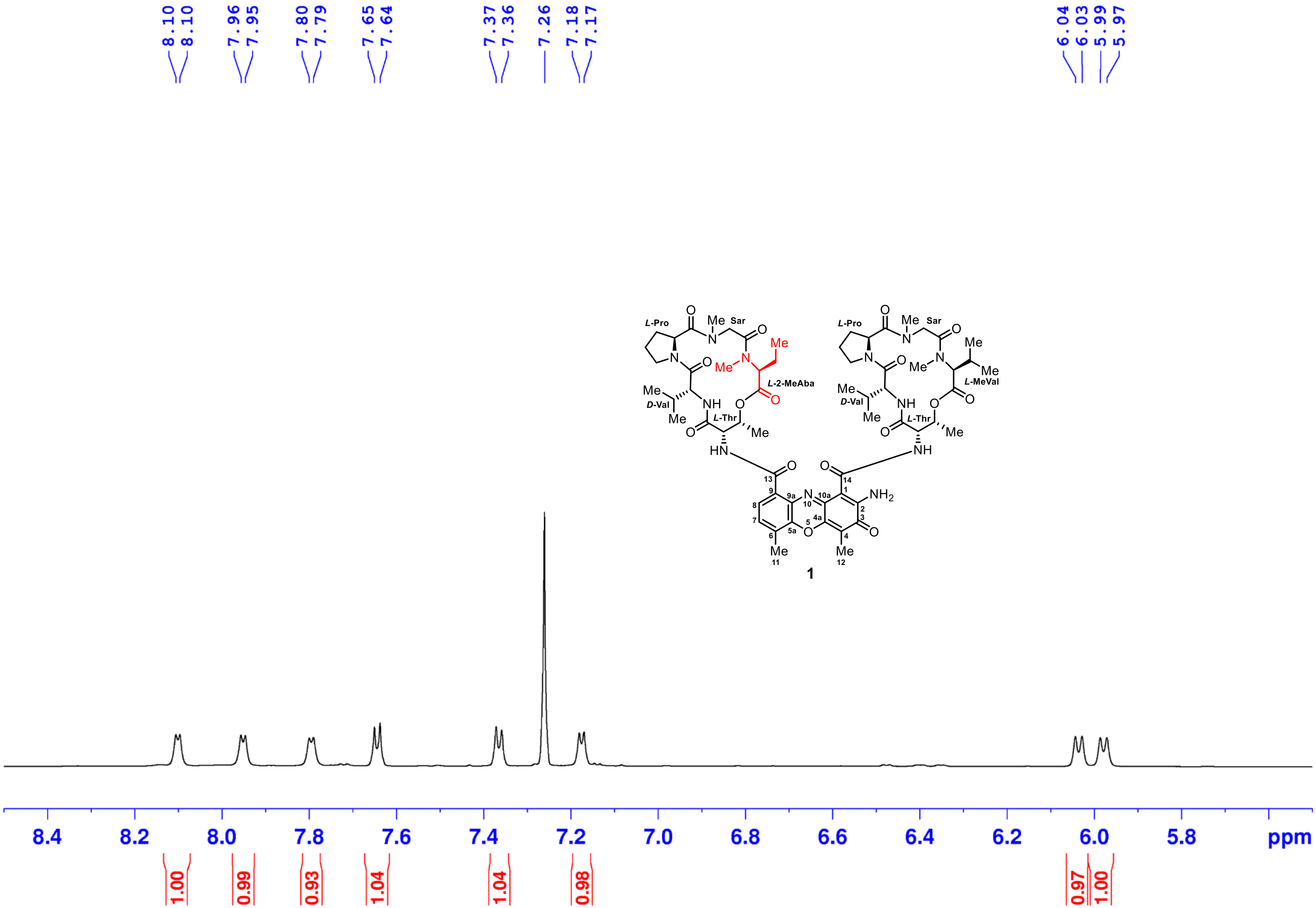

Figure S4.^{13}C NMR (150 MHz) spectrum of compound **1** in $CDCl_3$

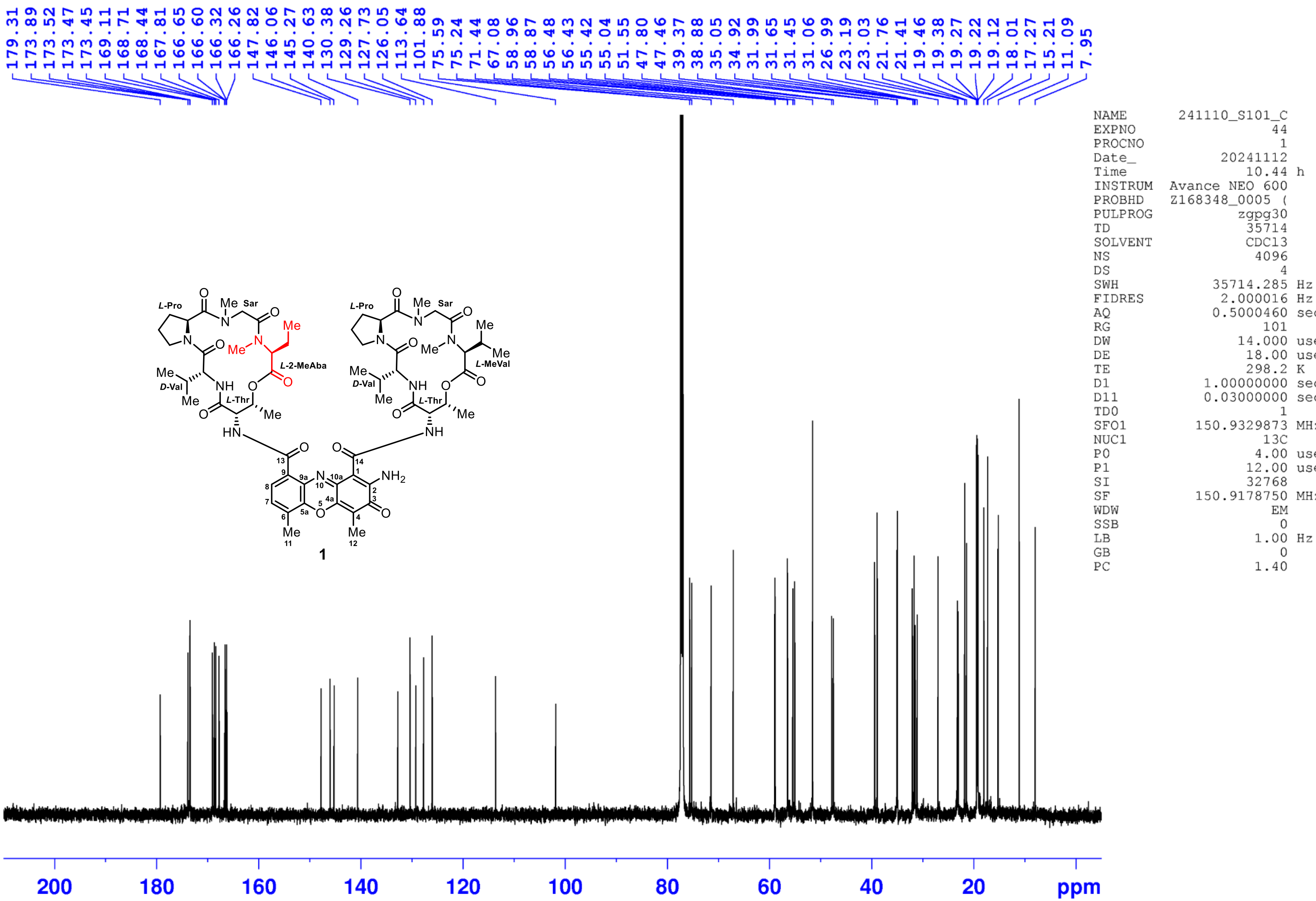

Figure S4-continue

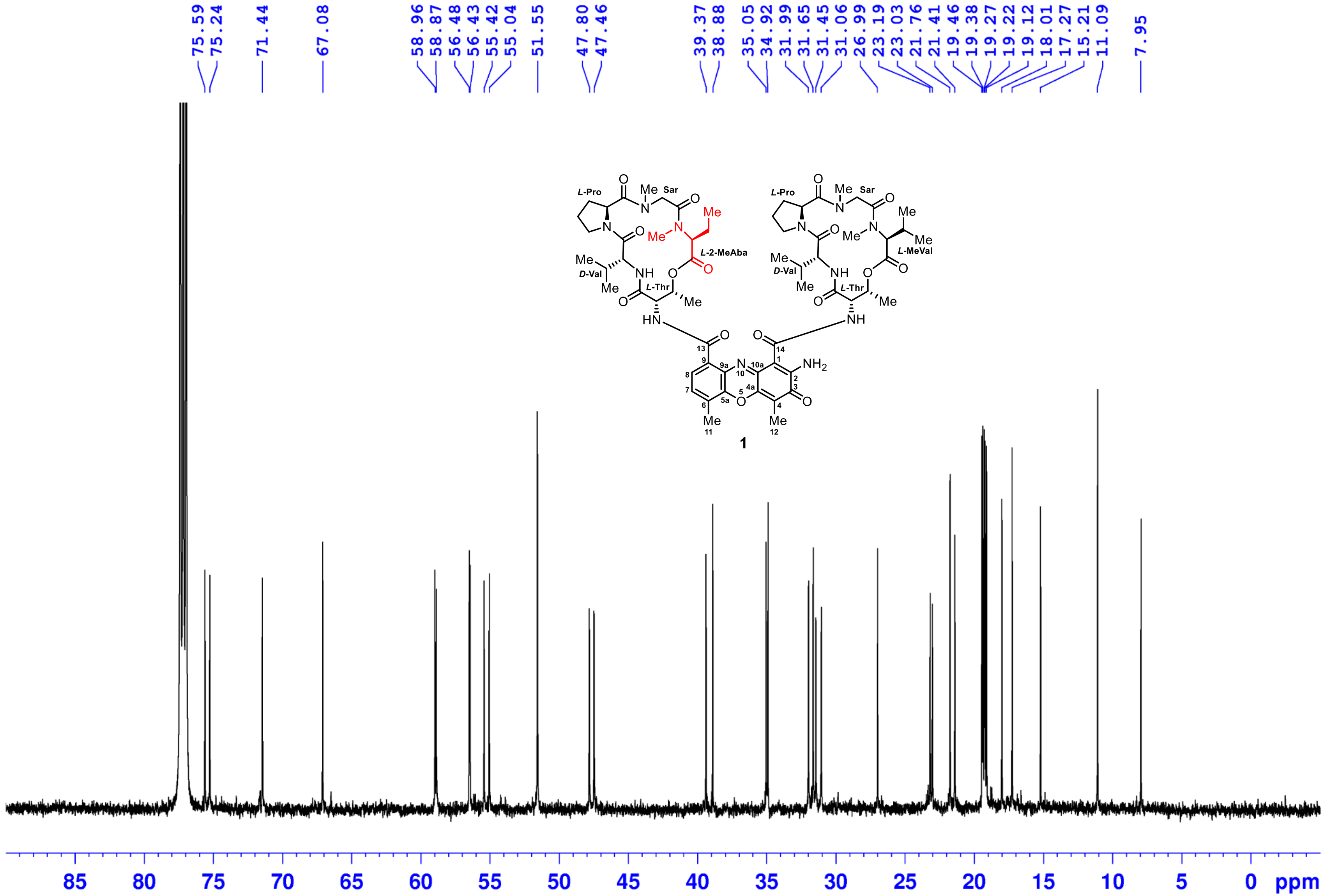

Figure S4-continue

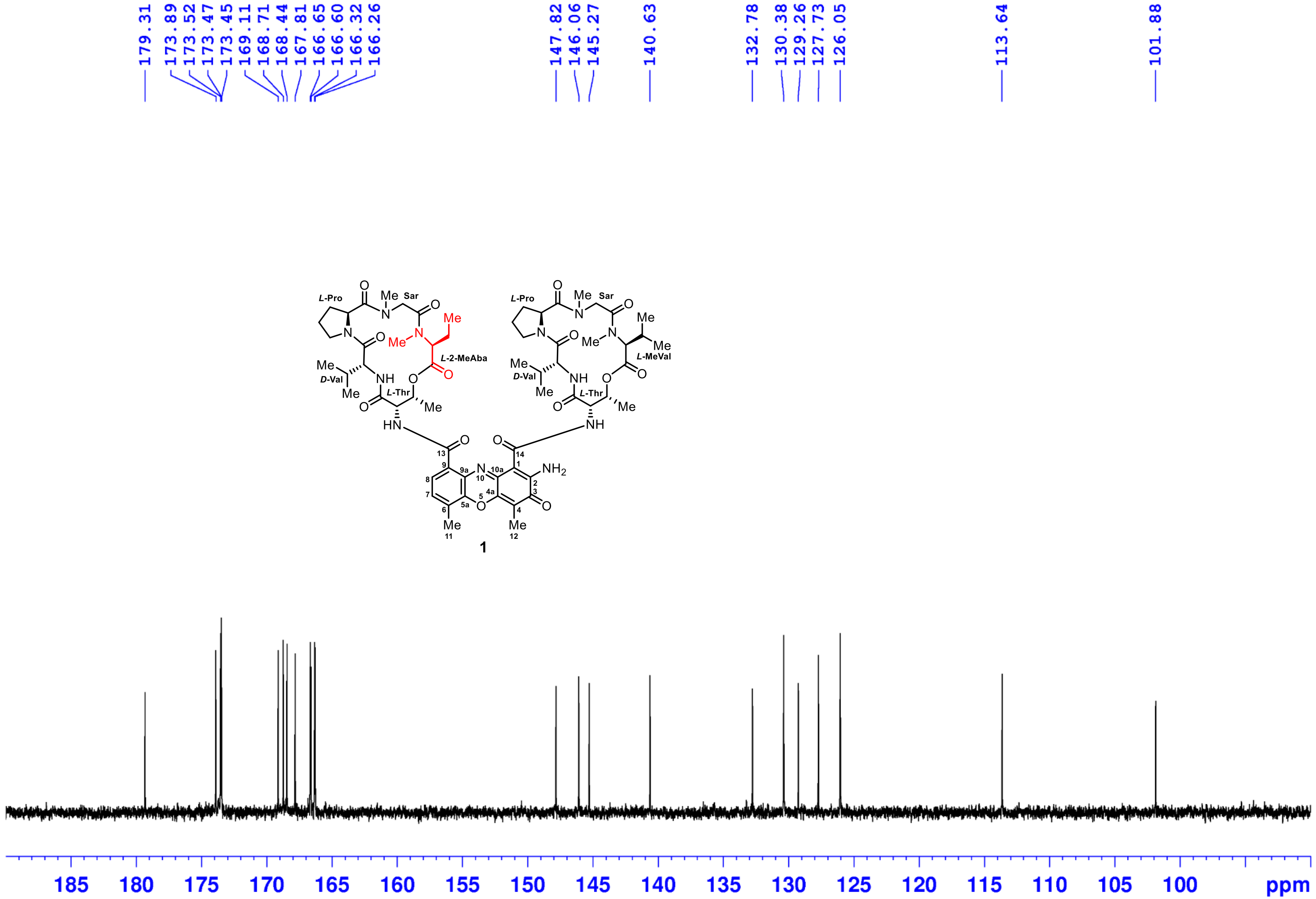

1

Figure S5. DEPT135 (150 MHz) spectrum of compound **1** in $CDCl_3$

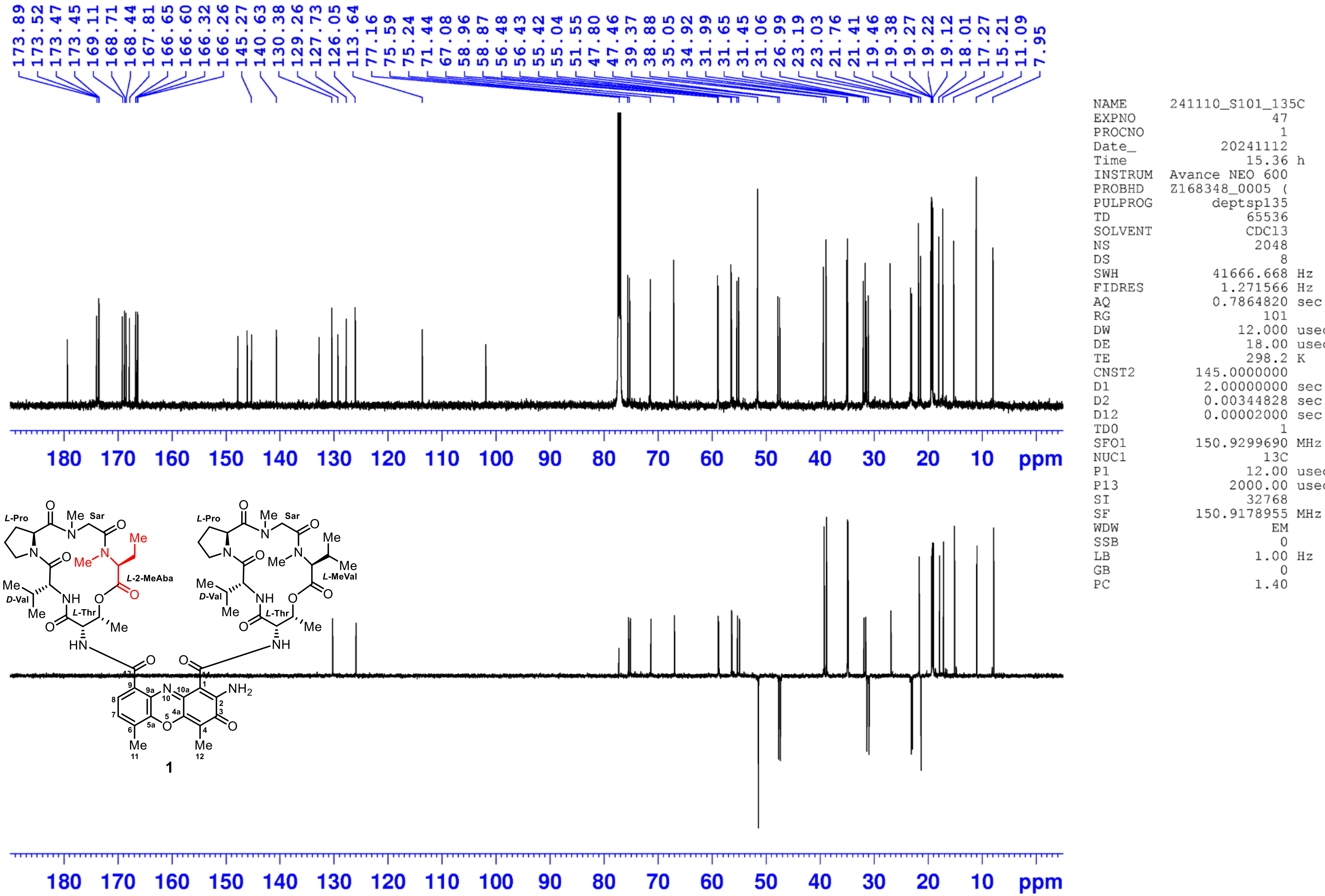

Figure S5-continue

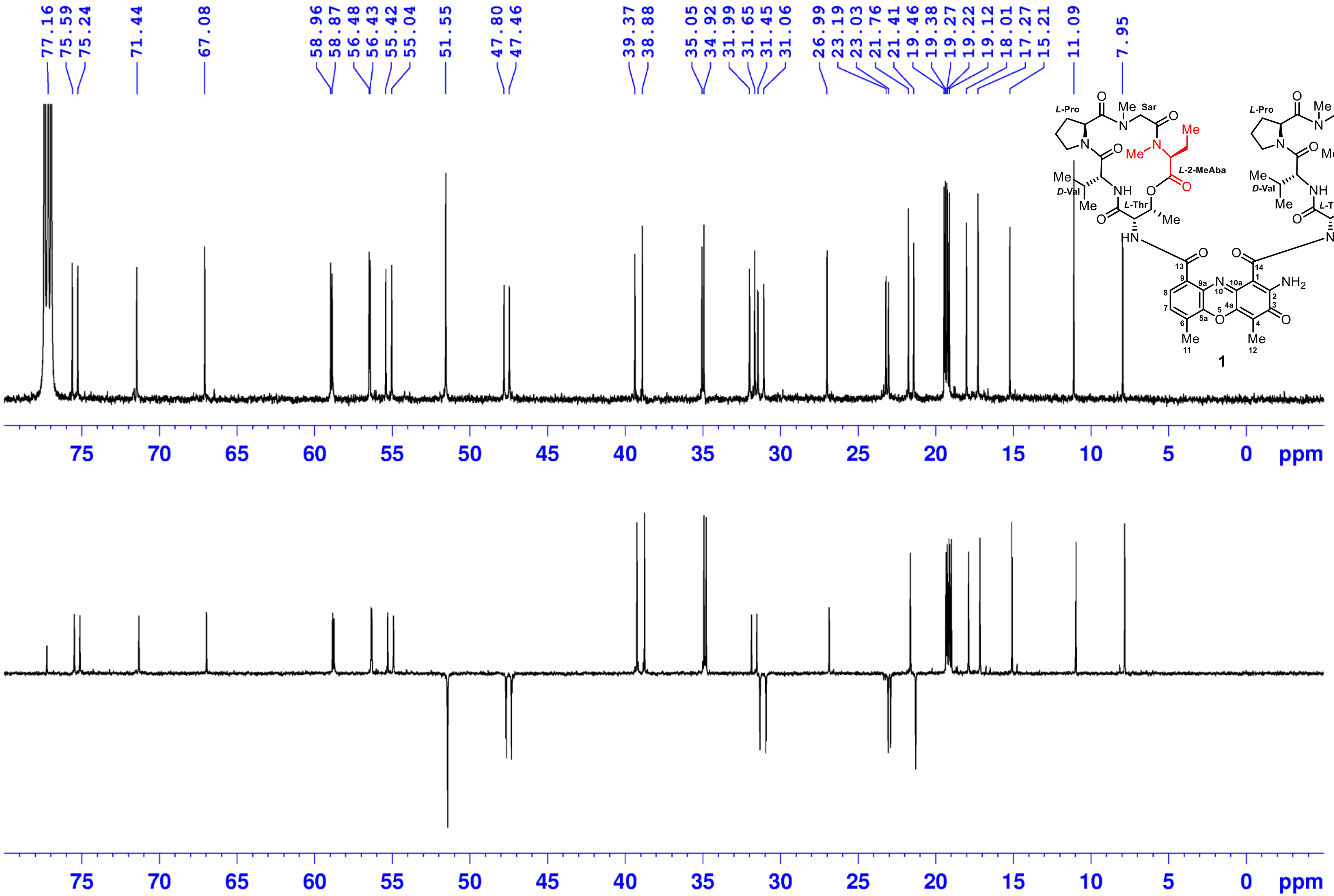

Figure S5-continue

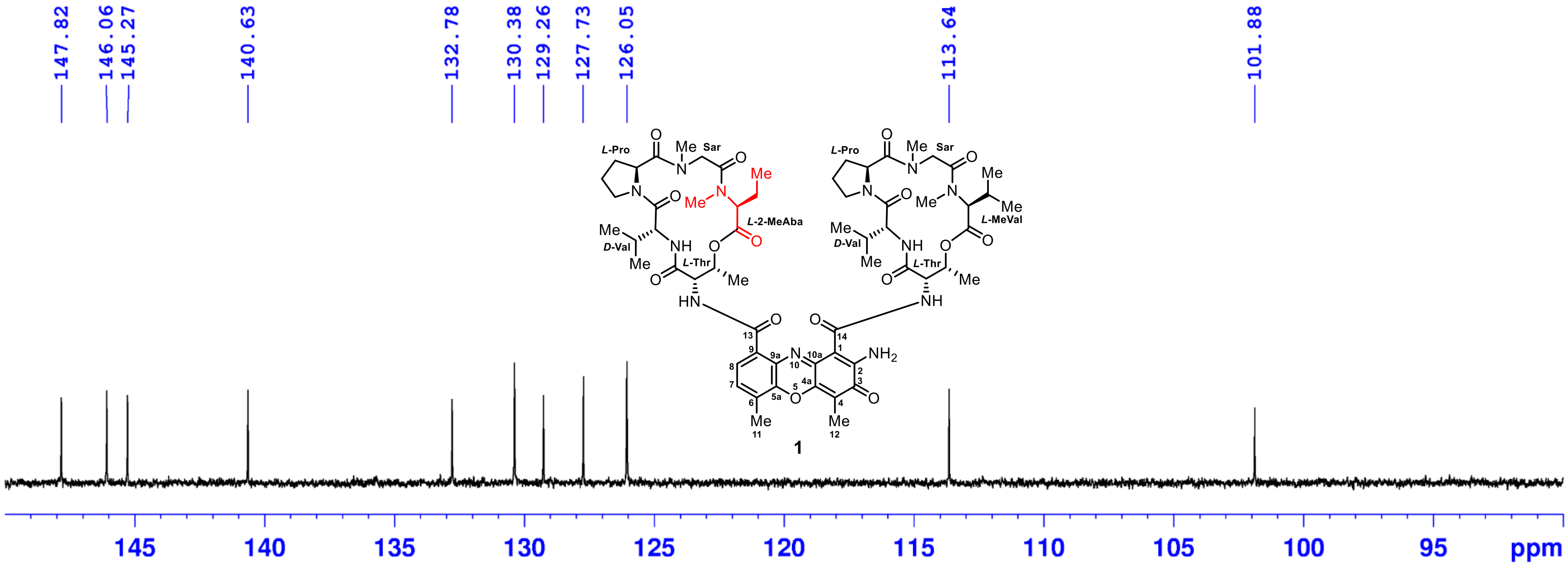

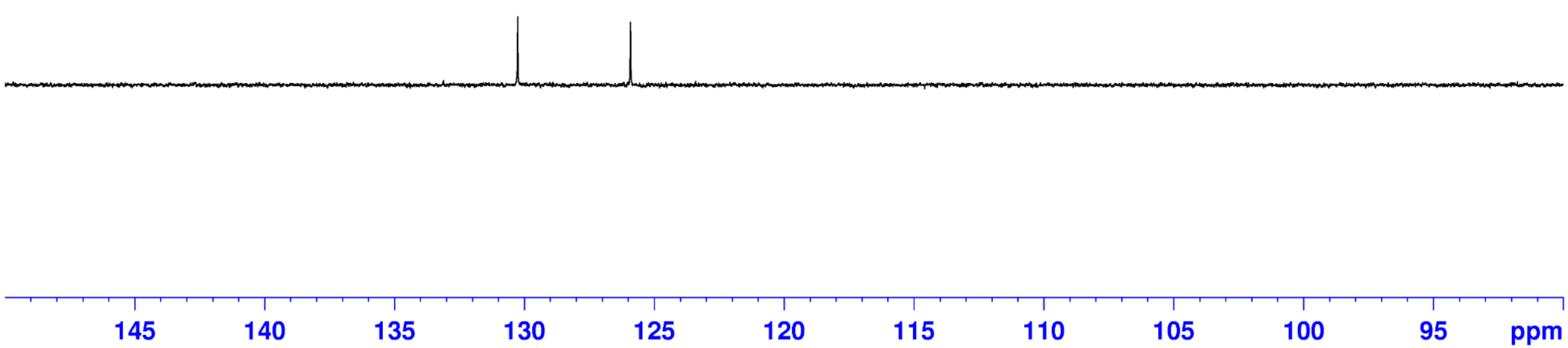

Figure S6. ^{1}H–^{1}H COSY (600 MHz) spectrum of compound **1** in $CDCl_3$

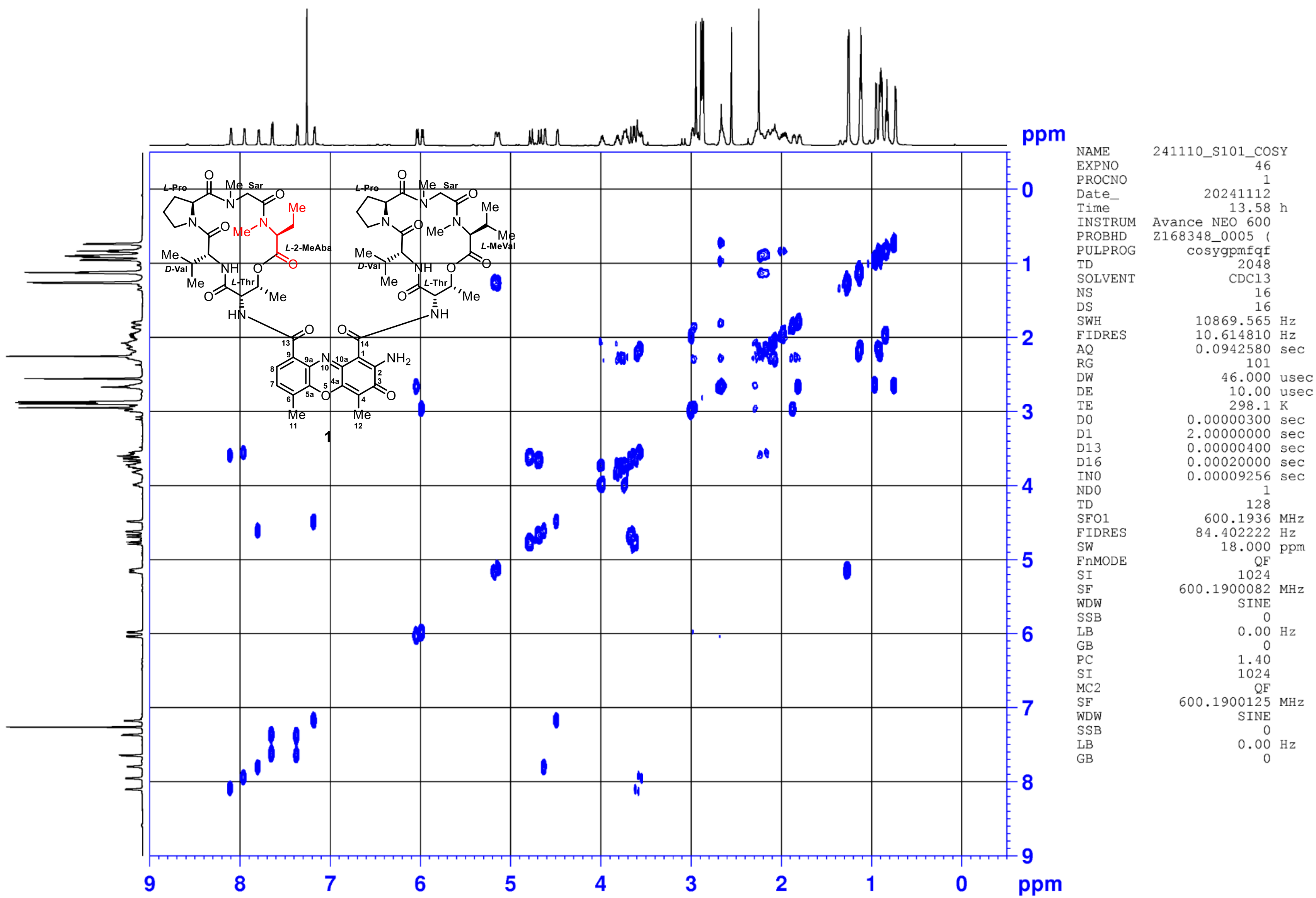

Figure S6-continue

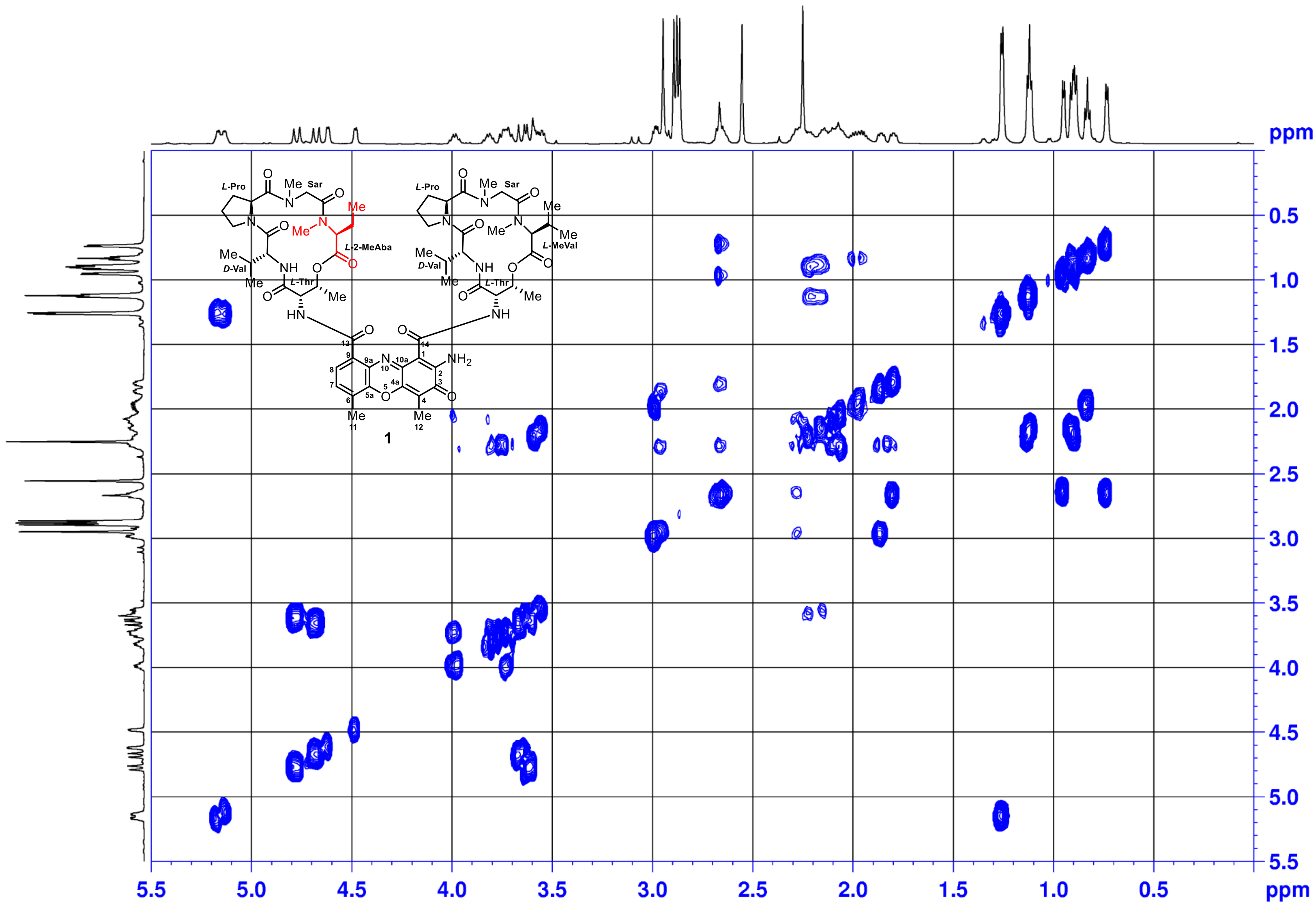

Figure S6-continue

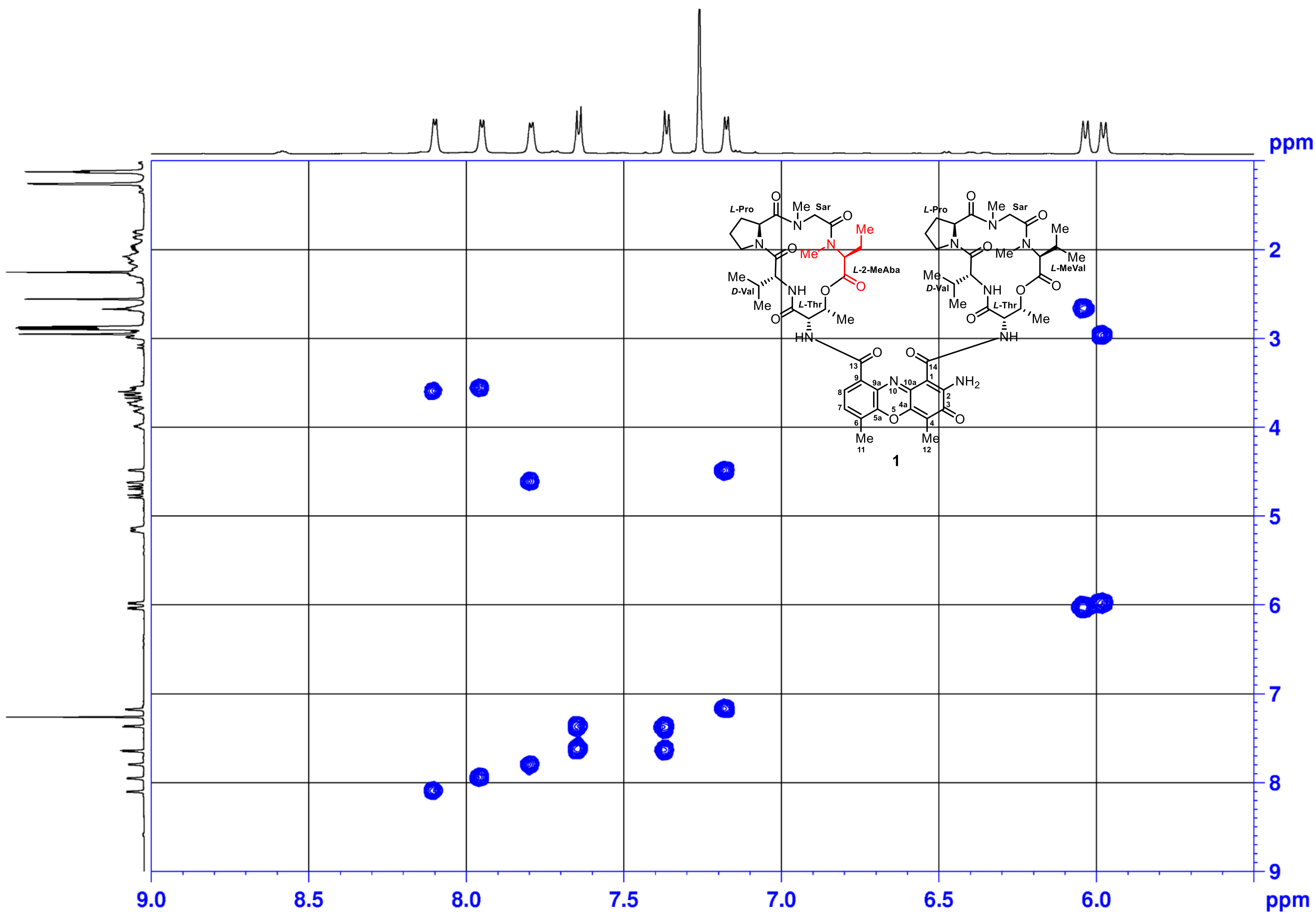

Figure S7. HSQC (600 MHz) spectrum of compound **1** in $CDCl_3$

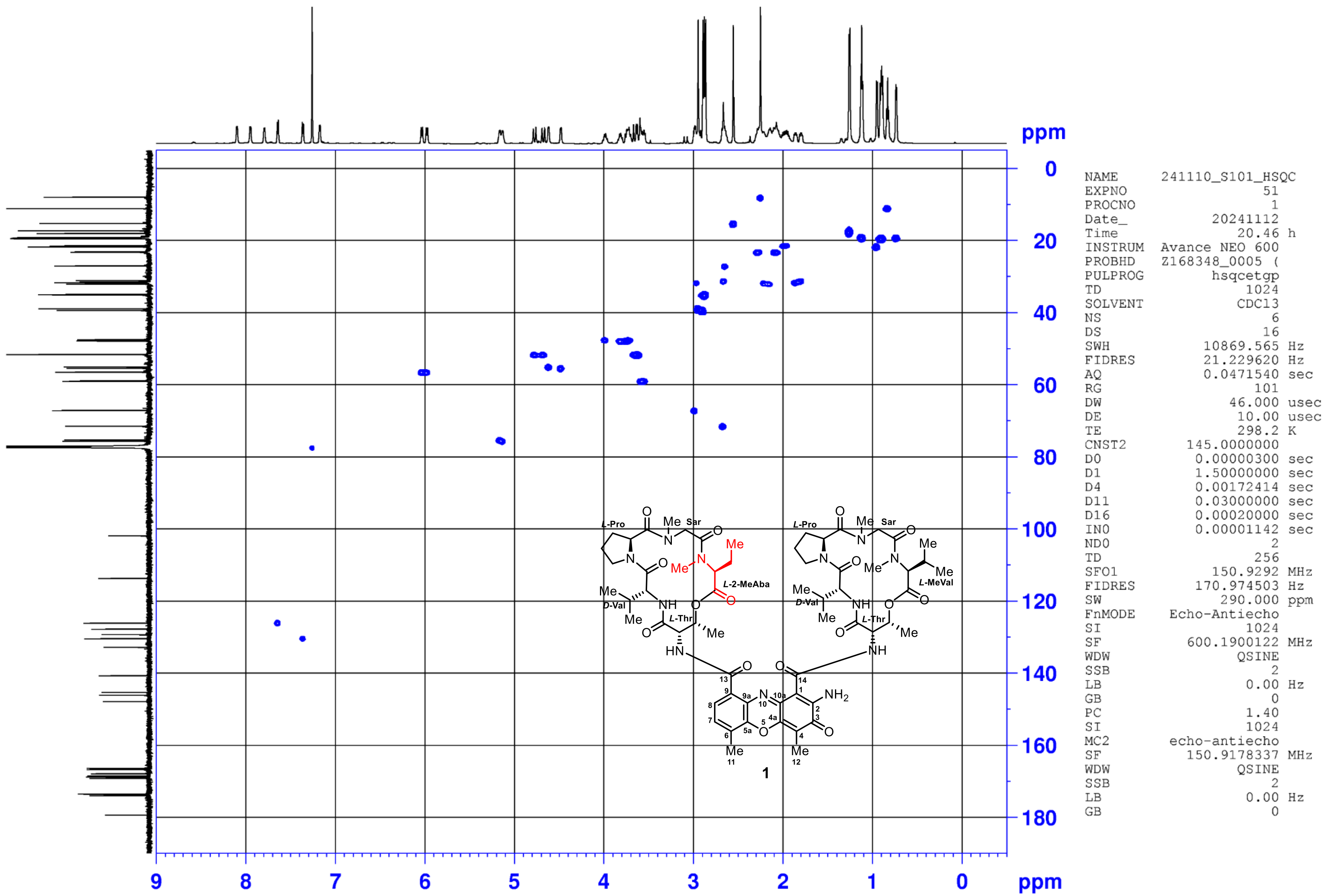

Figure S7-continue

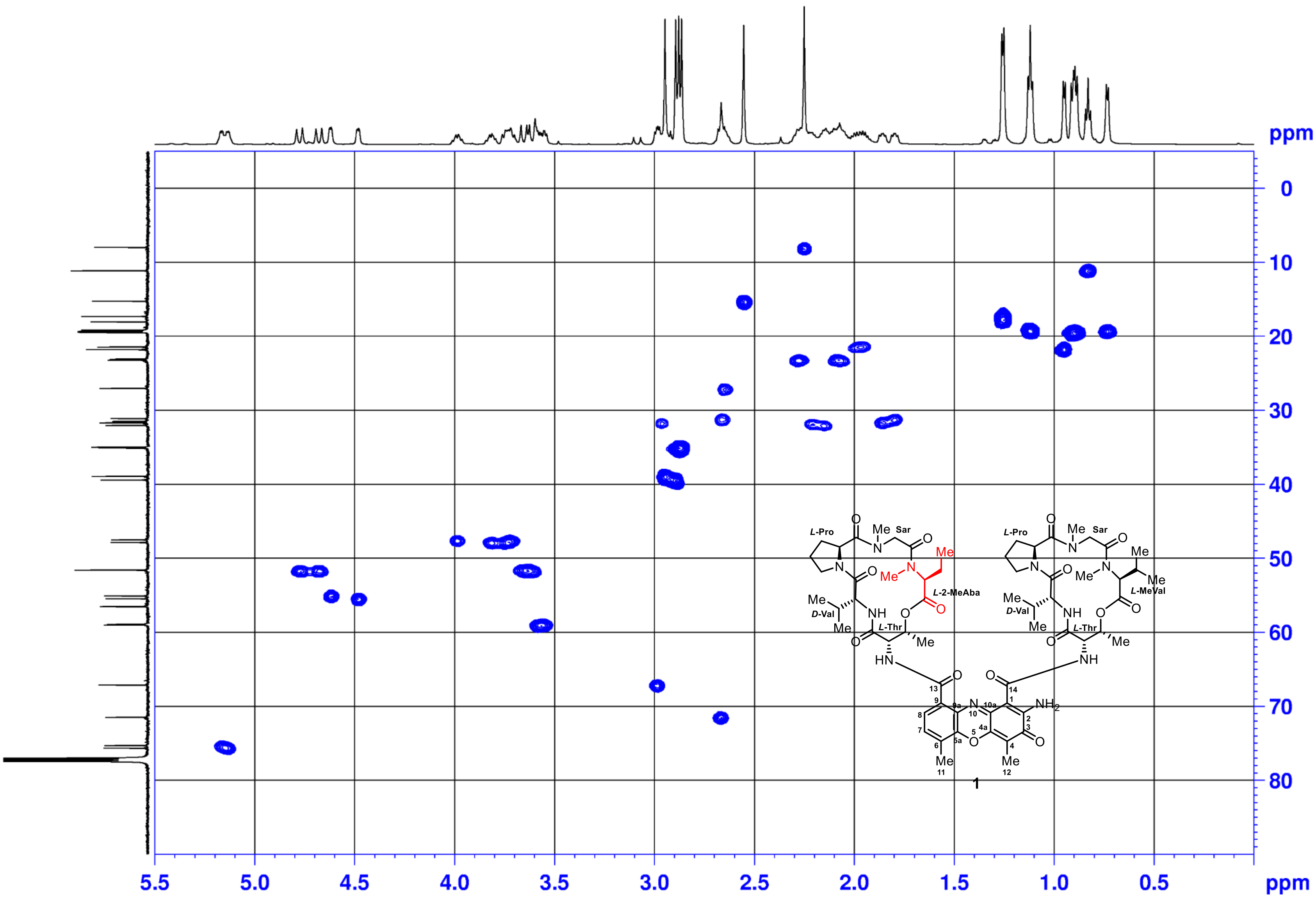

Figure S7-continue

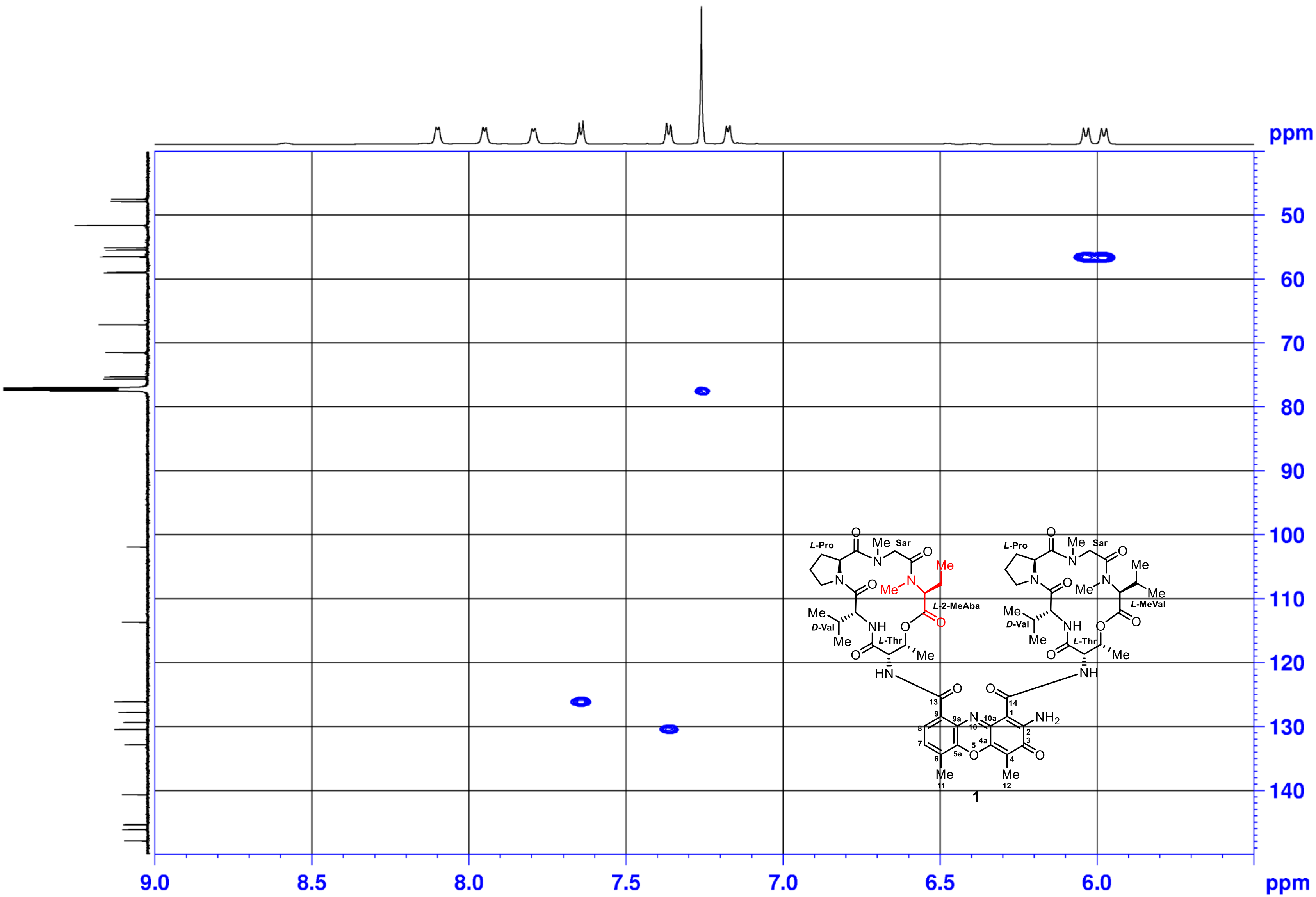

Figure S8. HMBC (600 MHz) spectrum of compound **1** in $CDCl_3$

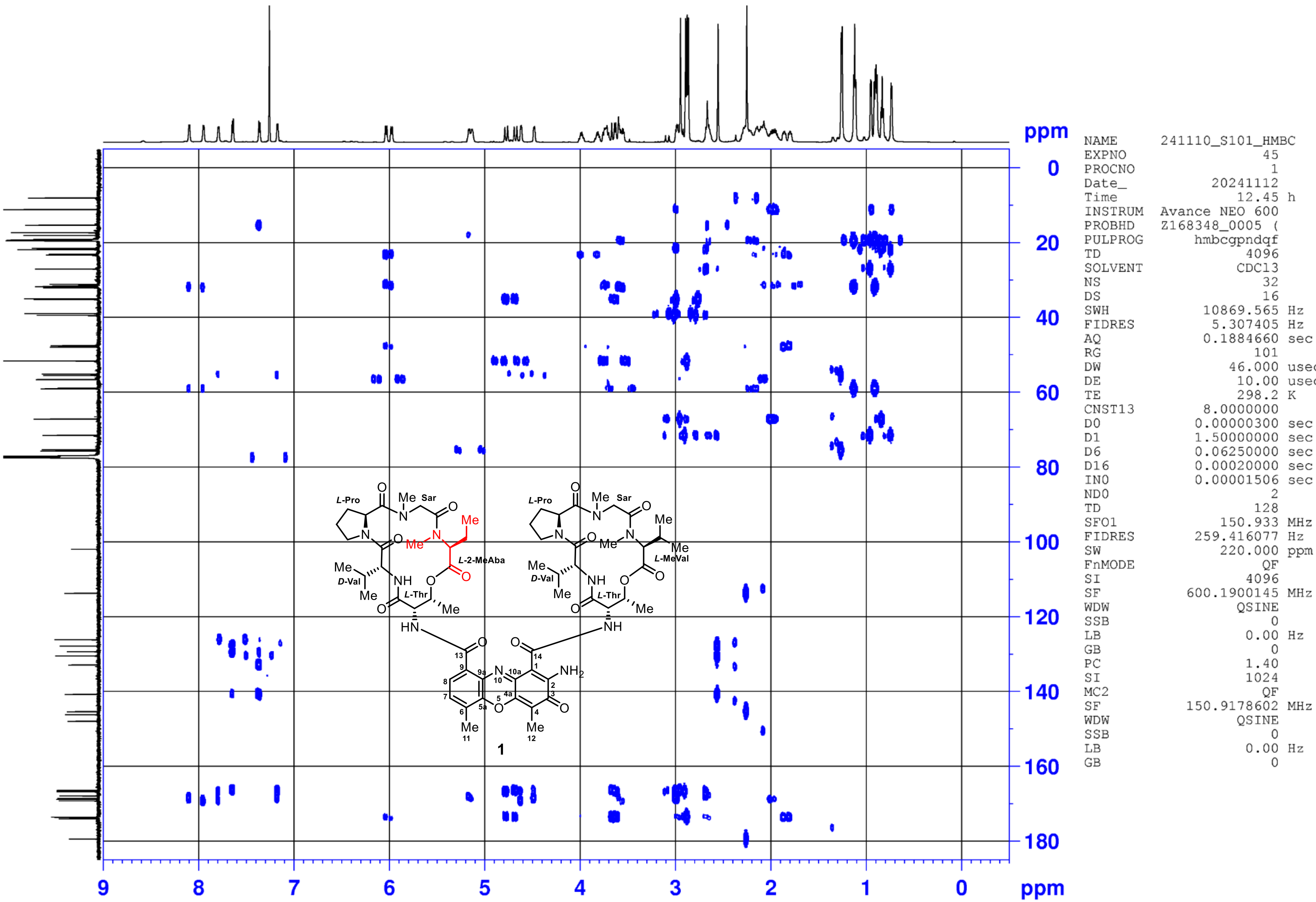

Figure S8-continue

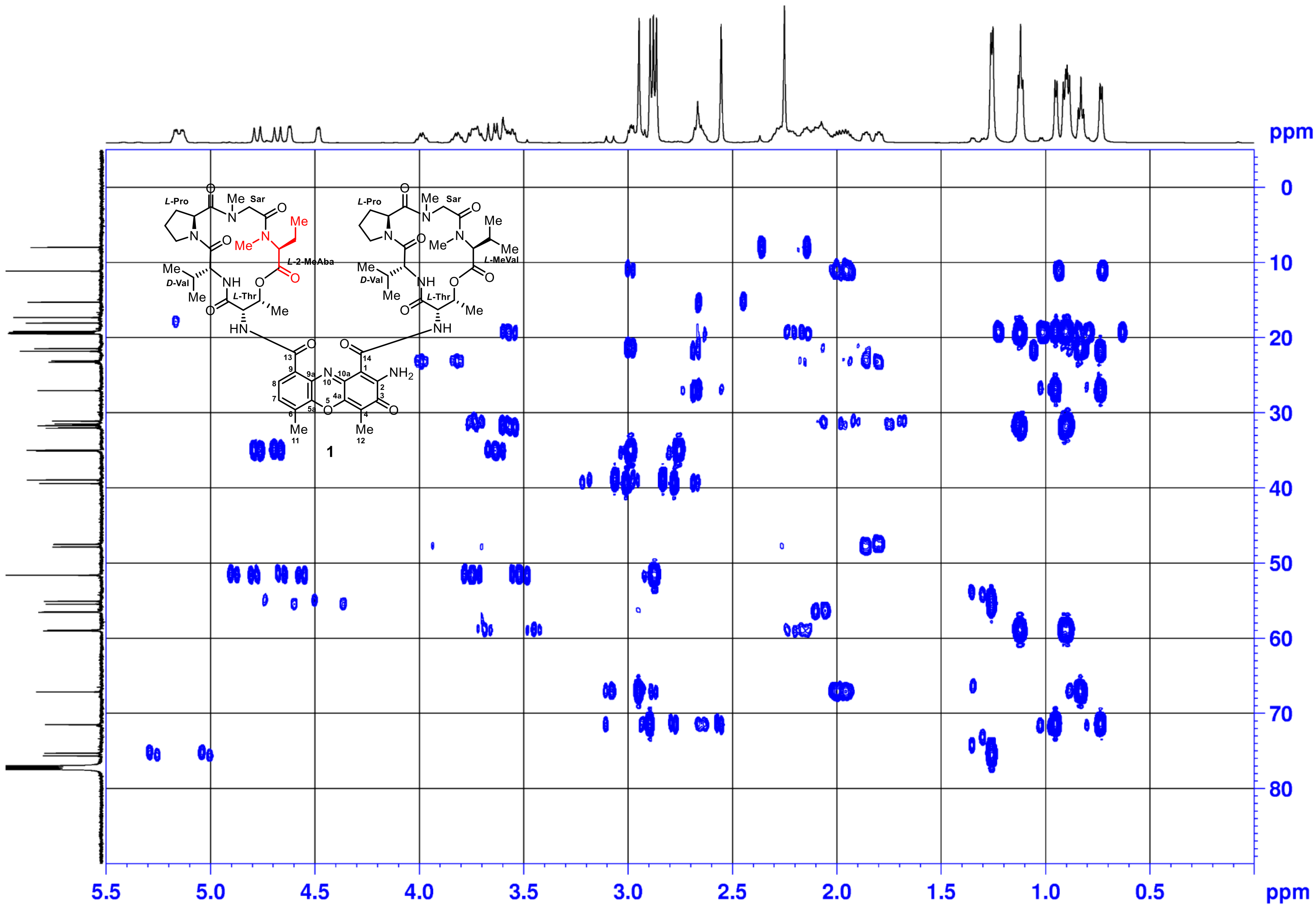

Figure S8-continue

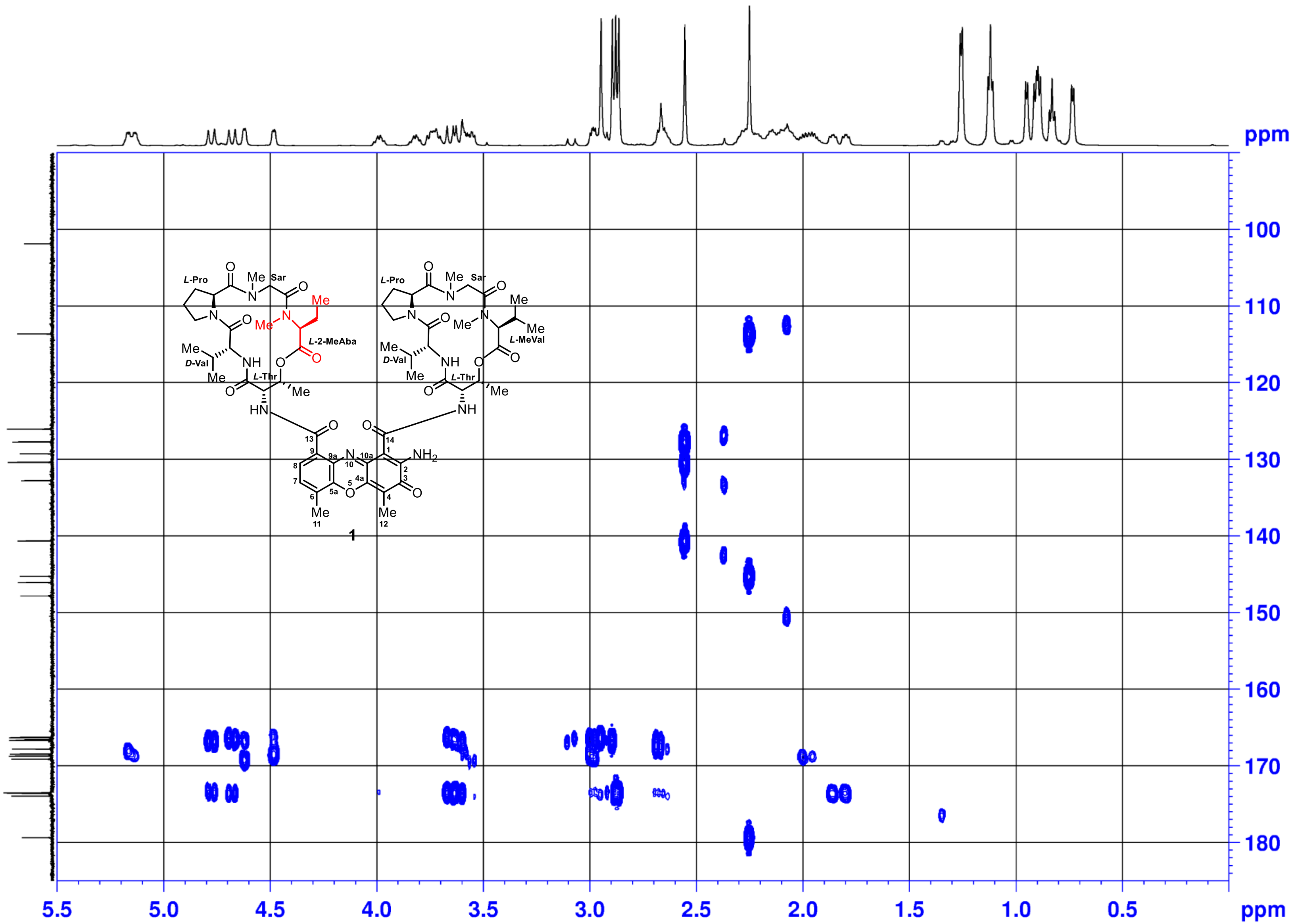

Figure S8-continue

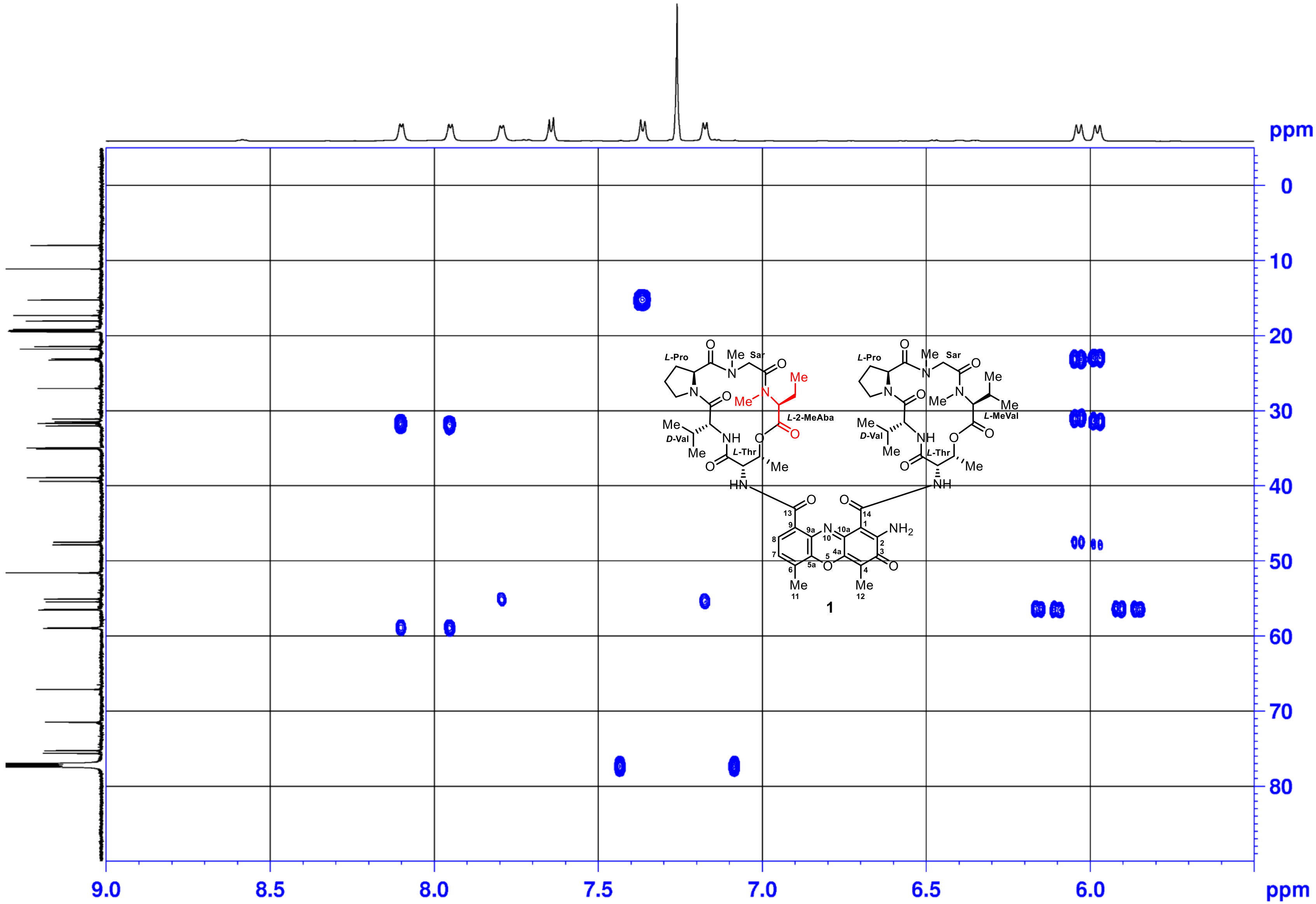

Figure S8-continue

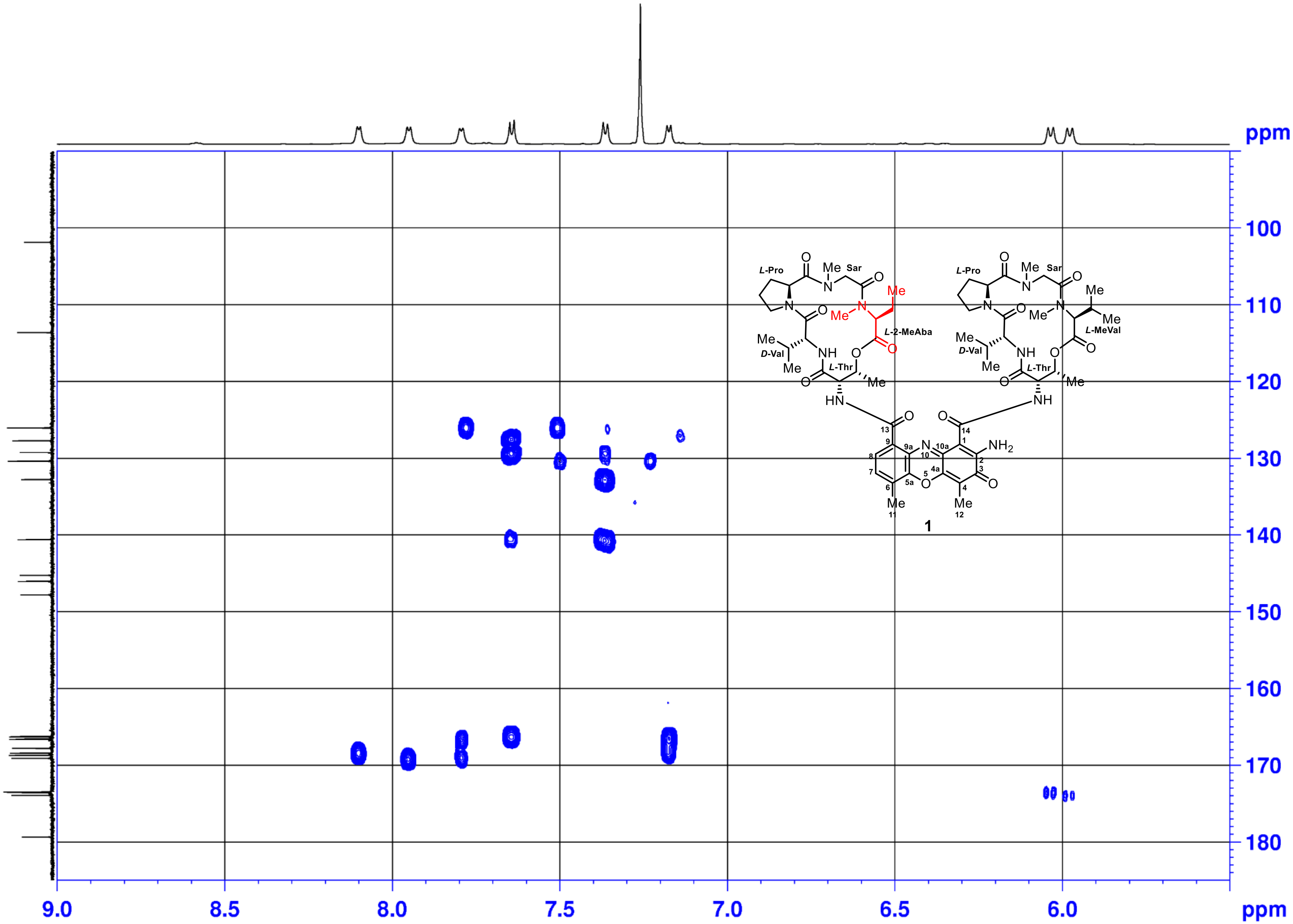

Figure S9. NOESY (600 MHz) spectrum of compound **1** in $CDCl_3$

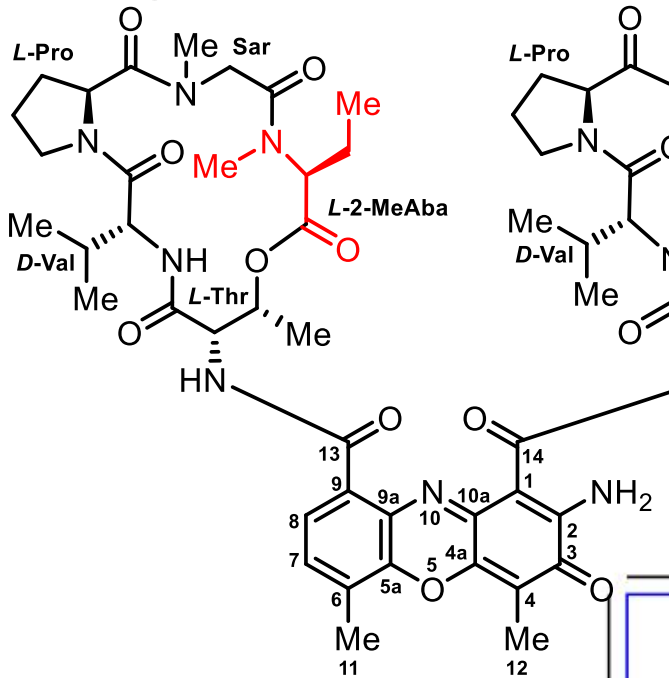

1

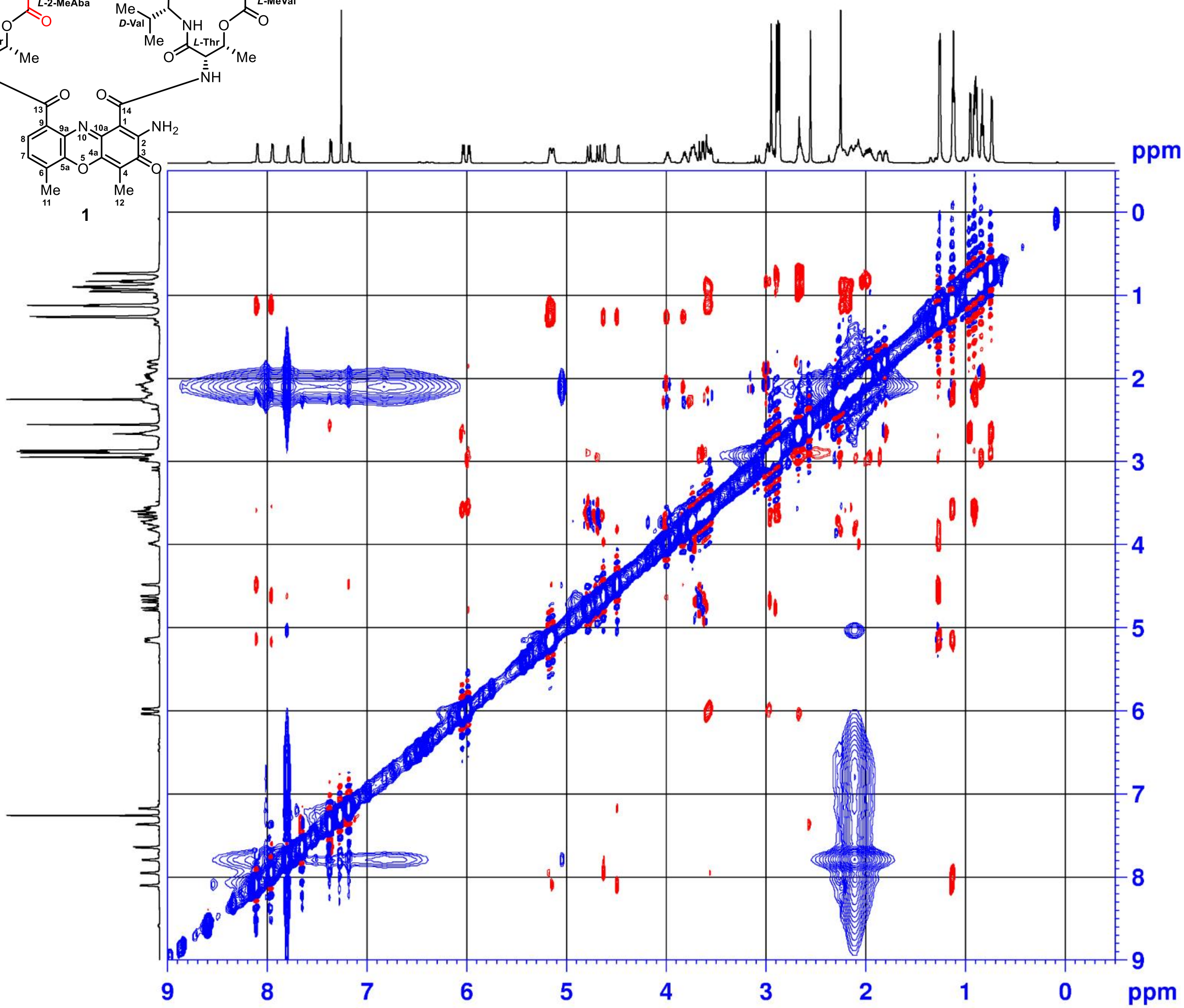

NAME	241110_S101_NOESY
EXPNO	49
PROCNO	1
Date_	20241112
Time	20.05 h
INSTRUM	Avance NEO 600
PROBHD	Z168348_0005 (
PULPROG	noesygpphpp
TD	2048
SOLVENT	CDCl3
NS	16
DS	32
SWH	11904.762 Hz
FIDRES	11.625744 Hz
AQ	0.0860660 sec
RG	101
DW	42.000 usec
DE	10.66 usec
TE	298.1 K
D0	0.00003147 sec
D1	2.00000000 sec
D8	0.30000001 sec
D11	0.03000000 sec
D12	0.00002000 sec
D16	0.00020000 sec
IN0	0.00008331 sec
ND0	1
TD	256
SFO1	600.1928 MHz
FIDRES	46.890064 Hz
SW	20.000 ppm
FnMODE	States-TPPI
SI	1024
SF	600.1900071 MHz
WDW	QSINE
SSB	2
LB	0.00 Hz
GB	0
PC	1.00
SI	1024
MC2	States-TPPI
SF	600.1900106 MHz
WDW	QSINE
SSB	2
LB	0.00 Hz
GB	0

Figure S9-continue

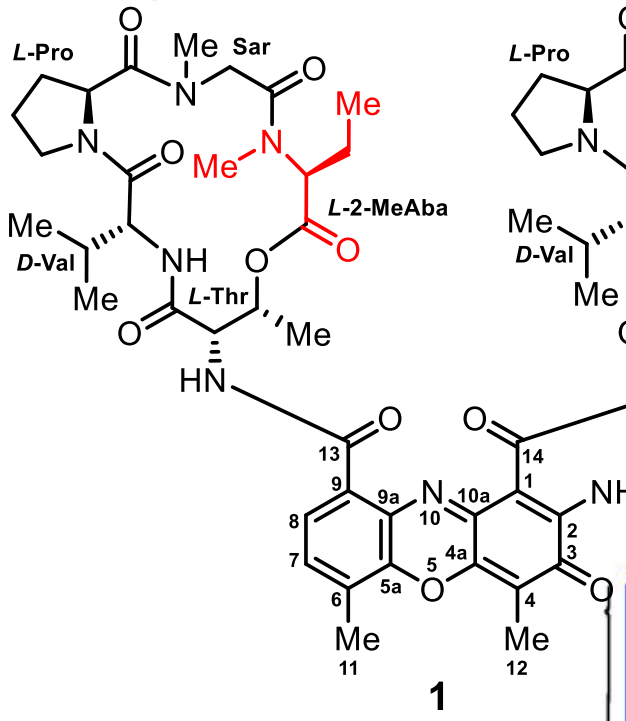

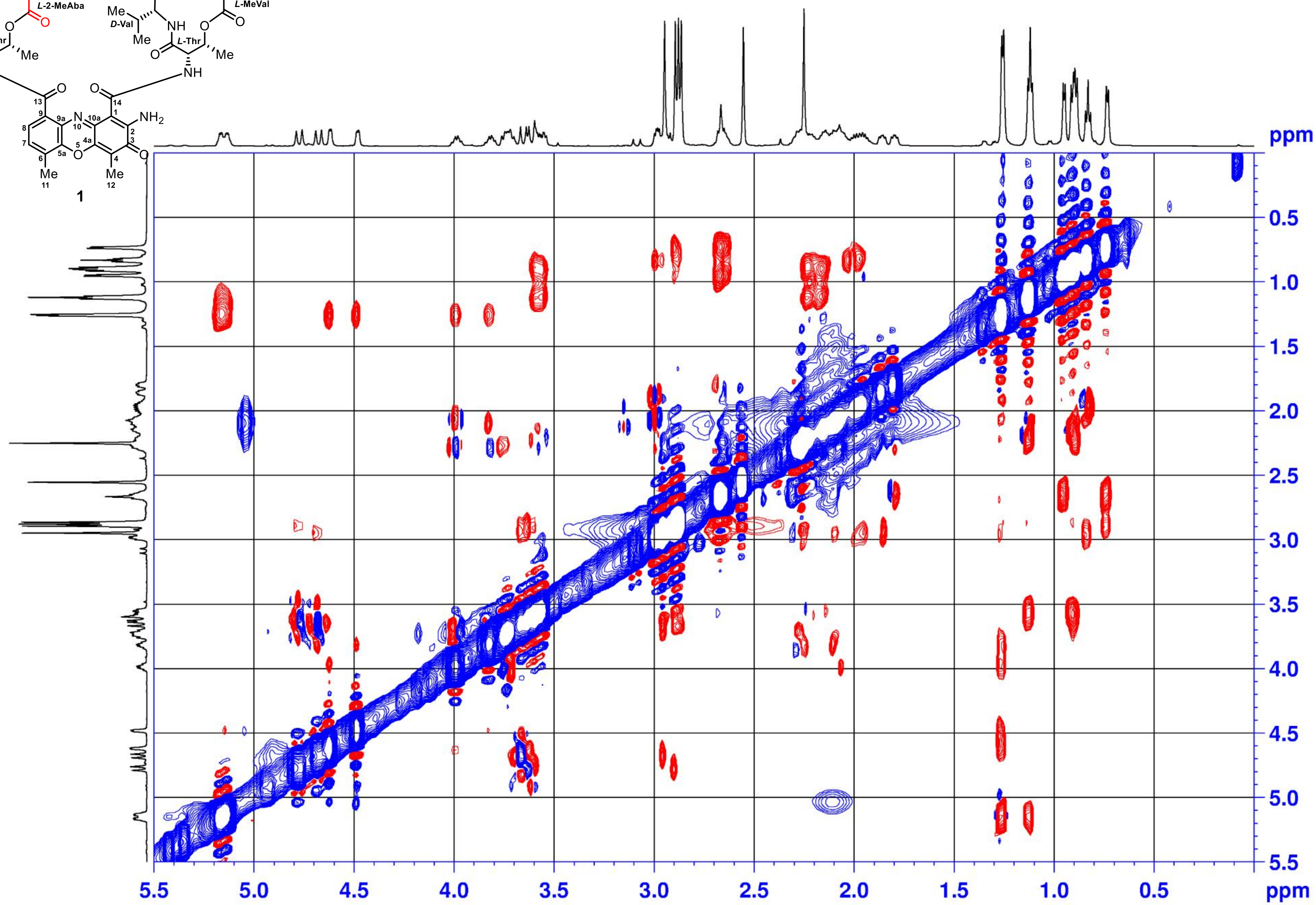

Figure S9-continue

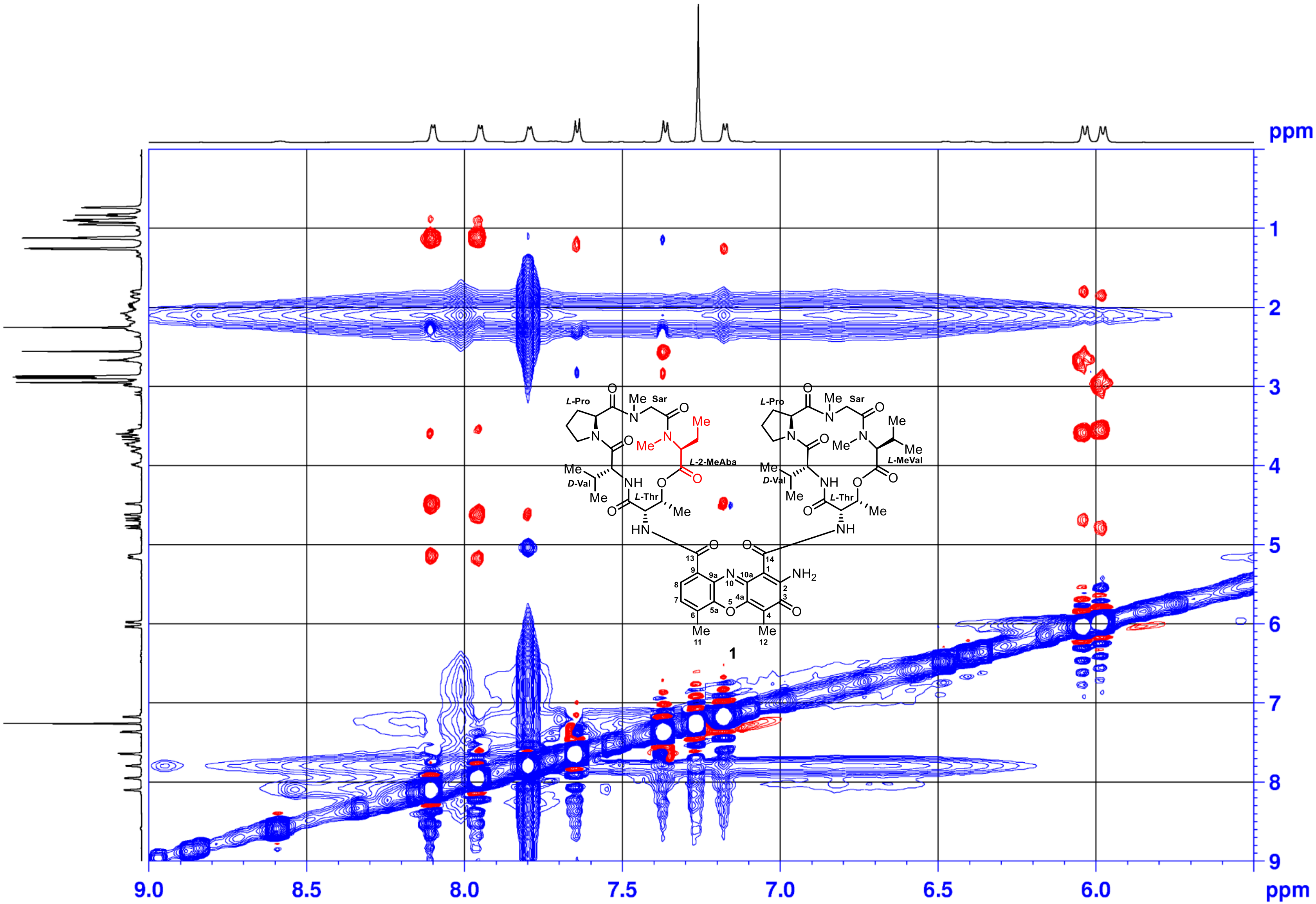

Figure S10. Marfey's analysis of Sar, Val, and Pro standards.

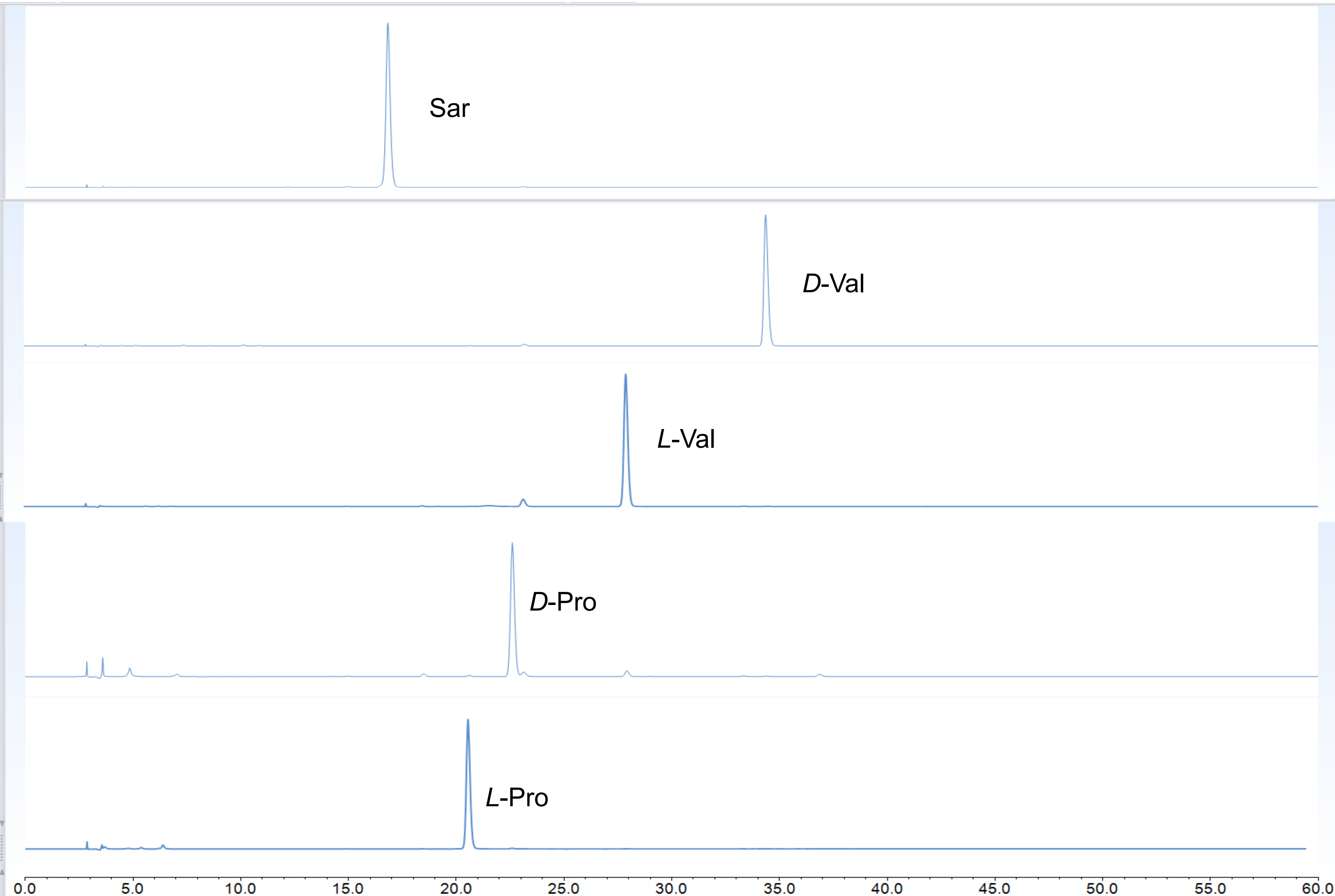

Figure S11. Marfey's analysis of MeVal, and Thr standards.

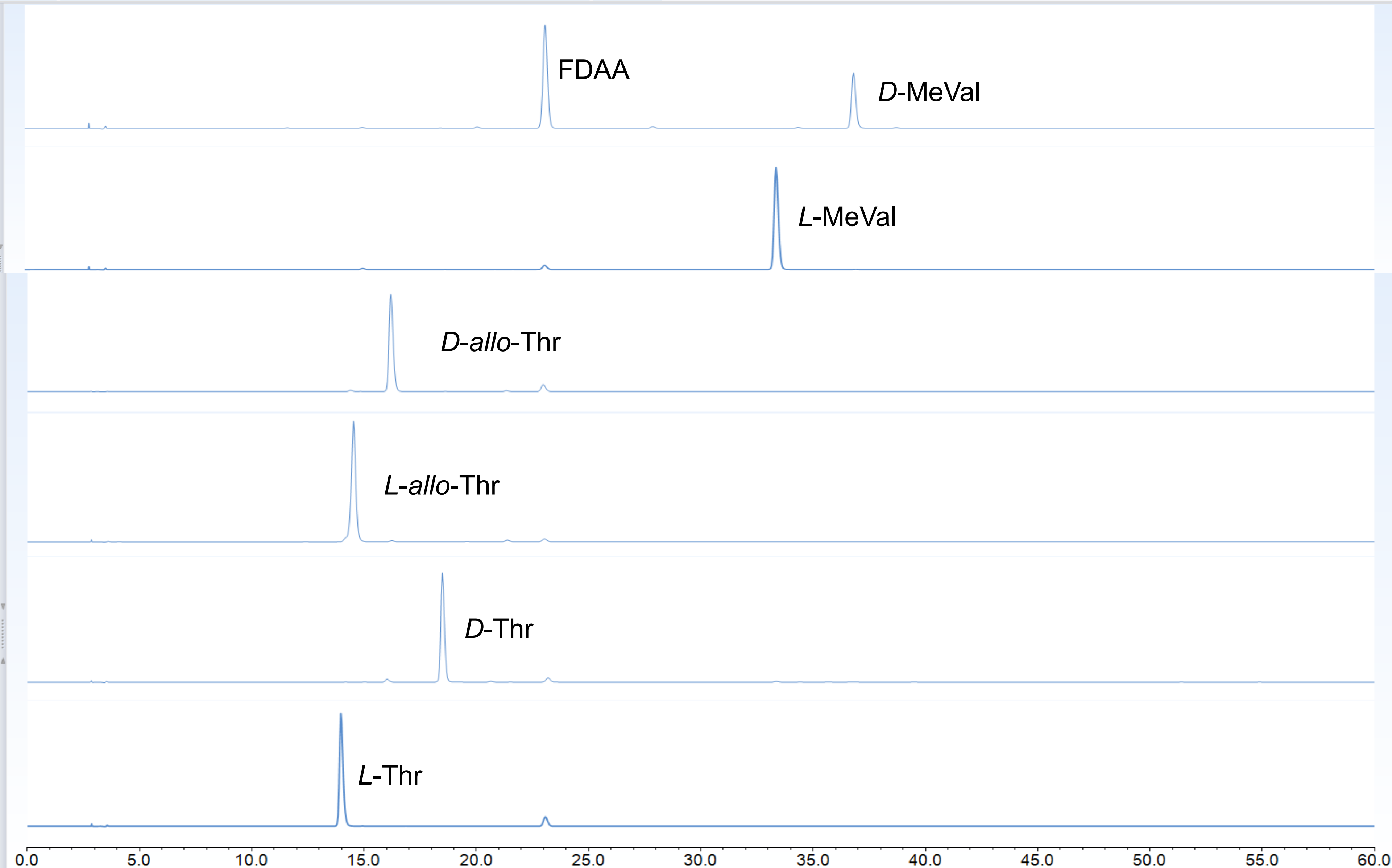

Figure S12. Marfey's analysis of MeAba standard.

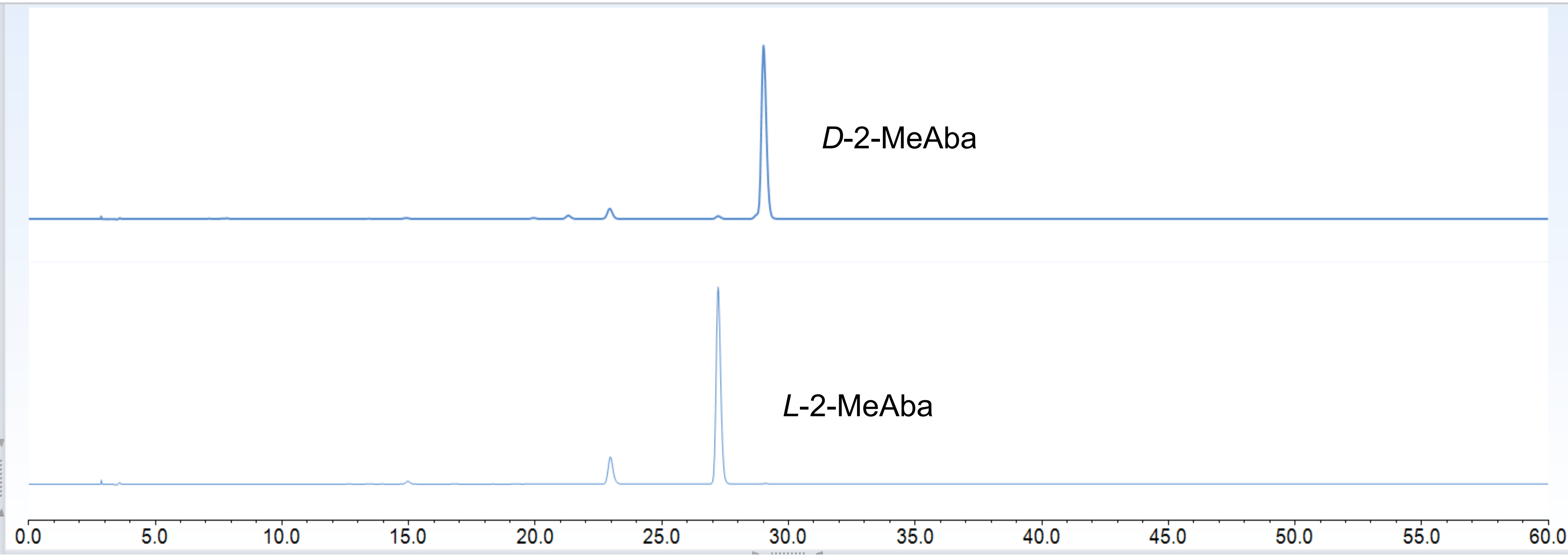

Figure S13. Marfey's analysis of compound **1** and standard amino acids

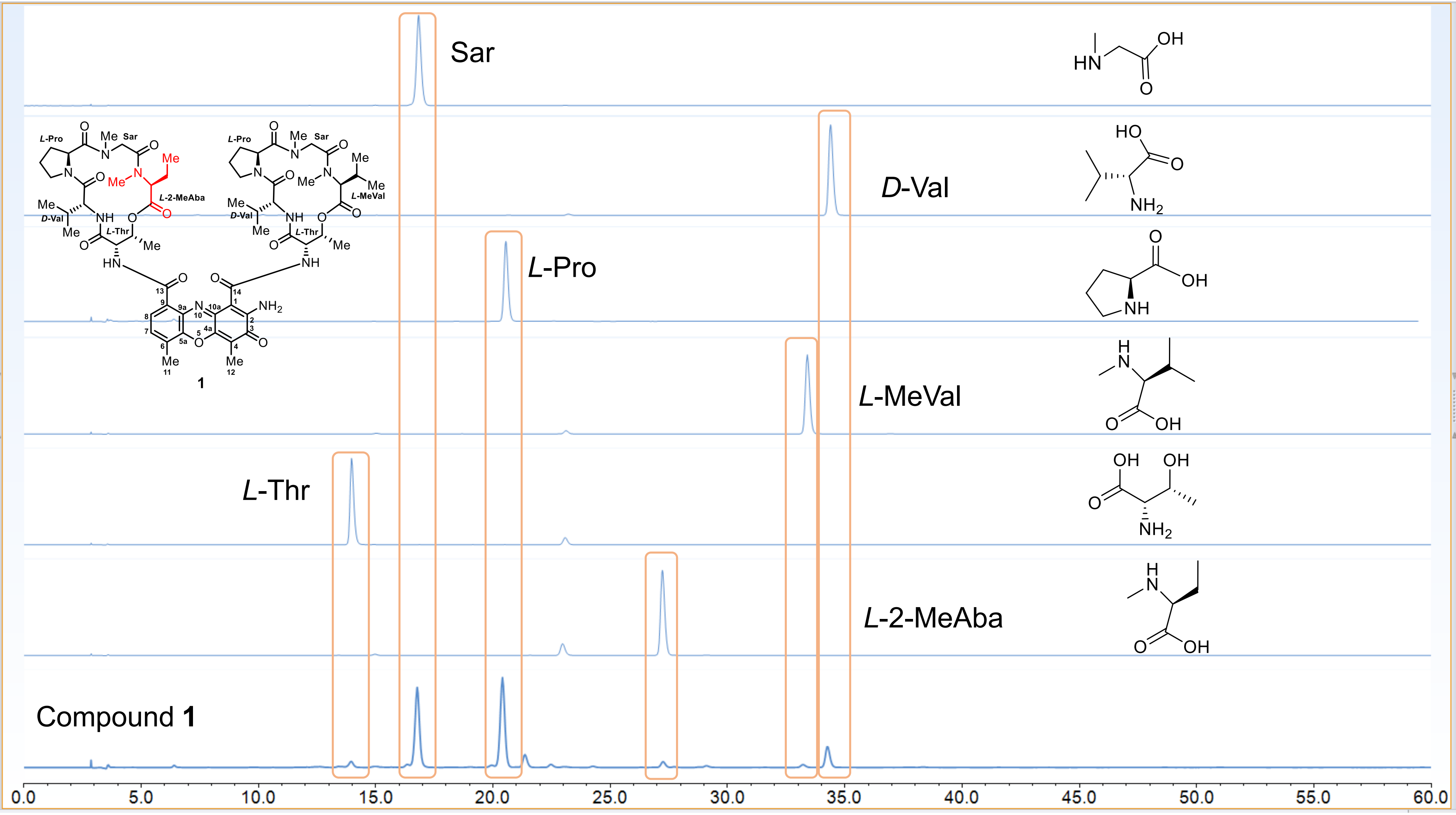

Figure S14. UV spectrum of compound **2**

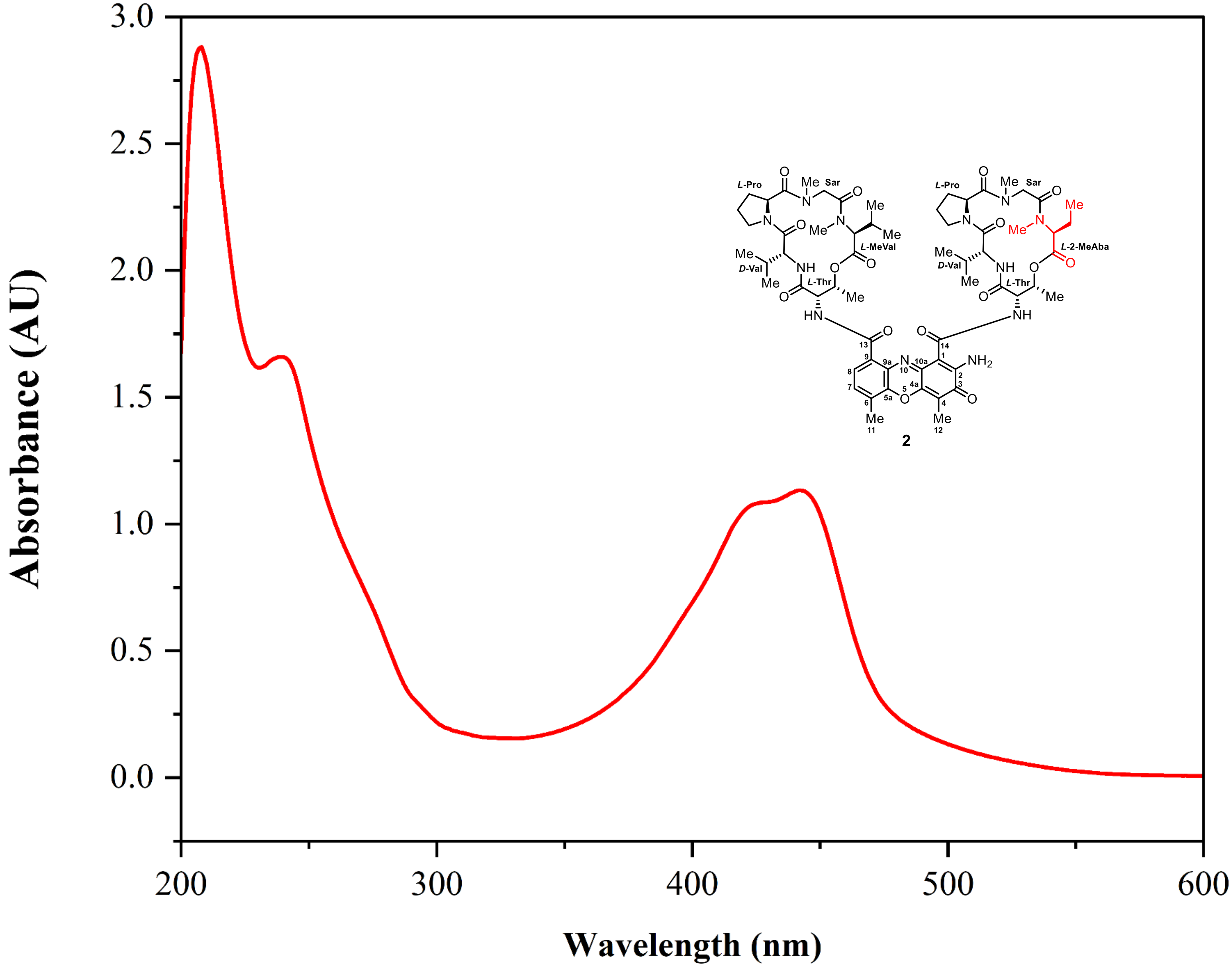

Figure S15. HR-ESIMS for compound **2**

Mass Spectrum SmartFormula Report

Analysis Info

Analysis Name	D:\Data\MS\data\202505\panghe_S-10-2_pos_32_01_19977.d	Acquisition Date	5/29/2025 5:36:43 PM
Method	LC_Direct Infusion_pos_100-1000mz.m	Operator	SCSIO
Sample Name	panghe_S-10-2_pos	Instrument	maXis 255552.00029
Comment			

Acquisition Parameter

Source Type	ESI	Ion Polarity	Positive	Set Nebulizer	0.4 Bar
Focus	Active	Set Capillary	4500 V	Set Dry Heater	180 °C
Scan Begin	100 m/z	Set End Plate Offset	-500 V	Set Dry Gas	4.0 l/min
Scan End	2000 m/z	Set Charging Voltage	0 V	Set Divert Valve	Waste
		Set Corona	0 nA	Set APCI Heater	0 °C

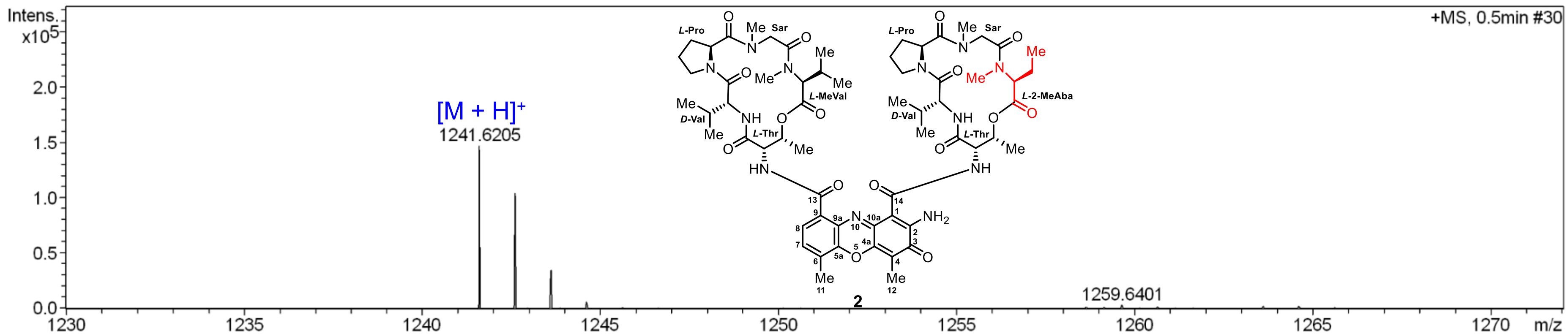

Meas. m/z	#	Ion Formula	Score	m/z	err [ppm]	err [mDa]	mSigma	rdb	e⁻ Conf	N-Rule
1241.6205	1	C61H85N12O16	100.00	1241.6201	-0.3	-0.4	28.5	25.5	even	ok
1258.6439	1	C61H88N13O16	100.00	1258.6467	-2.2	-2.7	289.8	24.5	even	ok
1263.6026	1	C61H84N12NaO16	100.00	1263.6020	-0.4	-0.6	127.6	25.5	even	ok

Figure S16. ^{1}H NMR (600 MHz) spectrum of compound **2** in $CDCl_3$

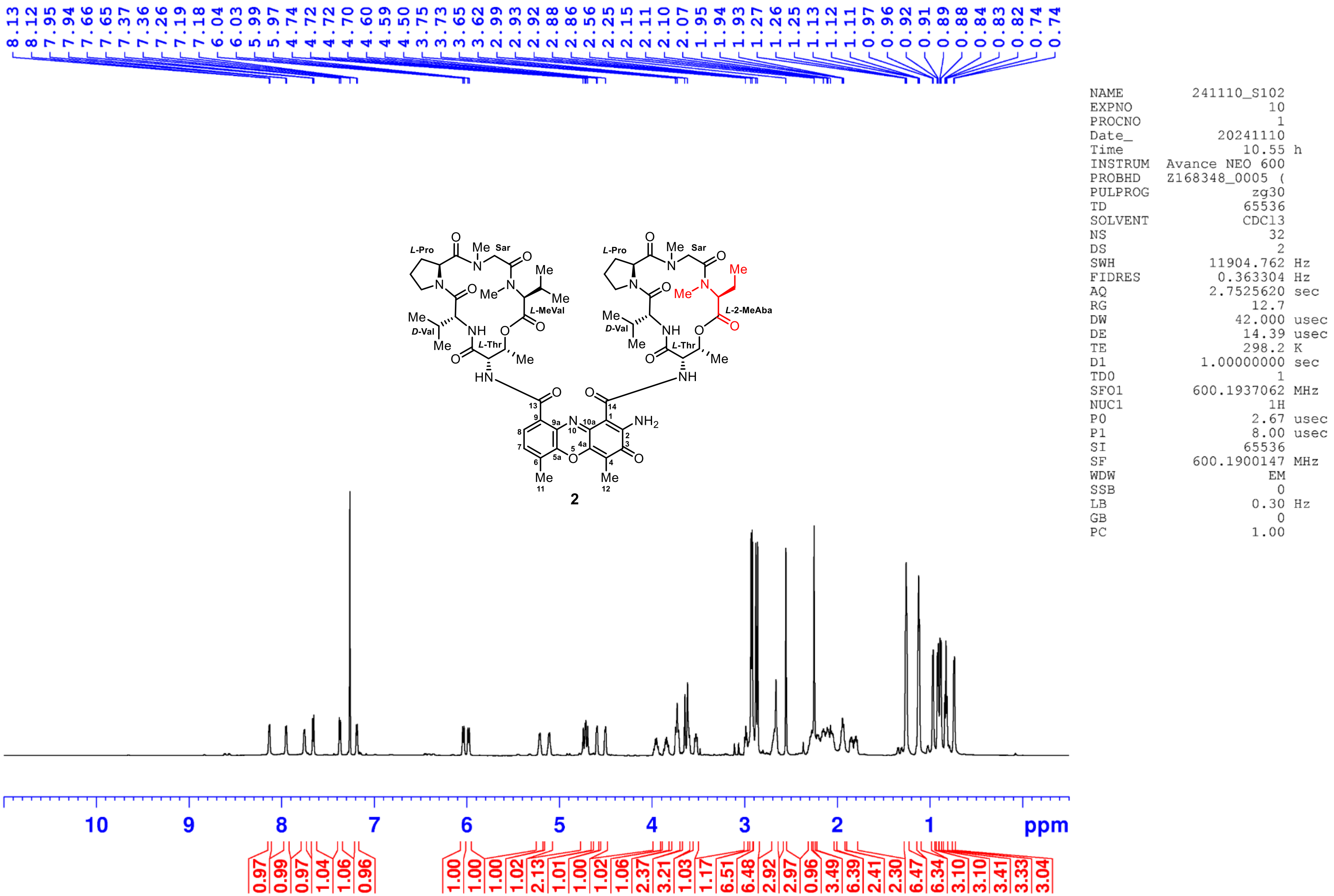

Figure S16-continue

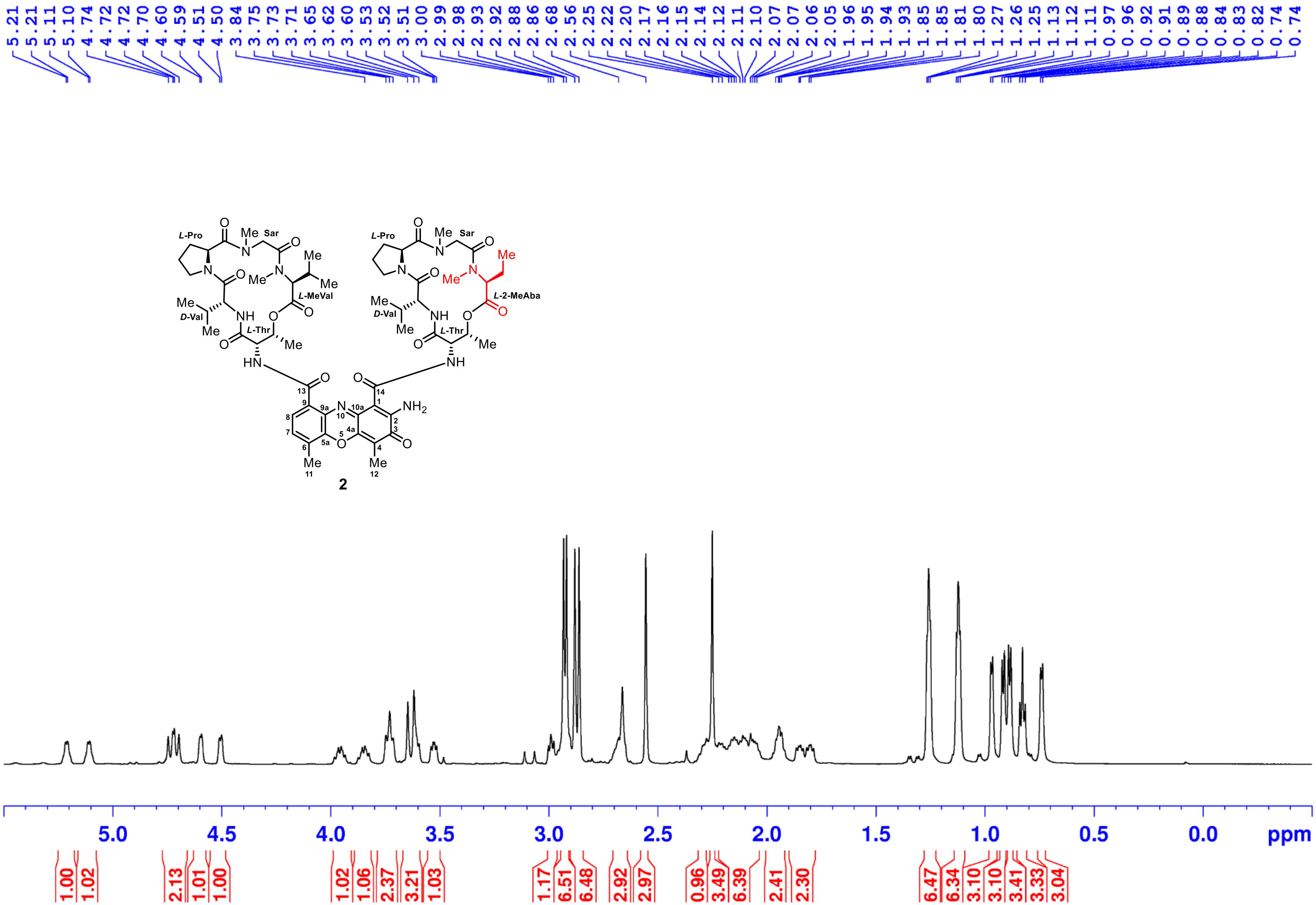

Figure S16-continue

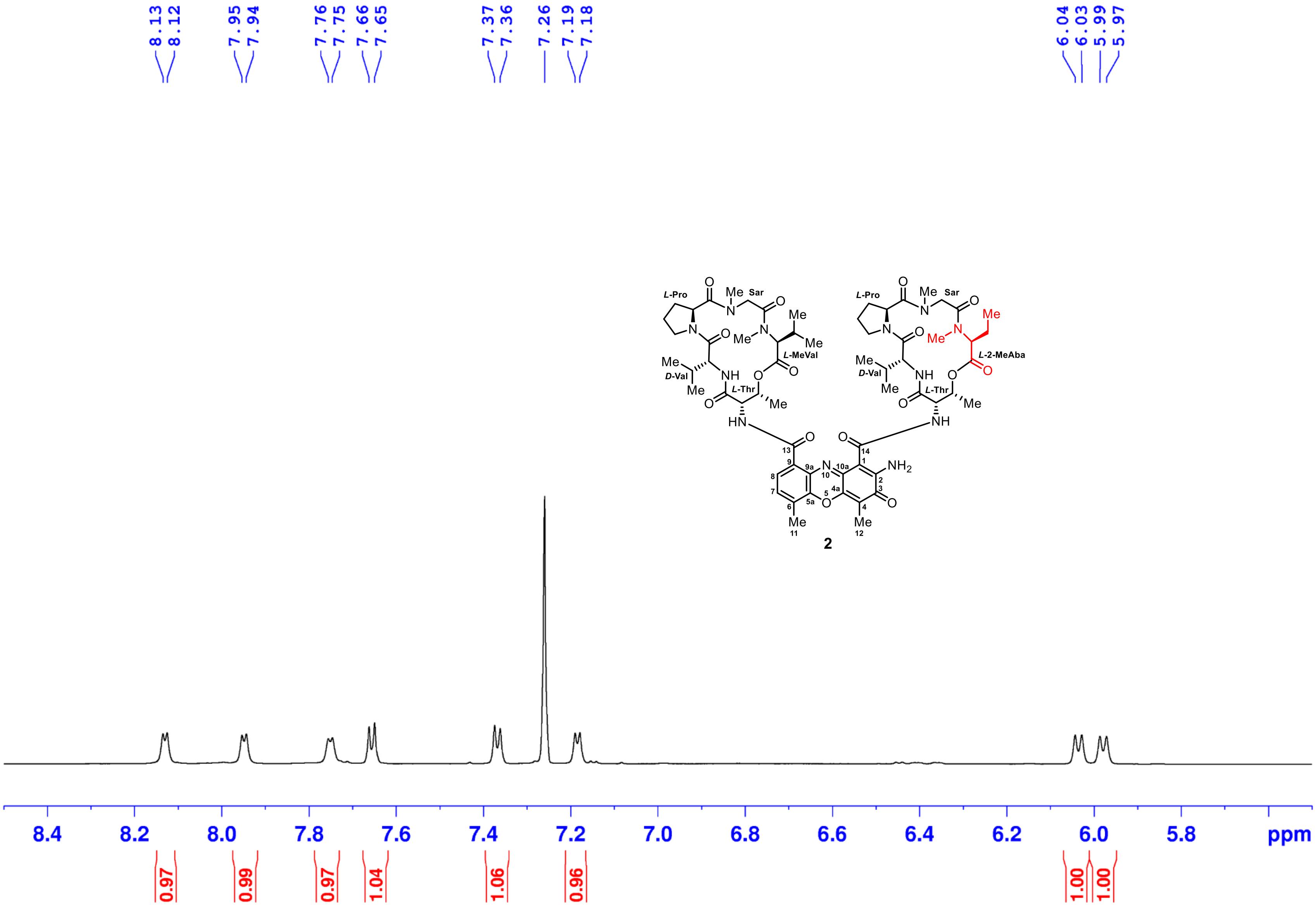

Figure S17. ^{13}C NMR (150 MHz) spectrum of compound **2** in $CDCl_3$

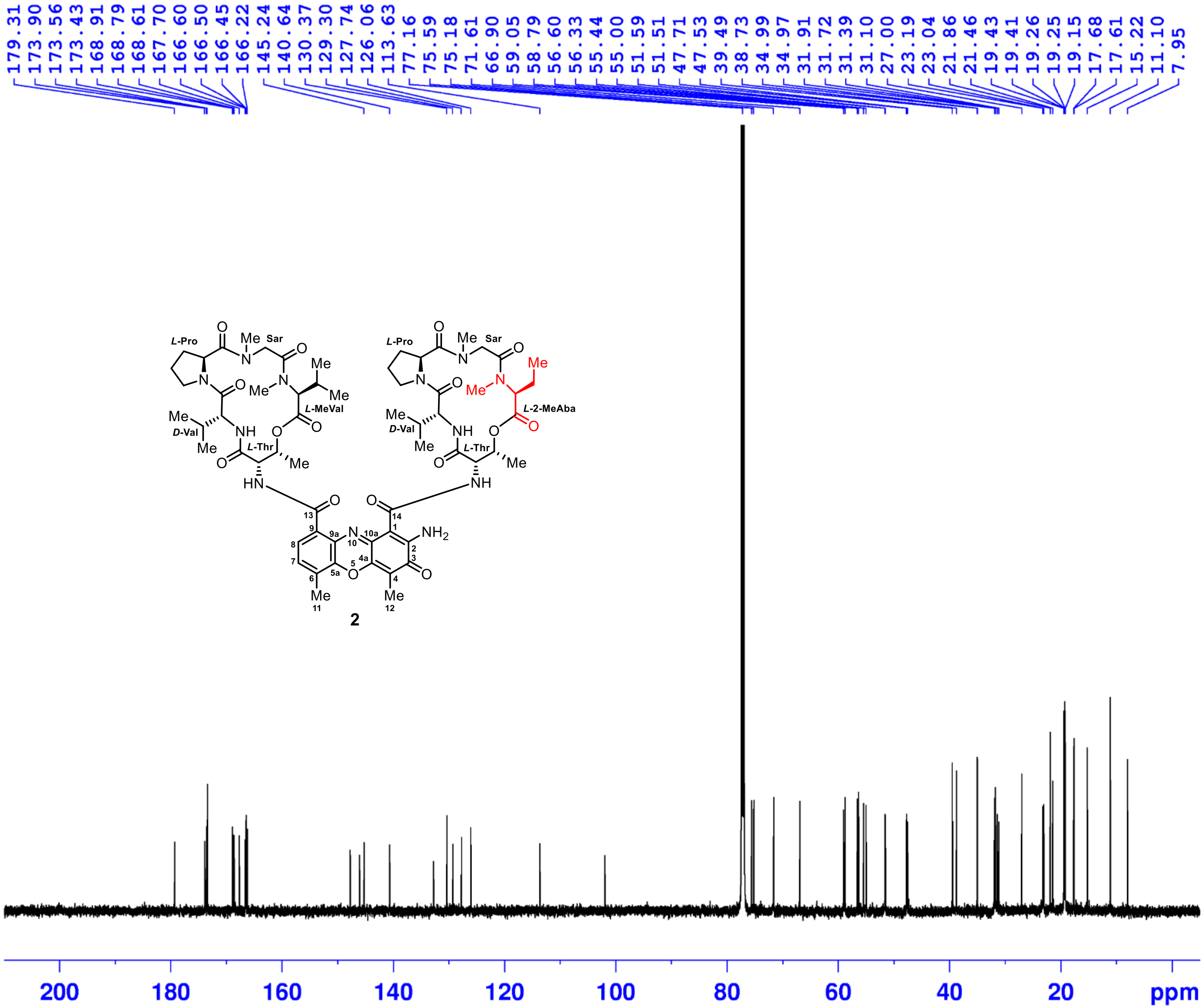

NAME	241110_S102C
EXPNO	30
PROCNO	1
Date_	20241112
Time	4.13 h
INSTRUM	Avance NEO 600
PROBHD	Z168348_0005 (
PULPROG	zgpg30
TD	35714
SOLVENT	CDCl3
NS	2048
DS	4
SWH	35714.285 Hz
FIDRES	2.000016 Hz
AQ	0.5000460 sec
RG	101
DW	14.000 usec
DE	18.00 usec
TE	298.2 K
D1	1.00000000 sec
D11	0.03000000 sec
TD0	1
SFO1	150.9329873 MHz
NUC1	13C
P0	4.00 usec
P1	12.00 usec
SI	32768
SF	150.9178748 MHz
WDW	EM
SSB	0
LB	1.00 Hz
GB	0
PC	1.40

Figure S17-continue

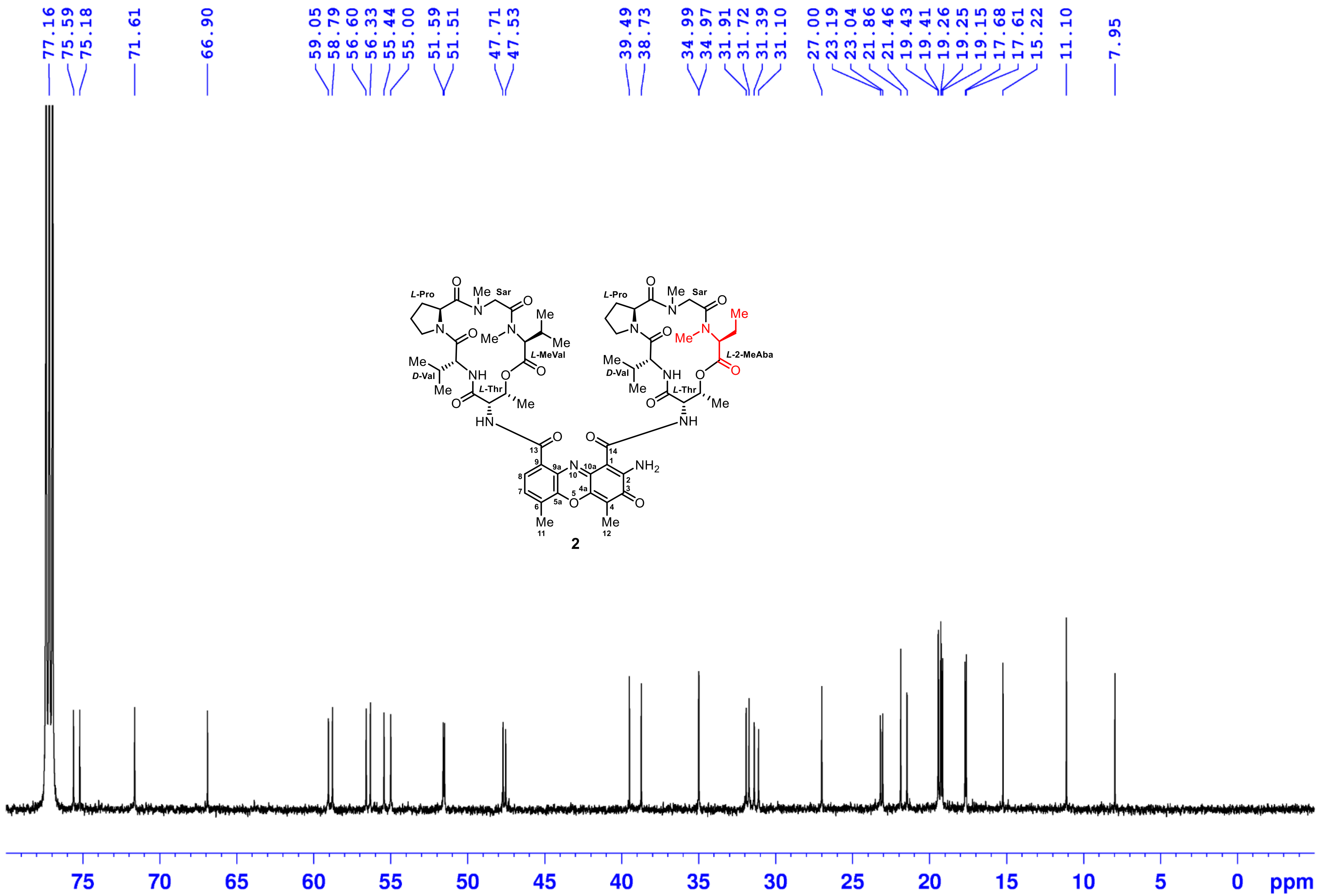

Figure S17-continue

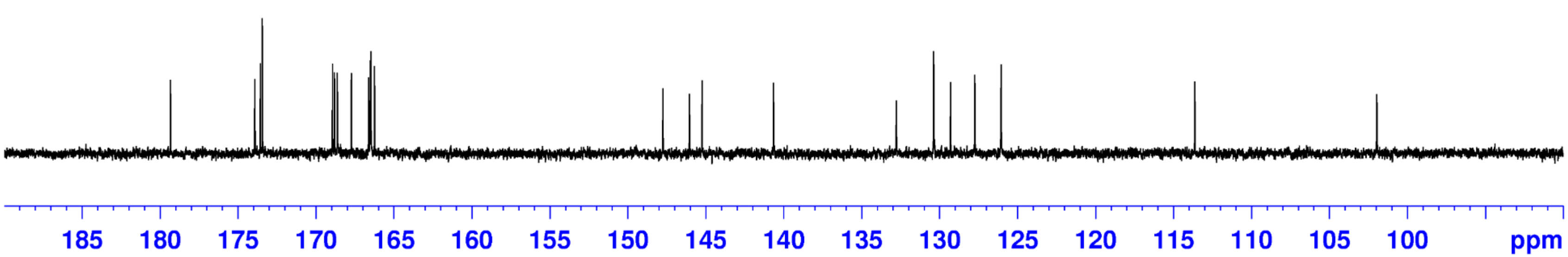

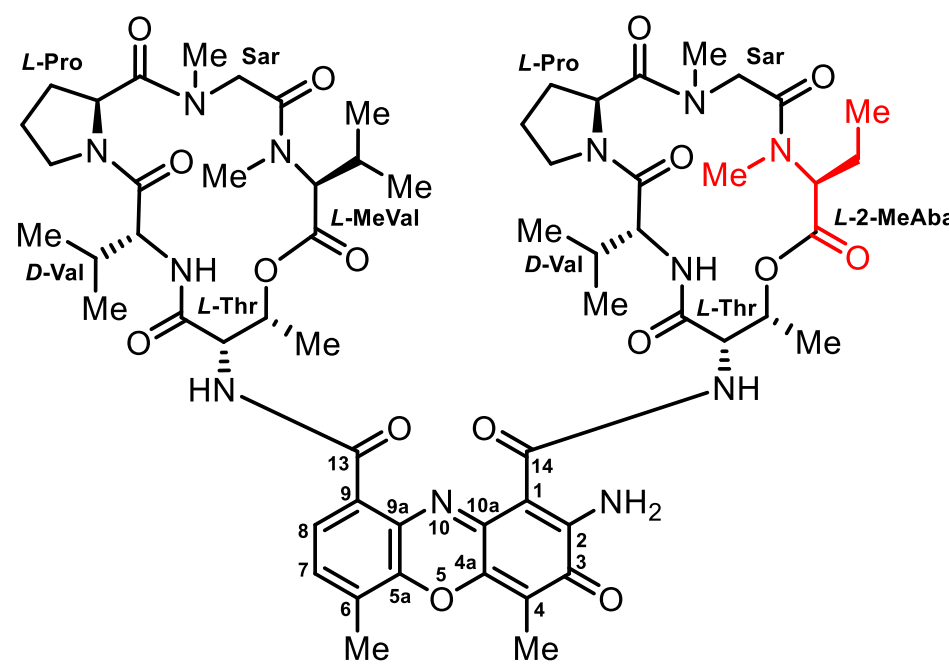

2

Figure S18. DEPT135 (150 MHz) spectrum of compound **2** in $CDCl_3$

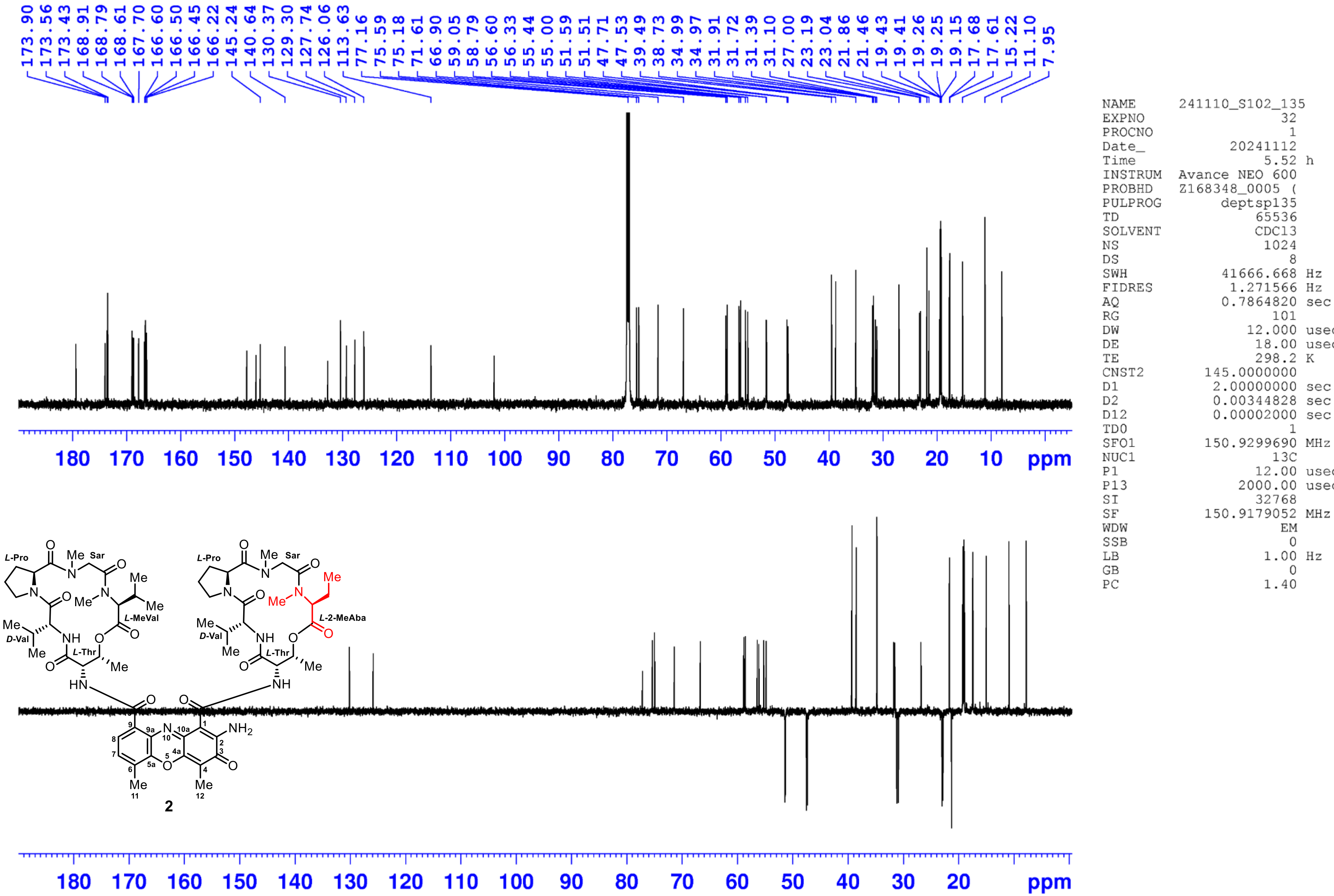

Figure S18-continue

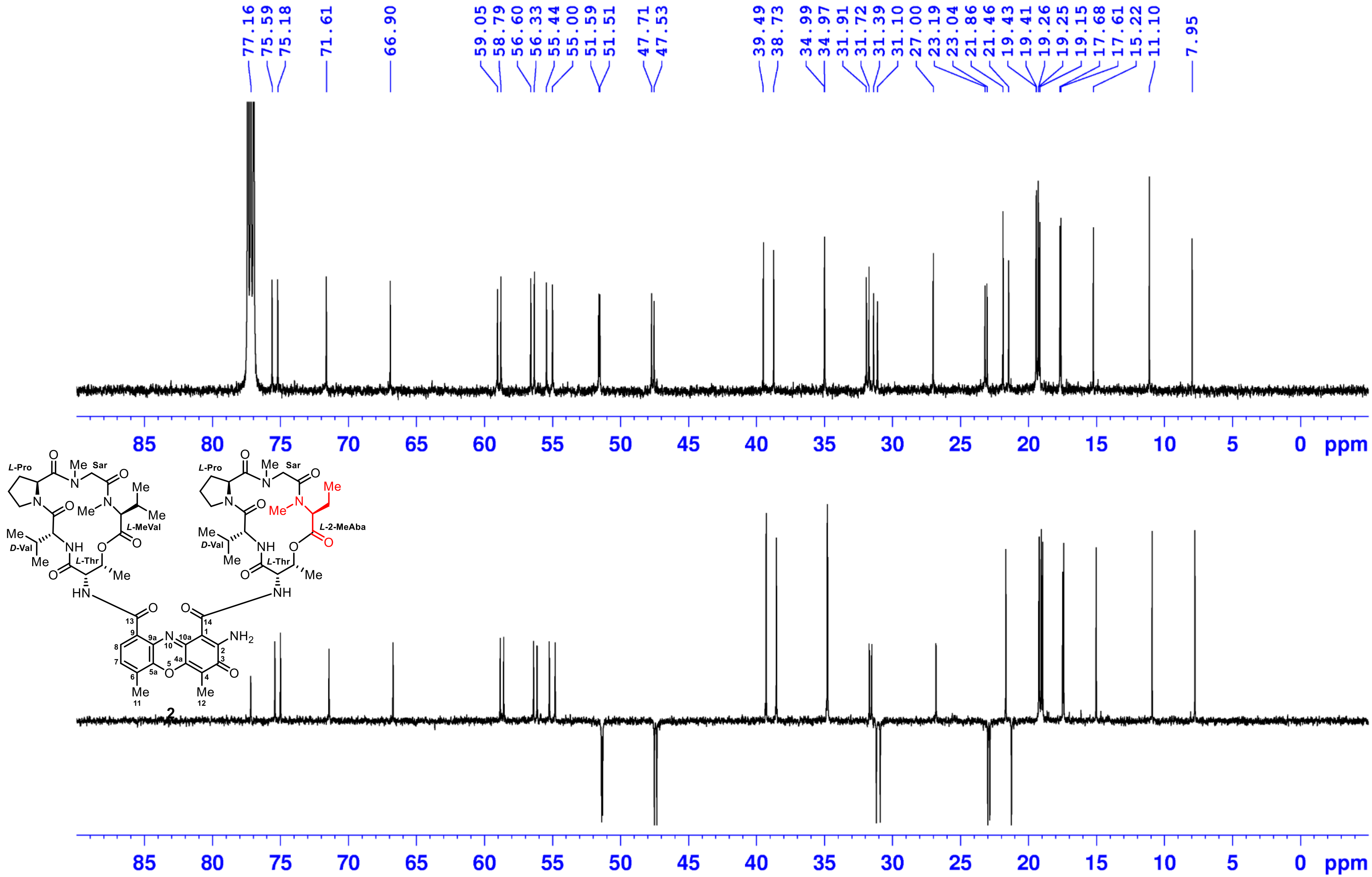

Figure S18-continue

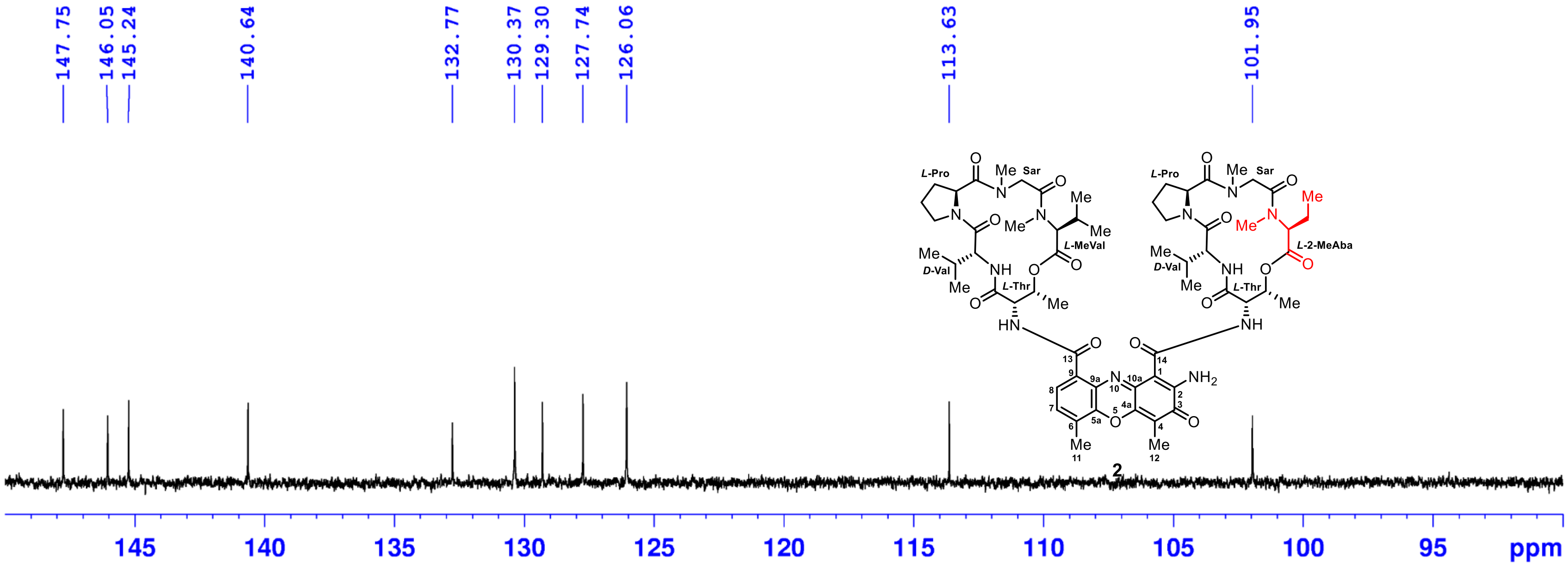

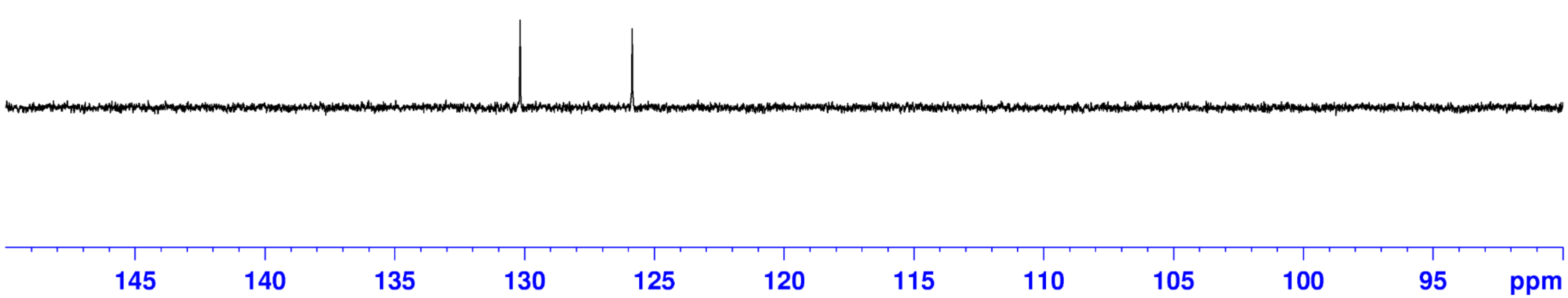

Figure S19. ^{1}H–^{1}H COSY (600 MHz) spectrum of compound **2** in $CDCl_3$

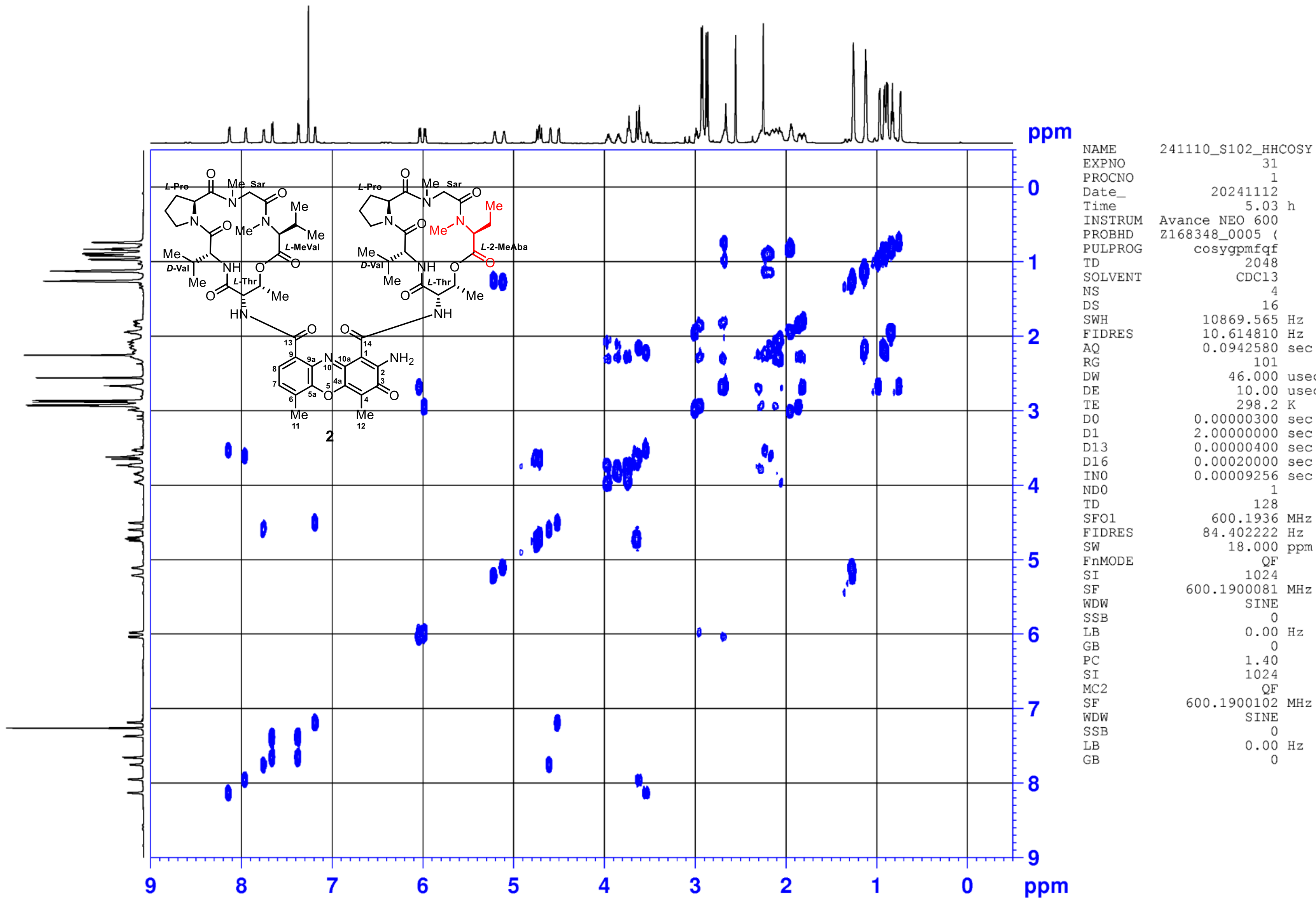

Figure S19-continue

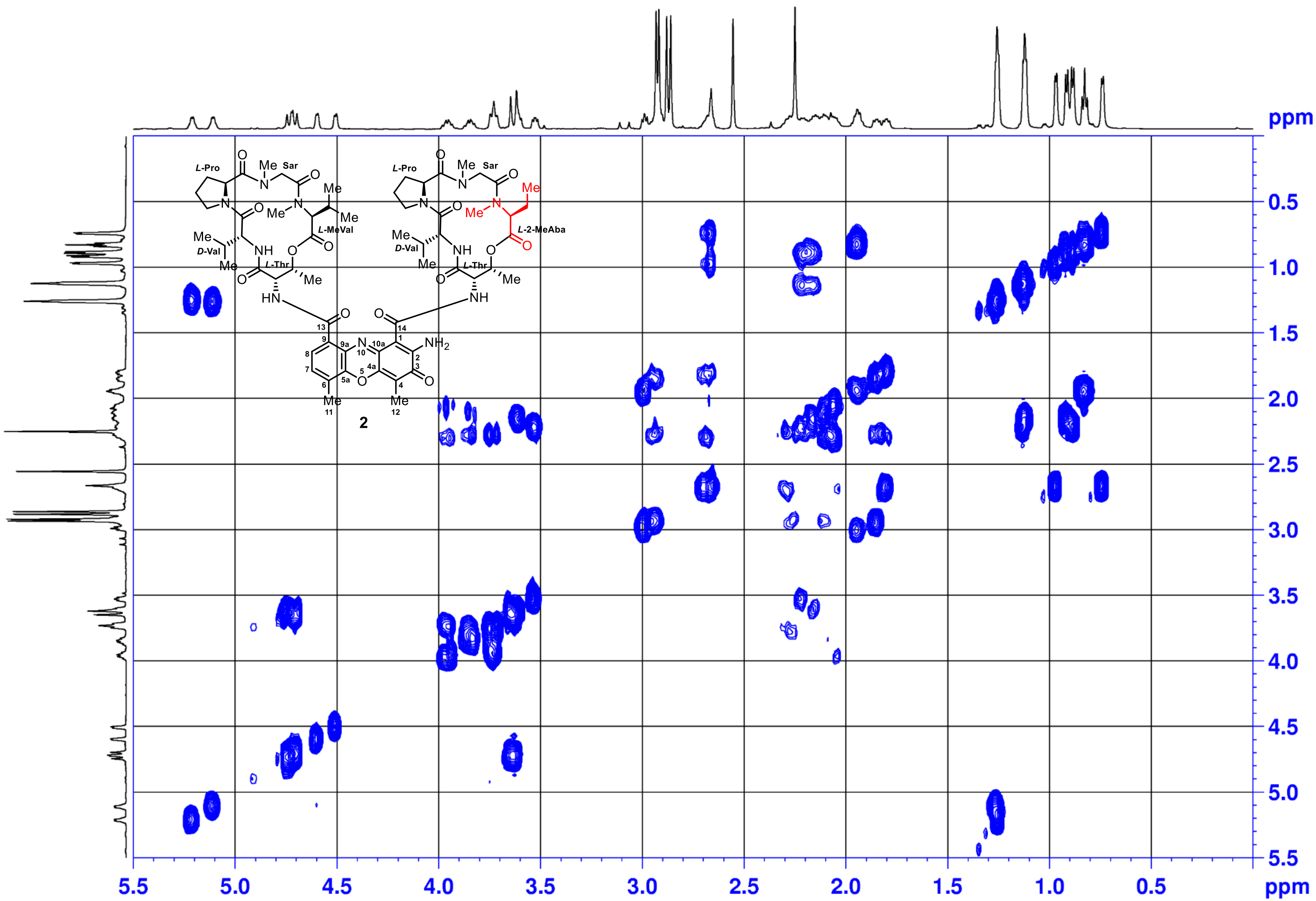

Figure S19-continue

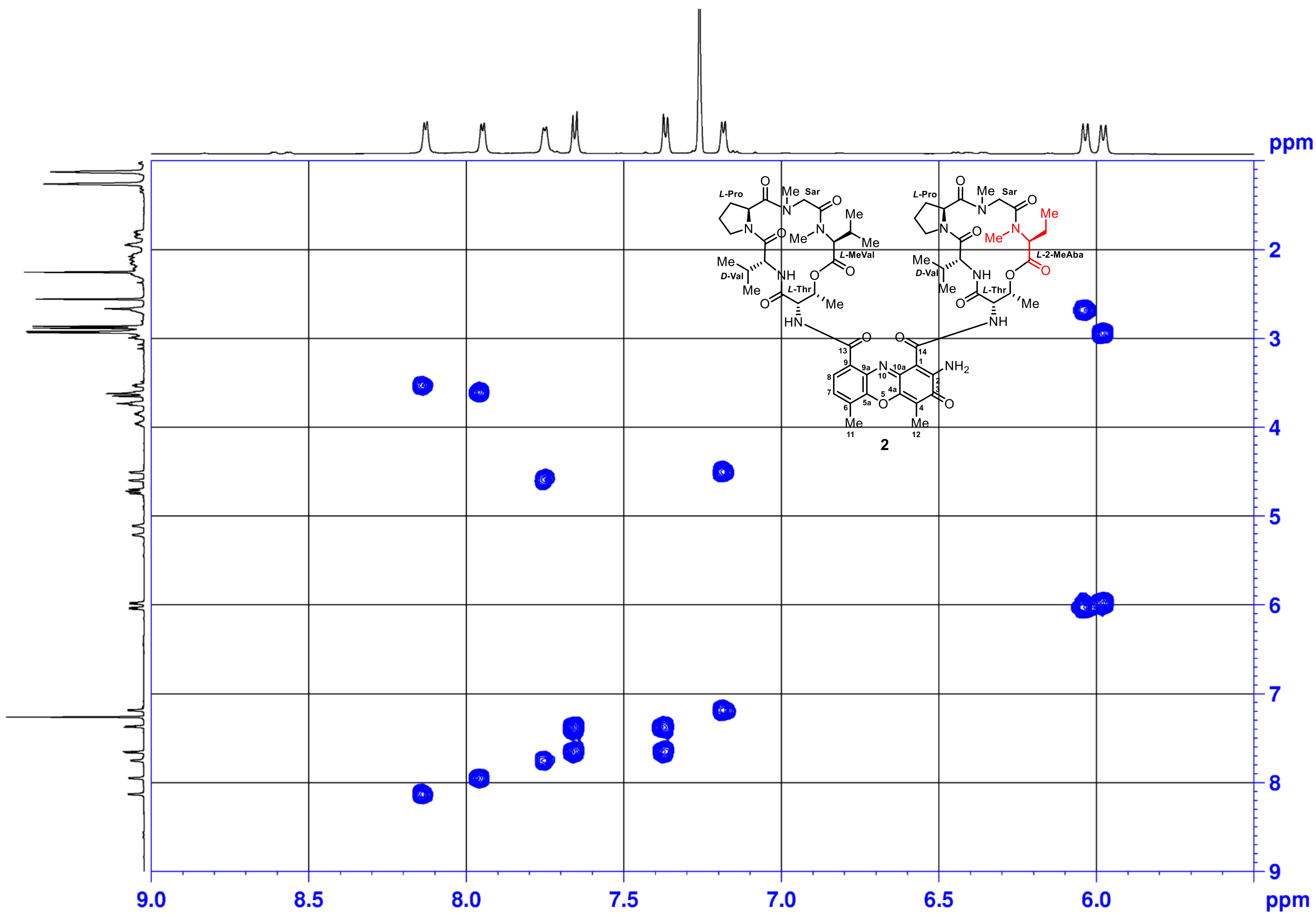

Figure S20. HSQC (600 MHz) spectrum of compound **2** in $CDCl_3$

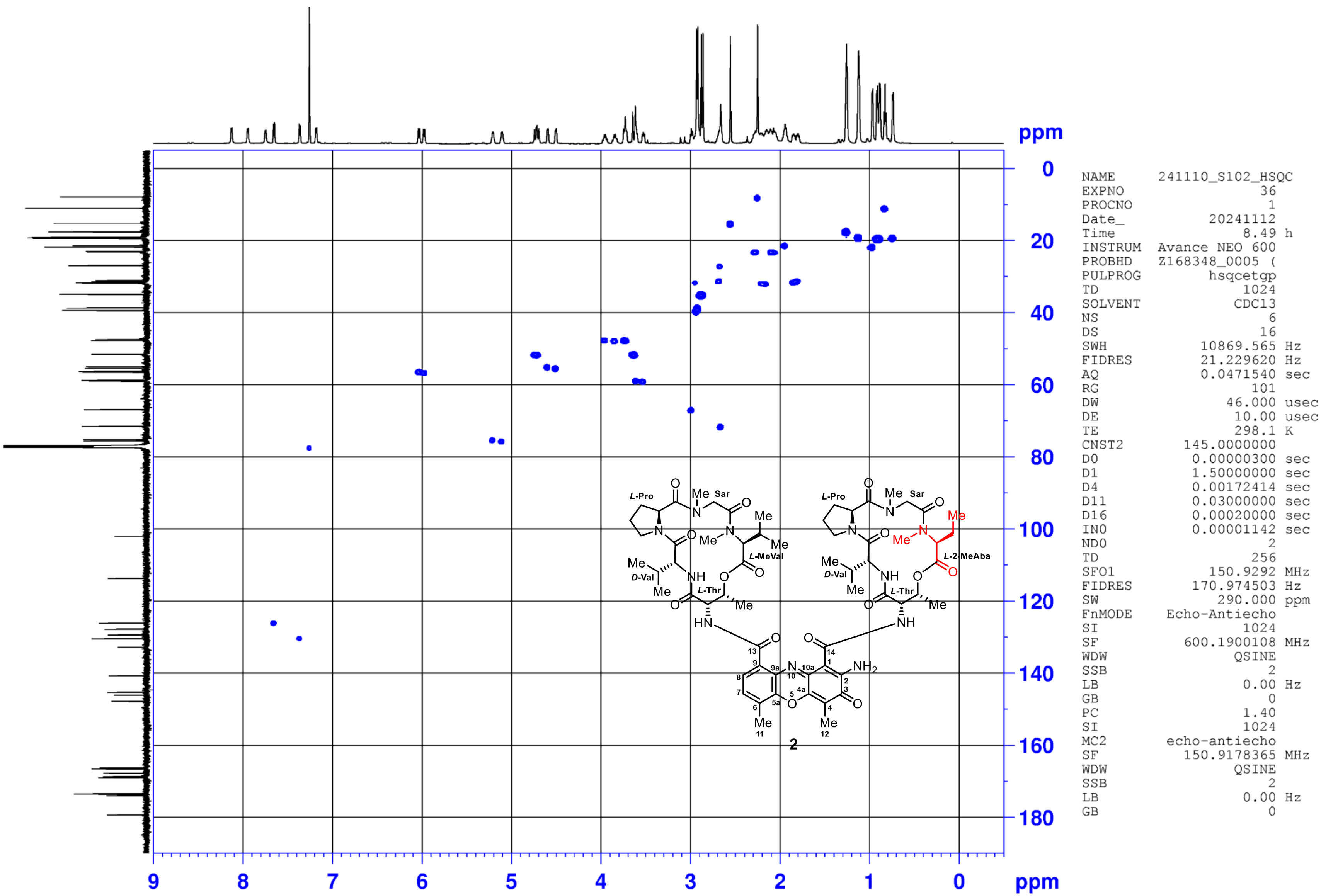

Figure S20-continue

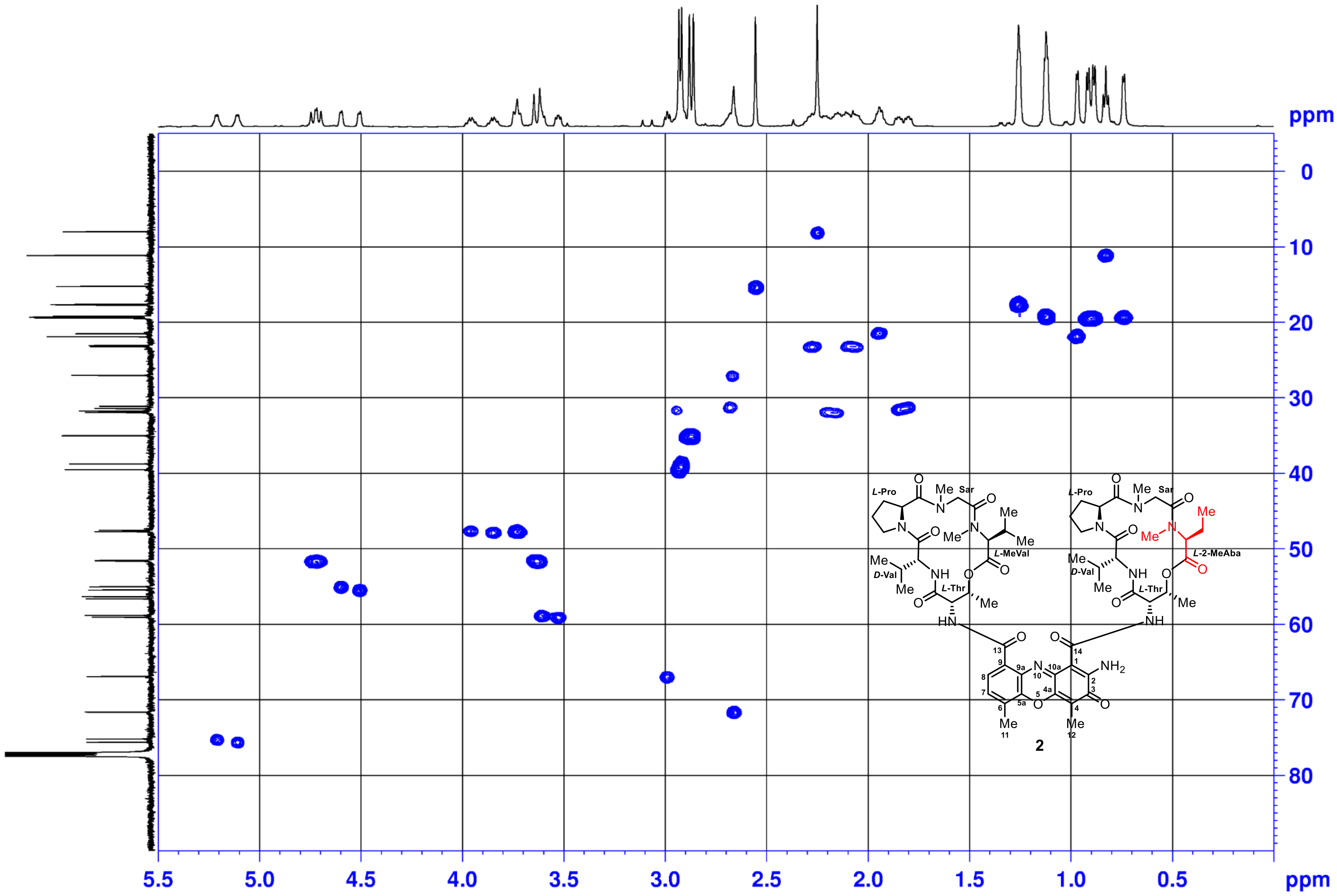

Figure S20-continue

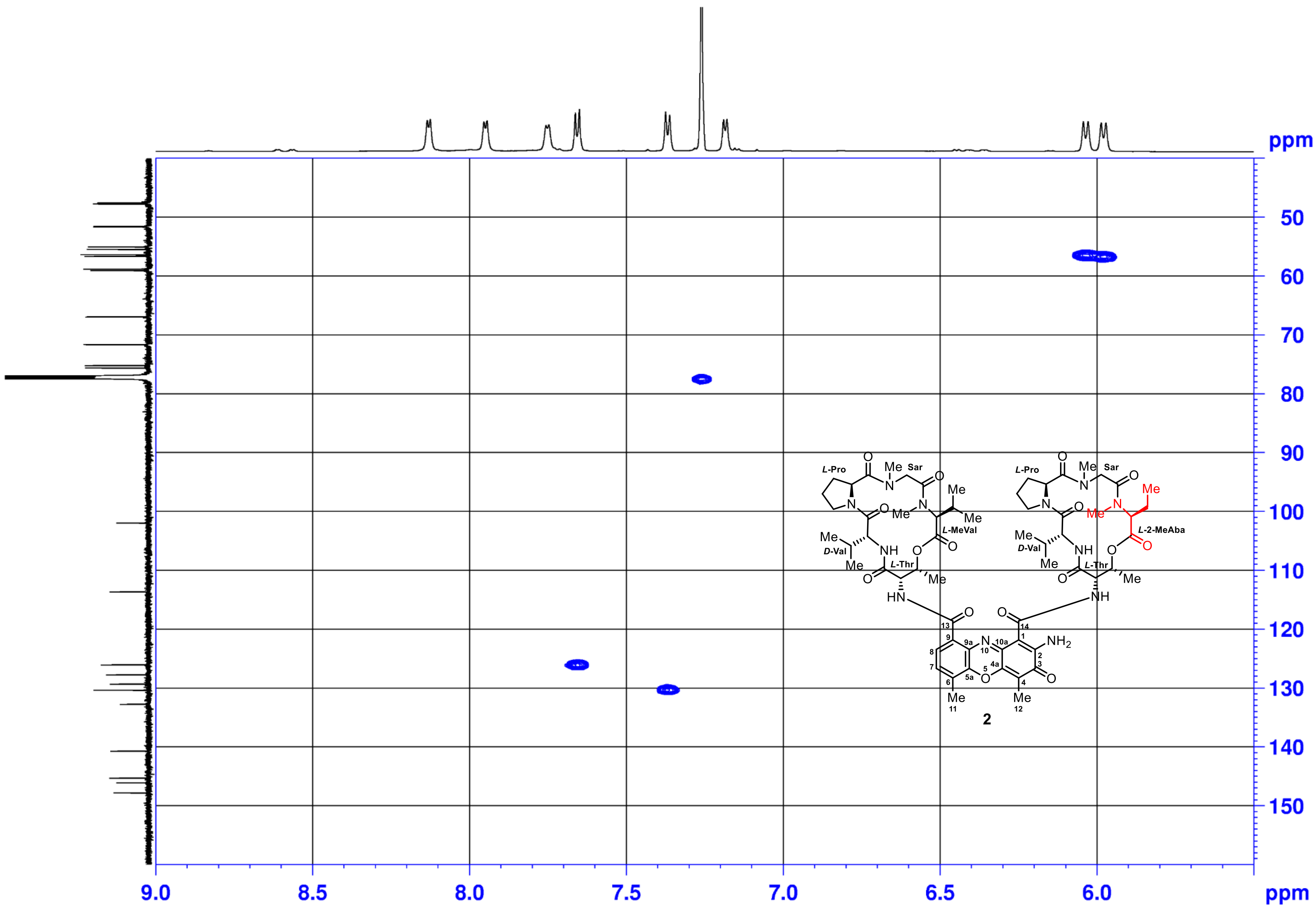

Figure S21. HMBC (600 MHz) spectrum of compound **2** in $CDCl_3$

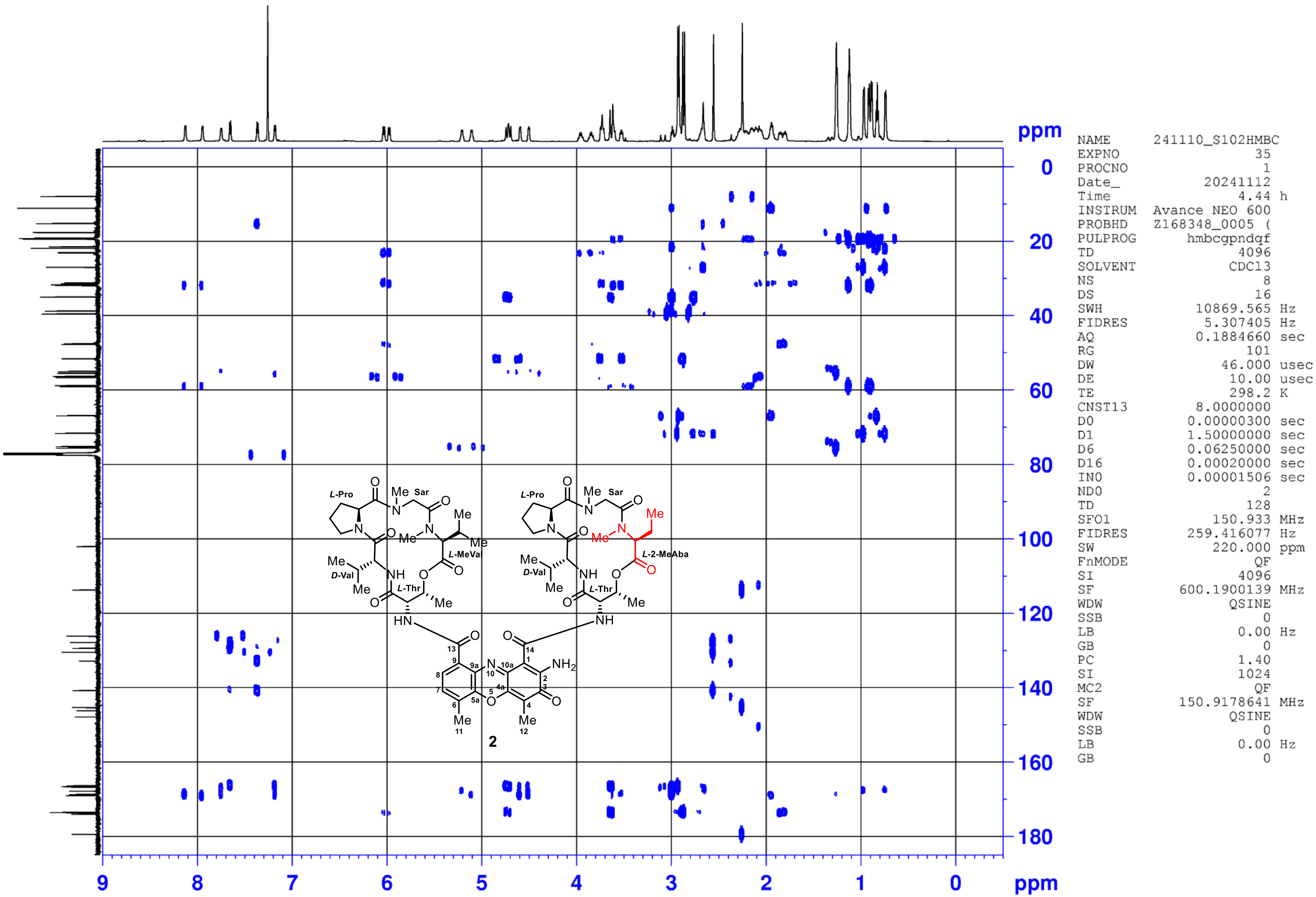

Figure S21-continue

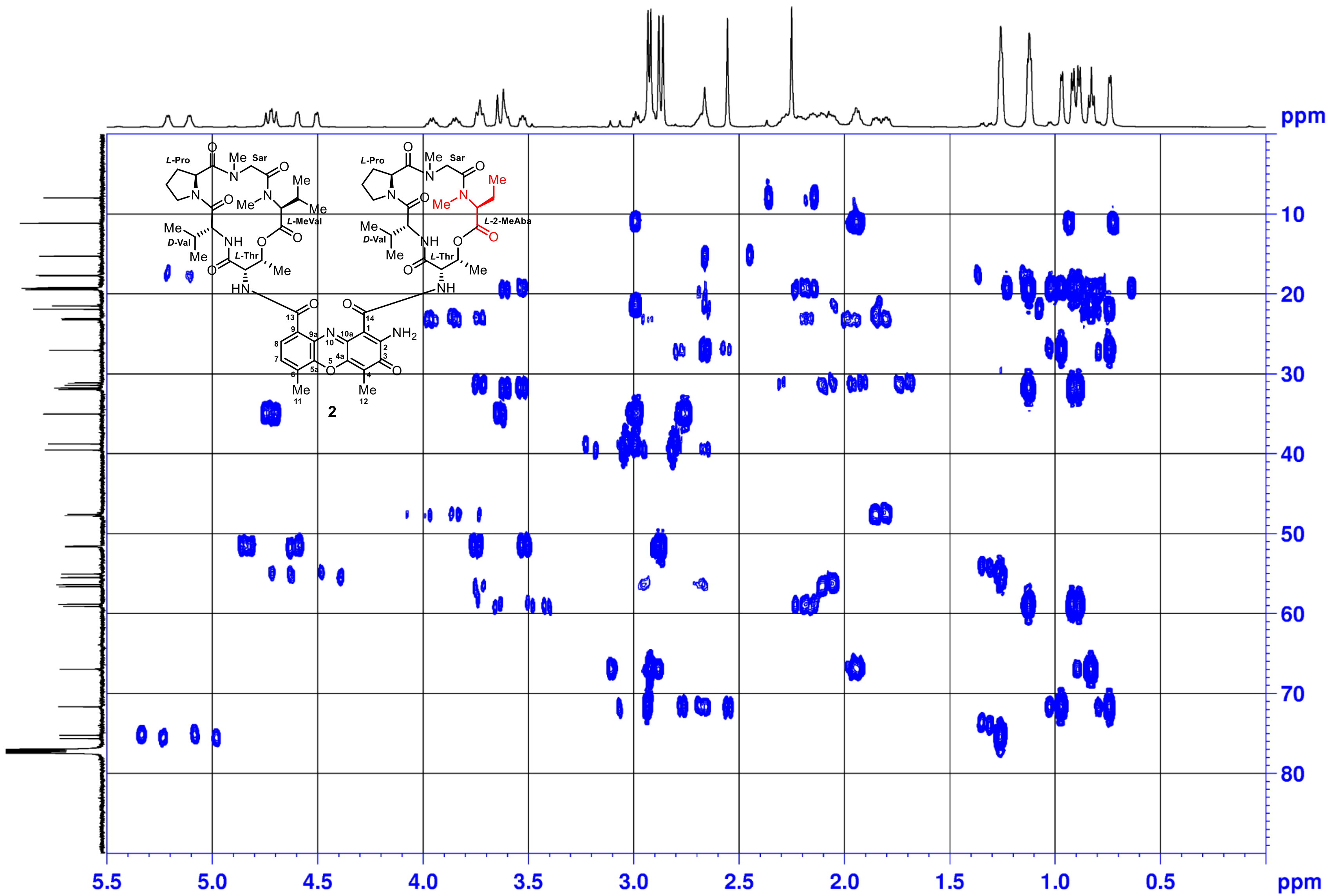

Figure S21-continue

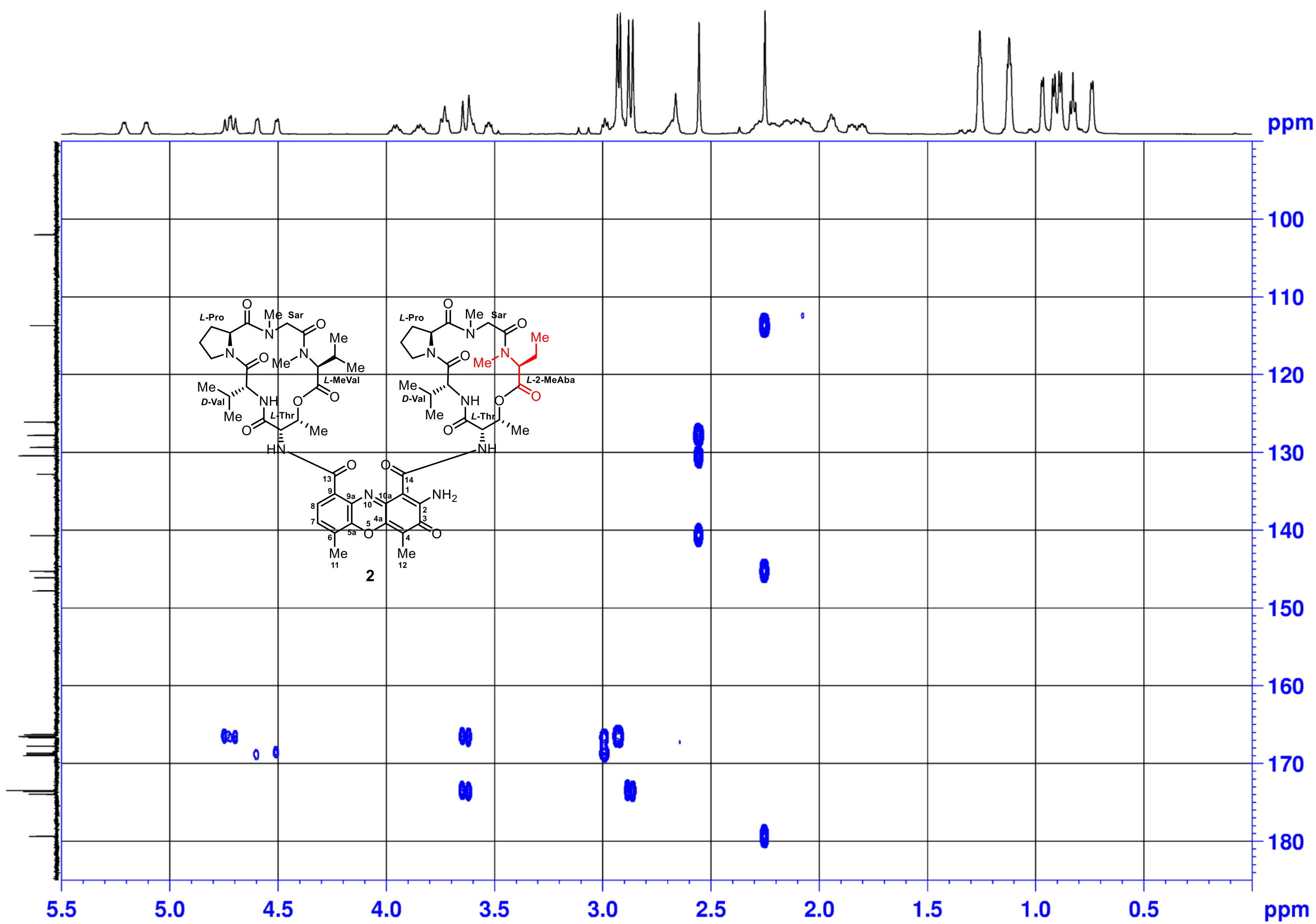

Figure S21-continue

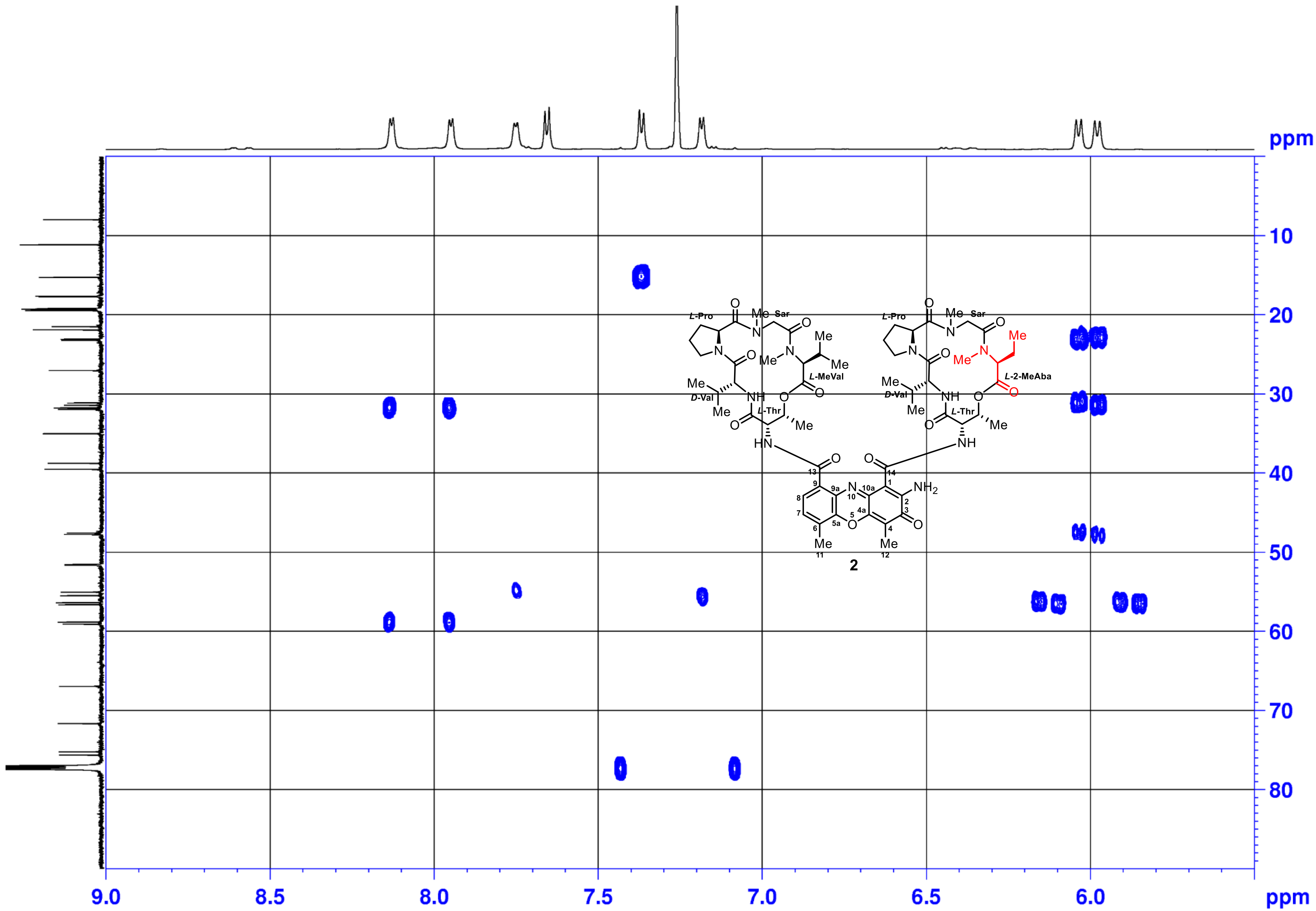

Figure S21-continue

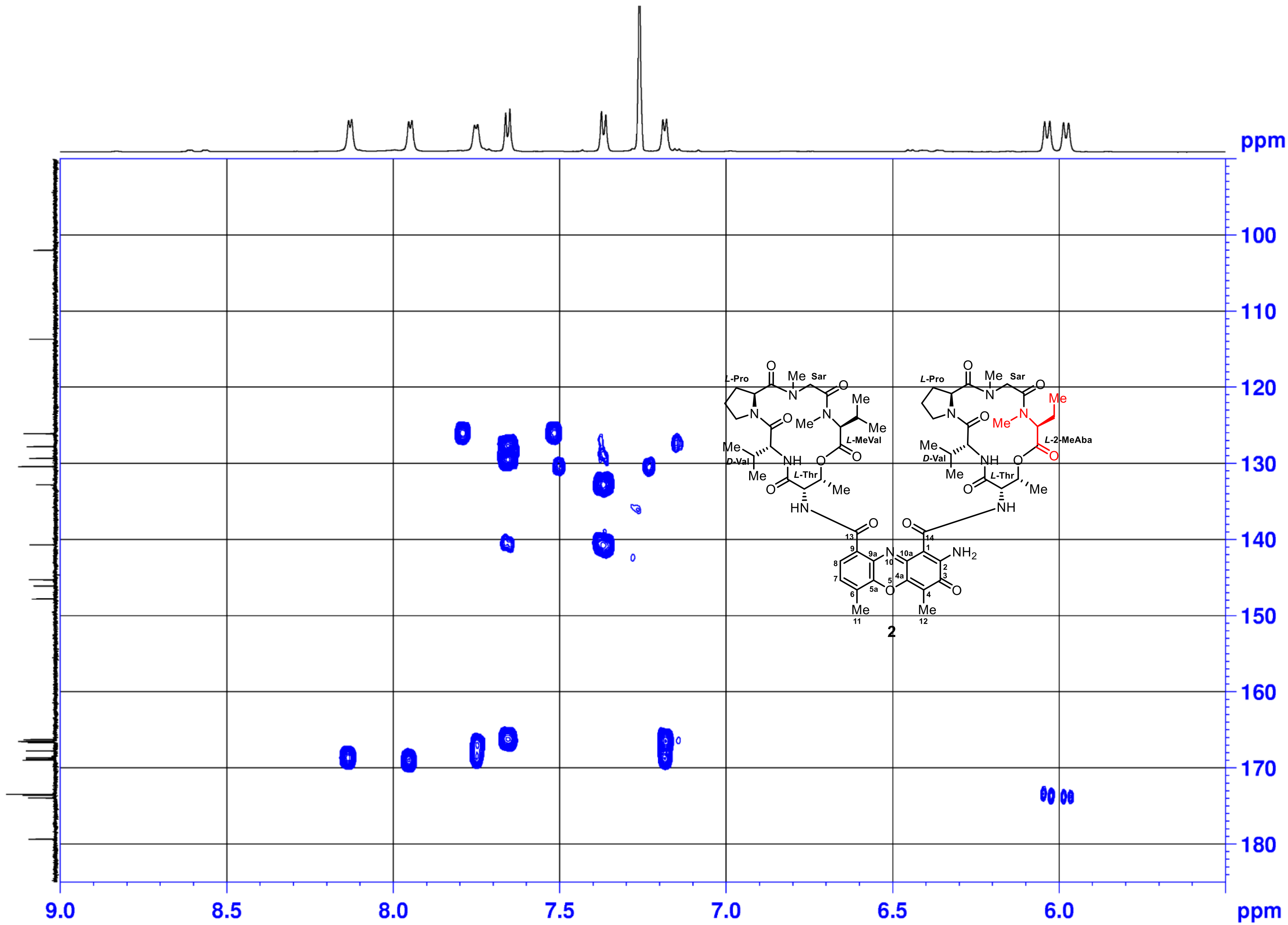

Figure S22. NOESY (600 MHz) spectrum of compound **2** in $CDCl_3$

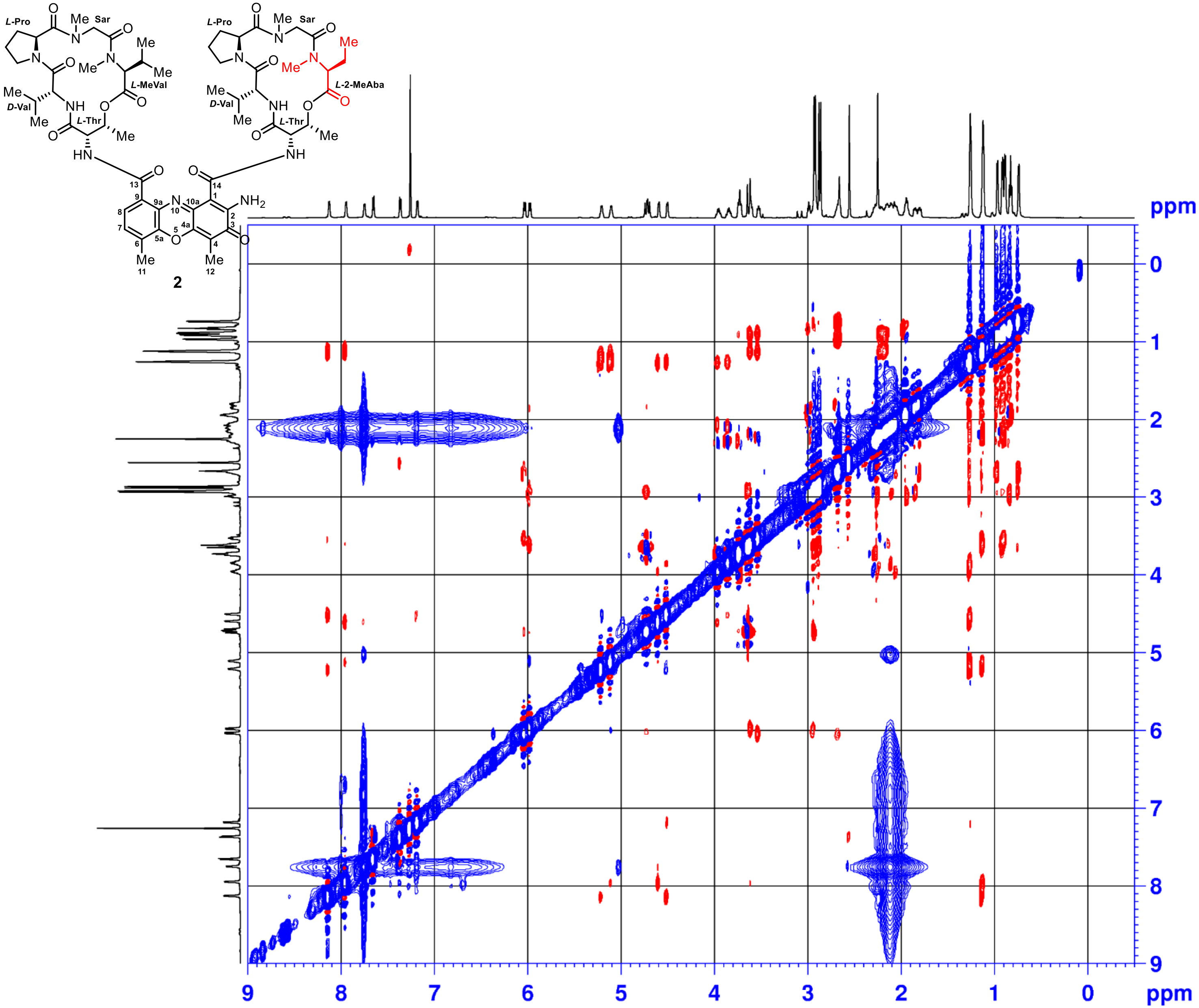

NAME	241110_S102_NOE
EXPNO	34
PROCNO	1
Date_	20241112
Time	8.08 h
INSTRUM	Avance NEO 600
PROBHD	Z168348_0005 (
PULPROG	noesygpphpp
TD	2048
SOLVENT	CDCl3
NS	8
DS	32
SWH	11904.762 Hz
FIDRES	11.625744 Hz
AQ	0.0860660 sec
RG	64
DW	42.000 usec
DE	10.66 usec
TE	298.2 K
D0	0.00003147 sec
D1	2.00000000 sec
D8	0.30000001 sec
D11	0.03000000 sec
D12	0.00002000 sec
D16	0.00020000 sec
IN0	0.00008331 sec
ND0	1
TD	256
SFO1	600.1928 MHz
FIDRES	46.890064 Hz
SW	20.000 ppm
FnMODE	States-TPPI
SI	1024
SF	600.1900062 MHz
WDW	QSINE
SSB	2
LB	0.00 Hz
GB	0
PC	1.00
SI	1024
MC2	States-TPPI
SF	600.1900084 MHz
WDW	QSINE
SSB	2
LB	0.00 Hz
GB	0

Figure S22-continue

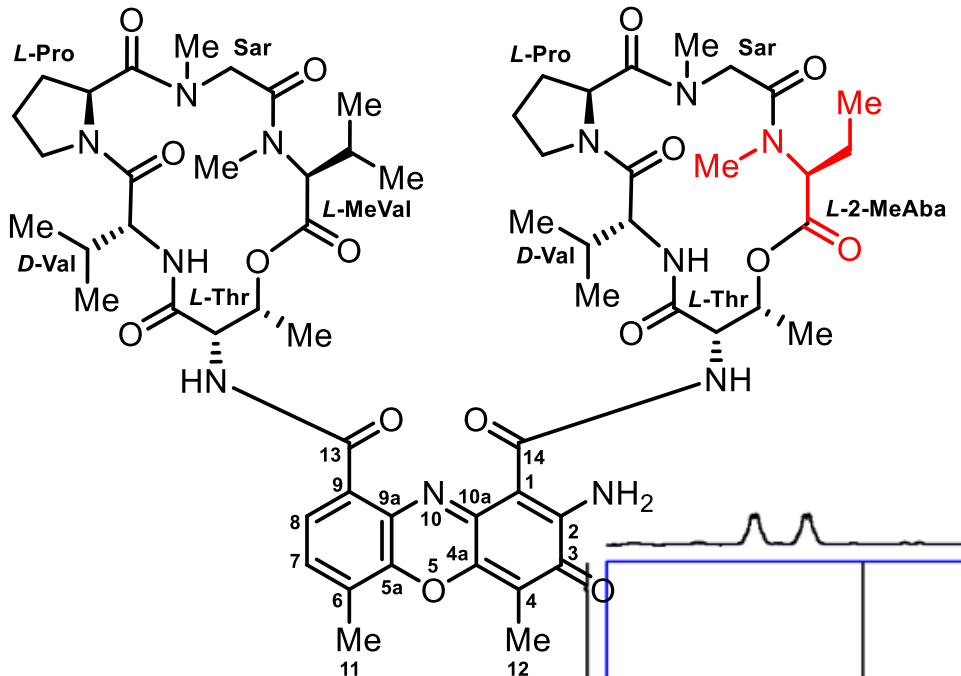

2

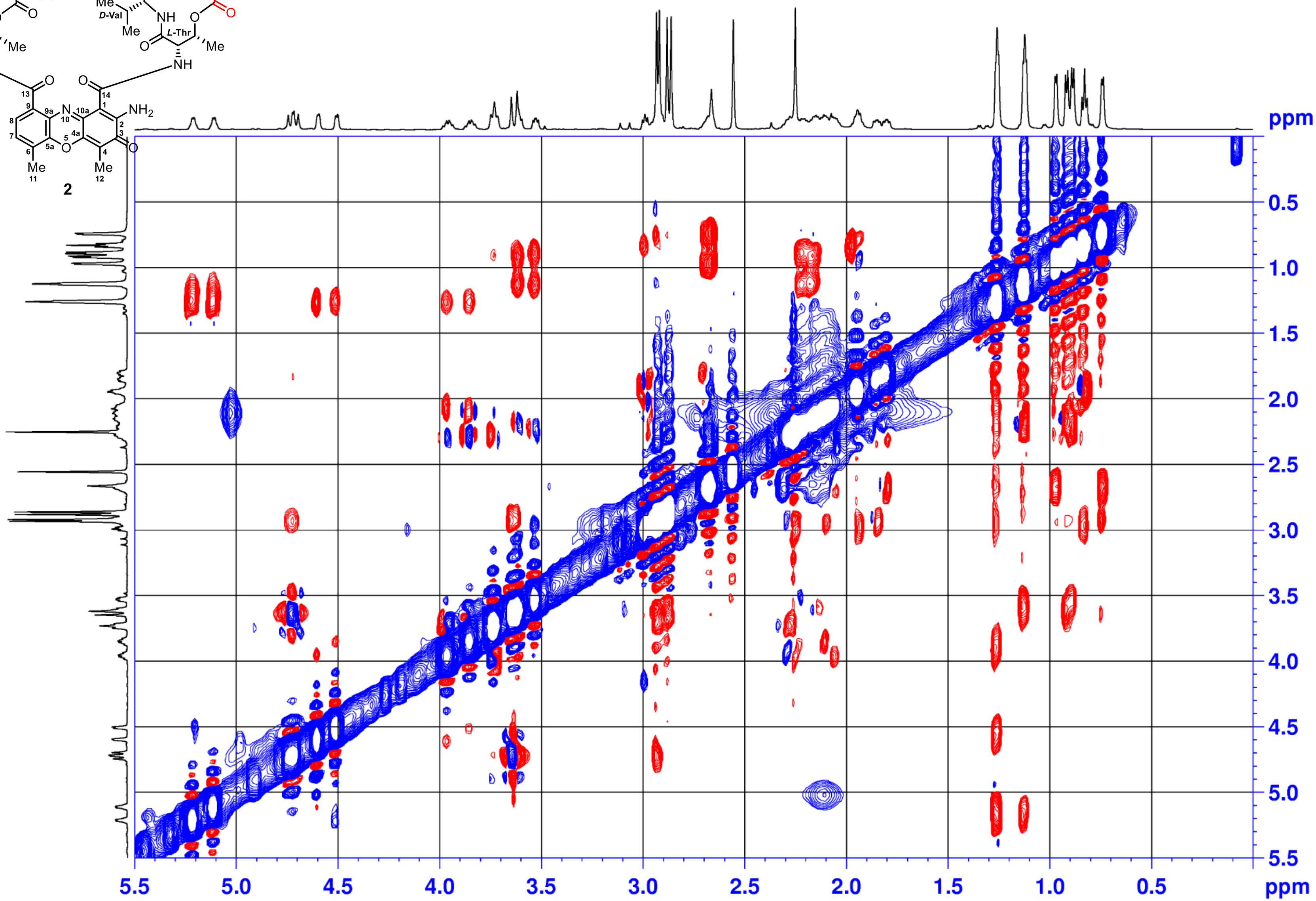

Figure S22-continue

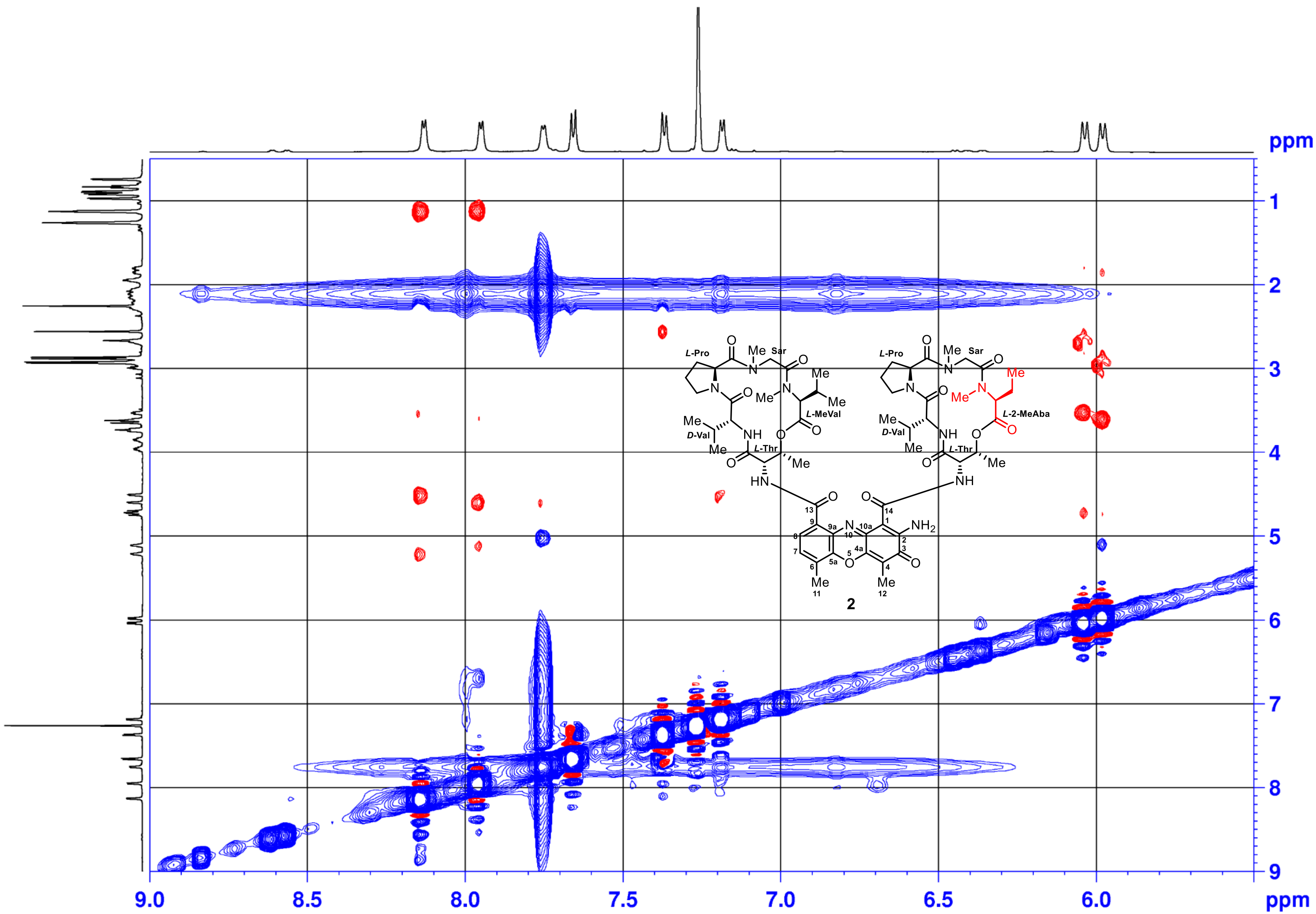

Figure S23. Marfey's analysis of compound **2** and standard amino acids

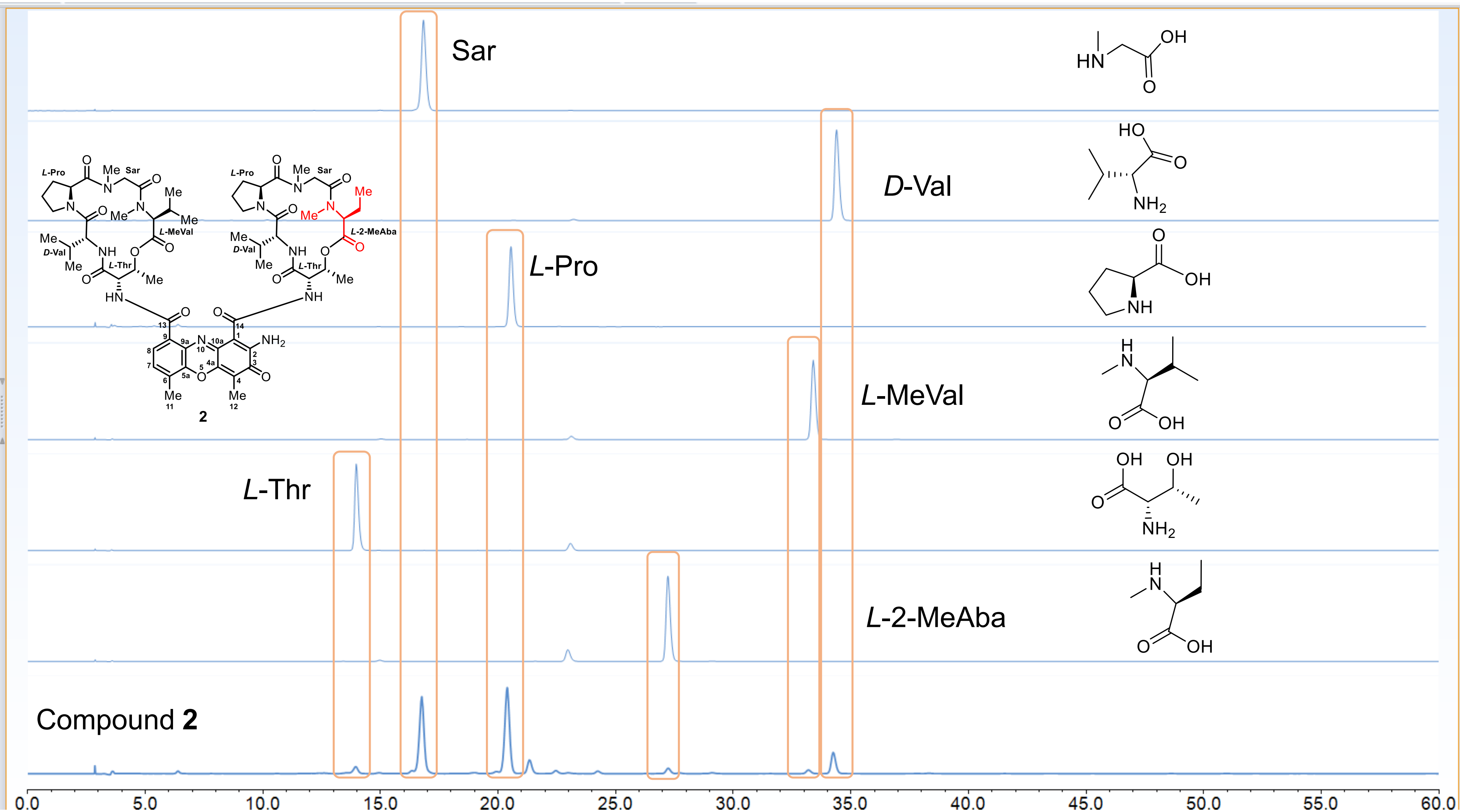

Figure S24. UV spectrum of compound **3**

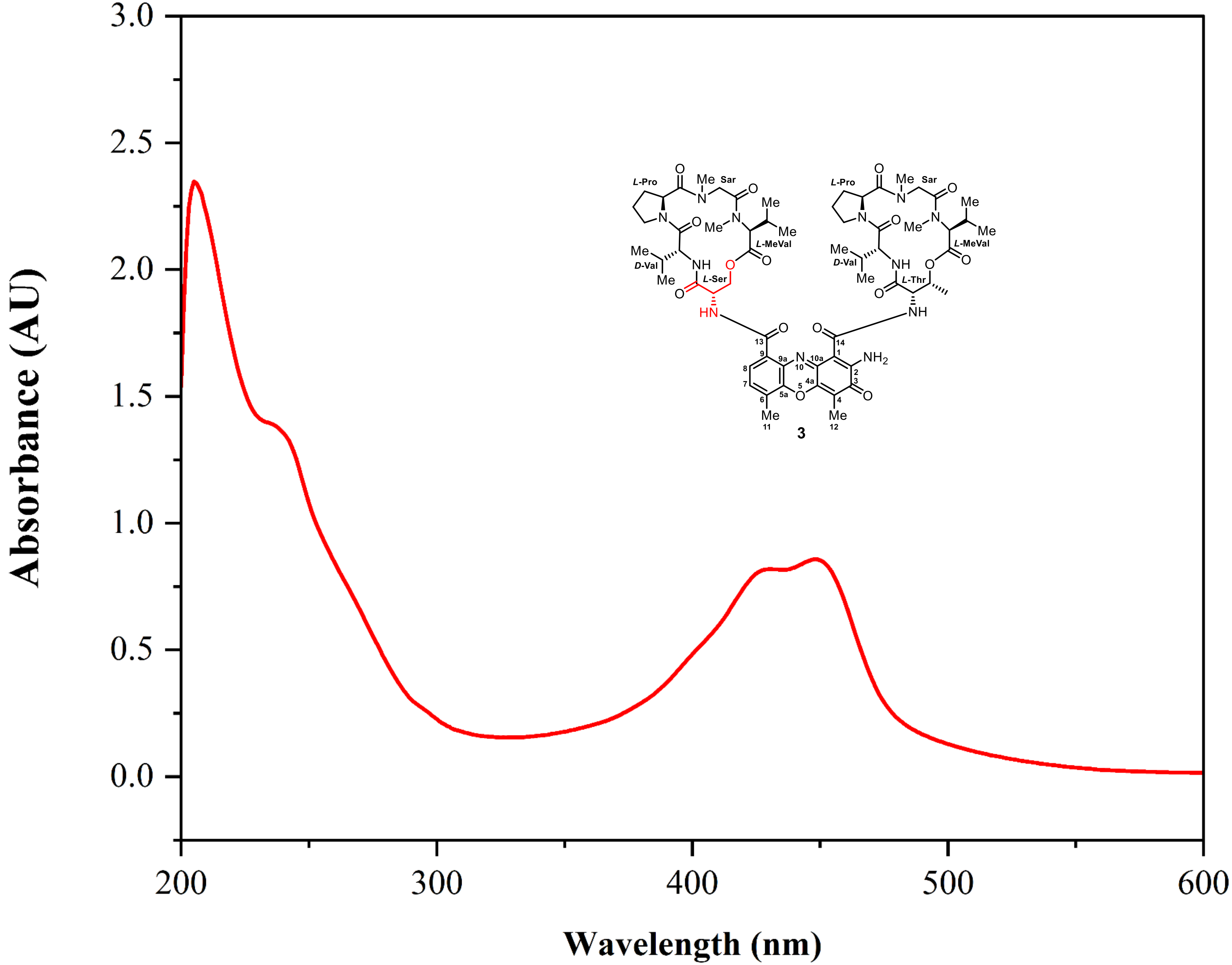

Figure S25. HR-ESIMS for compound **3**

Mass Spectrum SmartFormula Report

Analysis Info

Analysis Name	D:\Data\MS\data\202505\panghe_S-11-2_pos_34_01_19979.d	Acquisition Date	5/29/2025 5:43:54 PM
Method	LC_Direct Infusion_pos_100-1000mz.m	Operator	SCSIO
Sample Name	panghe_S-11-2_pos	Instrument	maXis 255552.00029
Comment			

Acquisition Parameter

Source Type	ESI	Ion Polarity	Positive	Set Nebulizer	0.4 Bar
Focus	Active	Set Capillary	4500 V	Set Dry Heater	180 °C
Scan Begin	100 m/z	Set End Plate Offset	-500 V	Set Dry Gas	4.0 l/min
Scan End	2000 m/z	Set Charging Voltage	0 V	Set Divert Valve	Waste
		Set Corona	0 nA	Set APCI Heater	0 °C

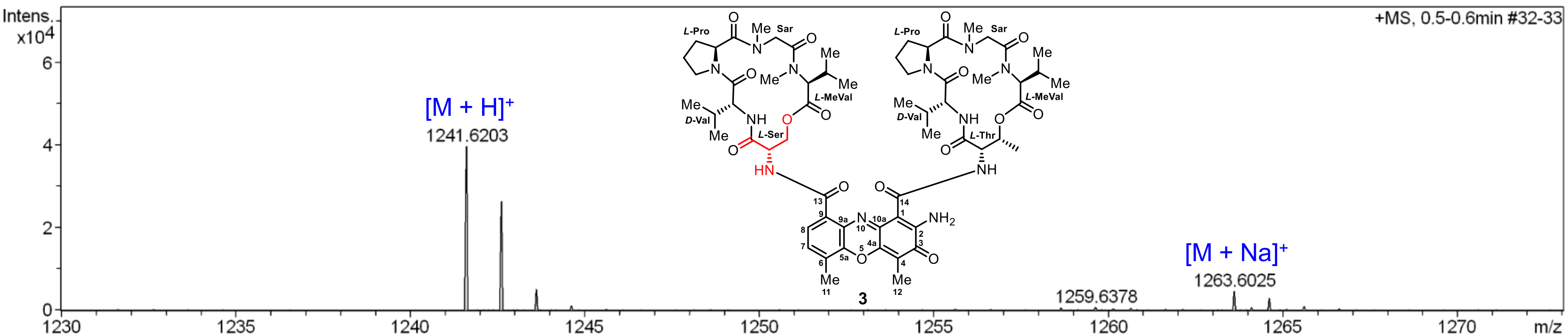

Meas. m/z	#	Ion Formula	Score	m/z	err [ppm]	err [mDa]	mSigma	rdb	e⁻ Conf	N-Rule
1241.6203	1	C61H85N12O16	100.00	1241.6201	-0.2	-0.2	71.6	25.5	even	ok
1263.6025	1	C61H84N12NaO16	100.00	1263.6020	-0.4	-0.5	51.4	25.5	even	ok

Figure S26. ^{1}H NMR (600 MHz) spectrum of compound **3** in $CDCl_3$

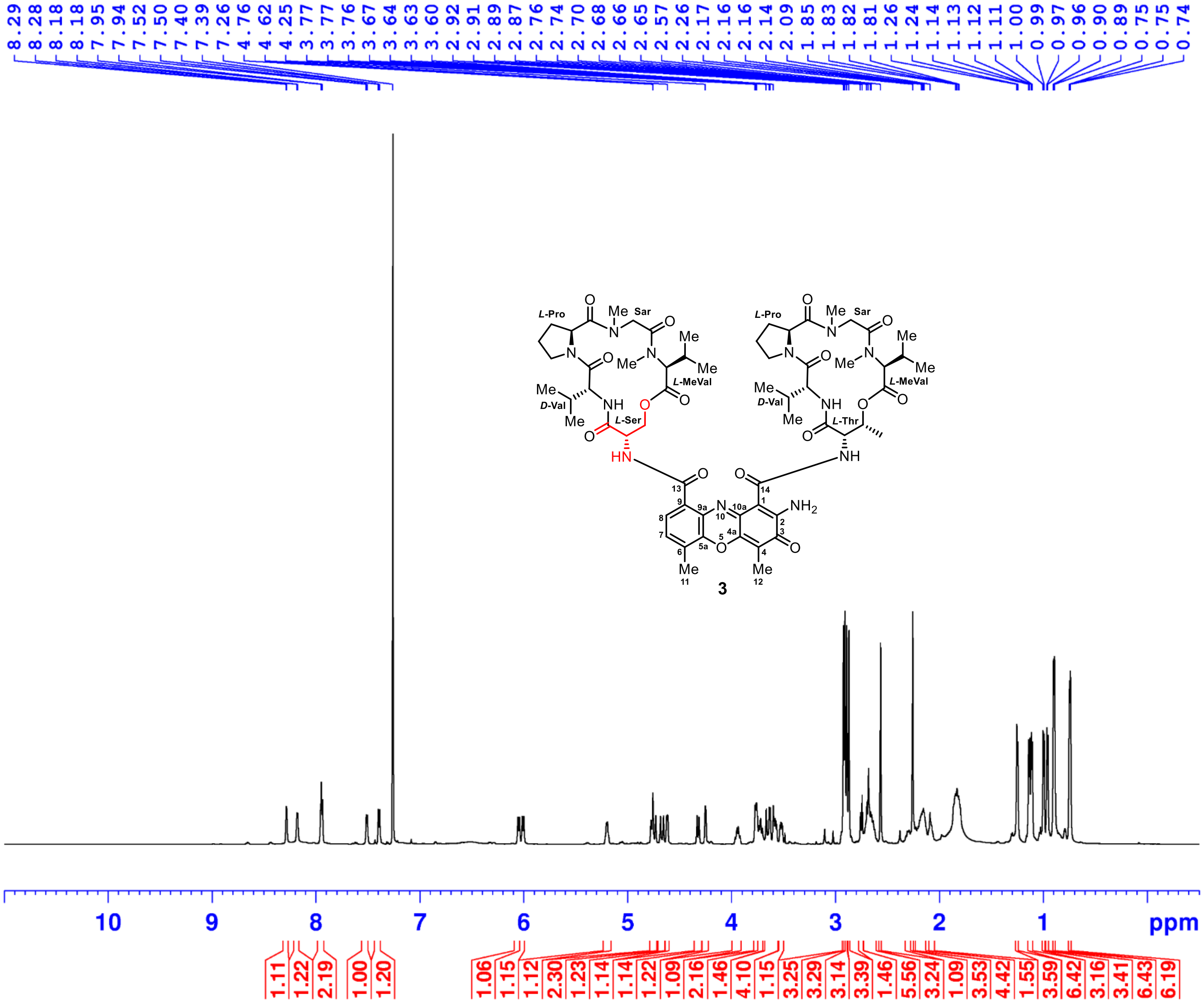

NAME	241110_S112_H
EXPNO	47
PROCNO	1
Date_	20241112
Time	20.55 h
INSTRUM	Avance NEO 600
PROBHD	Z168348_0005 (
PULPROG	zg30
TD	65536
SOLVENT	CDCl3
NS	64
DS	2
SWH	11904.762 Hz
FIDRES	0.363304 Hz
AQ	2.7525620 sec
RG	22.6
DW	42.000 usec
DE	14.39 usec
TE	298.2 K
D1	1.00000000 sec
TD0	1
SFO1	600.1937062 MHz
NUC1	1H
P0	2.67 usec
P1	8.00 usec
SI	65536
SF	600.1900148 MHz
WDW	EM
SSB	0
LB	0.30 Hz
GB	0
PC	1.00

Figure S26-continue

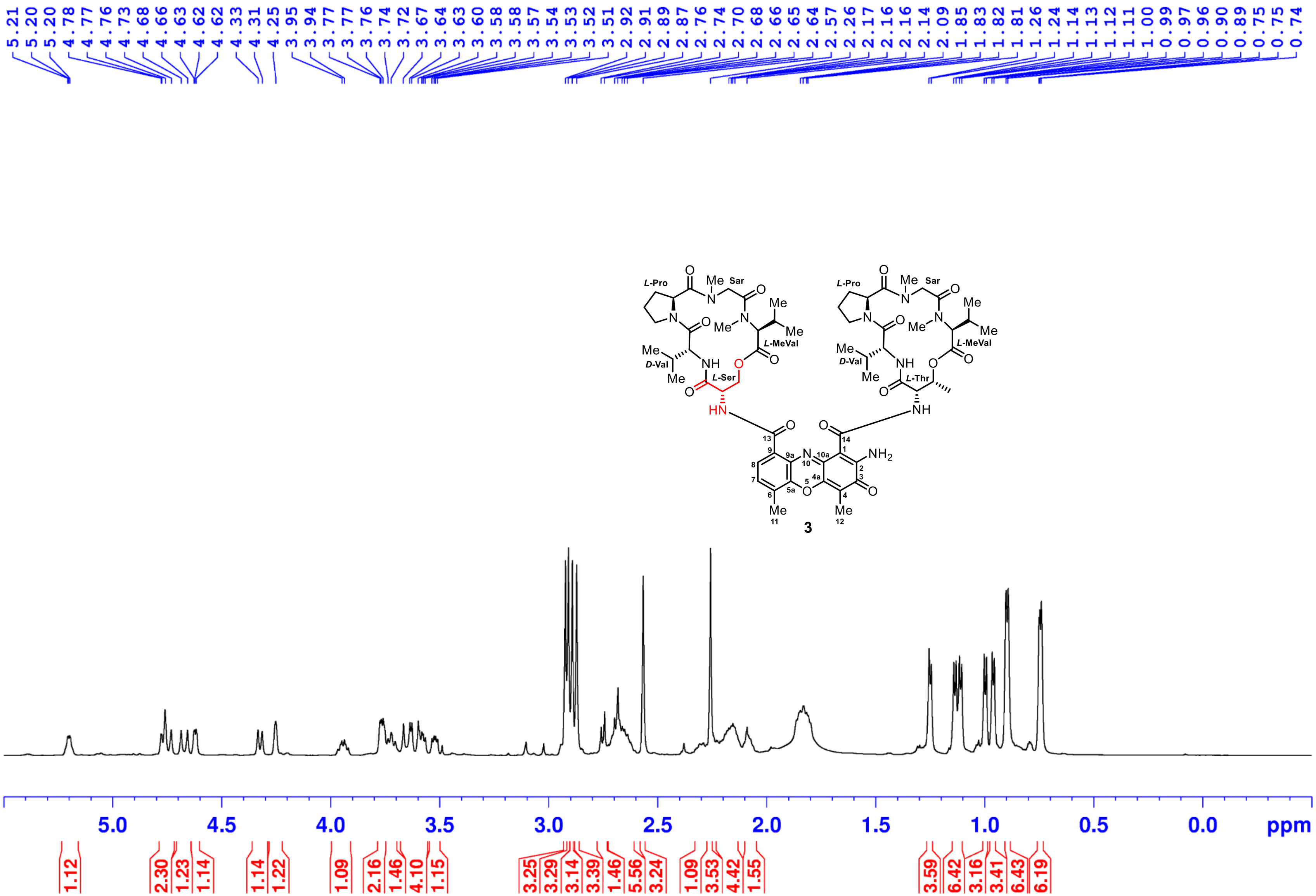

Figure S26-continue

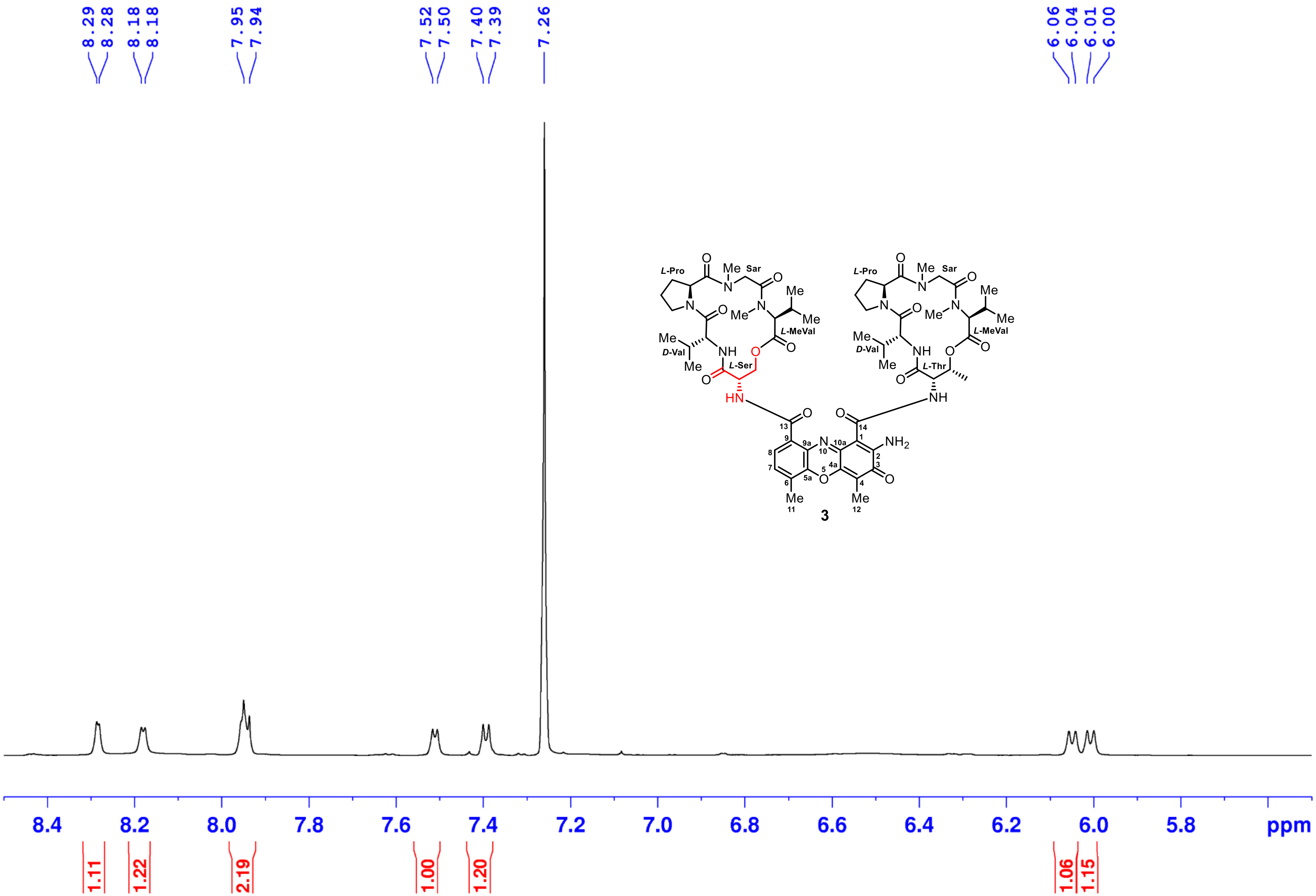

Figure S27. ^{13}C NMR (150 MHz) spectrum of compound **3** in $CDCl_3$

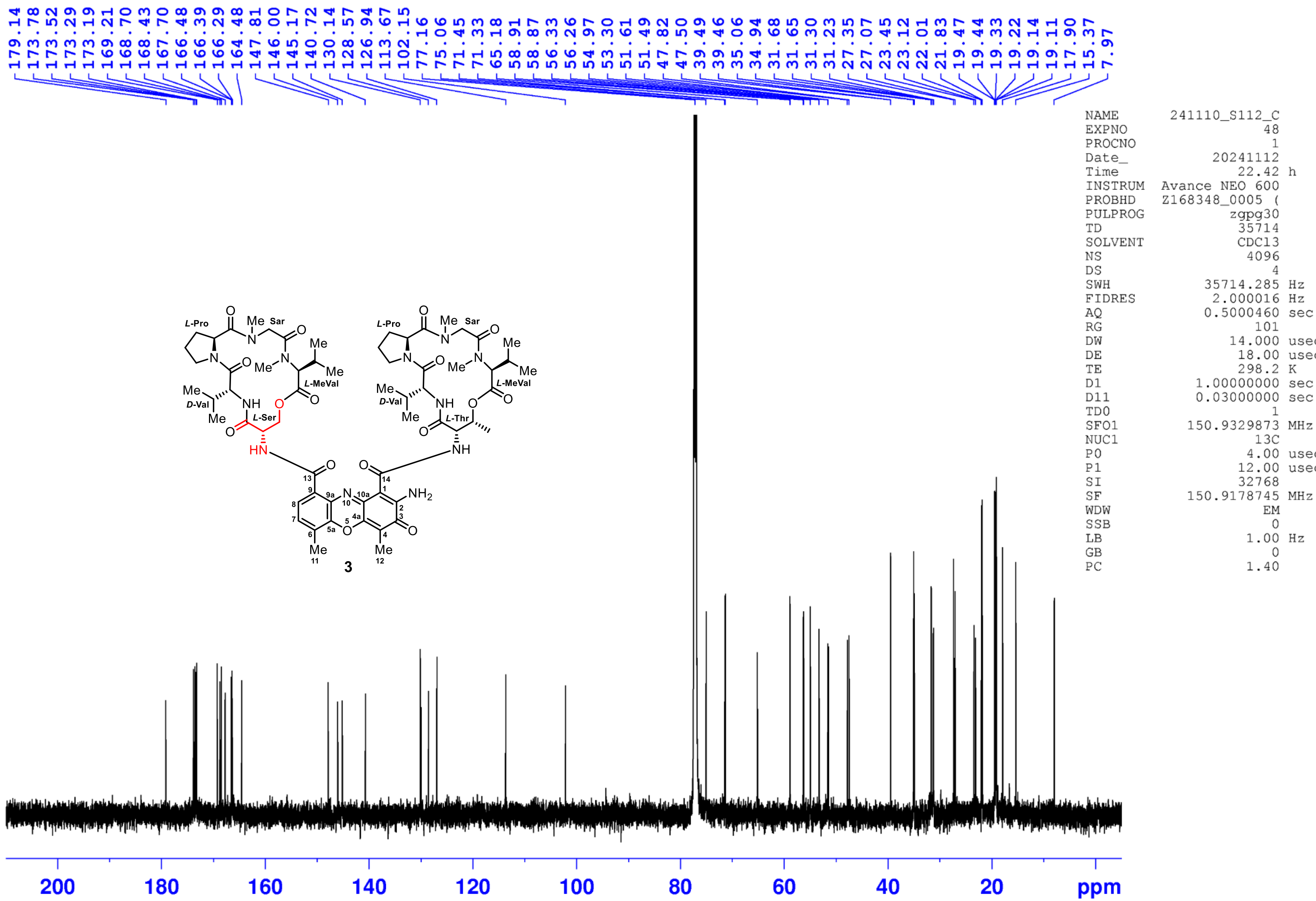

Figure S27-continue

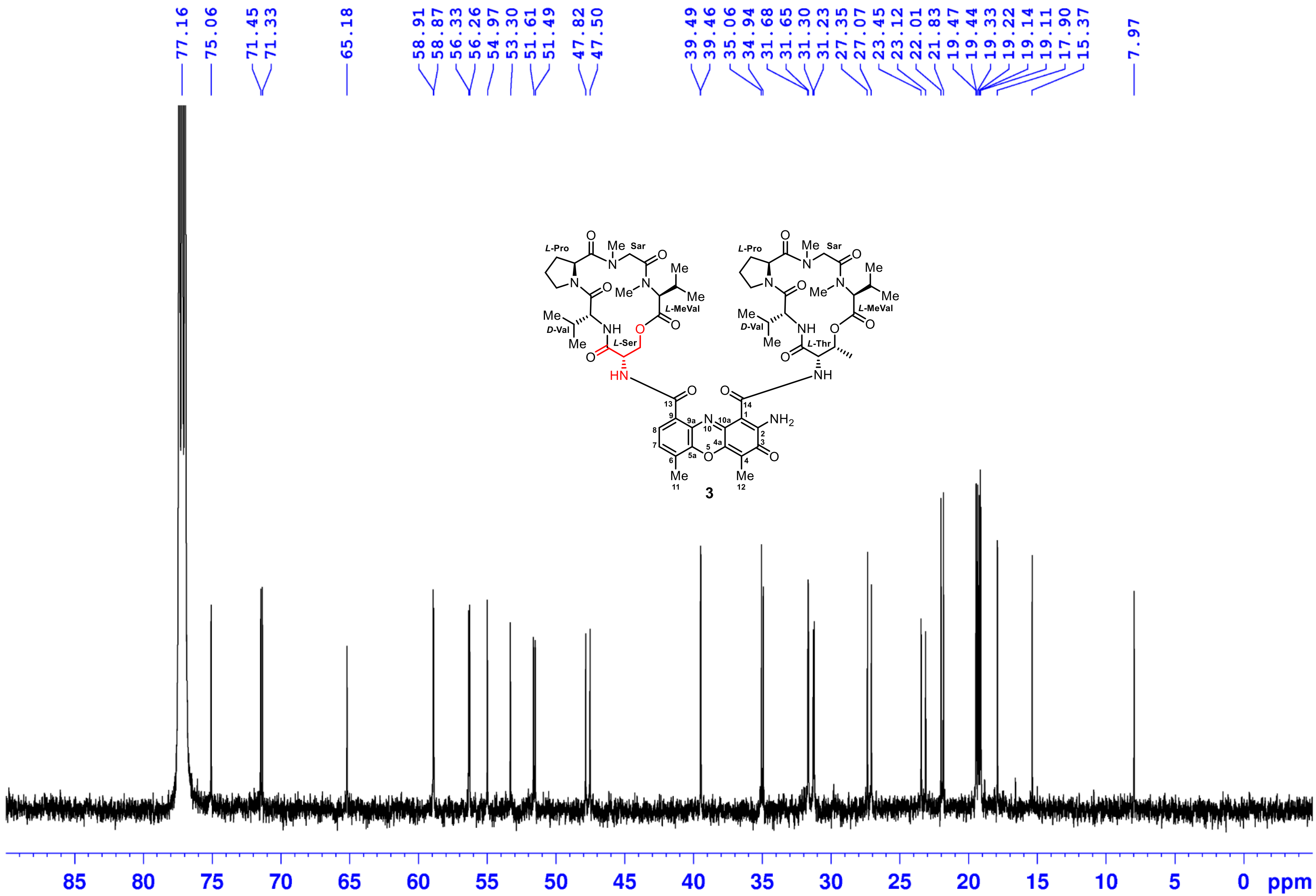

Figure S27-continue

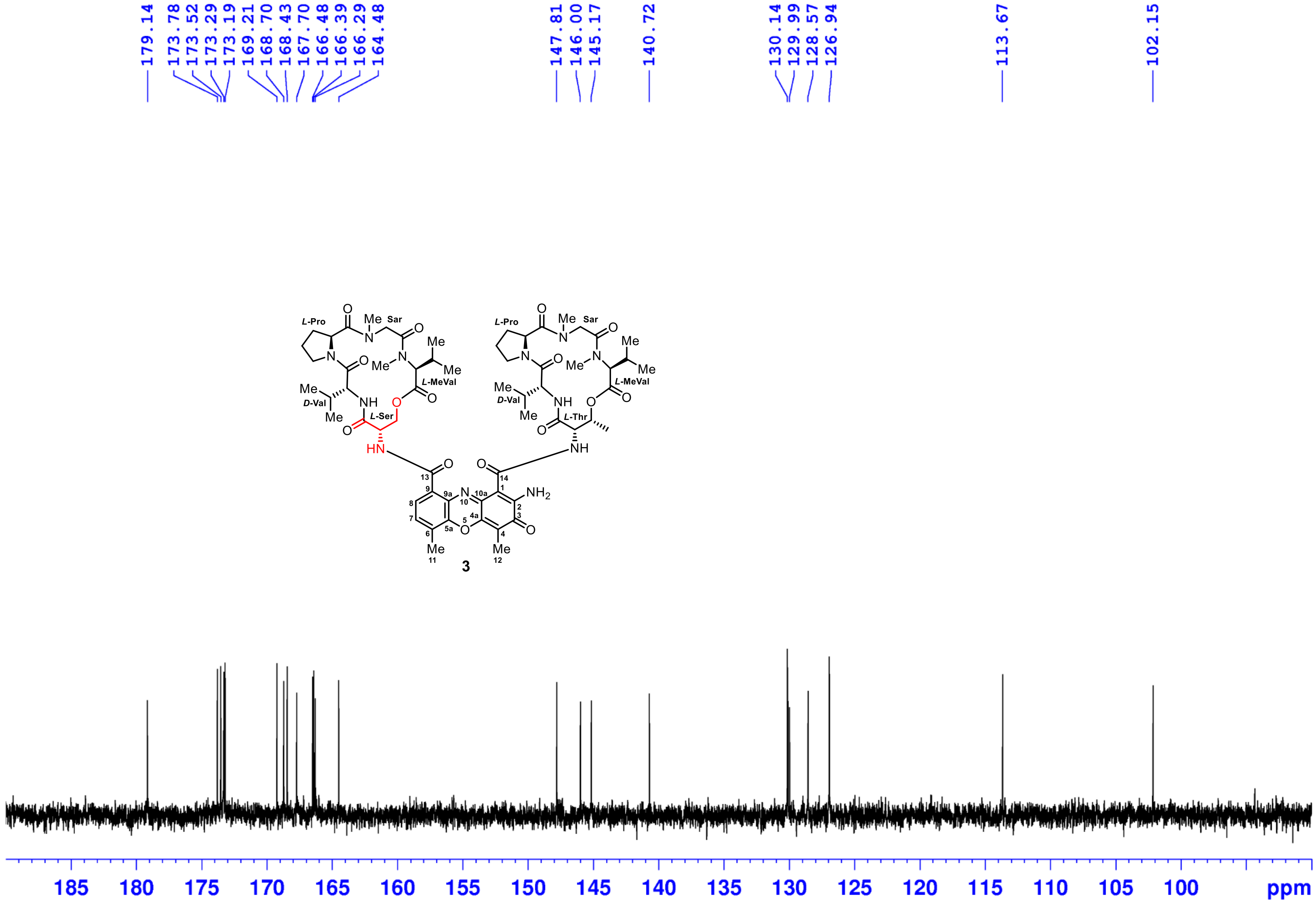

3

Figure S28. DEPT135 (150 MHz) spectrum of compound **3** in $CDCl_3$

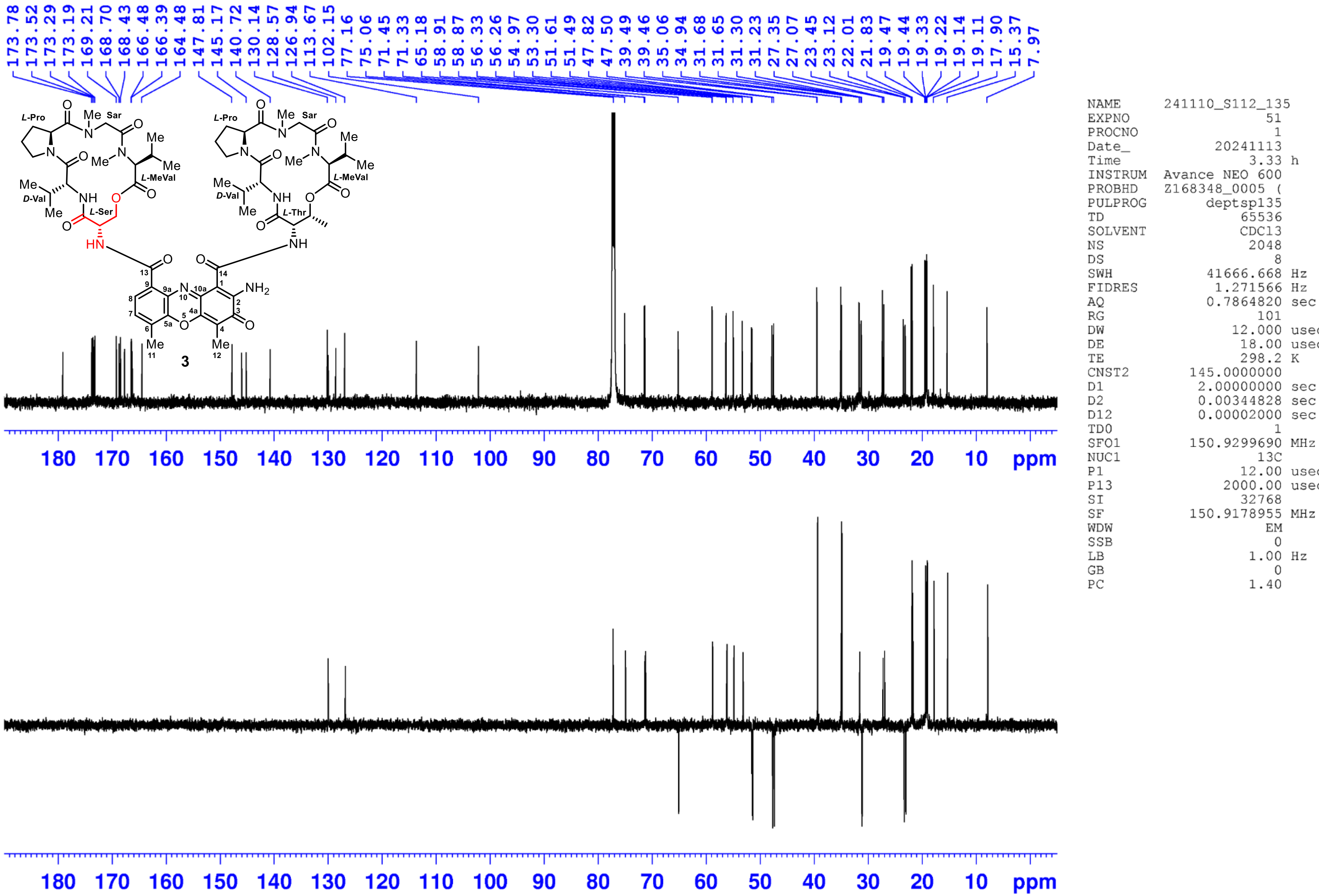

Figure S28-continue

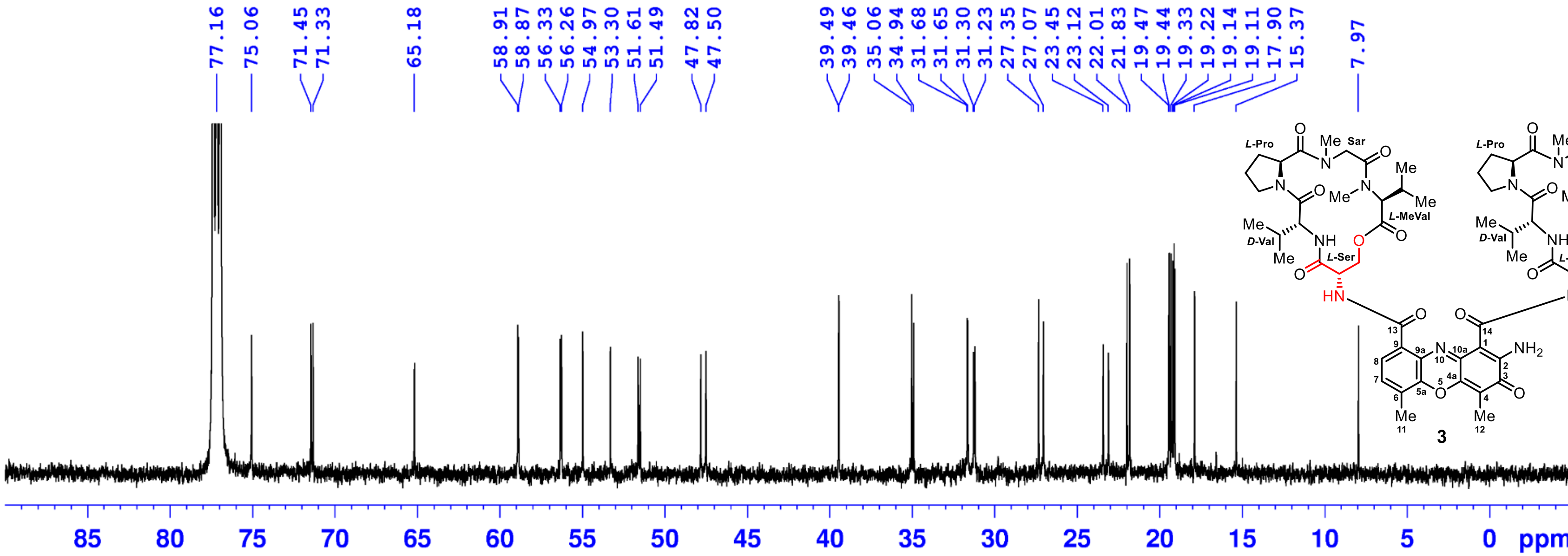

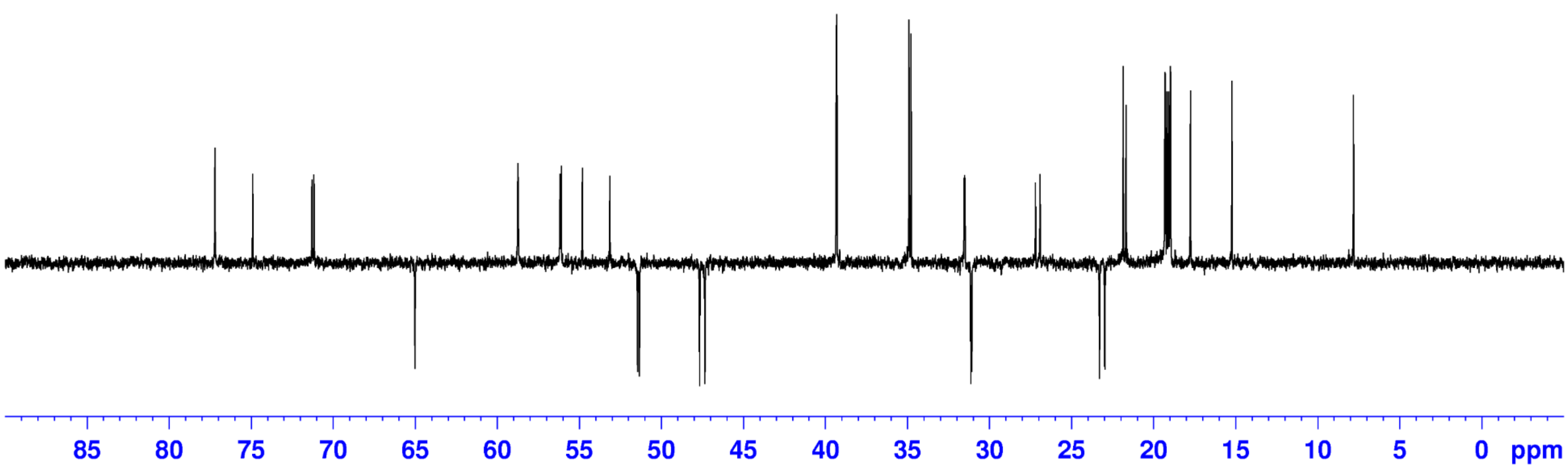

Figure S28-continue

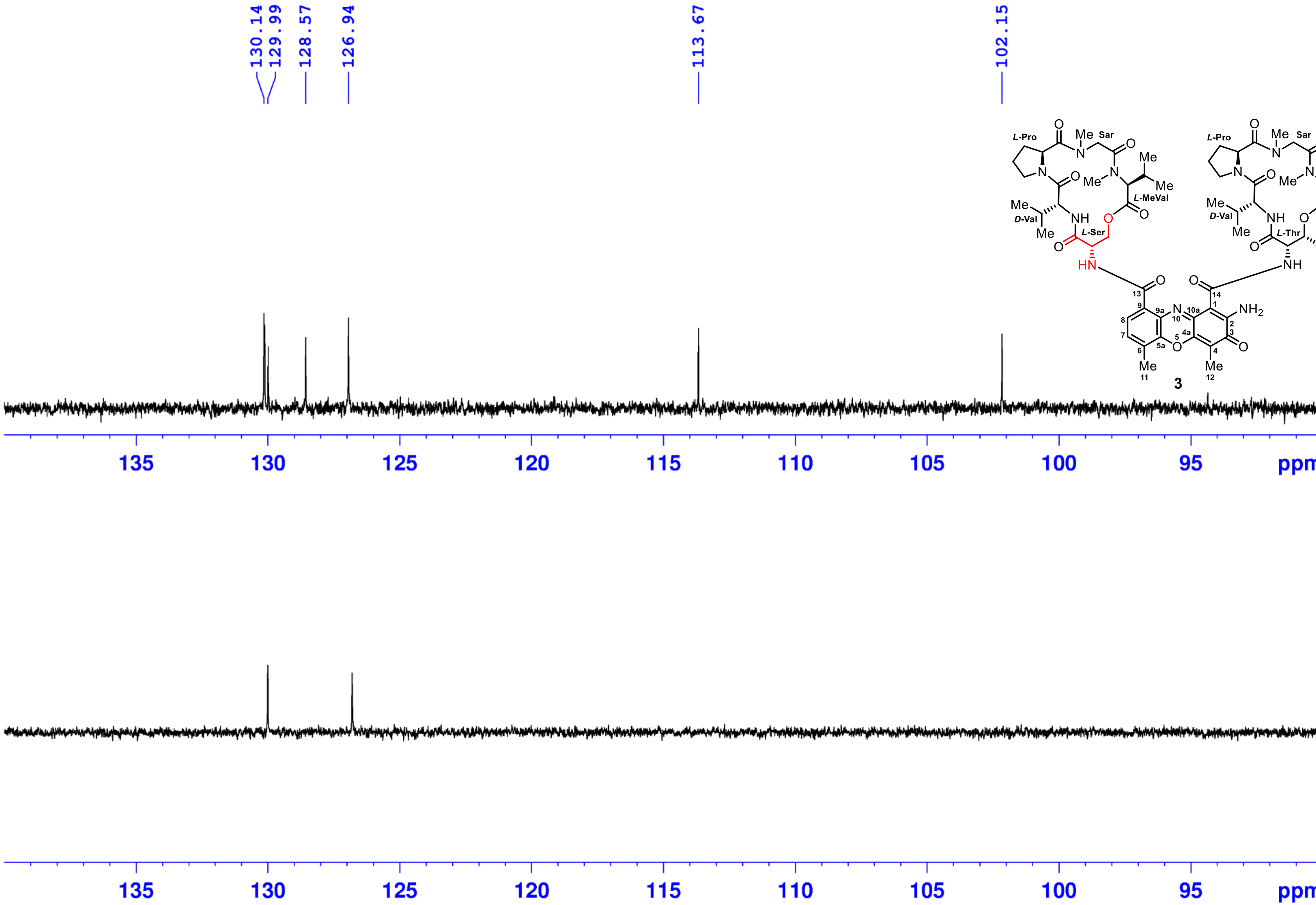

Figure S29. ^{1}H–^{1}H COSY (600 MHz) spectrum of compound **3** in $CDCl_3$

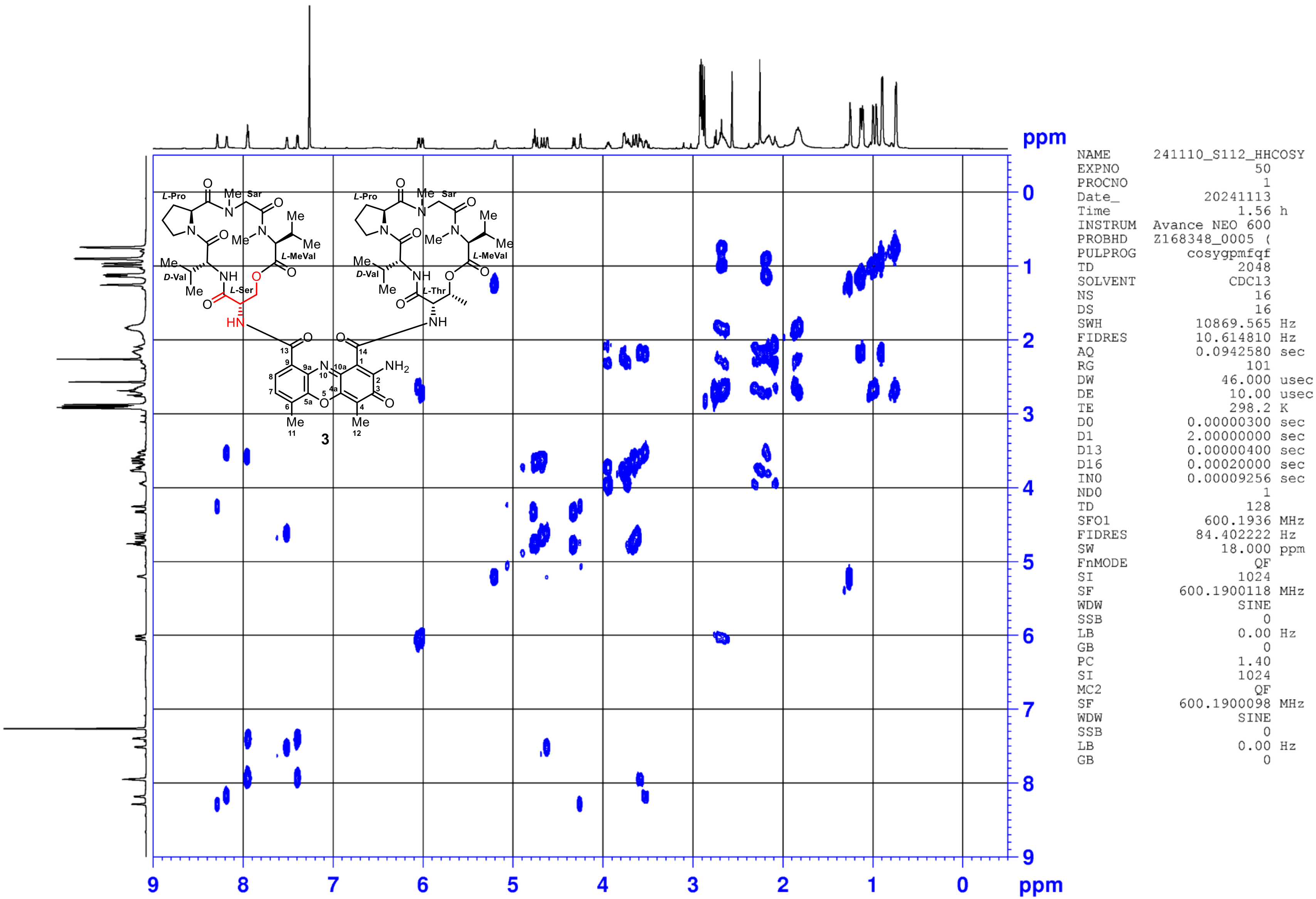

Figure S29-continue

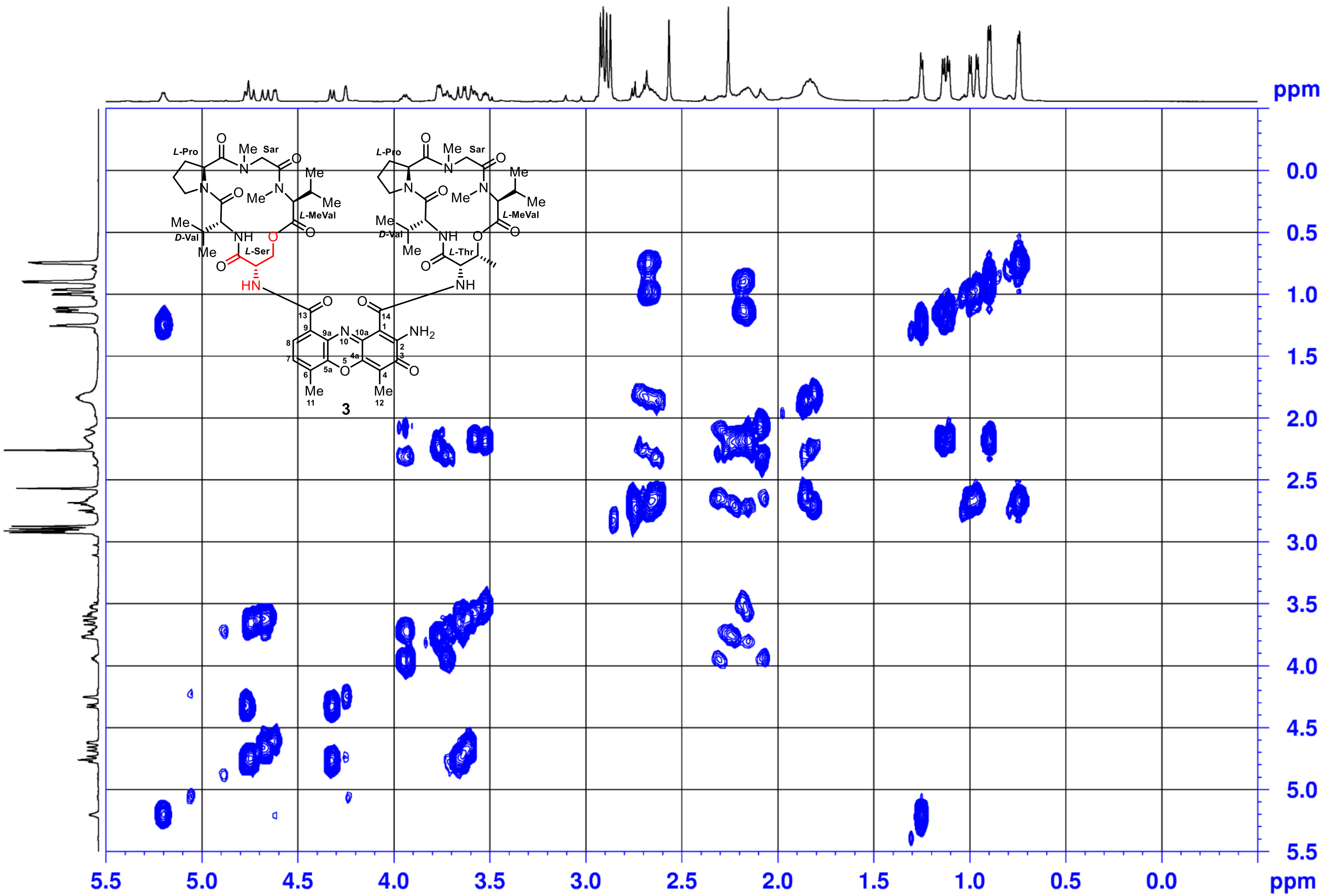

Figure S29-continue

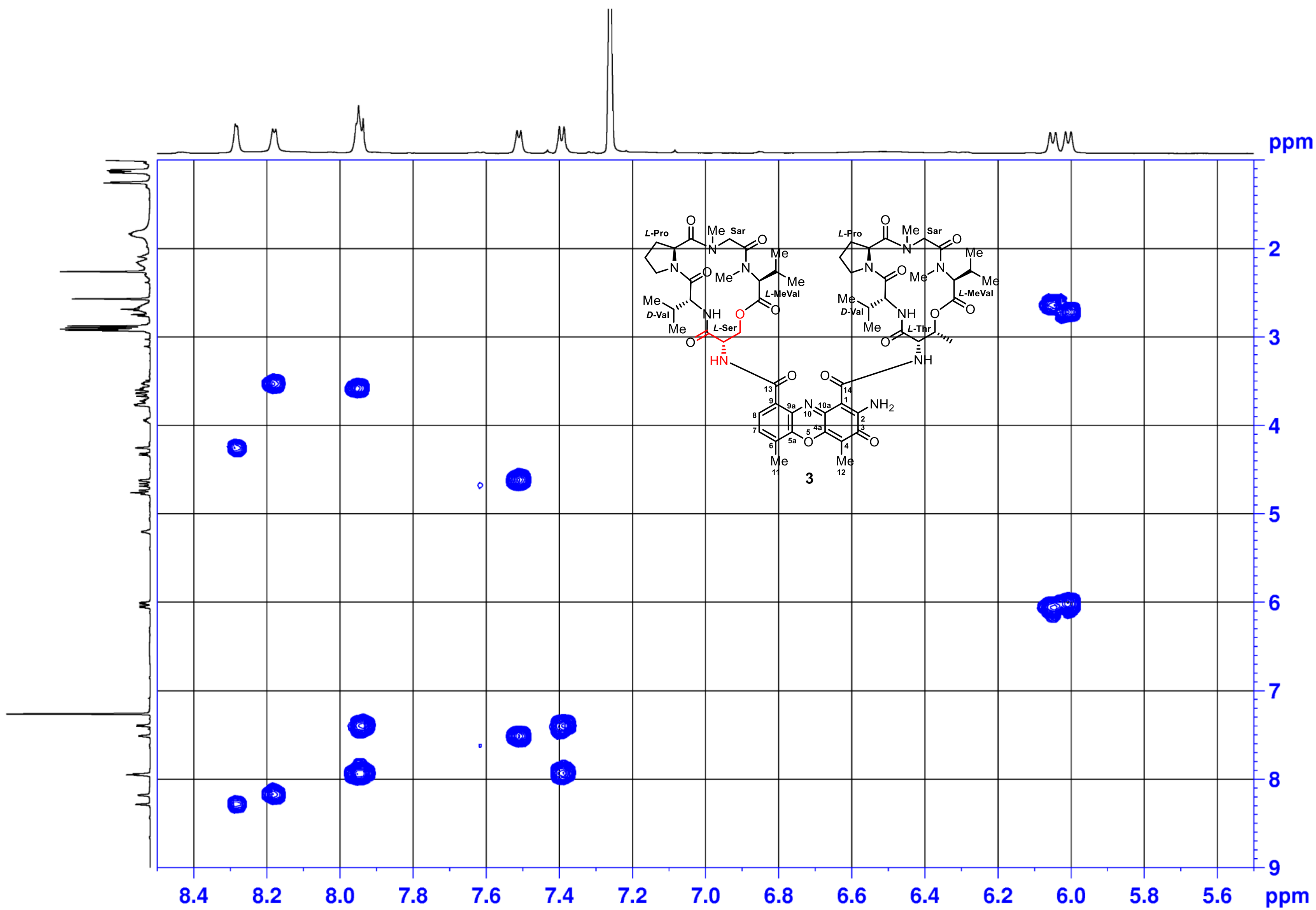

Figure S30. HSQC (600 MHz) spectrum of compound **3** in $CDCl_3$

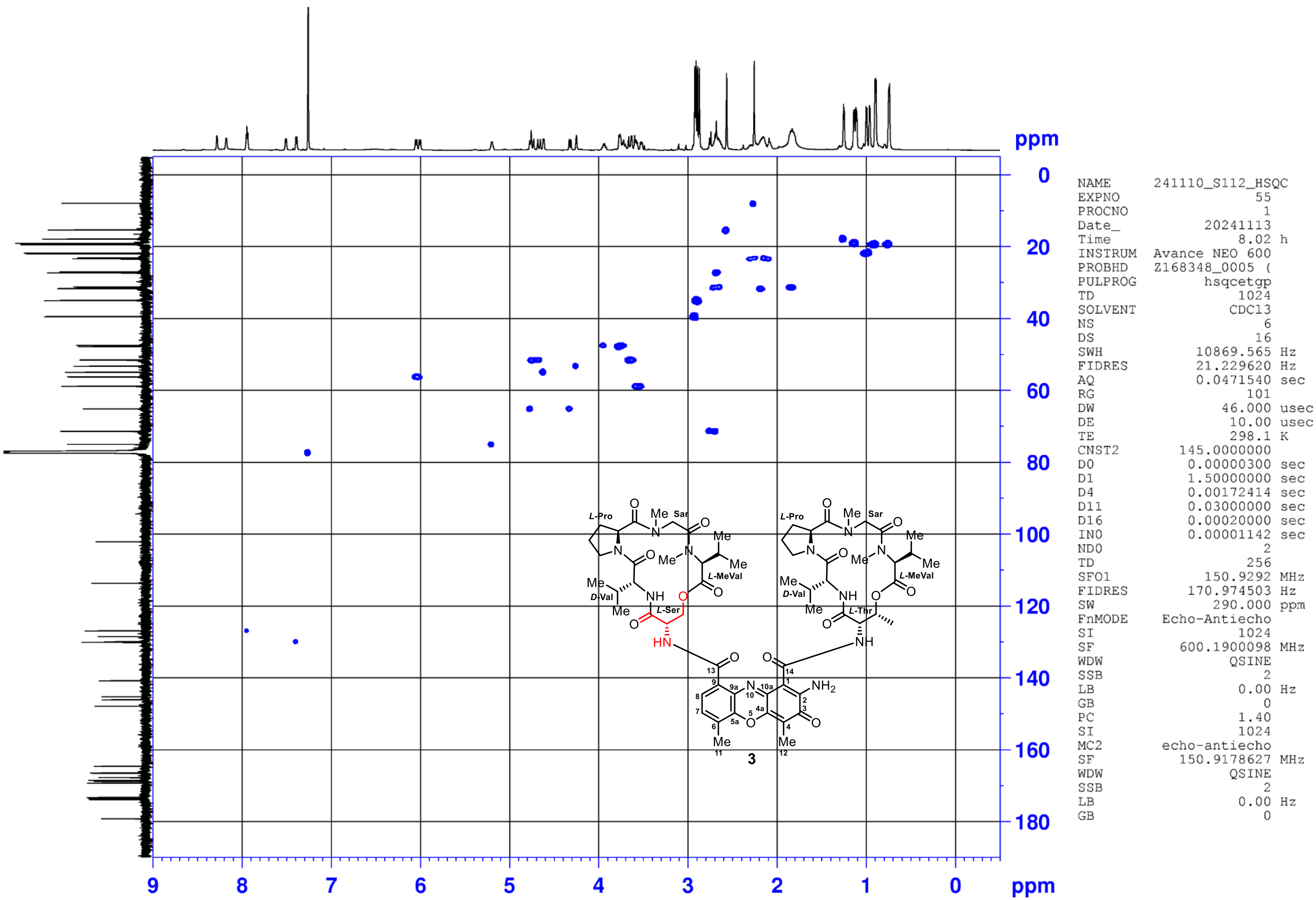

Figure S30-continue

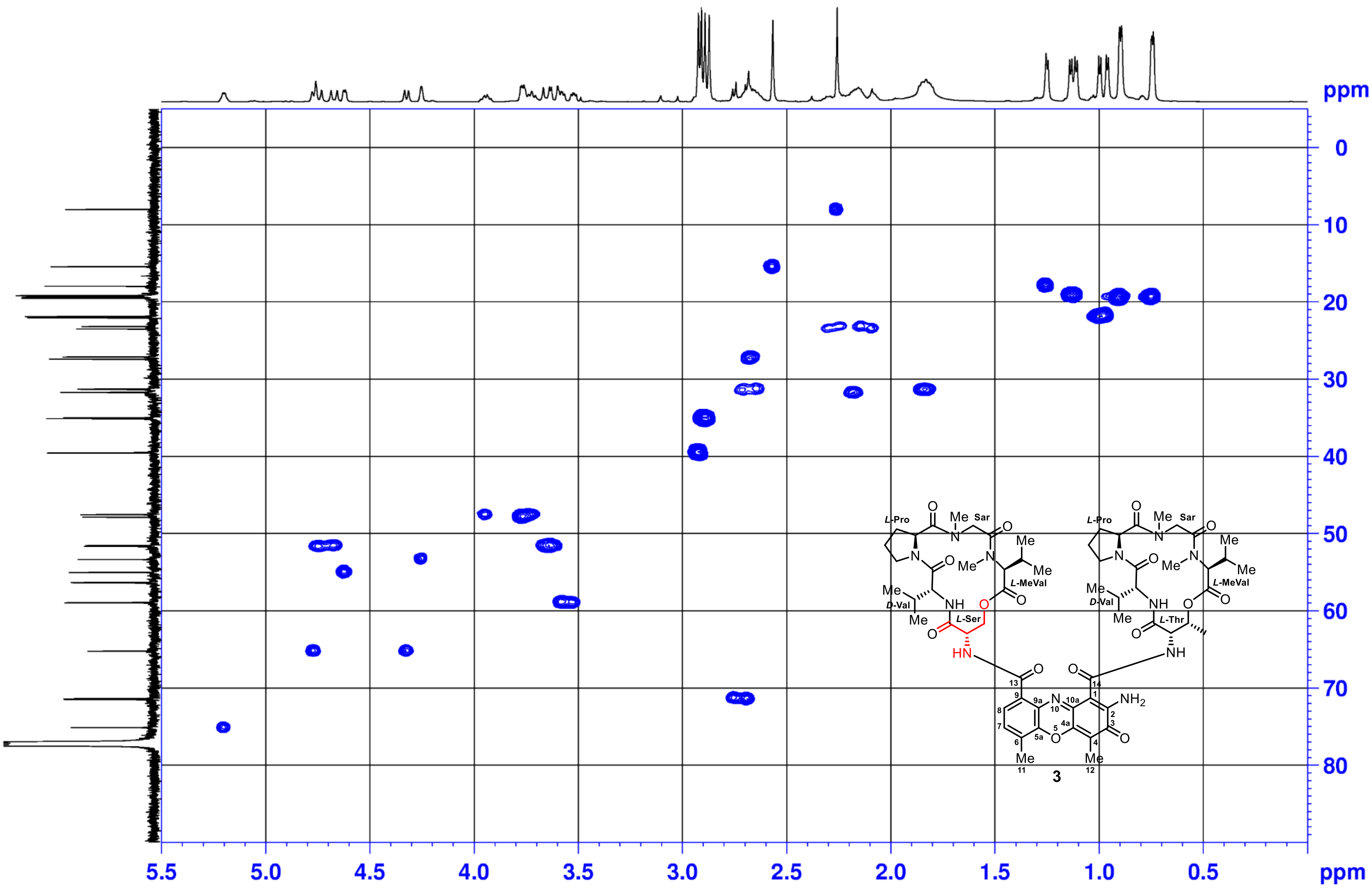

Figure S30-continue

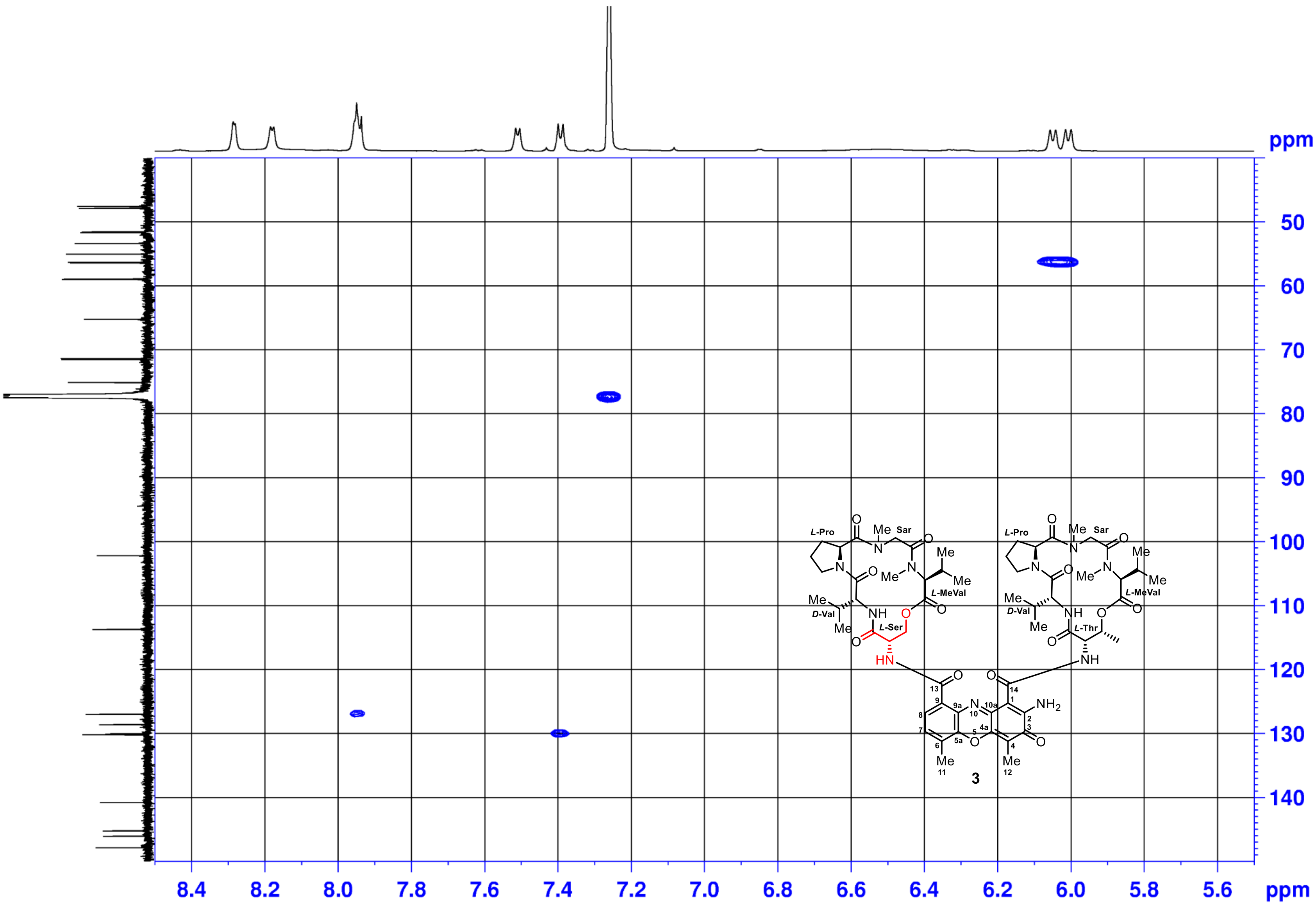

Figure S31. HMBC (600 MHz) spectrum of compound **3** in $CDCl_3$

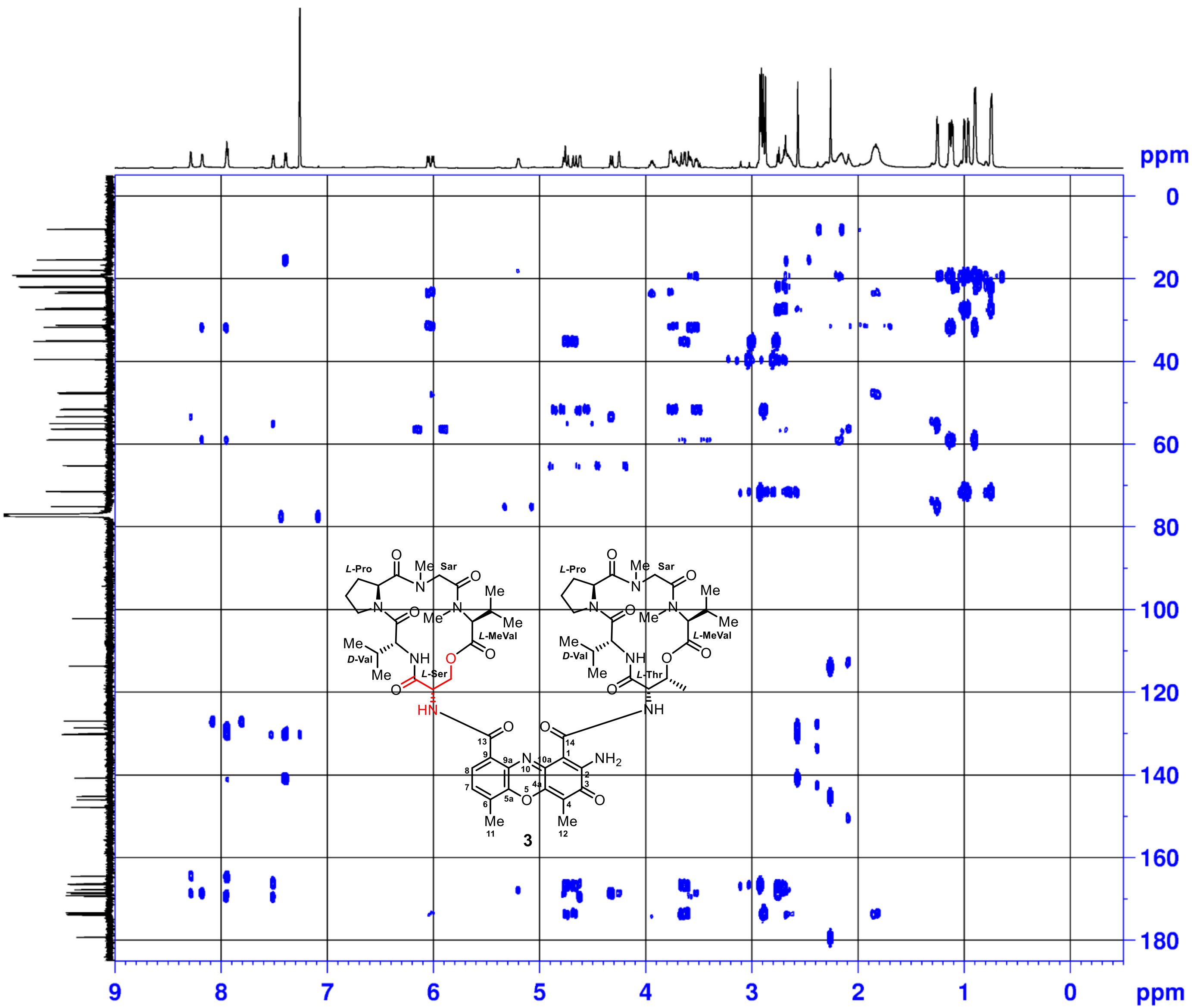

NAME	241110_S112_HMBC
EXPNO	49
PROCNO	1
Date_	20241113
Time	0.43 h
INSTRUM	Avance NEO 600
PROBHD	Z168348_0005 (
PULPROG	hmbcgpndqf
TD	4096
SOLVENT	CDCl3
NS	32
DS	16
SWH	10869.565 Hz
FIDRES	5.307405 Hz
AQ	0.1884660 sec
RG	101
DW	46.000 usec
DE	10.00 usec
TE	298.2 K
CNST13	8.0000000
D0	0.00000300 sec
D1	1.50000000 sec
D6	0.06250000 sec
D16	0.00020000 sec
IN0	0.00001506 sec
ND0	2
TD	128
SFO1	150.933 MHz
FIDRES	259.416077 Hz
SW	220.000 ppm
FnMODE	QF
SI	4096
SF	600.1900149 MHz
WDW	QSINE
SSB	0
LB	0.00 Hz
GB	0
PC	1.40
SI	1024
MC2	QF
SF	150.9178580 MHz
WDW	QSINE
SSB	0
LB	0.00 Hz
GB	0

Figure S31-continue

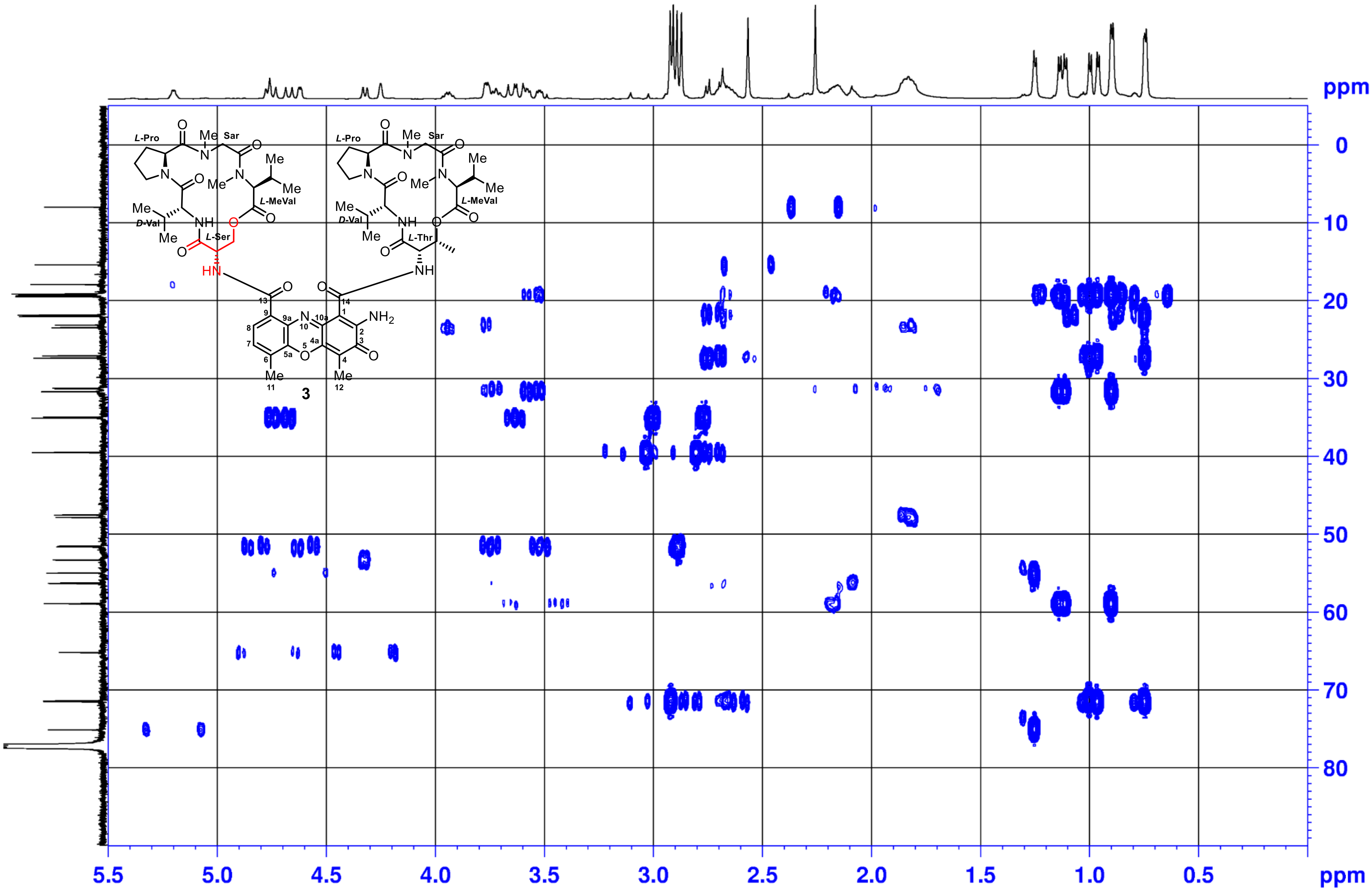

Figure S31-continue

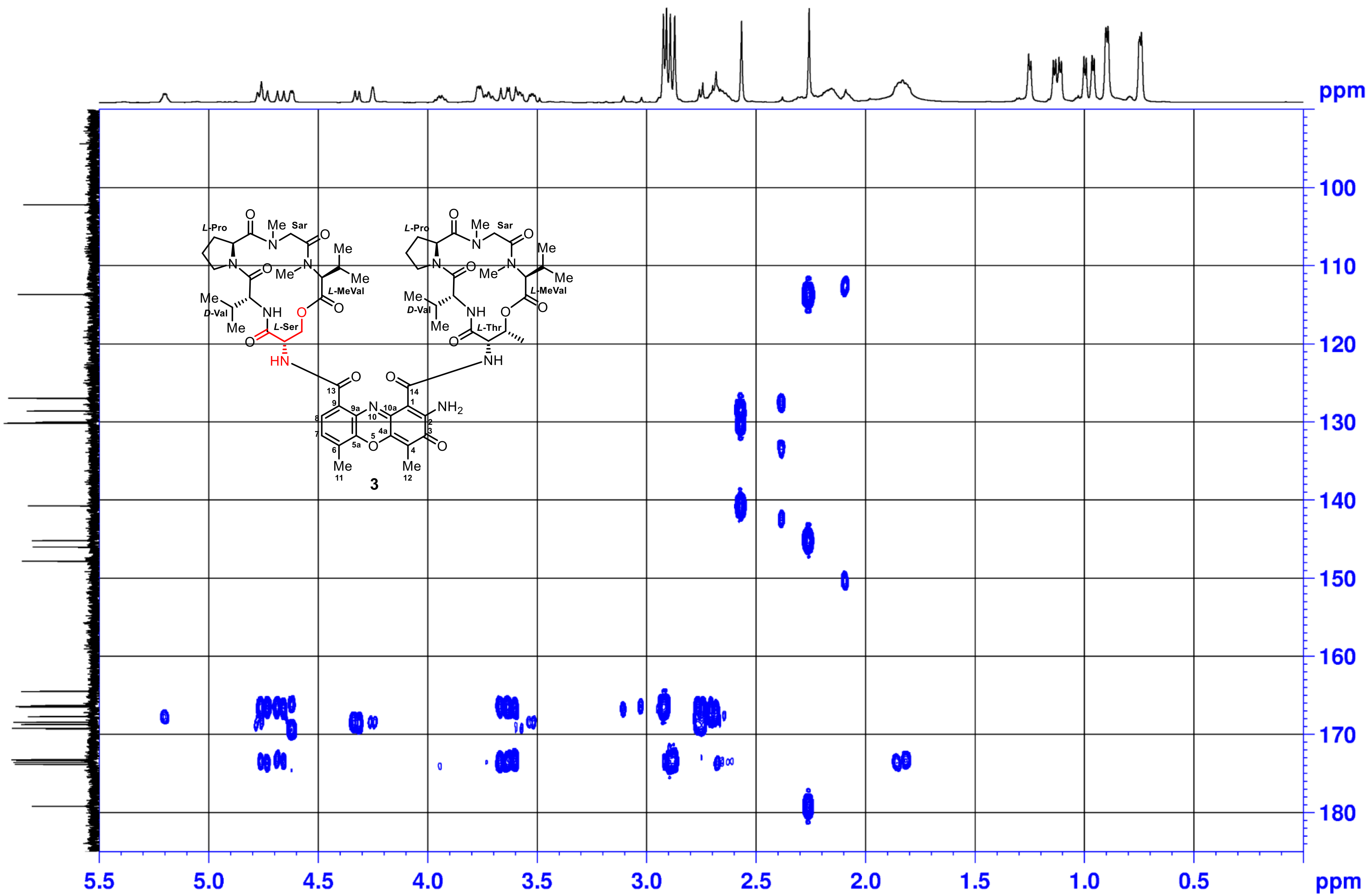

Figure S31-continue

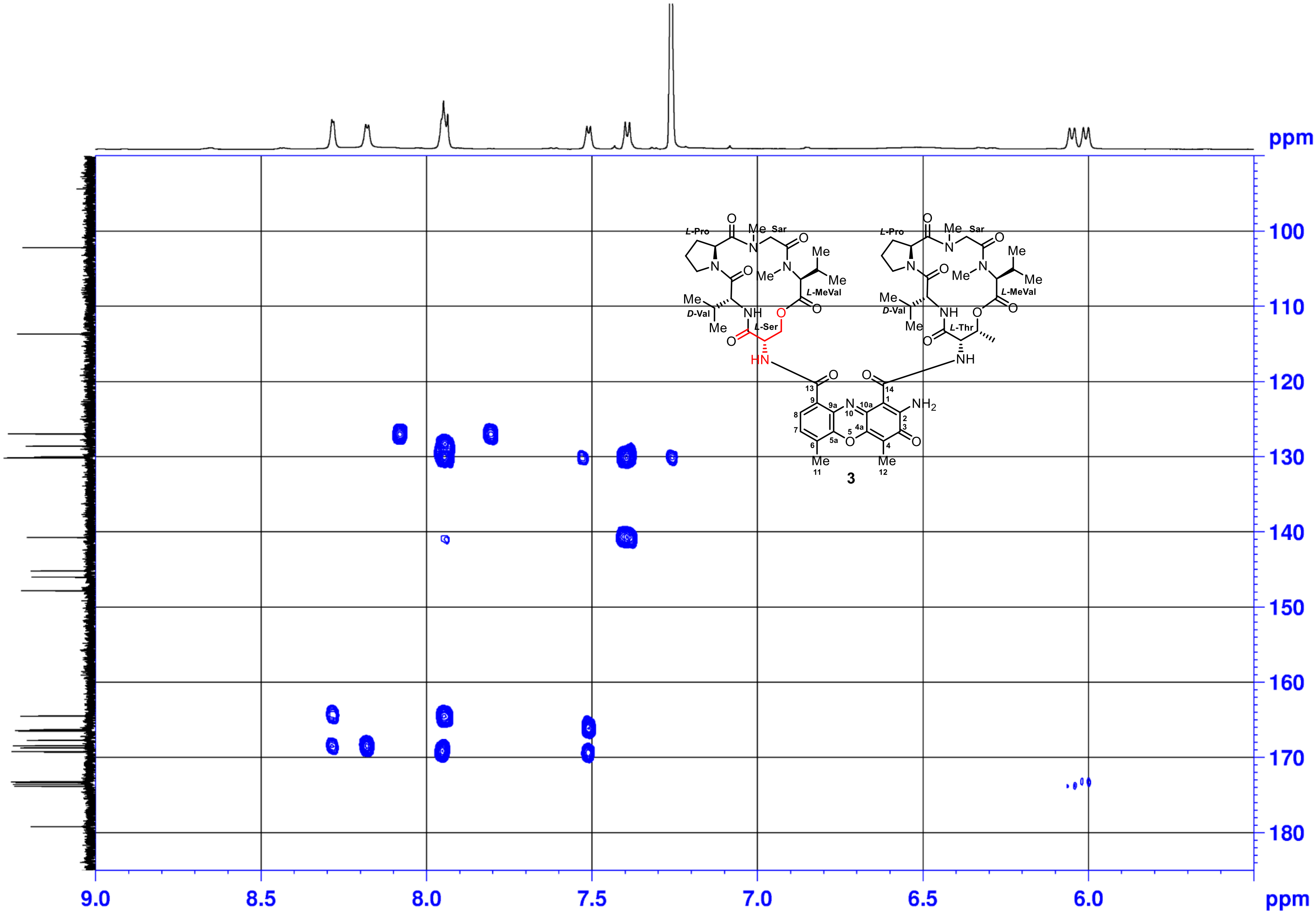

Figure S31-continue

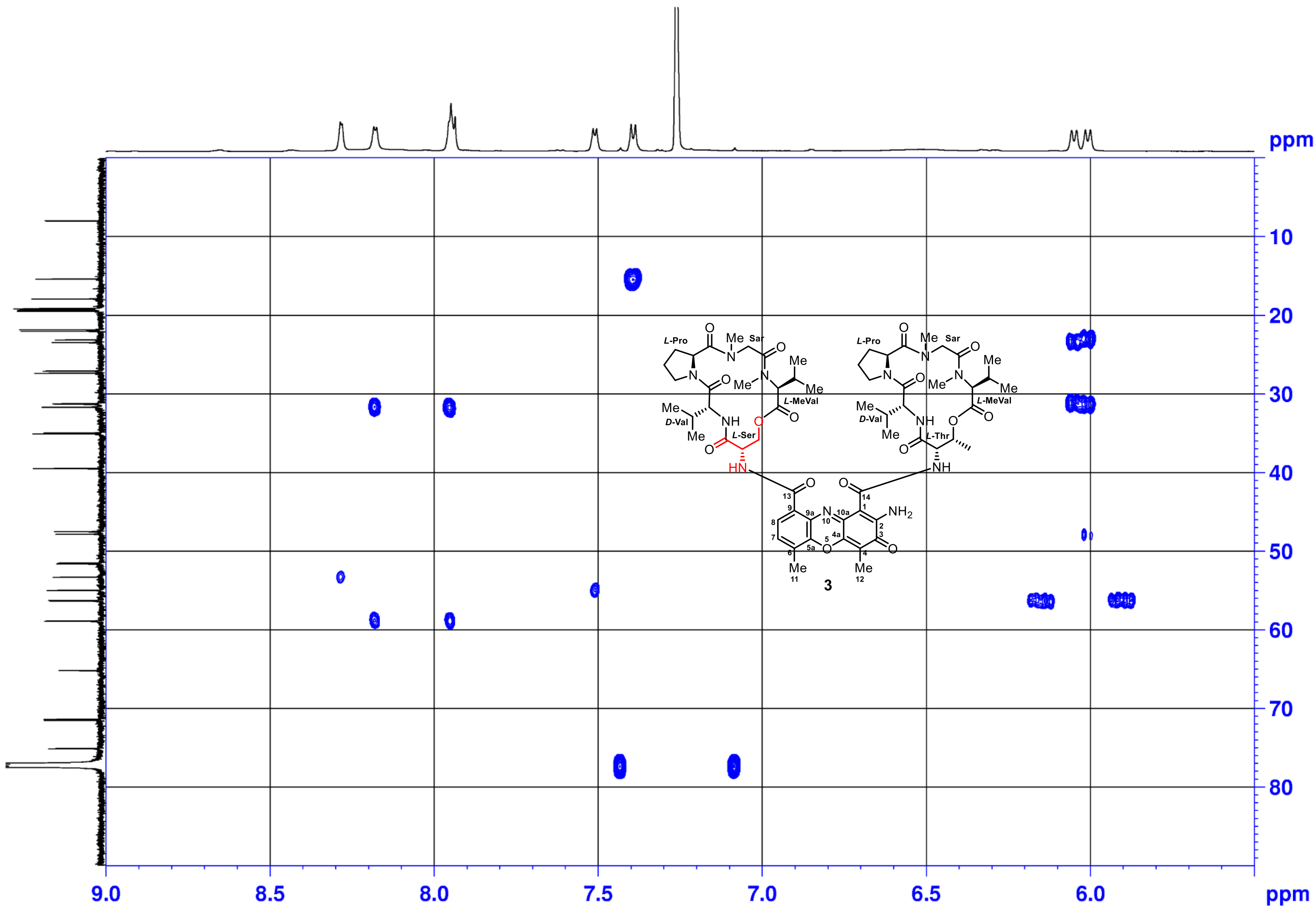

Figure S32. NOESY (600 MHz) spectrum of compound **3** in $CDCl_3$

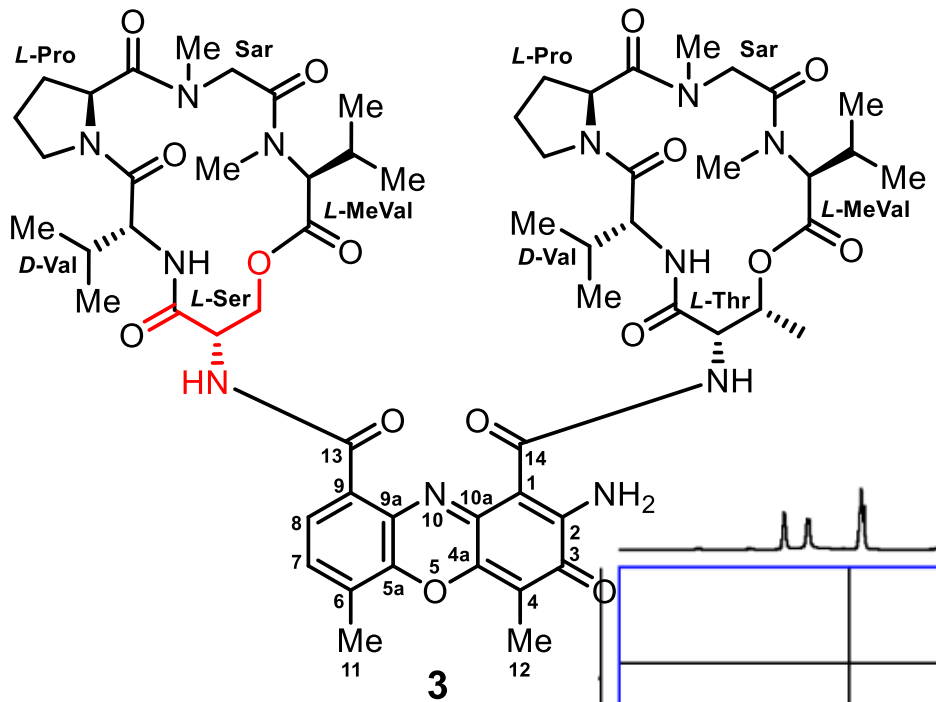

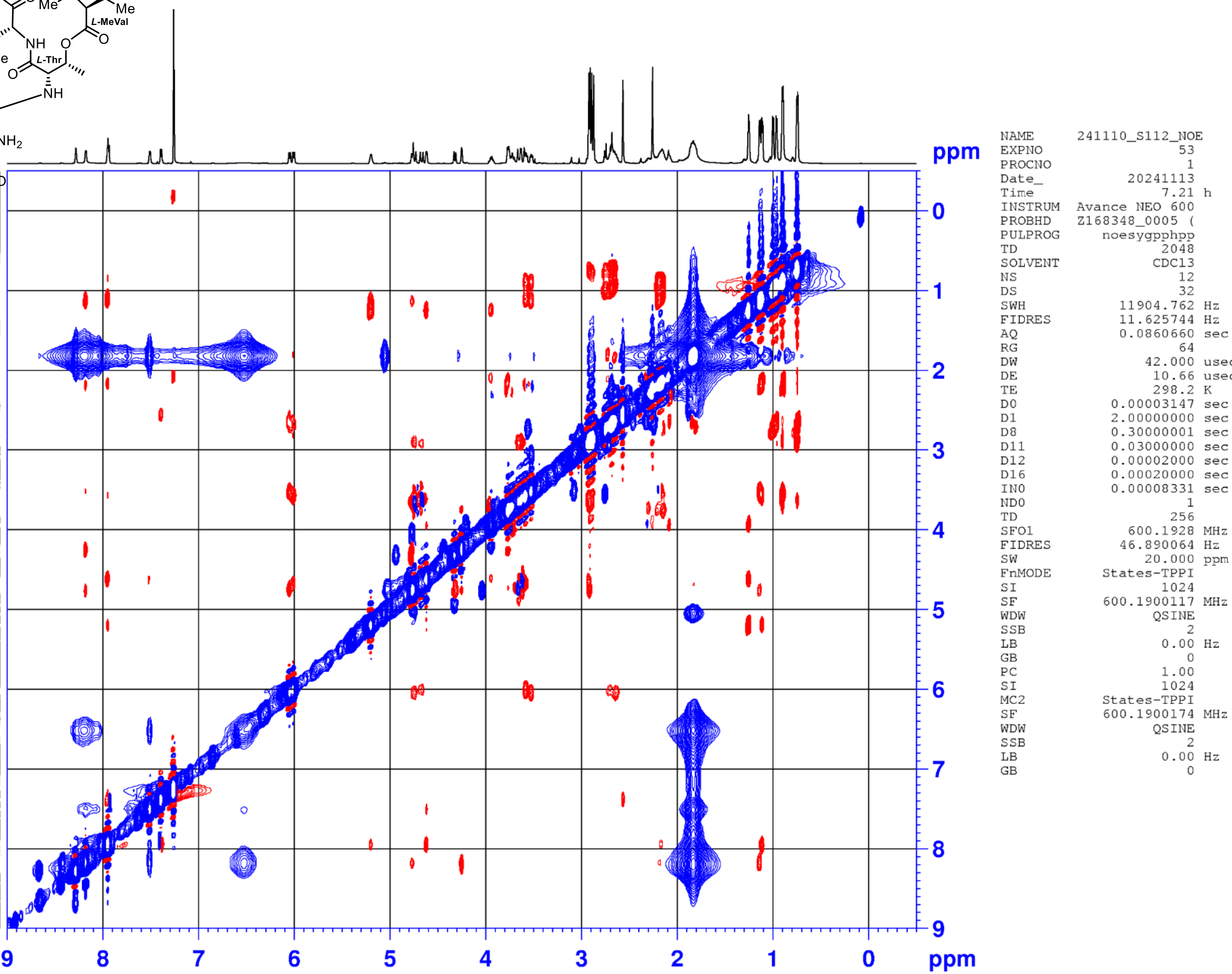

Figure S32-continue

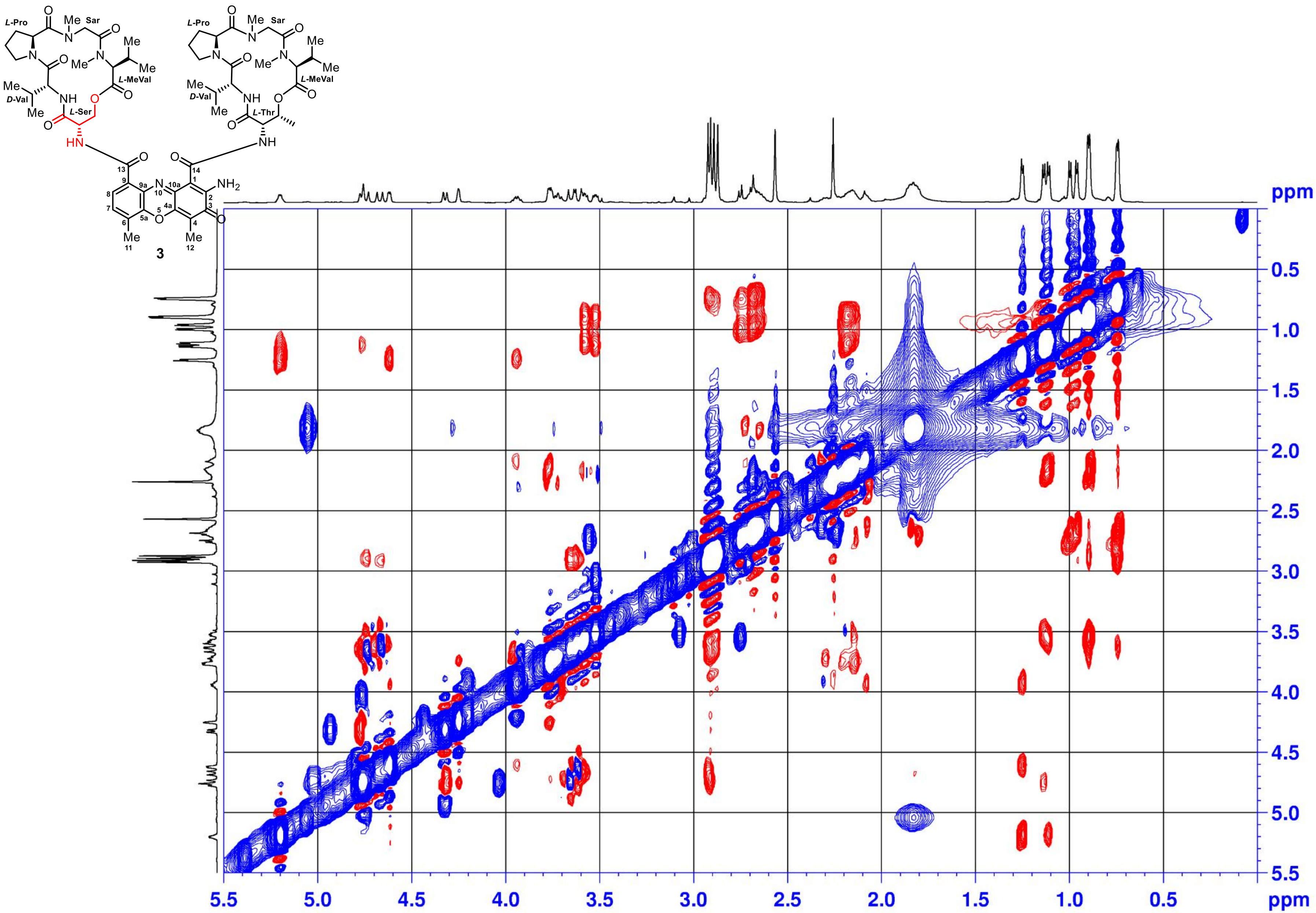

Figure S32-continue

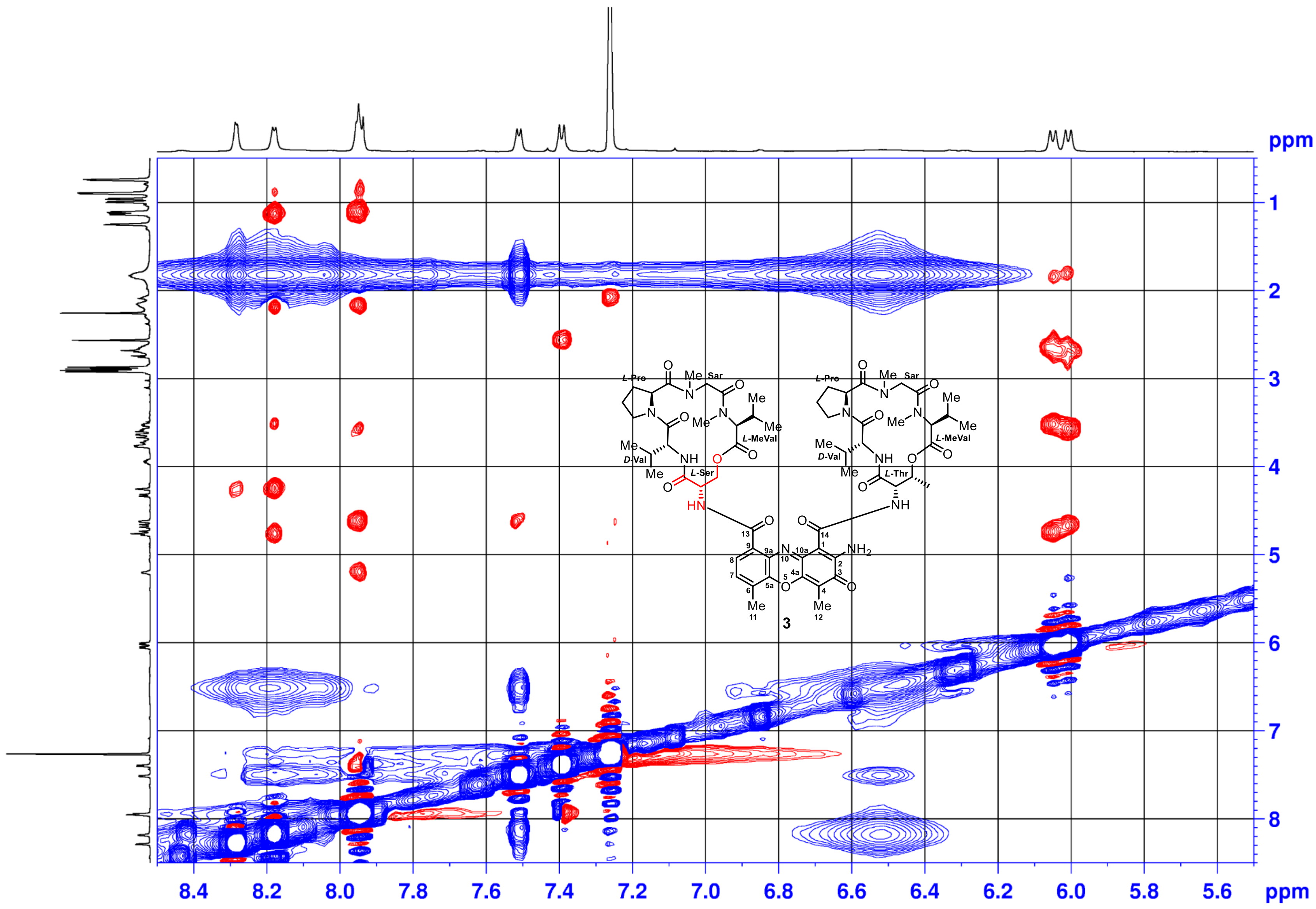

Figure S33. Marfey's analysis of Ser standard

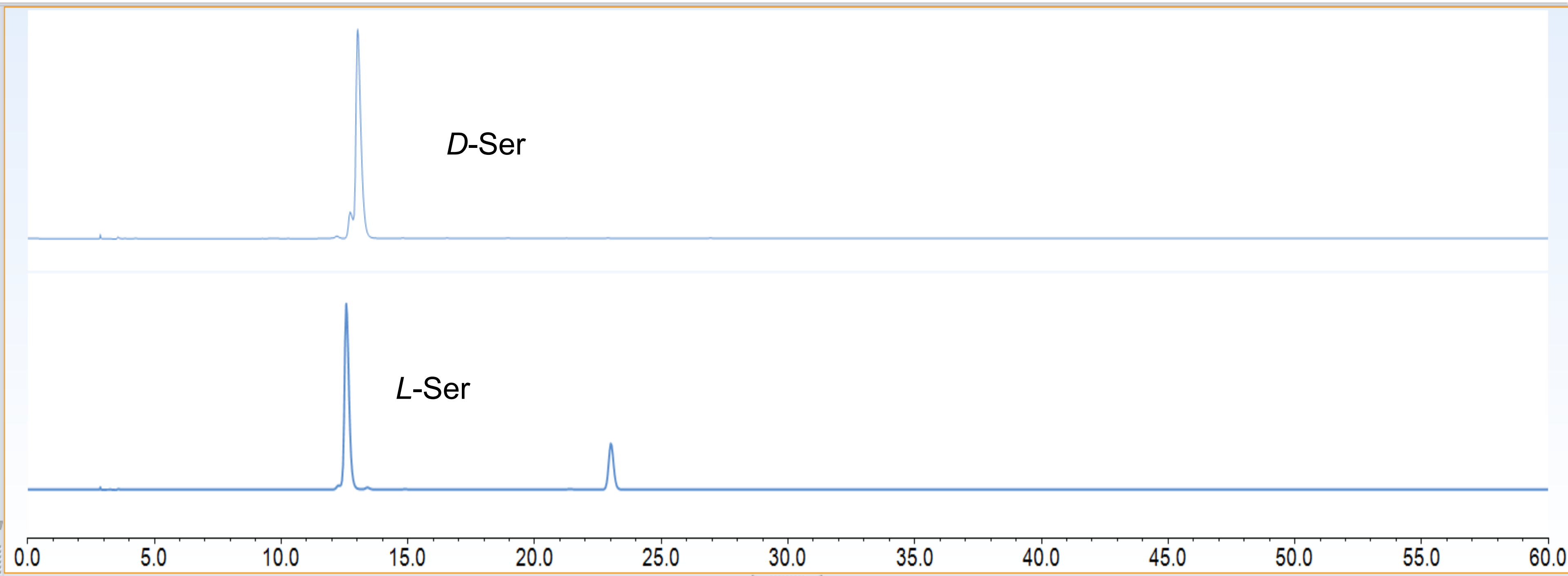

Figure S34. Marfey's analysis of compound **3** and standard amino acids

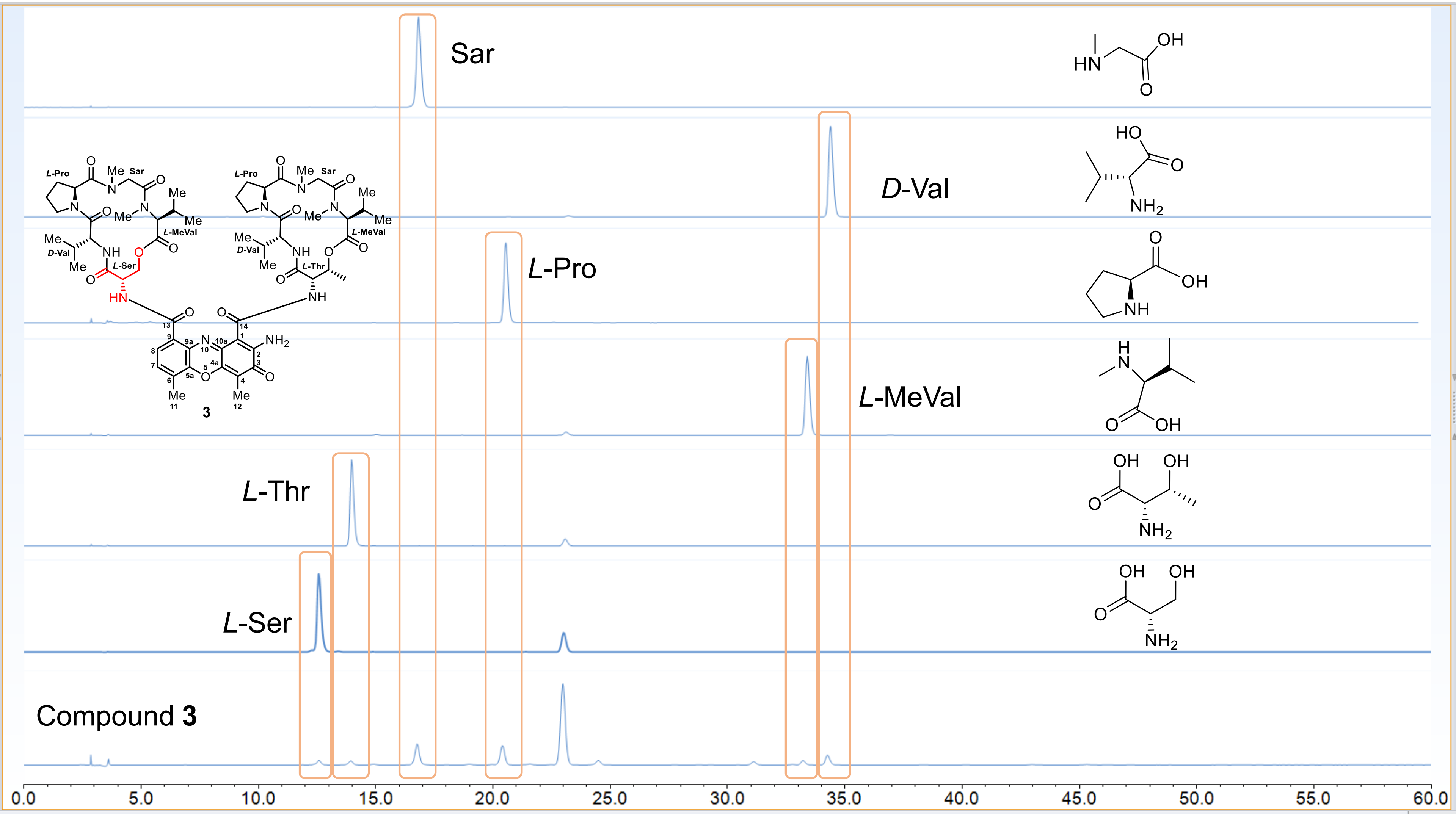

Figure S35. UV spectrum of compound **4**

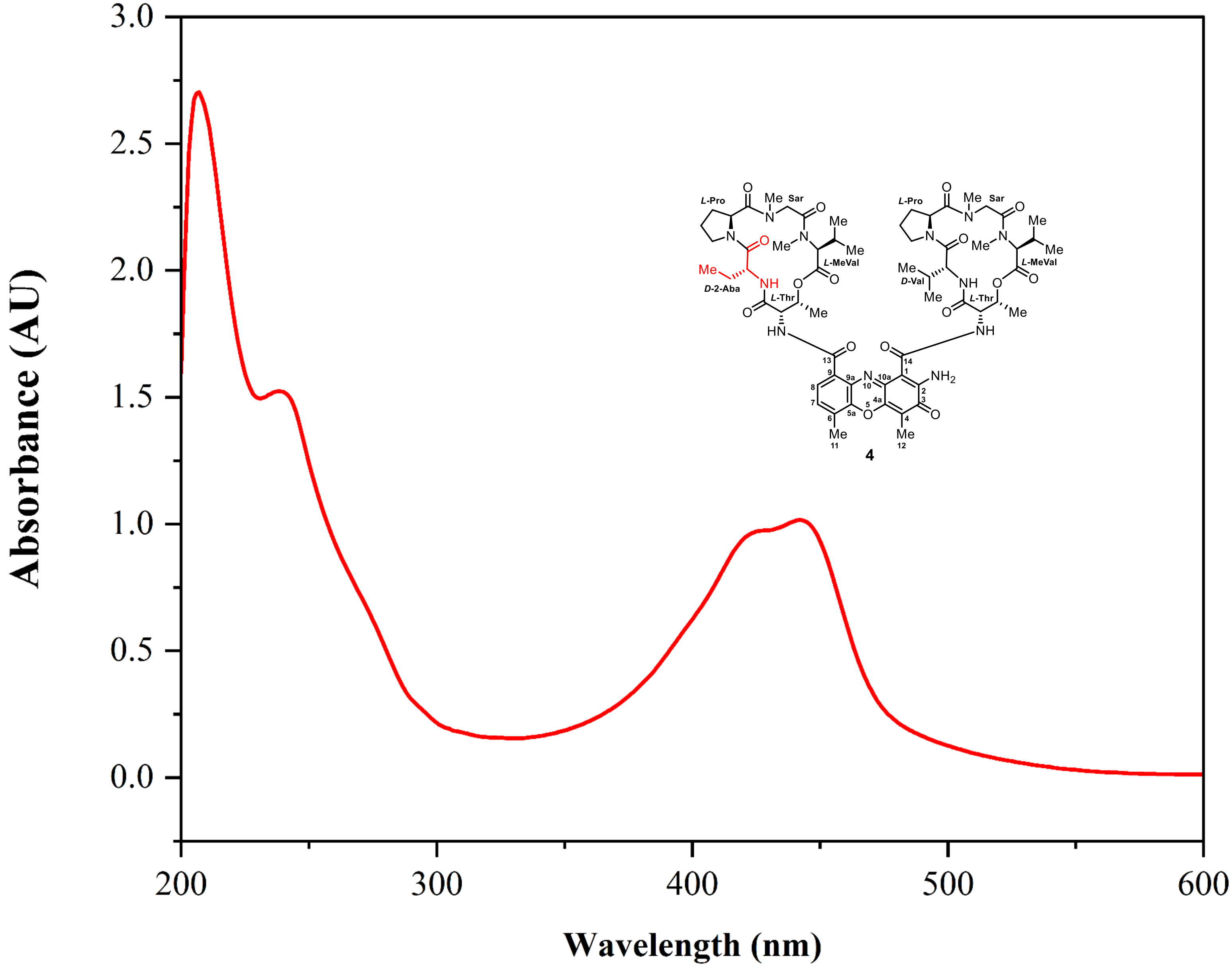

Figure S36. HR-ESIMS for compound **4**

Mass Spectrum SmartFormula Report

Analysis Info

Analysis Name	D:\Data\MS\data\202505\panghe_S-11-1_pos_33_01_19978.d	Acquisition Date	5/29/2025 5:40:19 PM
Method	LC_Direct Infusion_pos_100-1000mz.m	Operator	SCSIO
Sample Name	panghe_S-11-1_pos	Instrument	maXis 255552.00029
Comment			

Acquisition Parameter

Source Type	ESI	Ion Polarity	Positive	Set Nebulizer	0.4 Bar
Focus	Active	Set Capillary	4500 V	Set Dry Heater	180 °C
Scan Begin	100 m/z	Set End Plate Offset	-500 V	Set Dry Gas	4.0 l/min
Scan End	2000 m/z	Set Charging Voltage	0 V	Set Divert Valve	Waste
		Set Corona	0 nA	Set APCI Heater	0 °C

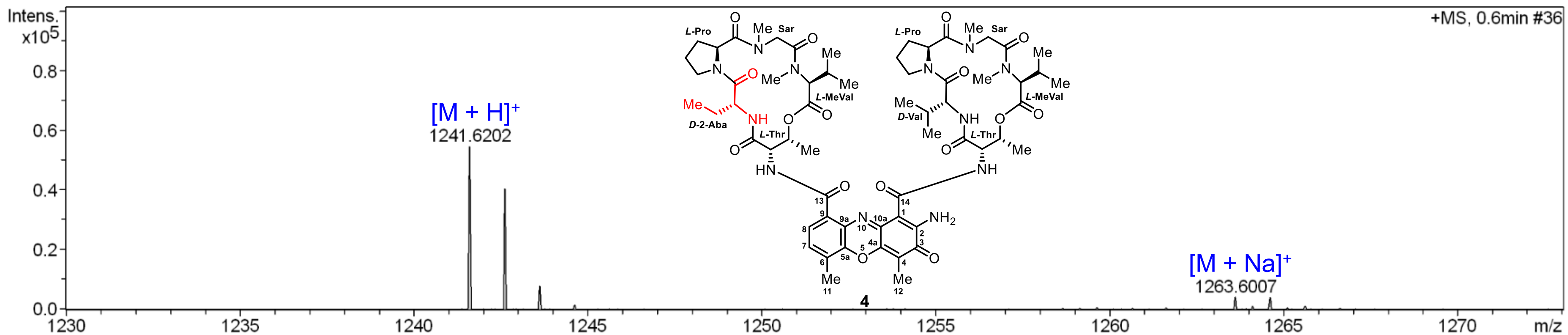

Meas. m/z	#	Ion Formula	Score	m/z	err [ppm]	err [mDa]	mSigma	rdb	e¯ Conf	N-Rule
1241.6202	1	C61H85N12O16	100.00	1241.6201	0.0	0.1	63.3	25.5	even	ok
1263.6007	1	C61H84N12NaO16	100.00	1263.6020	-1.1	-1.3	105.5	25.5	even	ok

Figure S37. ^{1}H NMR (600 MHz) spectrum of compound **4** in $CDCl_3$

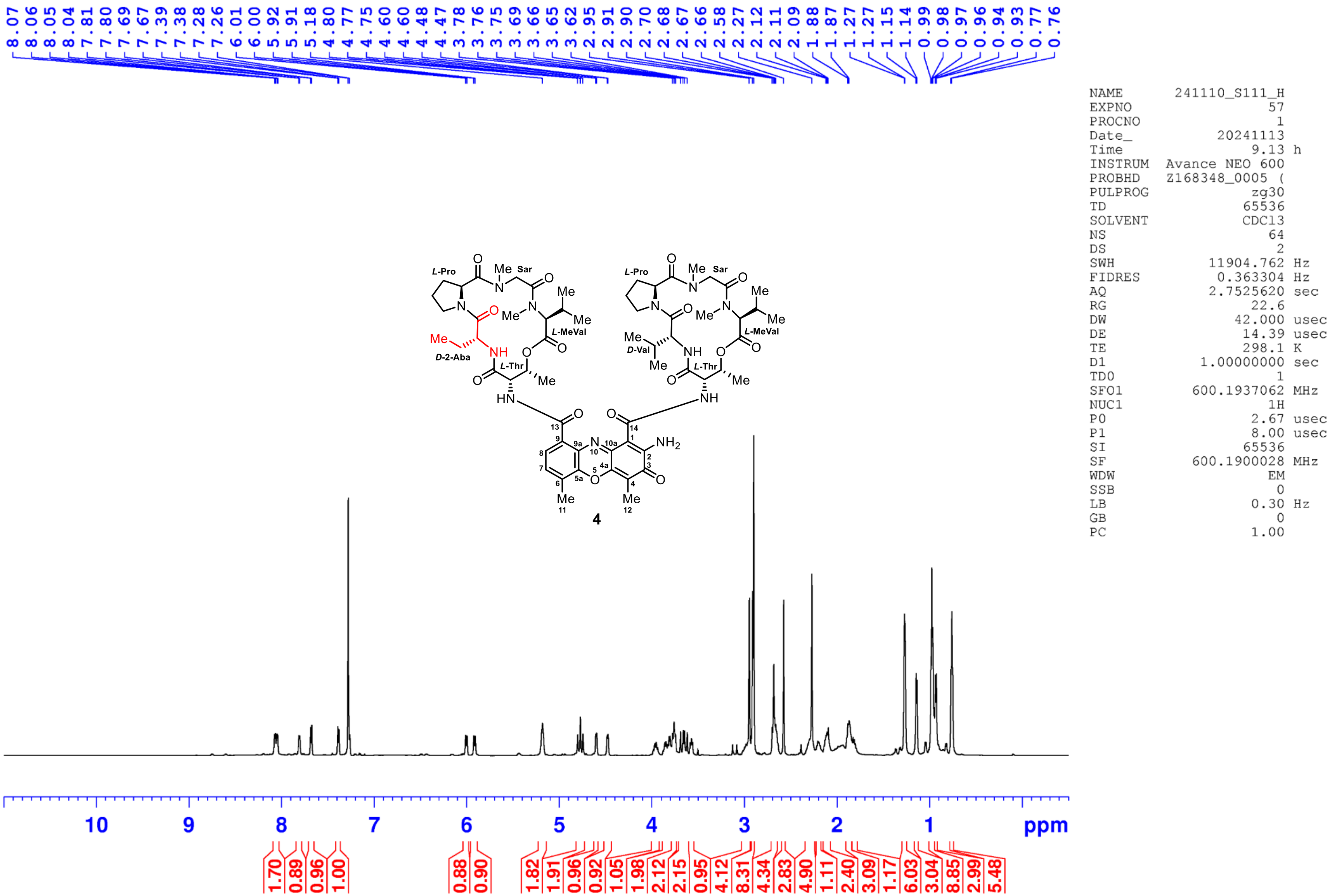

Figure S37-continue

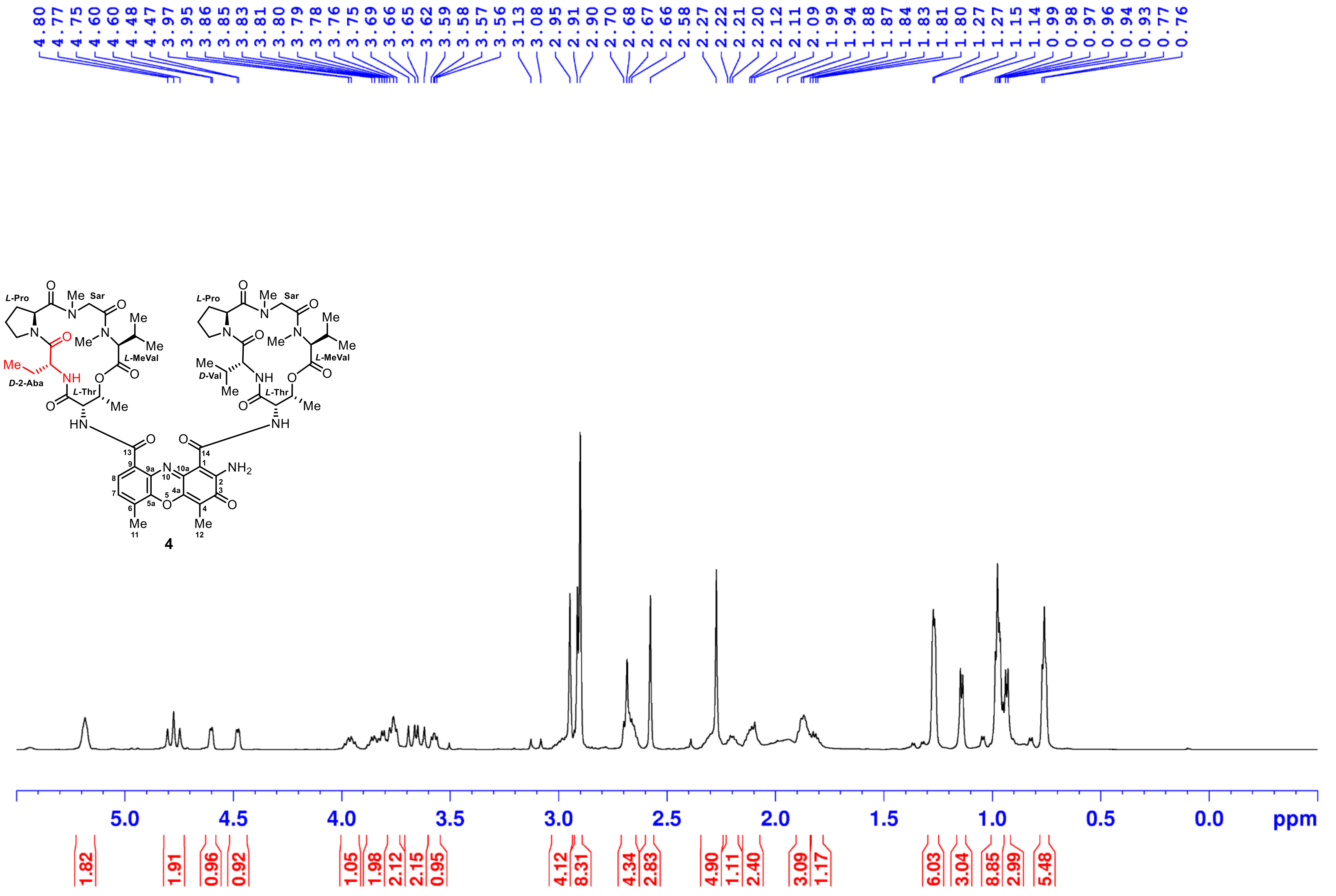

Figure S37-continue

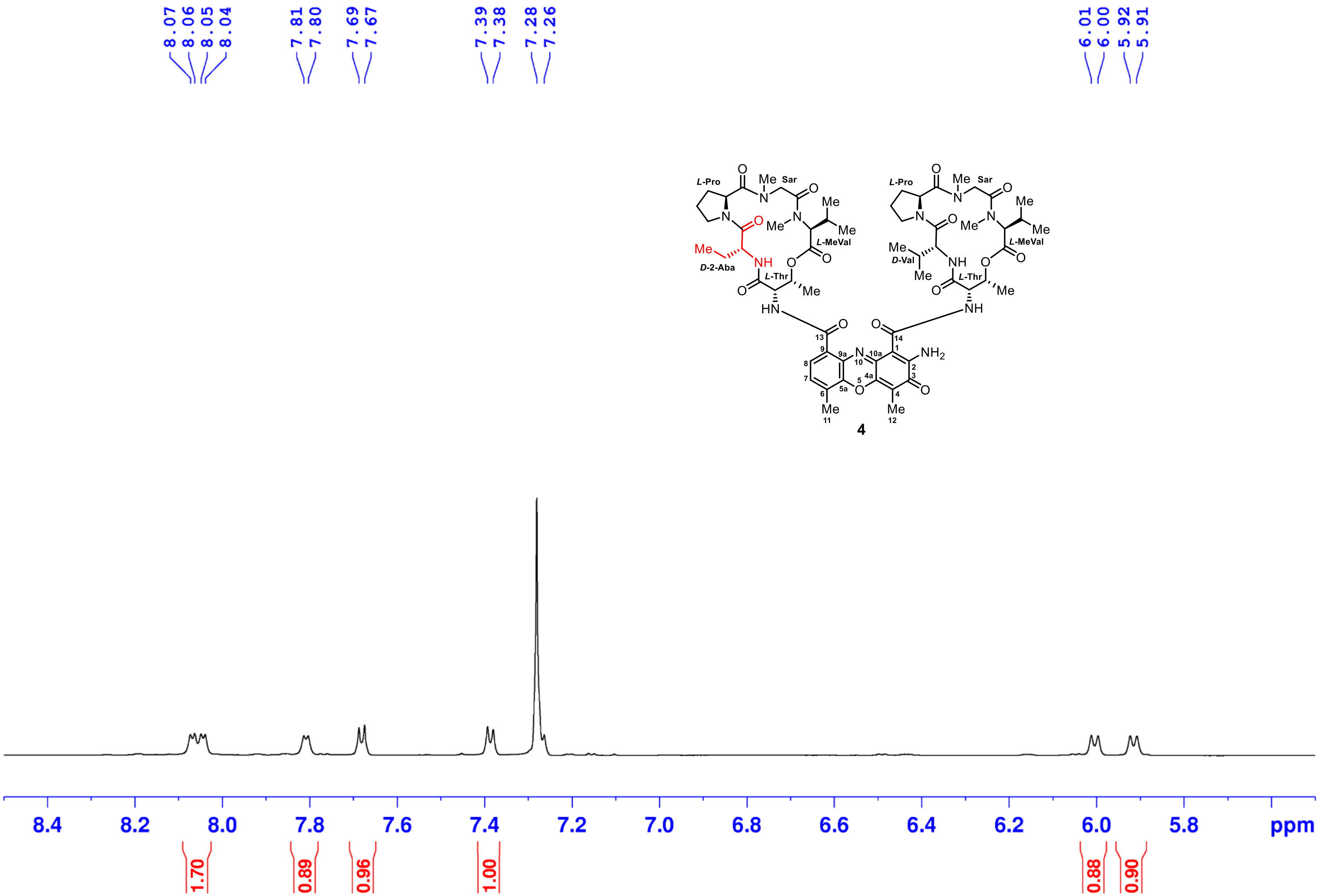

Figure S38. ^{13}C NMR (150 MHz) spectrum of compound **4** in $CDCl_3$

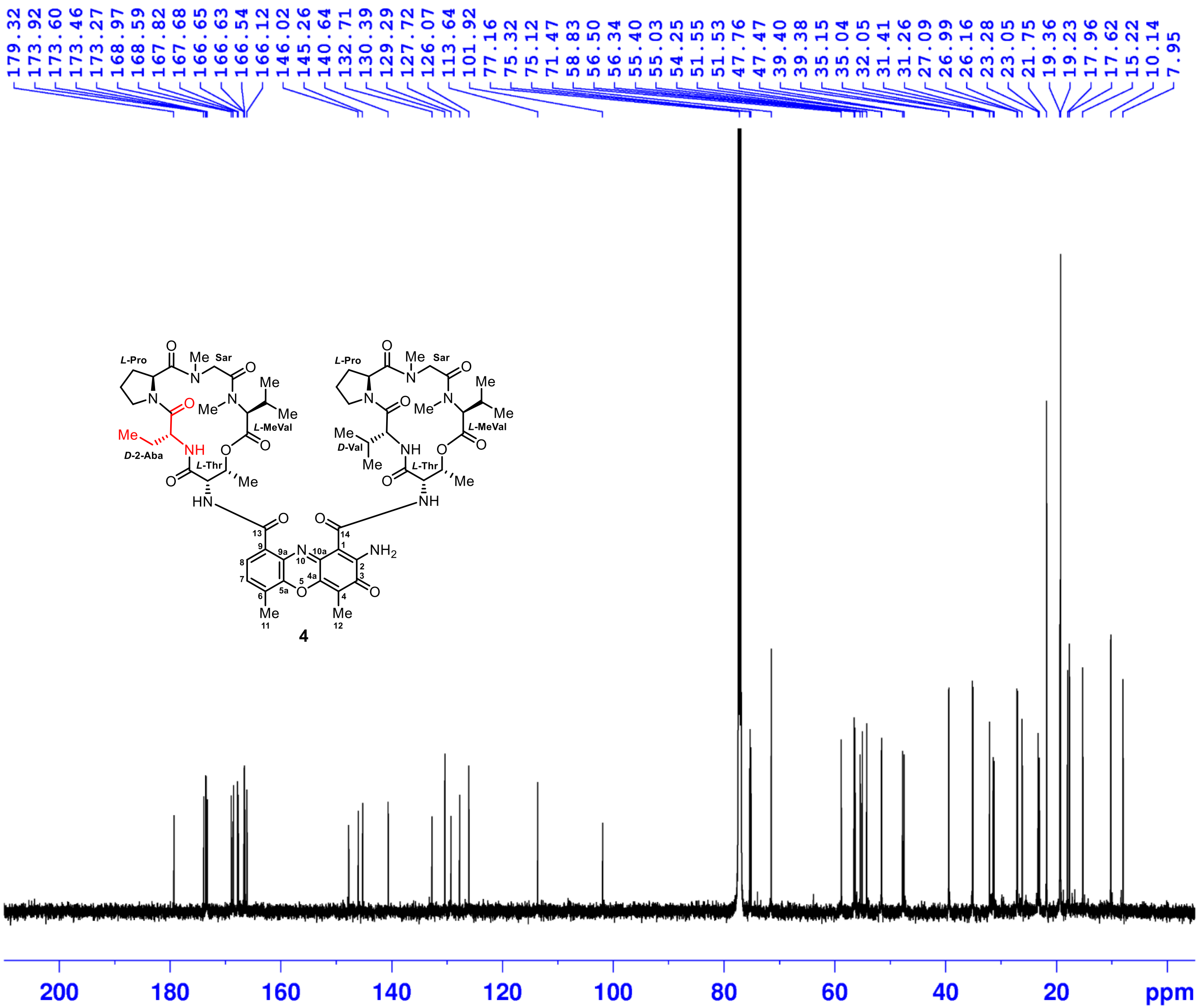

NAME	241110_S111_C
EXPNO	58
PROCNO	1
Date_	20241113
Time	11.01 h
INSTRUM	Avance NEO 600
PROBHD	Z168348_0005 (
PULPROG	zgpg30
TD	35714
SOLVENT	CDCl3
NS	4096
DS	4
SWH	35714.285 Hz
FIDRES	2.000016 Hz
AQ	0.5000460 sec
RG	101
DW	14.000 usec
DE	18.00 usec
TE	298.2 K
D1	1.00000000 sec
D11	0.03000000 sec
TD0	1
SFO1	150.9329873 MHz
NUC1	13C
P0	4.00 usec
P1	12.00 usec
SI	32768
SF	150.9178749 MHz
WDW	EM
SSB	0
LB	1.00 Hz
GB	0
PC	1.40

Figure S38-continue

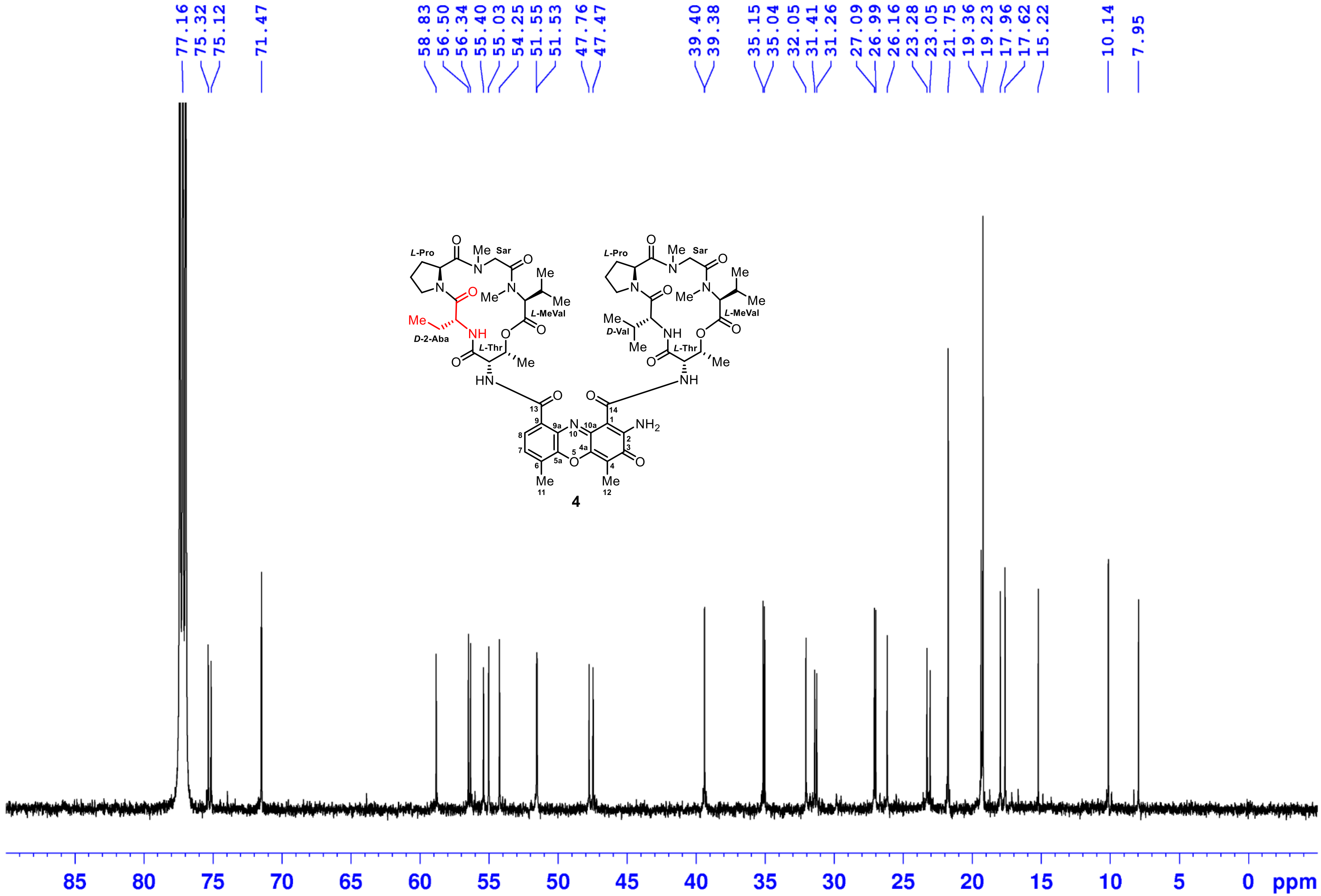

Figure S38-continue

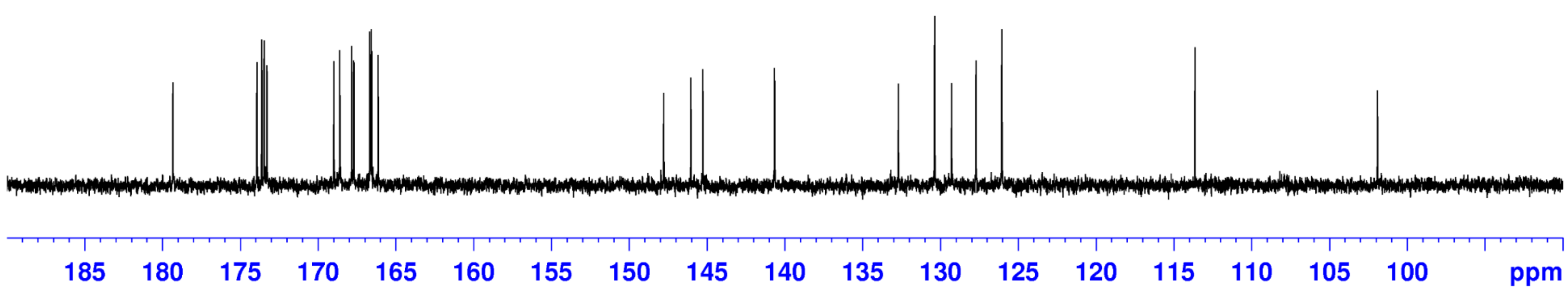

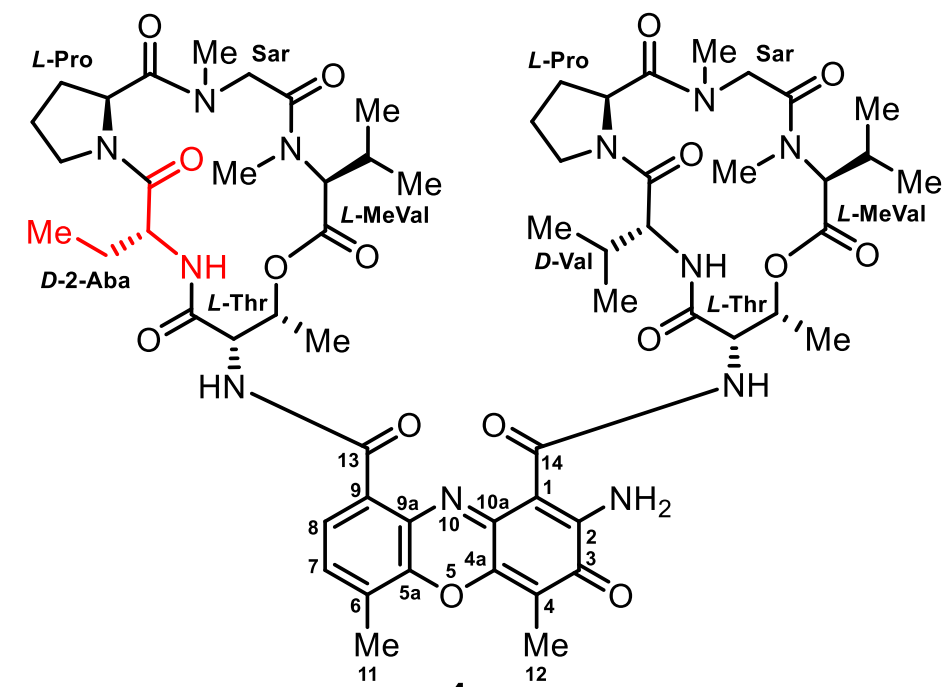

4

Figure S39. DEPT135 (150 MHz) spectrum of compound **4** in $CDCl_3$

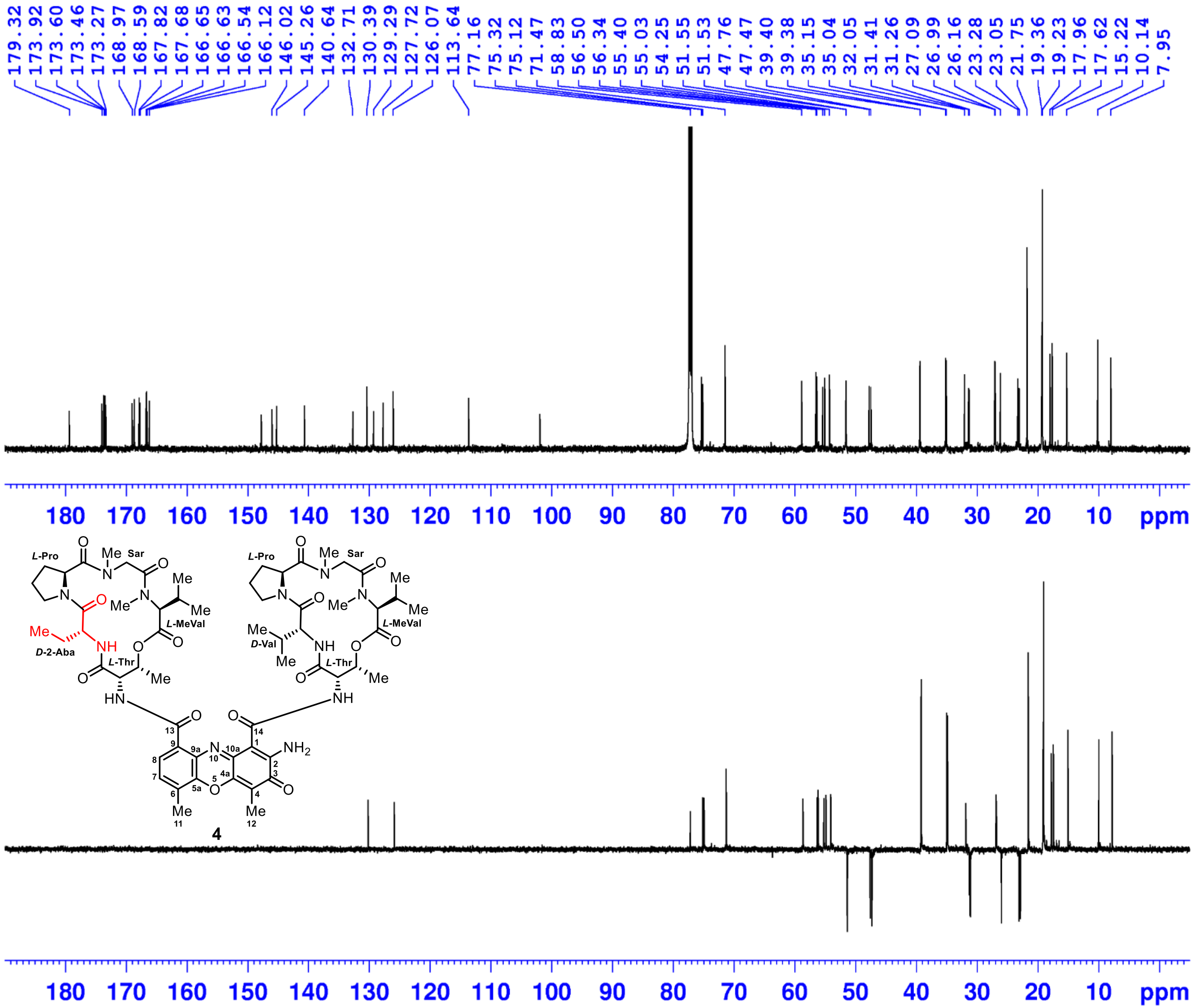

NAME	241110_S111_135
EXPNO	61
PROCNO	1
Date_	20241113
Time	15.52 h
INSTRUM	Avance NEO 600
PROBHD	Z168348_0005 (
PULPROG	deptsp135
TD	65536
SOLVENT	CDCl3
NS	2048
DS	8
SWH	41666.668 Hz
FIDRES	1.271566 Hz
AQ	0.7864820 sec
RG	101
DW	12.000 usec
DE	18.00 usec
TE	298.1 K
CNST2	145.0000000
D1	2.00000000 sec
D2	0.00344828 sec
D12	0.00002000 sec
TD0	1
SFO1	150.9299690 MHz
NUC1	13C
P1	12.00 usec
P13	2000.00 usec
SI	32768
SF	150.9179048 MHz
WDW	EM
SSB	0
LB	1.00 Hz
GB	0
PC	1.40

Figure S39-continue

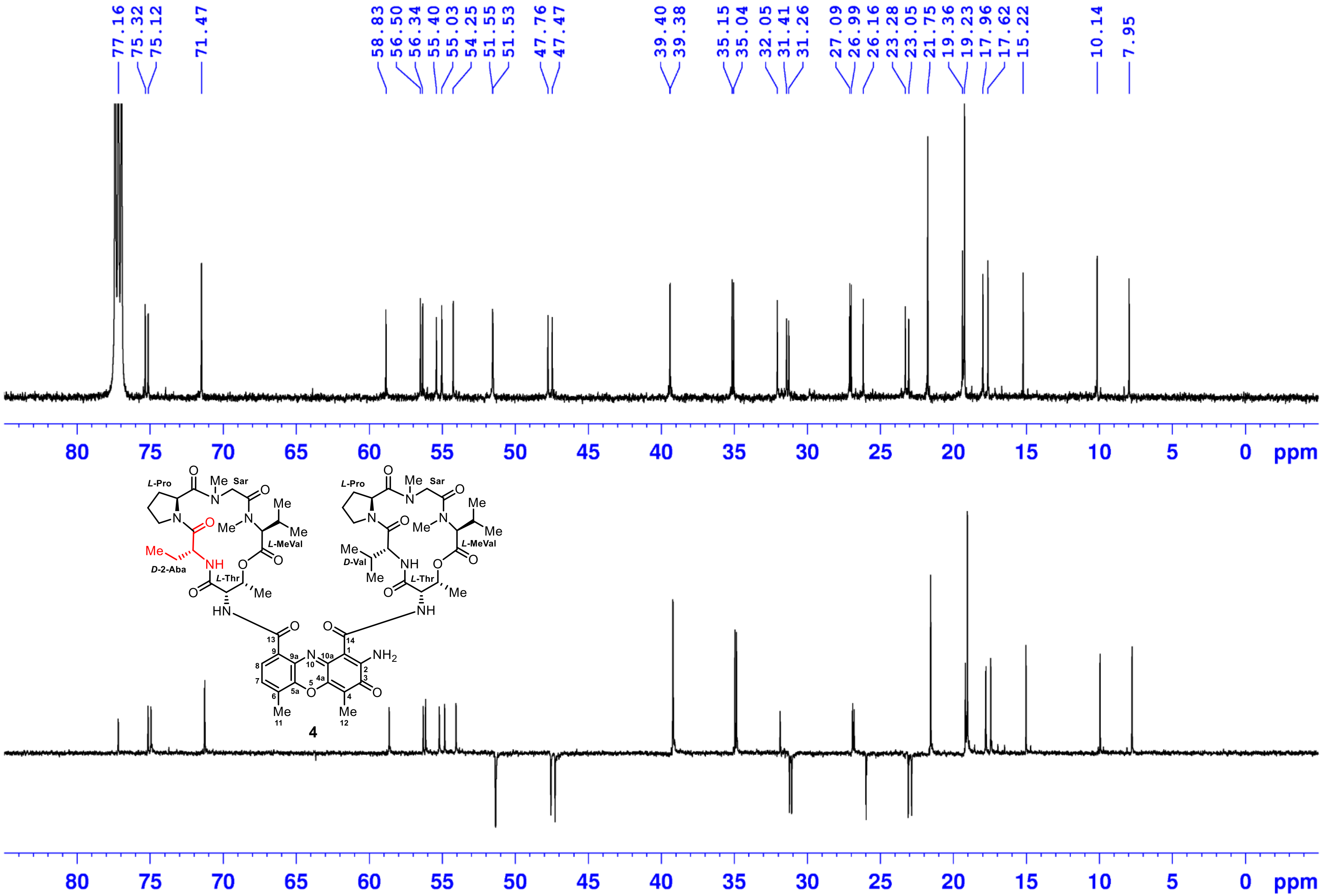

Figure S39-continue

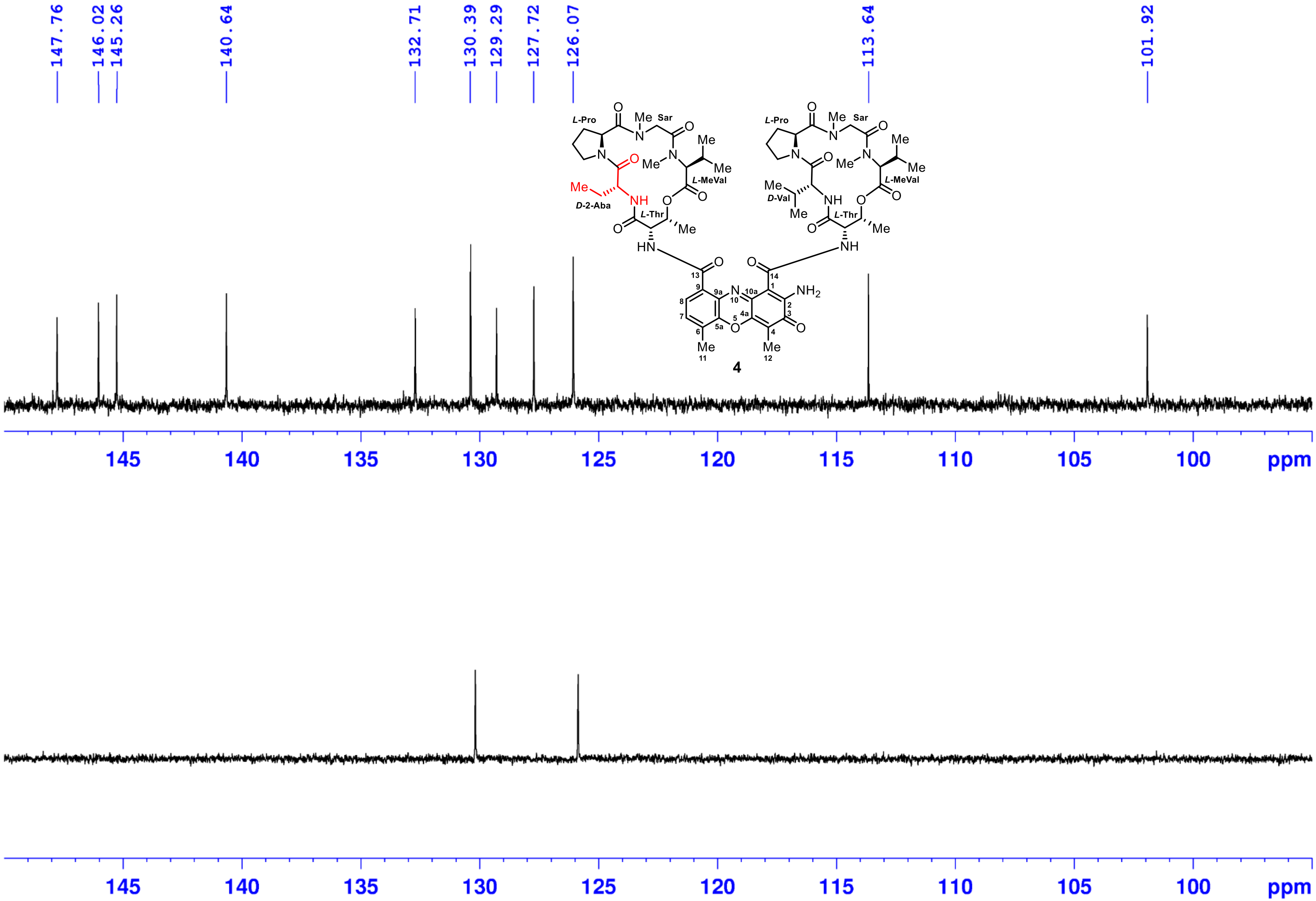

Figure S40. ^1H–^1H COSY (600 MHz) spectrum of compound **4** in $CDCl_3$

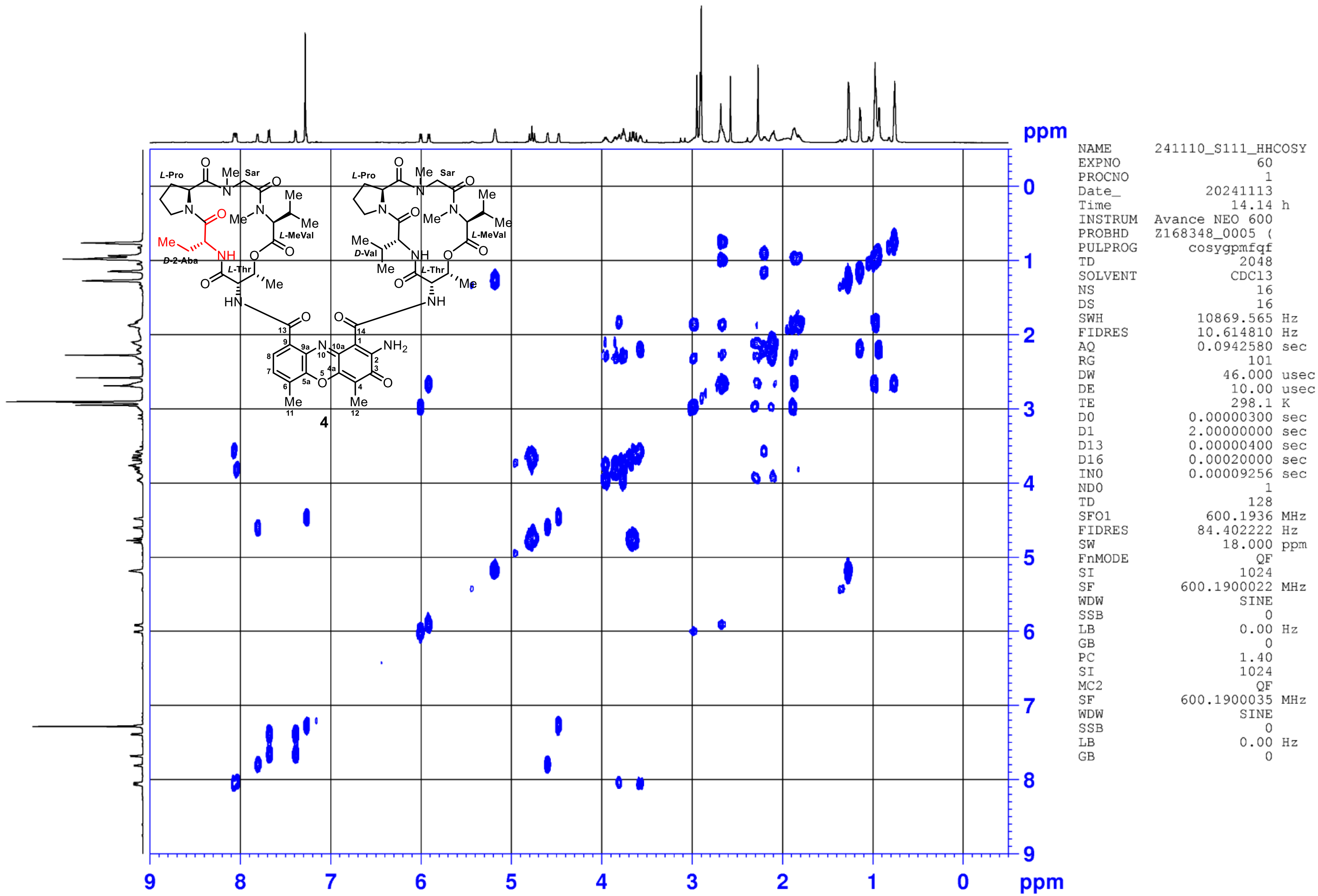

Figure S40-continue

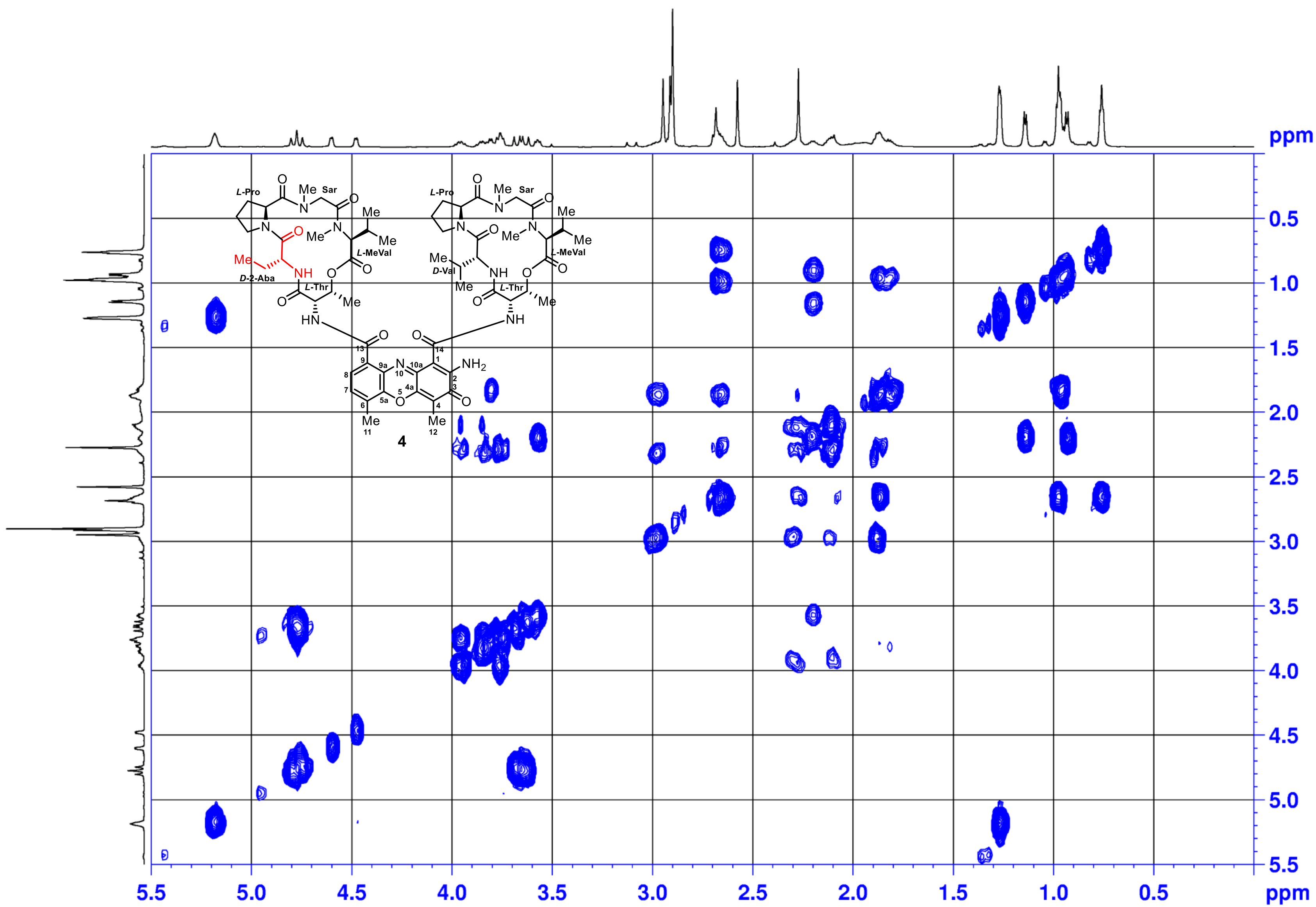

Figure S40-continue

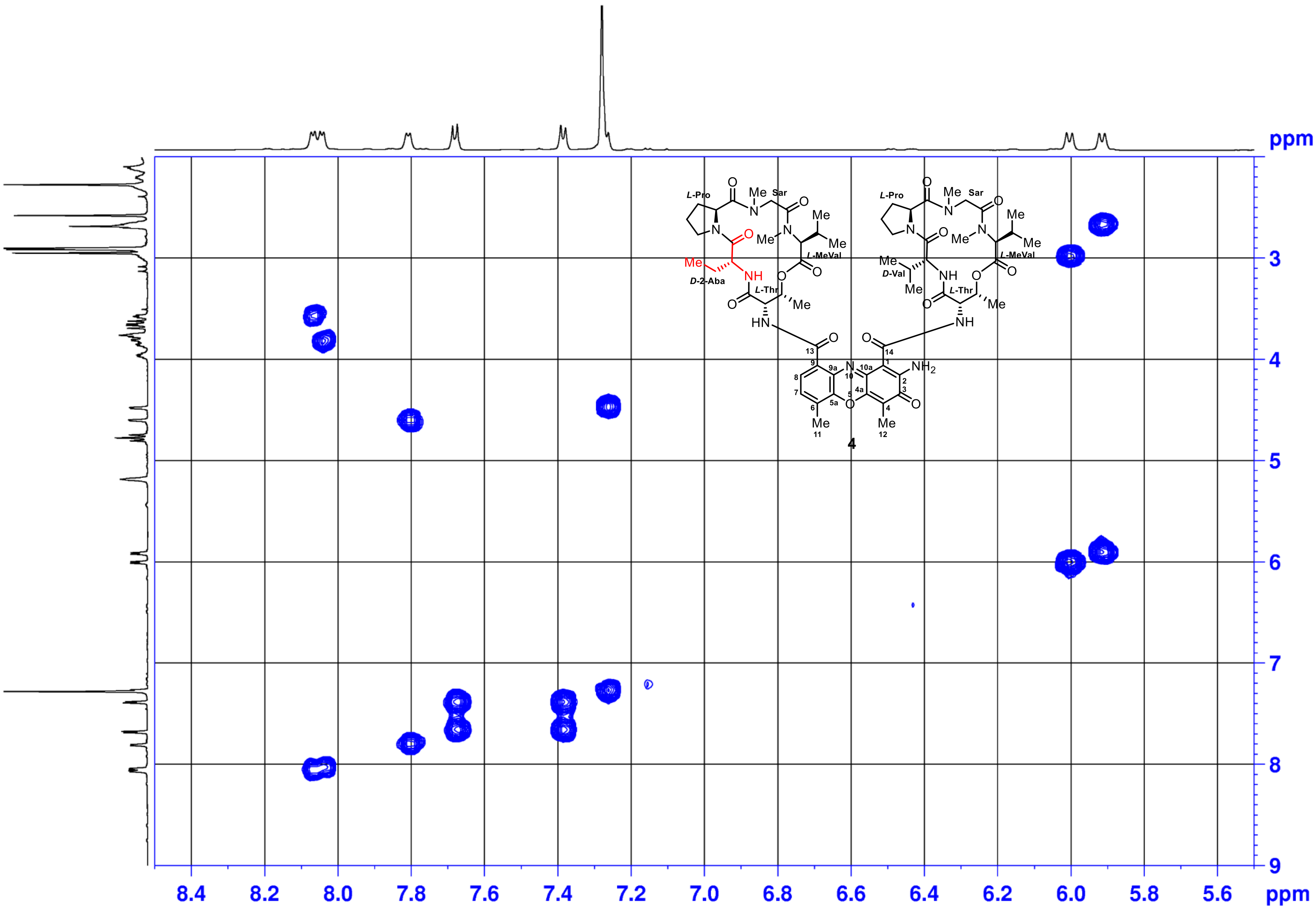

Figure S41. HSQC (600 MHz) spectrum of compound **14** in $CDCl_3$

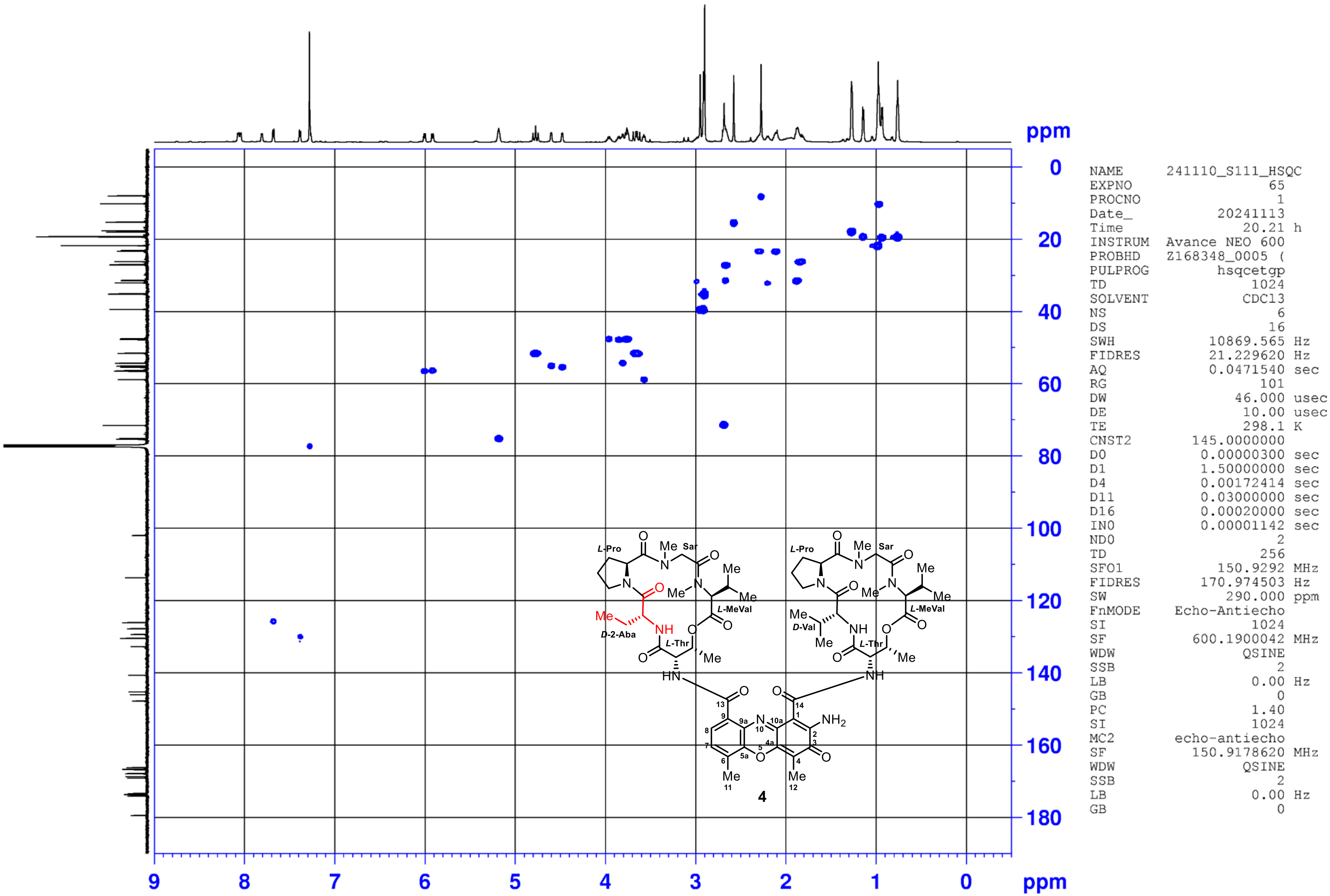

Figure S41-continue

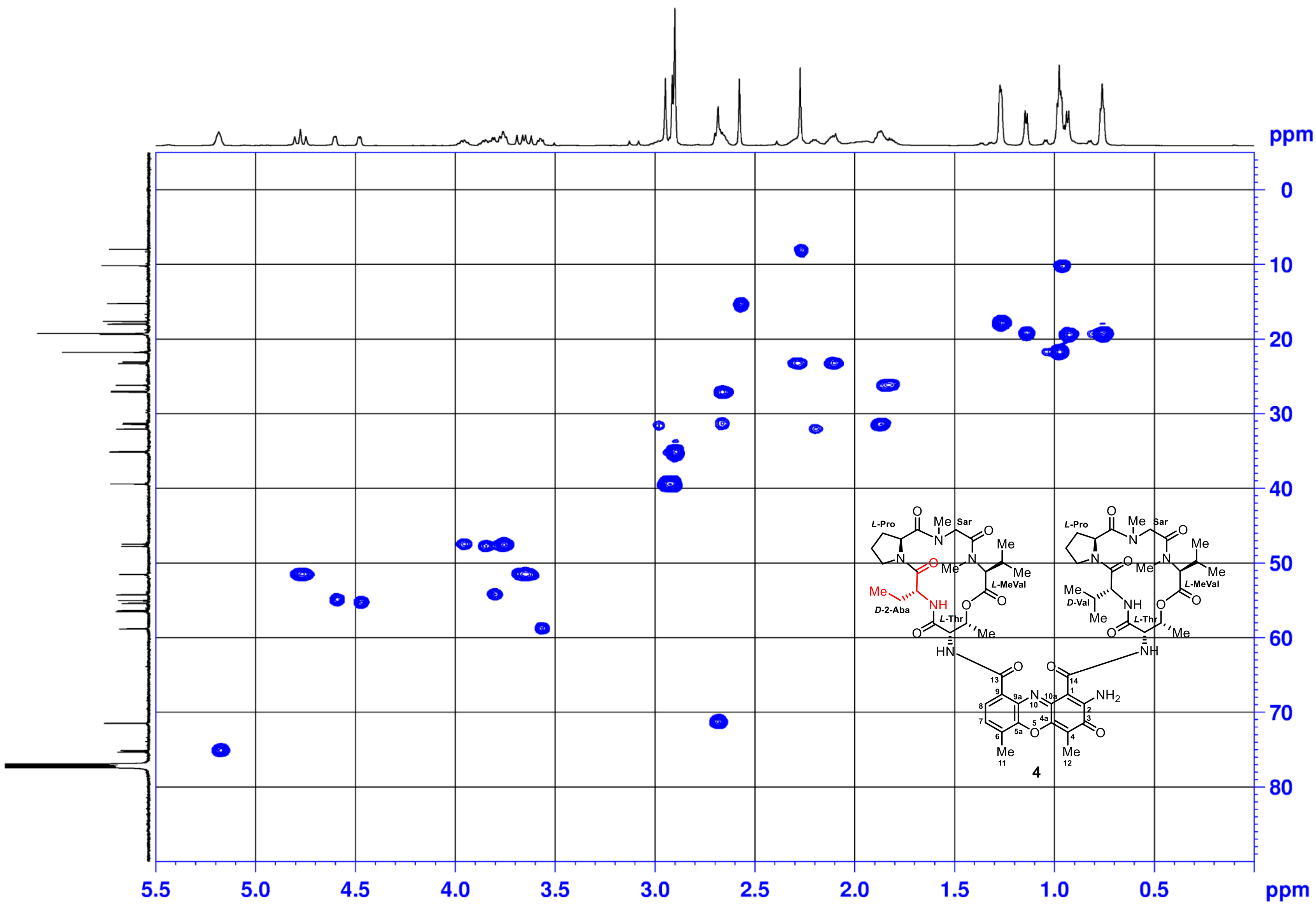

Figure S41-continue

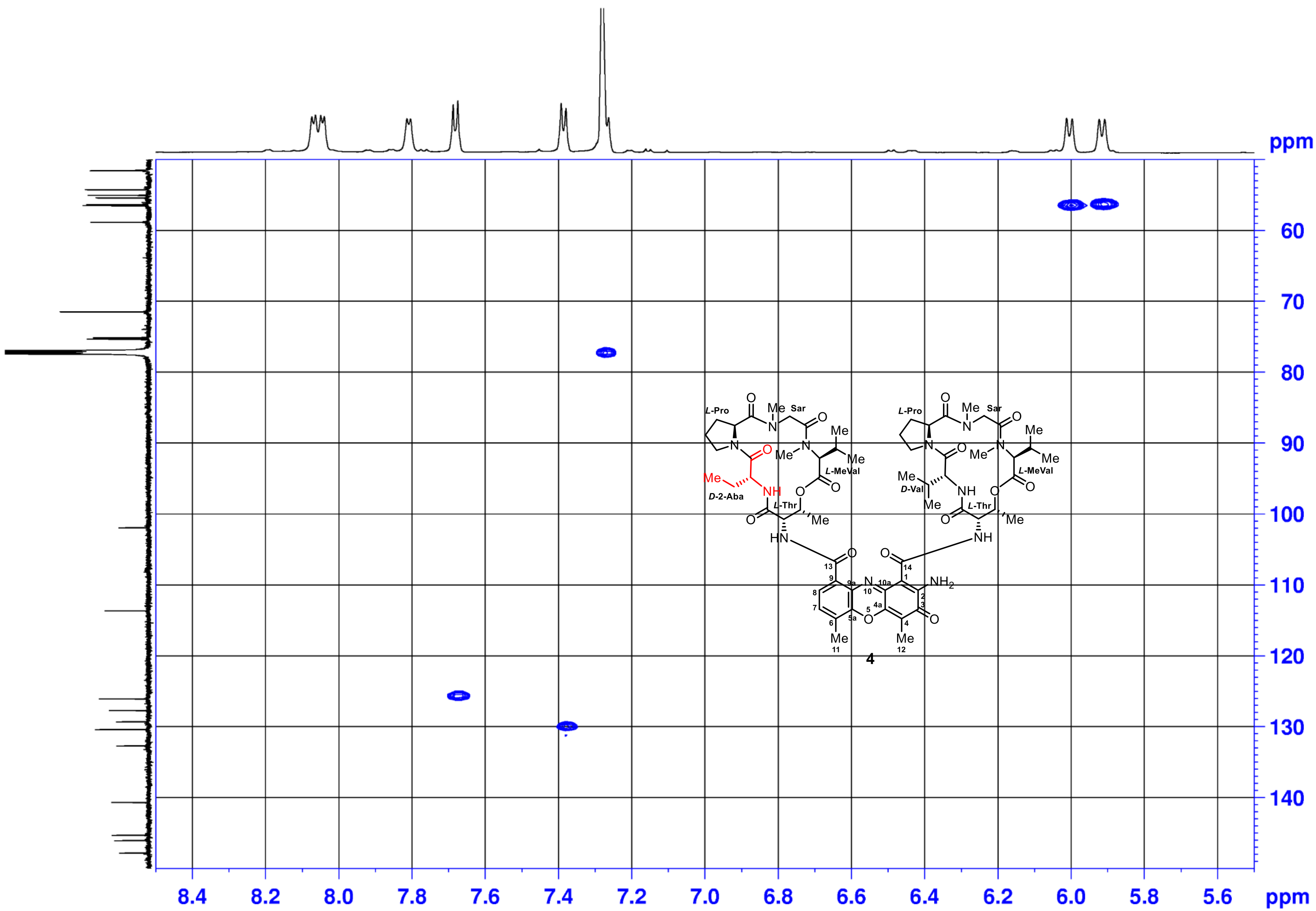

Figure S42. HMBC (600 MHz) spectrum of compound **4** in $CDCl_3$

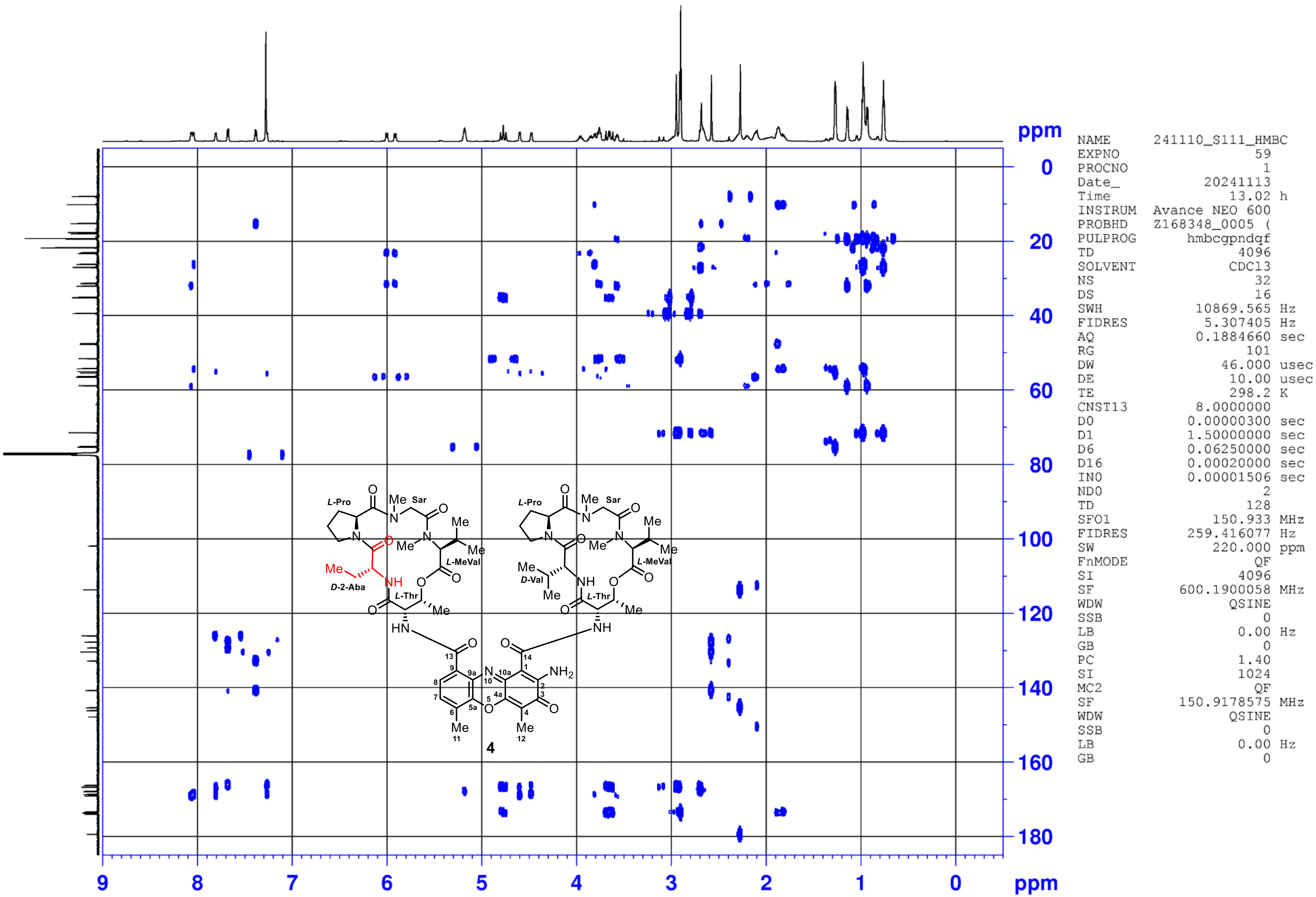

Figure S42-continue

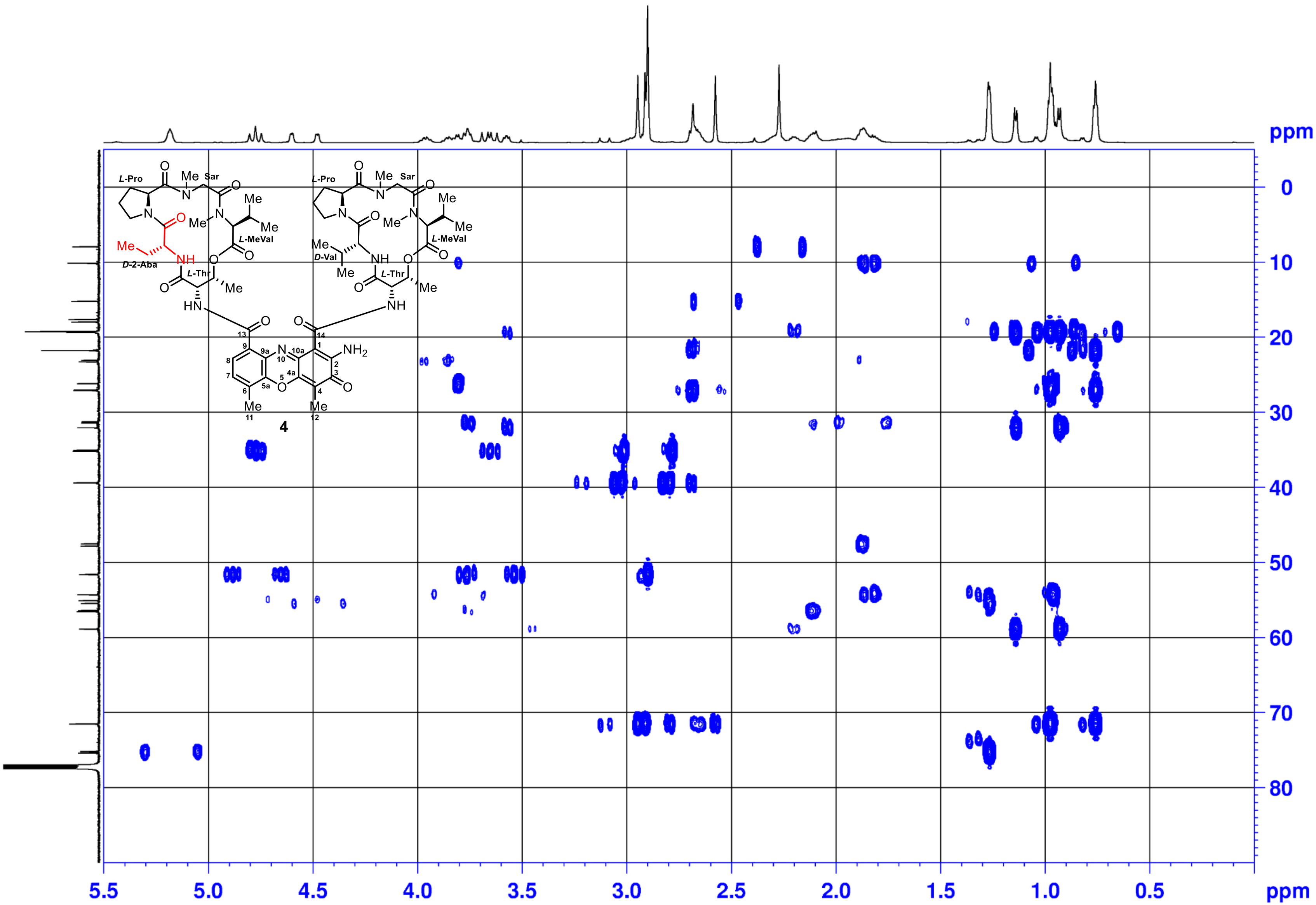

Figure S42-continue

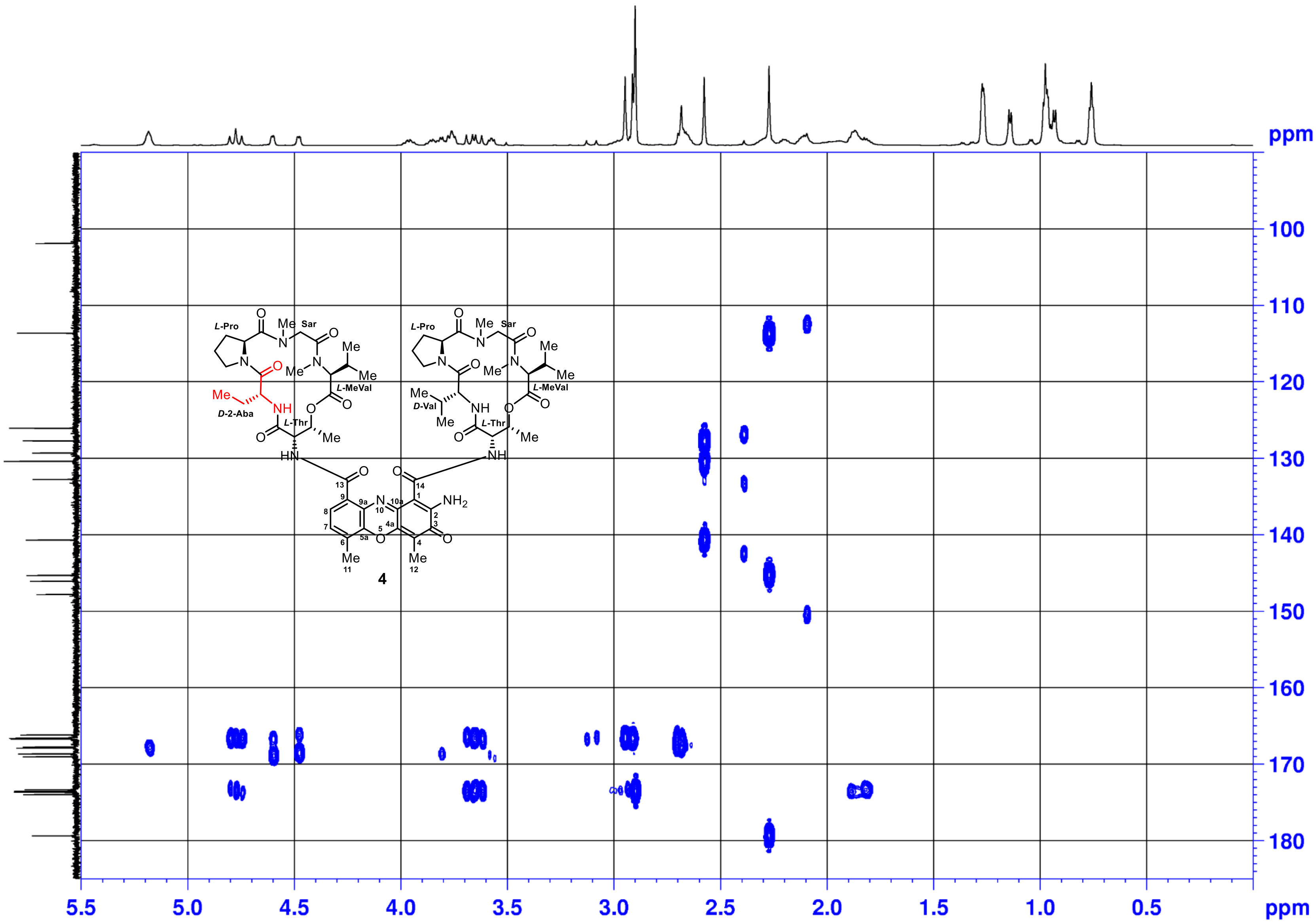

Figure S42-continue

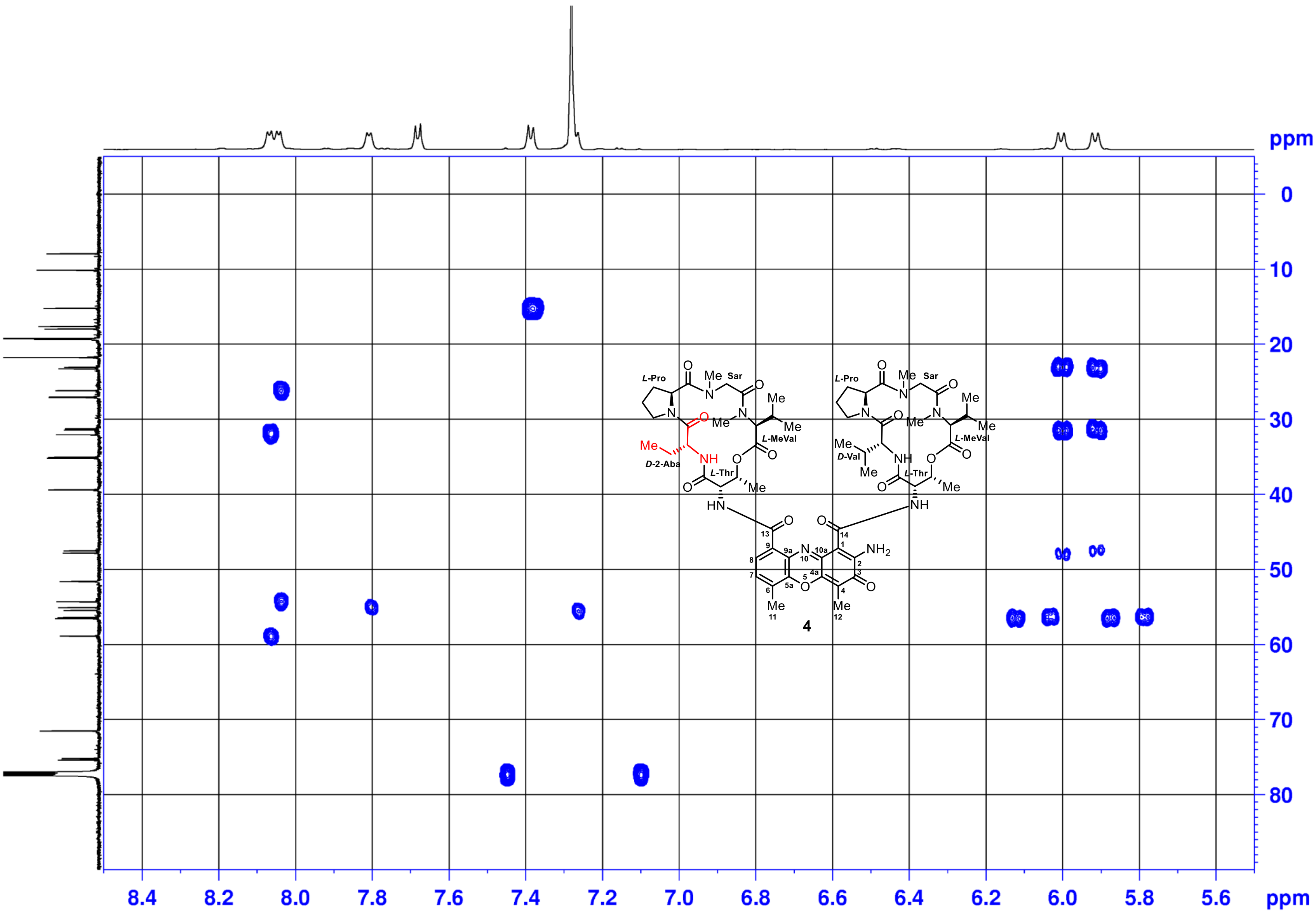

Figure S42-continue

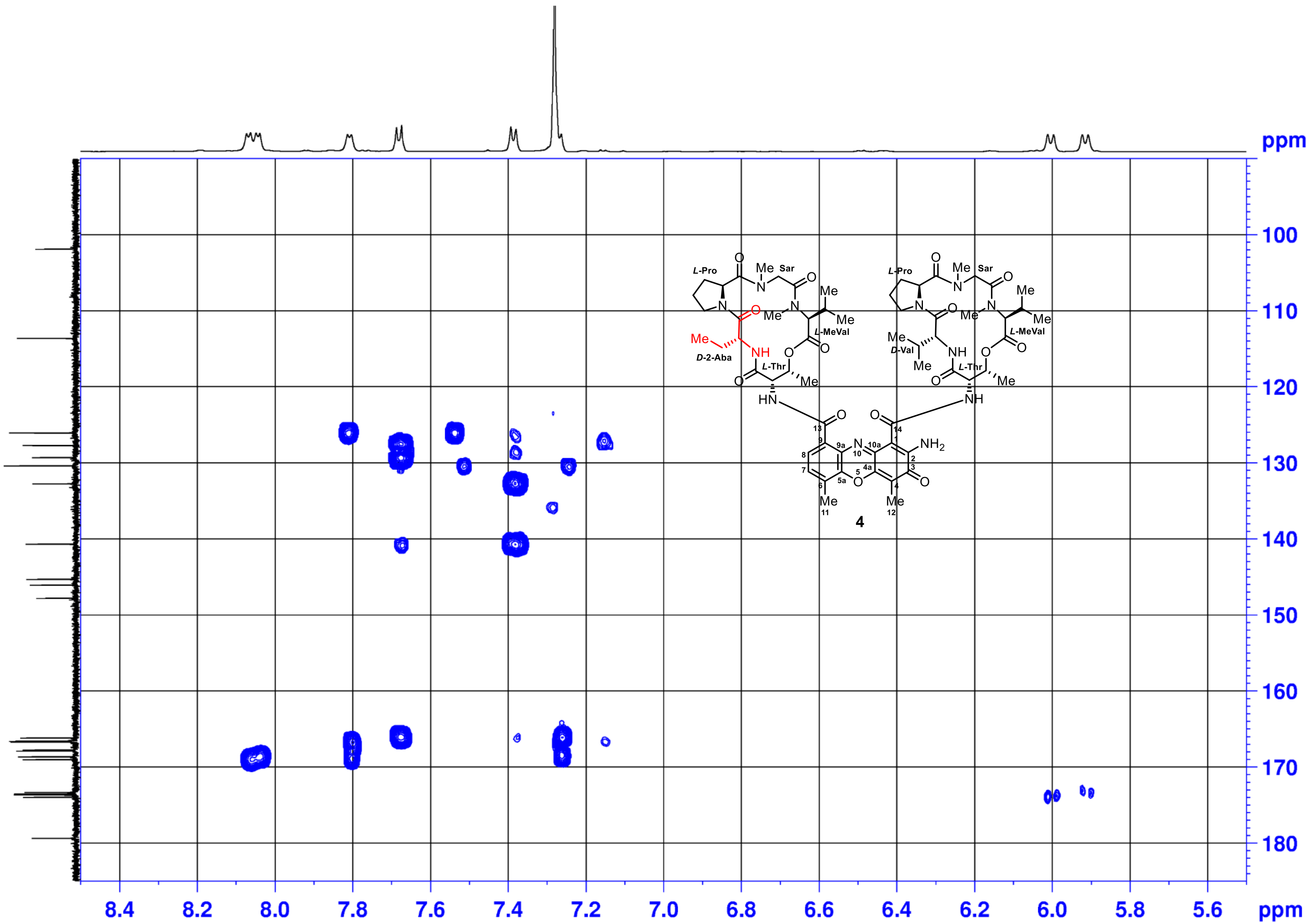

Figure S43. NOESY (600 MHz) spectrum of compound **4** in $CDCl_3$

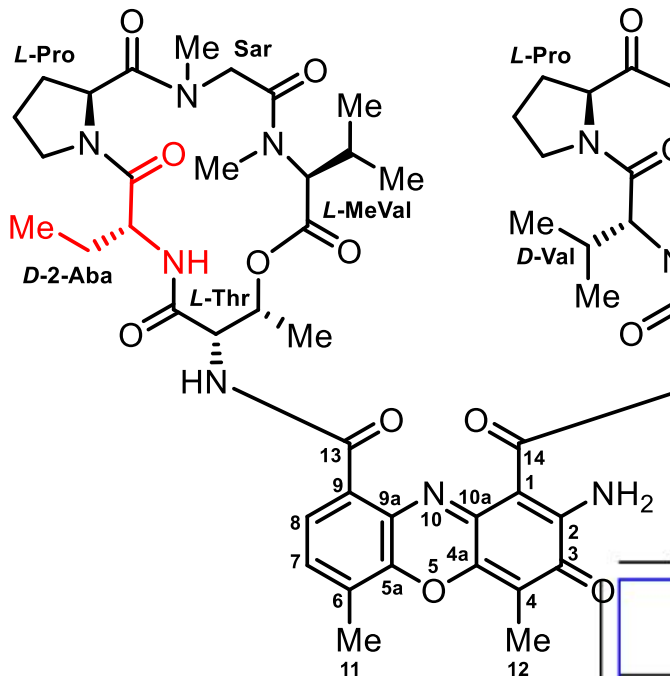

4

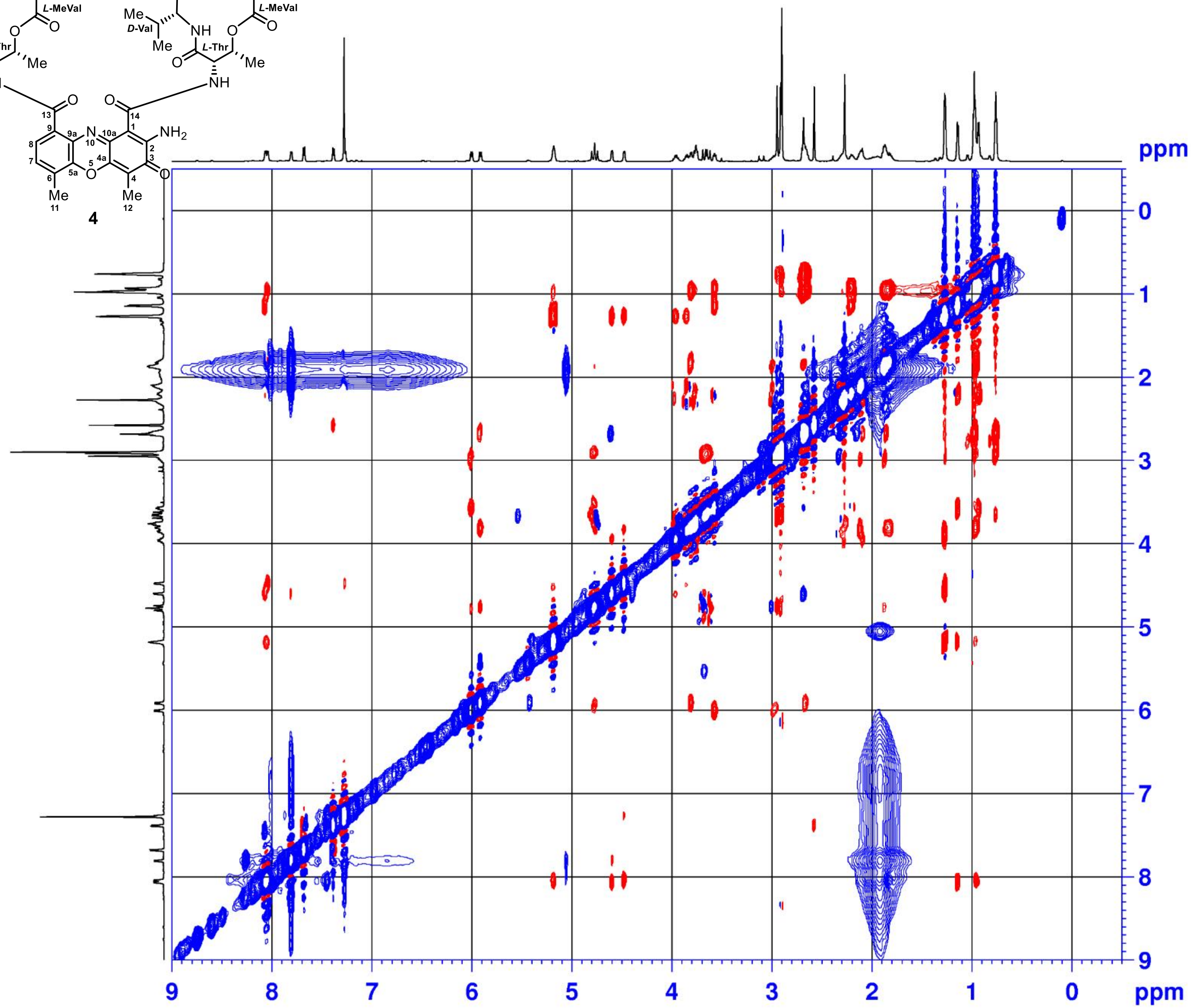

NAME	241110_S111_NOE
EXPNO	63
PROCNO	1
Date_	20241113
Time	19.40 h
INSTRUM	Avance NEO 600
PROBHD	Z168348_0005 (
PULPROG	noesygpphpp
TD	2048
SOLVENT	CDCl3
NS	12
DS	32
SWH	11904.762 Hz
FIDRES	11.625744 Hz
AQ	0.0860660 sec
RG	64
DW	42.000 usec
DE	10.66 usec
TE	298.1 K
D0	0.00003147 sec
D1	2.00000000 sec
D8	0.30000001 sec
D11	0.03000000 sec
D12	0.00002000 sec
D16	0.00020000 sec
IN0	0.00008331 sec
ND0	1
TD	256
SFO1	600.1928 MHz
FIDRES	46.890064 Hz
SW	20.000 ppm
FnMODE	States-TPPI
SI	1024
SF	600.1900003 MHz
WDW	QSINE
SSB	2
LB	0.00 Hz
GB	0
PC	1.00
SI	1024
MC2	States-TPPI
SF	600.1900023 MHz
WDW	QSINE
SSB	2
LB	0.00 Hz
GB	0

Figure S43-continue

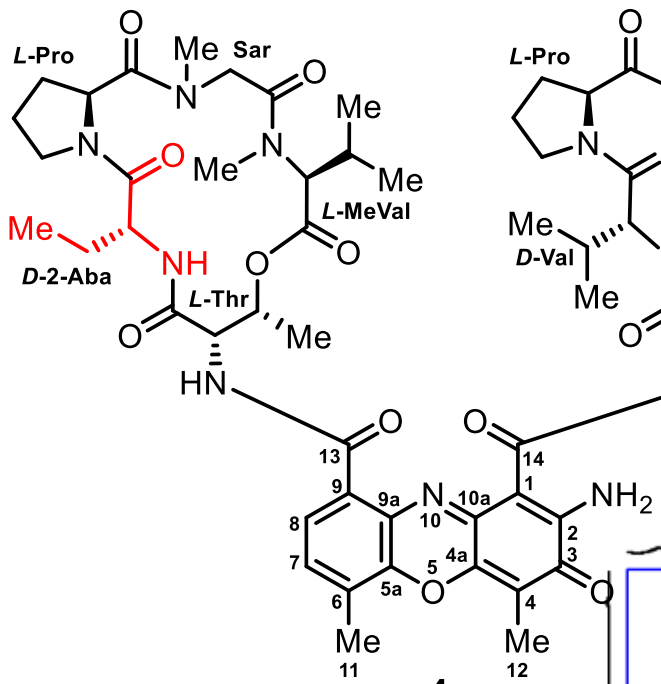

4

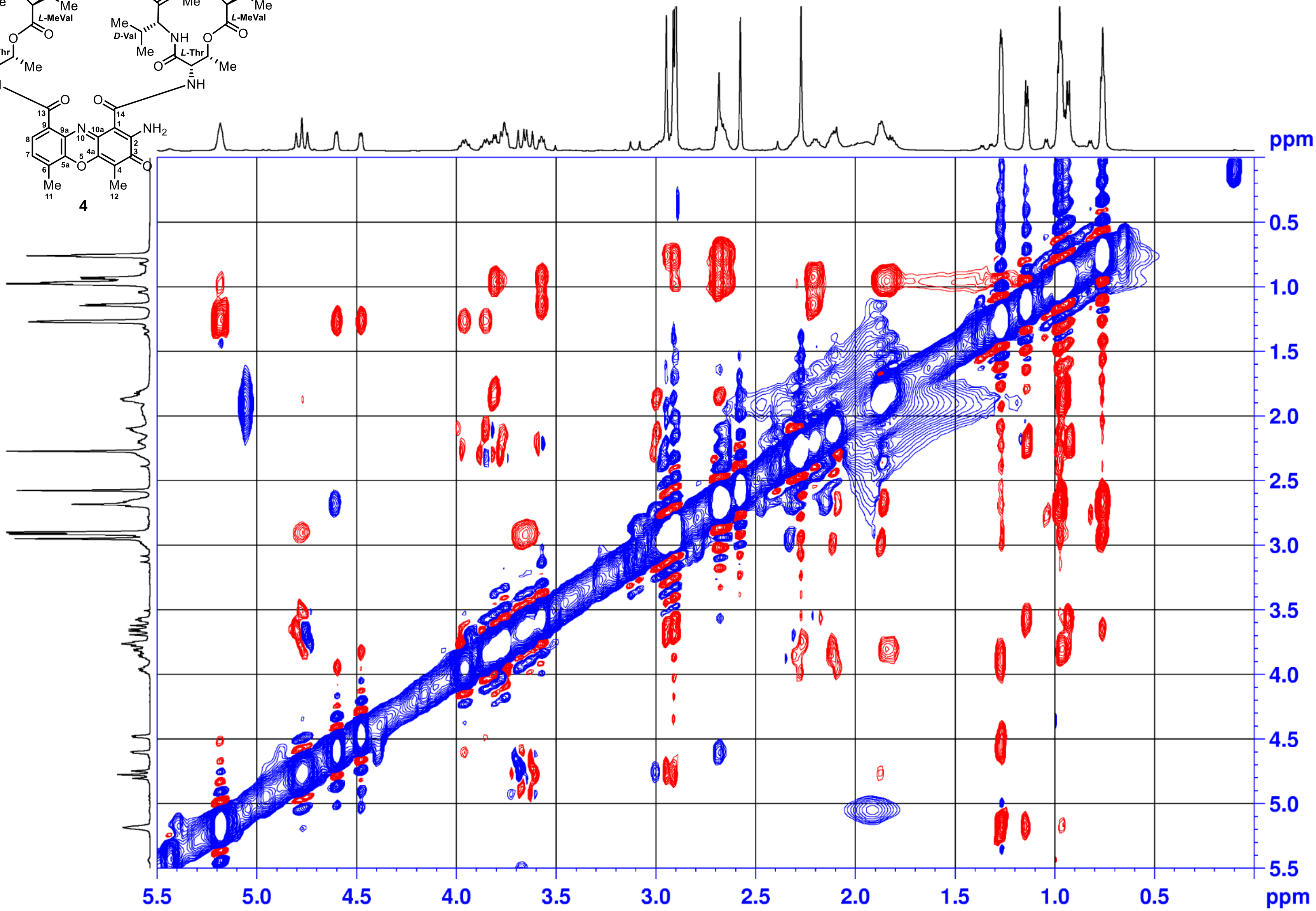

Figure S43-continue

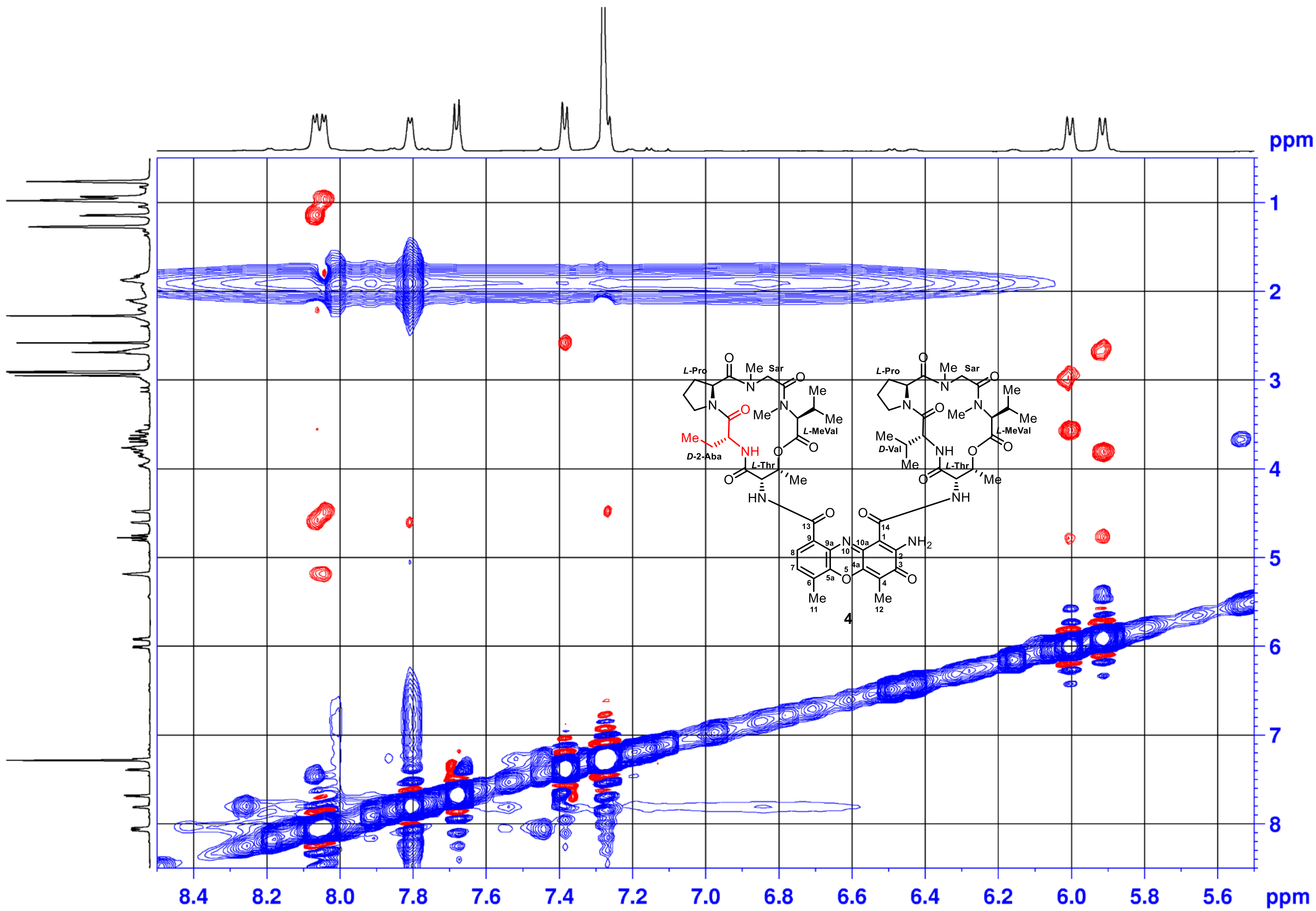

Figure S44. Marfey's analysis of 2-Aba standard

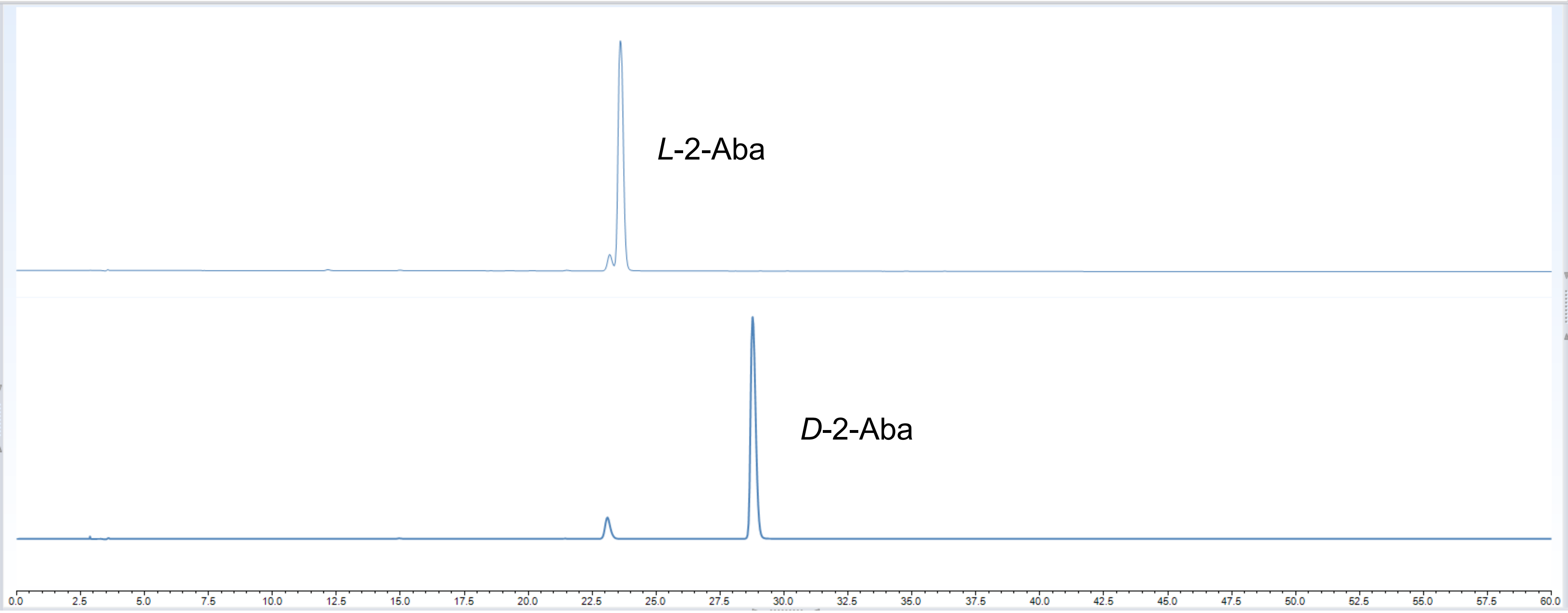

Figure S45. Marfey's analysis of compound **4** and standard amino acids

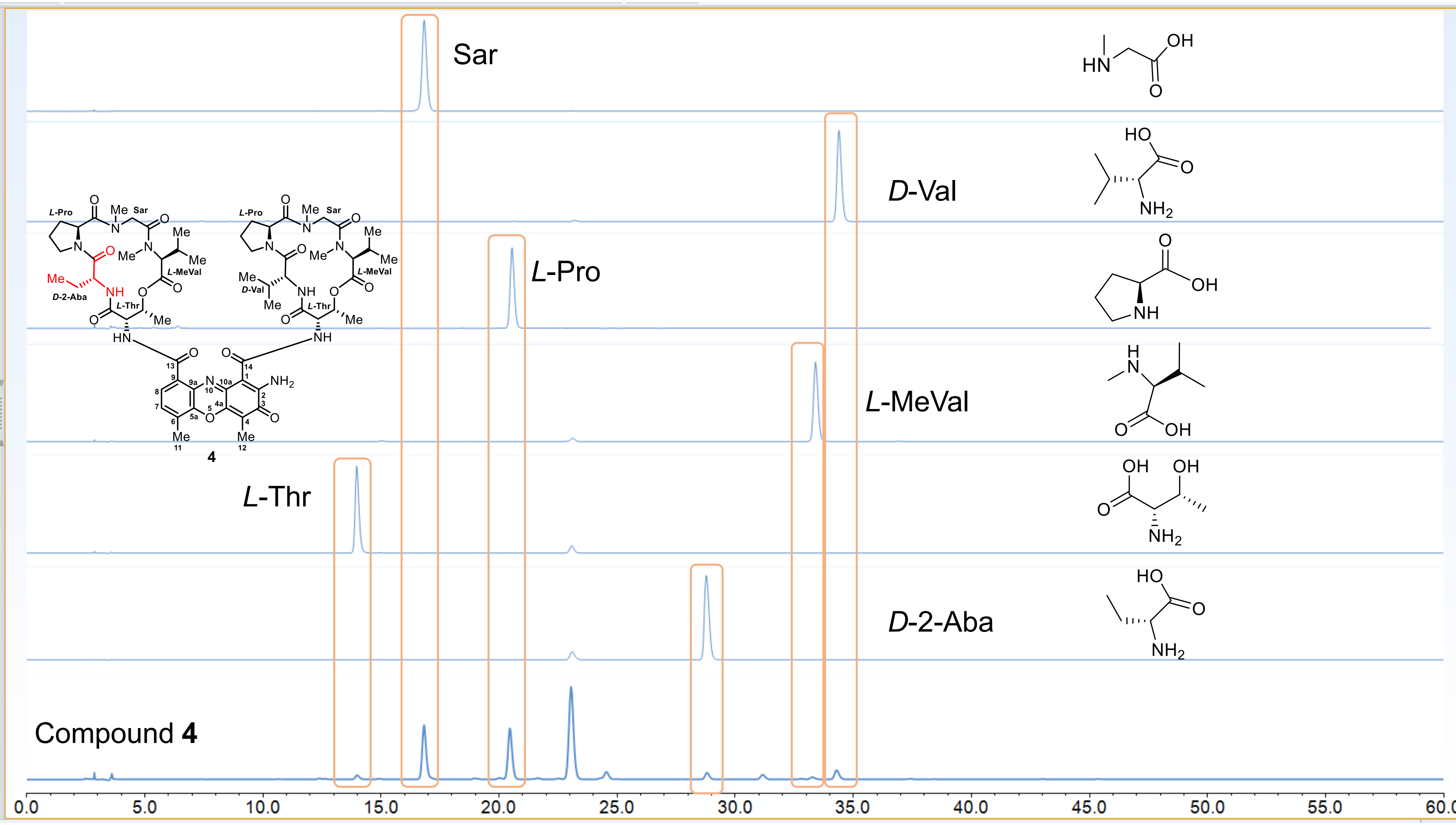

Figure S46. UV spectrum of compound **5**

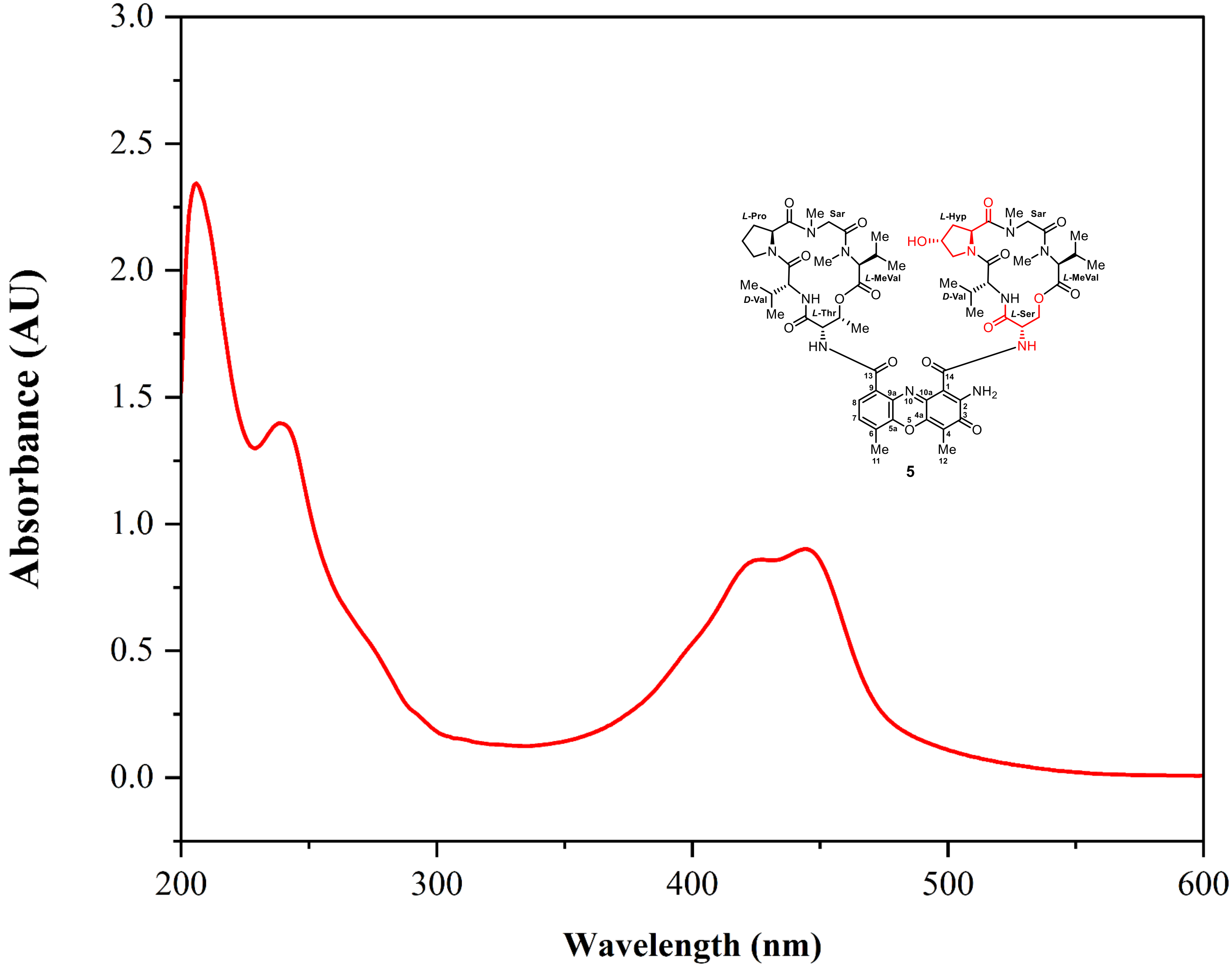

Figure S47. HR-ESIMS for compound **5**

Mass Spectrum SmartFormula Report

Analysis Info

Analysis Name	D:\Data\MS\data\202505\panghe_S-29-31-2_pos_35_01_19965.d	Acquisition Date	5/29/2025 4:44:31 PM
Method	LC_Direct Infusion_pos_100-1000mz.m	Operator	SCSIO
Sample Name	panghe_S-29-31-2_pos	Instrument	maXis 255552.00029
Comment			

Acquisition Parameter

Source Type	ESI	Ion Polarity	Positive	Set Nebulizer	0.4 Bar
Focus	Active	Set Capillary	4500 V	Set Dry Heater	180 °C
Scan Begin	100 m/z	Set End Plate Offset	-500 V	Set Dry Gas	4.0 l/min
Scan End	2000 m/z	Set Charging Voltage	0 V	Set Divert Valve	Waste
		Set Corona	0 nA	Set APCI Heater	0 °C

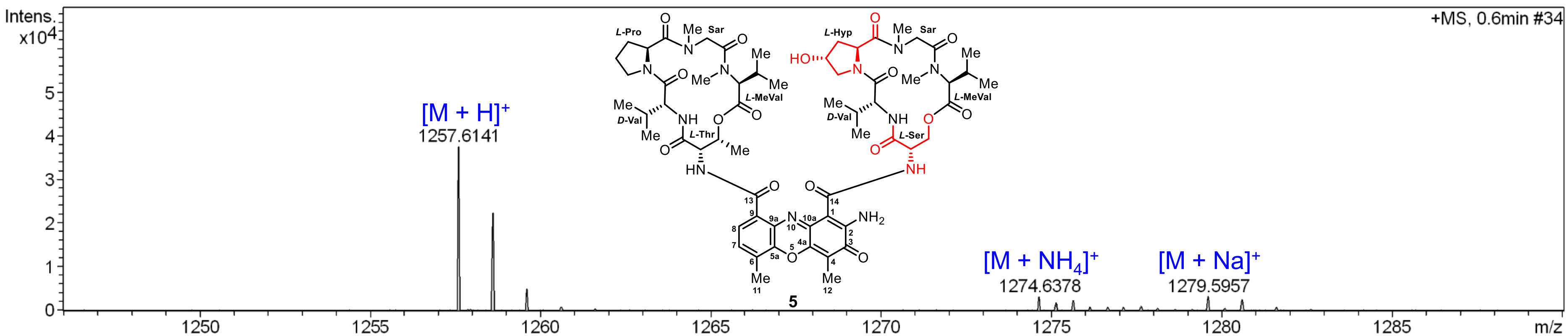

Meas. m/z	#	Ion Formula	Score	m/z	err [ppm]	err [mDa]	mSigma	rdb	e⁻ Conf	N-Rule
1257.6141	1	C61H85N12O17	100.00	1257.6150	0.7	0.9	85.1	25.5	even	ok
1274.6378	1	C61H88N13O17	100.00	1274.6416	3.0	3.8	107.4	24.5	even	ok
1279.5957	1	C61H84N12NaO17	100.00	1279.5970	1.0	1.3	35.9	25.5	even	ok

Figure S48. ^{1}H NMR (600 MHz) spectrum of compound **5** in $CDCl_3$

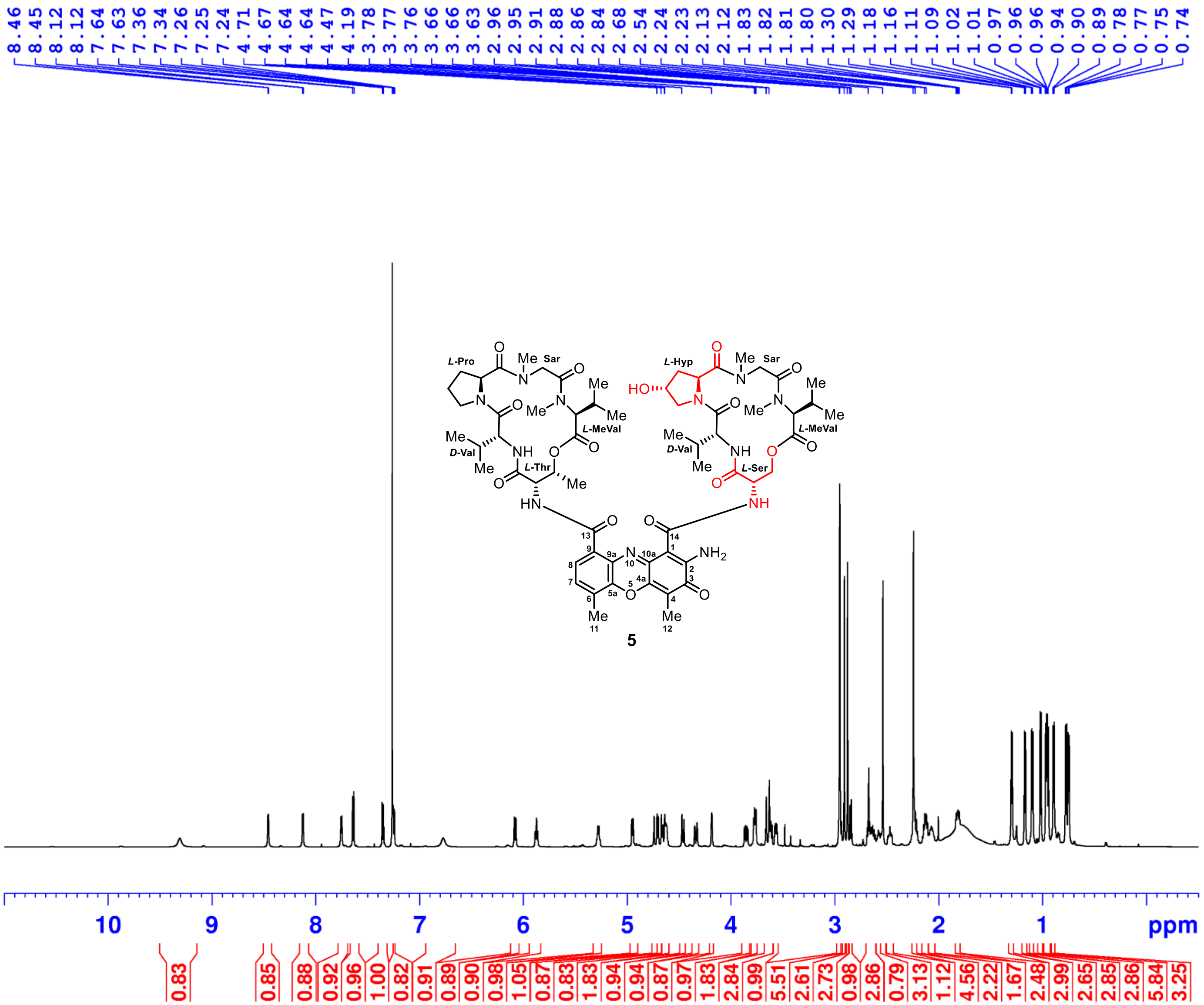

NAME	24720_S29312_H
EXPNO	62
PROCNO	1
Date_	20240720
Time	12.46 h
INSTRUM	Avance NEO 600
PROBHD	Z168348_0005 (
PULPROG	zg30
TD	65536
SOLVENT	CDCl3
NS	160
DS	2
SWH	11904.762 Hz
FIDRES	0.363304 Hz
AQ	2.7525620 sec
RG	16
DW	42.000 usec
DE	14.39 usec
TE	298.2 K
D1	1.00000000 sec
TD0	1
SFO1	600.1937062 MHz
NUC1	1H
P0	2.67 usec
P1	8.00 usec
SI	65536
SF	600.1900148 MHz
WDW	EM
SSB	0
LB	0.30 Hz
GB	0
PC	1.00

Figure S48-continue

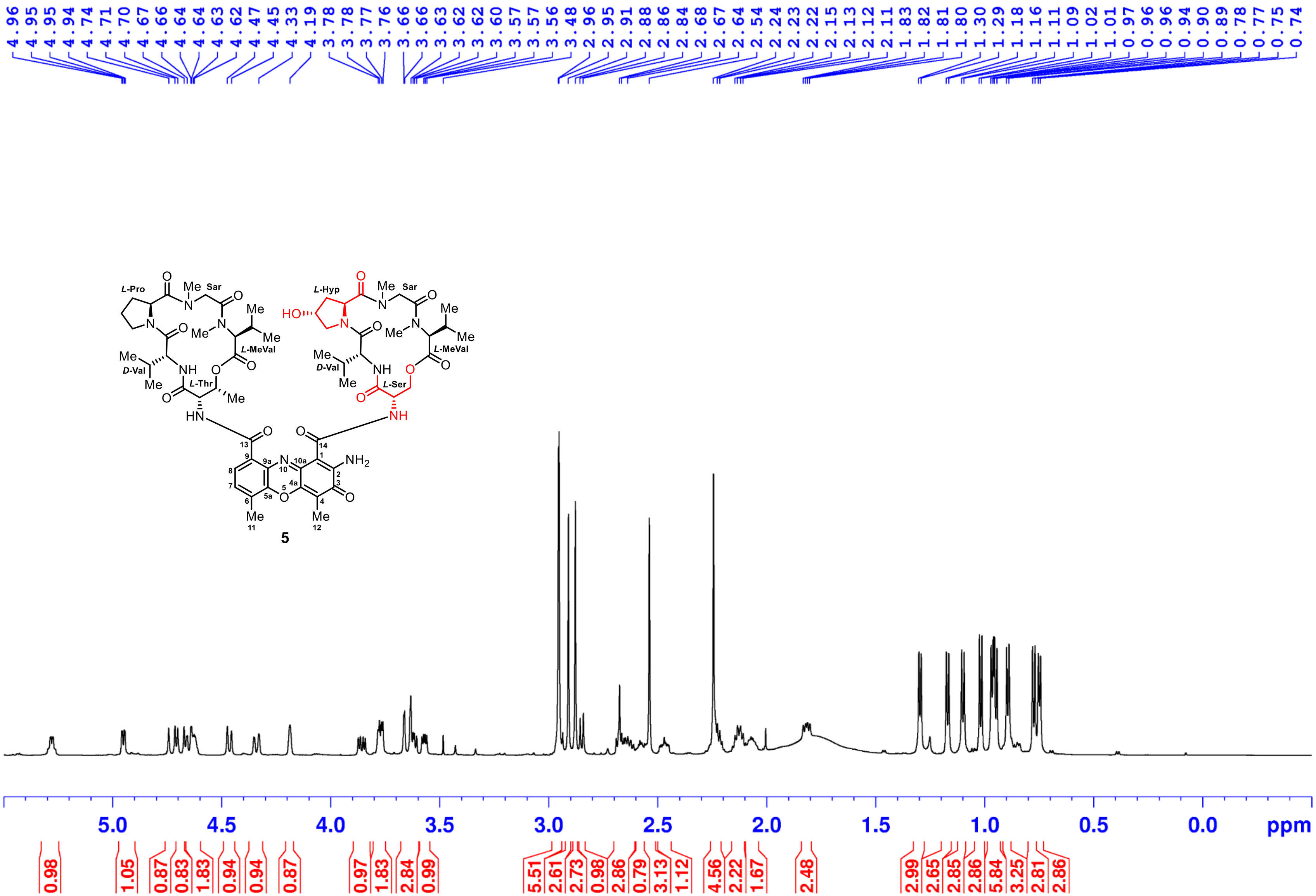

Figure S48-continue

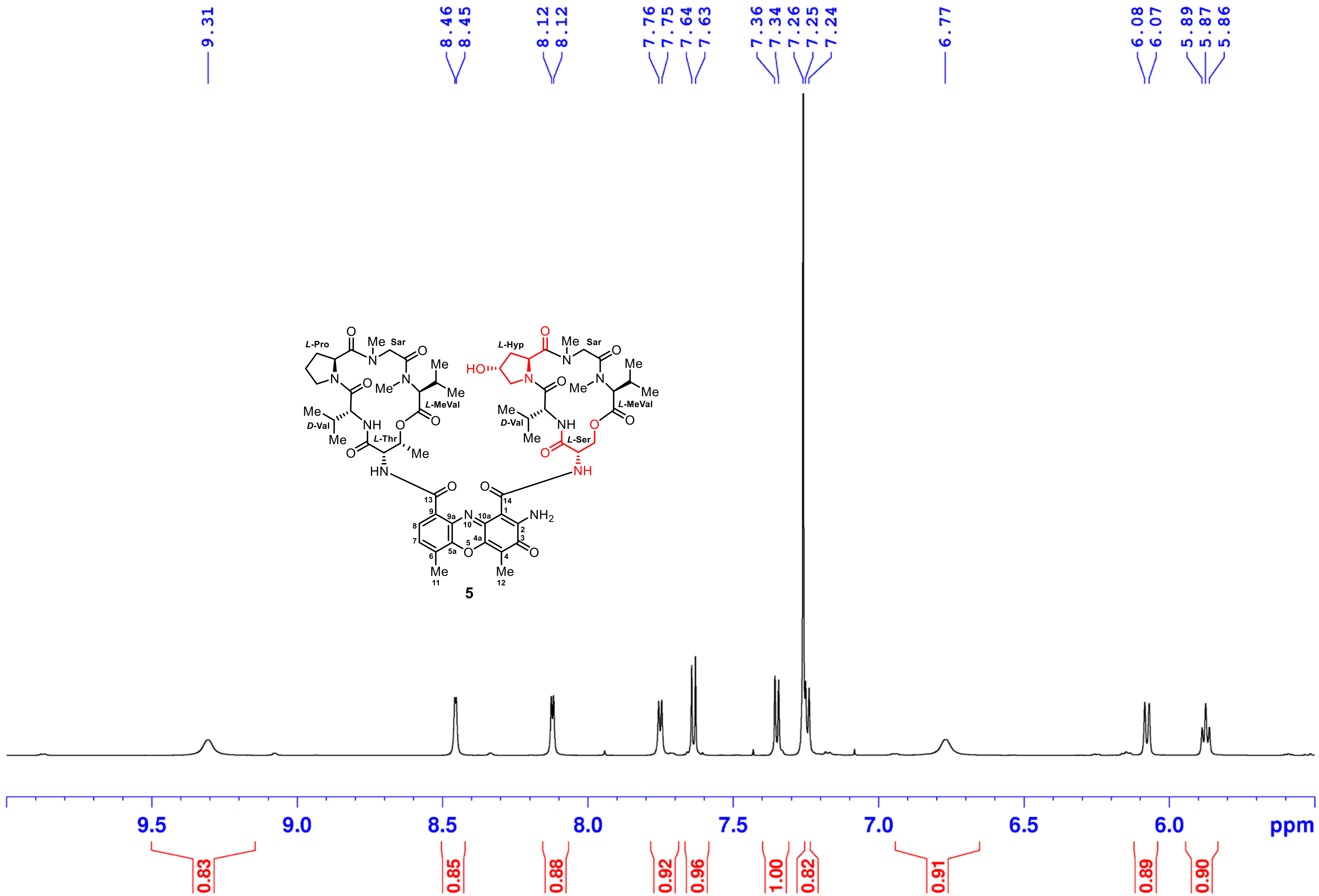

Figure S49. ^{13}C NMR (150 MHz) spectrum of compound **5** in $CDCl_3$

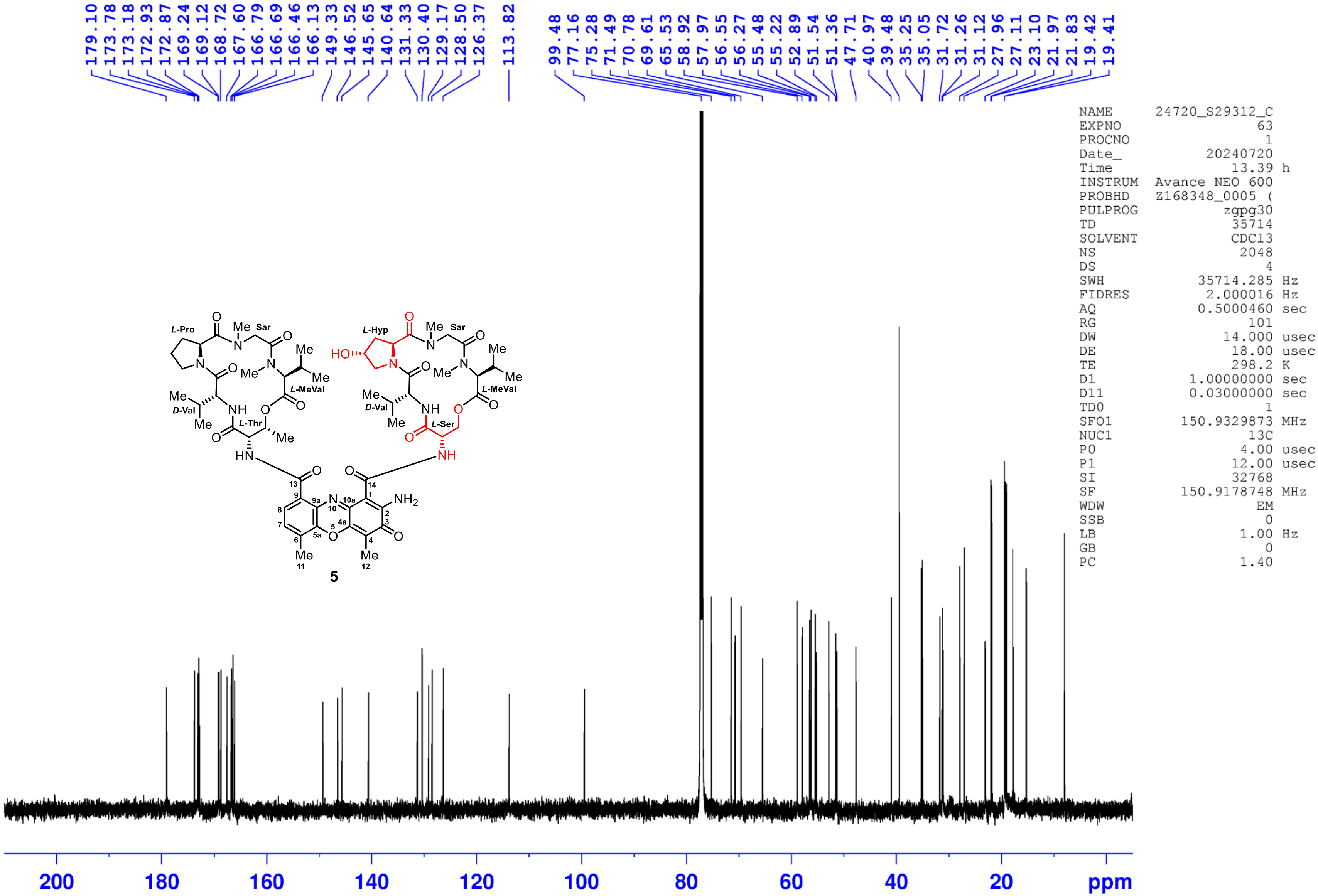

Figure S49-continue

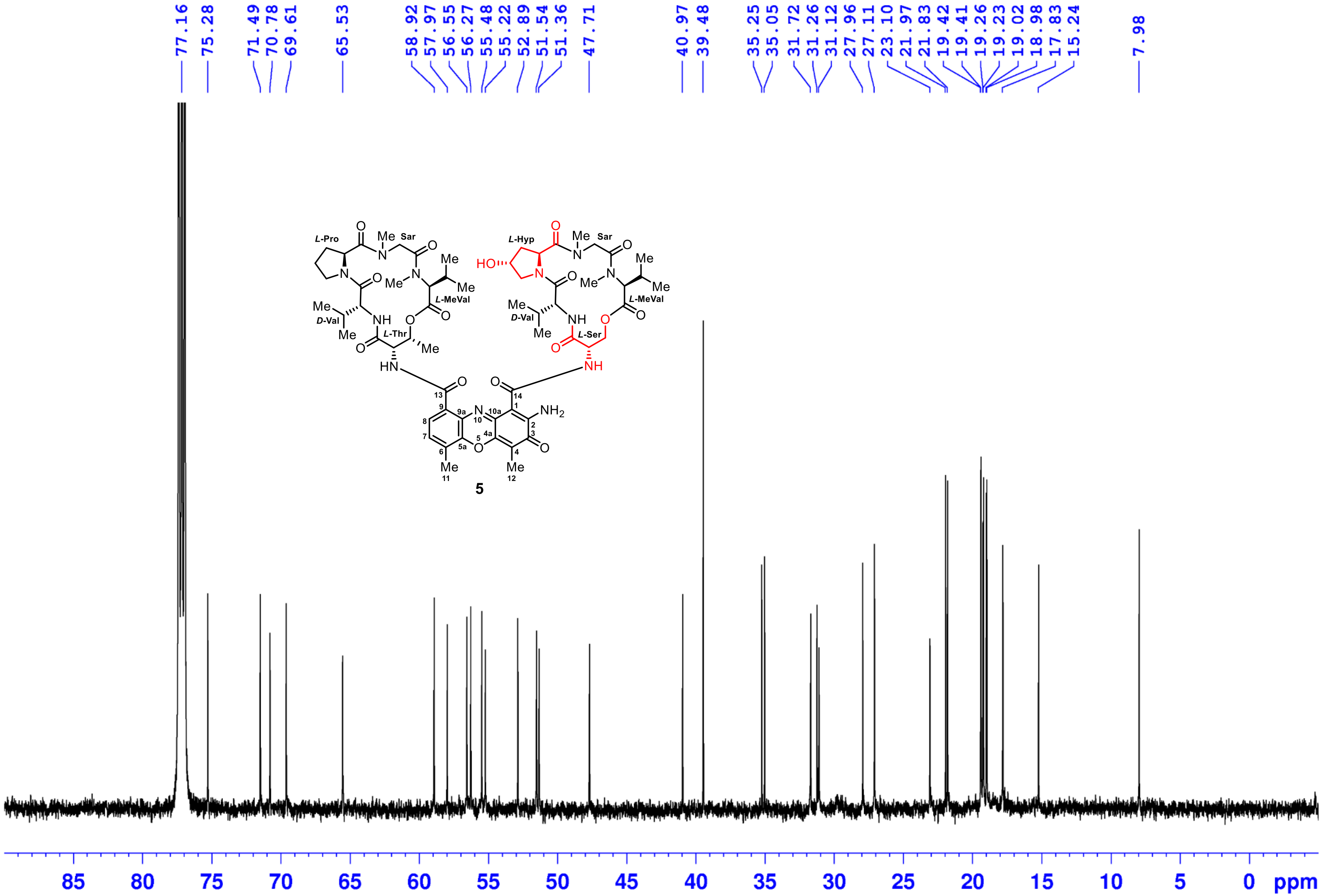

Figure S49-continue

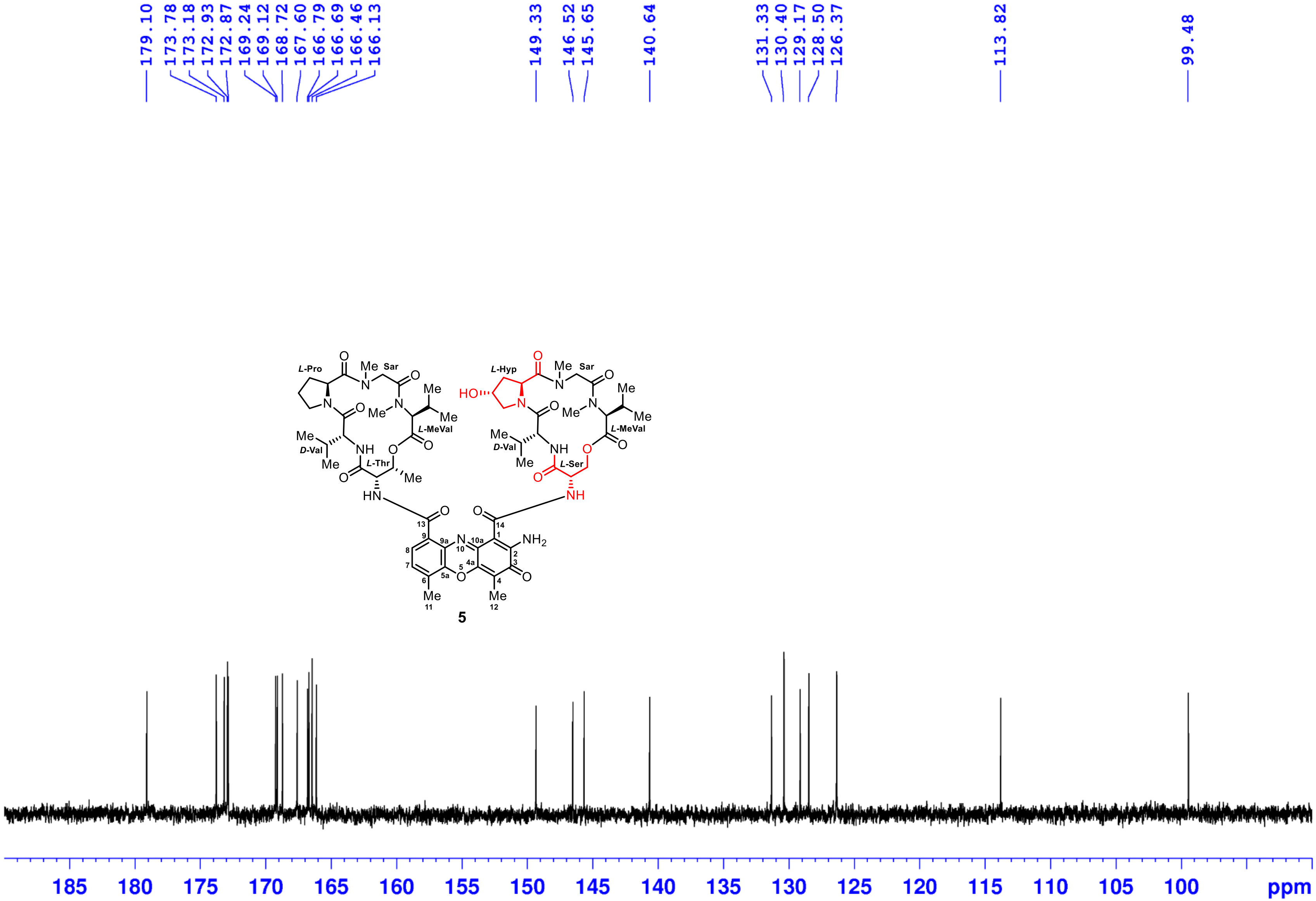

Figure S50. DEPT135 (150 MHz) spectrum of compound **5** in $CDCl_3$

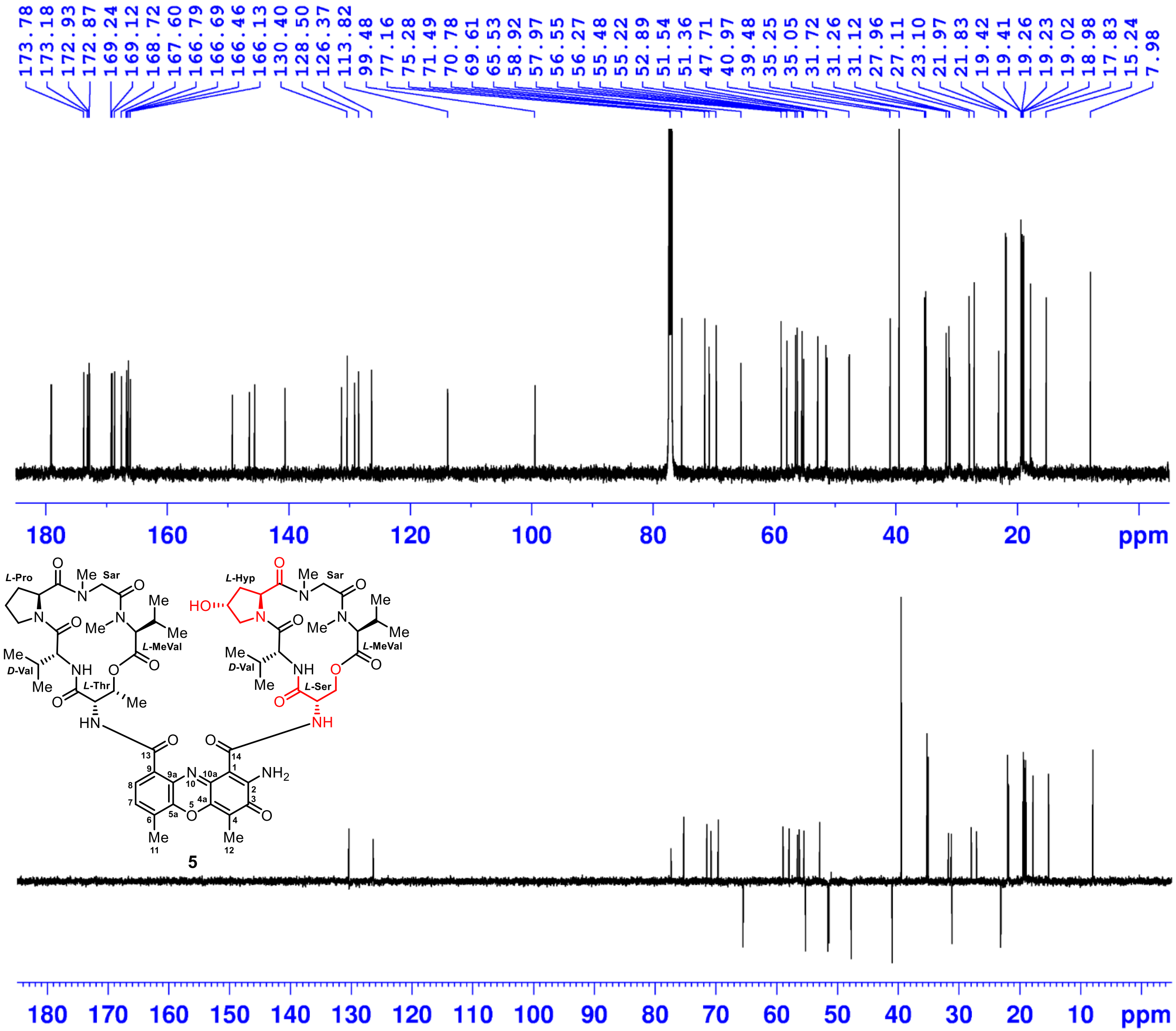

NAME	24830_S29312_135C
EXPNO	26
PROCNO	1
Date_	20240831
Time	4.03 h
INSTRUM	Avance NEO 600
PROBHD	Z168348_0005 (
PULPROG	deptsp135
TD	65536
SOLVENT	CDCl3
NS	512
DS	8
SWH	41666.668 Hz
FIDRES	1.271566 Hz
AQ	0.7864820 sec
RG	101
DW	12.000 usec
DE	18.00 usec
TE	298.2 K
CNST2	145.0000000
D1	2.00000000 sec
D2	0.00344828 sec
D12	0.00002000 sec
TD0	1
SFO1	150.9299690 MHz
NUC1	13C
P1	12.00 usec
P13	2000.00 usec
SI	32768
SF	150.9178750 MHz
WDW	EM
SSB	0
LB	1.00 Hz
GB	0
PC	1.40

Figure S50-continue

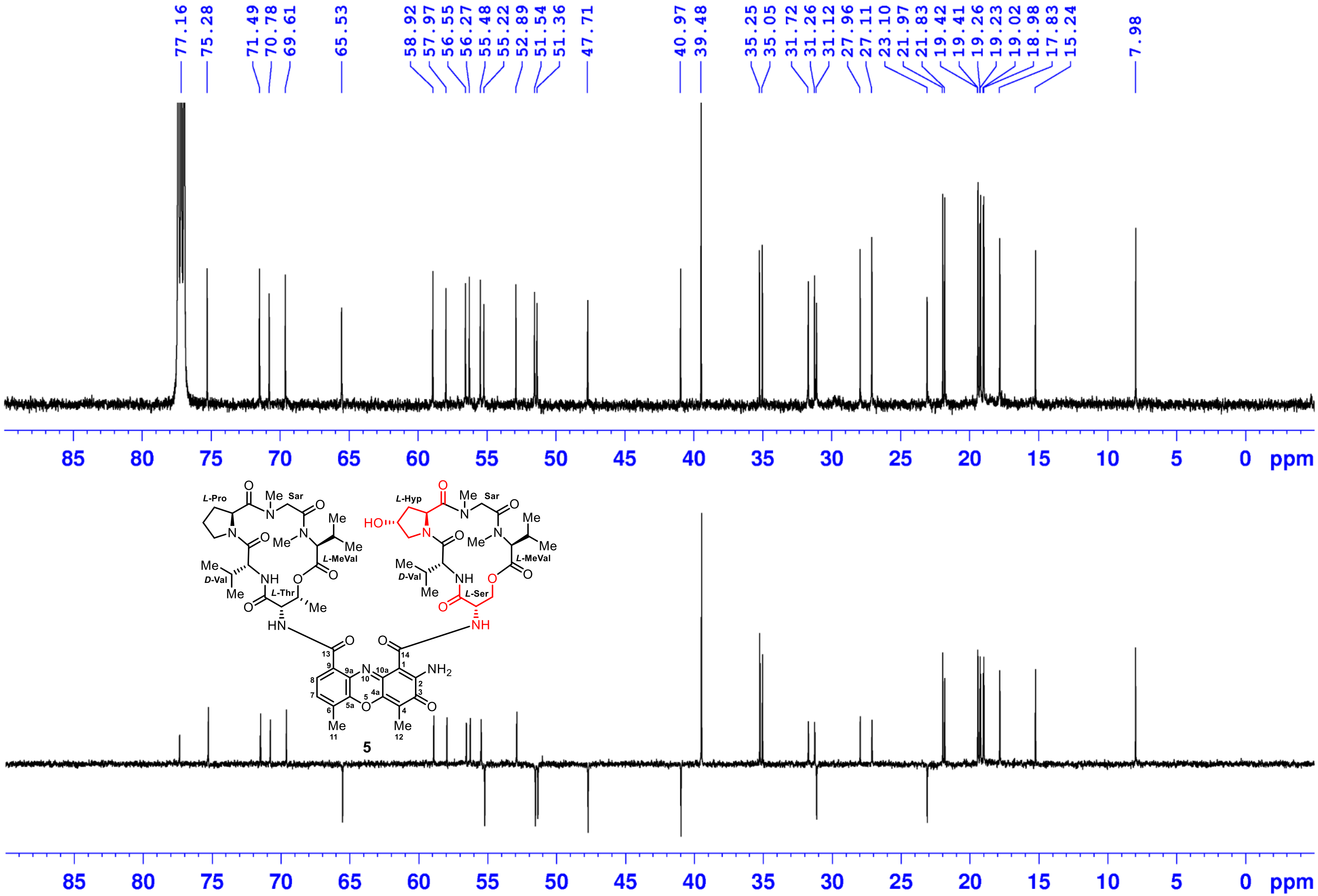

Figure S50-continue

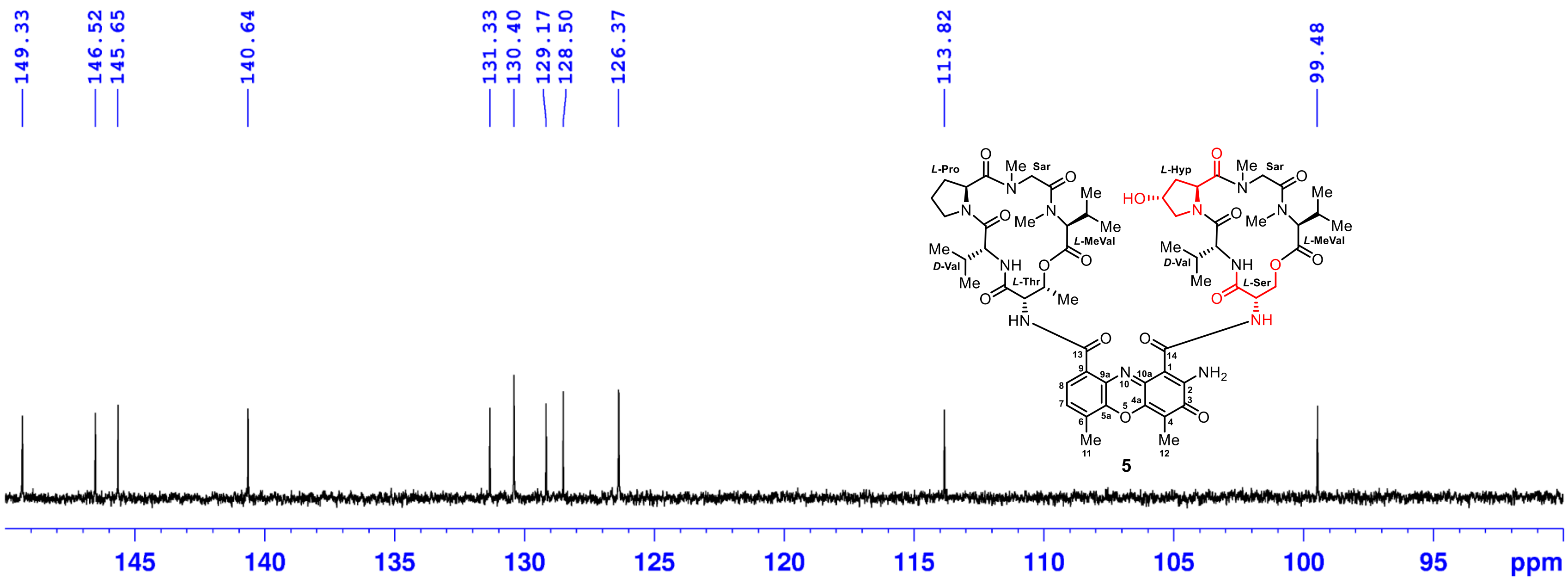

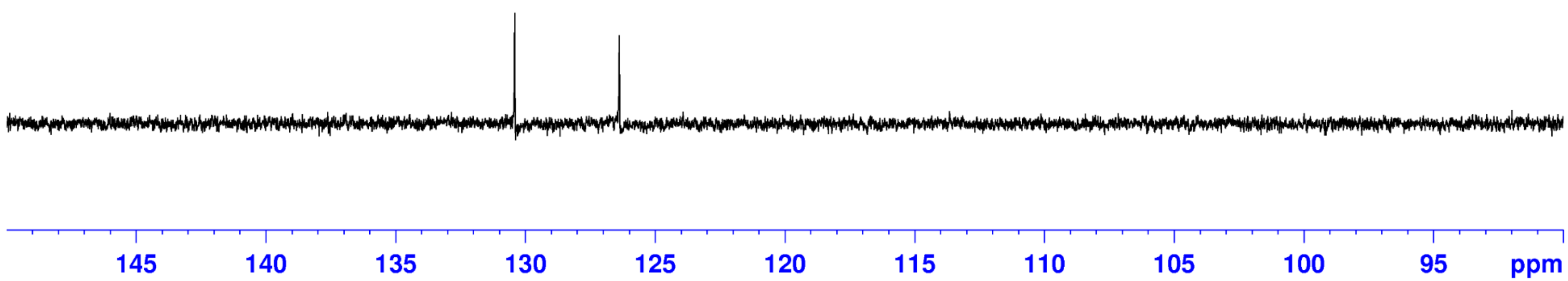

Figure S51. ^{1}H–^{1}H COSY (600 MHz) spectrum of compound **5** in $CDCl_3$

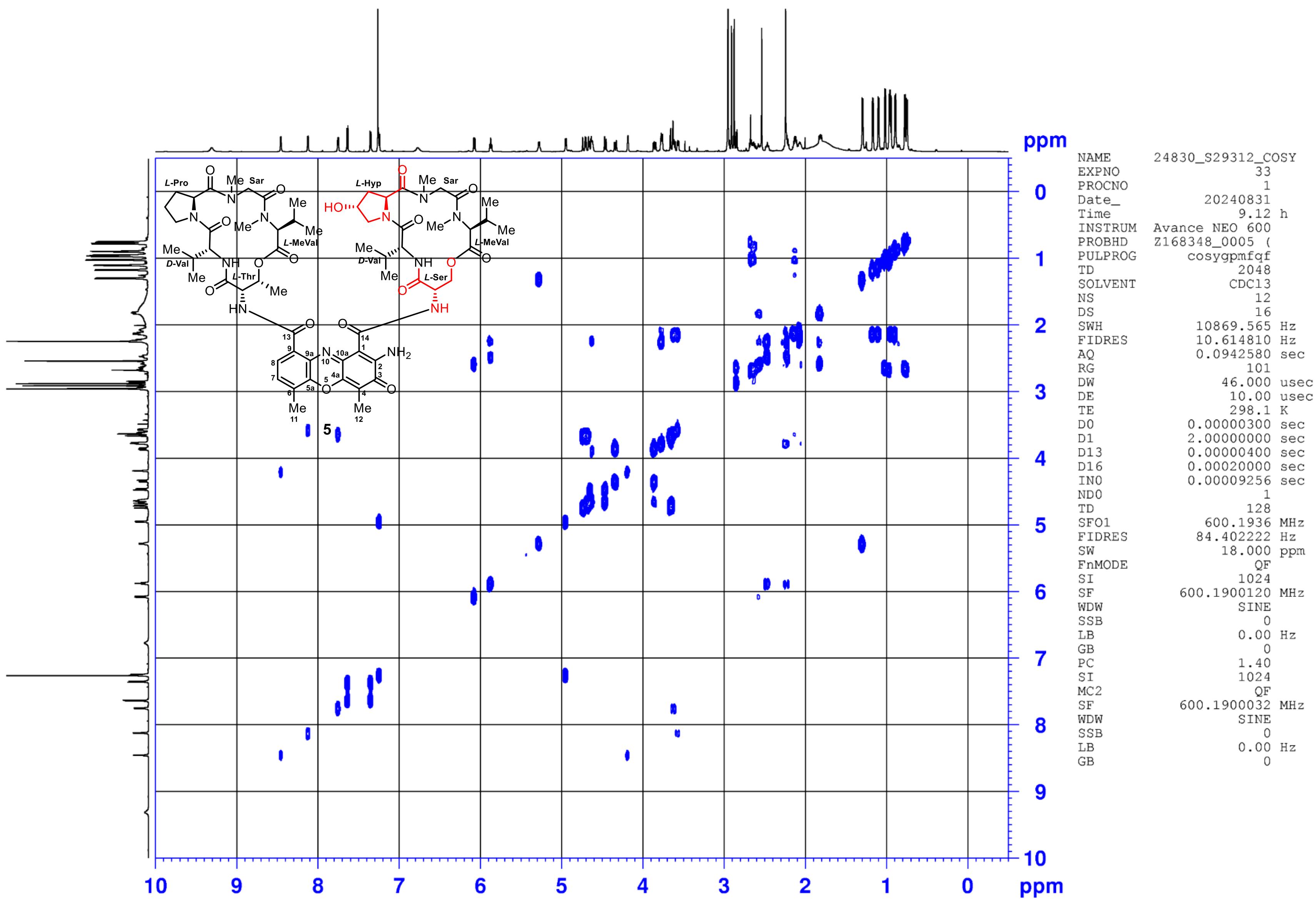

Figure S51-continue

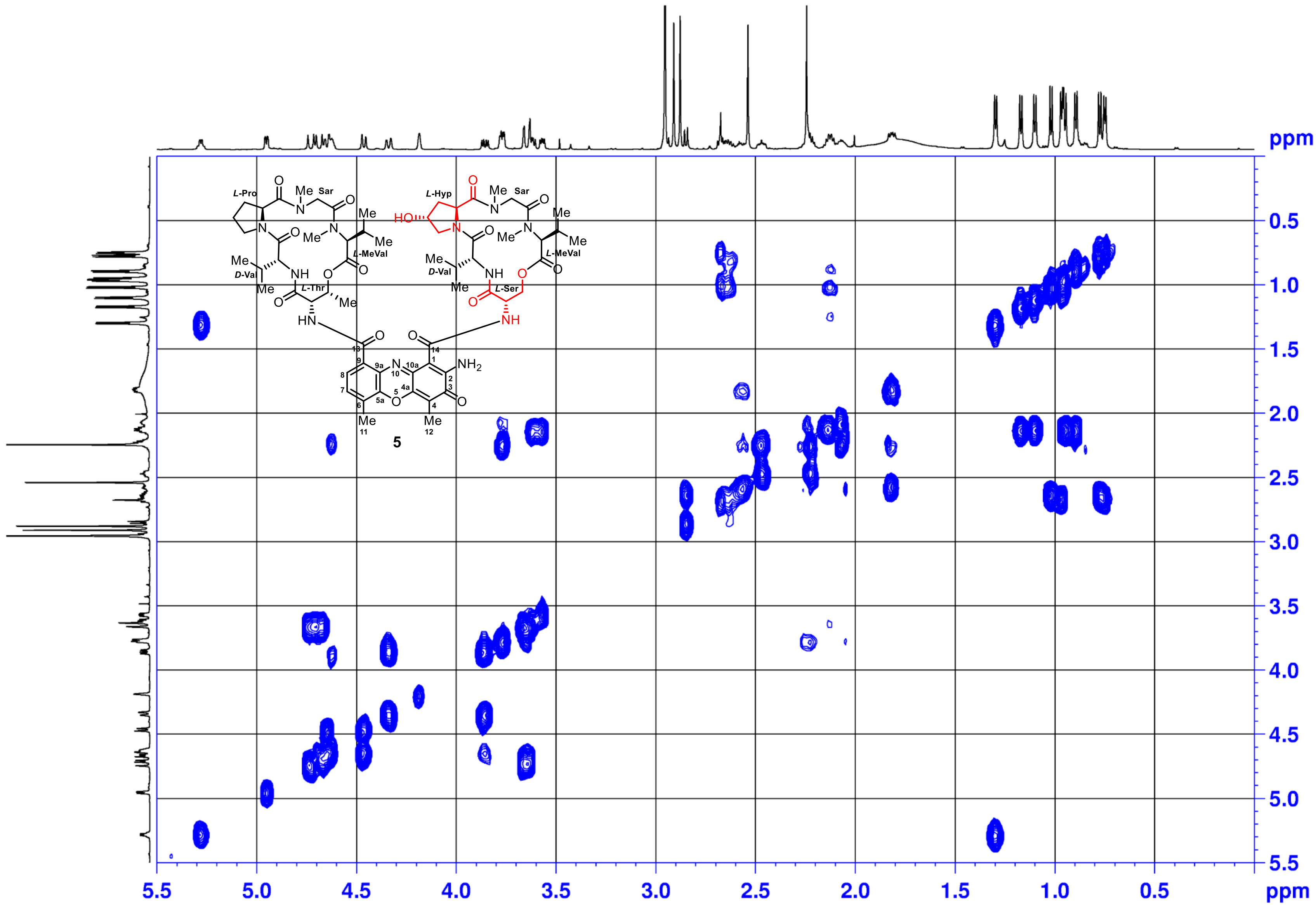

Figure S51-continue

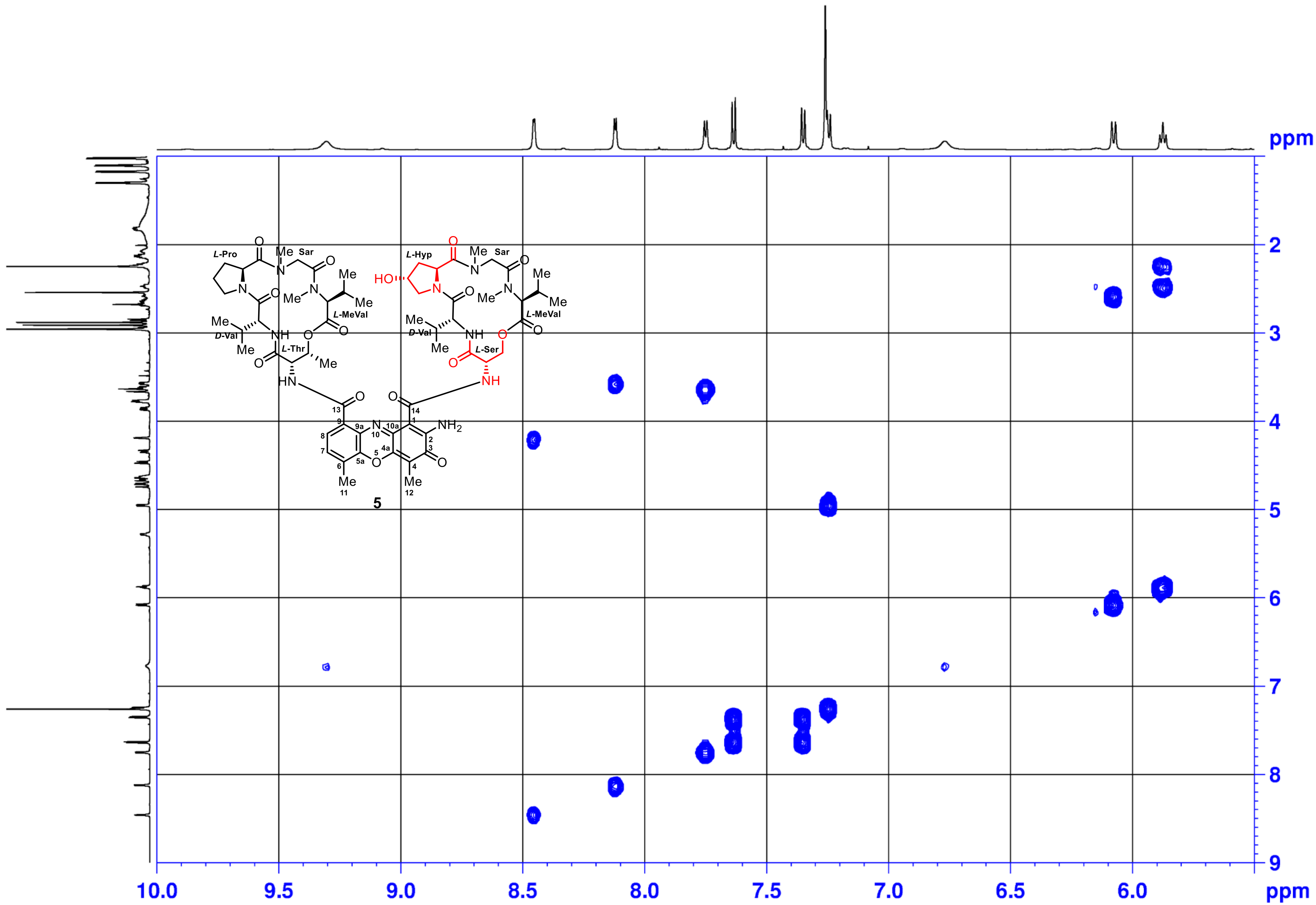

Figure S52. HSQC (600 MHz) spectrum of compound **5** in $CDCl_3$

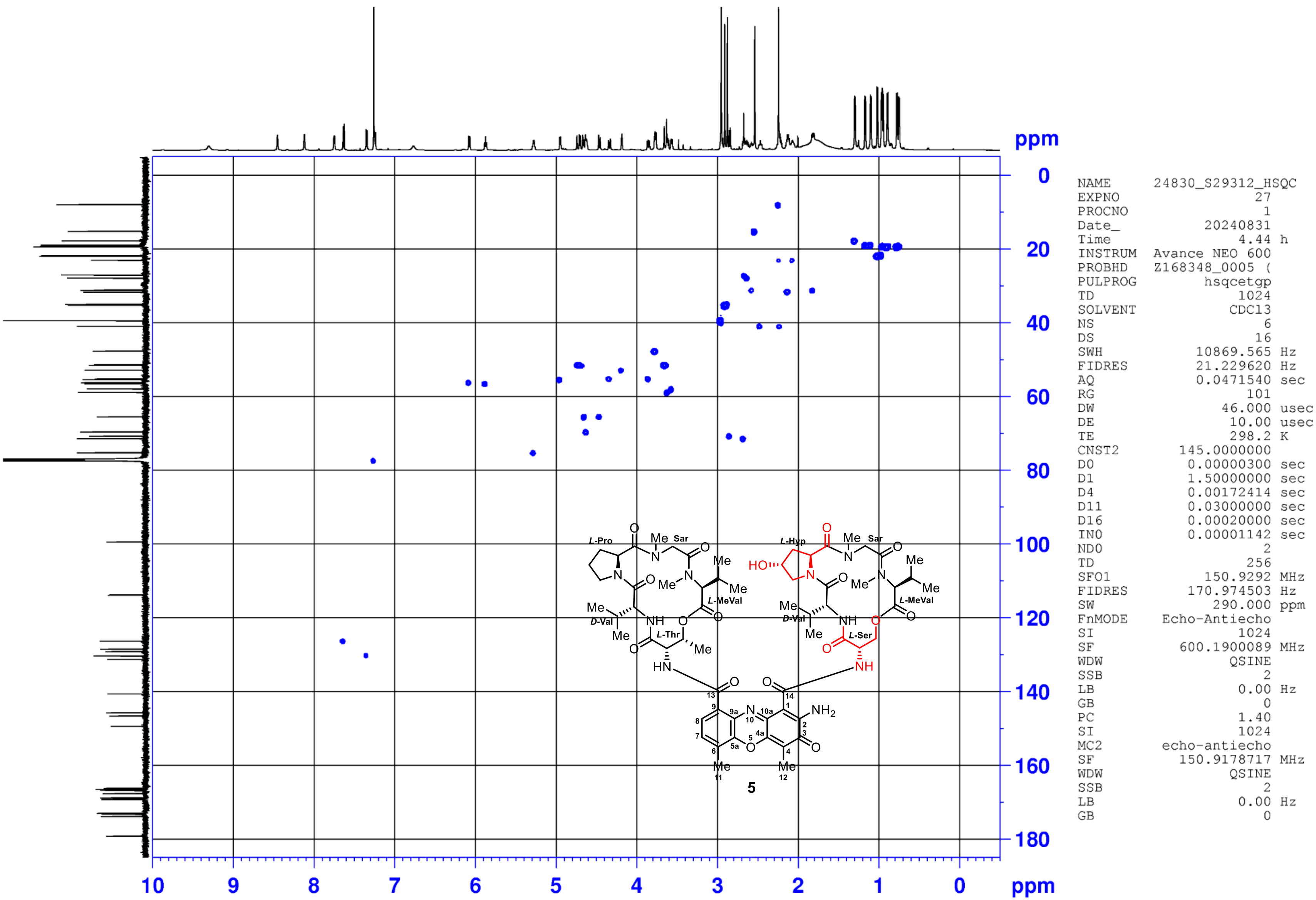

Figure S52-continue

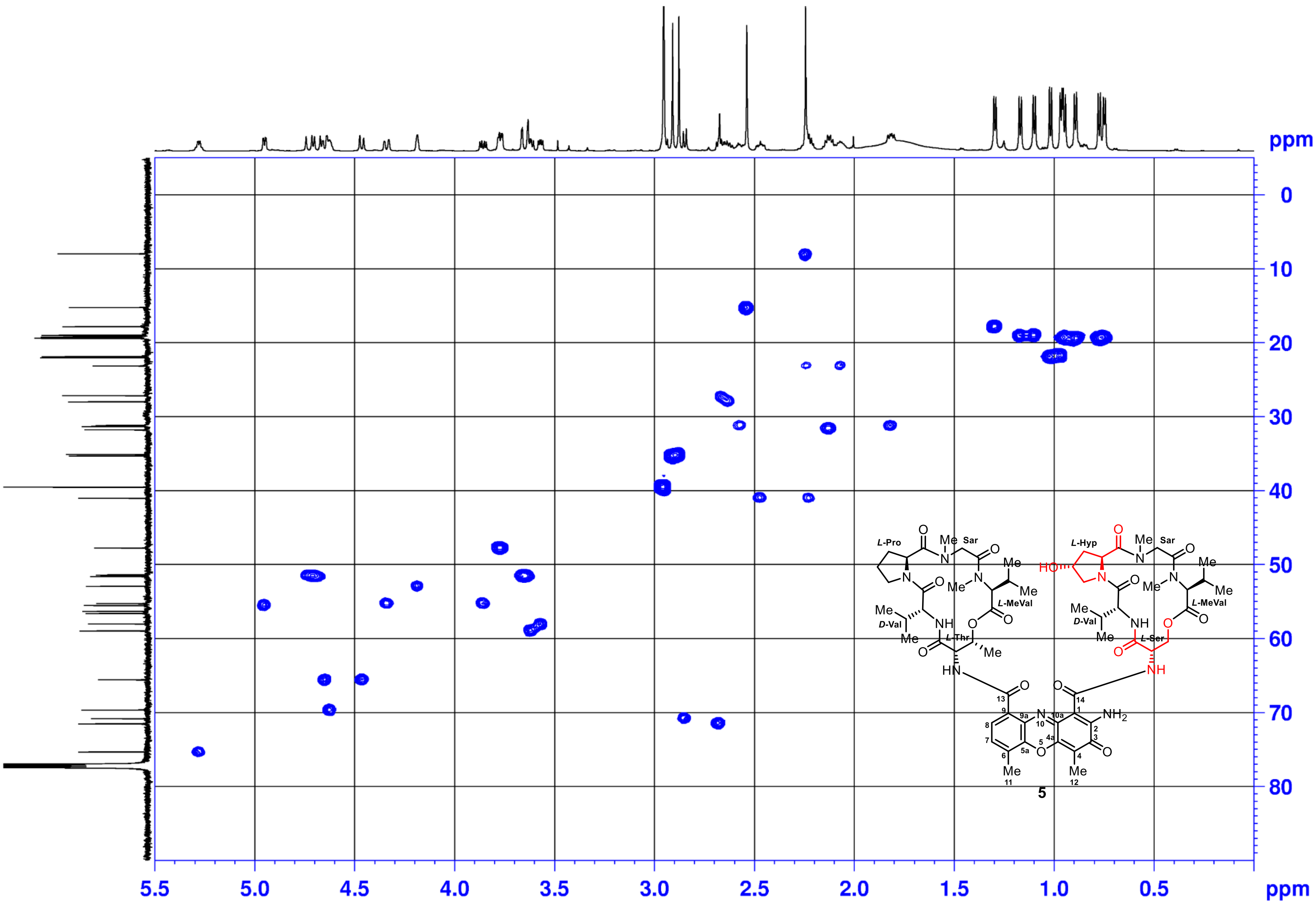

Figure S52-continue

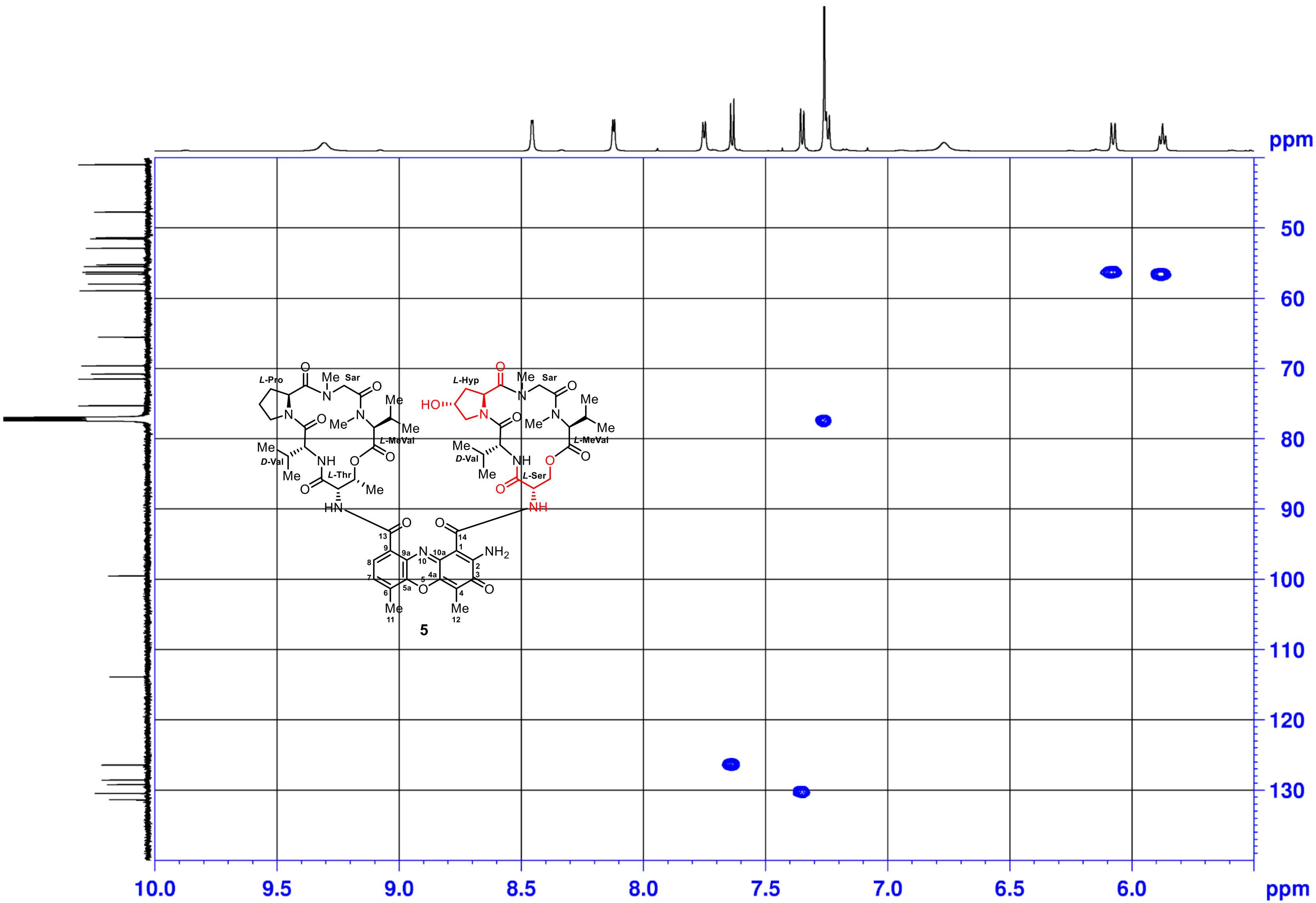

Figure S53. HMBC (600 MHz) spectrum of compound **5** in $CDCl_3$

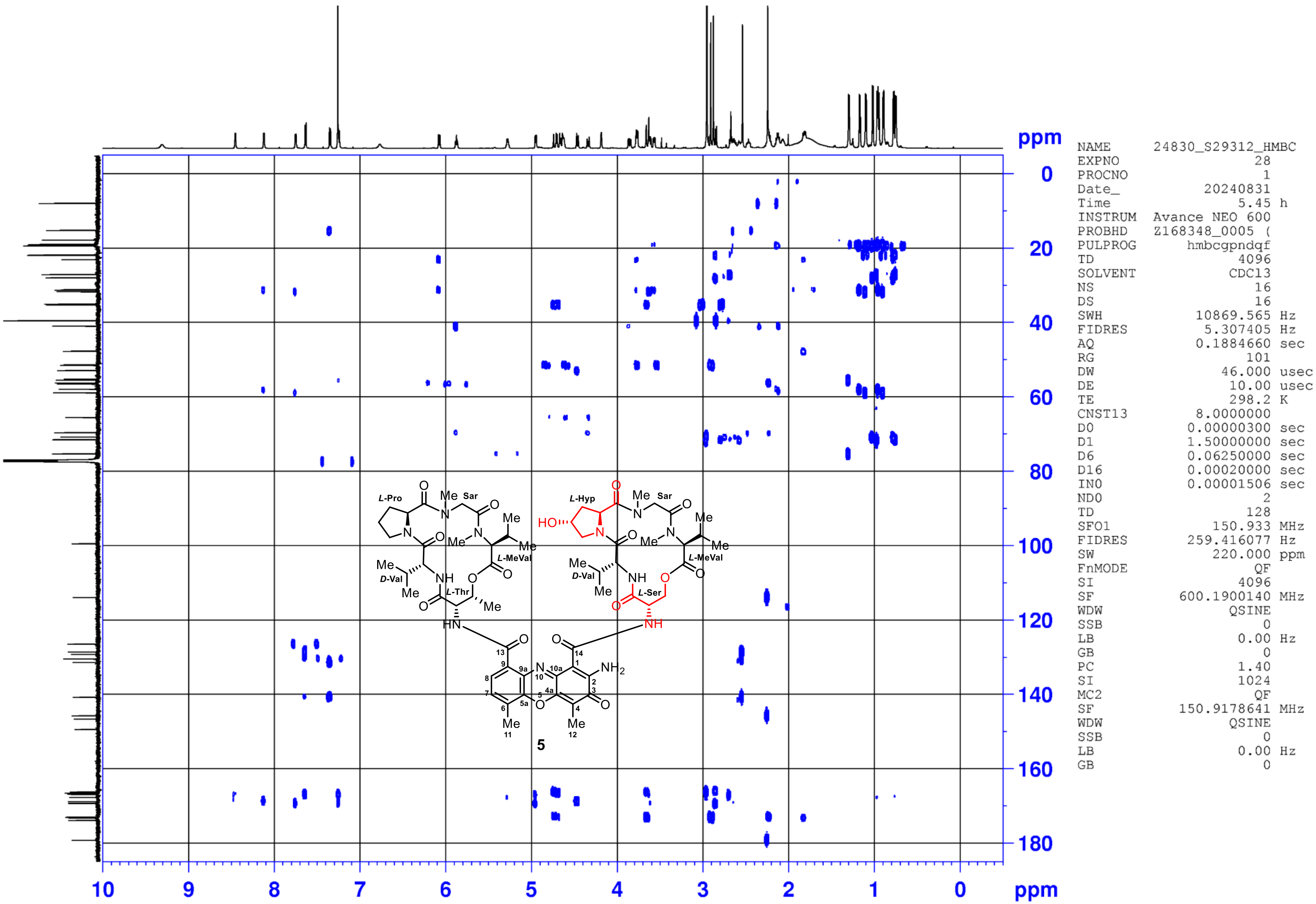

Figure S53-continue

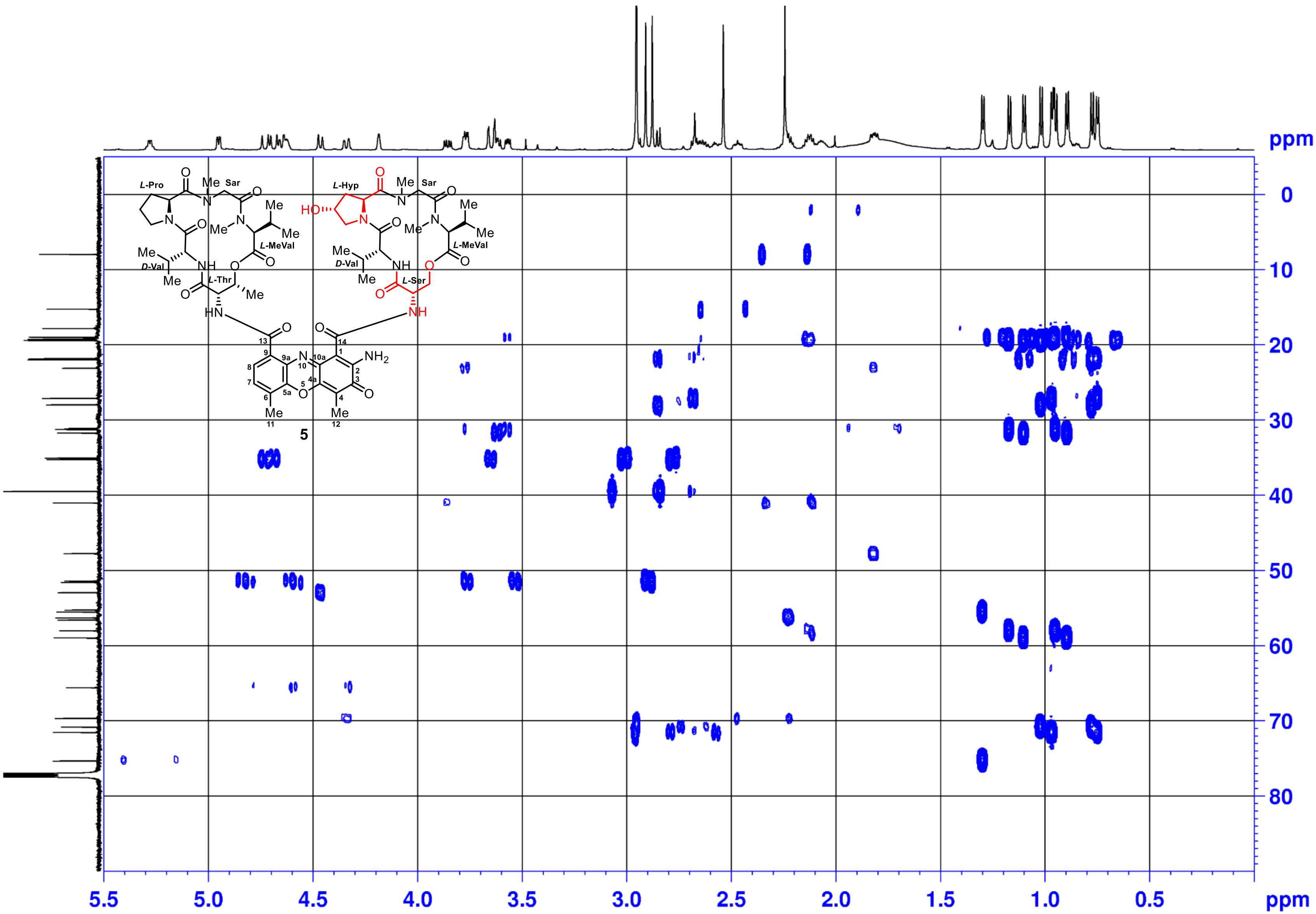

Figure S53-continue

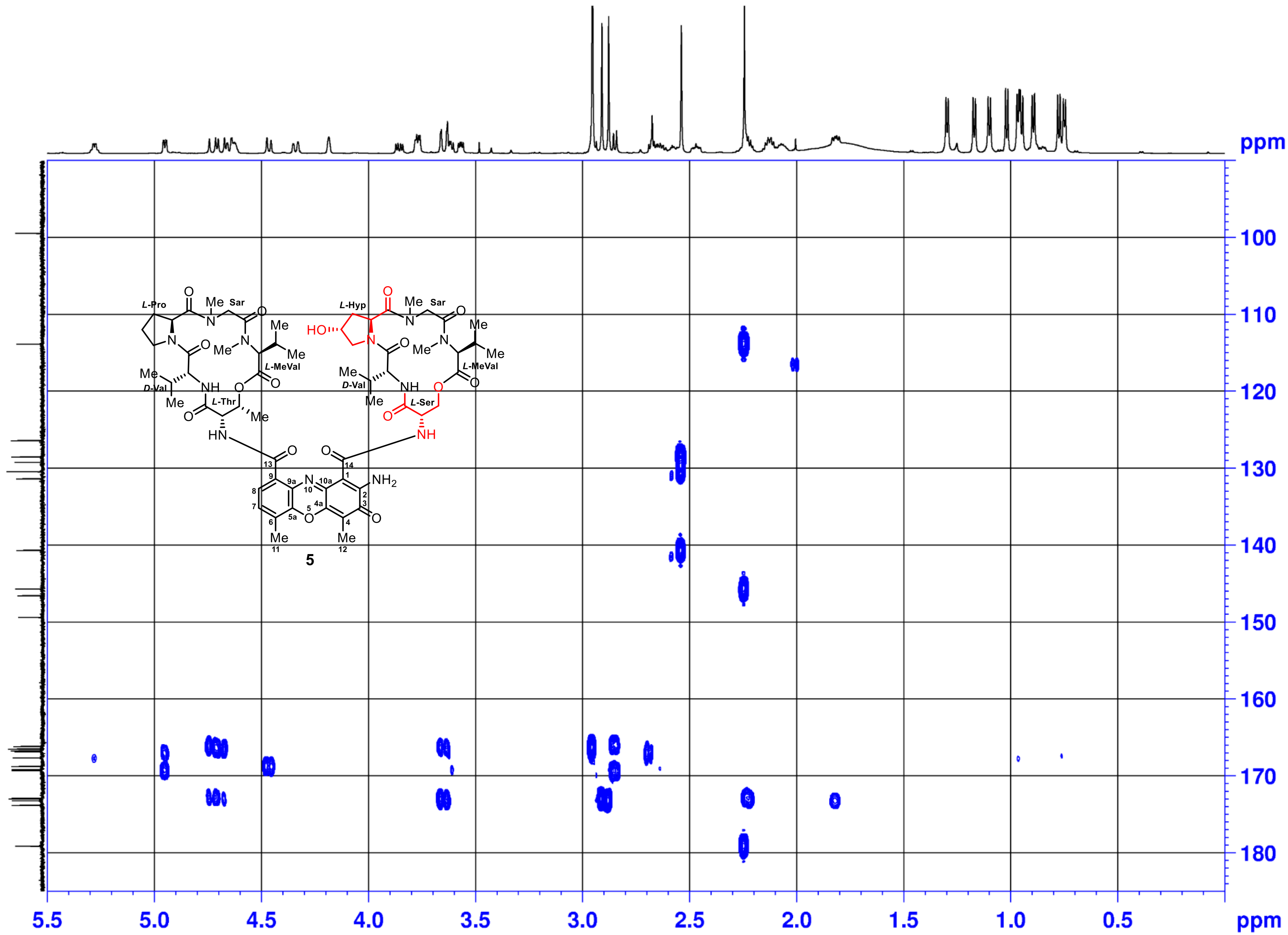

Figure S53-continue

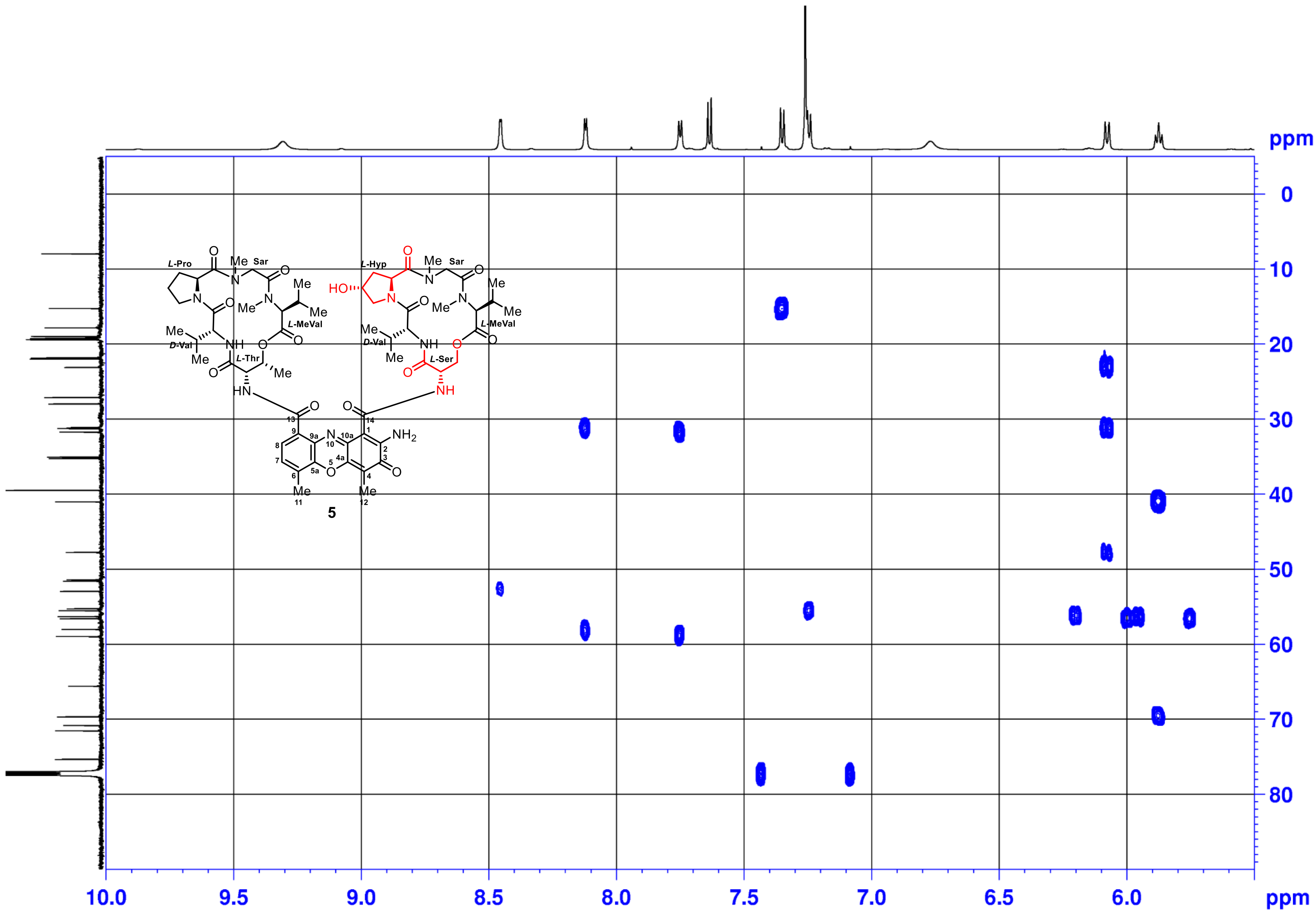

Figure S53-continue

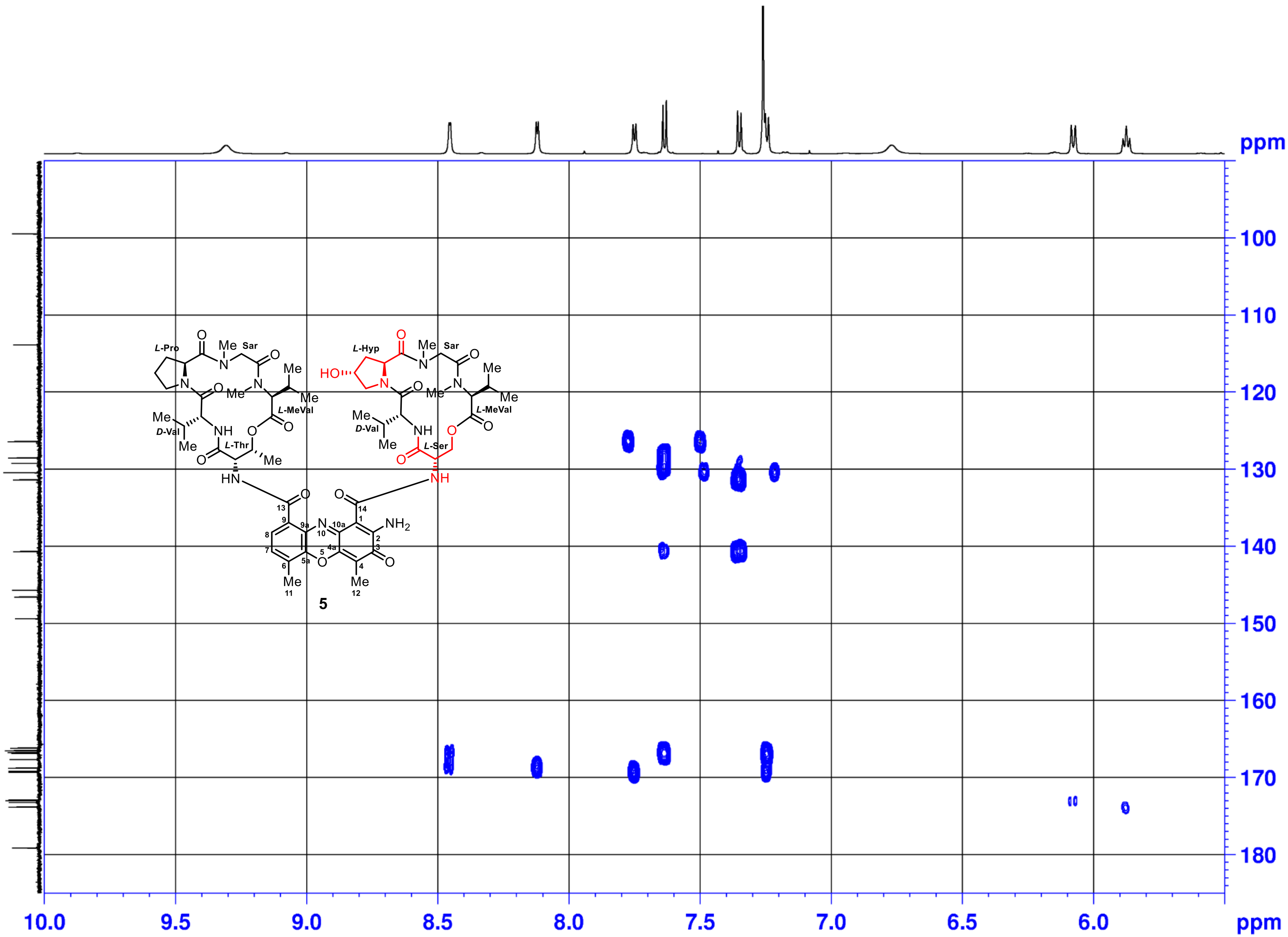

Figure S54. NOESY (600 MHz) spectrum of compound **5** in $CDCl_3$

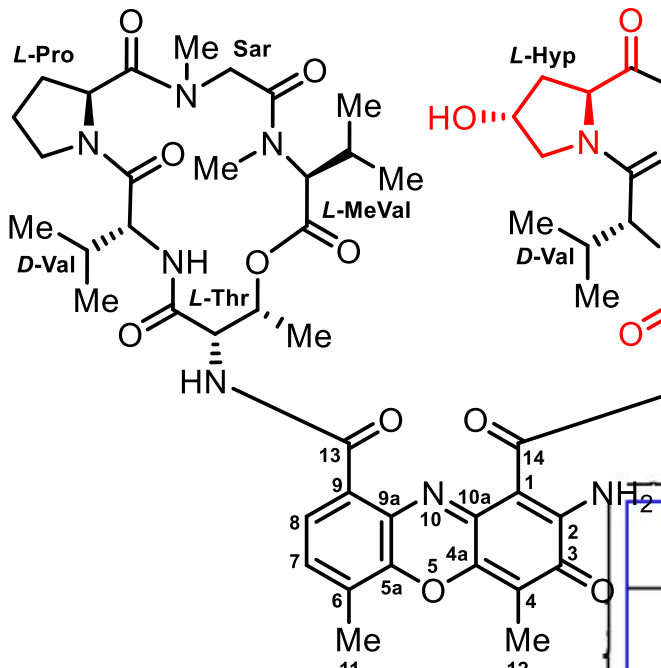

5

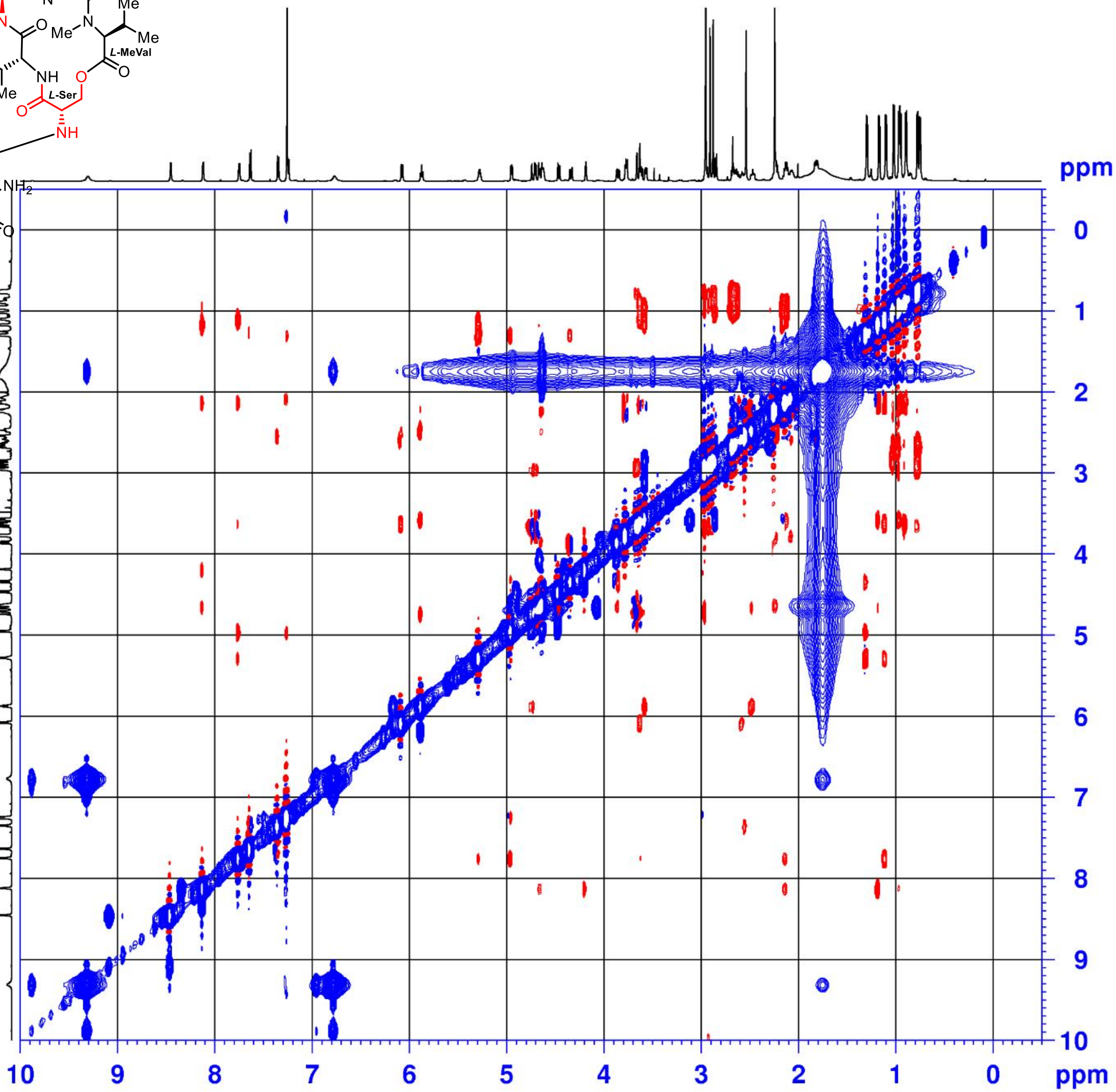

NAME	24830_S29312_NOE	
EXPNO	29	
PROCNO	1	
Date_	20240831	
Time	7.51	h
INSTRUM	Avance NEO 600	
PROBHD	Z168348_0005 (	
PULPROG	noesygpphpp	
TD	2048	
SOLVENT	CDCl3	
NS	12	
DS	32	
SWH	11904.762	Hz
FIDRES	11.625744	Hz
AQ	0.0860660	sec
RG	64	
DW	42.000	usec
DE	10.66	usec
TE	298.1	K
D0	0.00003147	sec
D1	2.00000000	sec
D8	0.30000001	sec
D11	0.03000000	sec
D12	0.00002000	sec
D16	0.00020000	sec
IN0	0.00008331	sec
ND0	1	
TD	256	
SFO1	600.1928	MHz
FIDRES	46.890064	Hz
SW	20.000	ppm
FnMODE	States-TPPI	
SI	1024	
SF	600.1900053	MHz
WDW	QSINE	
SSB	2	
LB	0.00	Hz
GB	0	
PC	1.00	
SI	1024	
MC2	States-TPPI	
SF	600.1900044	MHz
WDW	QSINE	
SSB	2	
LB	0.00	Hz
GB	0	

Figure S54-continue

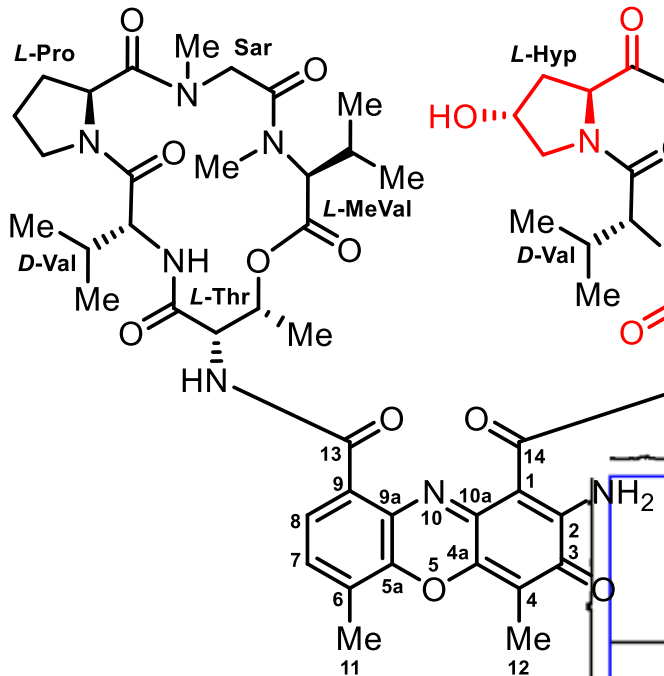

5

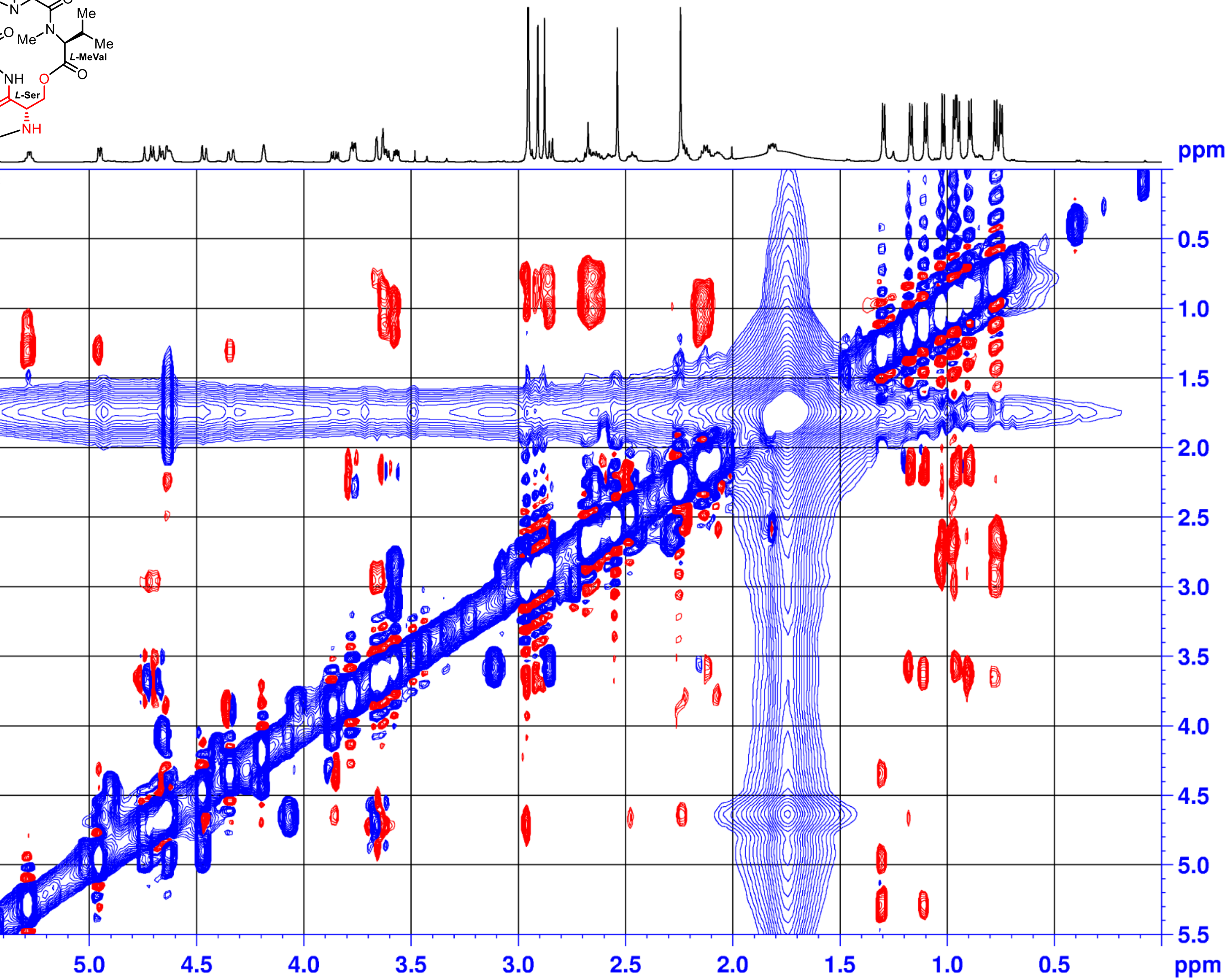

Figure S54-continue

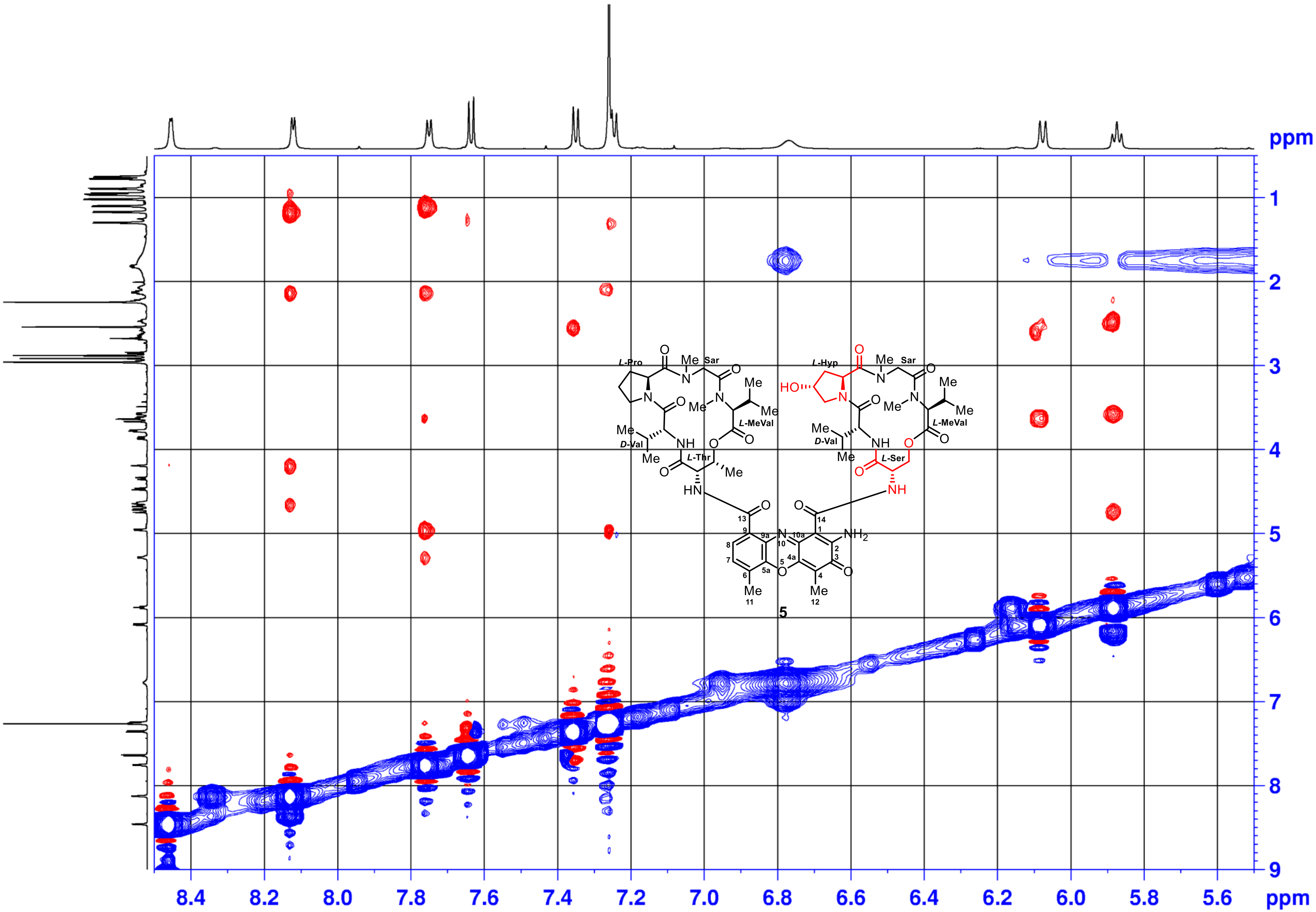

Figure S55. Marfey's analysis of Hyp standard

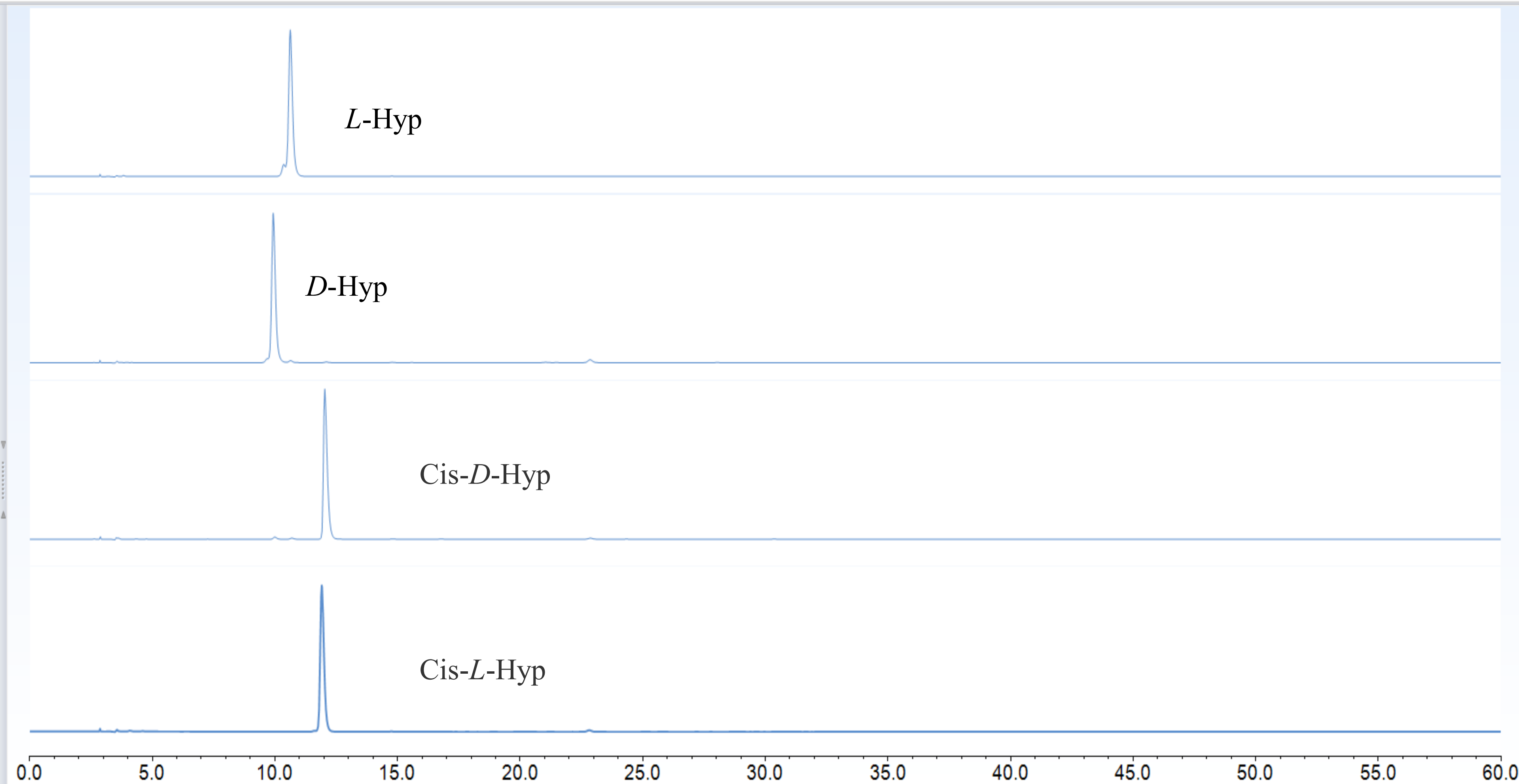

Figure S56. Marfey's analysis of compound **5** and standard amino acids

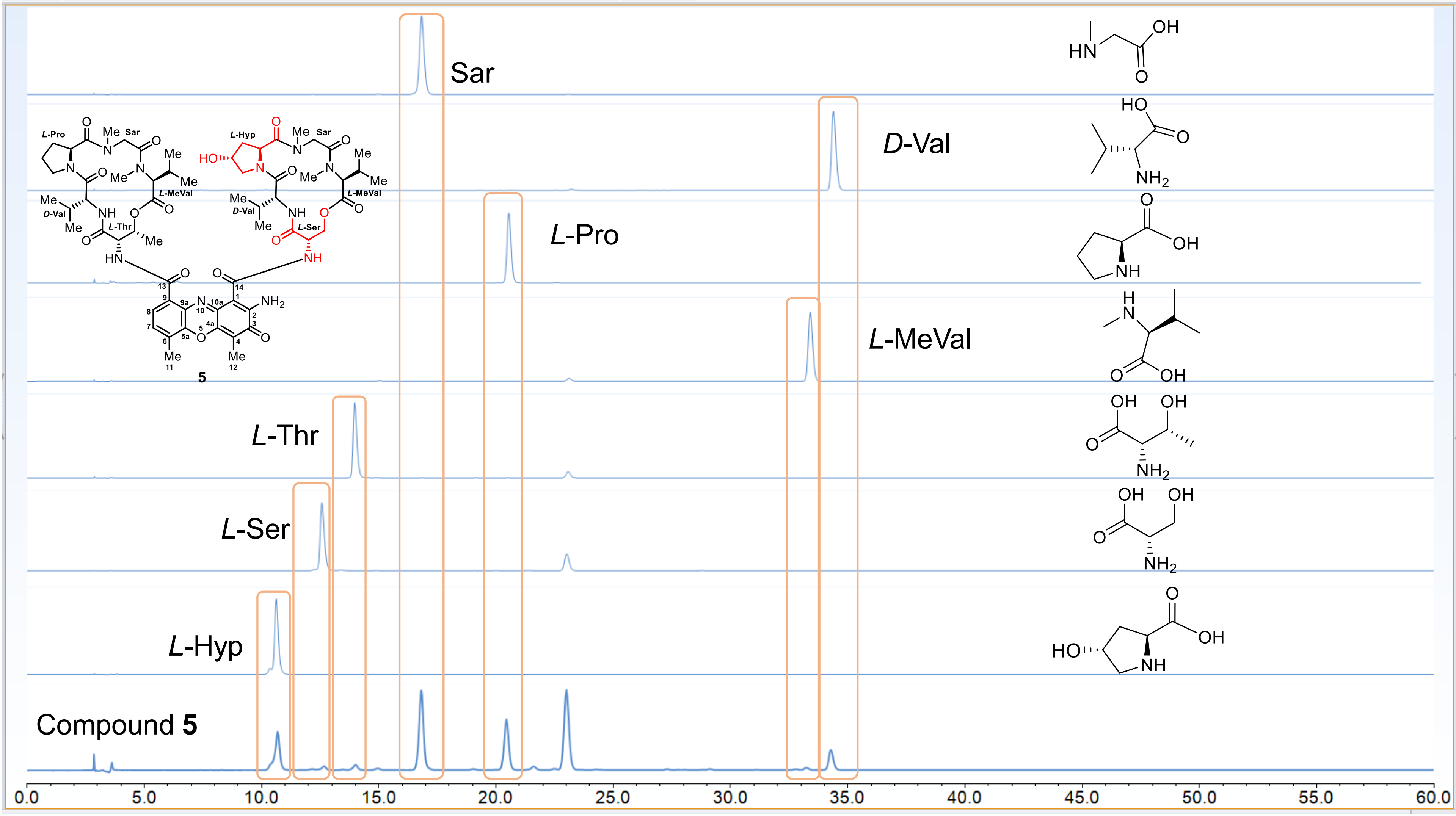

Figure S57. UV spectrum of compound **6**

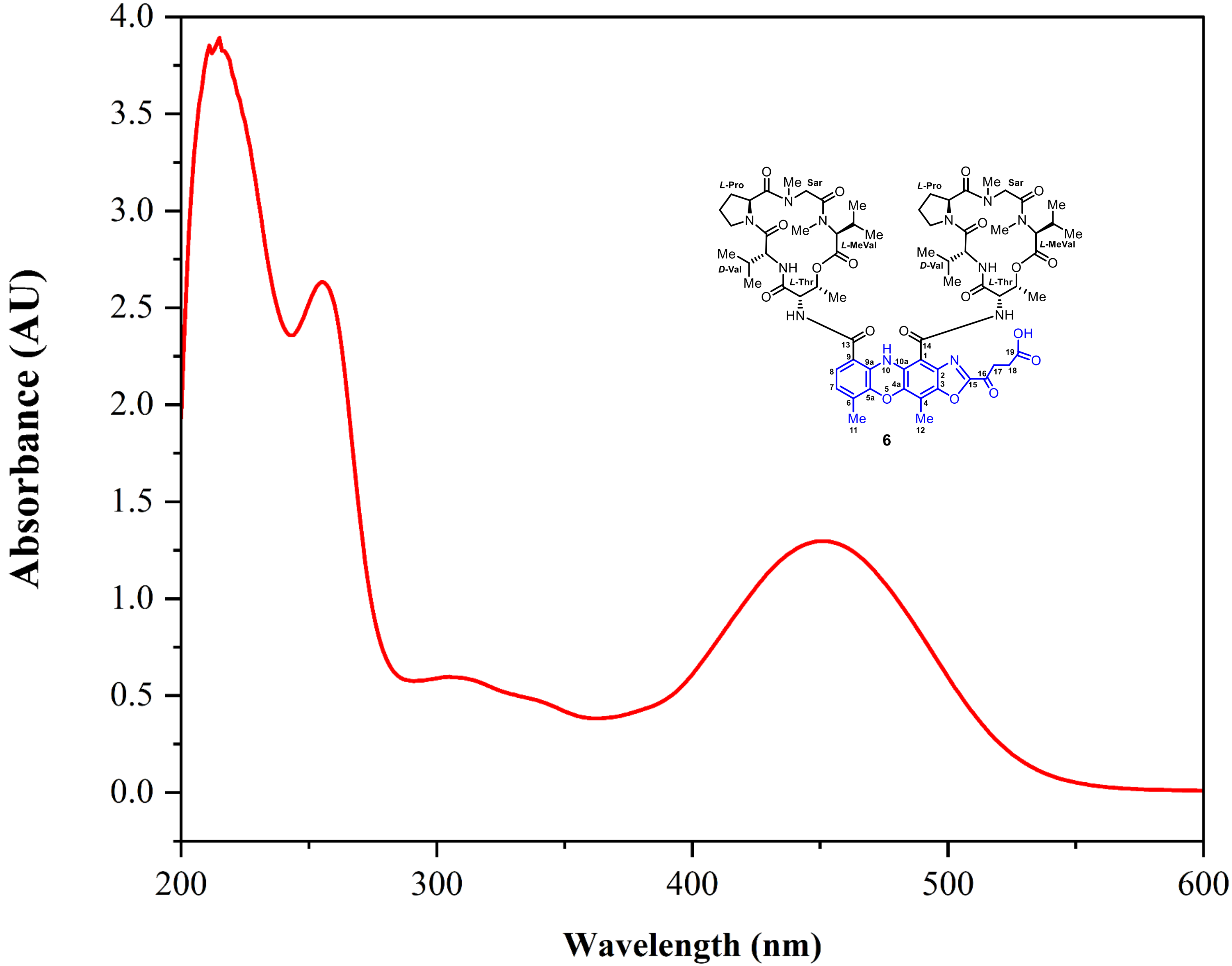

Figure S58. HR-ESIMS for compound **6**

Mass Spectrum SmartFormula Report

Analysis Info

Analysis Name	D:\Data\MS\data\202505\panghe_S-26-28-1_pos_41_01_19980.d	Acquisition Date	5/29/2025 5:47:30 PM
Method	LC_Direct Infusion_pos_100-1000mz.m	Operator	SCSIO
Sample Name	panghe_S-26-28-1_pos	Instrument	maXis 255552.00029
Comment			

Acquisition Parameter

Source Type	ESI	Ion Polarity	Positive	Set Nebulizer	0.4 Bar
Focus	Active	Set Capillary	4500 V	Set Dry Heater	180 °C
Scan Begin	100 m/z	Set End Plate Offset	-500 V	Set Dry Gas	4.0 l/min
Scan End	2000 m/z	Set Charging Voltage	0 V	Set Divert Valve	Waste
		Set Corona	0 nA	Set APCI Heater	0 °C

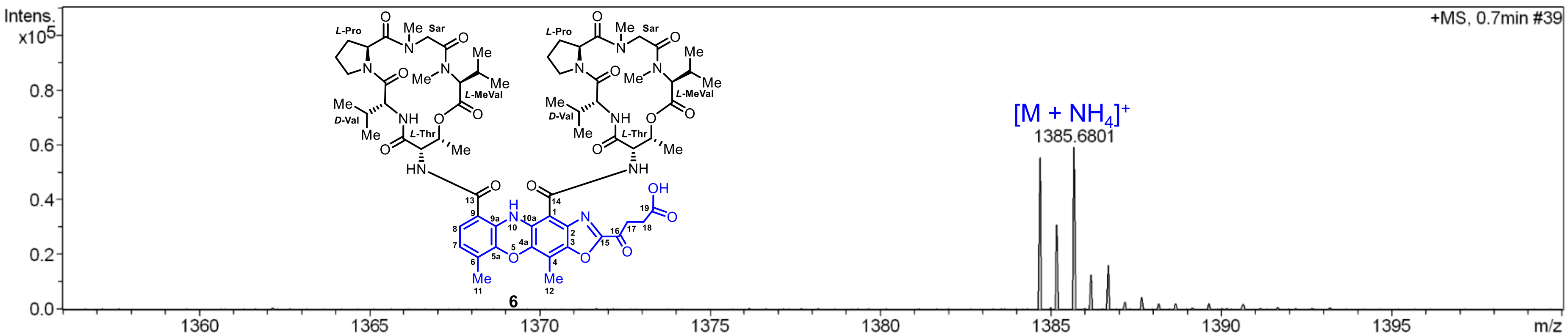

Meas. m/z	#	Ion Formula	Score	m/z	err [ppm]	err [mDa]	mSigma	rdb	e⁻ Conf	N-Rule
1367.6523	1	C67H91N12O19	100.00	1367.6518	0.4	0.5	546.3	28.5	even	ok
1384.6774	1	C67H94N13O19	100.00	1384.6783	-0.7	-0.9	96.5	27.5	even	ok
1389.6358	1	C67H90N12NaO19	100.00	1389.6337	-1.5	-2.1	74.3	28.5	even	ok

Figure S59. ^{1}H NMR (600 MHz) spectrum of compound **6** in DMSO-d_6

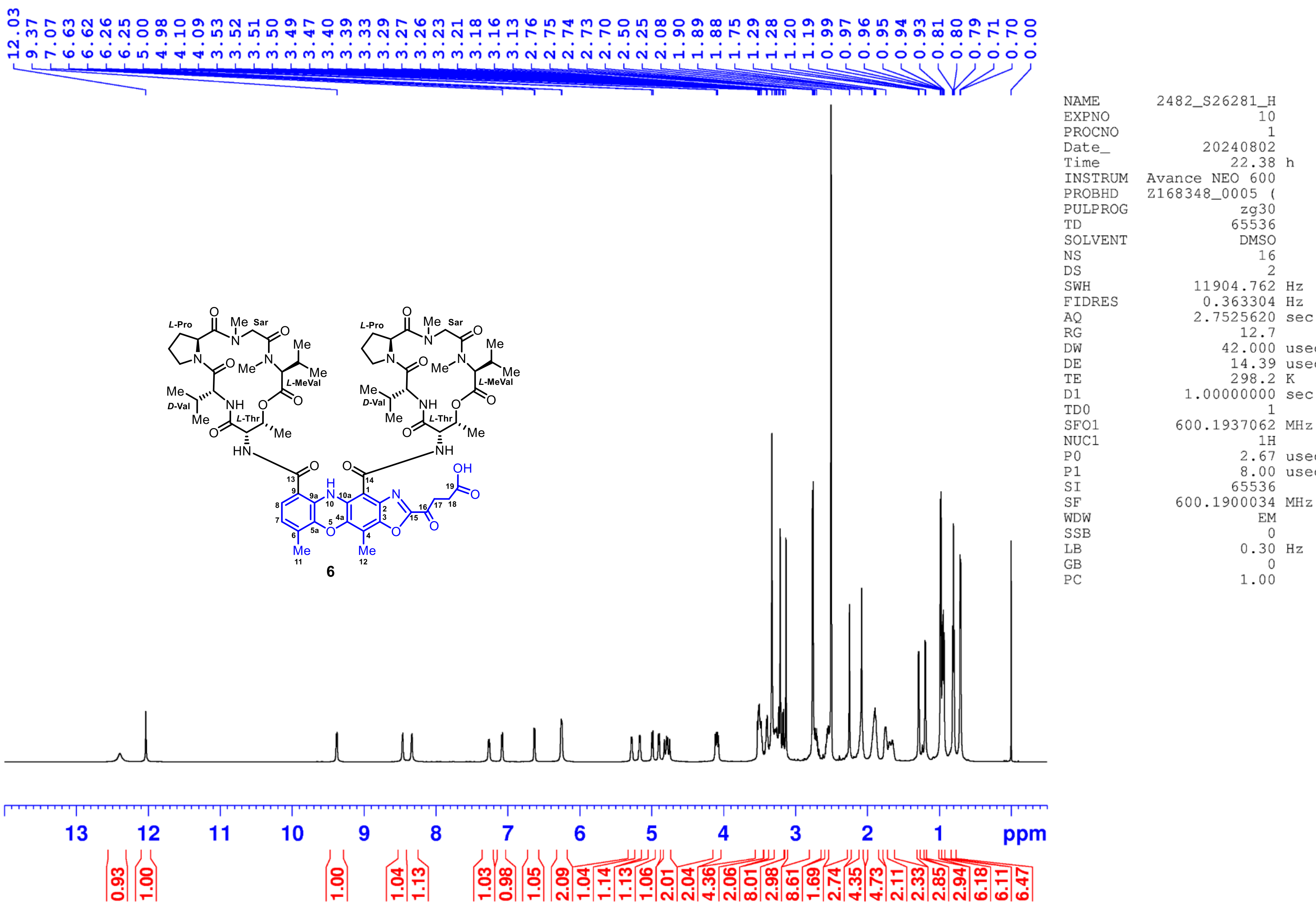

Figure S59-continue

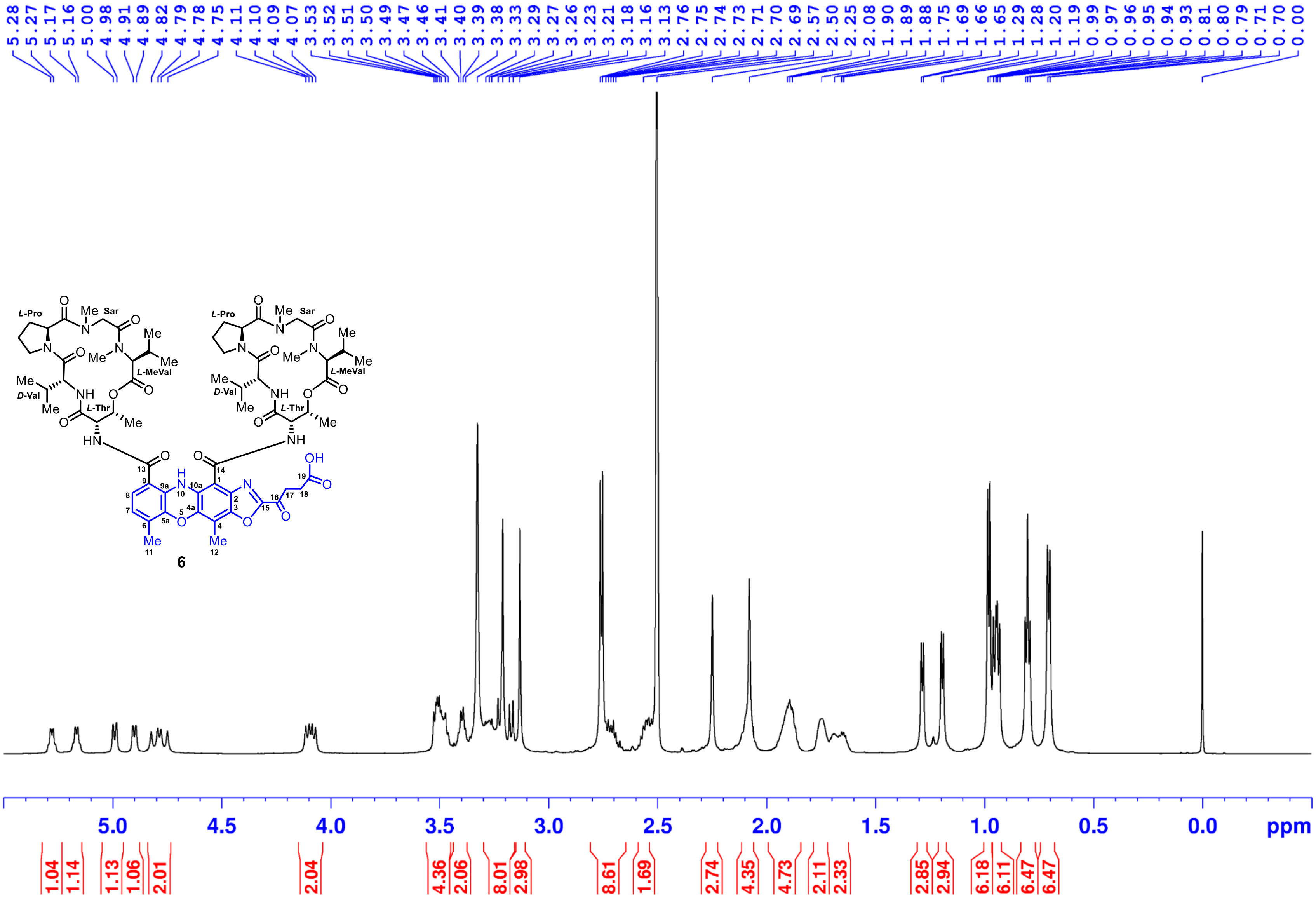

Figure S59-continue

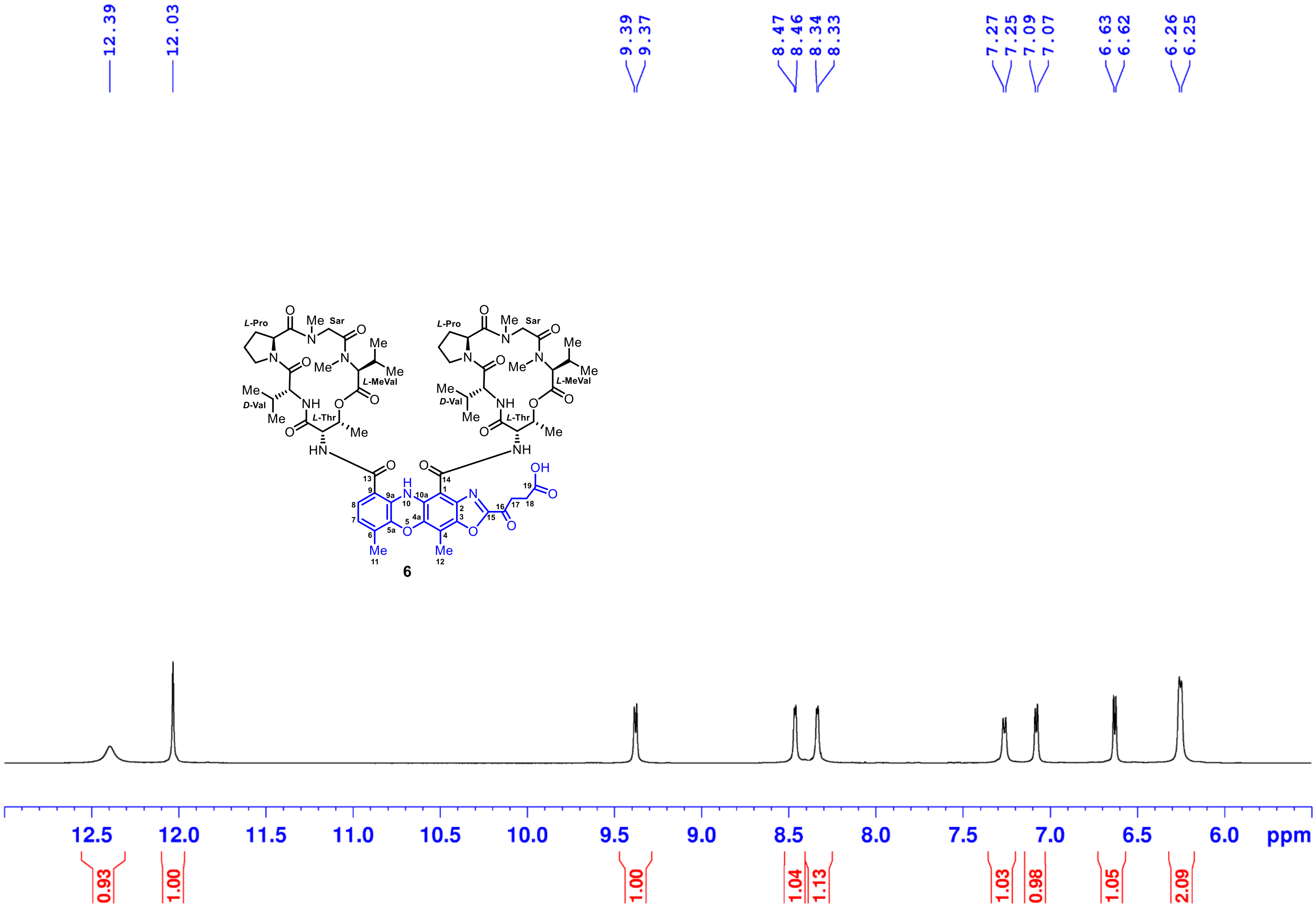

Figure S60. ^{13}C NMR (150 MHz) spectrum of compound **6** in DMSO-d_6

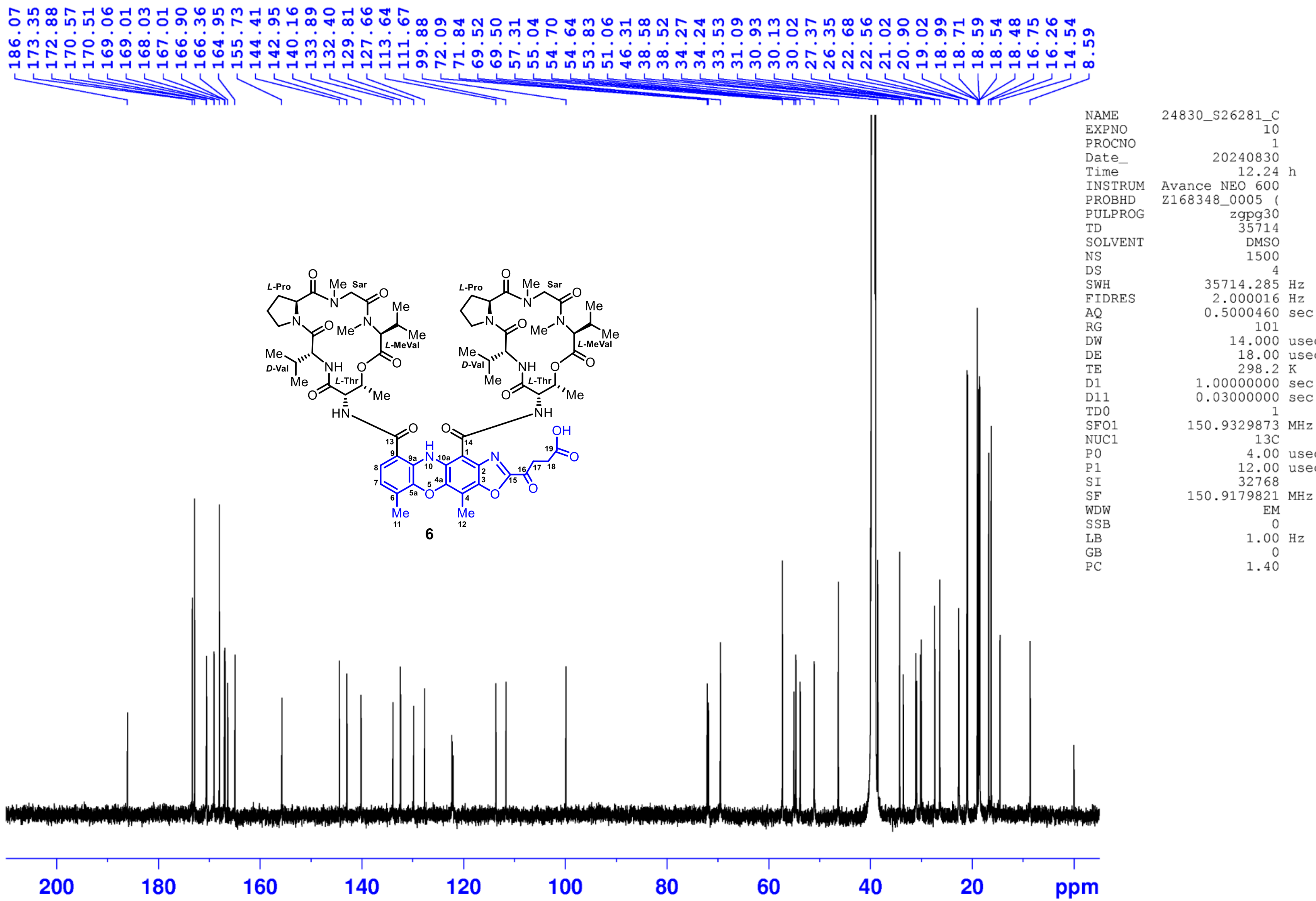

Figure S60-continue

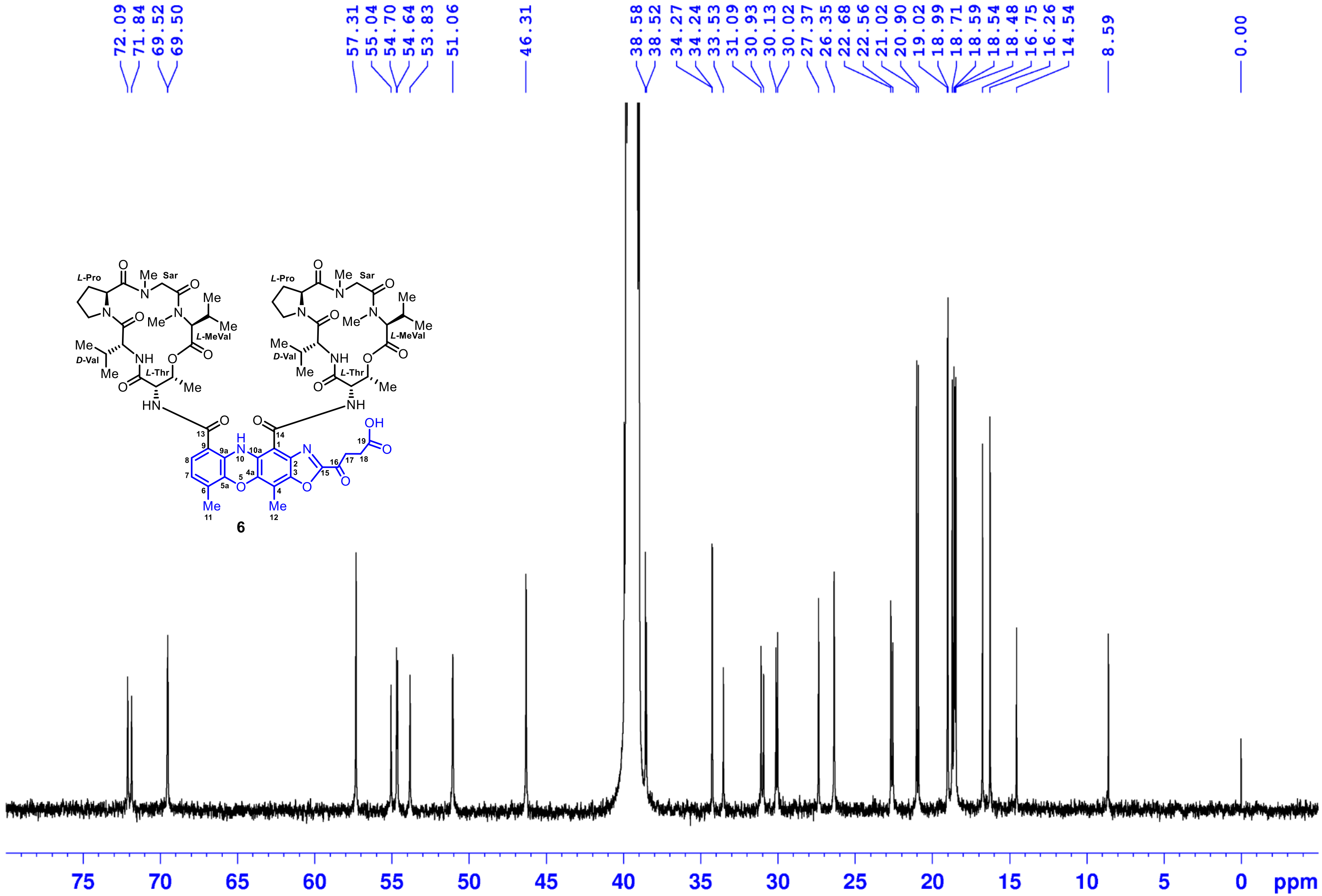

Figure S60-continue

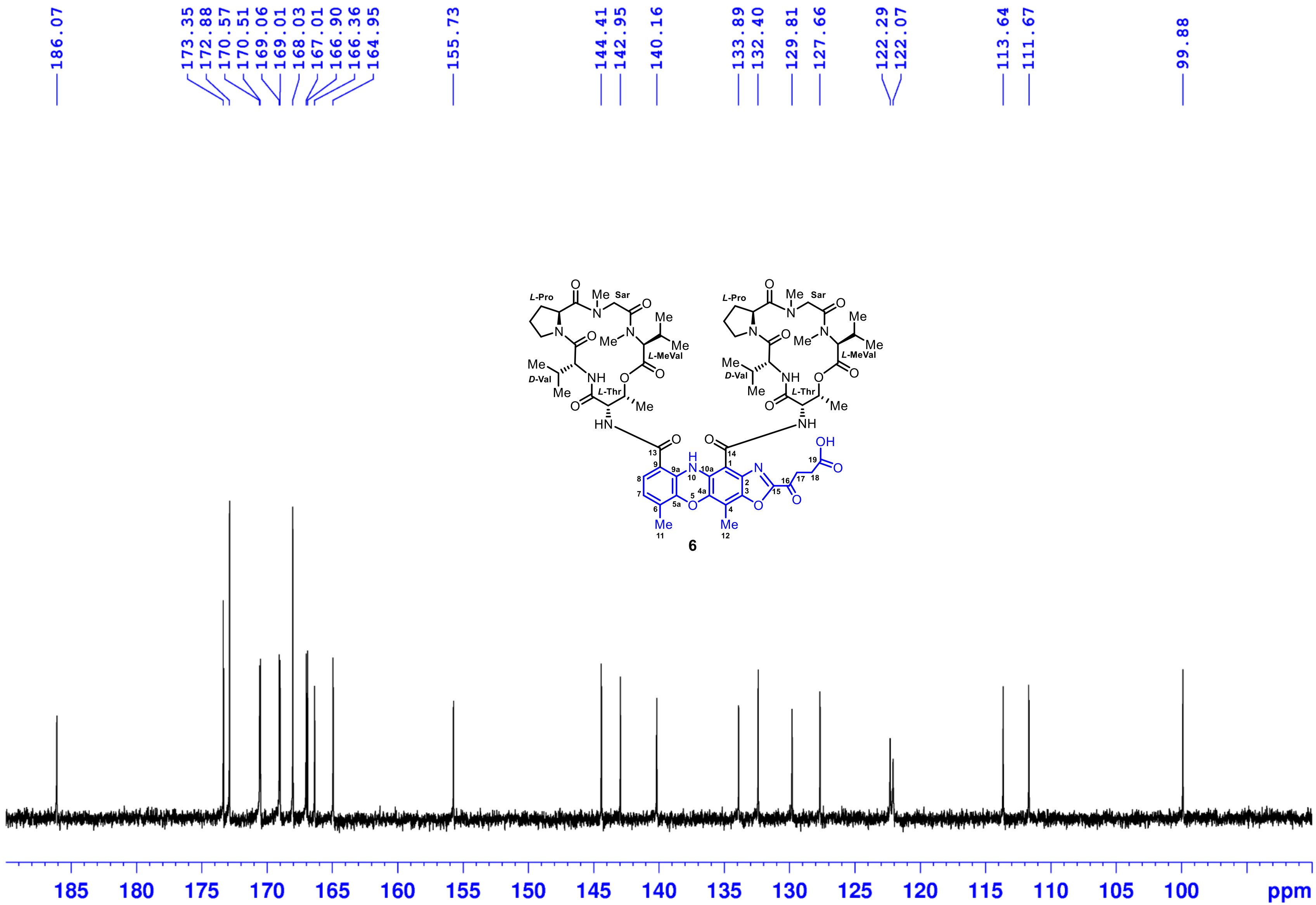

Figure S61. DEPT135 (150 MHz) spectrum of compound **6** in DMSO-d_6

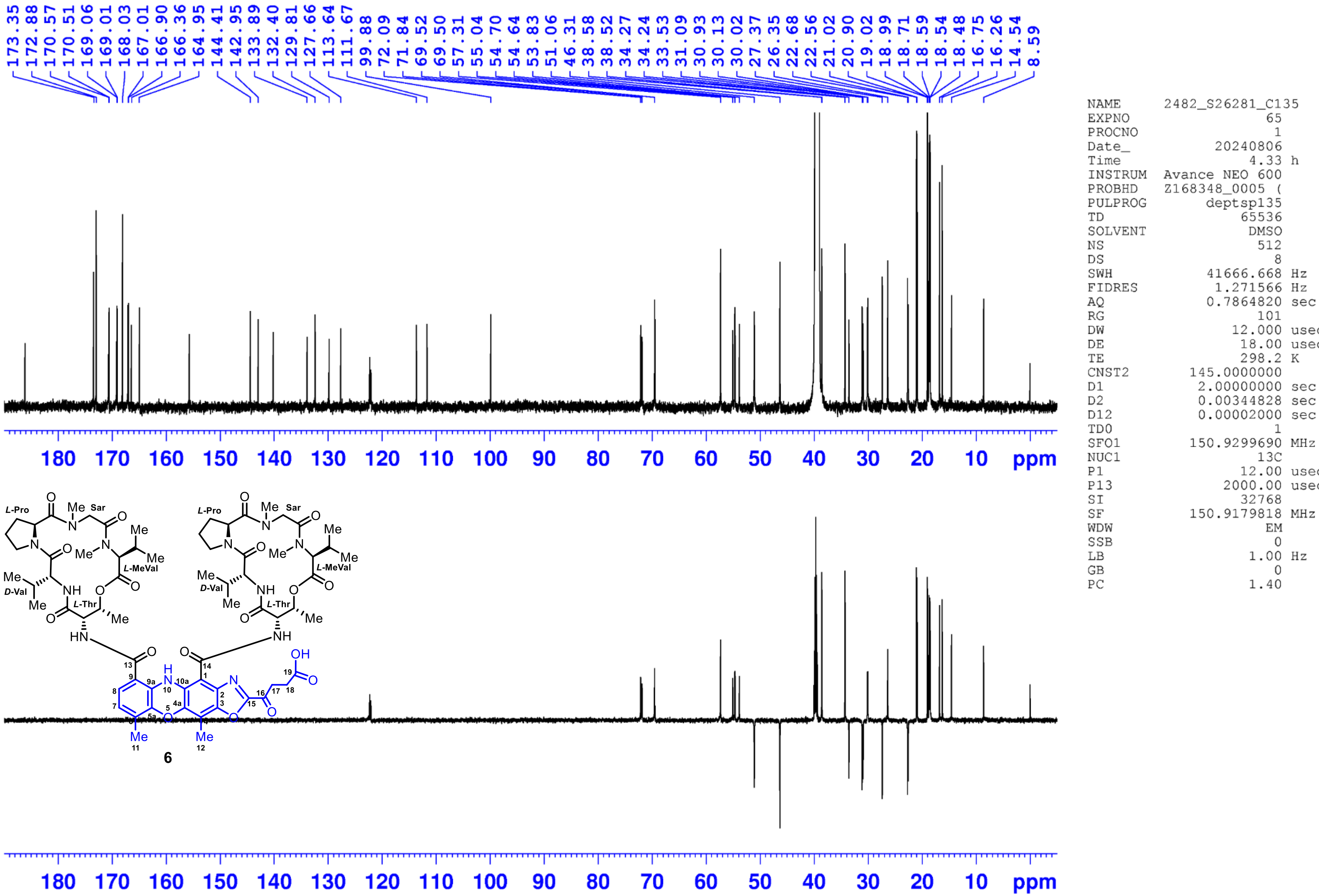

Figure S61-continue

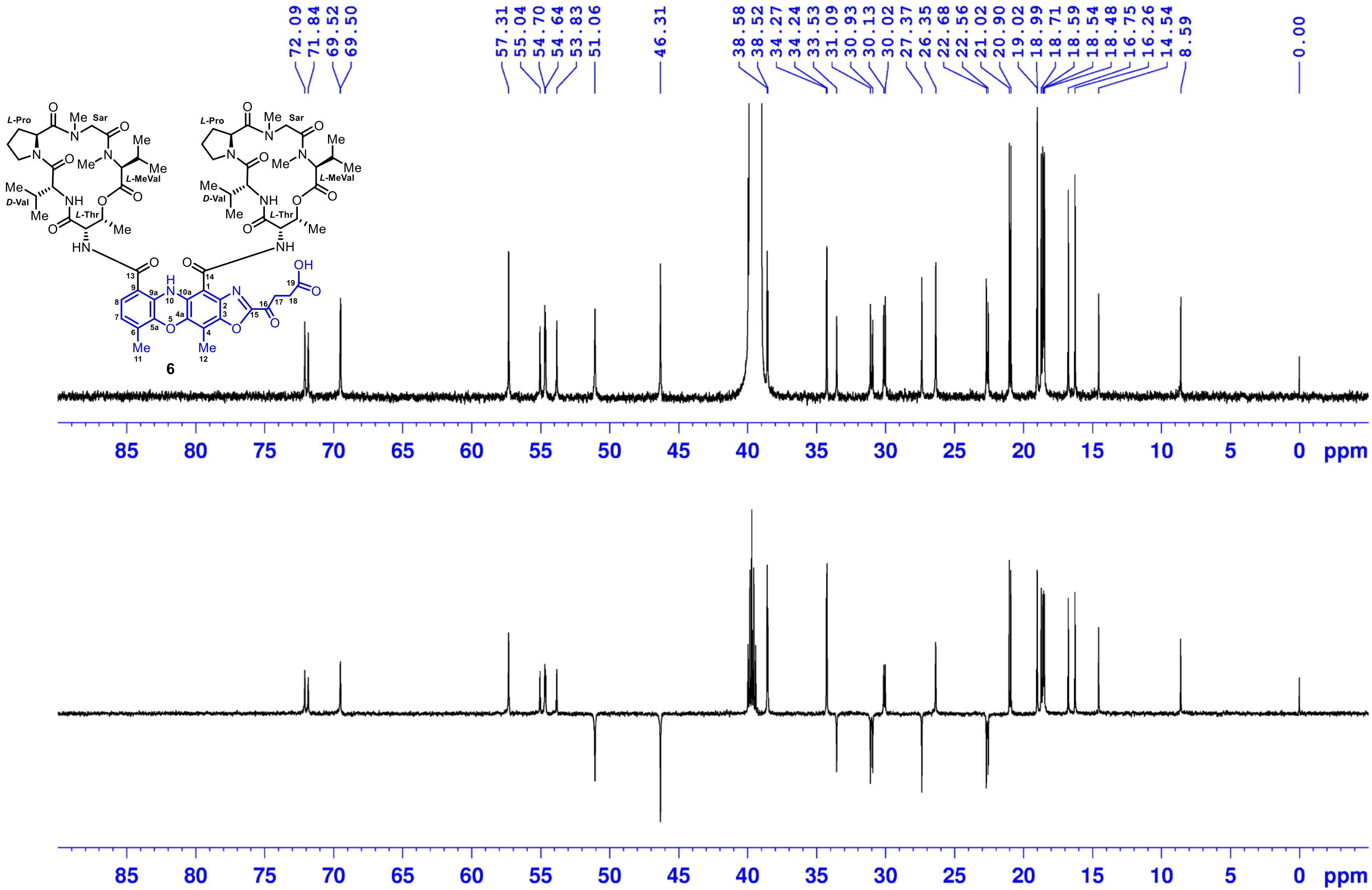

Figure S61-continue

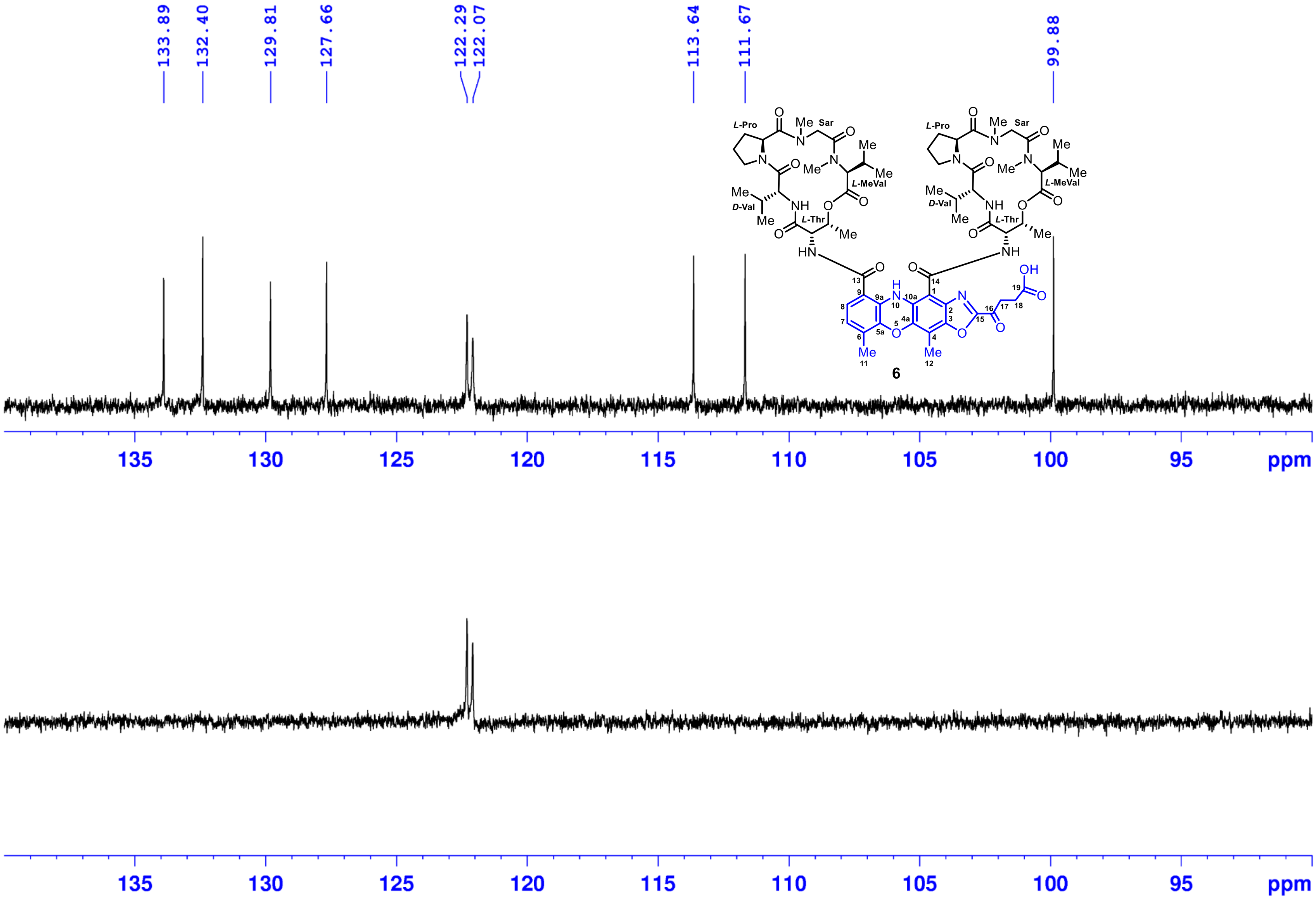

Figure S62. ^{1}H–^{1}H COSY (600 MHz) spectrum of compound **6** in DMSO-d_6

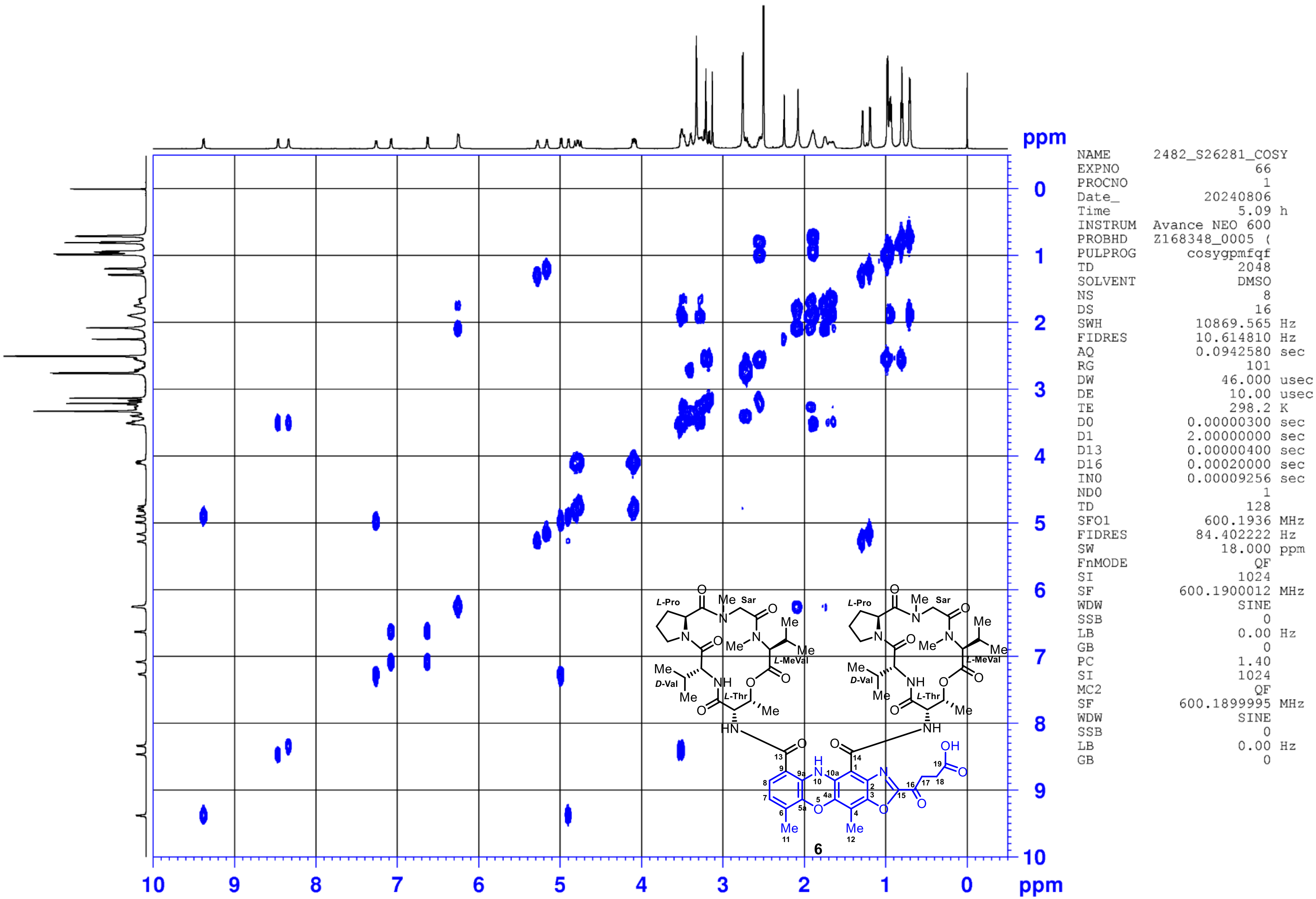

Figure S62-continue

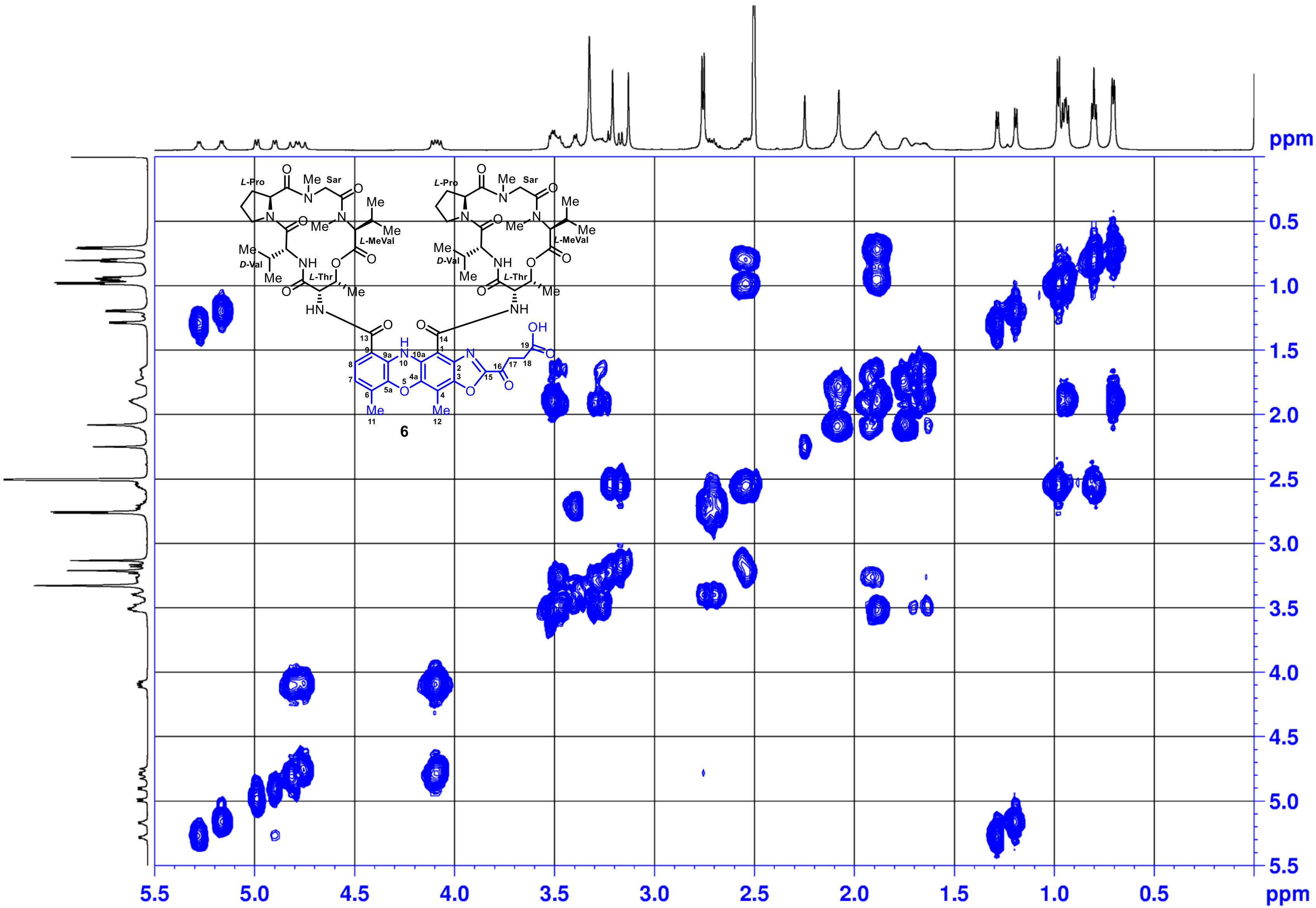

Figure S62-continue

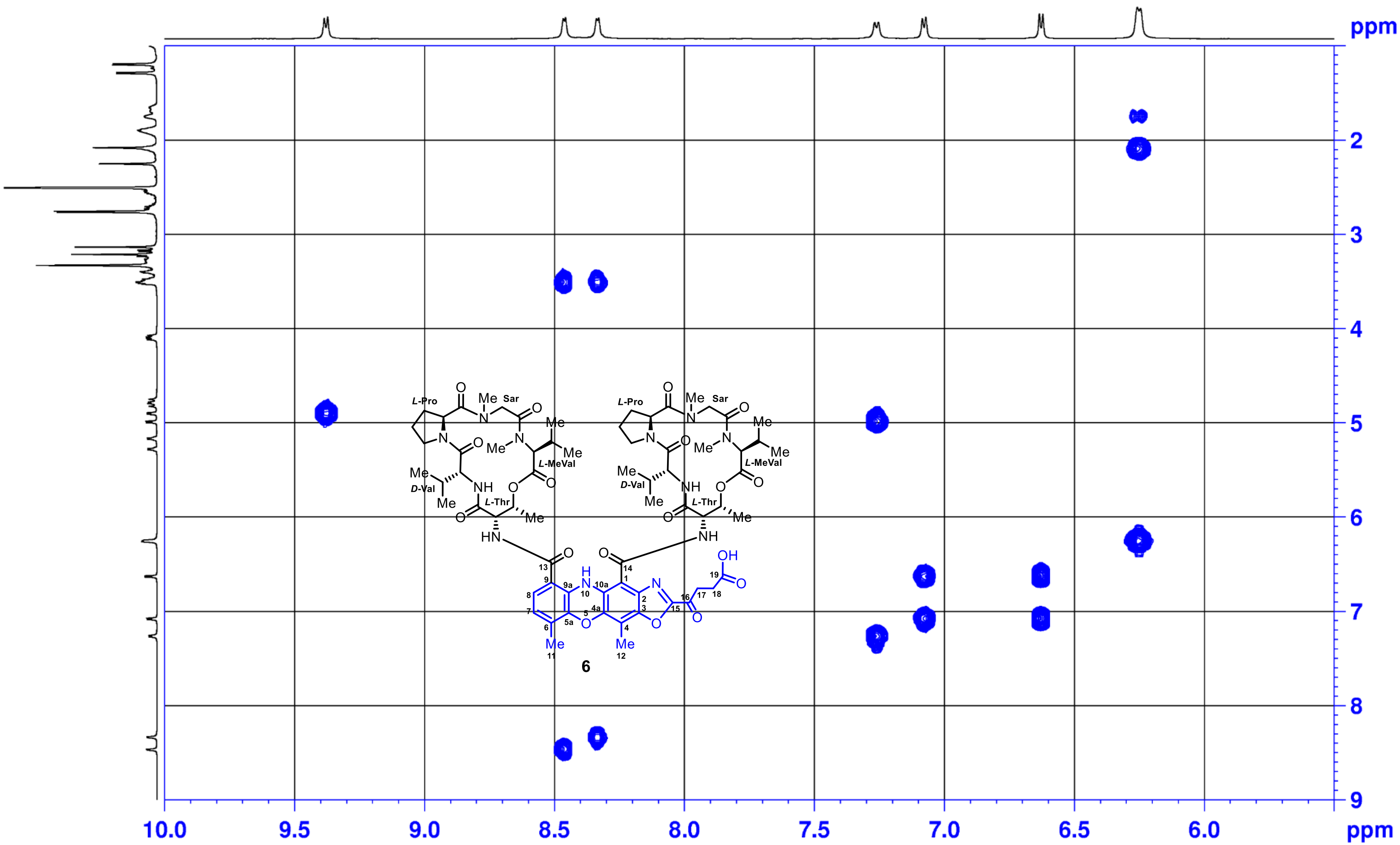

Figure S63. HSQC (600 MHz) spectrum of compound **6** in DMSO-d_6

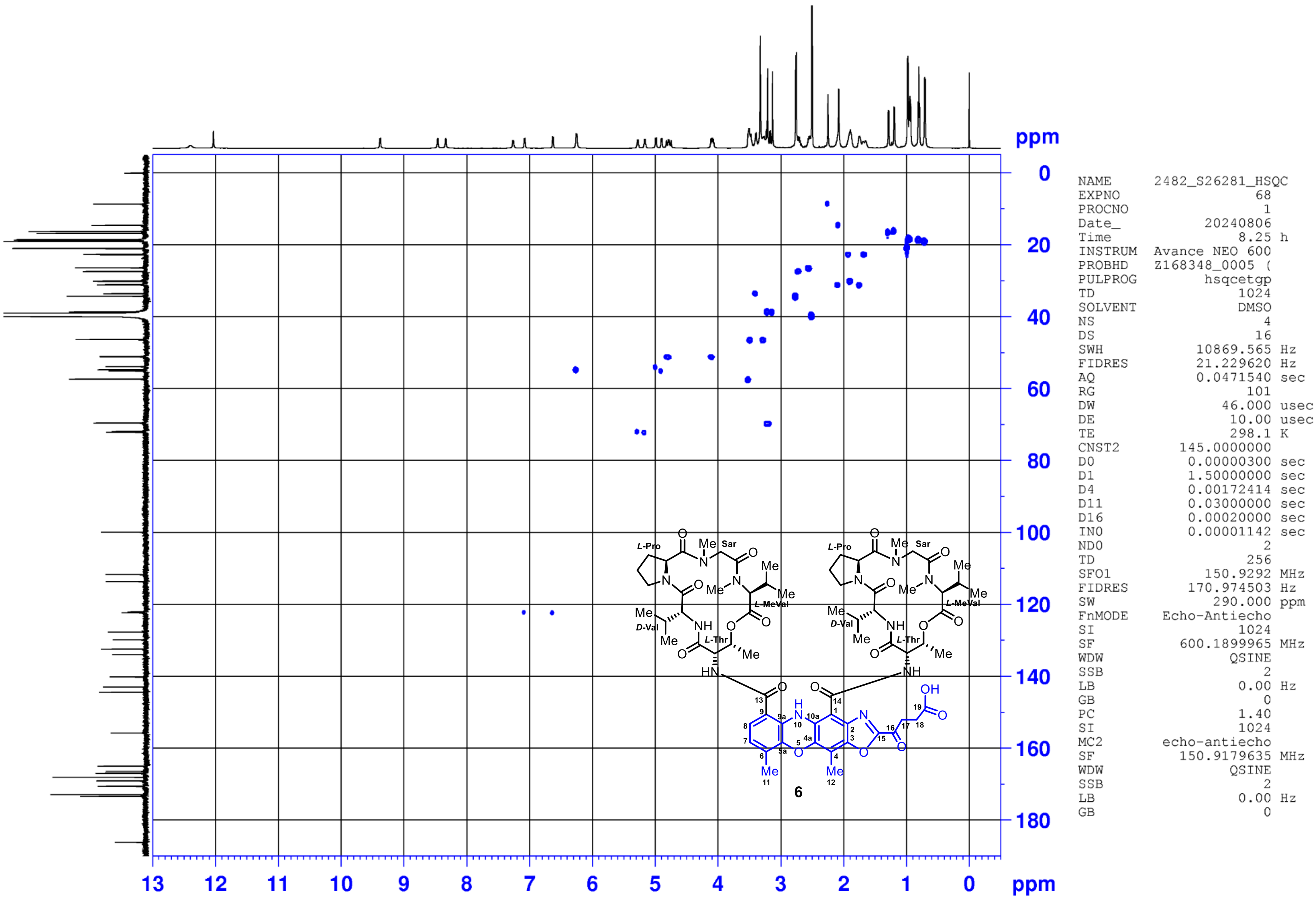

Figure S63-continue

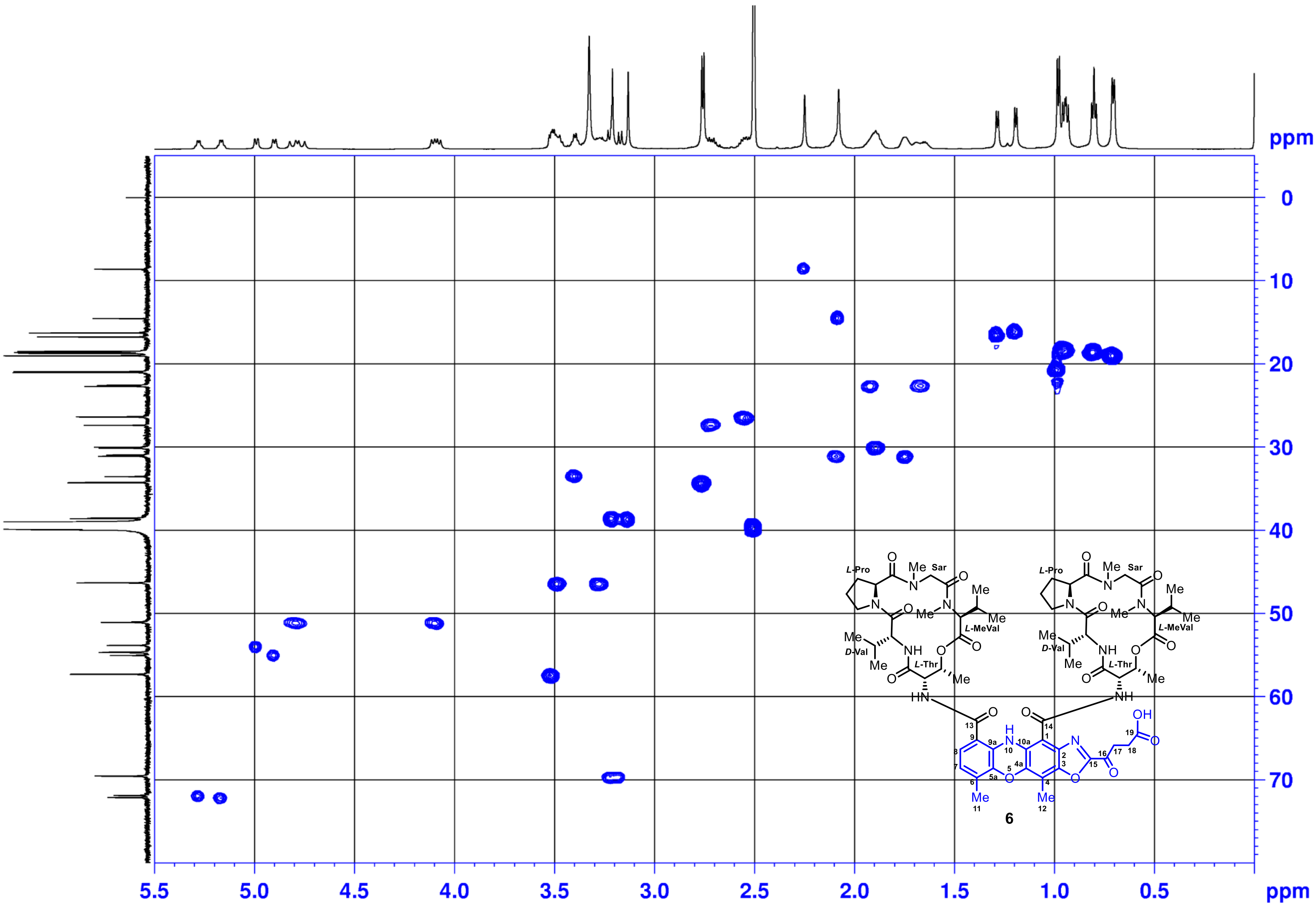

Figure S63-continue

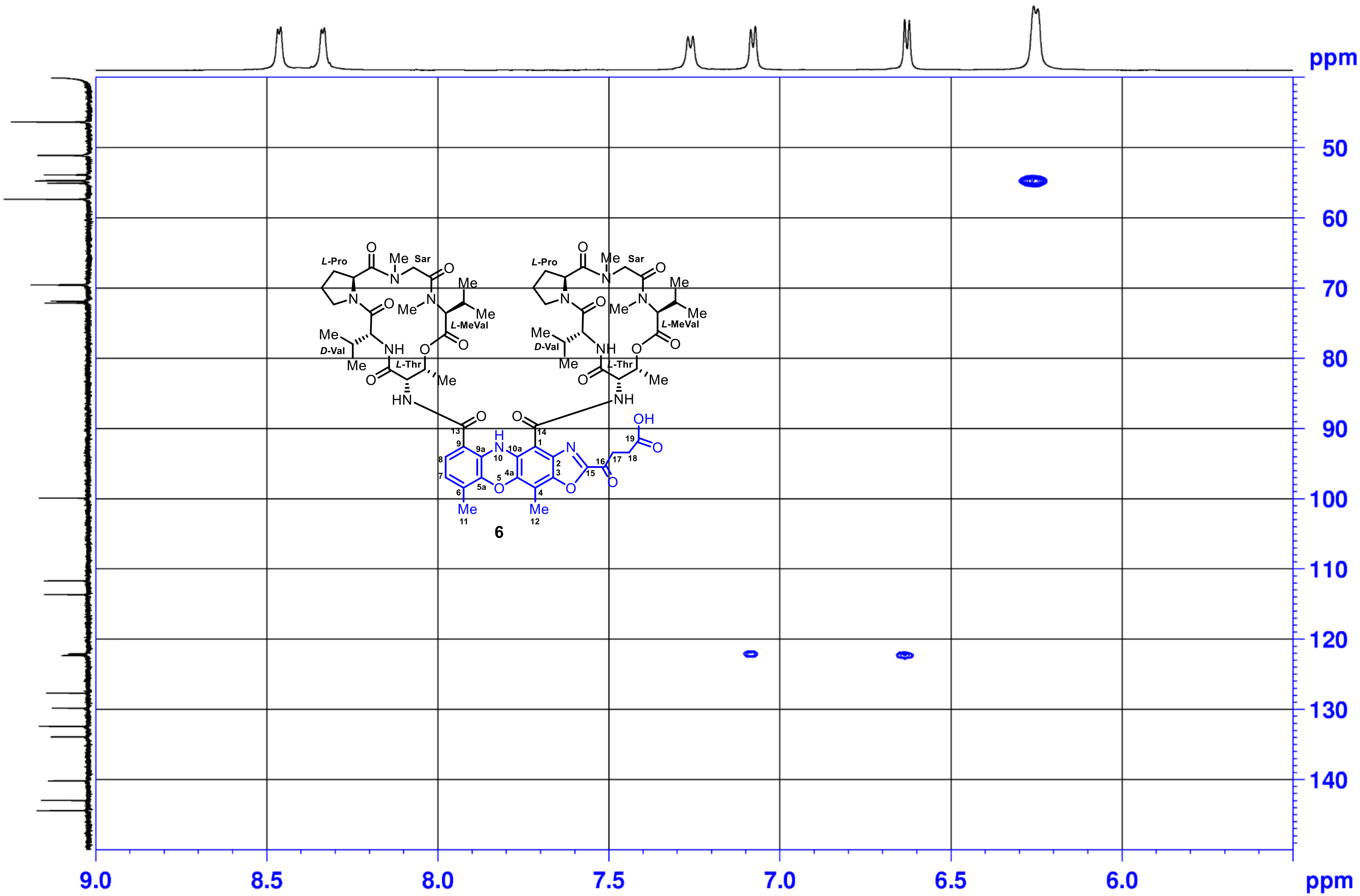

Figure S64. HMBC (600 MHz) spectrum of compound **6** in DMSO-d_6

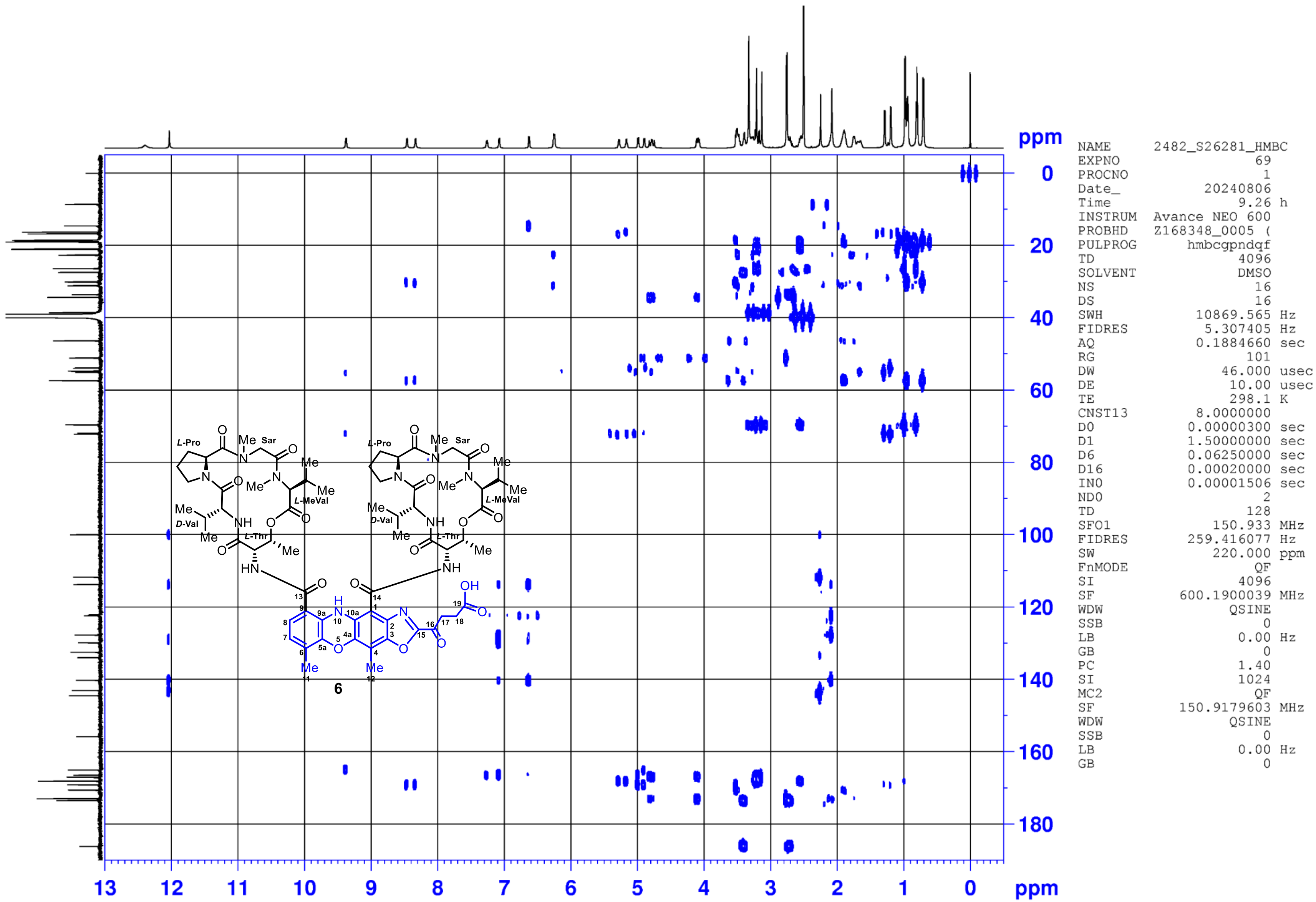

Figure S64-continue

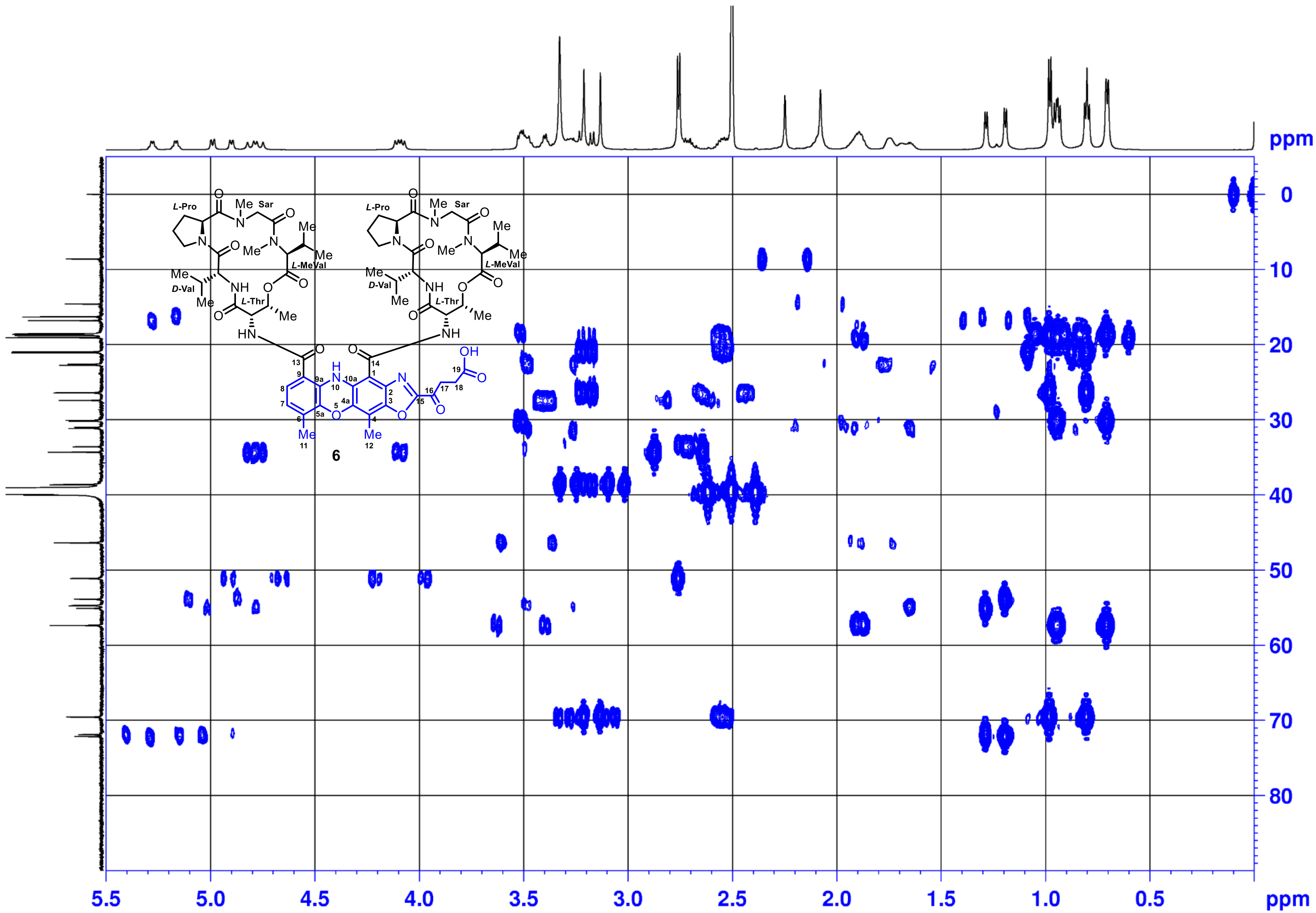

Figure S64-continue

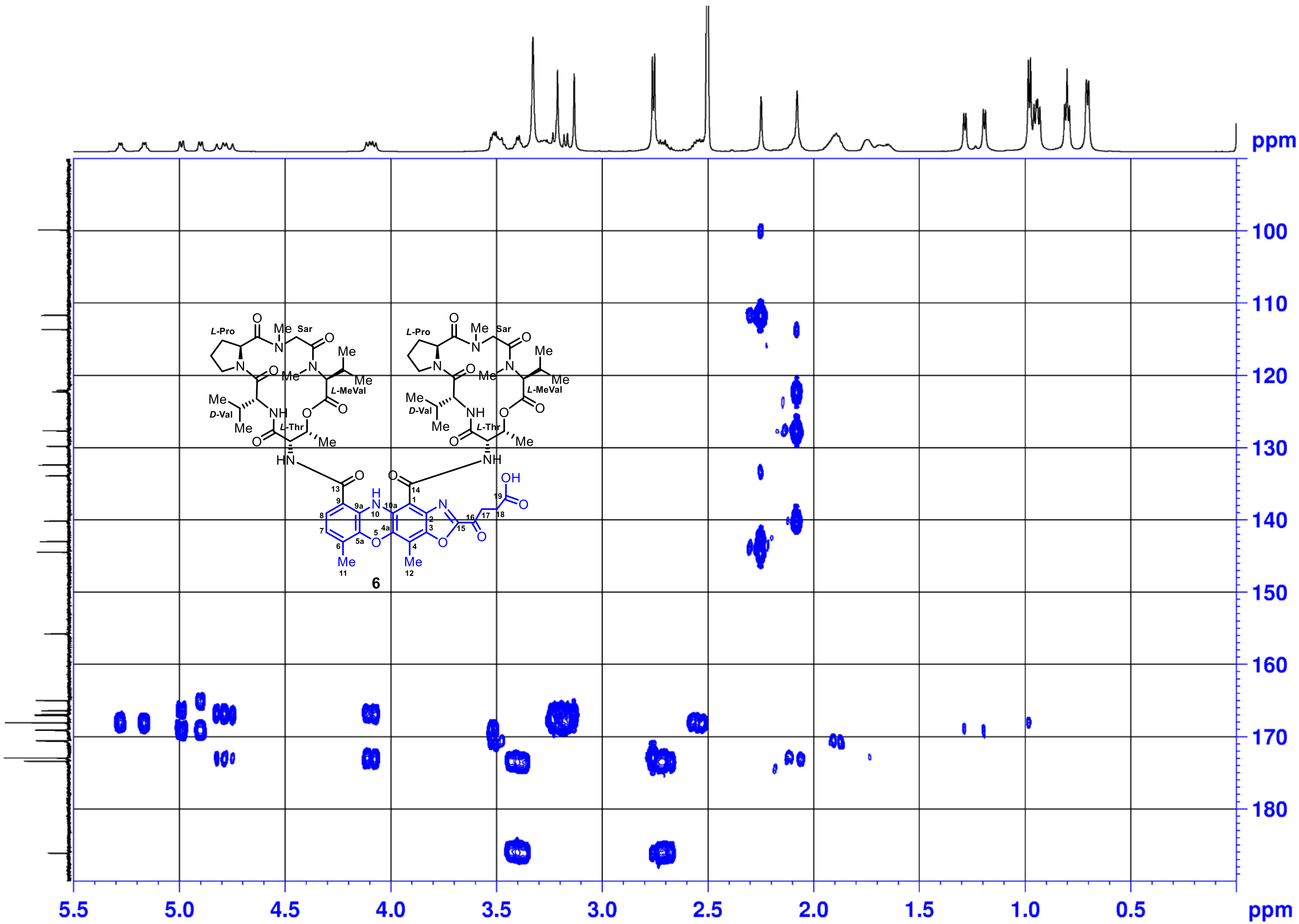

Figure S64-continue

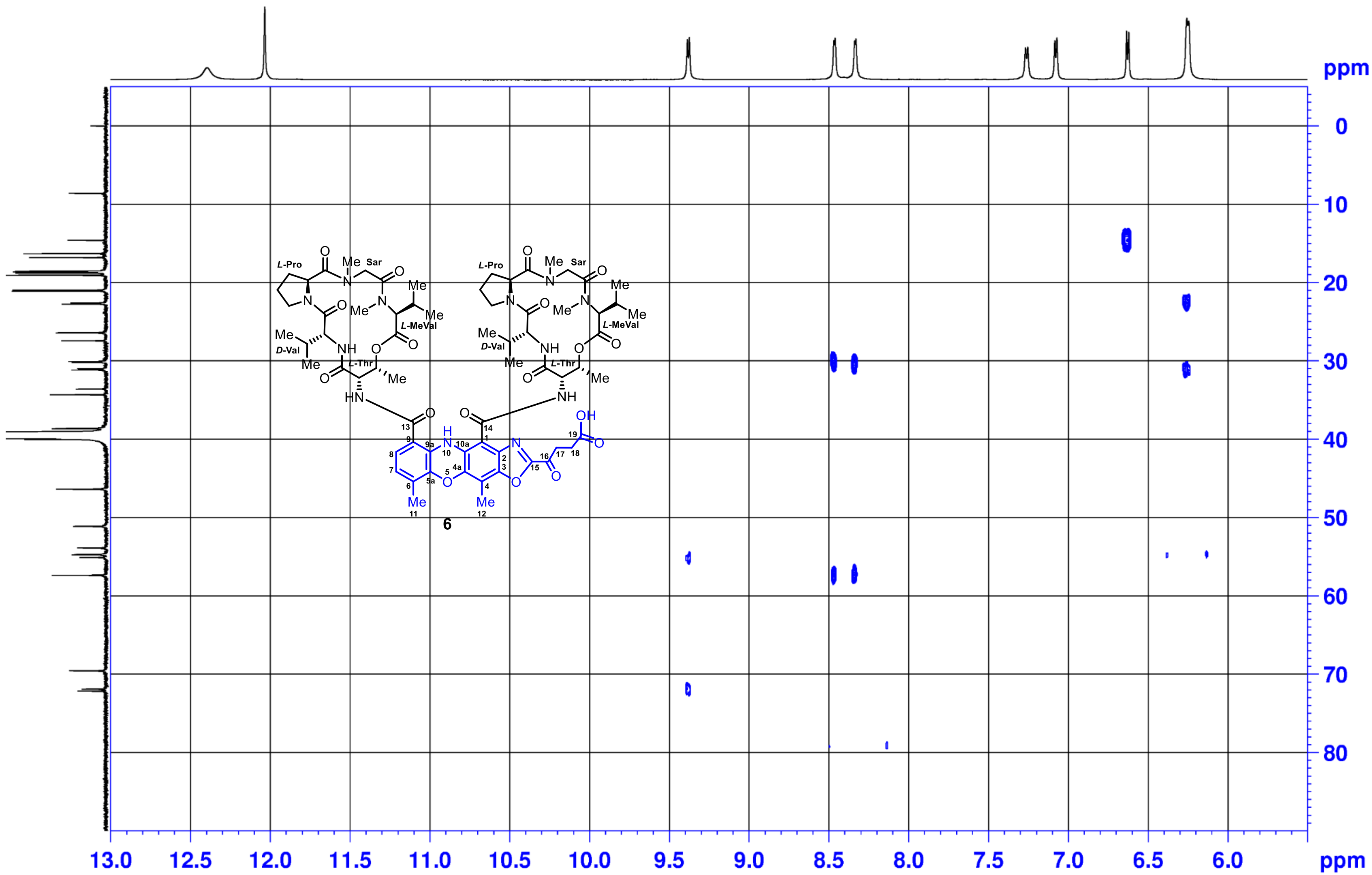

Figure S64-continue

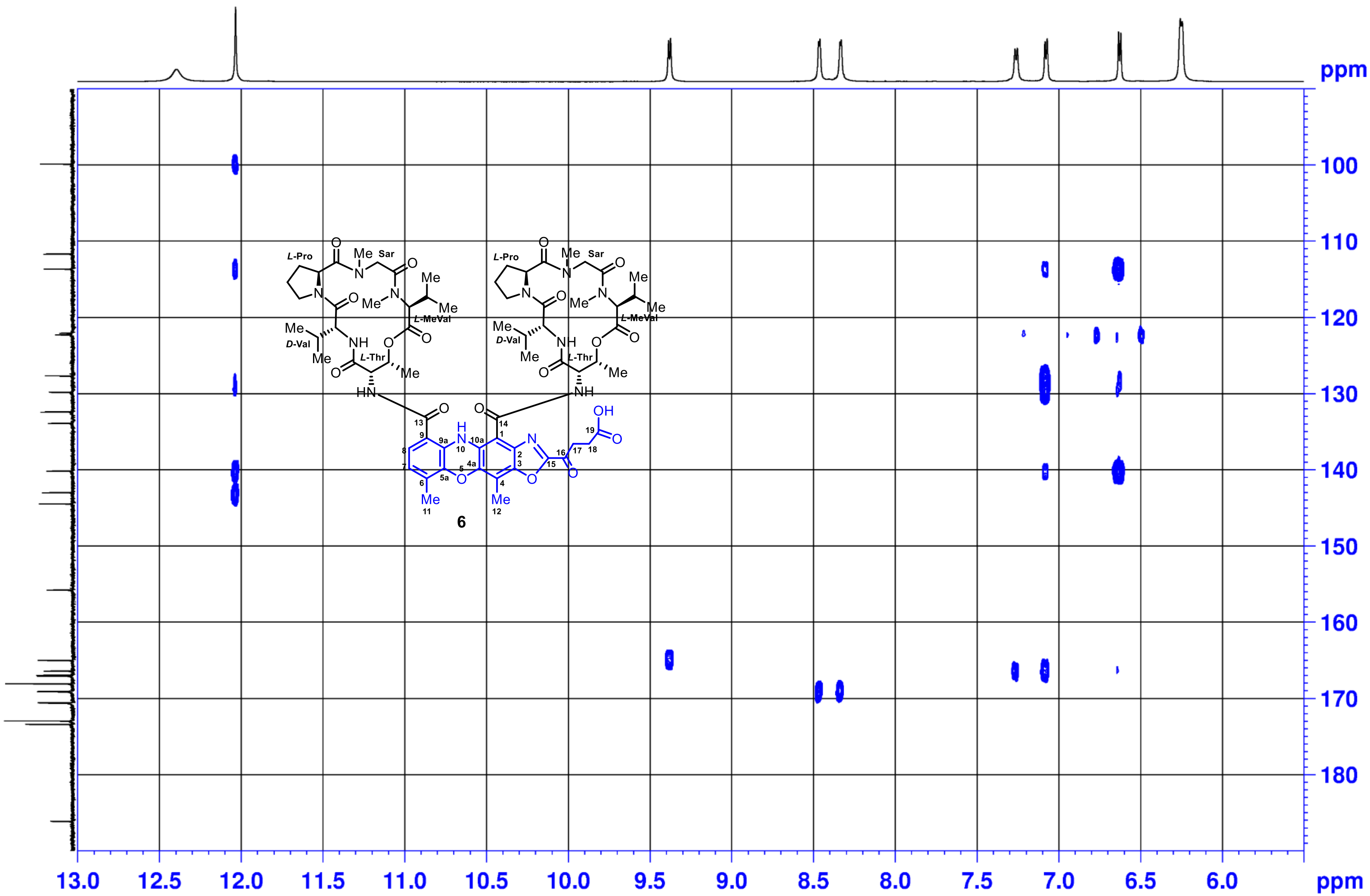

Figure S65. ROESY (600 MHz) spectrum of compound **6** in DMSO-d_6

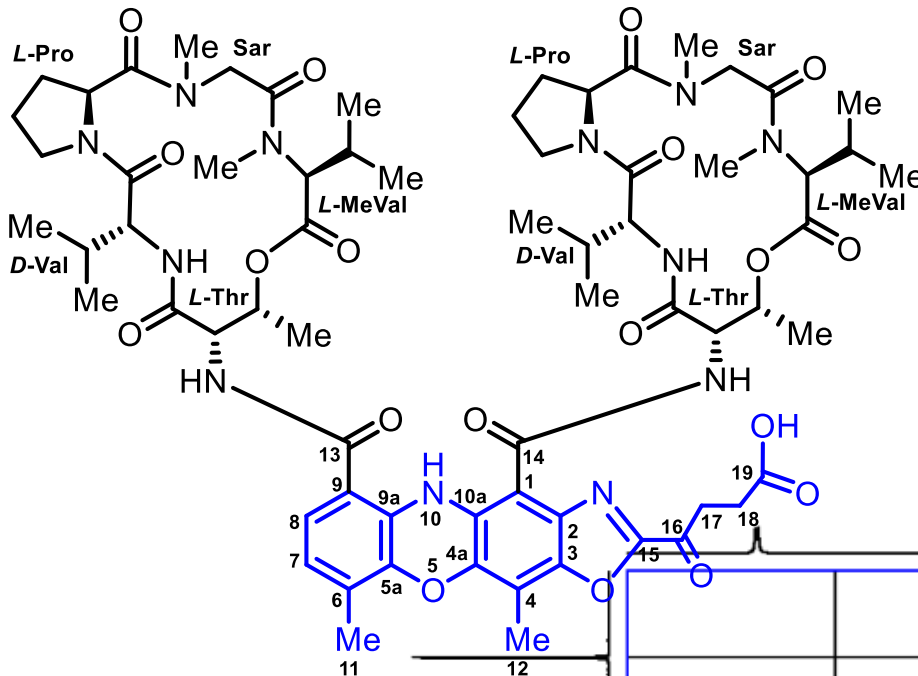

6

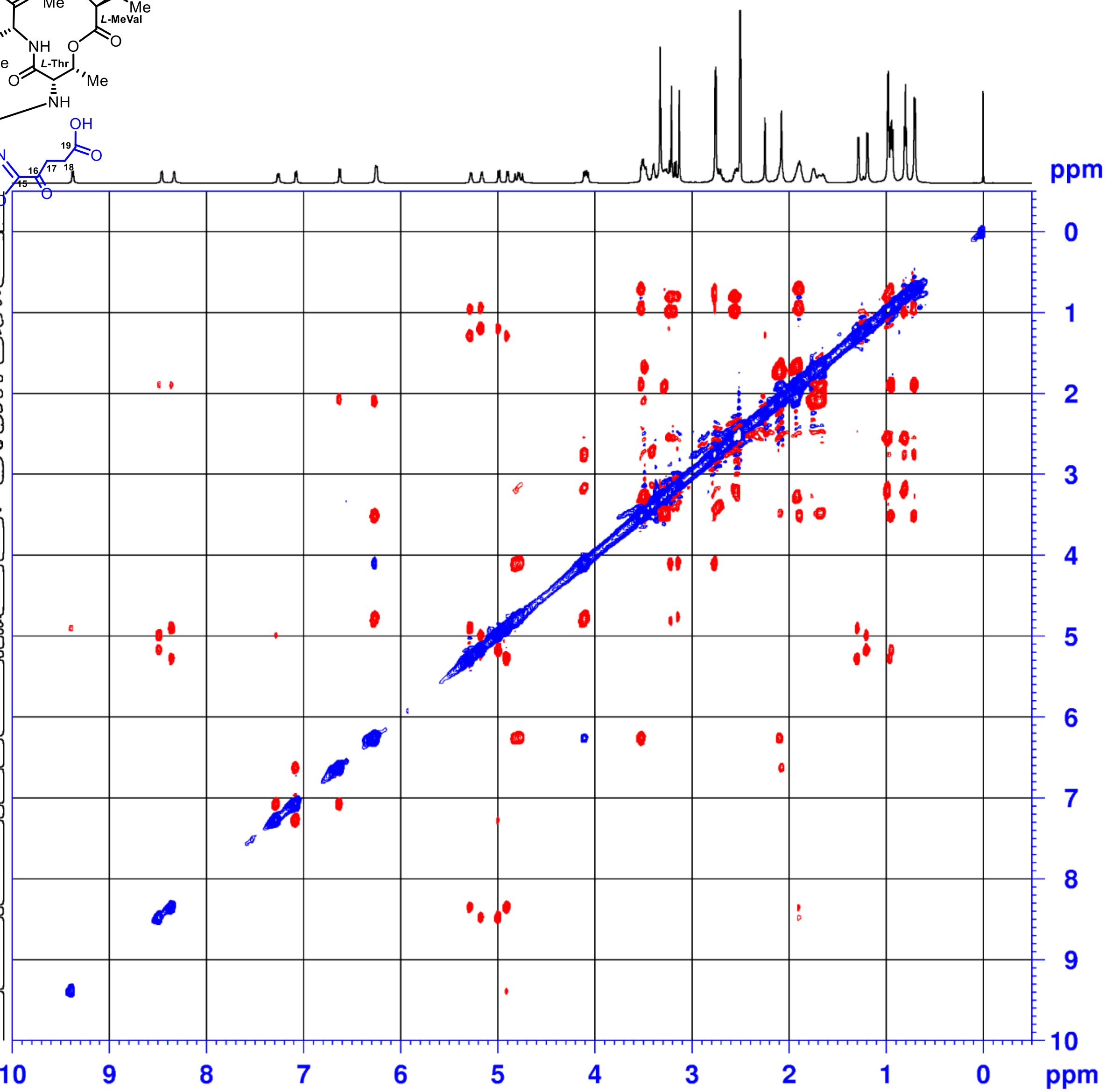

NAME	0526_S_26_28_1ROE
EXPNO	10
PROCNO	1
Date_	20250527
Time	5.27 h
INSTRUM	Avance NEO 600
PROBHD	Z154705_0139 (
PULPROG	roesyphpp.2
TD	2048
SOLVENT	DMSO
NS	8
DS	32
SWH	13888.889 Hz
FIDRES	13.563368 Hz
AQ	0.0737780 sec
RG	101
DW	36.000 usec
DE	6.50 usec
TE	293.5 K
D0	0.00002650 sec
D1	2.00000000 sec
D11	0.03000000 sec
D12	0.00002000 sec
IN0	0.00007373 sec
L4	556
P15	200000.00 usec
ND0	1
TD	256
SFO1	600.1928 MHz
FIDRES	52.985771 Hz
SW	22.600 ppm
FnMODE	States-TPPI
SI	1024
SF	600.1899934 MHz
WDW	QSINE
SSB	2
LB	0.00 Hz
GB	0
PC	1.00
SI	1024
MC2	States-TPPI
SF	600.1899950 MHz
WDW	QSINE
SSB	2
LB	0.00 Hz
GB	0

Figure S65-continue

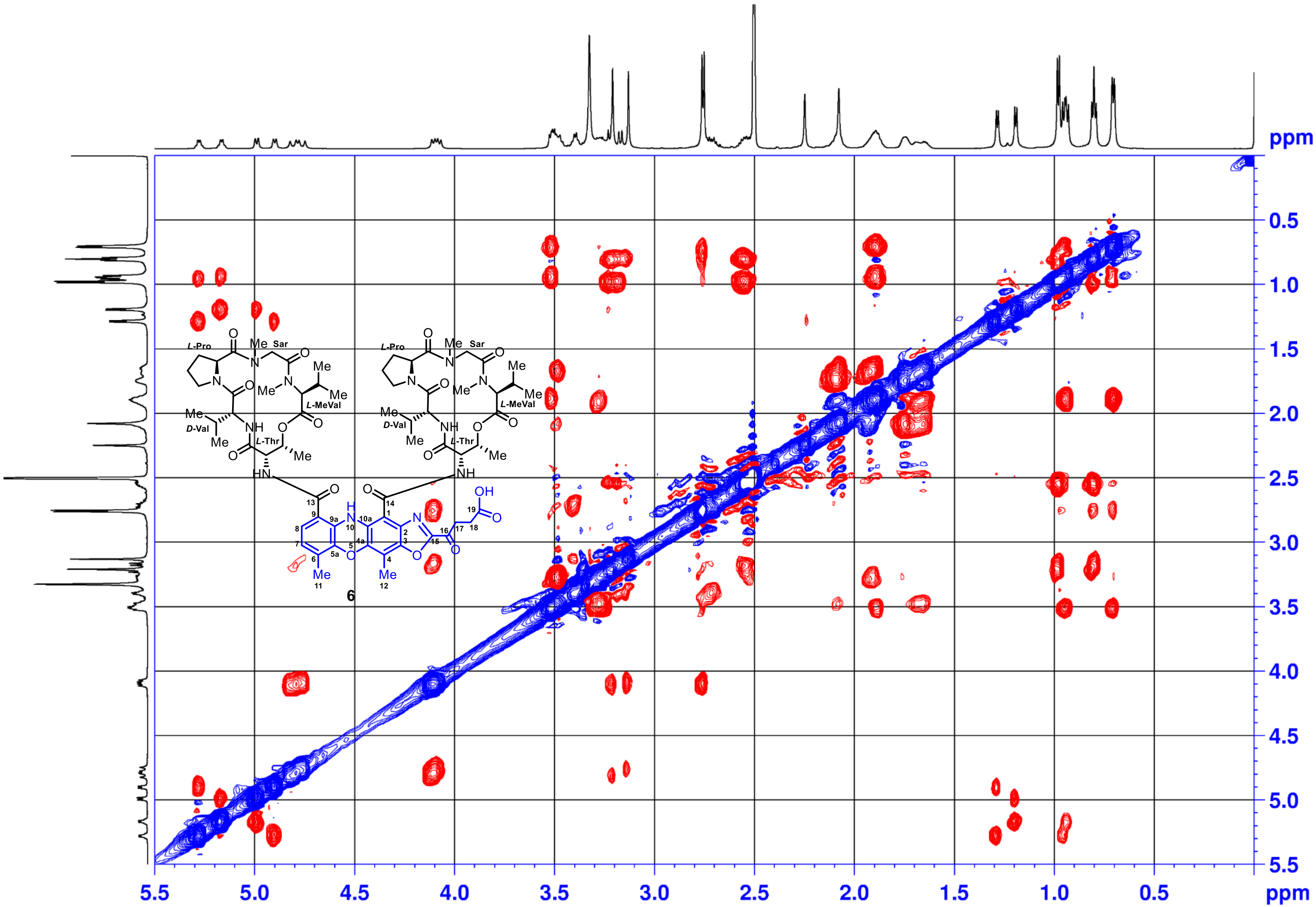

Figure S65-continue

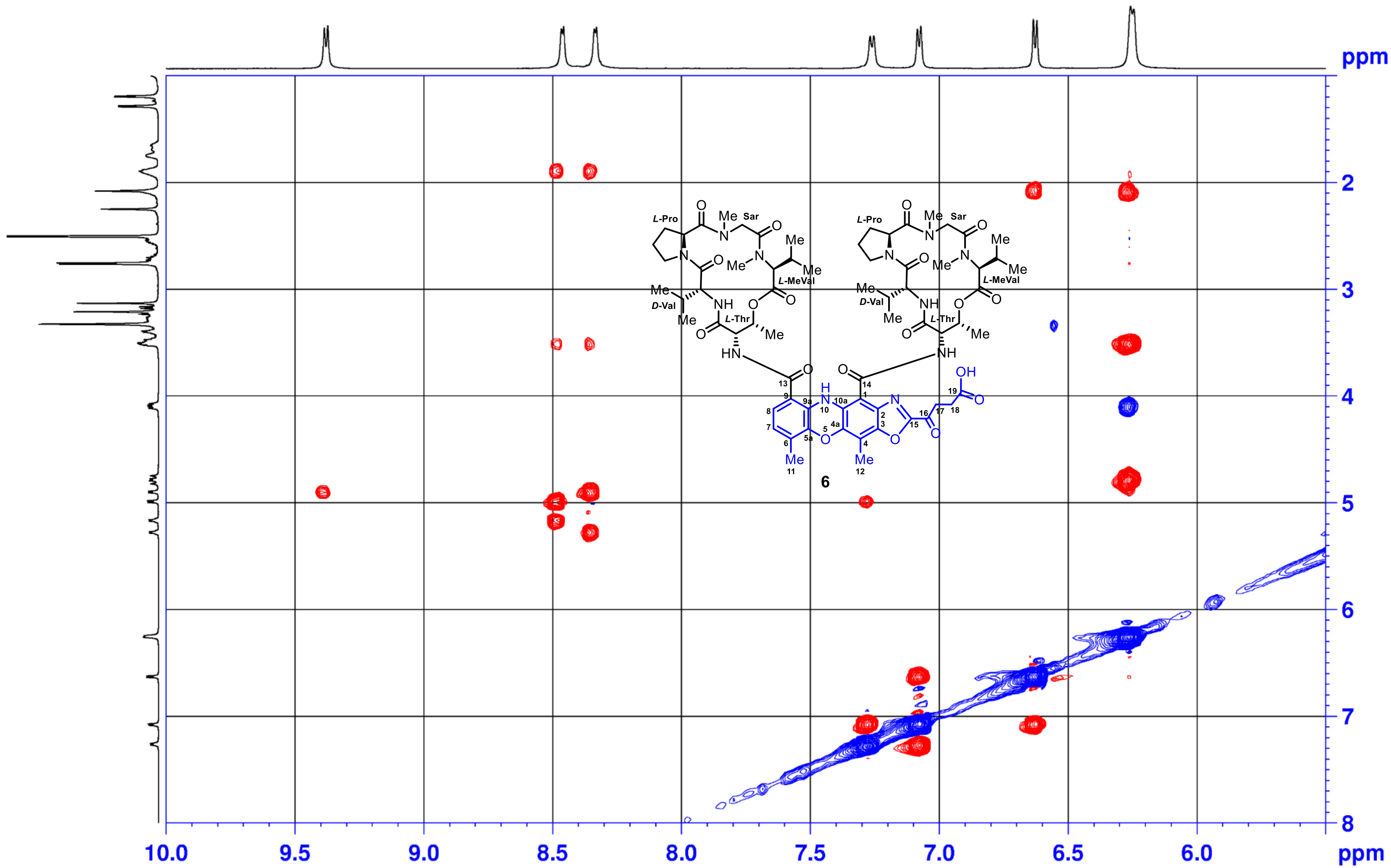

Figure S66. Marfey's analysis of compound **6** and standard amino acids

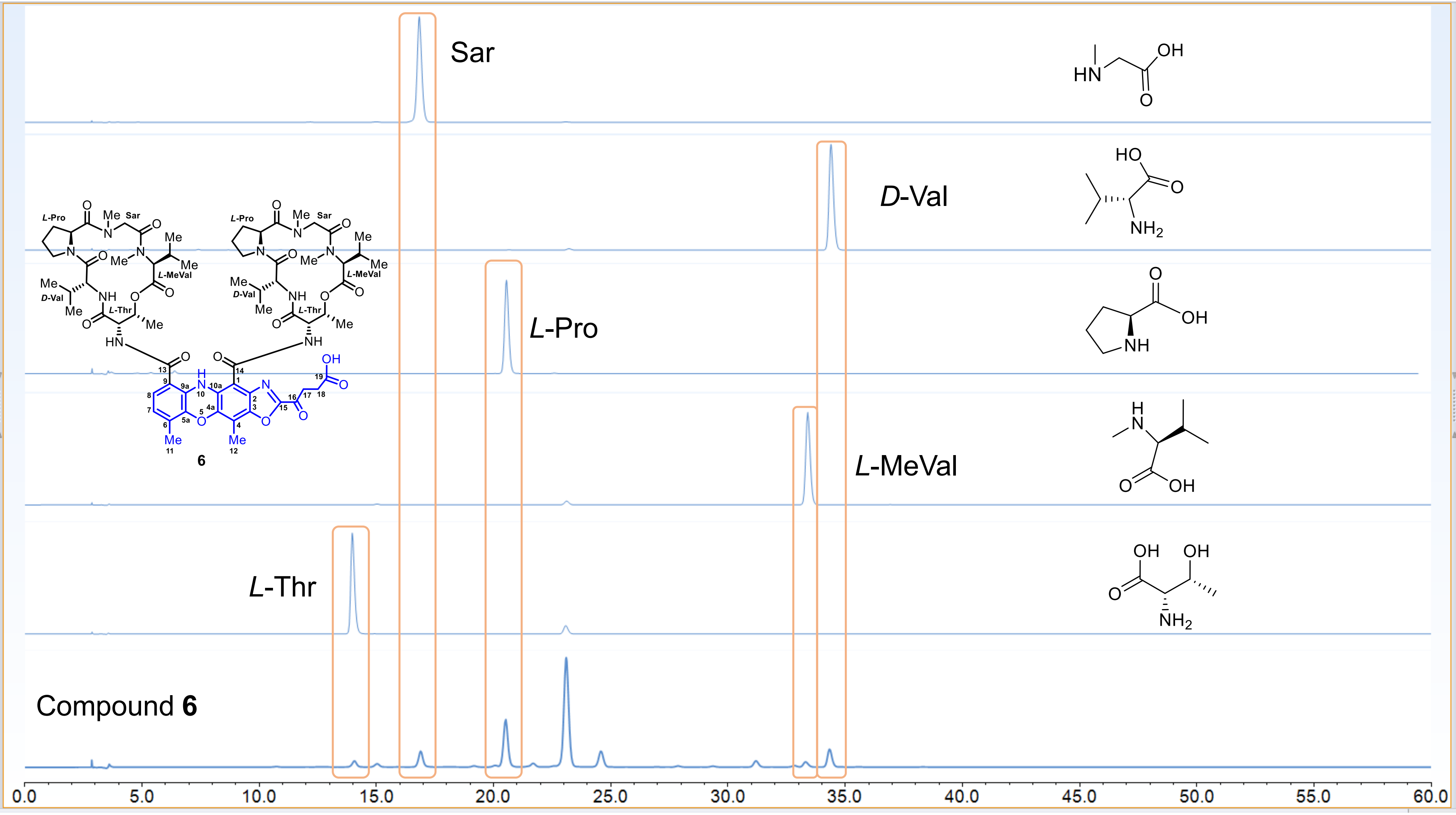

Figure S67. UV spectrum of compound **7**

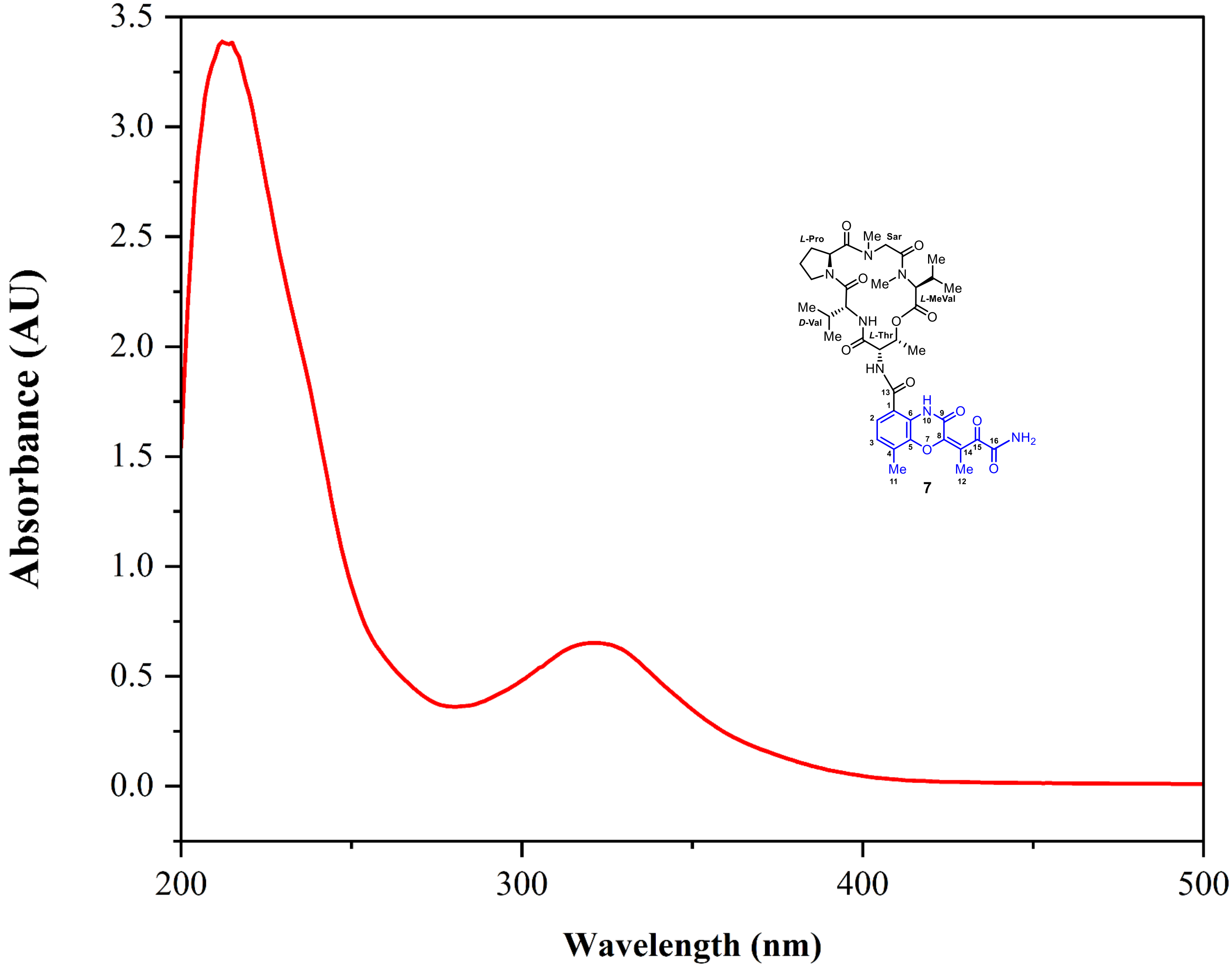

Figure S68. HR-ESIMS for compound **7**

Mass Spectrum SmartFormula Report

Analysis Info

Analysis Name	D:\Data\MS\data\202505\panghe_S-4-1_pos_45_01_19984.d	Acquisition Date	5/29/2025 6:02:01 PM
Method	LC_Direct Infusion_pos_100-1000mz.m	Operator	SCSIO
Sample Name	panghe_S-4-1_pos	Instrument	maXis 255552.00029
Comment			

Acquisition Parameter

Source Type	ESI	Ion Polarity	Positive	Set Nebulizer	0.4 Bar
Focus	Active	Set Capillary	4500 V	Set Dry Heater	180 °C
Scan Begin	100 m/z	Set End Plate Offset	-500 V	Set Dry Gas	4.0 l/min
Scan End	2000 m/z	Set Charging Voltage	0 V	Set Divert Valve	Waste
		Set Corona	0 nA	Set APCI Heater	0 °C

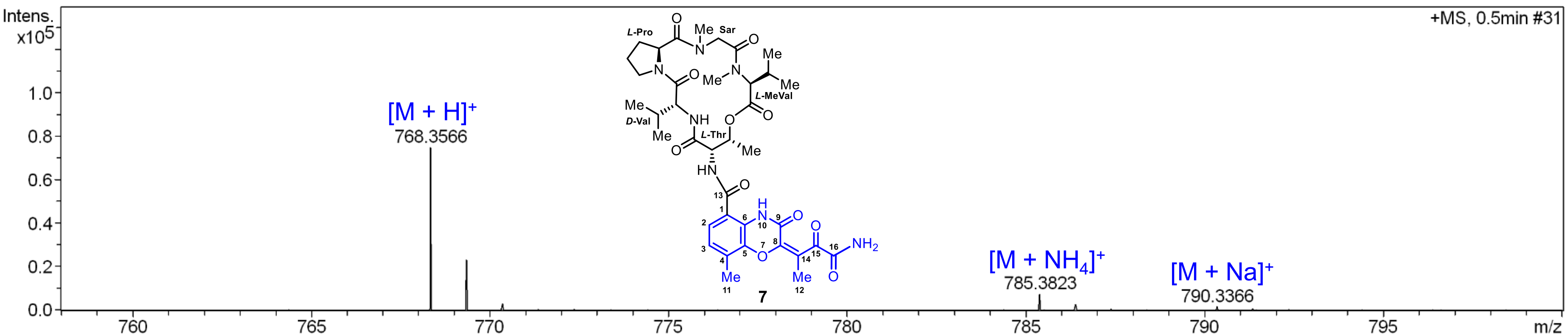

Meas. m/z	#	Ion Formula	Score	m/z	err [ppm]	err [mDa]	mSigma	rdb	e⁻ Conf	N-Rule
768.3566	1	C37H50N7O11	100.00	768.3563	0.5	0.4	65.7	16.5	even	ok
785.3823	1	C37H53N8O11	100.00	785.3828	-0.7	-0.5	33.7	15.5	even	ok
790.3366	1	C37H49N7NaO11	100.00	790.3382	2.1	1.7	52.1	16.5	even	ok

Figure S69. ^{1}H NMR (600 MHz) spectrum of compound **7** in DMSO-d_6

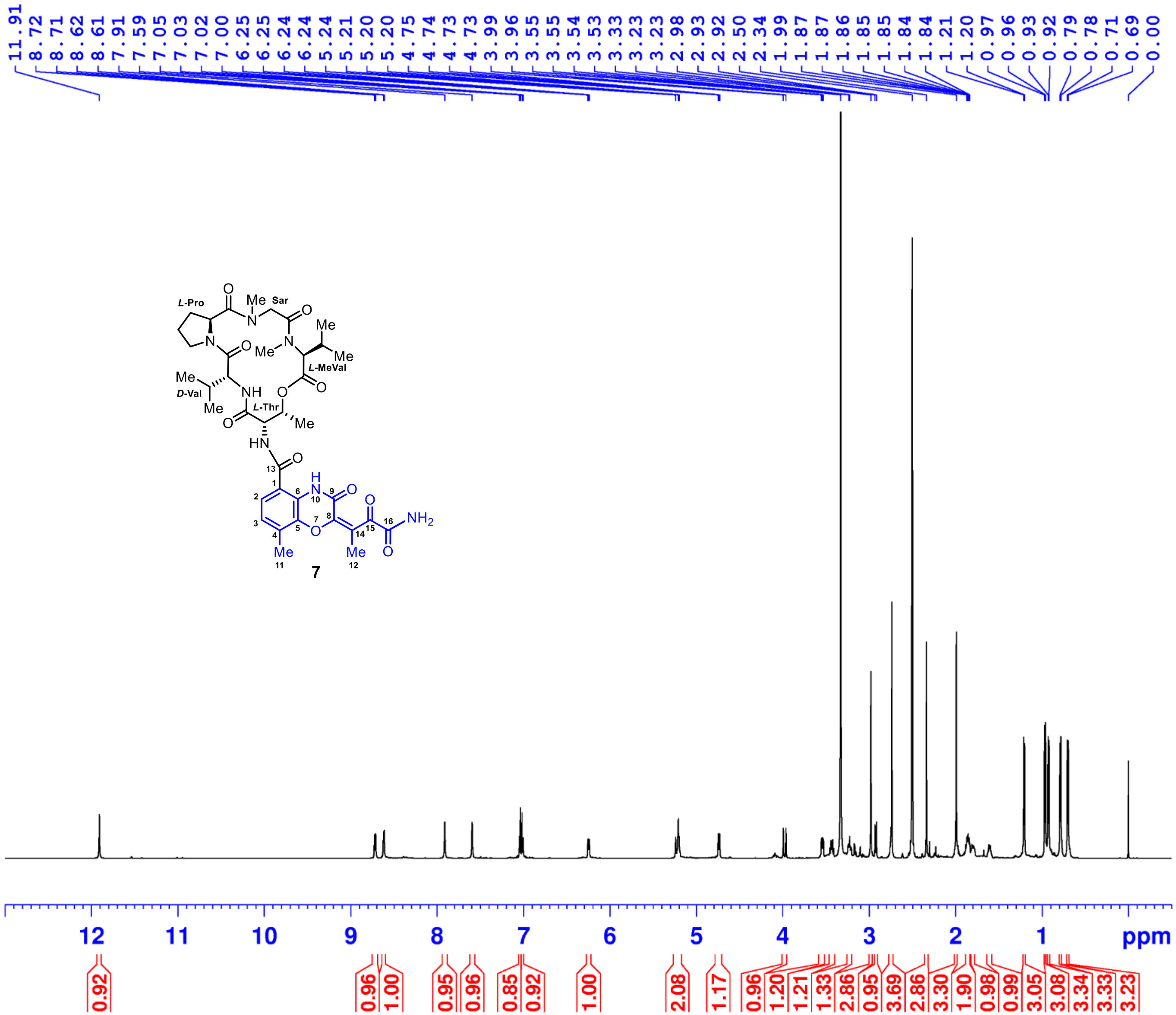

NAME	241129_S41_H
EXPNO	10
PROCNO	1
Date_	20241129
Time	23.40 h
INSTRUM	Avance NEO 600
PROBHD	Z168348_0005 (
PULPROG	zg30
TD	65536
SOLVENT	DMSO
NS	16
DS	2
SWH	11904.762 Hz
FIDRES	0.363304 Hz
AQ	2.7525620 sec
RG	22.6
DW	42.000 usec
DE	14.39 usec
TE	298.2 K
D1	1.00000000 sec
TD0	1
SFO1	600.1937062 MHz
NUC1	1H
P0	2.67 usec
P1	8.00 usec
SI	65536
SF	600.1900038 MHz
WDW	EM
SSB	0
LB	0.30 Hz
GB	0
PC	1.00

Figure S69-continue

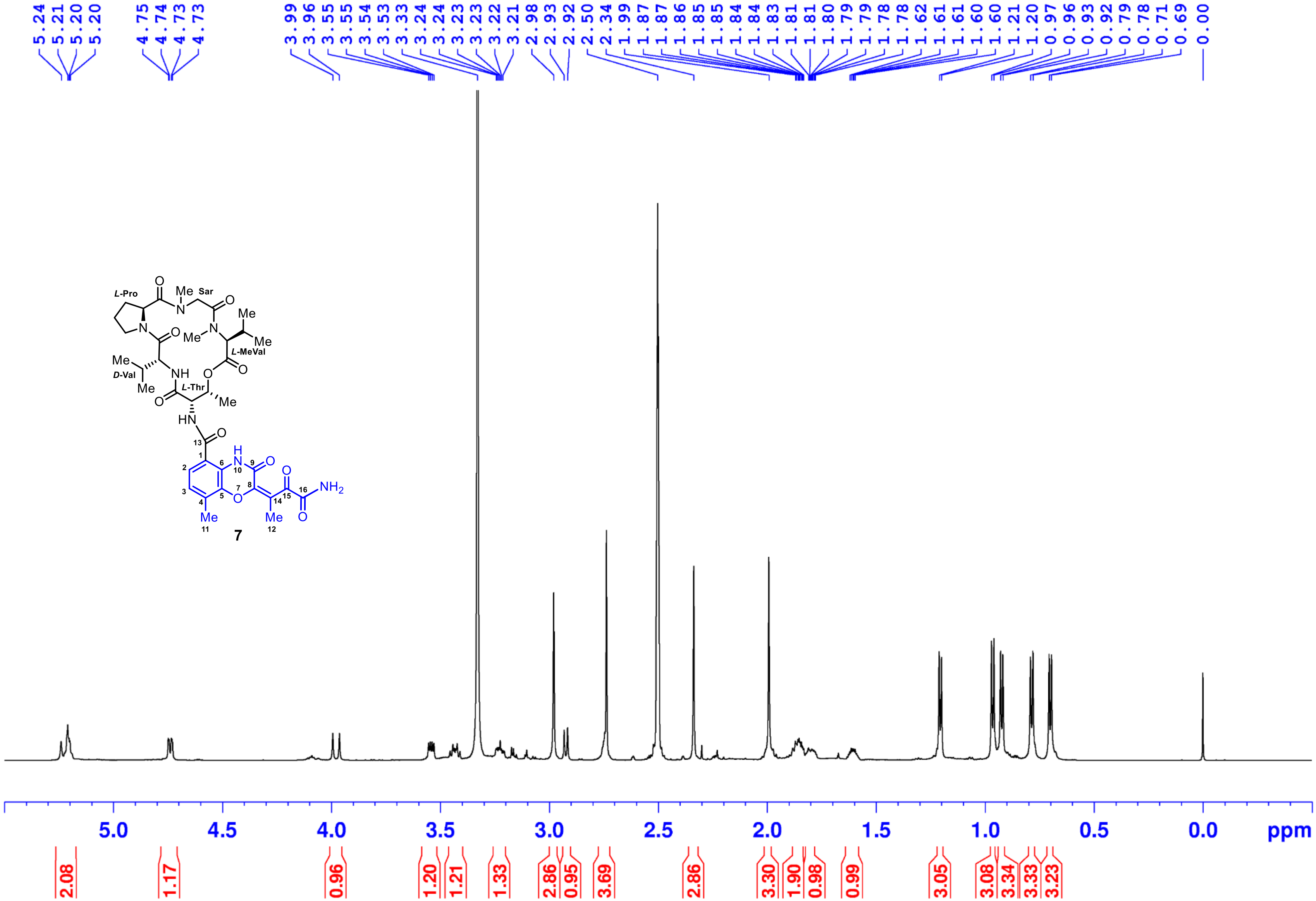

Figure S69-continue

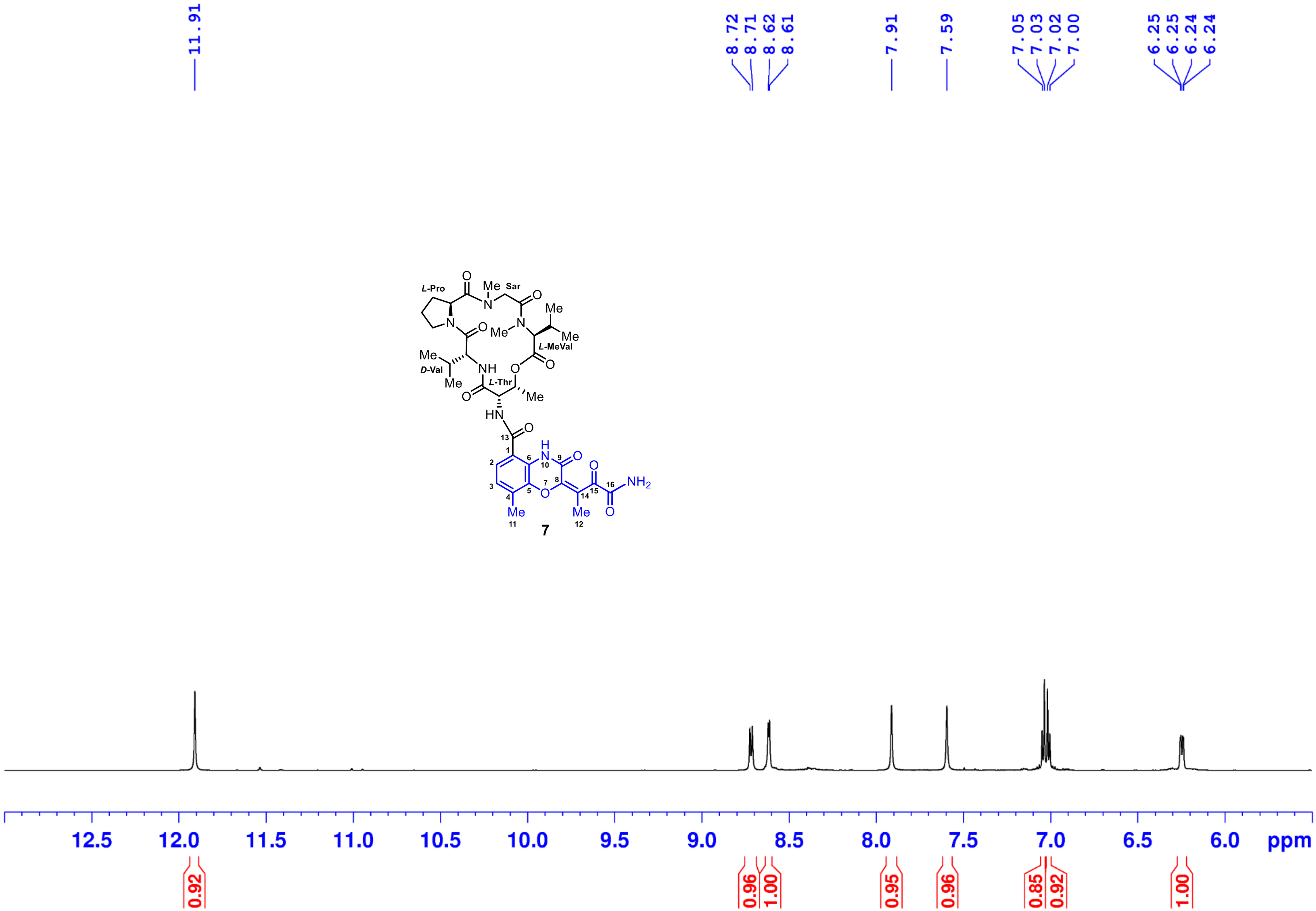

Figure S70. ^{13}C NMR (150 MHz) spectrum of compound **7** in DMSO-d_6

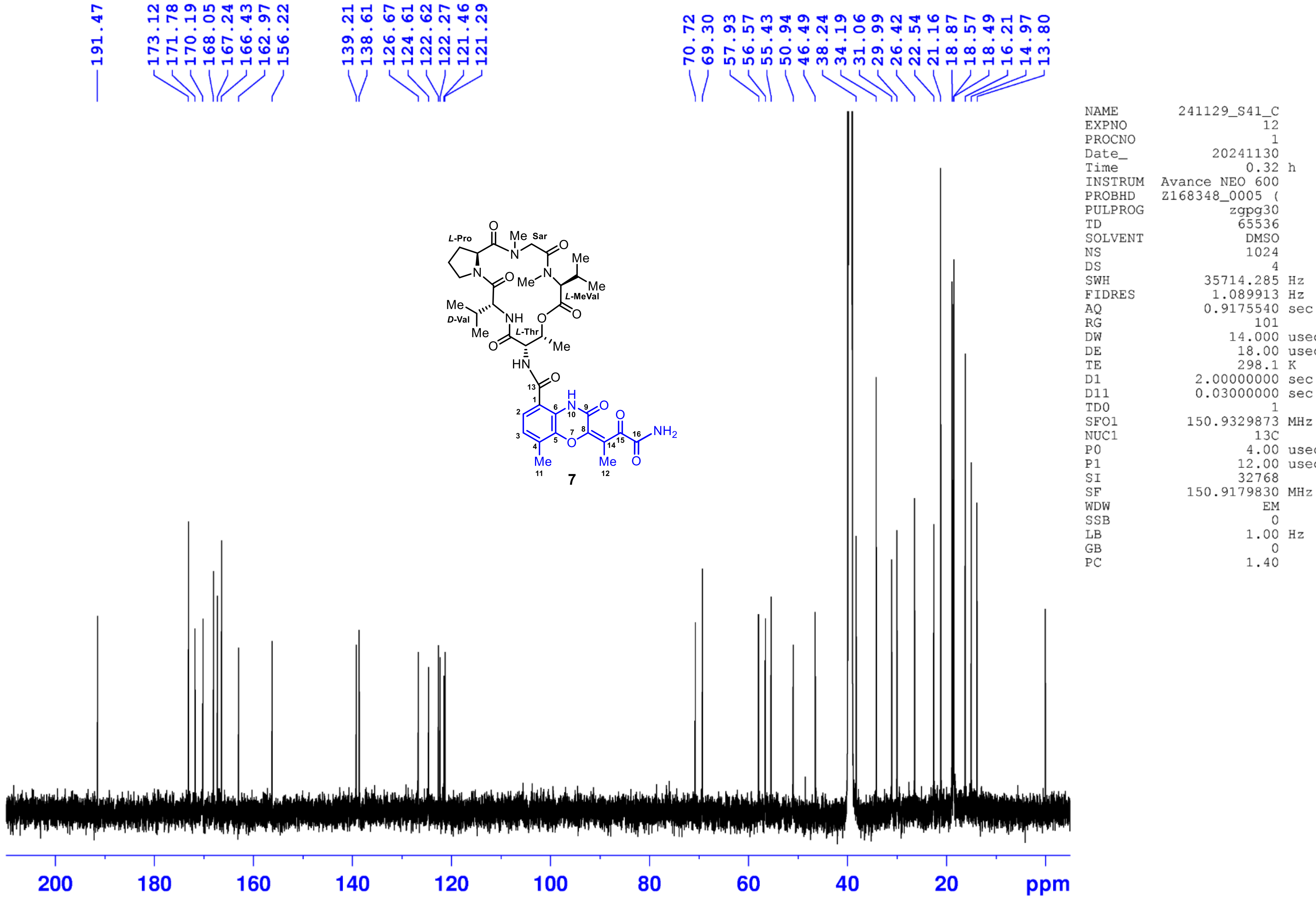

Figure S70-continue

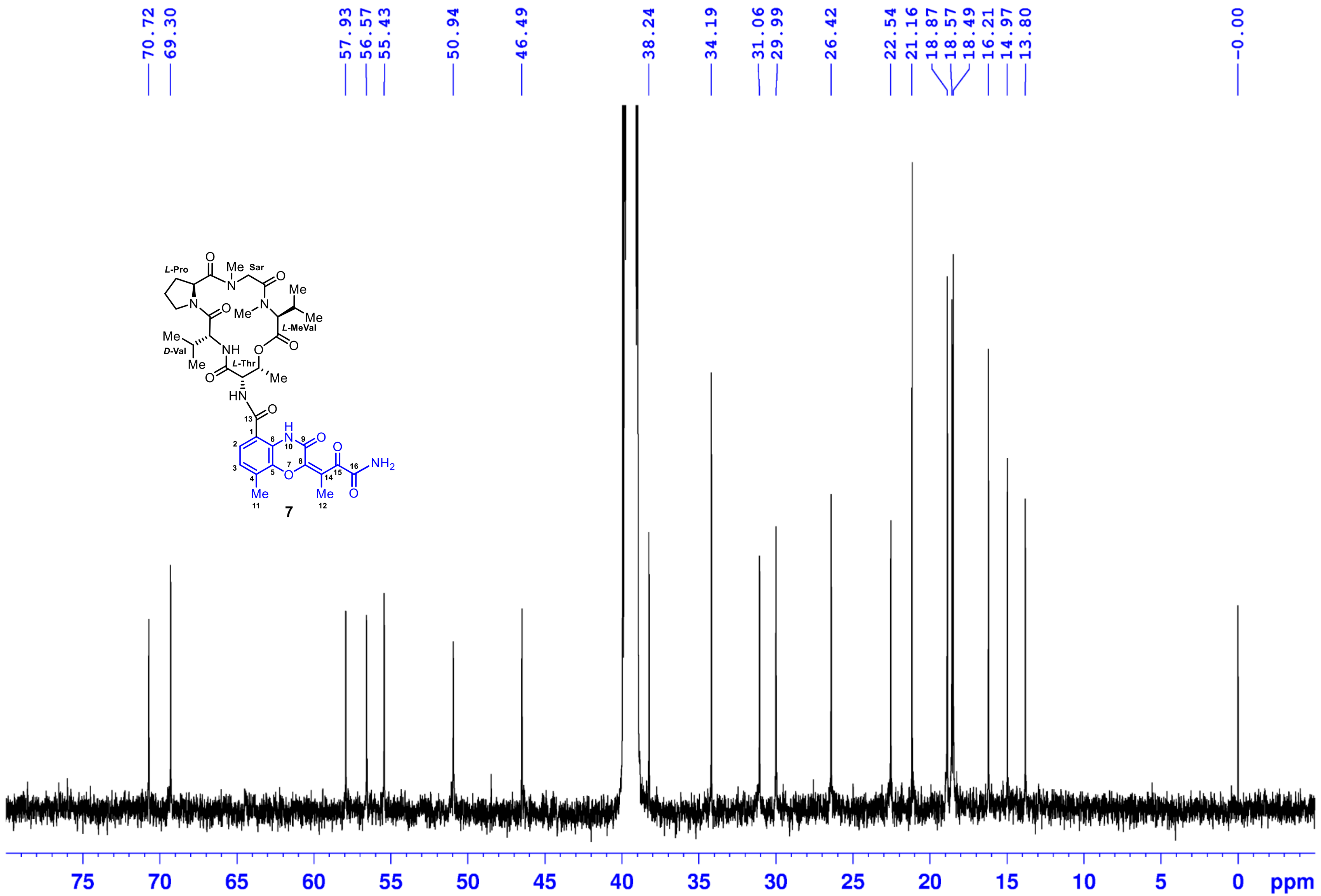

Figure S70-continue

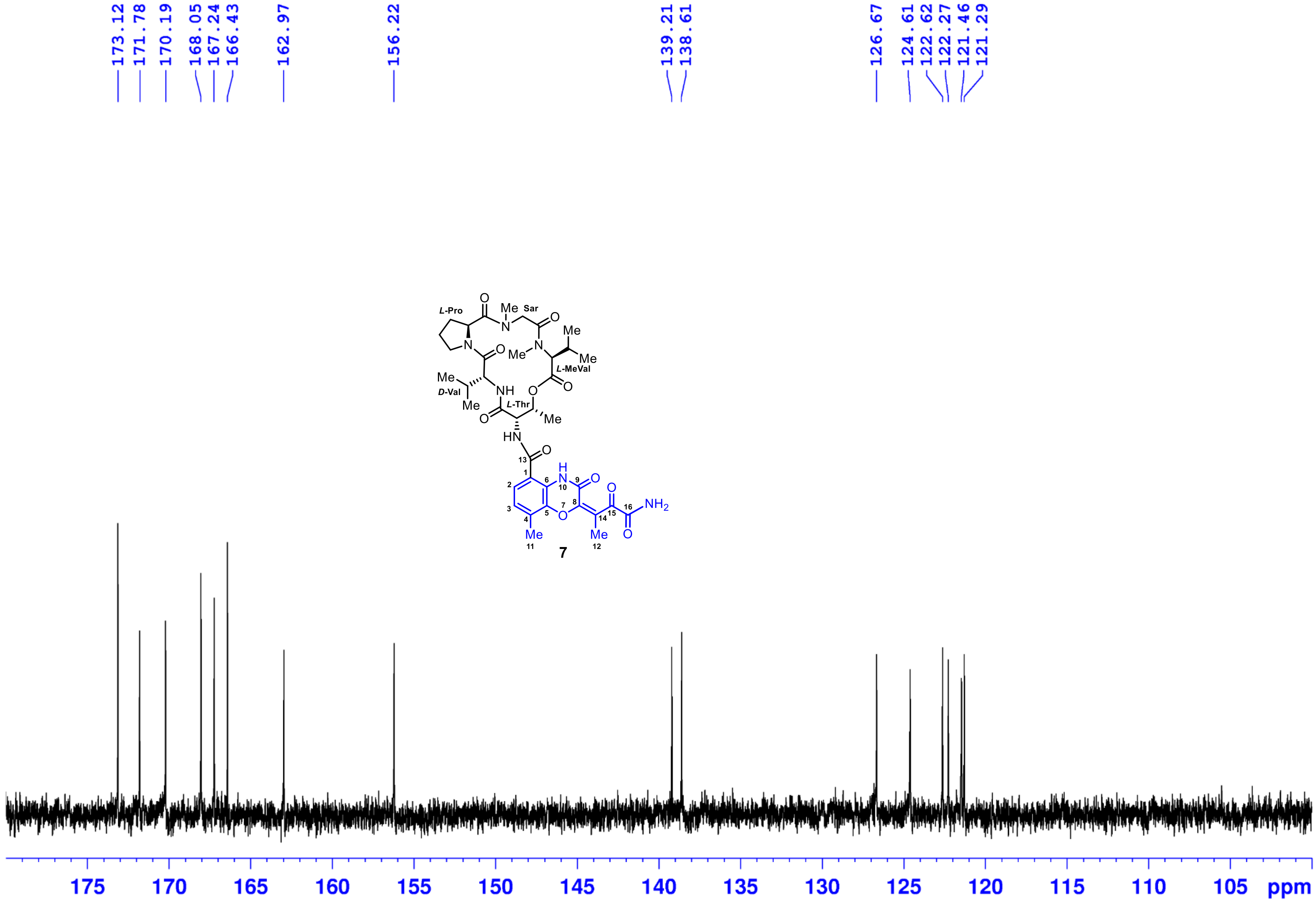

Figure S71. DEPT135 (150 MHz) spectrum of compound **7** in DMSO-d_6

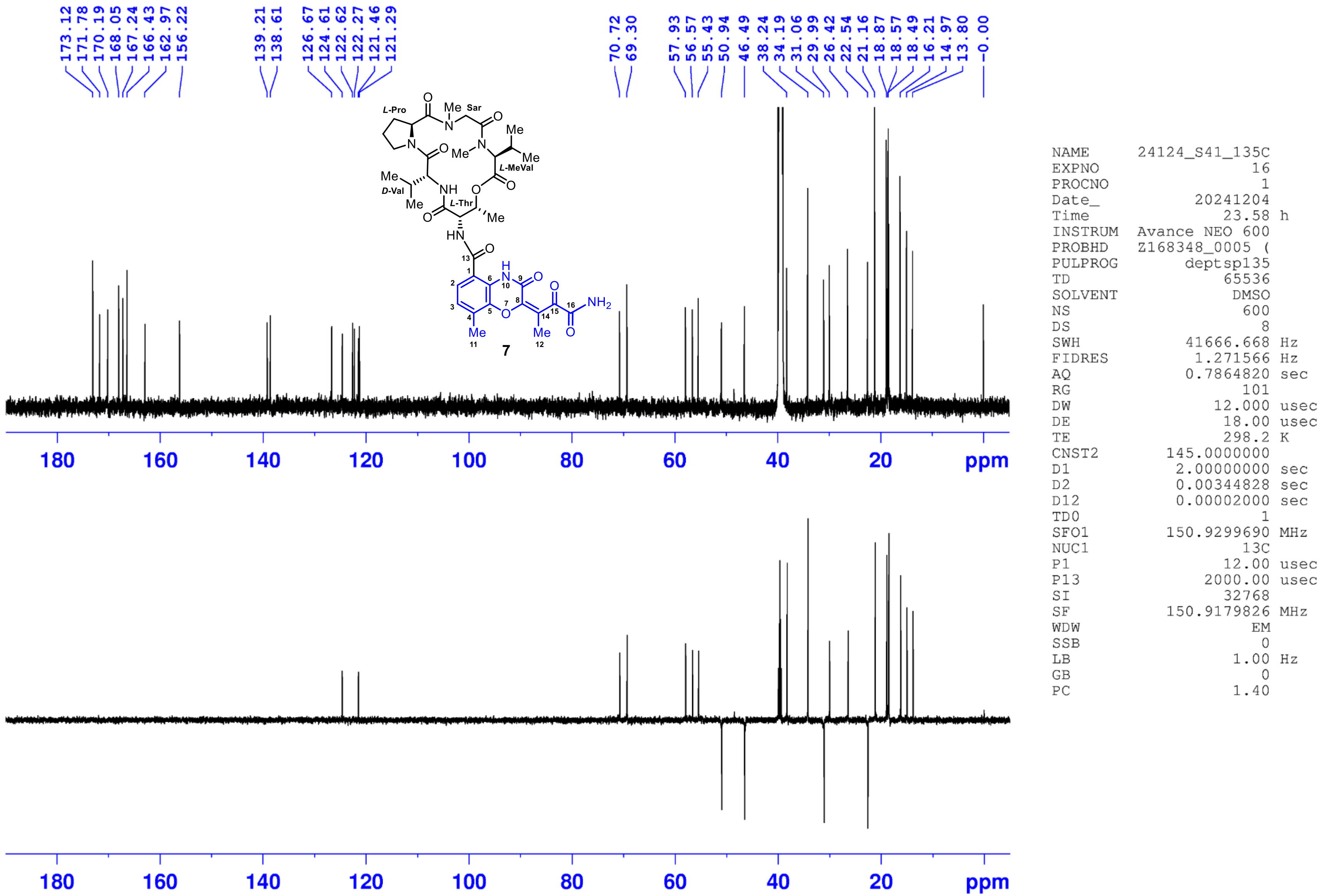

Figure S71-continue

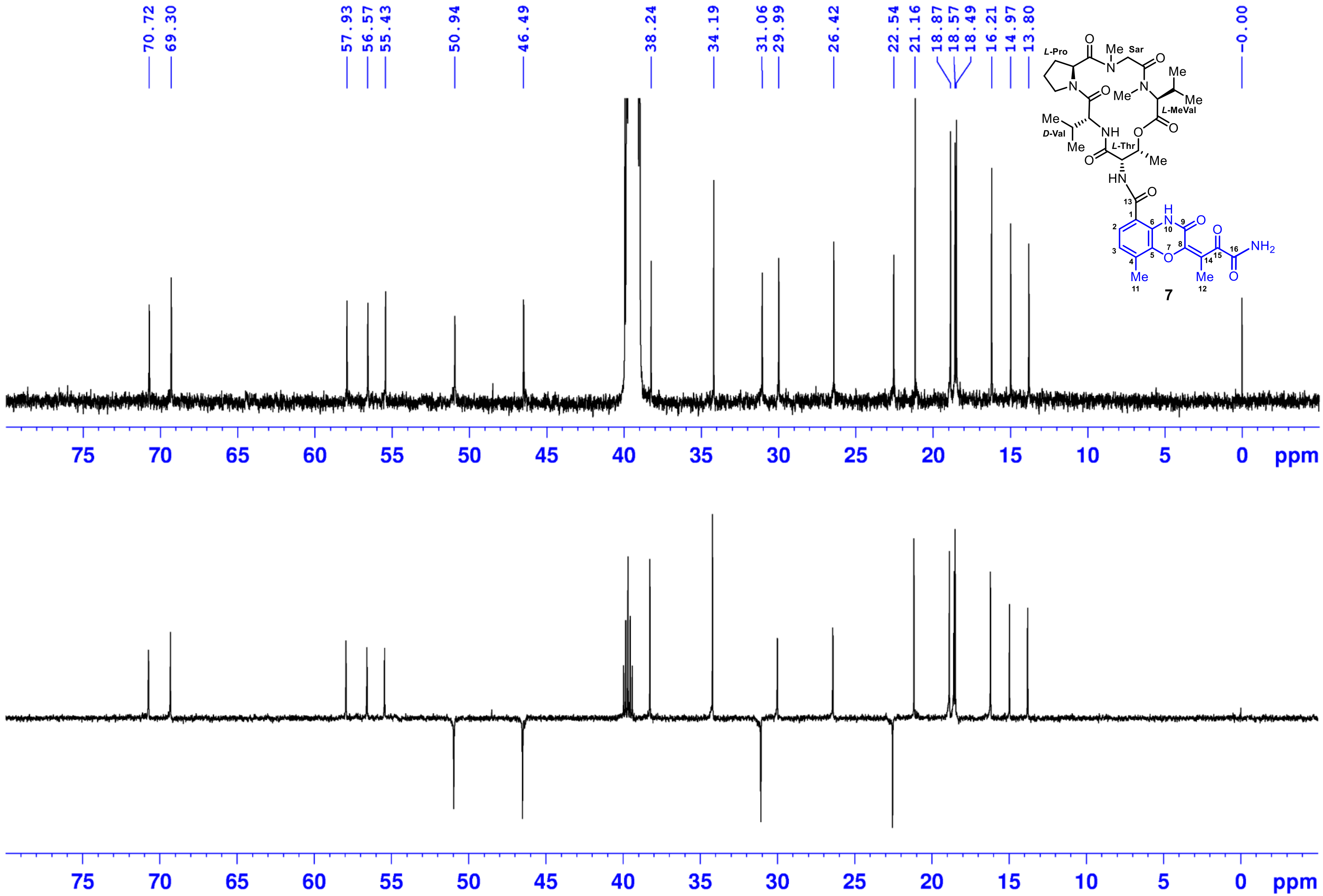

Figure S71-continue

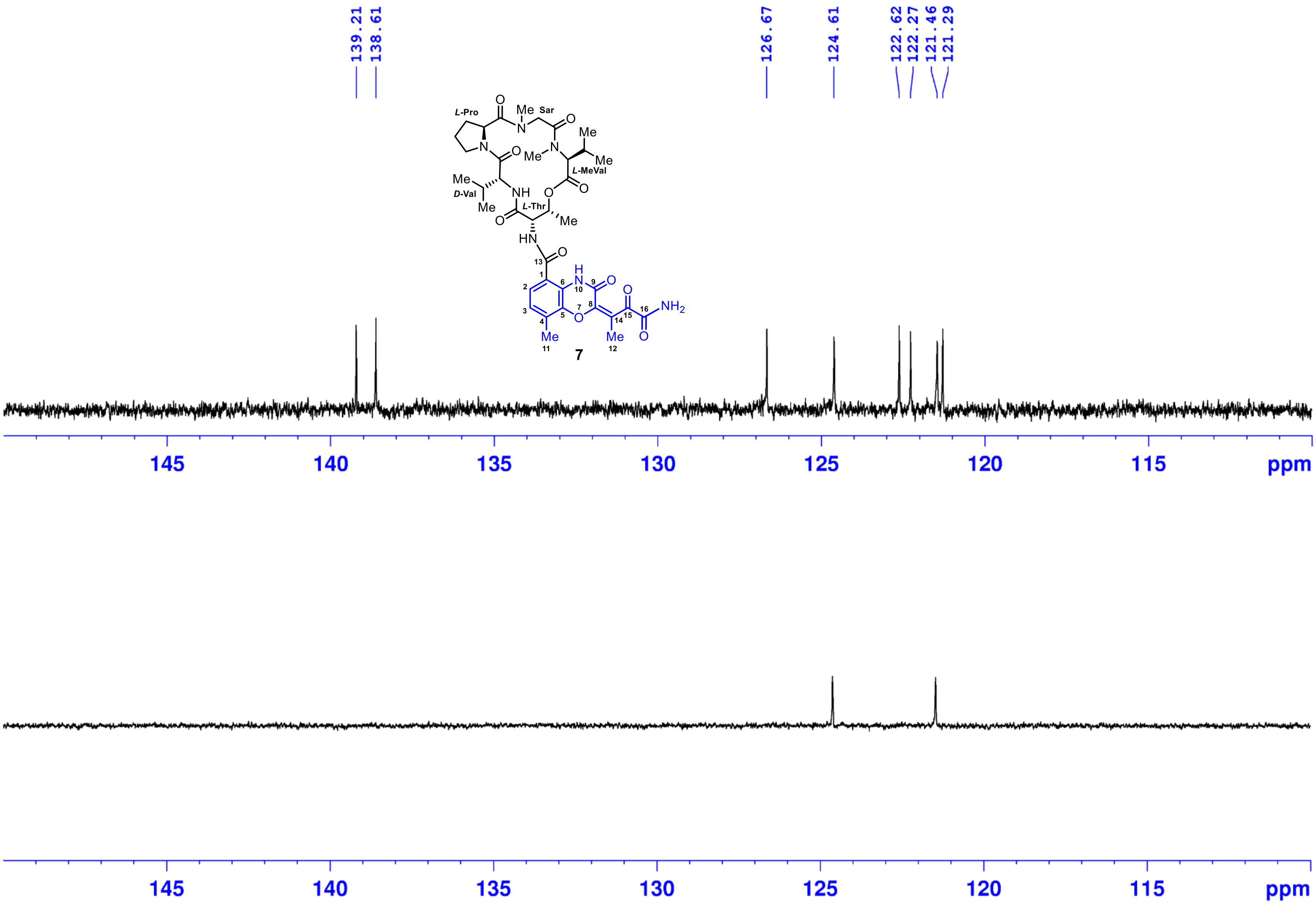

Figure S72. ^1H–^1H COSY (600 MHz) spectrum of compound **7** in DMSO-d_6

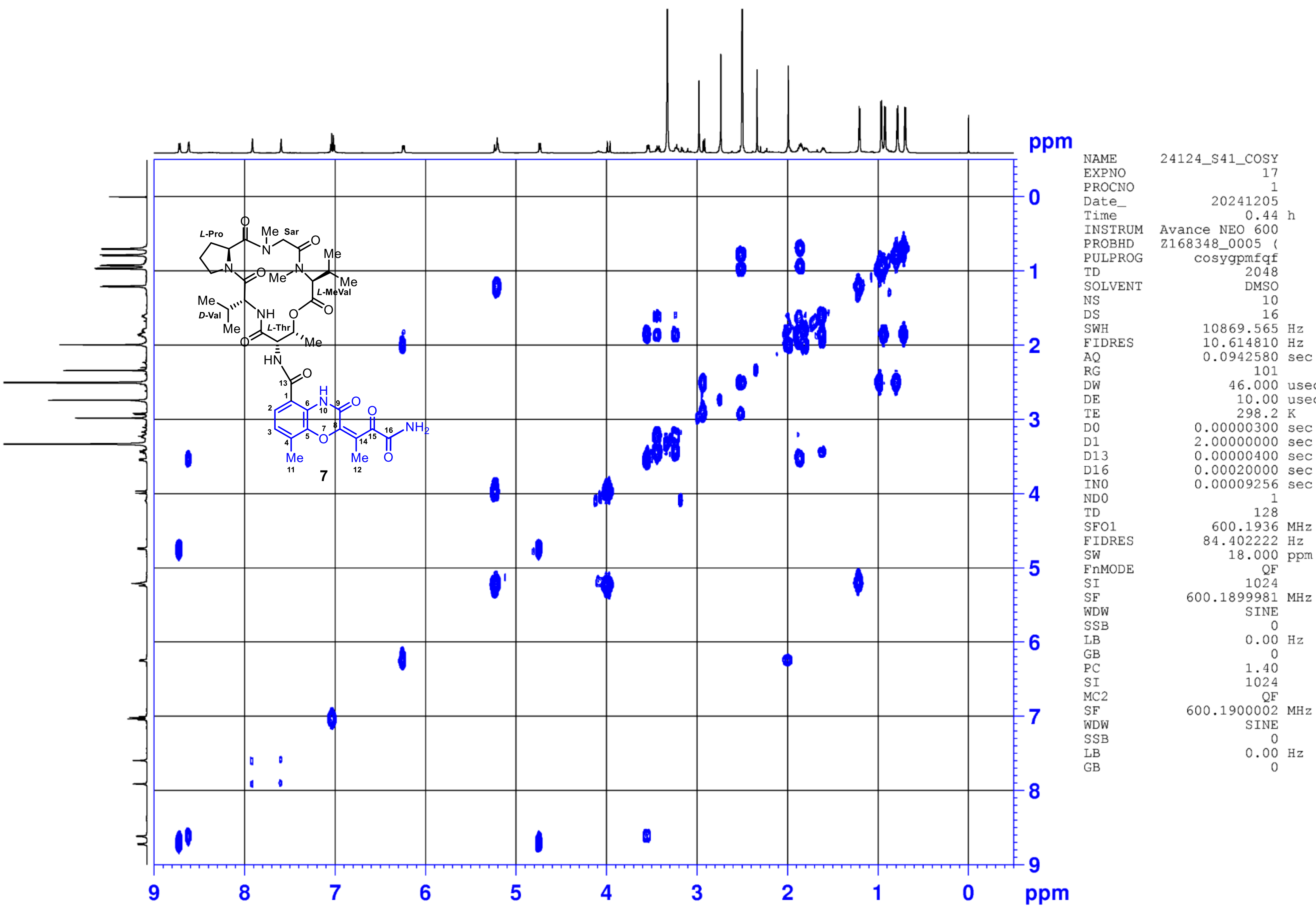

Figure S72-continue

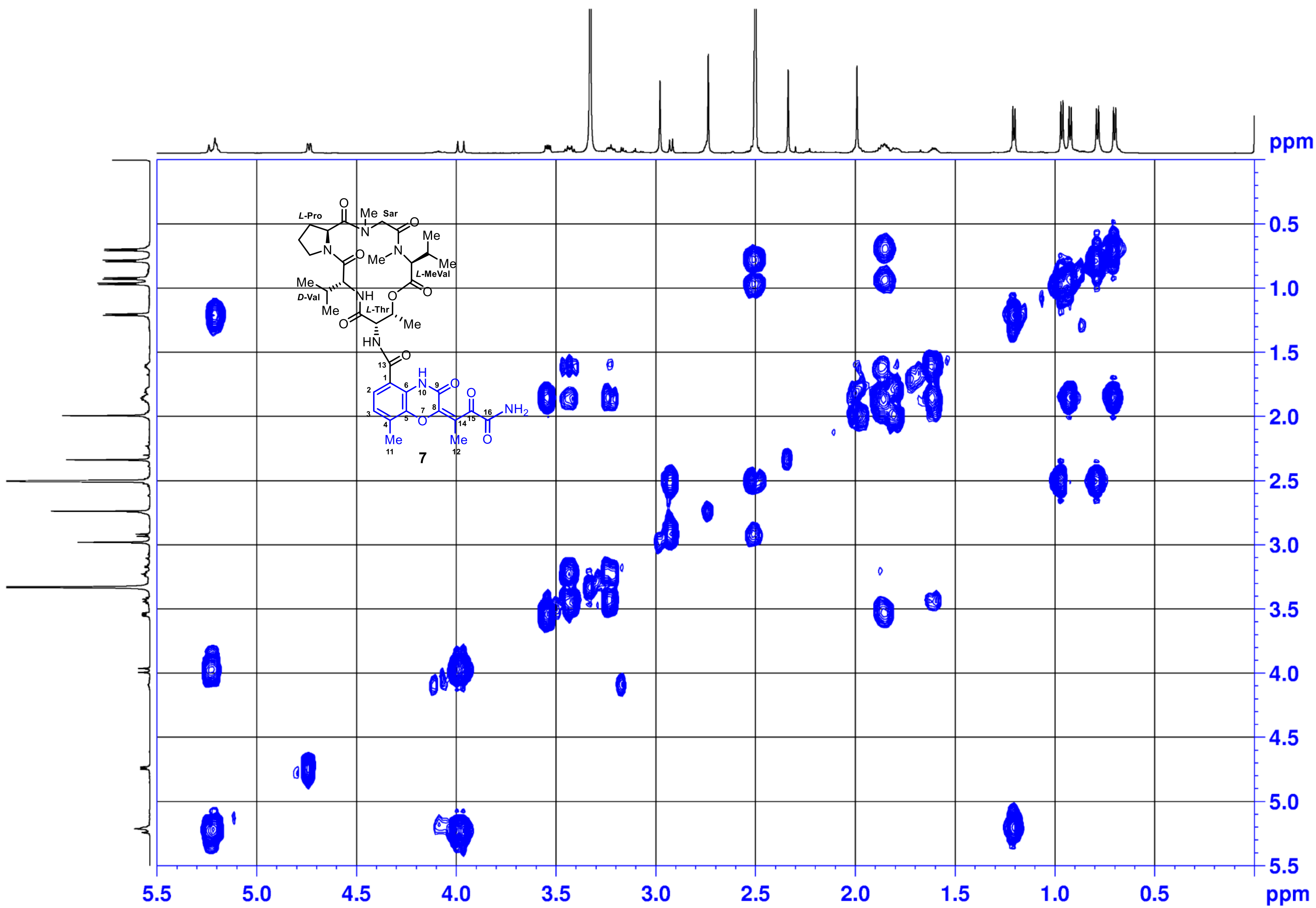

Figure S72-continue

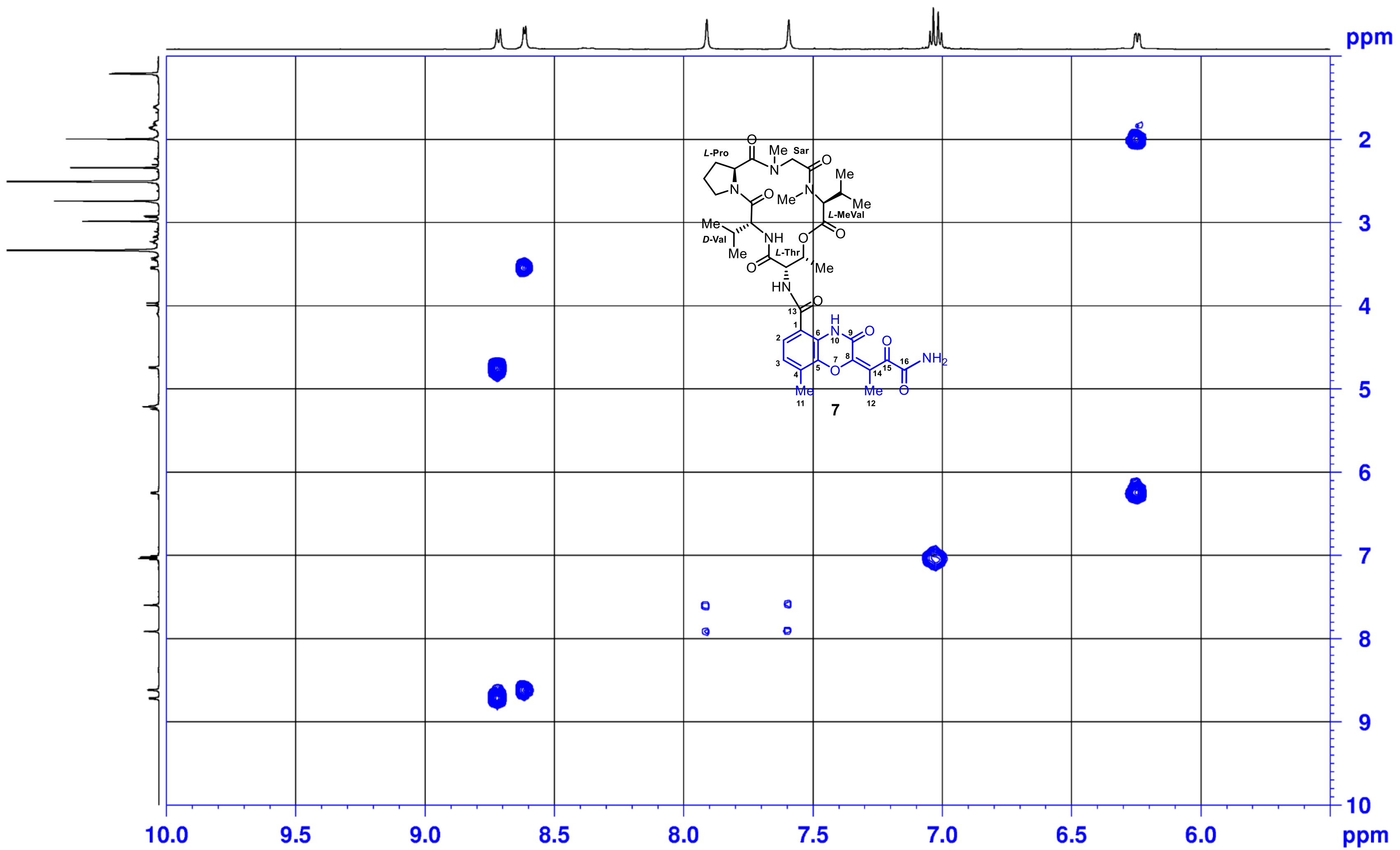

Figure S73. HSQC (600 MHz) spectrum of compound **7** in DMSO-d_6

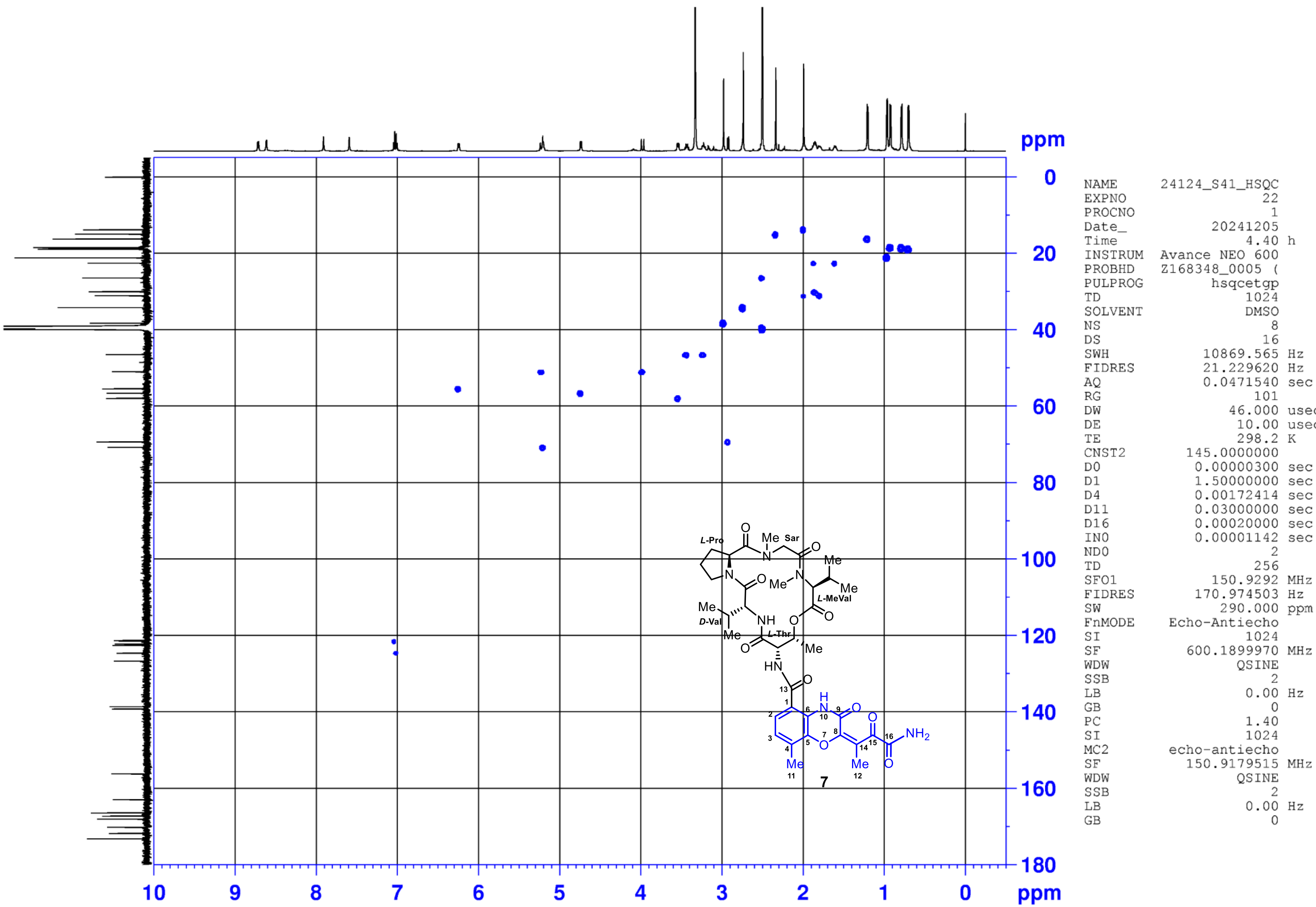

Figure S73-continue

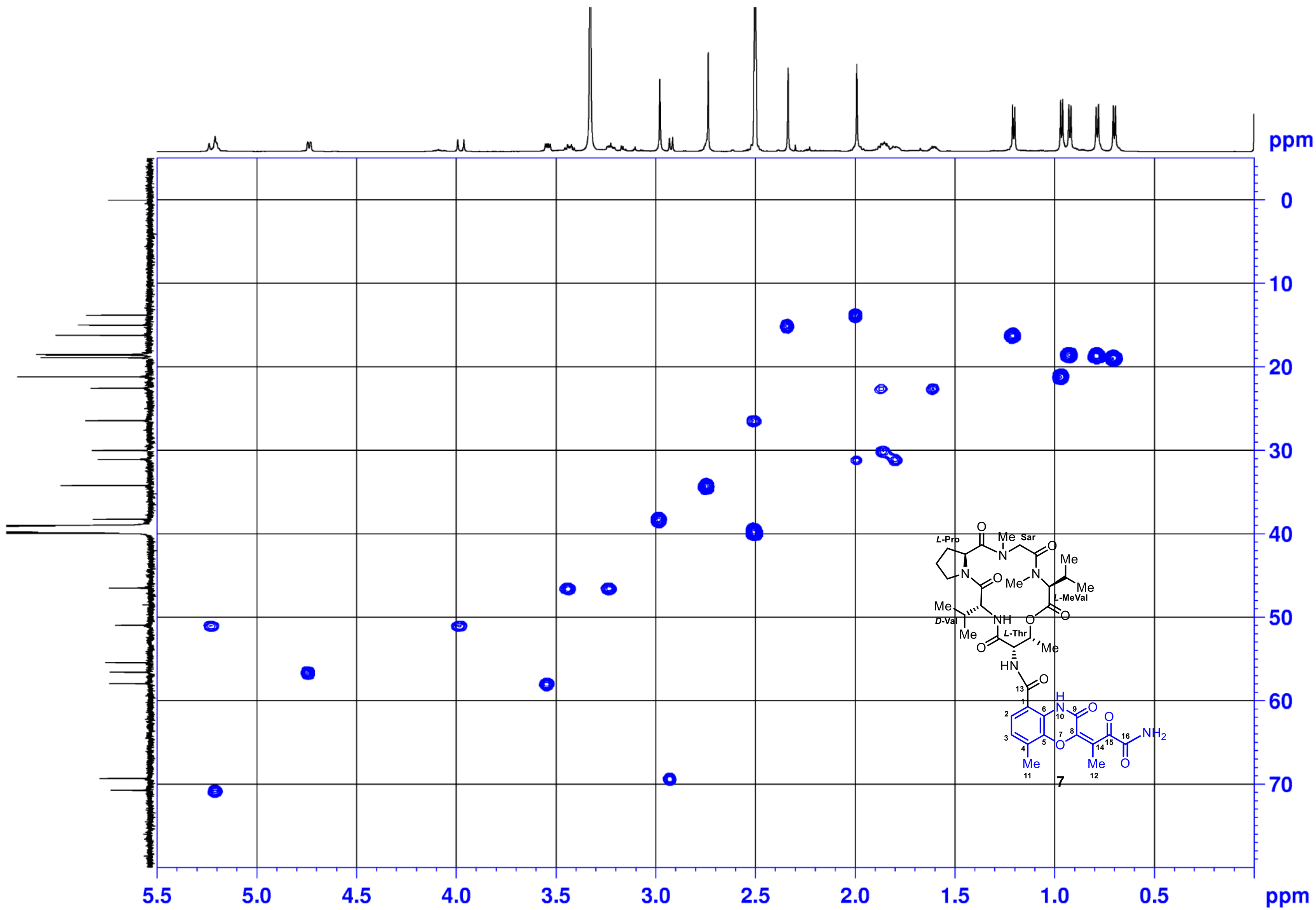

Figure S73-continue

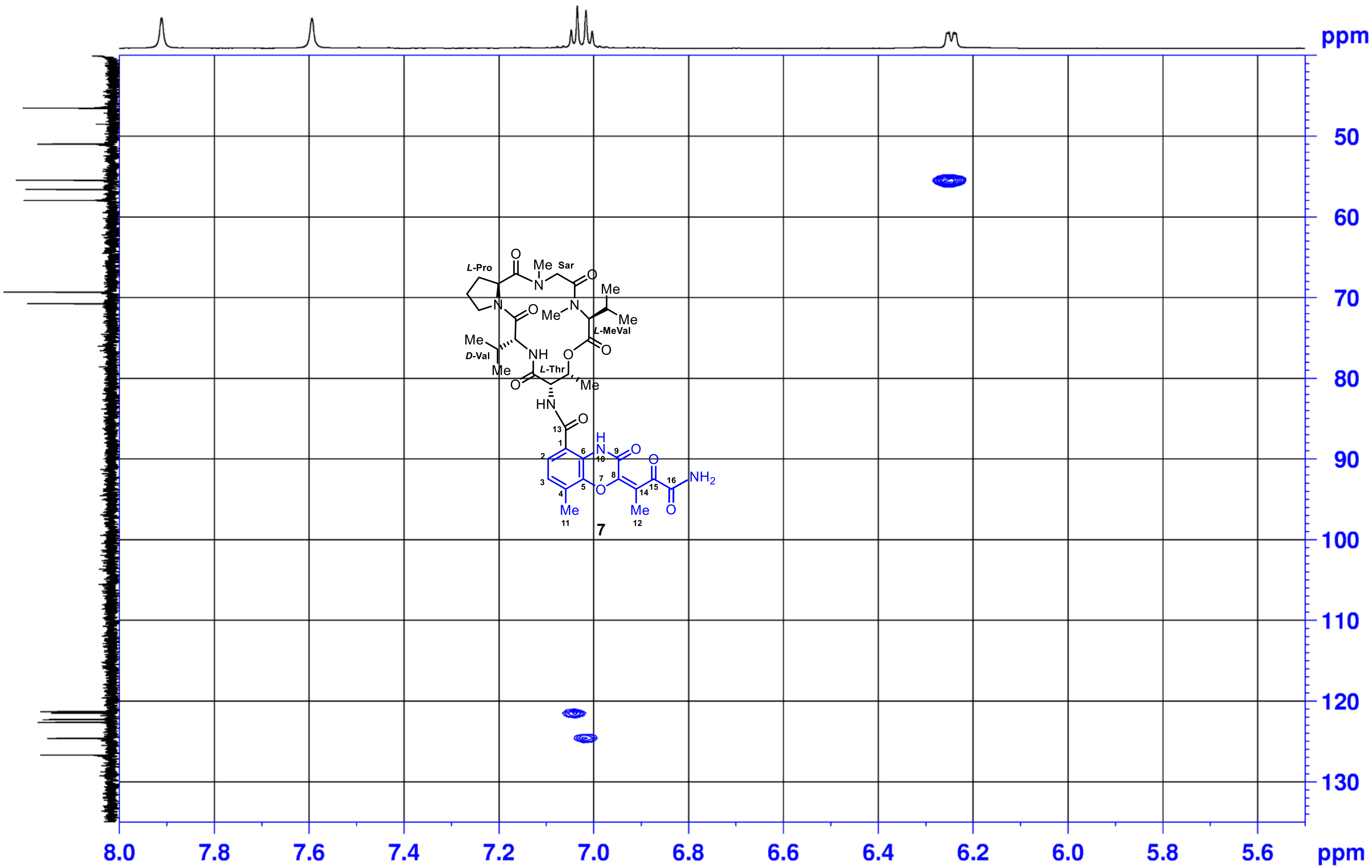

Figure S74. HMBC (600 MHz) spectrum of compound **7** in DMSO-d_6

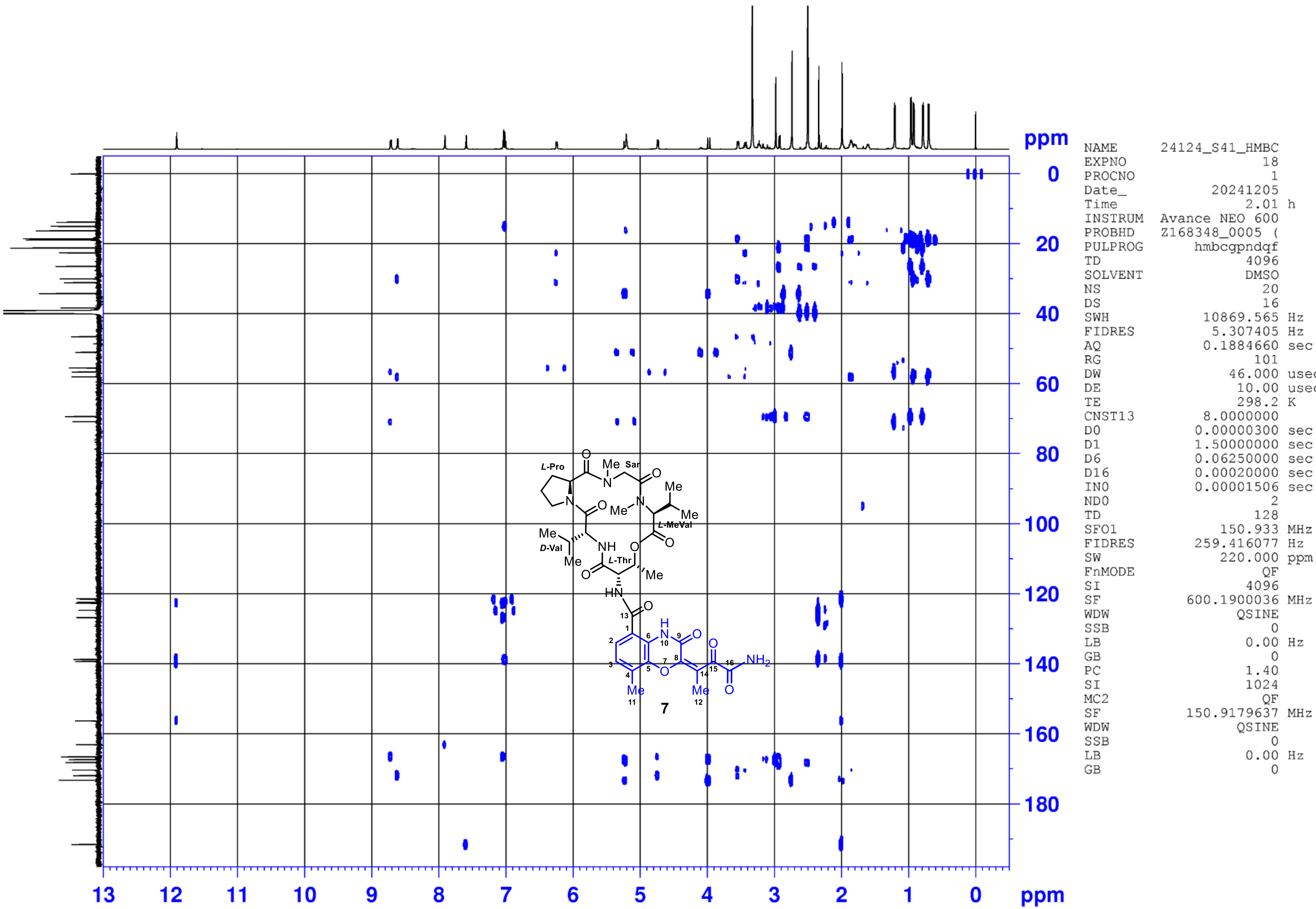

Figure S74-continue

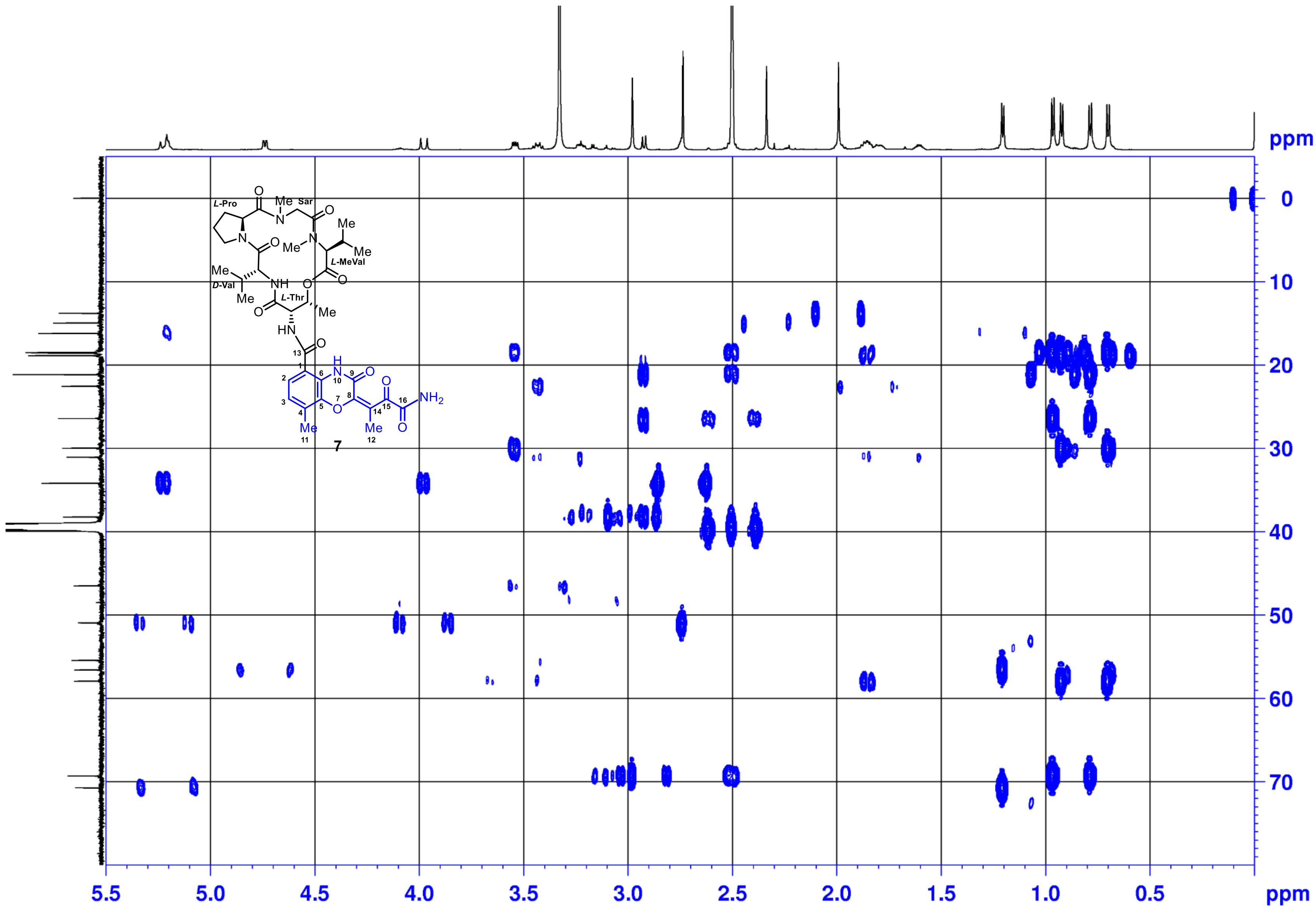

Figure S74-continue

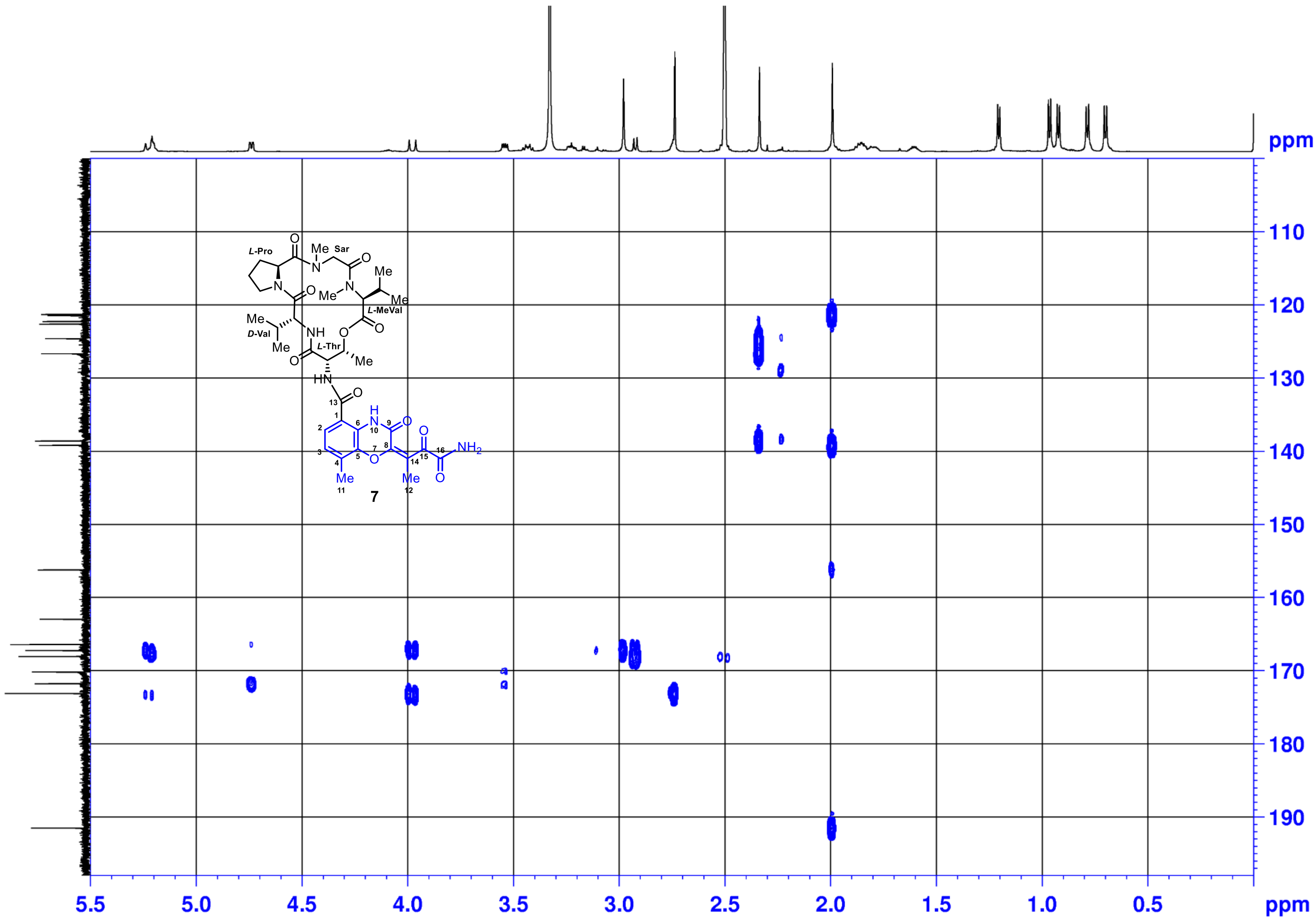

Figure S74-continue

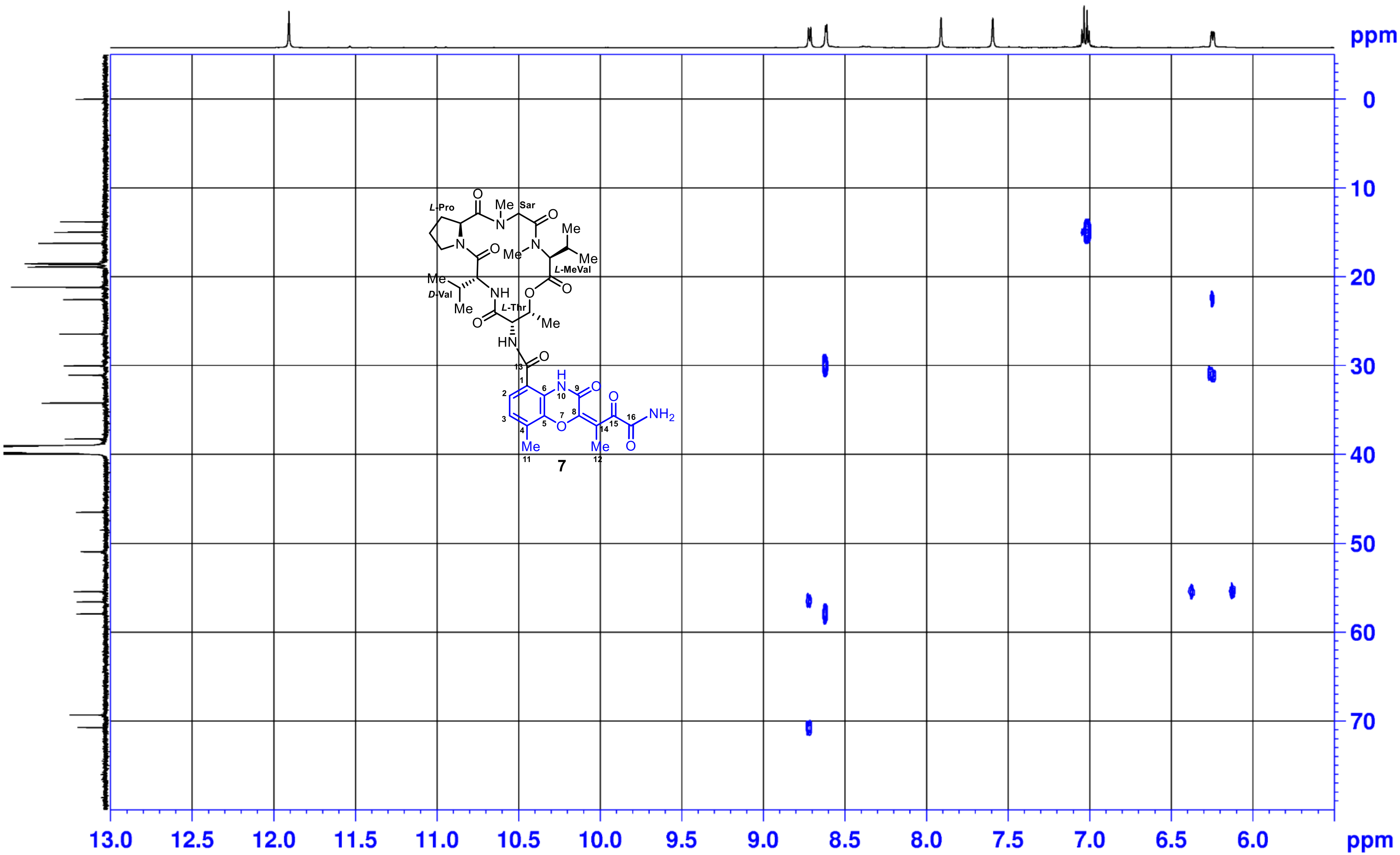

Figure S74-continue

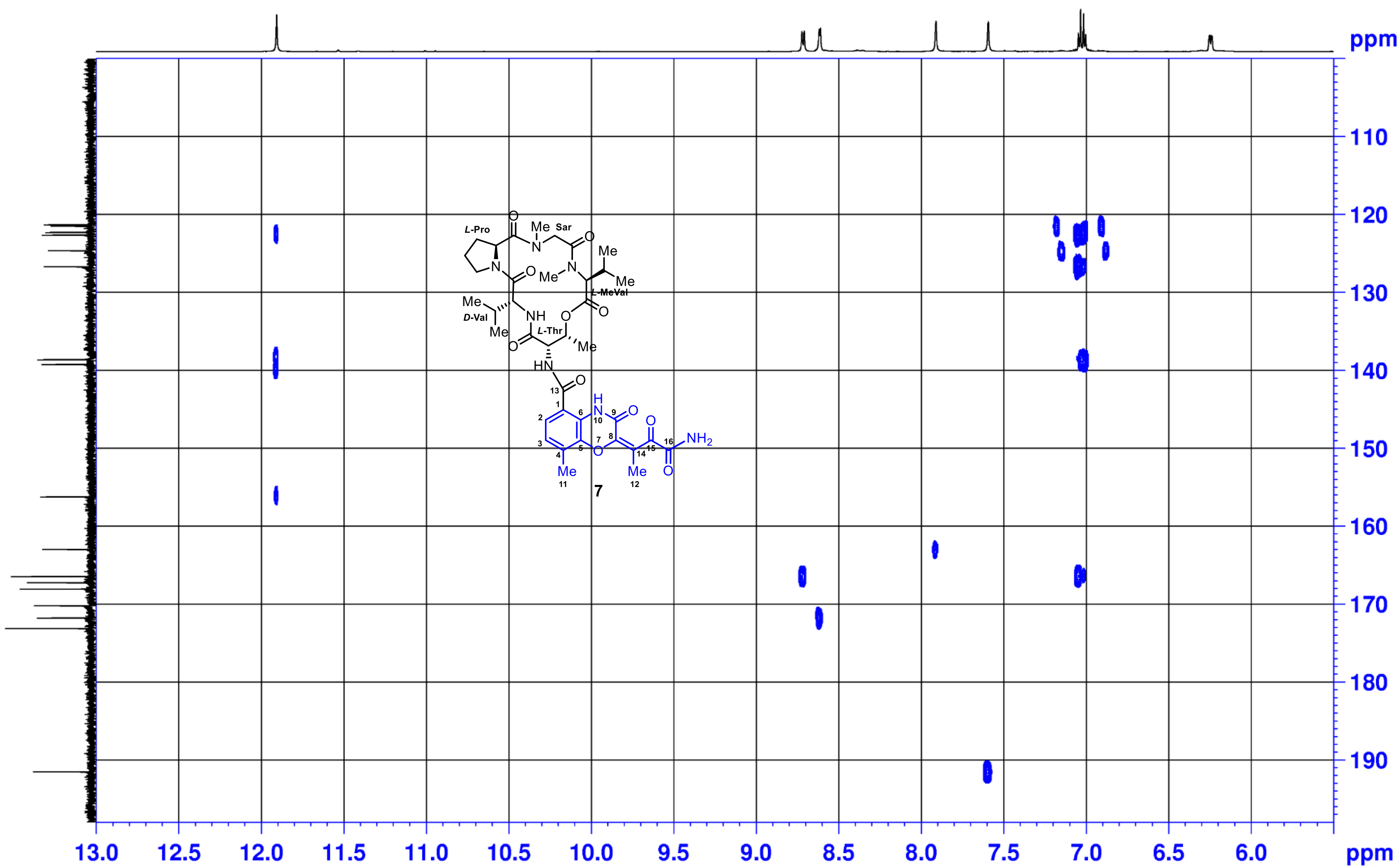

Figure S75. ROESY (600 MHz) spectrum of compound **7** in DMSO-d_6

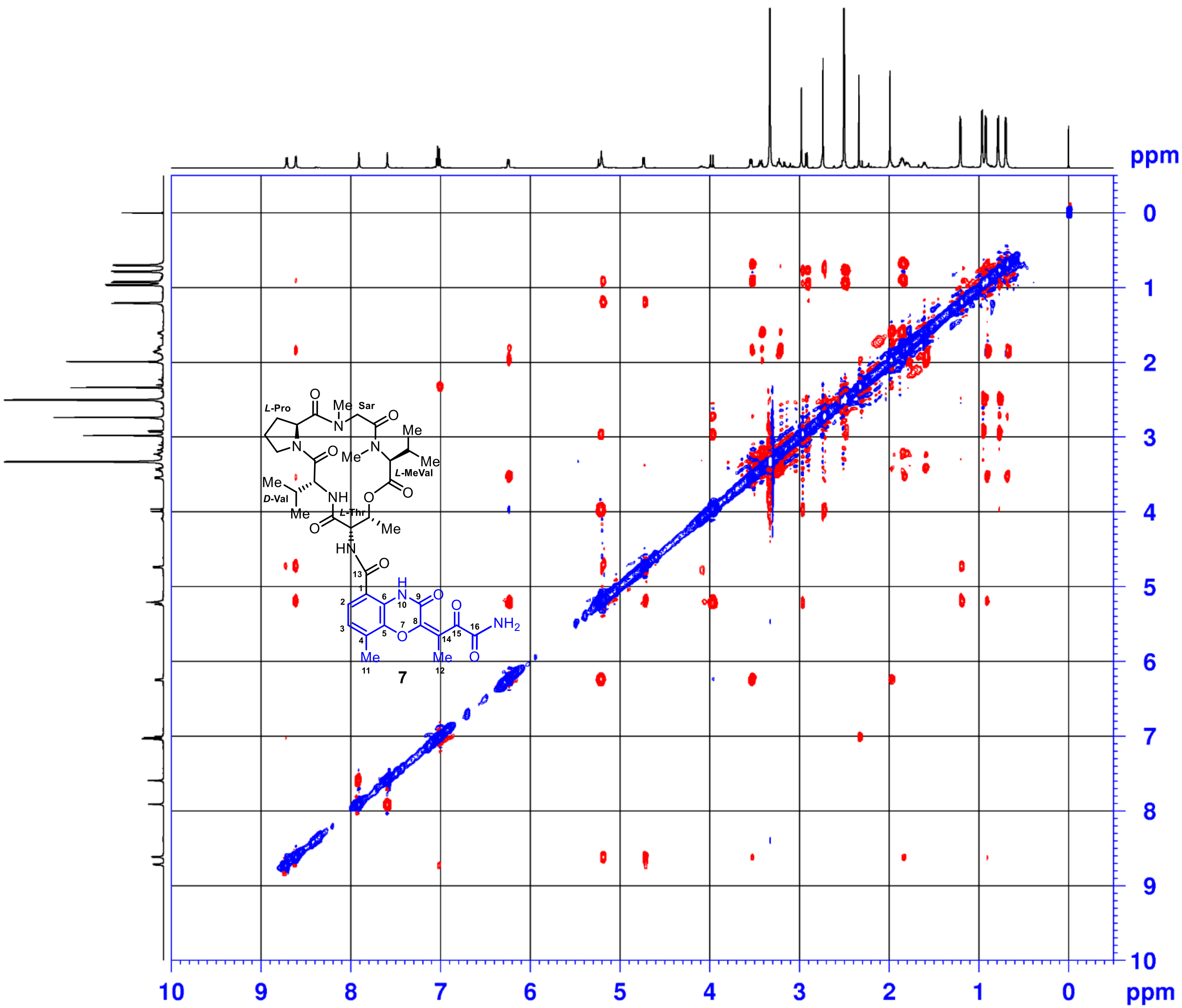

NAME	0526_S_4_1ROE
EXPNO	12
PROCNO	1
Date_	20250527
Time	7.30 h
INSTRUM	Avance NEO 600
PROBHD	Z154705_0139 (
PULPROG	roesyphpp.2
TD	2048
SOLVENT	DMSO
NS	12
DS	32
SWH	13888.889 Hz
FIDRES	13.563368 Hz
AQ	0.0737780 sec
RG	101
DW	36.000 usec
DE	6.50 usec
TE	293.9 K
D0	0.00002650 sec
D1	2.00000000 sec
D11	0.03000000 sec
D12	0.00002000 sec
IN0	0.00007373 sec
L4	556
P15	200000.00 usec
ND0	1
TD	256
SFO1	600.1928 MHz
FIDRES	52.985771 Hz
SW	22.600 ppm
FnMODE	States-TPPI
SI	1024
SF	600.1900077 MHz
WDW	QSINE
SSB	2
LB	0.00 Hz
GB	0
PC	1.00
SI	1024
MC2	States-TPPI
SF	600.1900068 MHz
WDW	QSINE
SSB	2
LB	0.00 Hz
GB	0

Figure S75-continue

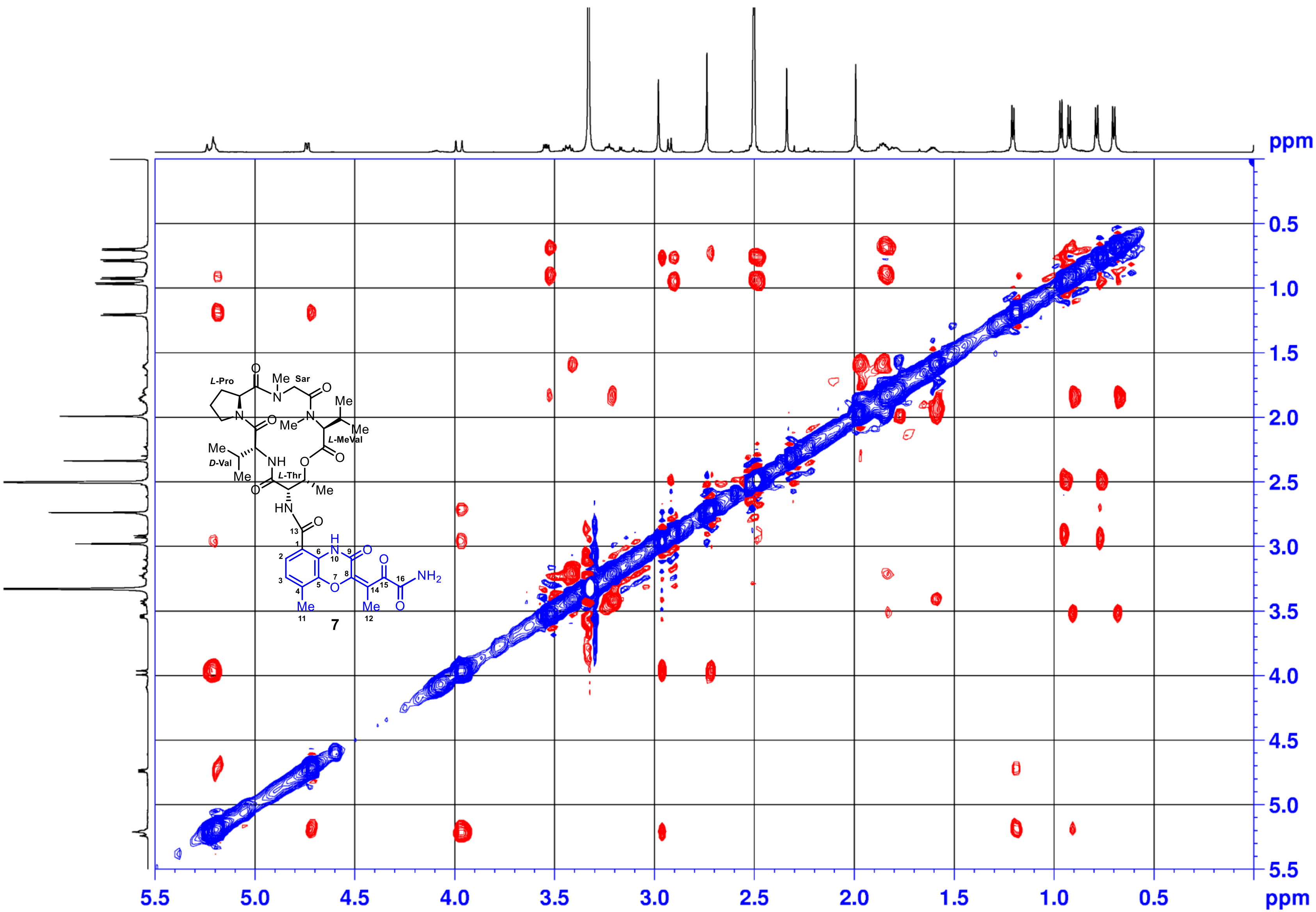

Figure S75-continue

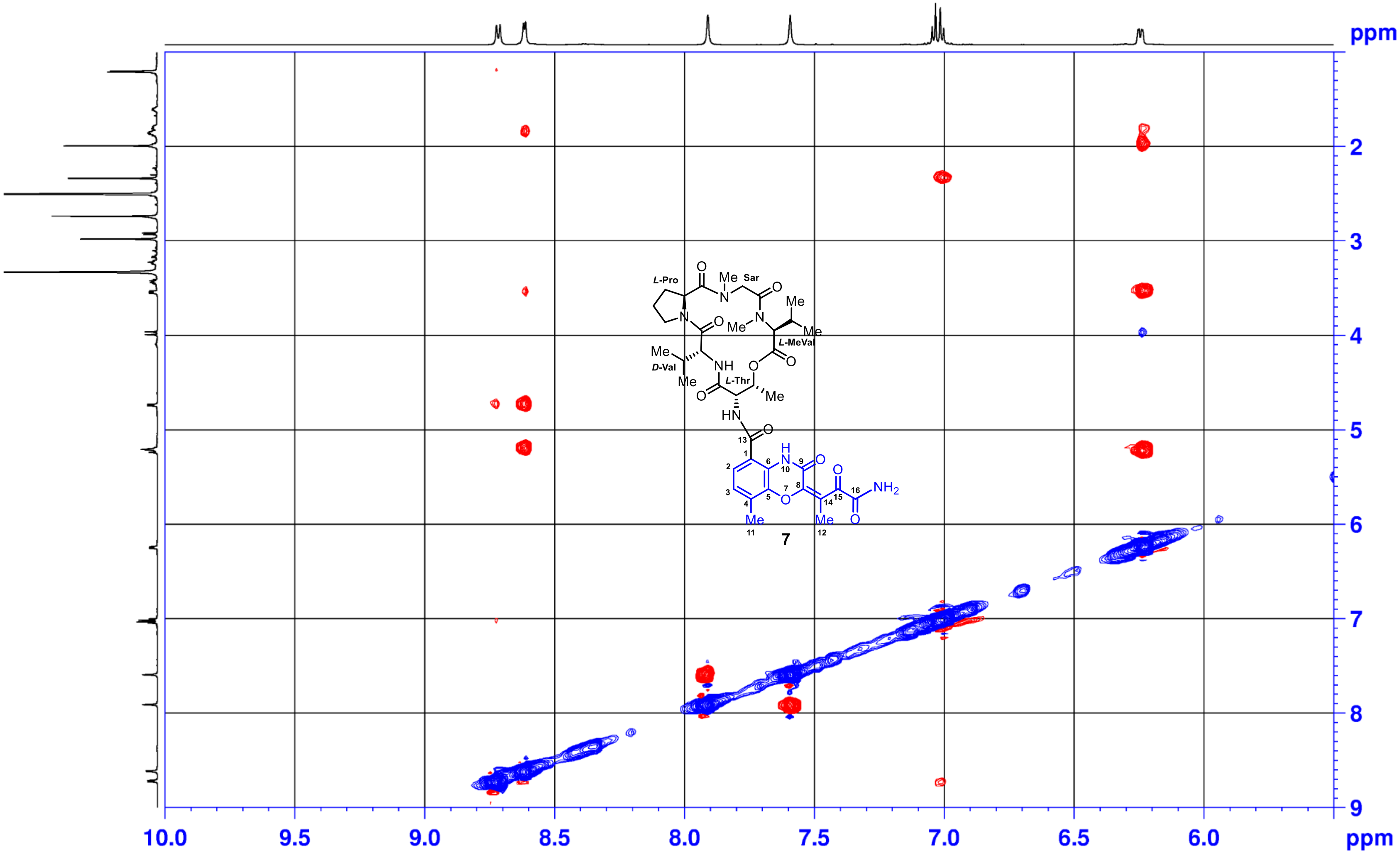

Figure S76. Marfey's analysis of compound **7** and standard amino acids

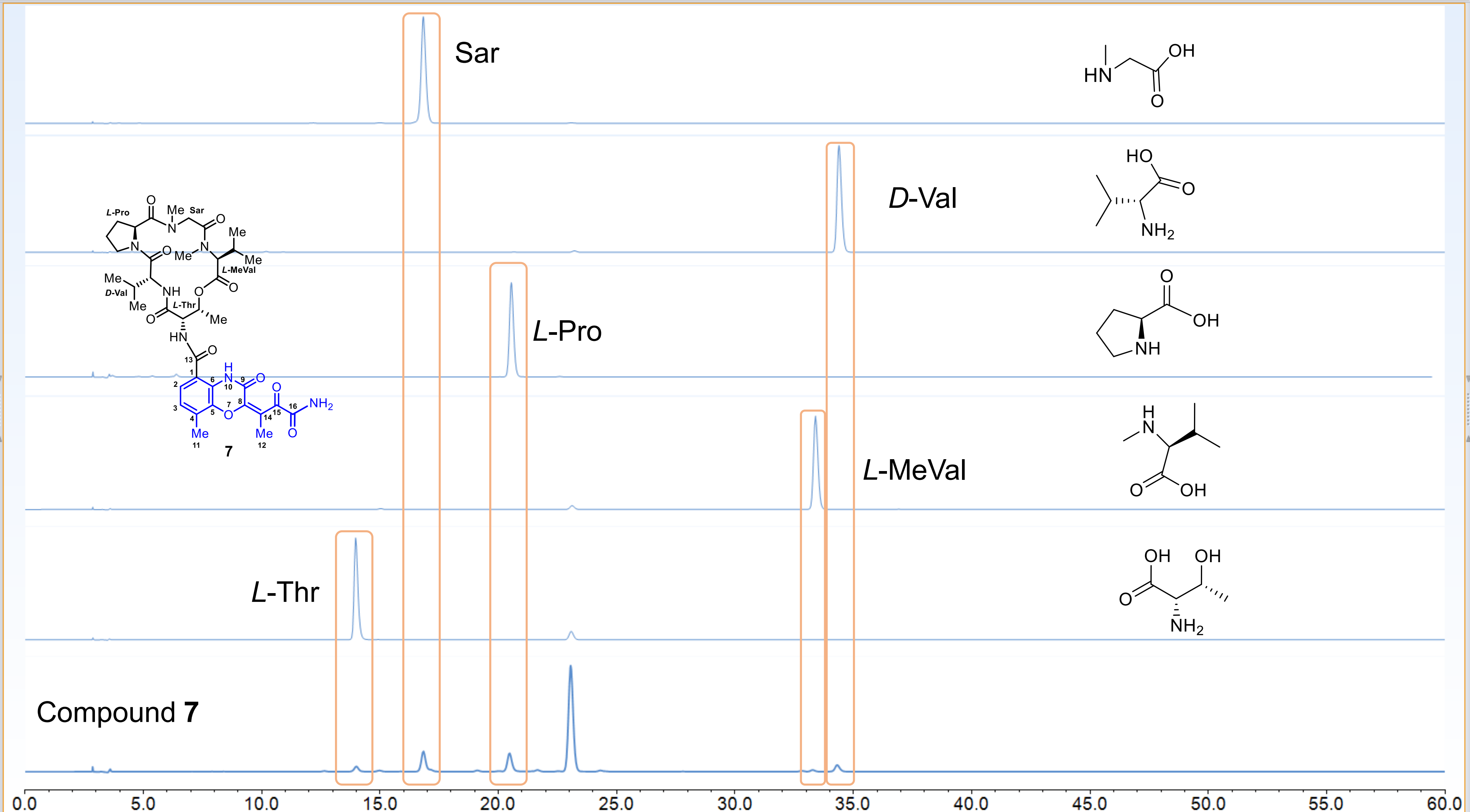

Figure S77. UV spectrum of compound **8**

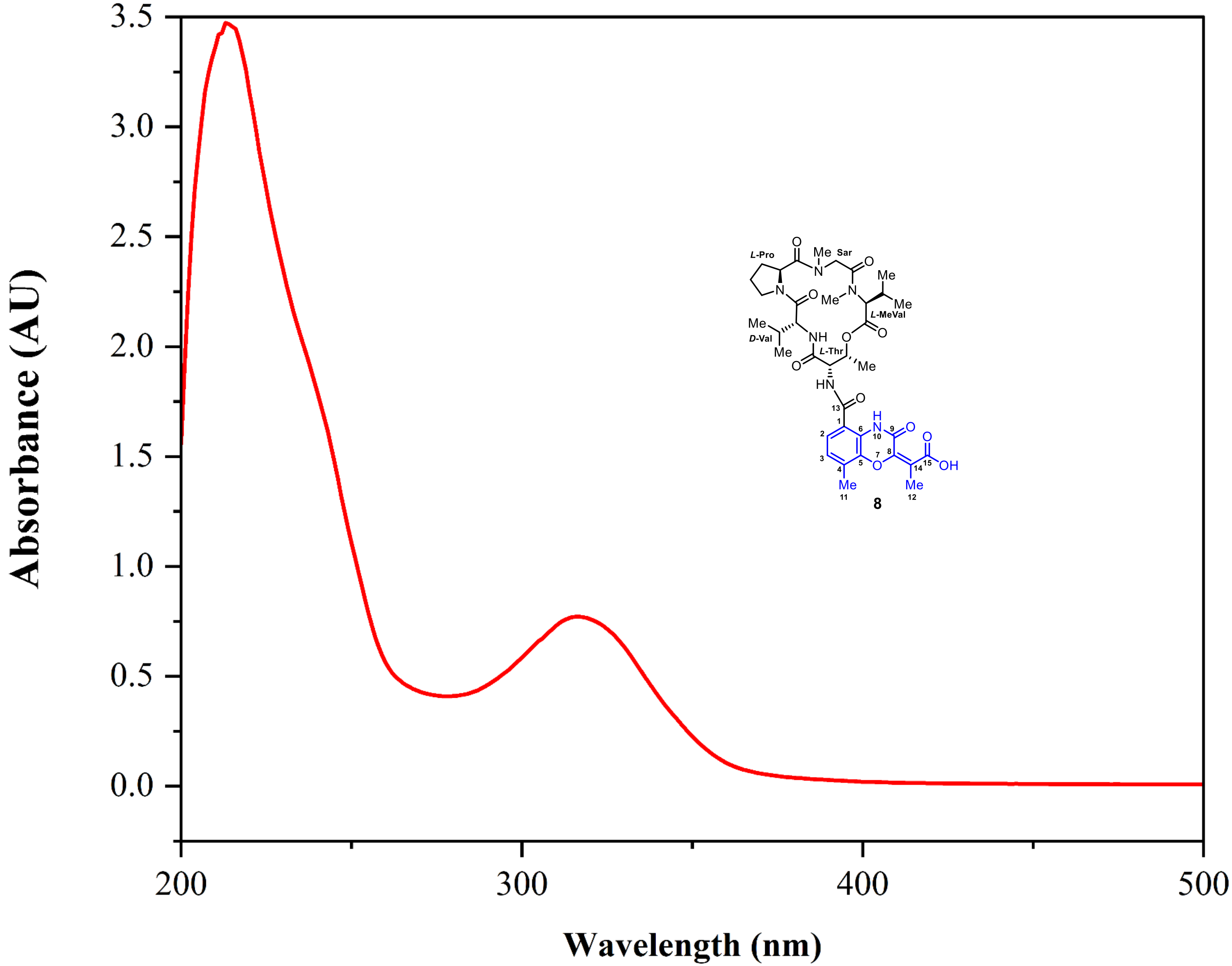

Figure S78. HR-ESIMS for compound **8**

Mass Spectrum SmartFormula Report

Analysis Info

Analysis Name	D:\Data\MS\data\202505\panghe_S-11-0_pos_43_01_19982.d	Acquisition Date	5/29/2025 5:54:42 PM
Method	LC_Direct Infusion_pos_100-1000mz.m	Operator	SCSIO
Sample Name	panghe_S-11-0_pos	Instrument	maXis 255552.00029
Comment			

Acquisition Parameter

Source Type	ESI	Ion Polarity	Positive	Set Nebulizer	0.4 Bar
Focus	Active	Set Capillary	4500 V	Set Dry Heater	180 °C
Scan Begin	100 m/z	Set End Plate Offset	-500 V	Set Dry Gas	4.0 l/min
Scan End	2000 m/z	Set Charging Voltage	0 V	Set Divert Valve	Waste
		Set Corona	0 nA	Set APCI Heater	0 °C

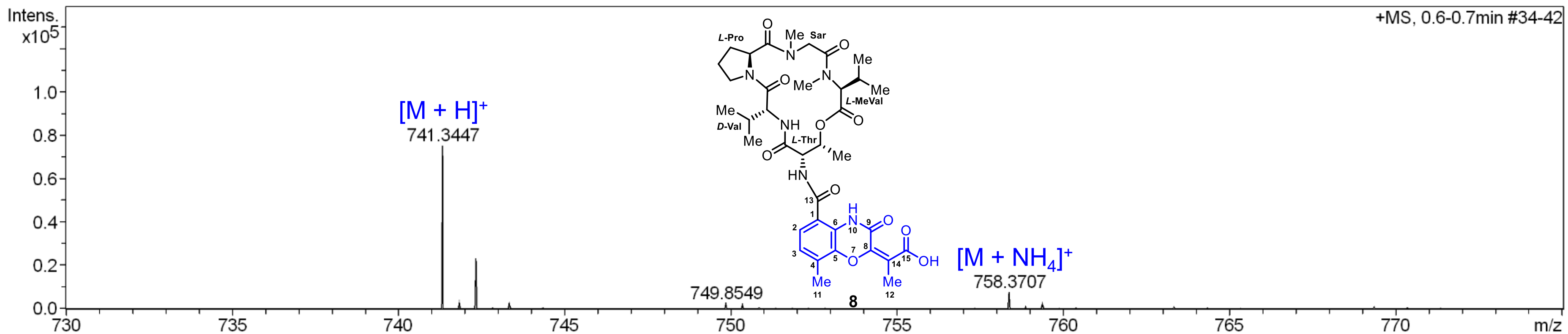

Meas. m/z	#	Ion Formula	Score	m/z	err [ppm]	err [mDa]	mSigma	rdb	e⁻ Conf	N-Rule
741.3447	1	C36H49N6O11	100.00	741.3454	-0.9	-0.7	67.1	15.5	even	ok
758.3707	1	C36H52N7O11	100.00	758.3719	-1.6	-1.2	63.8	14.5	even	ok
763.3251	1	C36H48N6NaO11	100.00	763.3273	-2.9	-2.2	12.6	15.5	even	ok

Figure S79. ^{1}H NMR (600 MHz) spectrum of compound **8** in DMSO-d_6

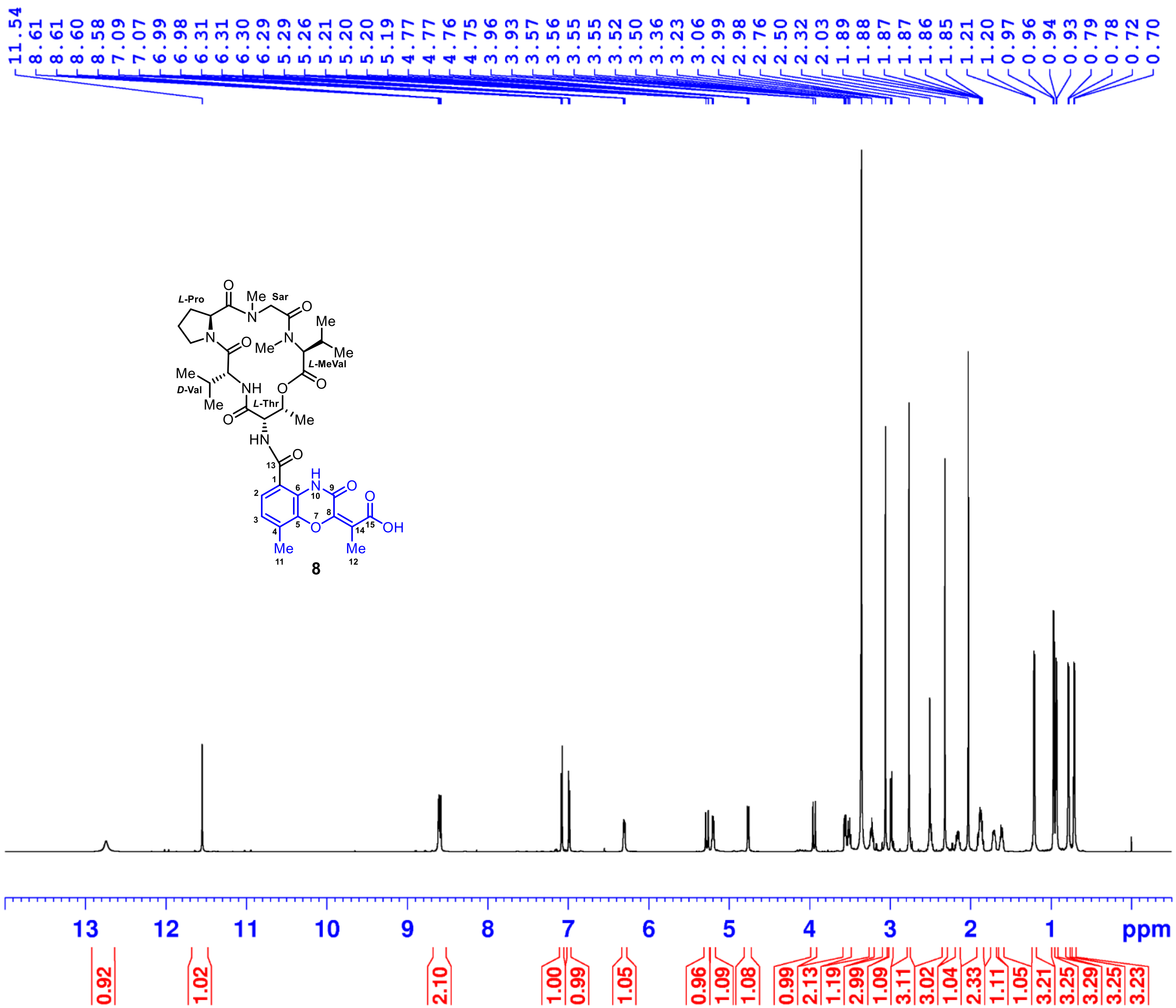

NAME	2531_S11_H
EXPNO	98
PROCNO	1
Date_	20250307
Time	3.30 h
INSTRUM	Avance NEO 600
PROBHD	Z154705_0139 (
PULPROG	zg30
TD	65536
SOLVENT	DMSO
NS	8
DS	2
SWH	11904.762 Hz
FIDRES	0.363304 Hz
AQ	2.7525620 sec
RG	57
DW	42.000 usec
DE	8.79 usec
TE	293.6 K
D1	1.00000000 sec
TD0	1
SFO1	600.1937062 MHz
NUC1	1H
P0	3.33 usec
P1	10.00 usec
SI	65536
SF	600.1900019 MHz
WDW	EM
SSB	0
LB	0.30 Hz
GB	0
PC	1.00

Figure S79-continue

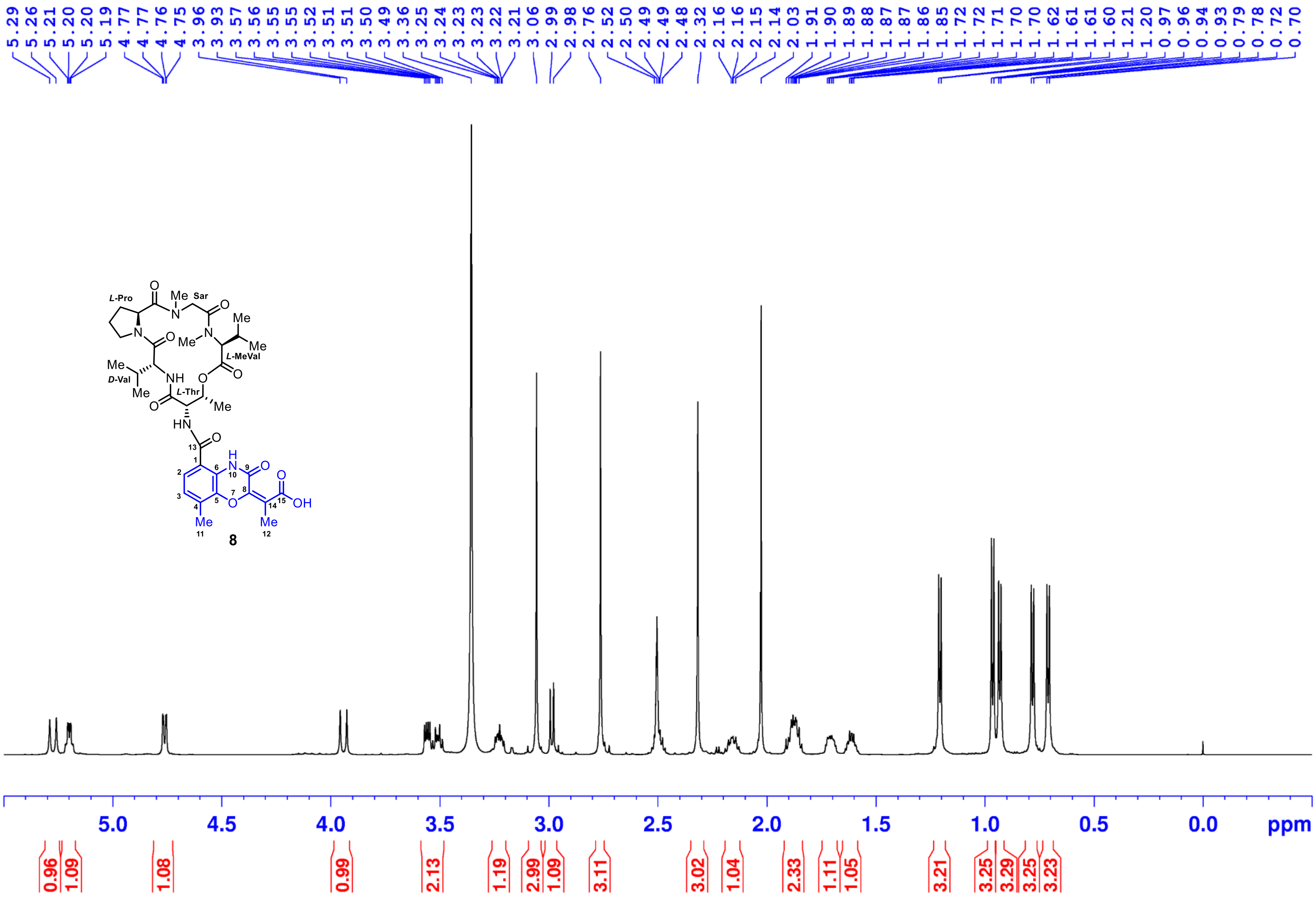

Figure S79-continue

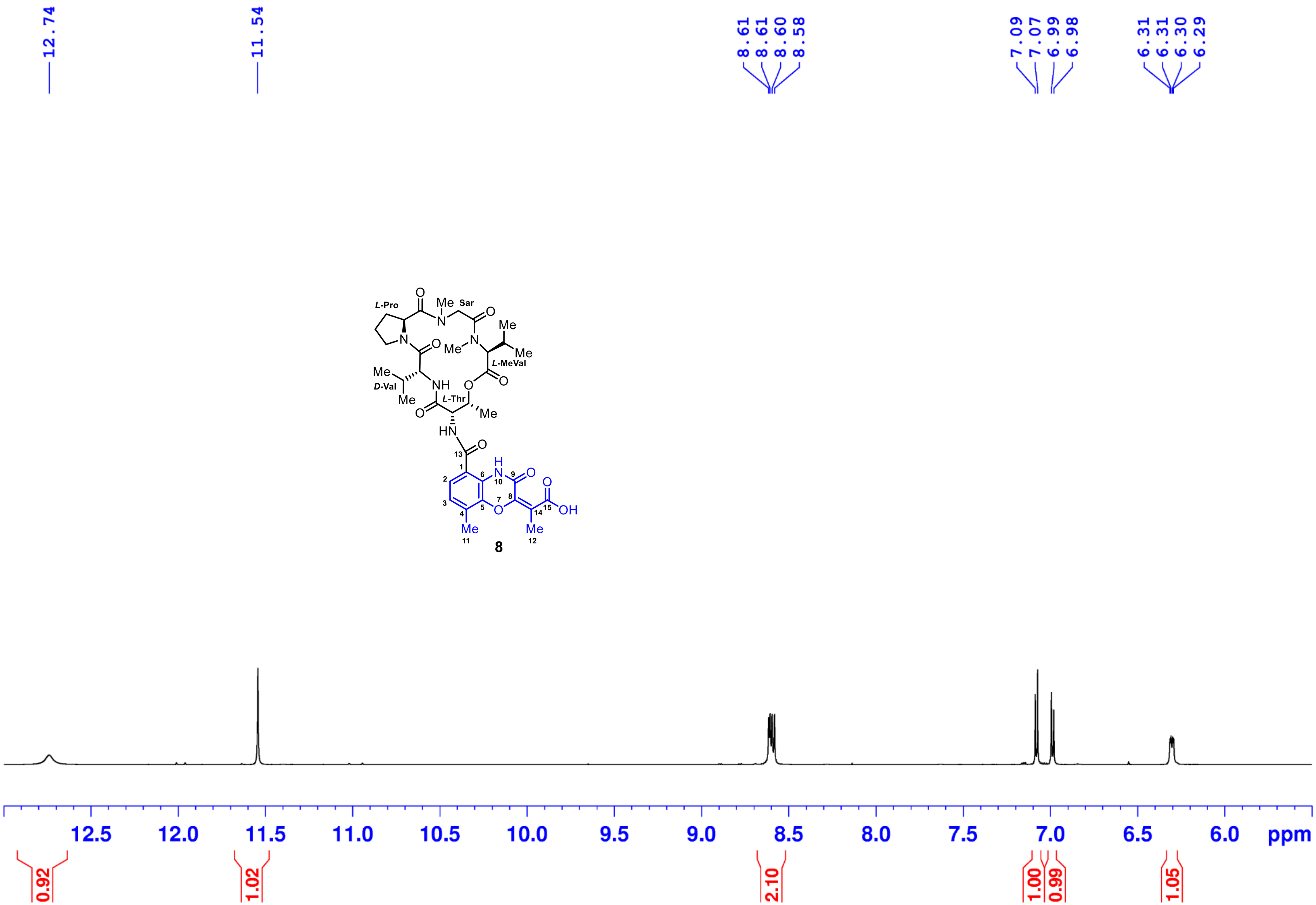

Figure S80. ^{13}C NMR (150 MHz) spectrum of compound **8** in DMSO-d_6

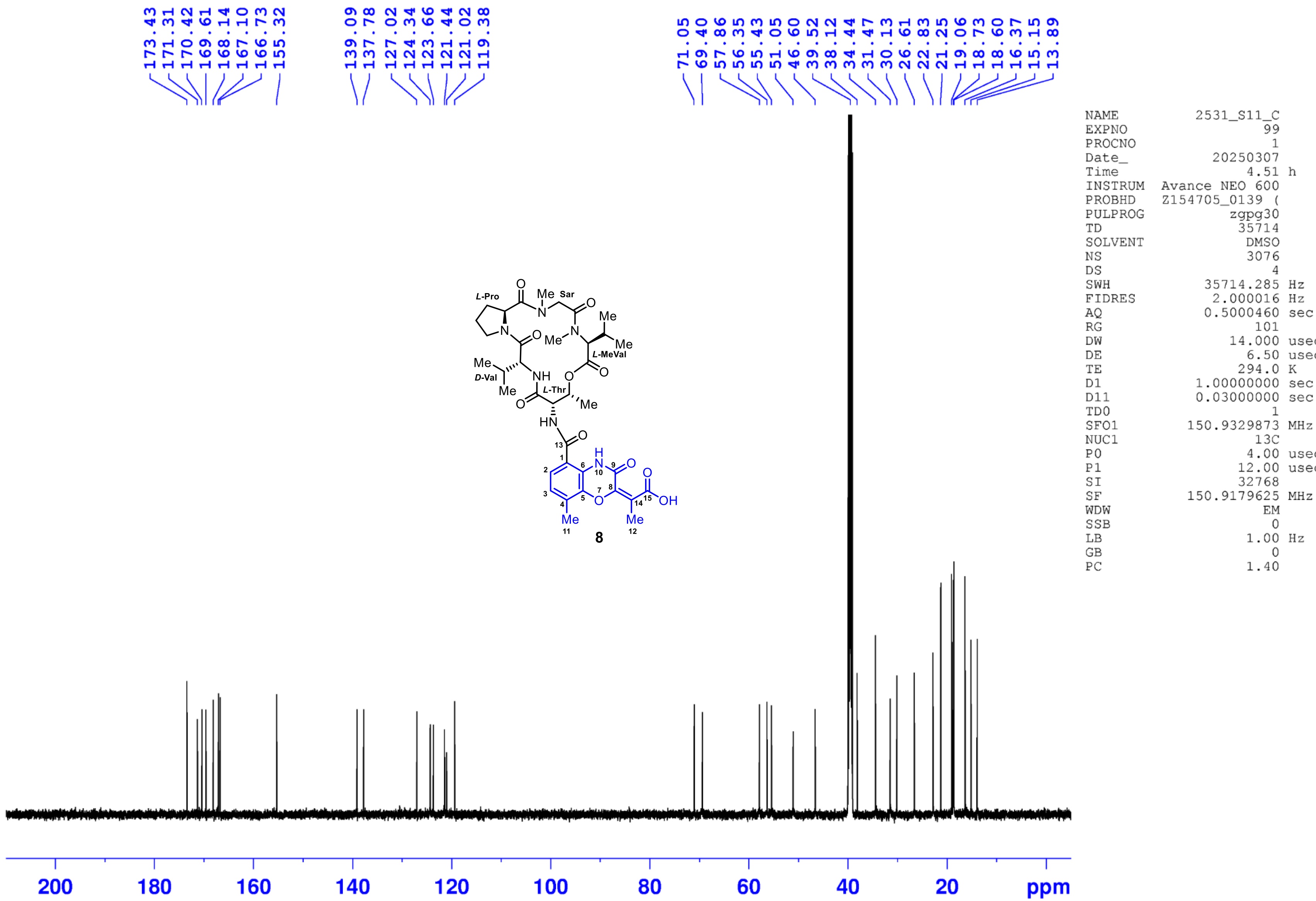

Figure S80-continue

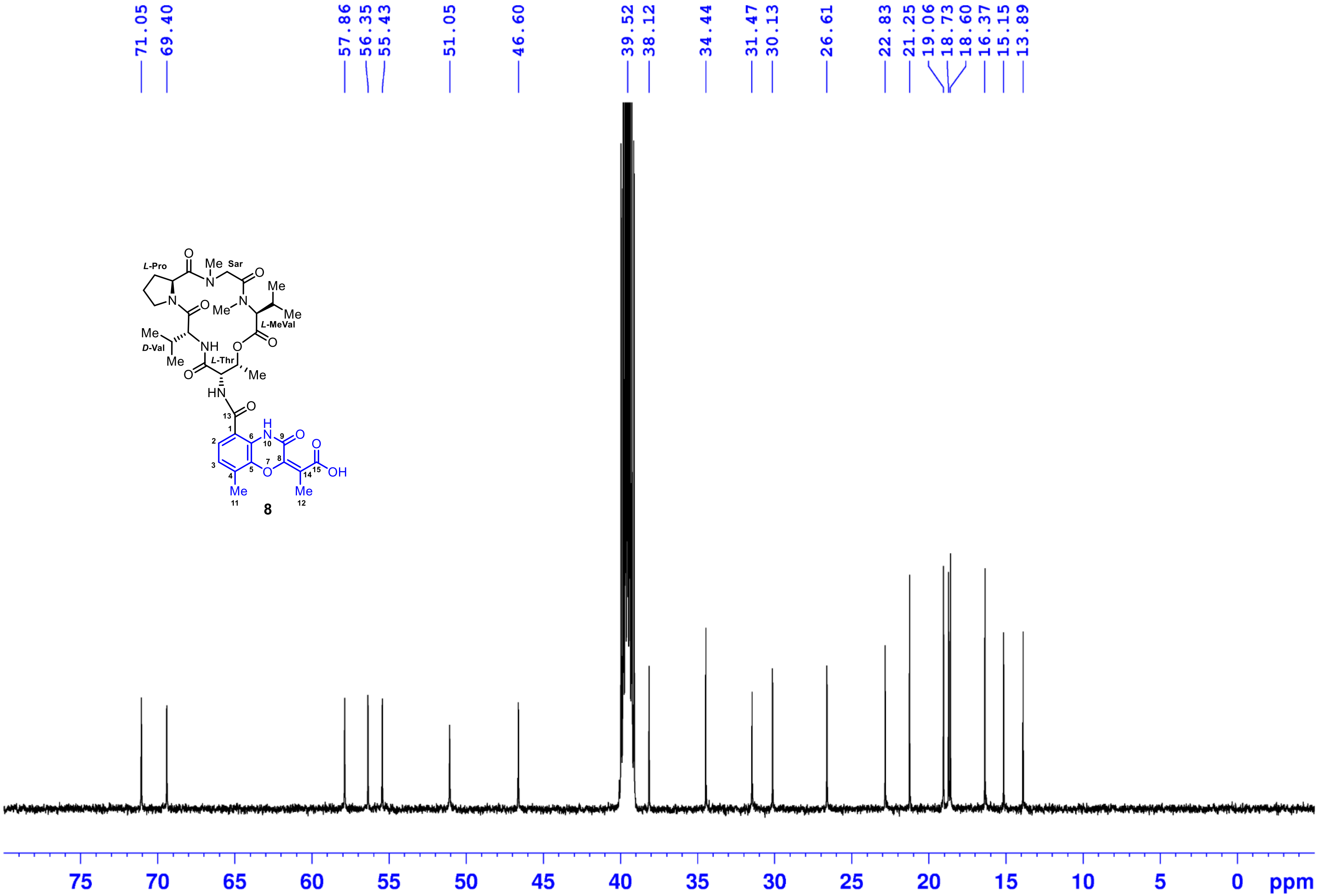

Figure S80-continue

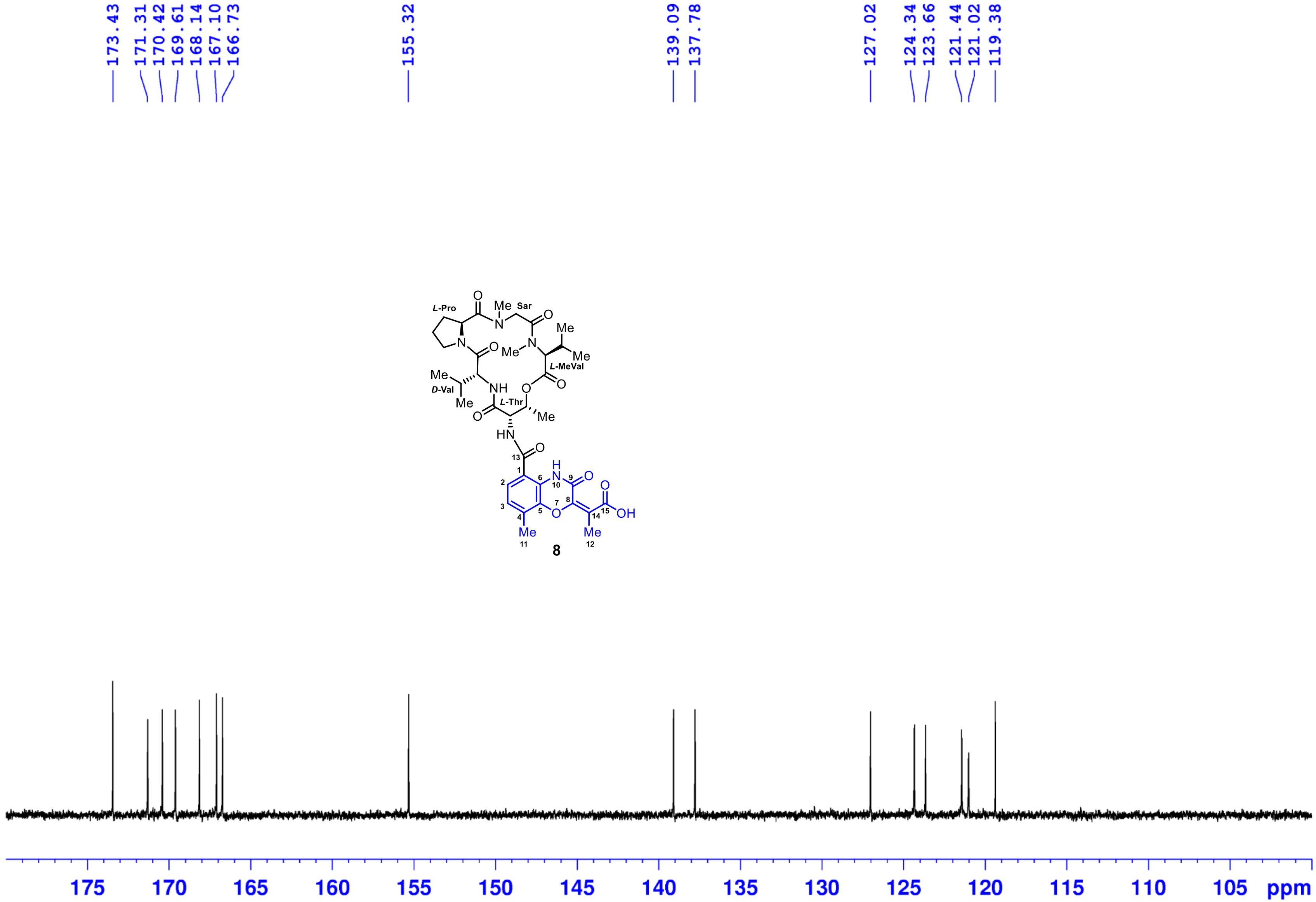

Figure S81. DEPT135 (150 MHz) spectrum of compound **8** in DMSO-d_6

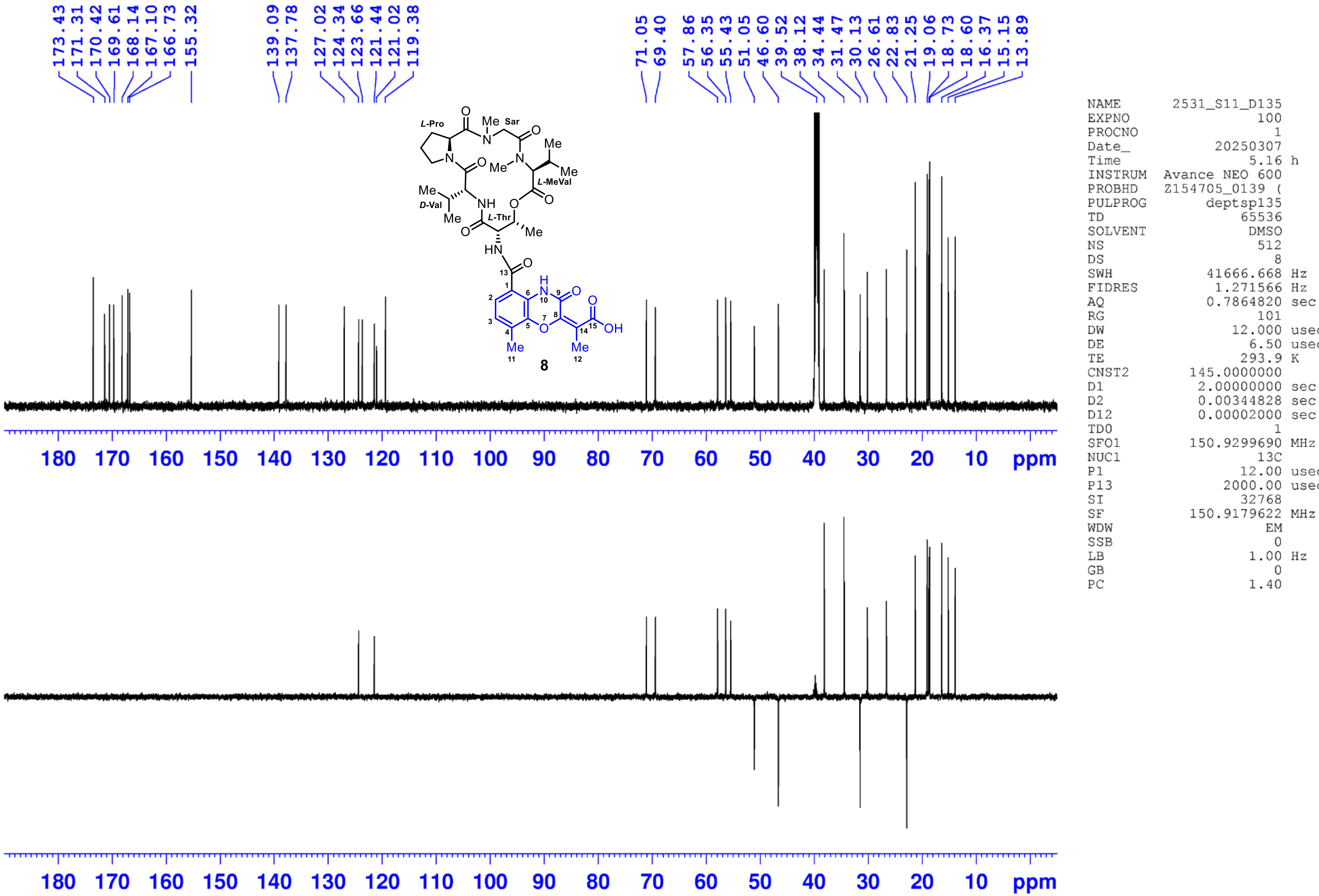

Figure S81-continue

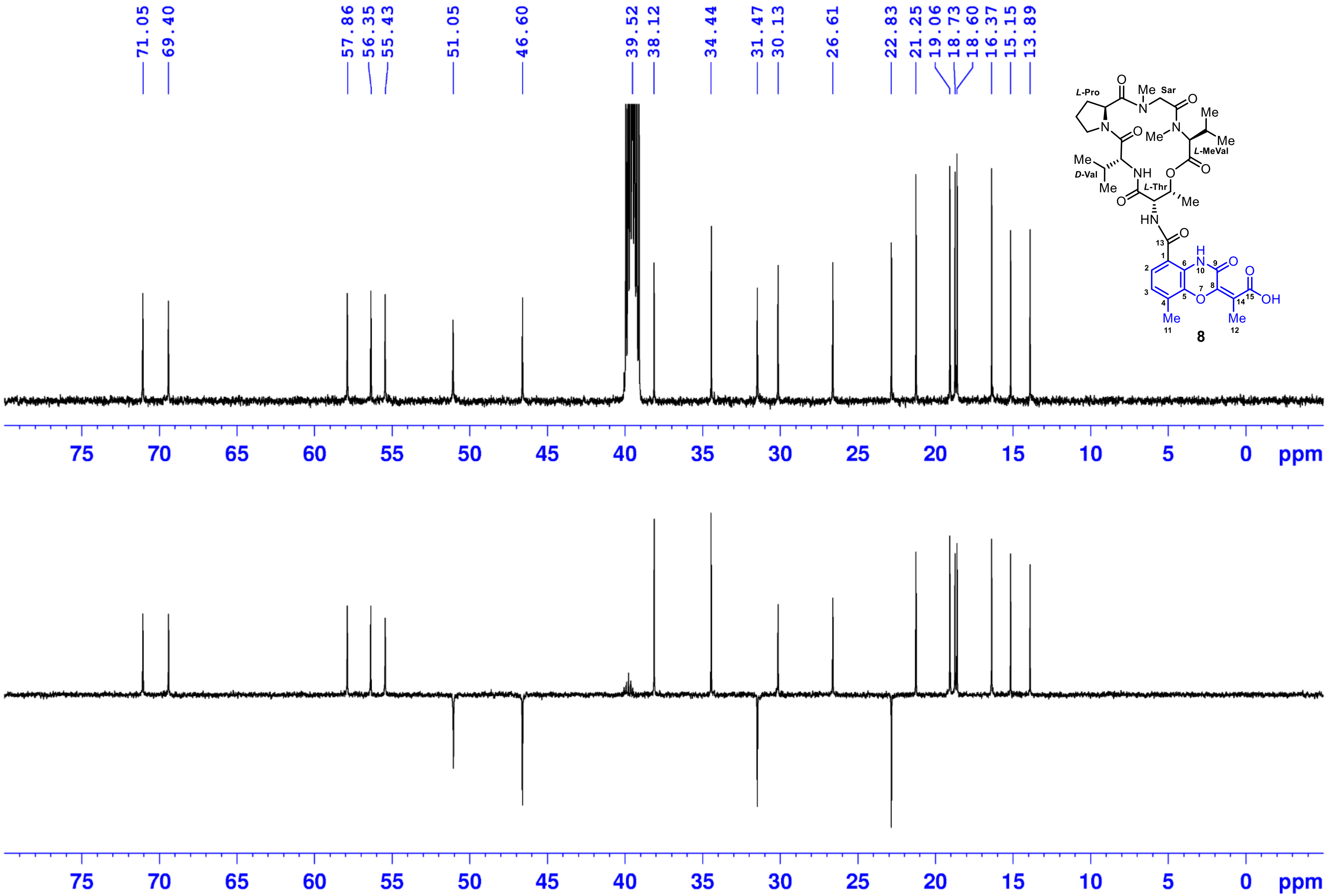

Figure S81-continue

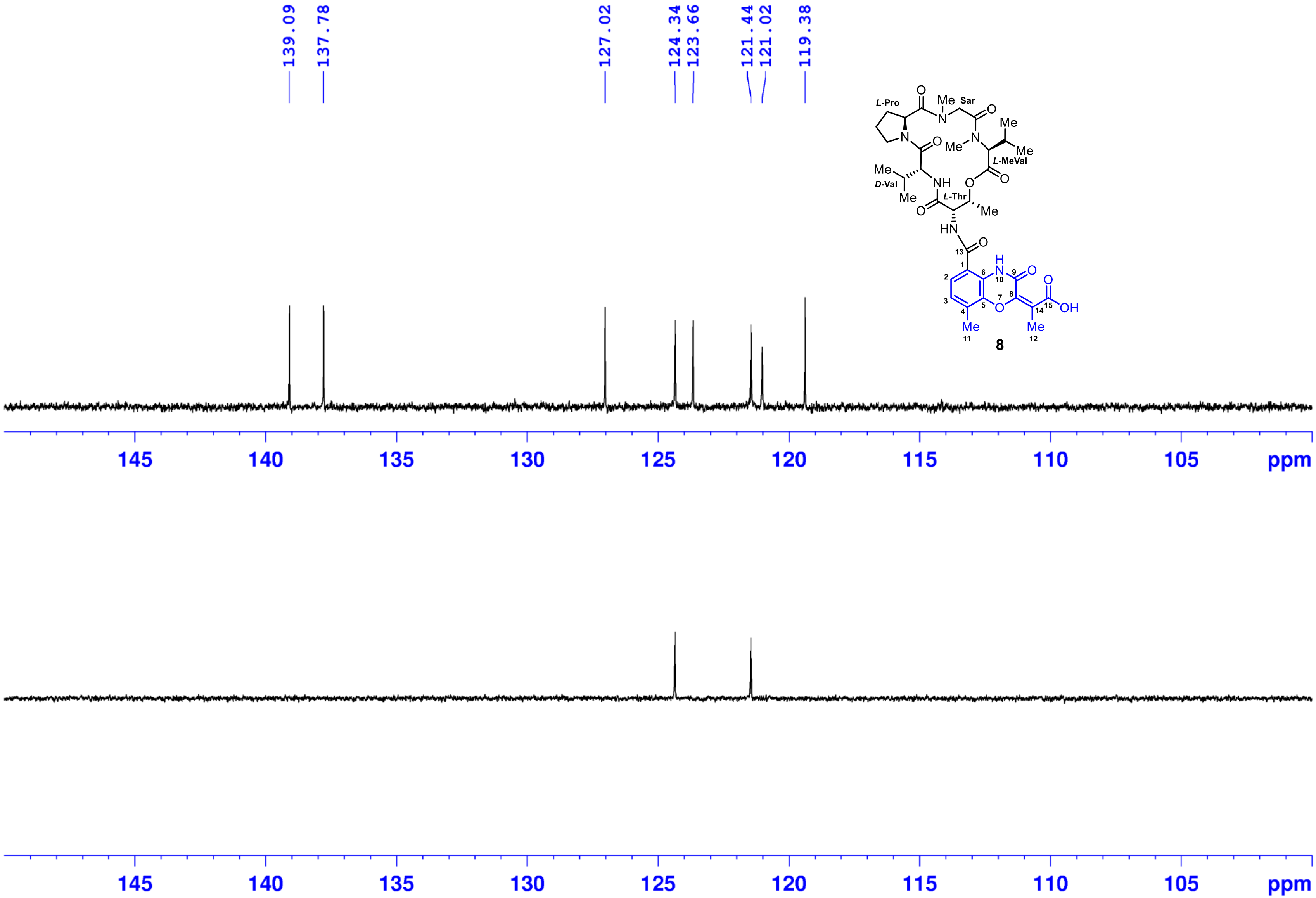

Figure S82. ^{1}H–^{1}H COSY (600 MHz) spectrum of compound **8** in DMSO-d_6

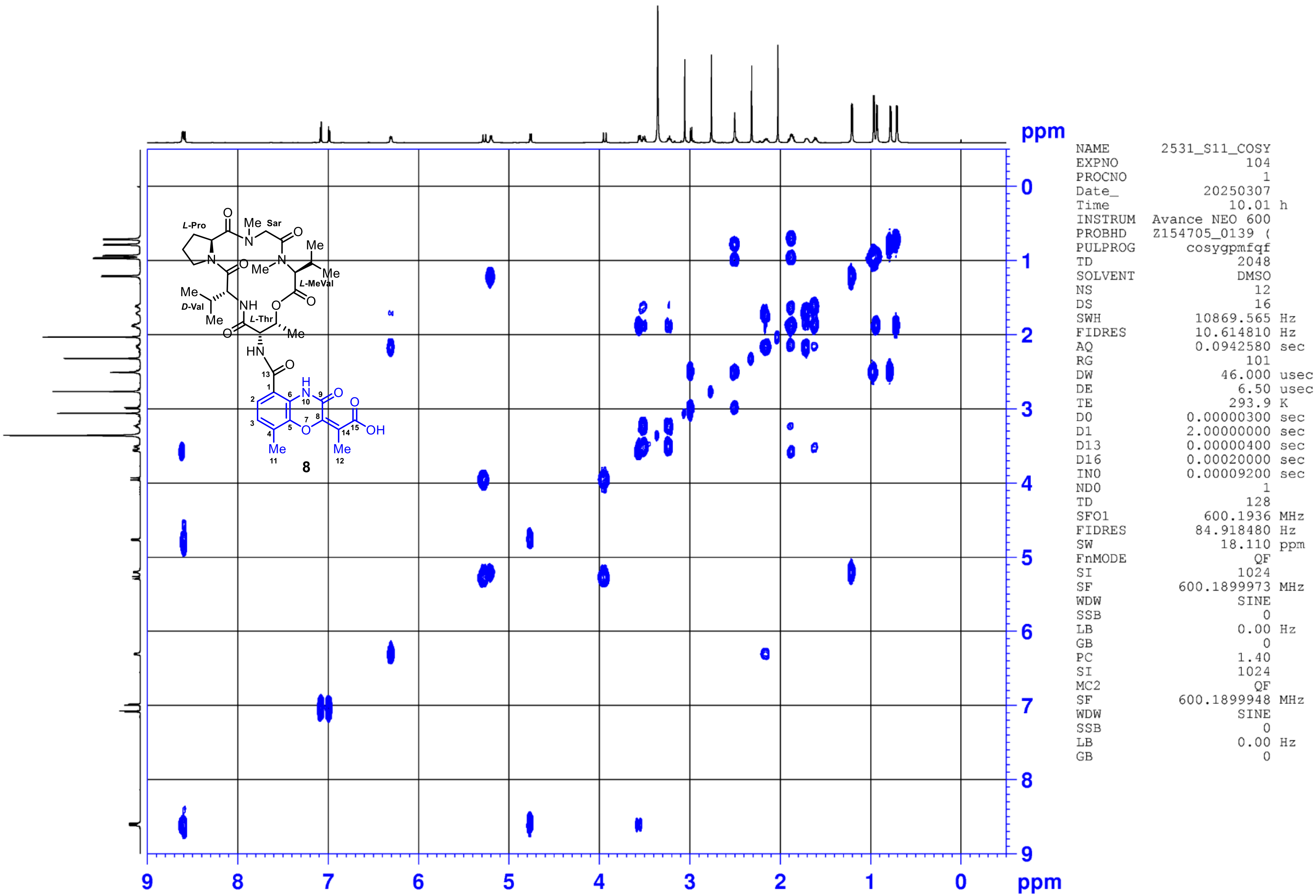

Figure S82-continue

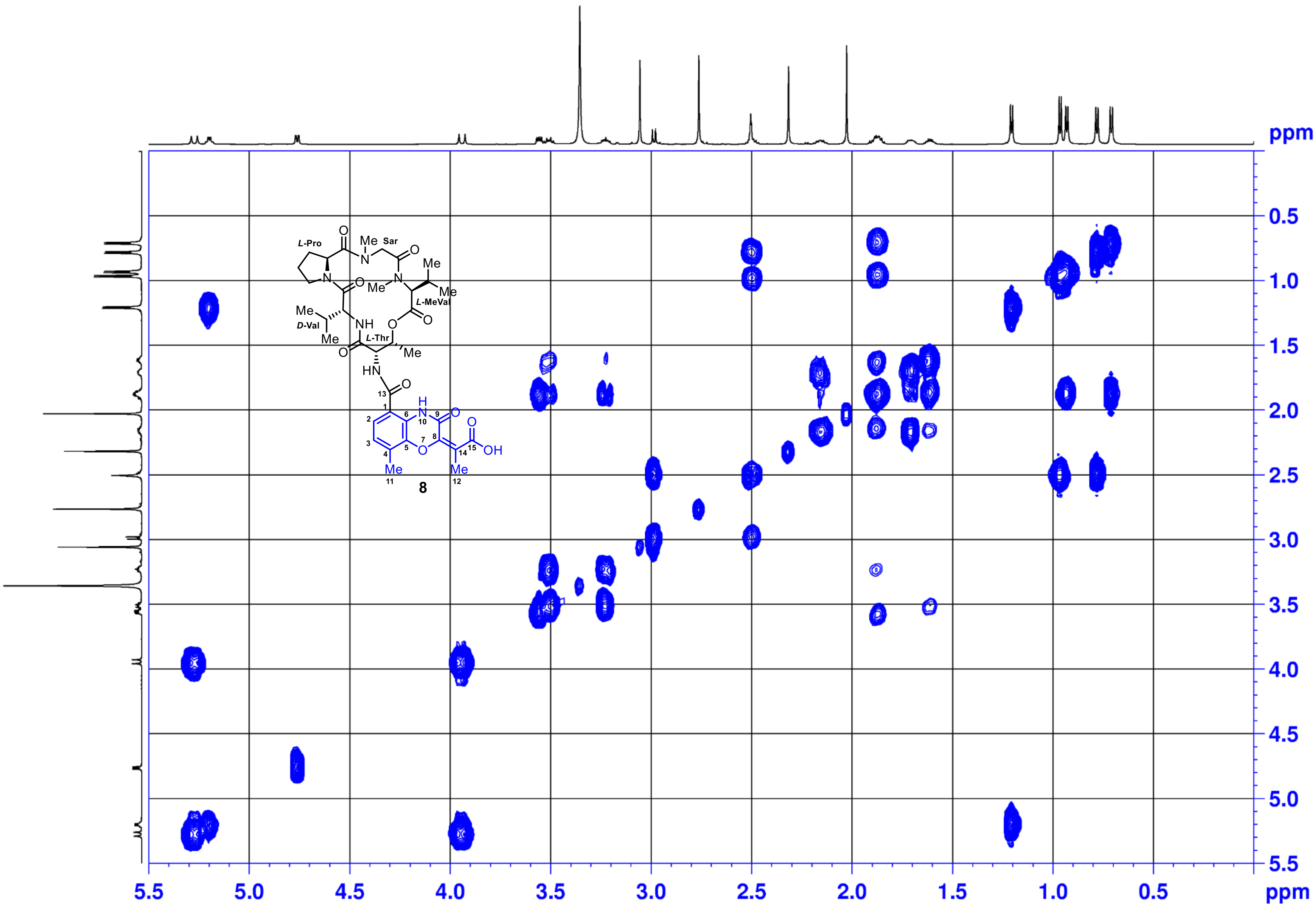

Figure S82-continue

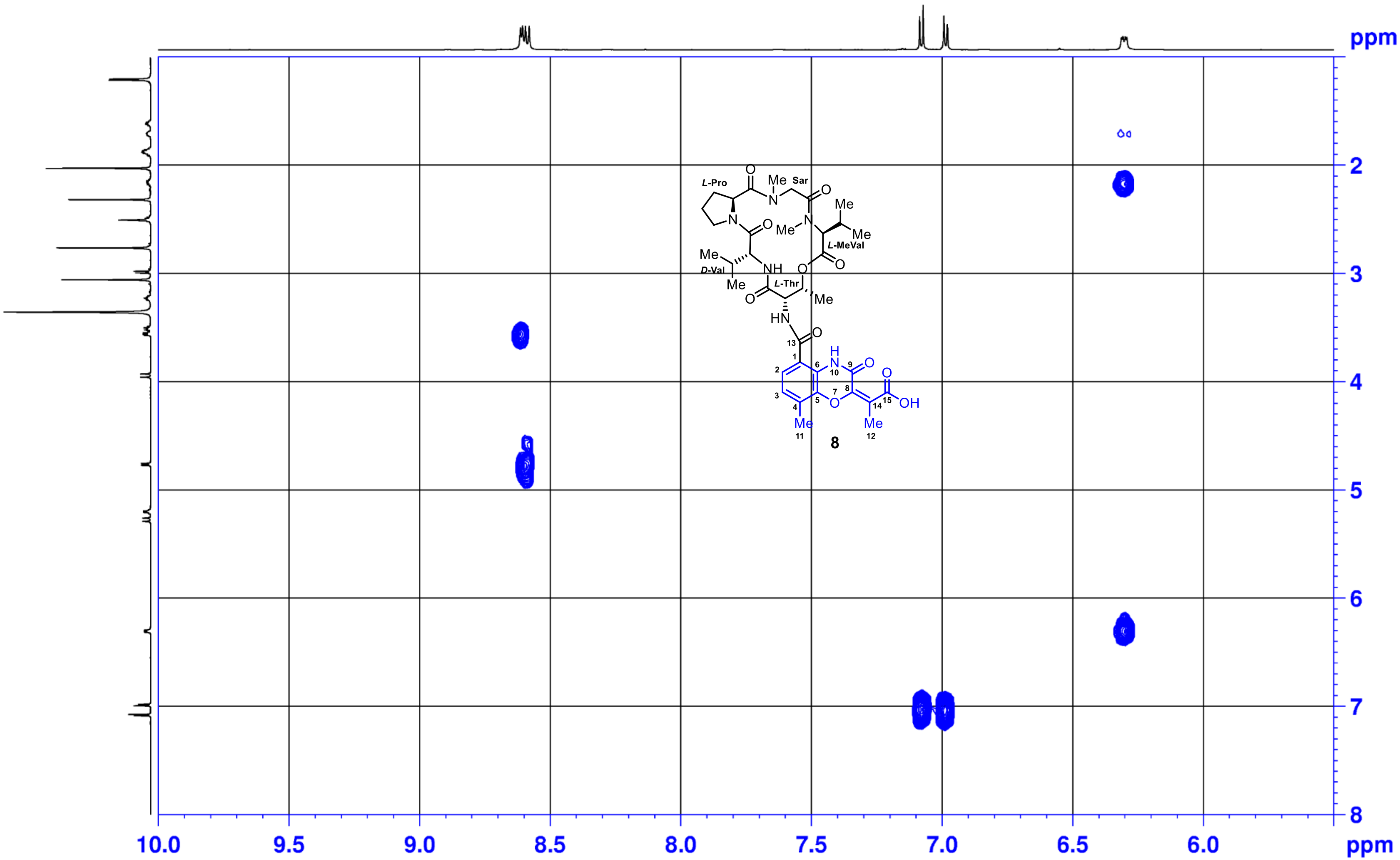

Figure S83. HSQC (600 MHz) spectrum of compound **8** in DMSO-d_6

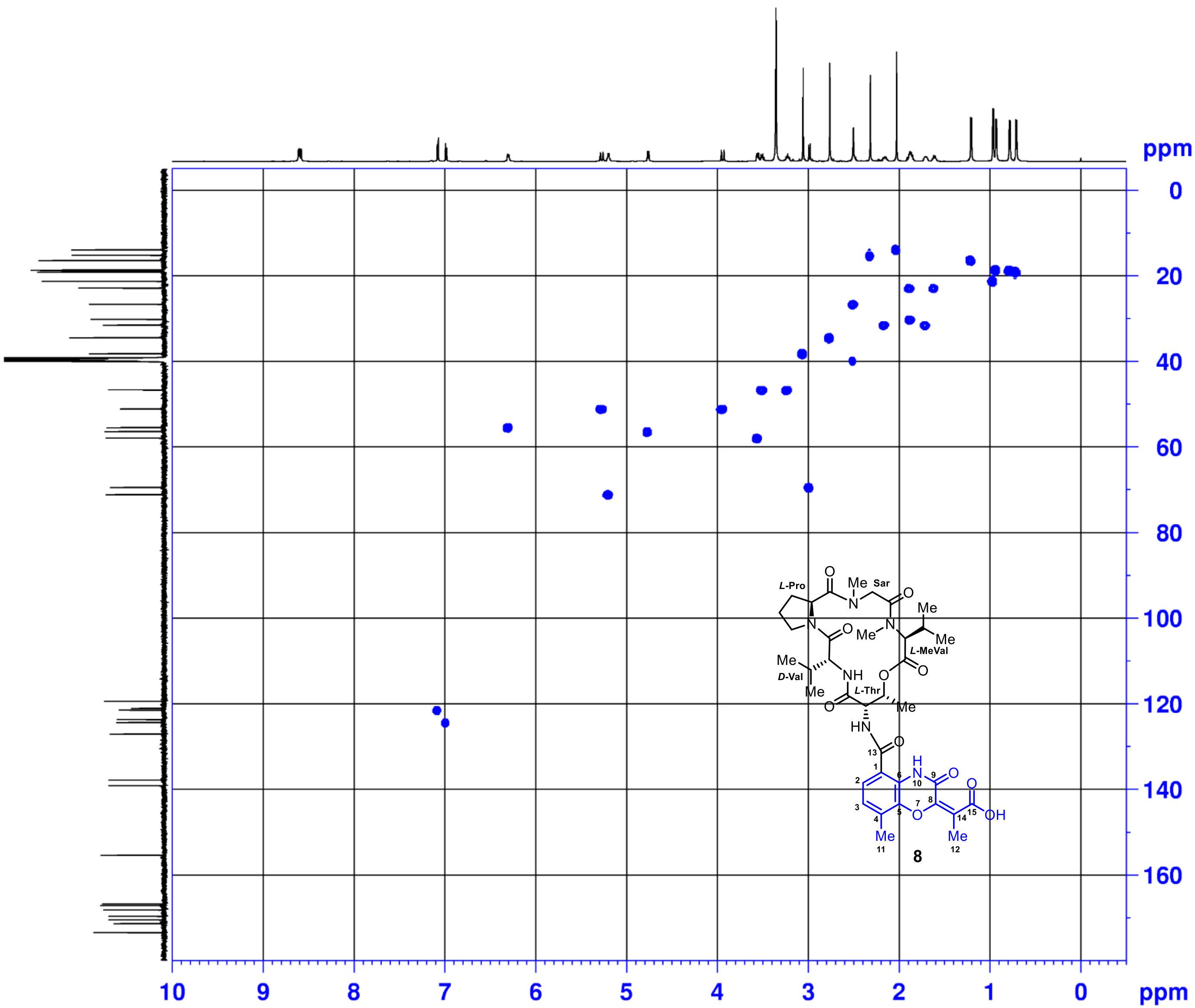

NAME	2531_S11_HSQC	
EXPNO	101	
PROCNO	1	
Date_	20250307	
Time	6.10	h
INSTRUM	Avance NEO 600	
PROBHD	Z154705_0139 (	
PULPROG	hsqcetgp	
TD	1024	
SOLVENT	DMSO	
NS	8	
DS	16	
SWH	10869.565	Hz
FIDRES	21.229620	Hz
AQ	0.0471540	sec
RG	101	
DW	46.000	usec
DE	6.50	usec
TE	293.7	K
CNST2	145.0000000	
D0	0.00000300	sec
D1	1.50000000	sec
D4	0.00172414	sec
D11	0.03000000	sec
D16	0.00020000	sec
IN0	0.00001142	sec
ND0	2	
TD	256	
SFO1	150.9292	MHz
FIDRES	170.974503	Hz
SW	290.000	ppm
FnMODE	Echo-Antiecho	
SI	1024	
SF	600.1899958	MHz
WDW	QSINE	
SSB	2	
LB	0.00	Hz
GB	0	
PC	1.40	
SI	1024	
MC2	echo-antiecho	
SF	150.9179445	MHz
WDW	QSINE	
SSB	2	
LB	0.00	Hz
GB	0	

Figure S83-continue

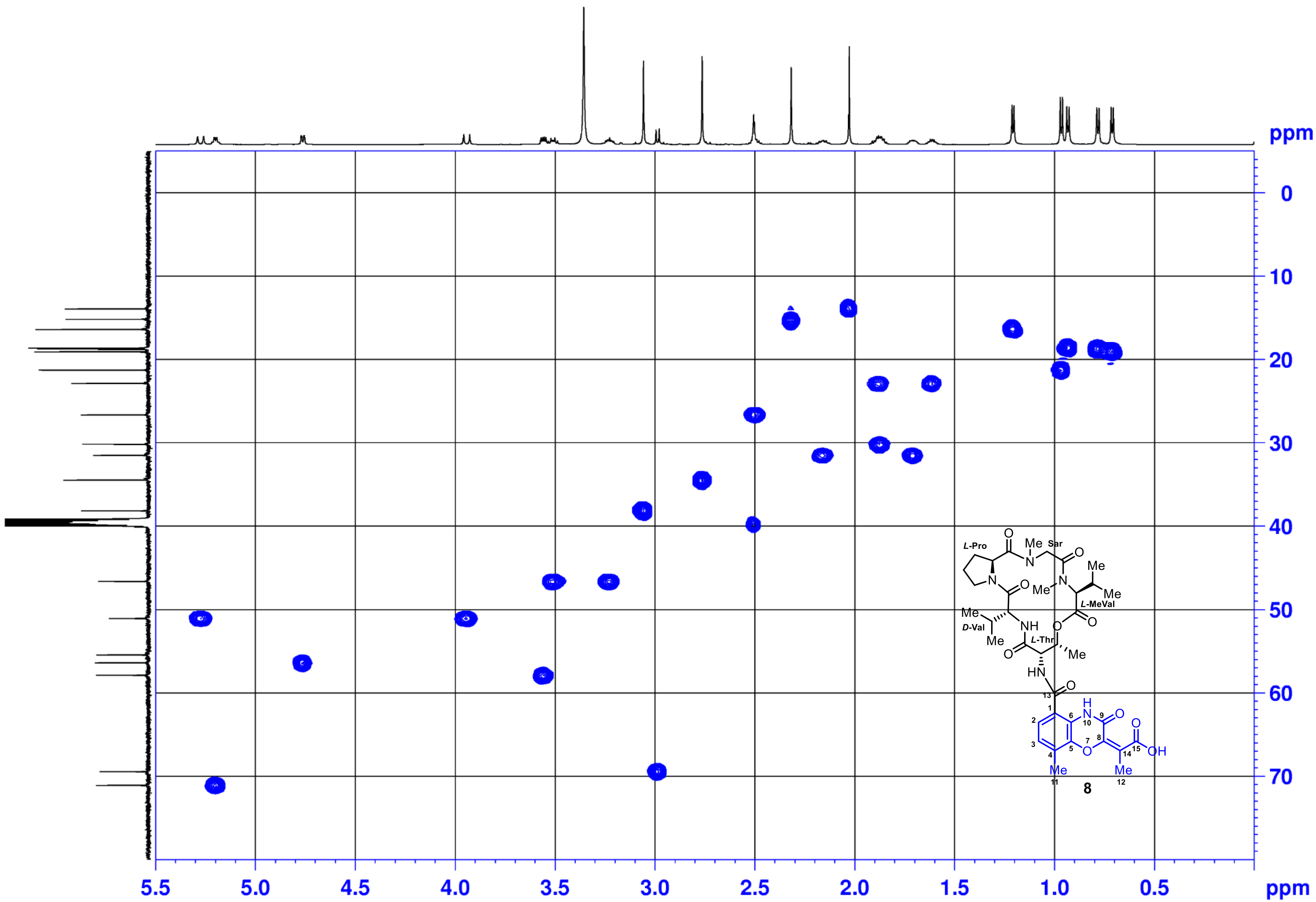

Figure S83-continue

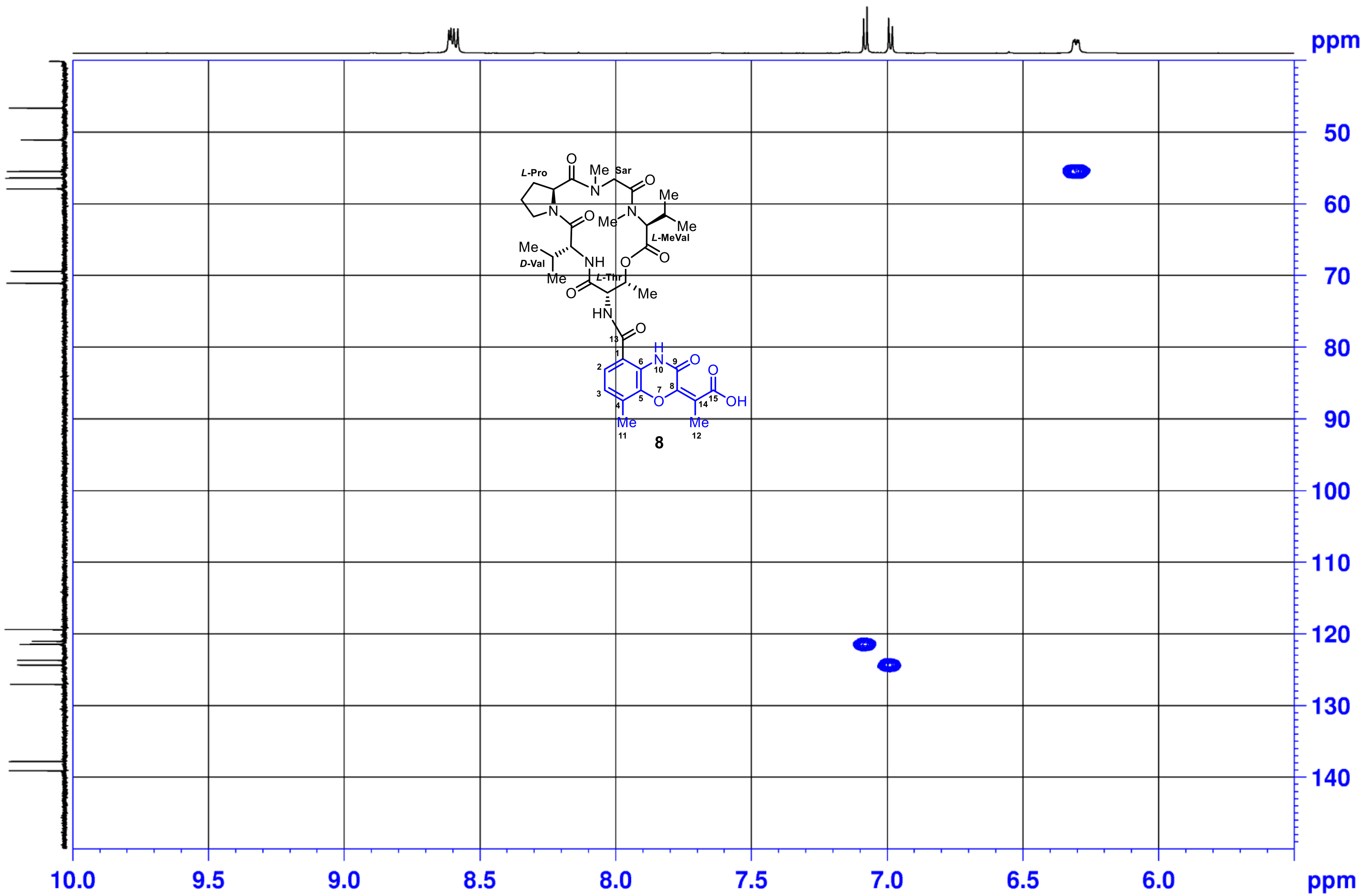

Figure S84. HMBC (600 MHz) spectrum of compound **8** in DMSO-d_6

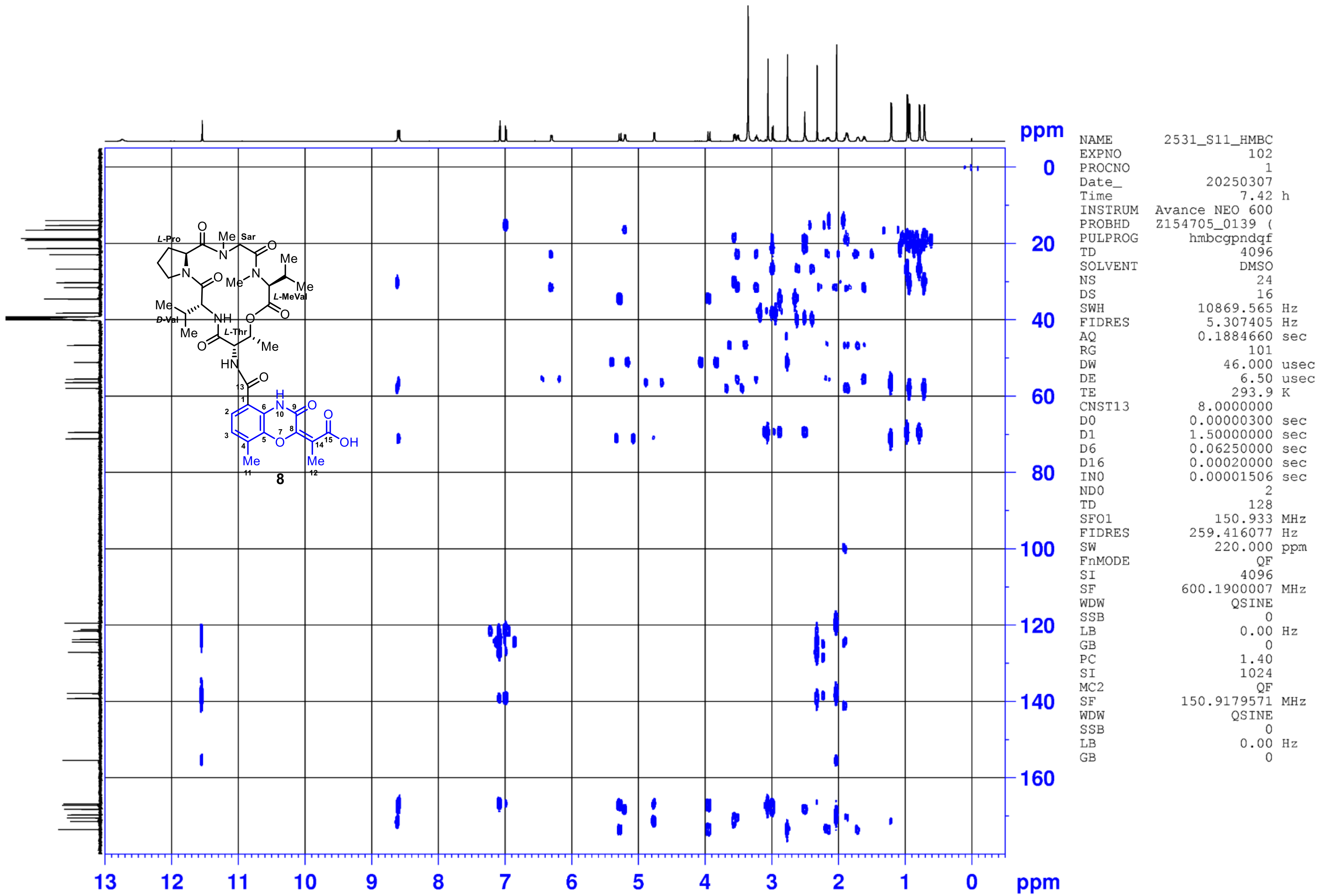

Figure S84-continue

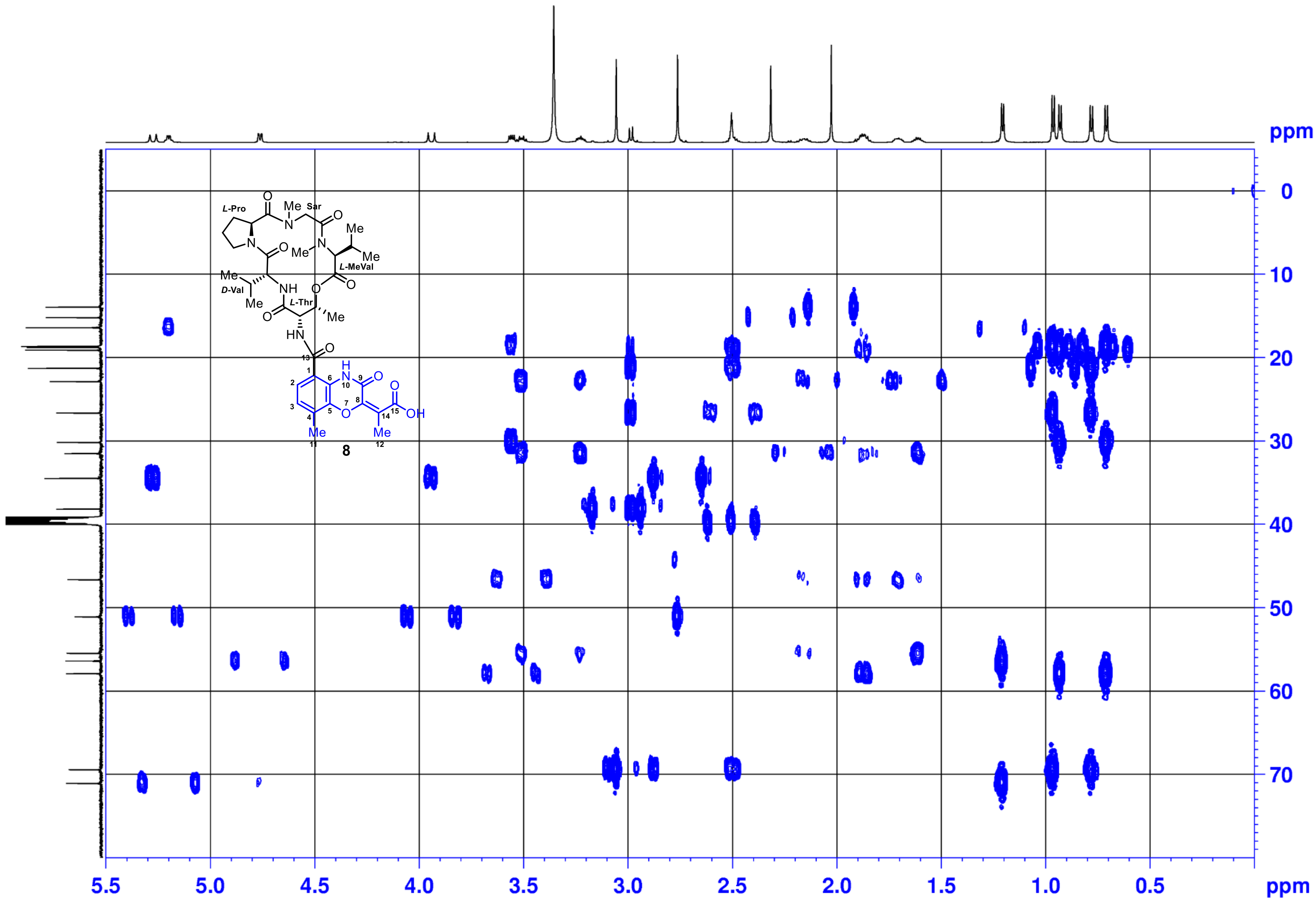

Figure S84-continue

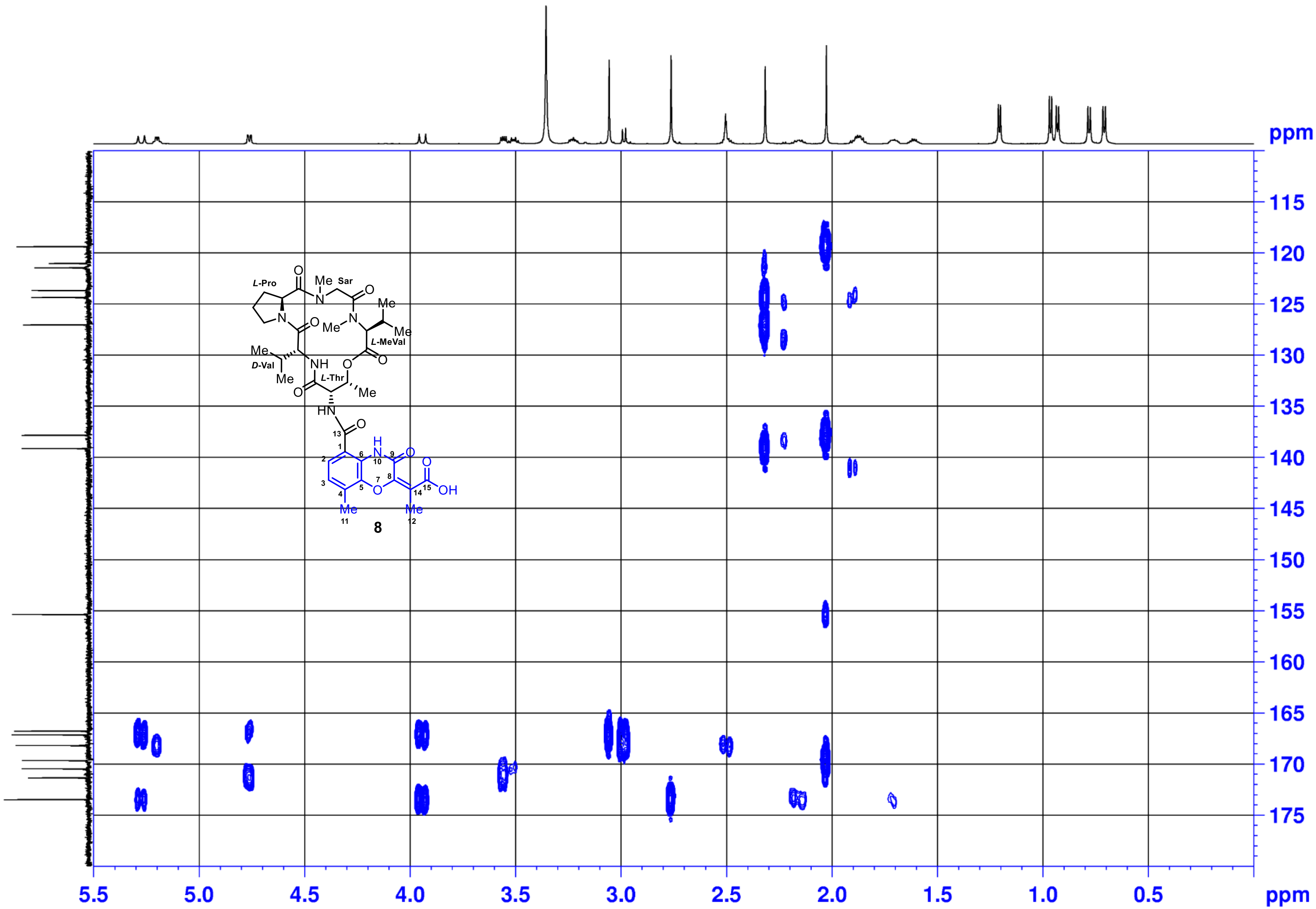

Figure S84-continue

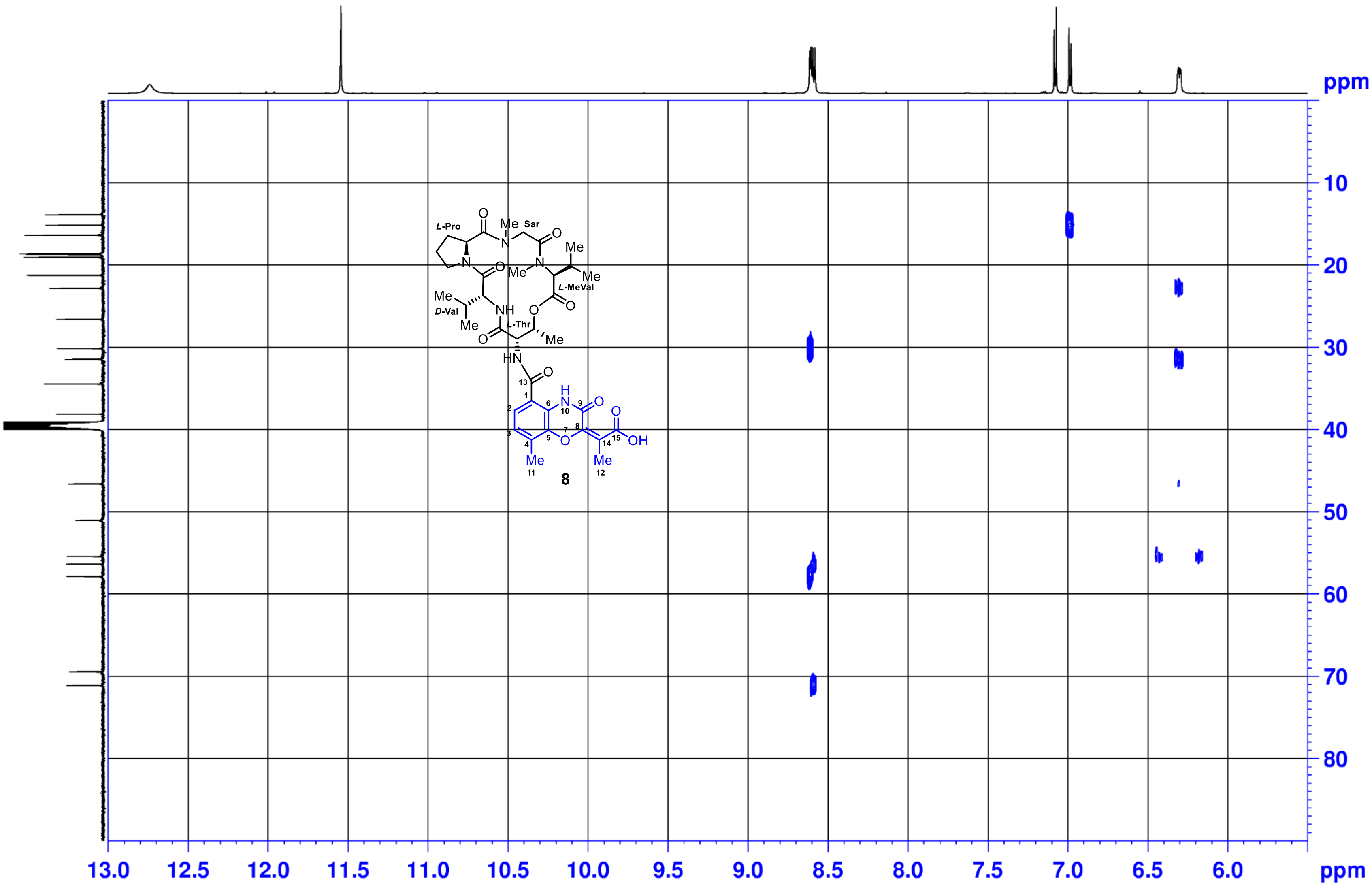

Figure S84-continue

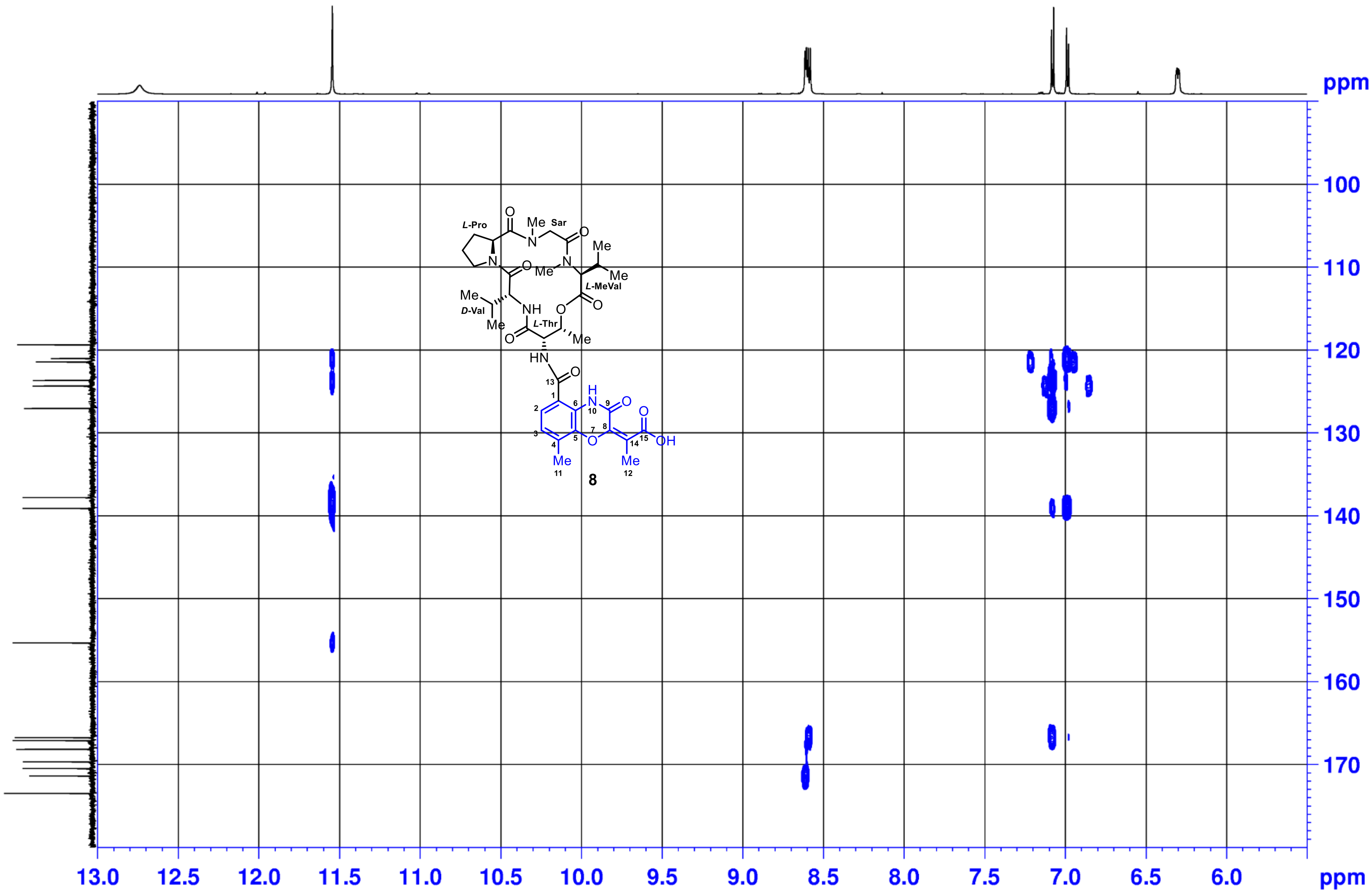

Figure S85. ROESY (600 MHz) spectrum of compound **8** in DMSO-d_6

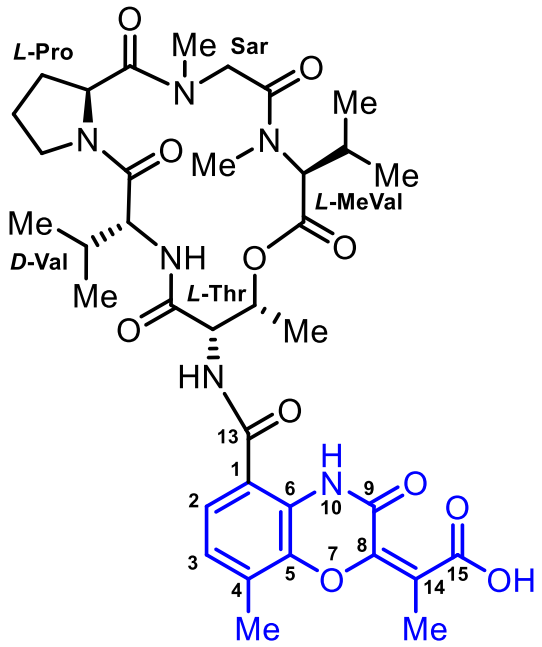

8

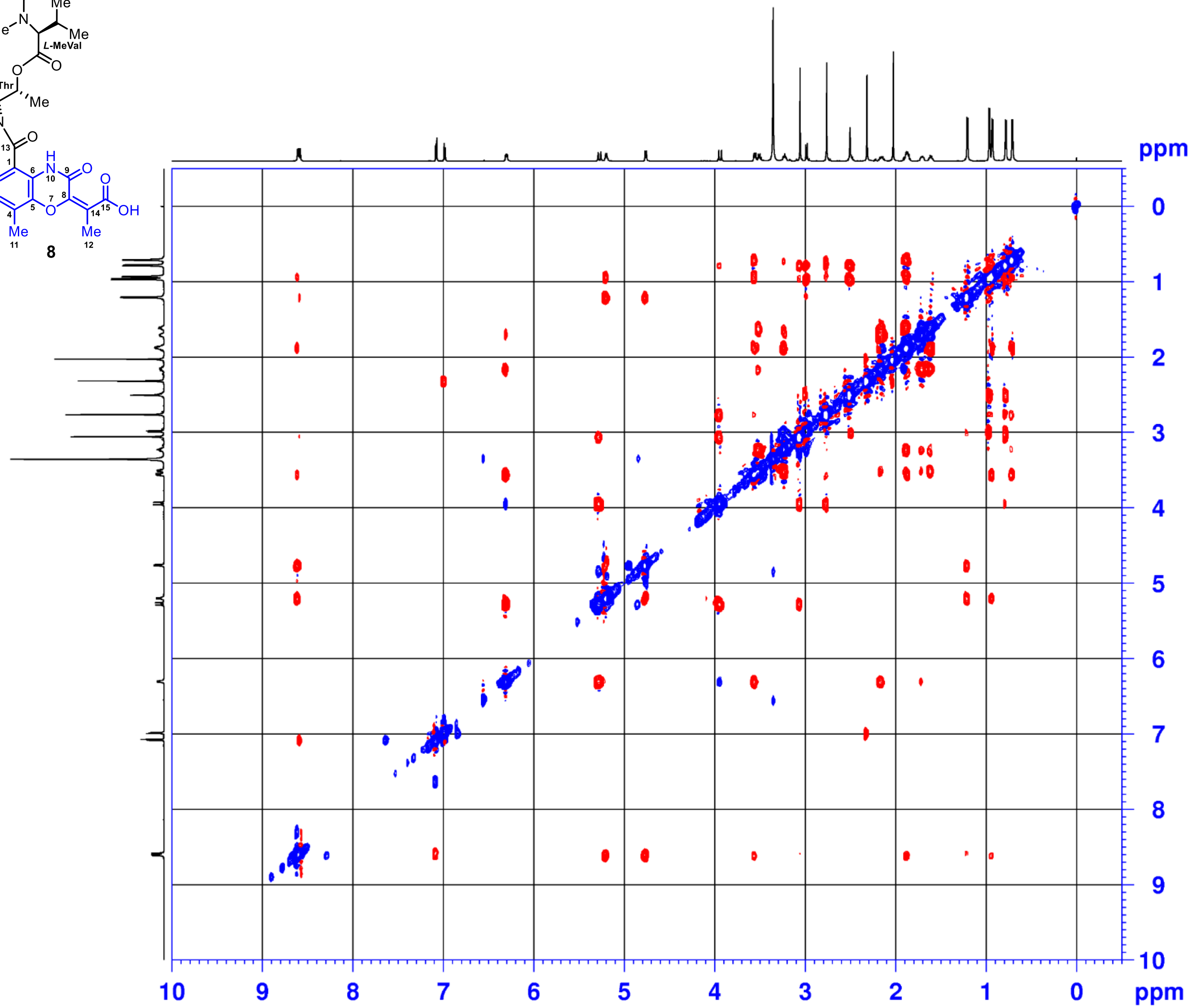

NAME	0526_S_11_1ROE
EXPNO	10
PROCNO	1
Date_	20250527
Time	10.18 h
INSTRUM	Avance NEO 600
PROBHD	Z154705_0139 (
PULPROG	roesyphpp.2
TD	2048
SOLVENT	DMSO
NS	8
DS	32
SWH	13888.889 Hz
FIDRES	13.563368 Hz
AQ	0.0737780 sec
RG	101
DW	36.000 usec
DE	6.50 usec
TE	293.3 K
D0	0.00002650 sec
D1	2.00000000 sec
D11	0.03000000 sec
D12	0.00002000 sec
IN0	0.00007373 sec
L4	556
P15	200000.00 usec
ND0	1
TD	256
SFO1	600.1928 MHz
FIDRES	52.985771 Hz
SW	22.600 ppm
FnMODE	States-TPPI
SI	1024
SF	600.1899936 MHz
WDW	QSINE
SSB	2
LB	0.00 Hz
GB	0
PC	1.00
SI	1024
MC2	States-TPPI
SF	600.1899933 MHz
WDW	QSINE
SSB	2
LB	0.00 Hz
GB	0

Figure S85-continue

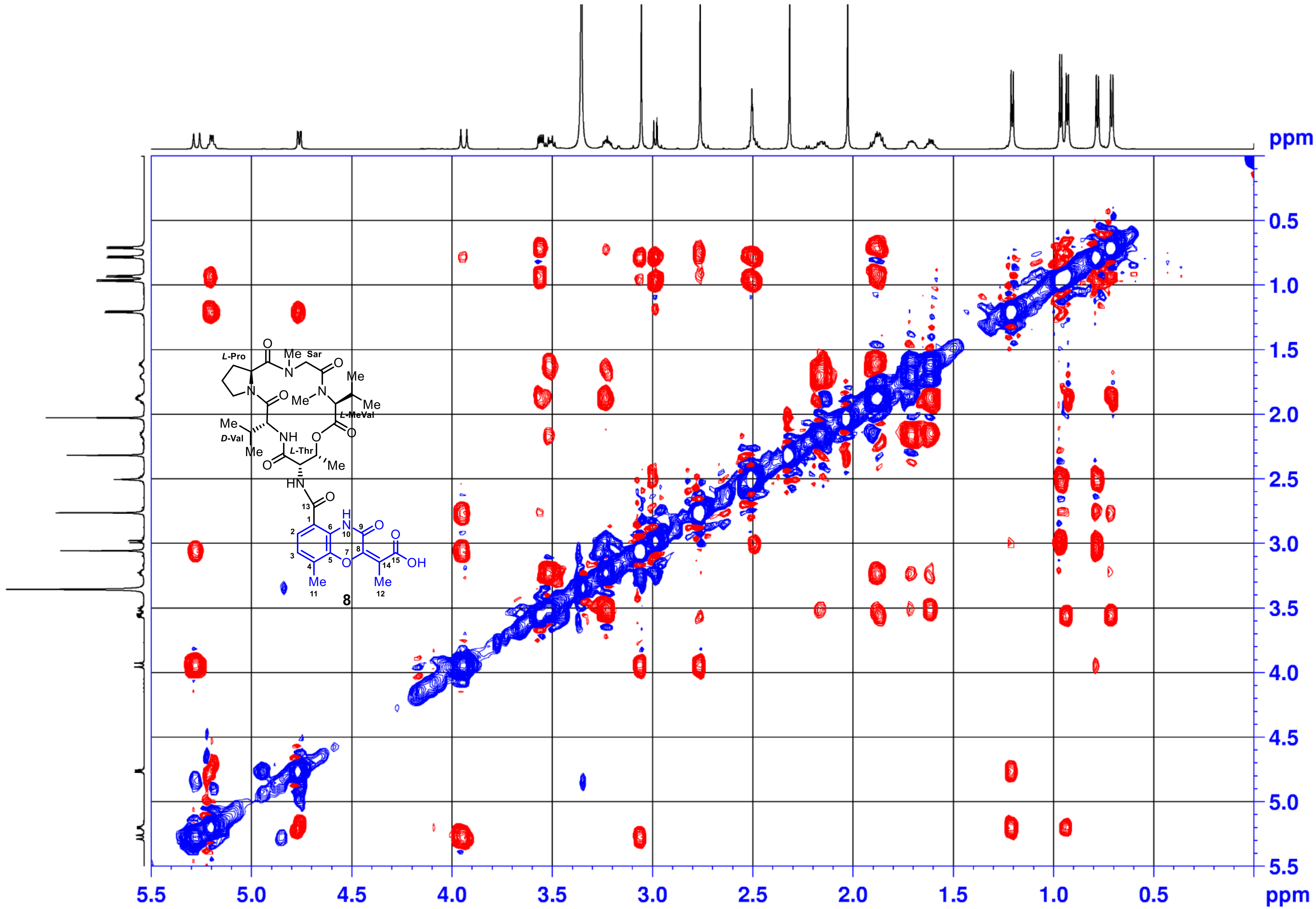

Figure S85-continue

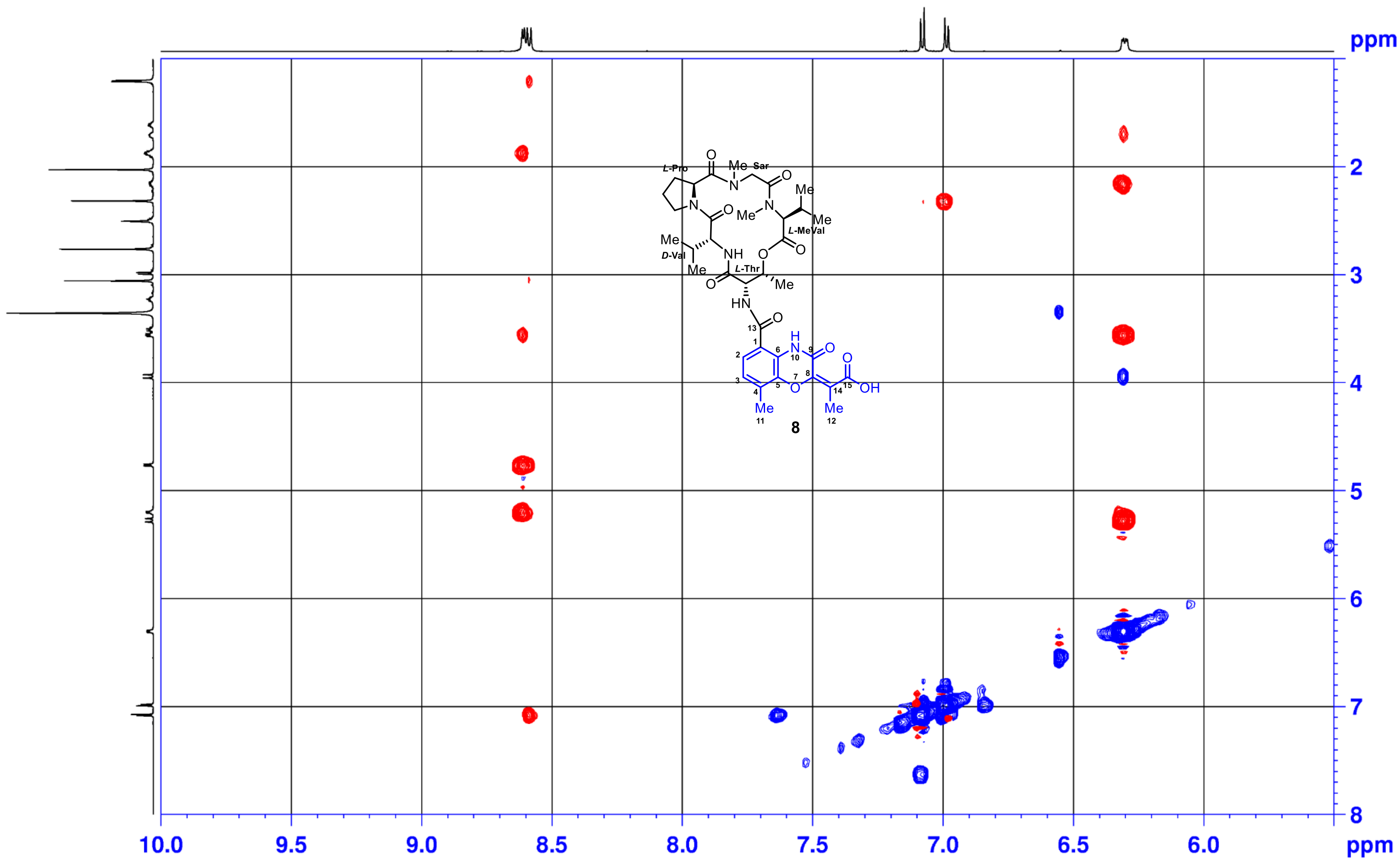

Figure S86. Marfey's analysis of compound **8** and standard amino acids

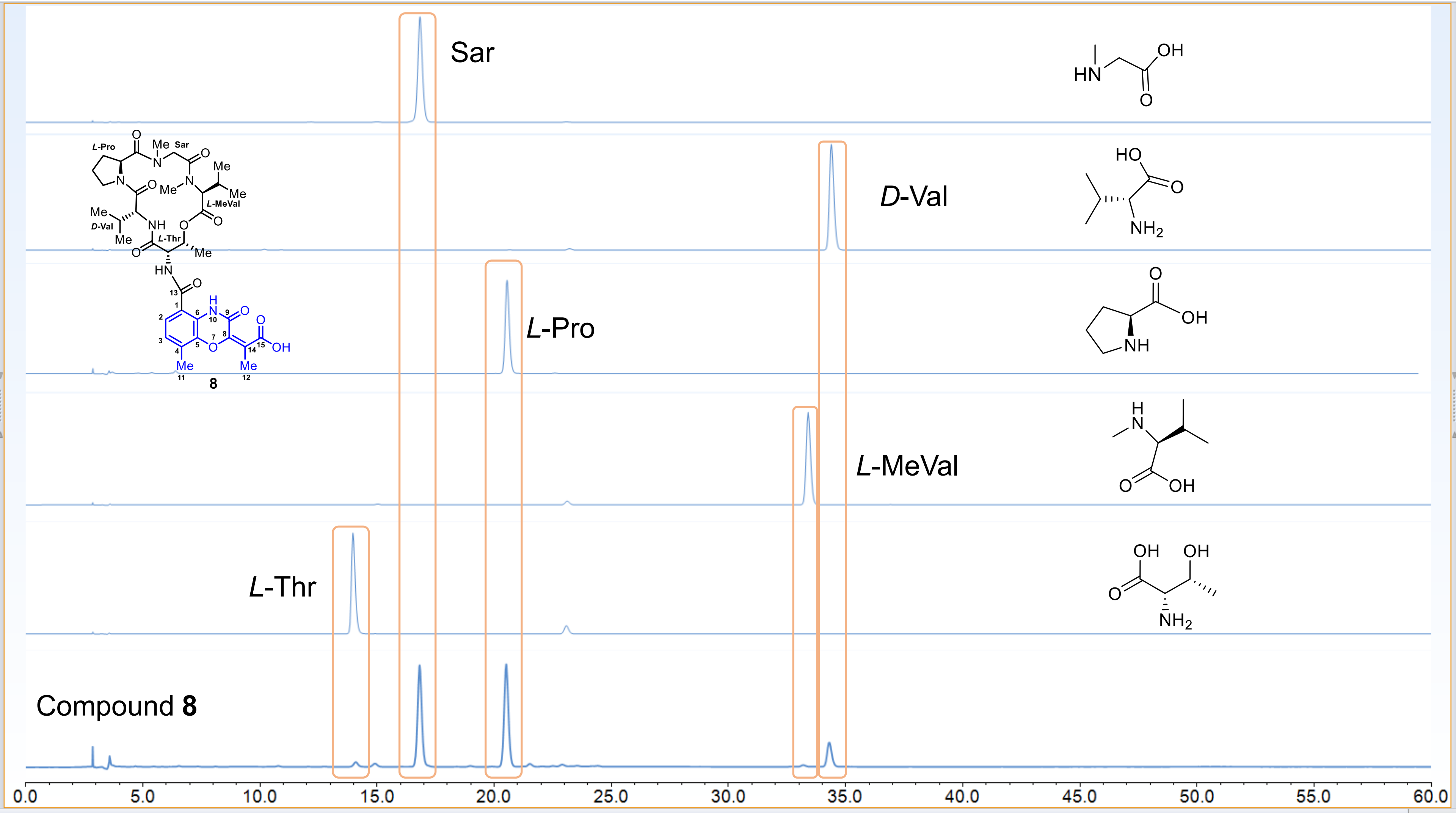

Figure S87. UV spectrum of compound **9**

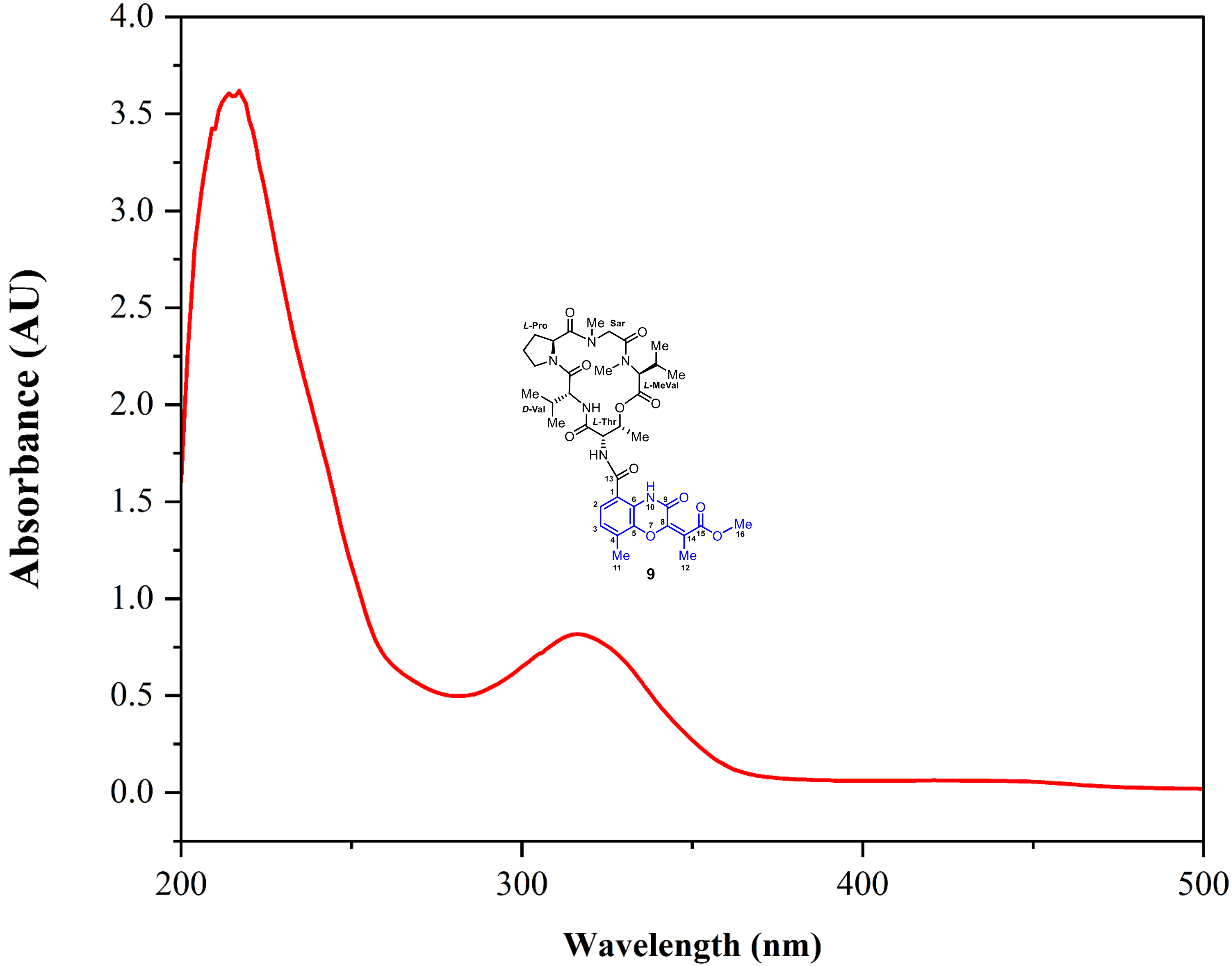

Figure S88. HR-ESIMS for compound **9**

Mass Spectrum SmartFormula Report

Analysis Info

Analysis Name	D:\Data\MS\data\202505\panghe_S-18-1_pos_42_01_19981.d	Acquisition Date	5/29/2025 5:51:03 PM	
Method	LC_Direct Infusion_pos_100-1000mz.m	Operator	SCSIO	
Sample Name	panghe_S-18-1_pos	Instrument	maXis	255552.00029
Comment				

Acquisition Parameter

Source Type	ESI	Ion Polarity	Positive	Set Nebulizer	0.4 Bar
Focus	Active	Set Capillary	4500 V	Set Dry Heater	180 °C
Scan Begin	100 m/z	Set End Plate Offset	-500 V	Set Dry Gas	4.0 l/min
Scan End	2000 m/z	Set Charging Voltage	0 V	Set Divert Valve	Waste
		Set Corona	0 nA	Set APCI Heater	0 °C

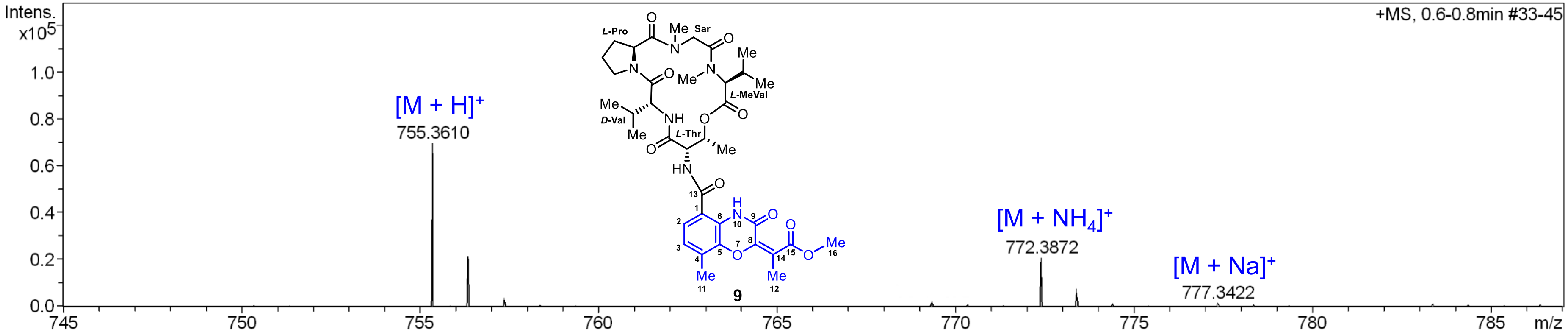

Meas. m/z	#	Ion Formula	Score	m/z	err [ppm]	err [mDa]	mSigma	rdb	e⁻ Conf	N-Rule
755.3610	1	C37H51N6O11	100.00	755.3610	-0.1	-0.0	64.7	15.5	even	ok
772.3872	1	C37H54N7O11	100.00	772.3876	-0.5	-0.4	80.9	14.5	even	ok
777.3422	1	C37H50N6NaO11	100.00	777.3430	1.0	0.8	20.6	15.5	even	ok

Figure S89. ^{1}H NMR (600 MHz) spectrum of compound **9** in DMSO-d_6

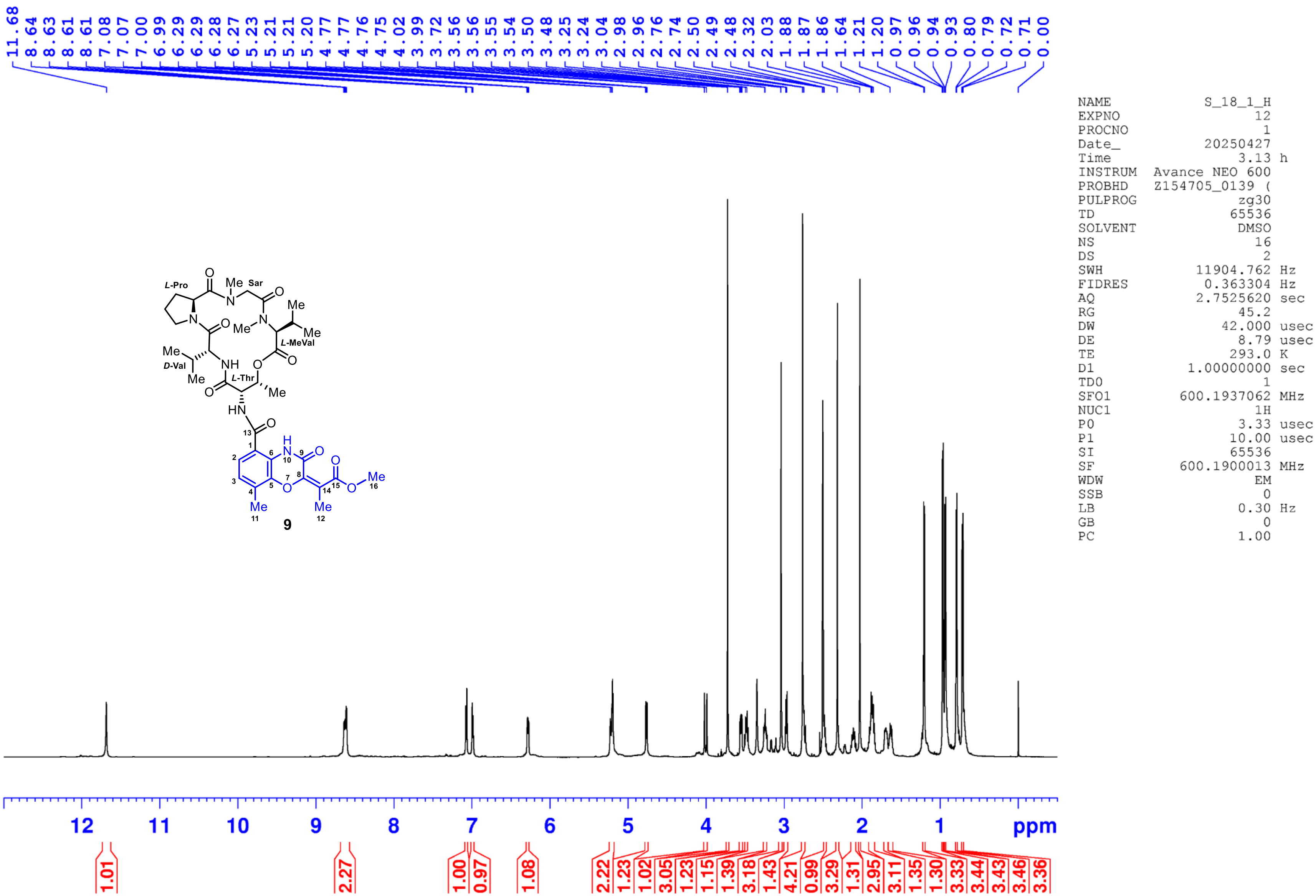

Figure S89-continue

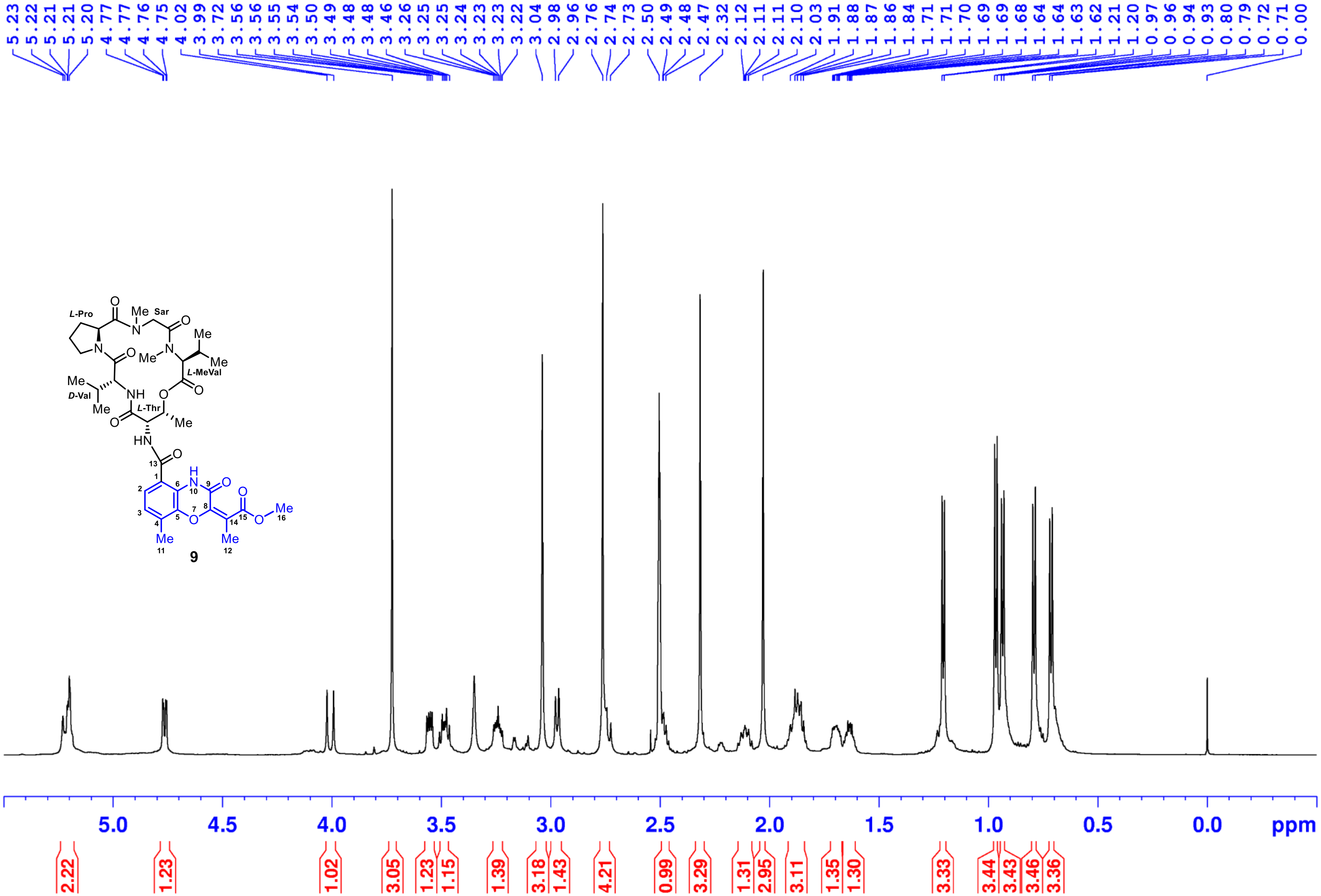

Figure S89-continue

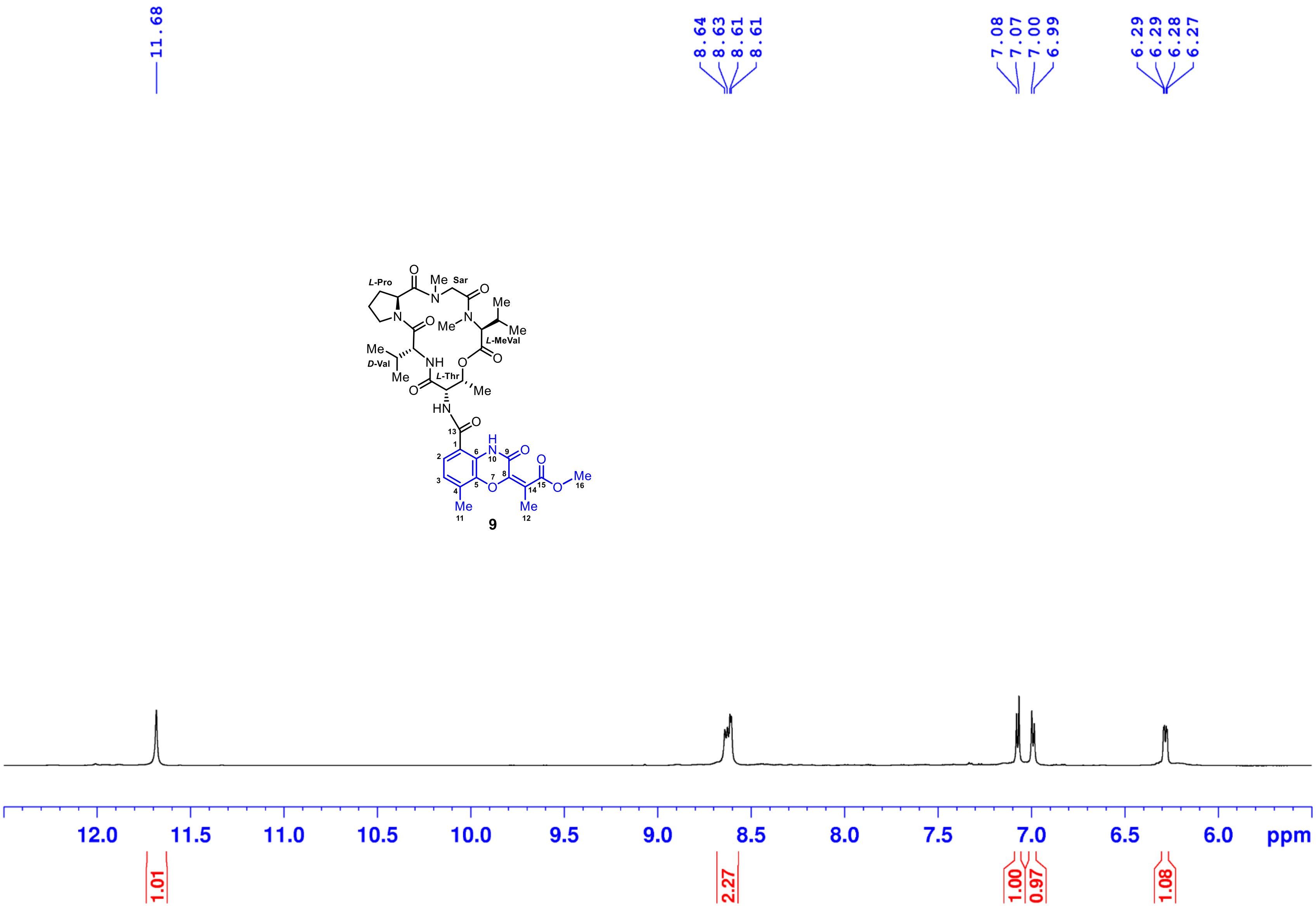

Figure S90. ^{13}C NMR (150 MHz) spectrum of compound **9** in DMSO-d_6

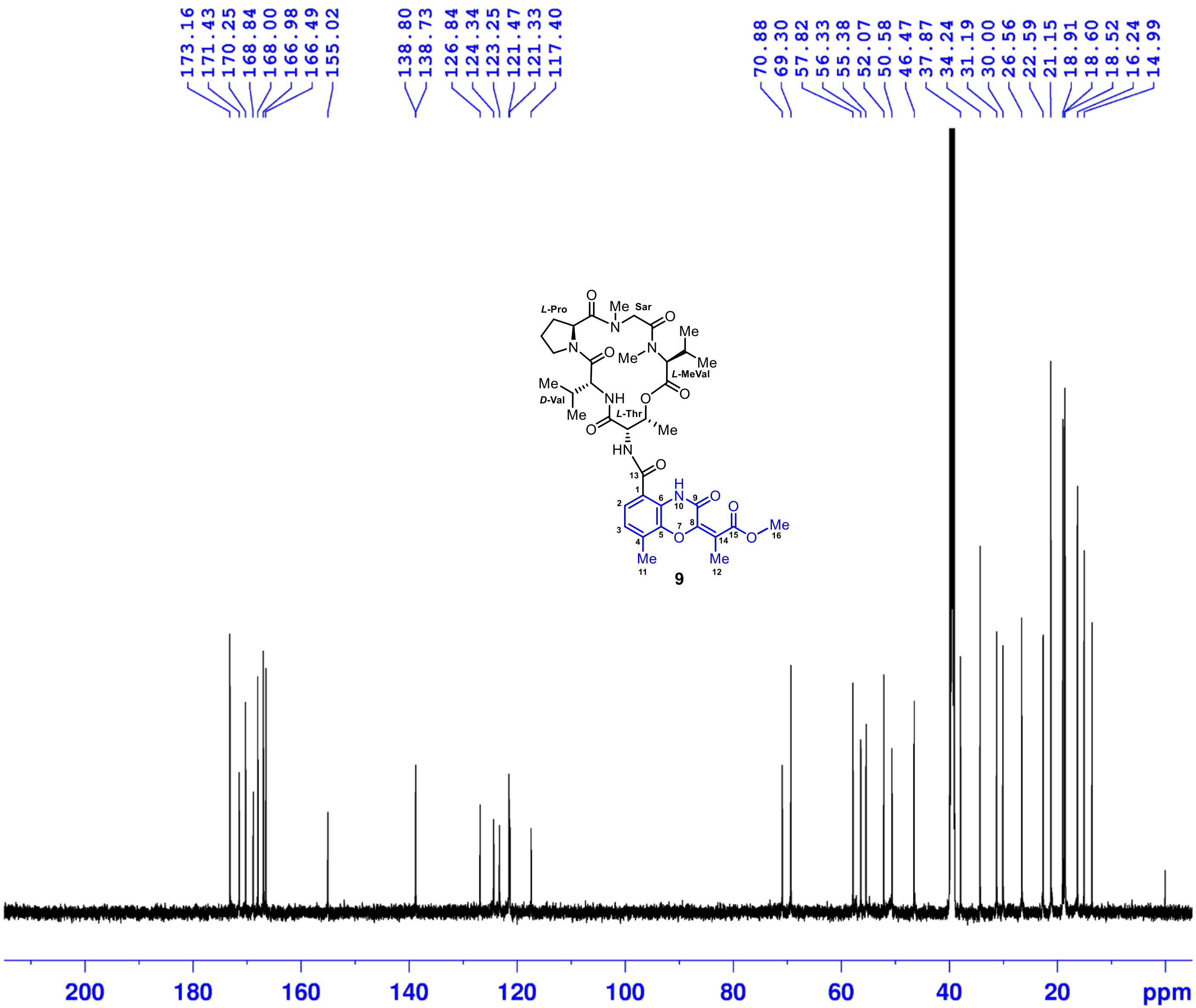

NAME	25411_S_18_1C
EXPNO	144
PROCNO	1
Date_	20250414
Time	12.14 h
INSTRUM	Avance NEO 600
PROBHD	Z154705_0139 (
PULPROG	zgpg30
TD	35714
SOLVENT	DMSO
NS	3000
DS	4
SWH	35714.285 Hz
FIDRES	2.000016 Hz
AQ	0.5000460 sec
RG	101
DW	14.000 usec
DE	6.50 usec
TE	293.8 K
D1	1.00000000 sec
D11	0.03000000 sec
TD0	1
SFO1	150.9329873 MHz
NUC1	13C
P0	4.00 usec
P1	12.00 usec
SI	32768
SF	150.9179807 MHz
WDW	EM
SSB	0
LB	1.00 Hz
GB	0
PC	1.40

Figure S90-continue

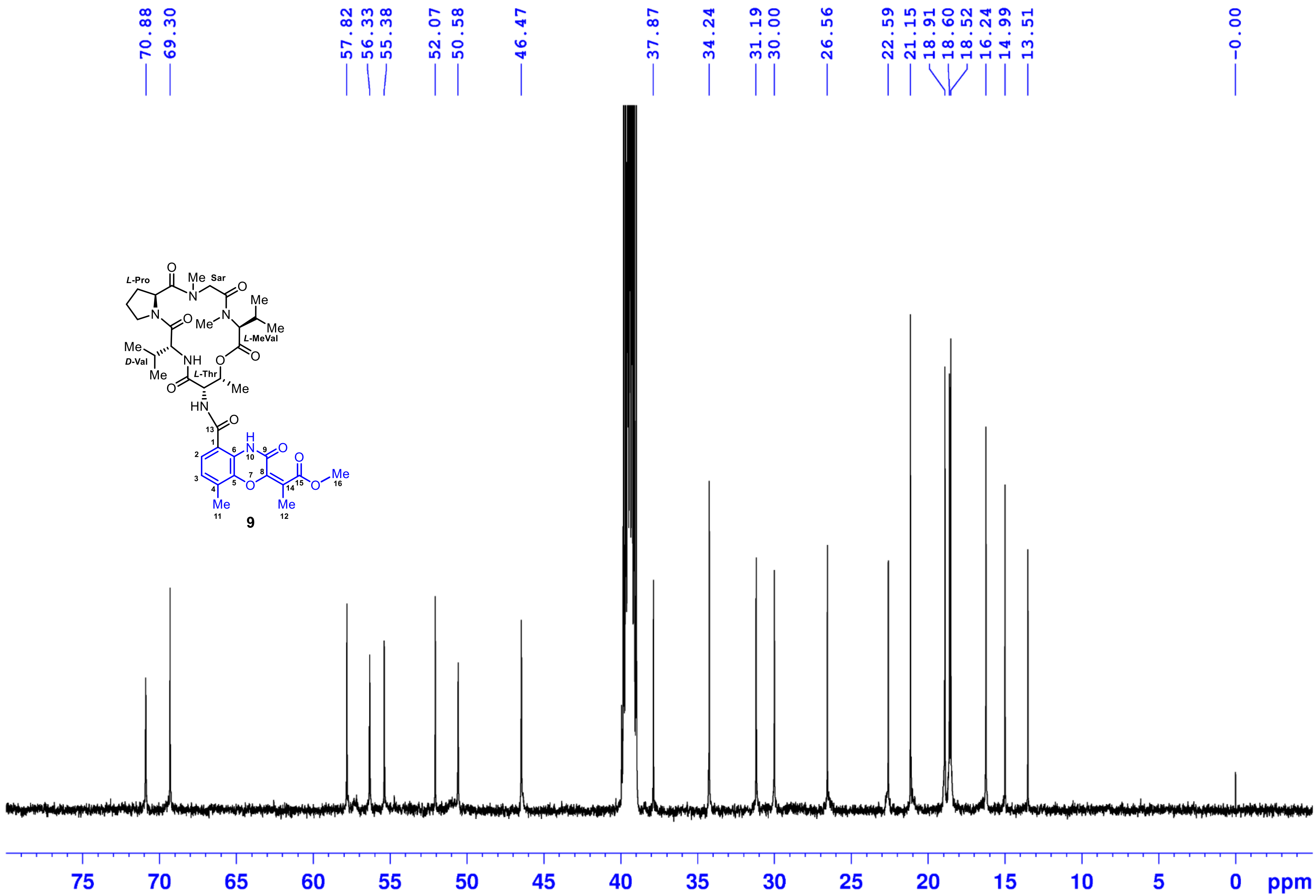

Figure S90-continue

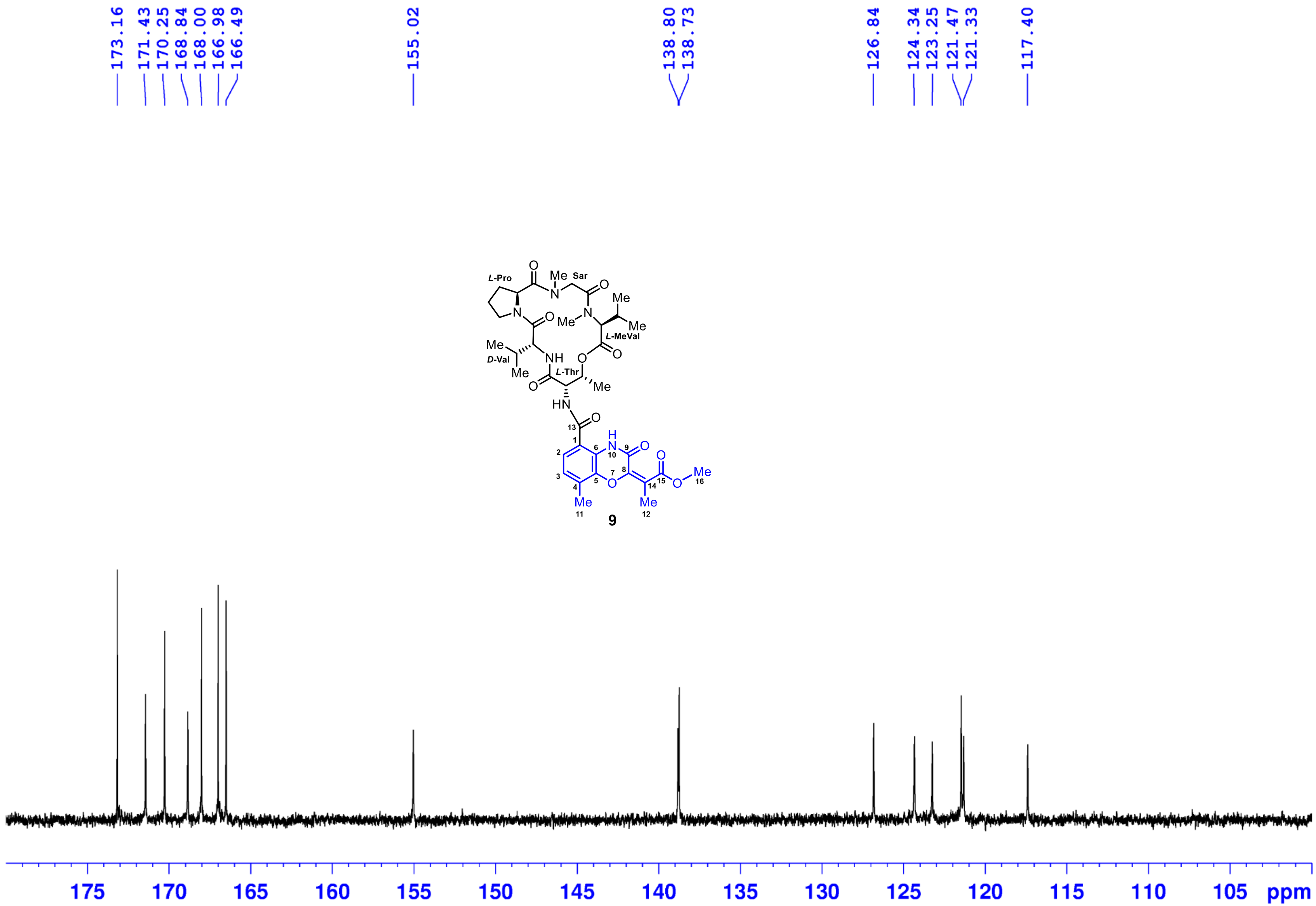

Figure S91. DEPT135 (150 MHz) spectrum of compound **9** in DMSO-d_6

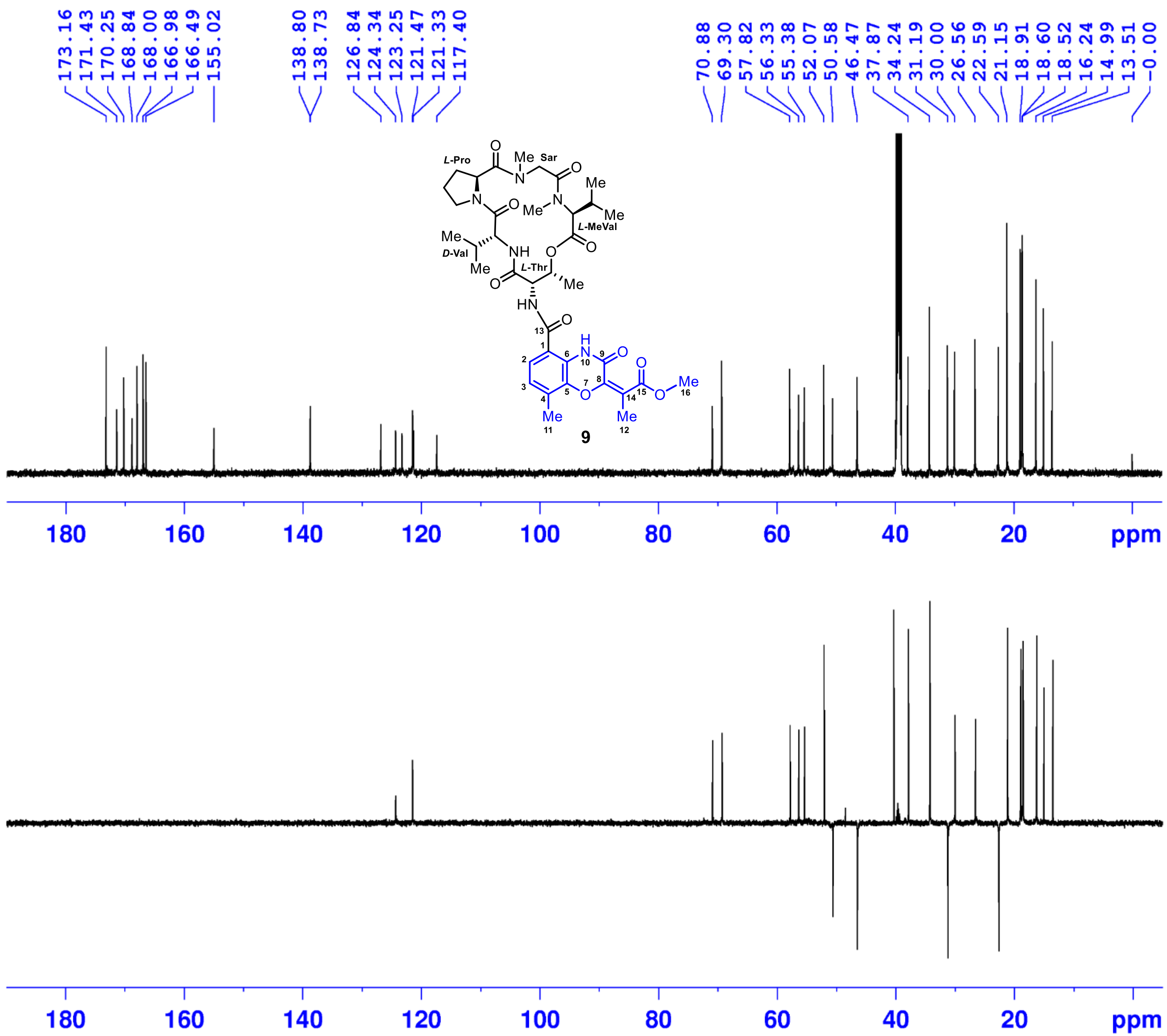

NAME	2531_S18_1D135
EXPNO	157
PROCNO	1
Date_	20250302
Time	11.52 h
INSTRUM	Avance NEO 600
PROBHD	Z154705_0139 (
PULPROG	deptsp135
TD	65536
SOLVENT	DMSO
NS	512
DS	8
SWH	41666.668 Hz
FIDRES	1.271566 Hz
AQ	0.7864820 sec
RG	101
DW	12.000 usec
DE	6.50 usec
TE	294.1 K
CNST2	145.0000000
D1	2.00000000 sec
D2	0.00344828 sec
D12	0.00002000 sec
TD0	1
SFO1	150.9299690 MHz
NUC1	13C
P1	12.00 usec
P13	2000.00 usec
SI	32768
SF	150.9179813 MHz
WDW	EM
SSB	0
LB	1.00 Hz
GB	0
PC	1.40

Figure S91-continue

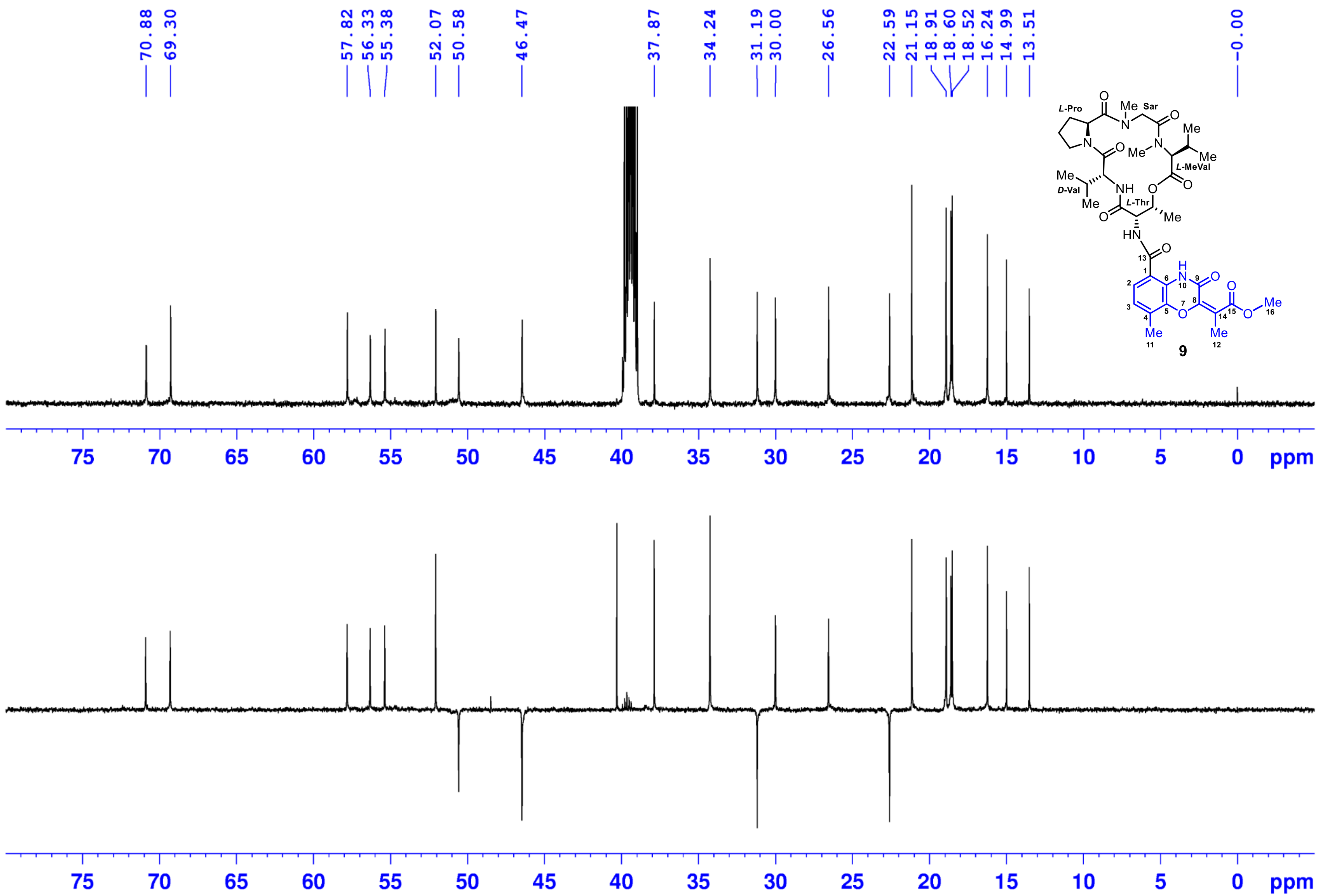

Figure S91-continue

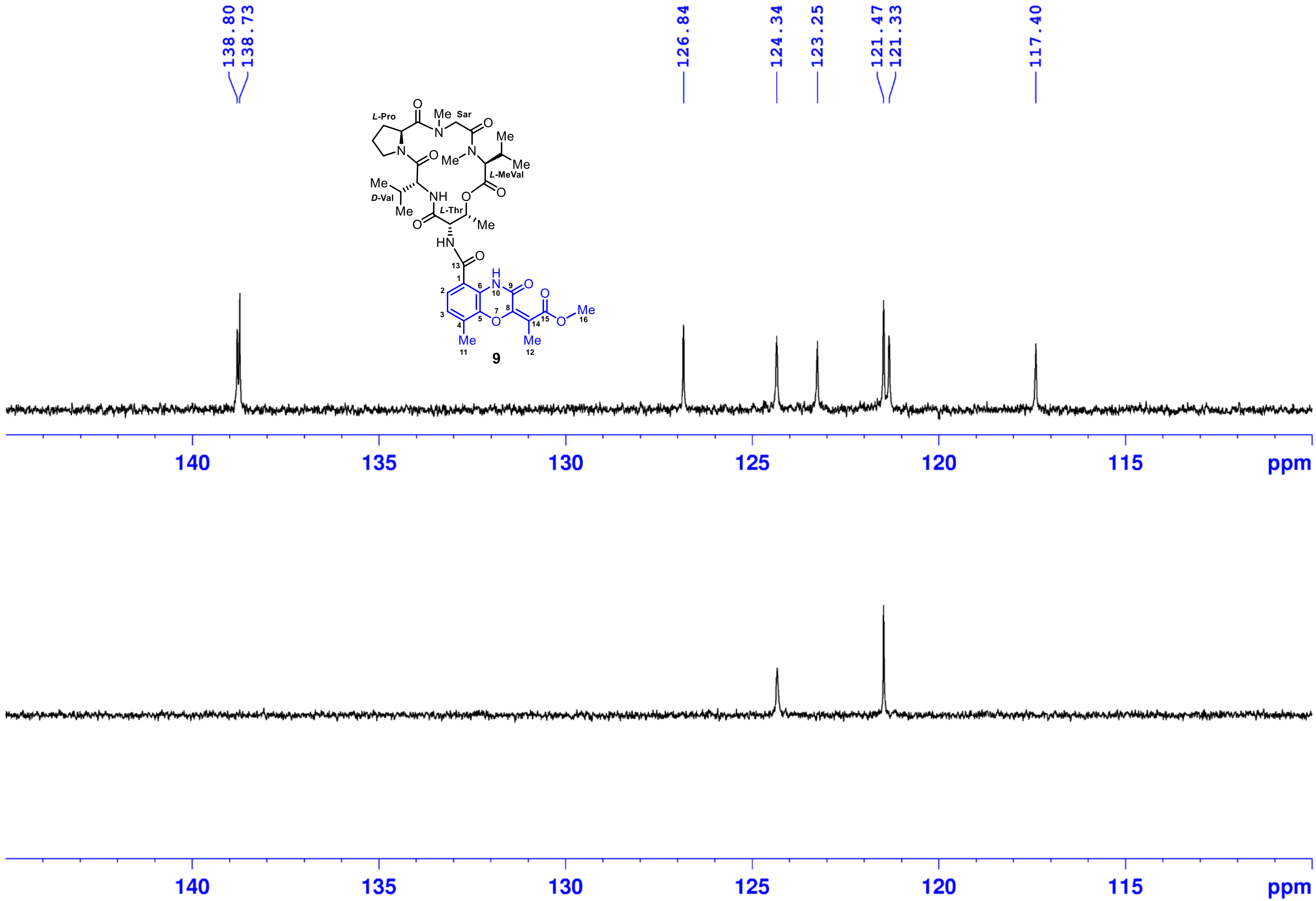

Figure S92. ^{1}H–^{1}H COSY (600 MHz) spectrum of compound **9** in DMSO-d_6

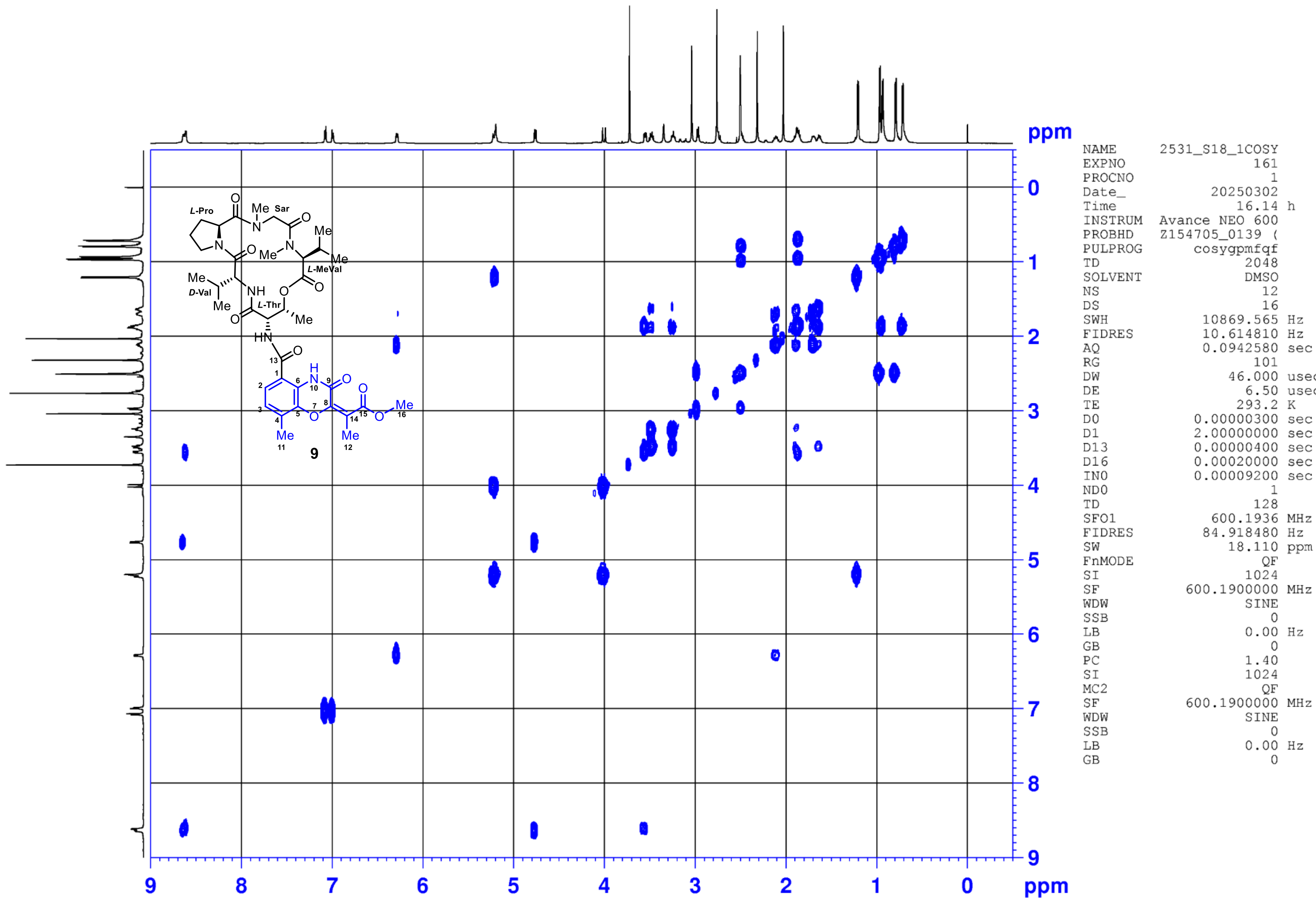

Figure S92-continue

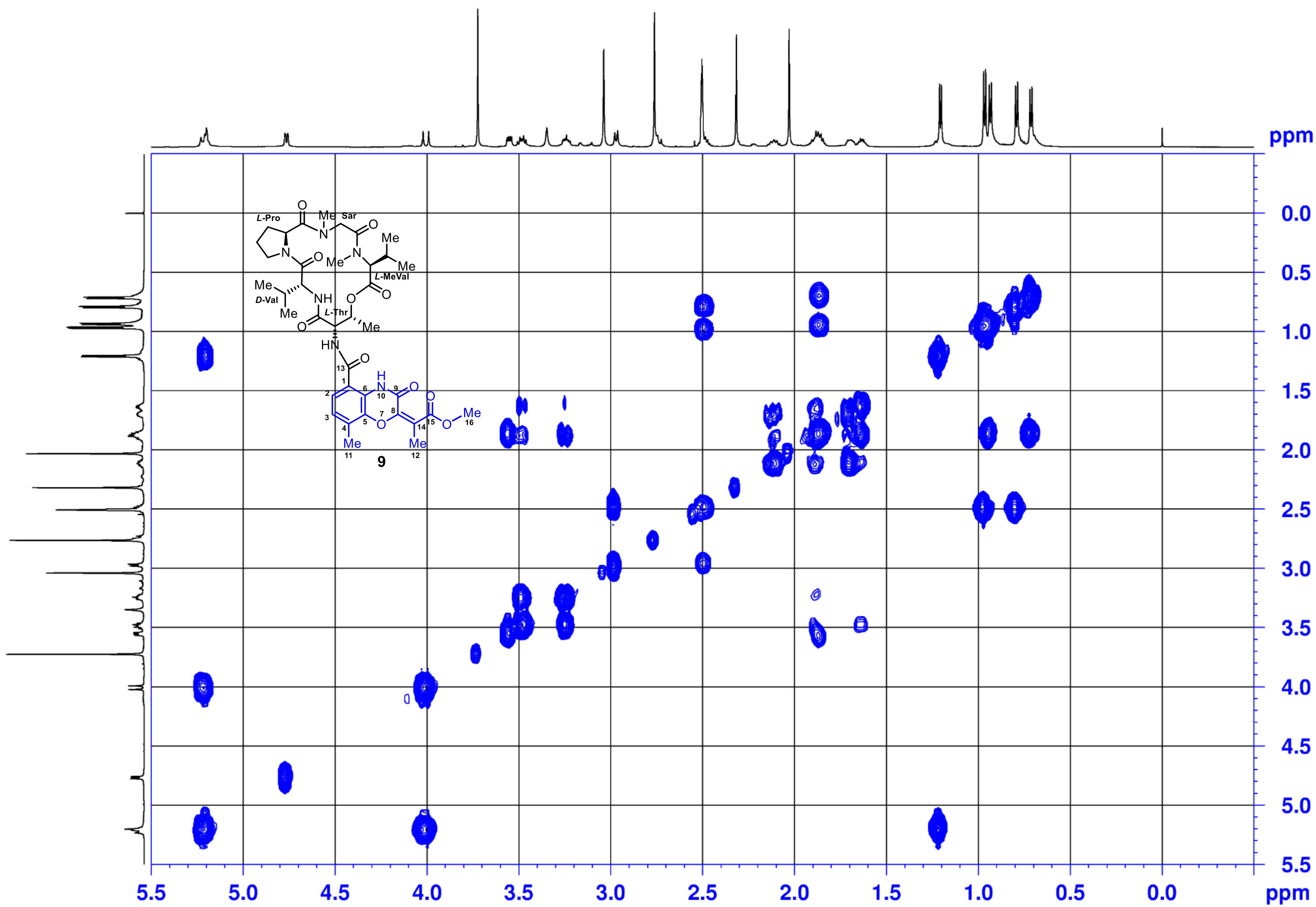

Figure S92-continue

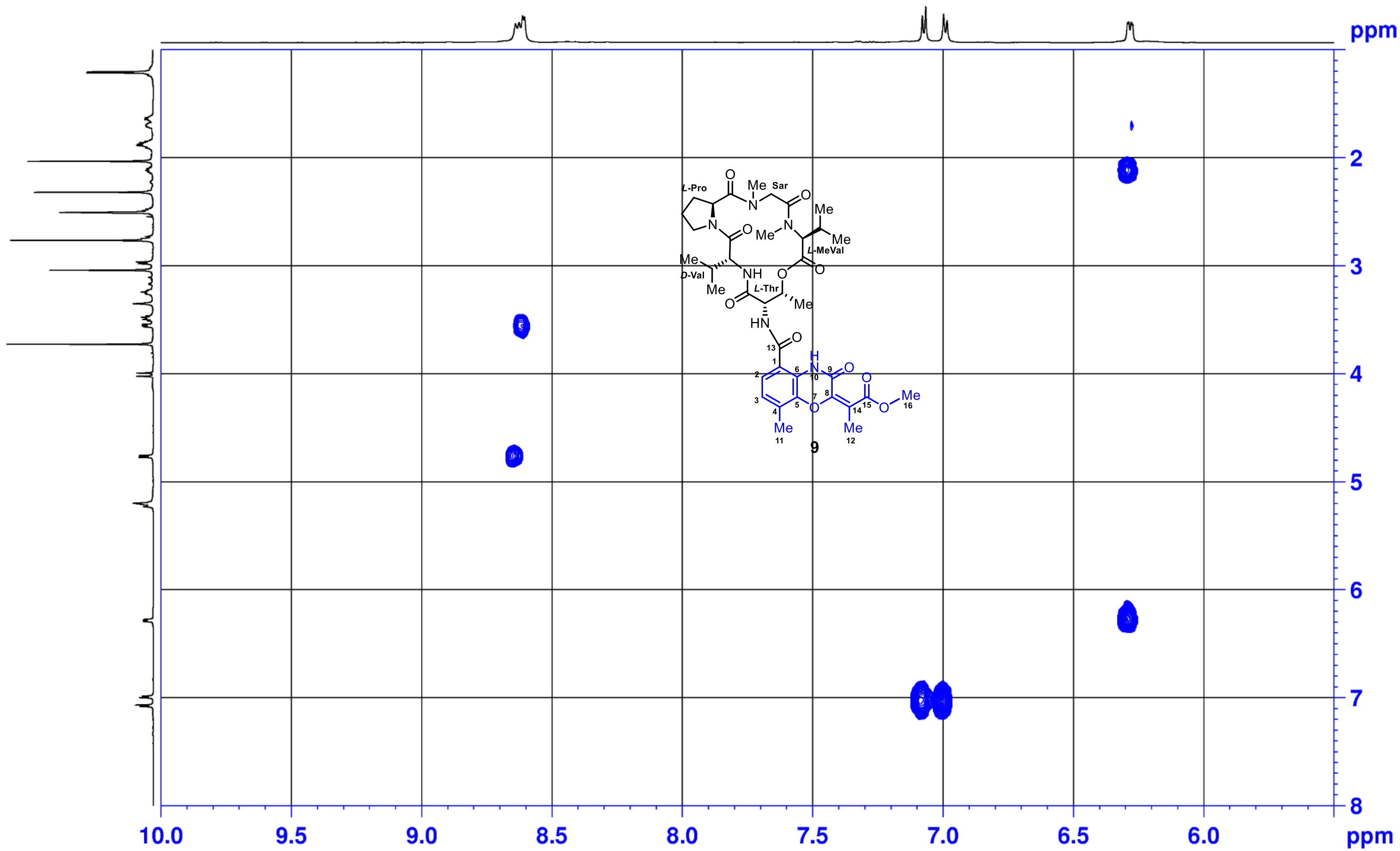

Figure S93. HSQC (600 MHz) spectrum of compound **9** in DMSO-d_6

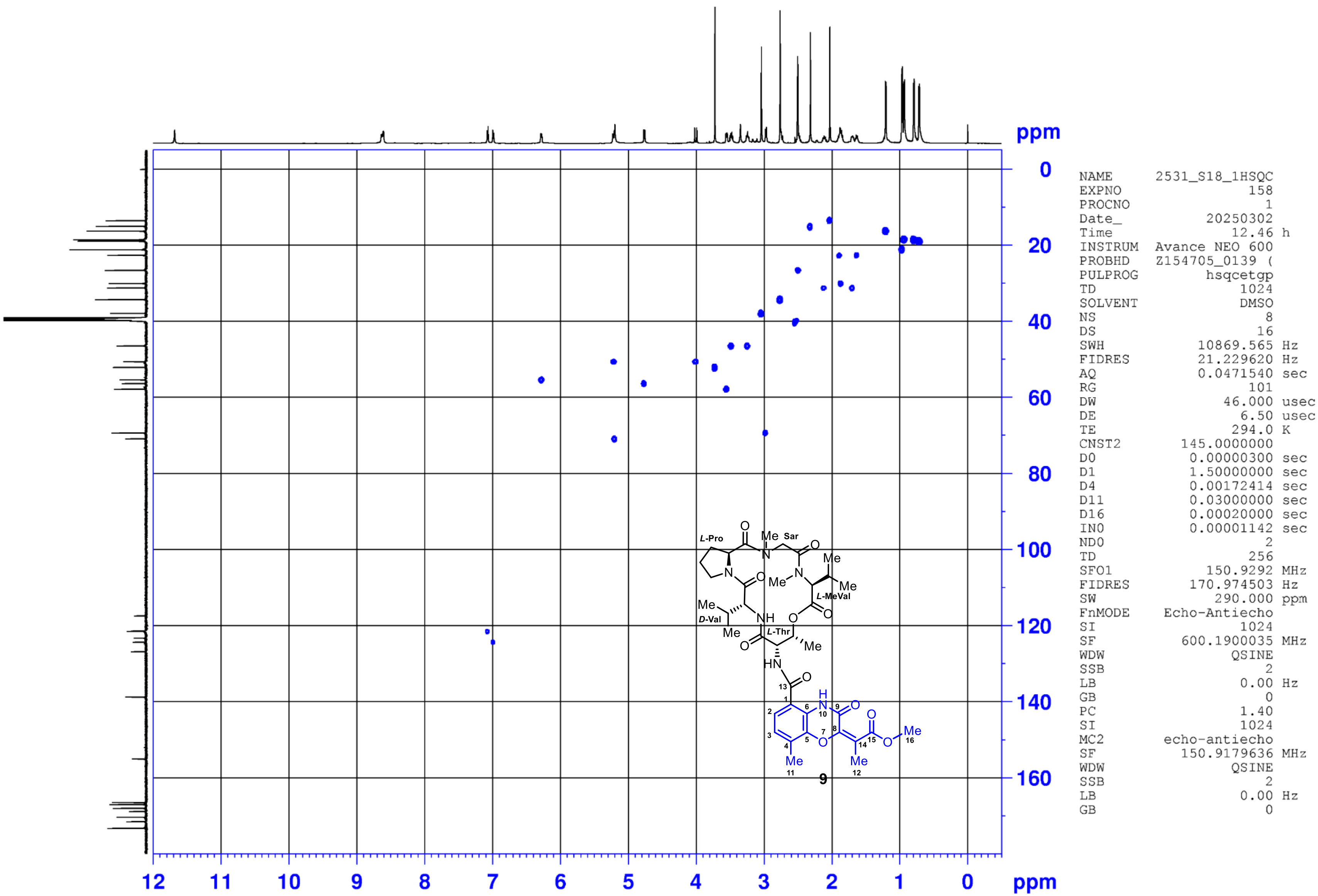

Figure S93-continue

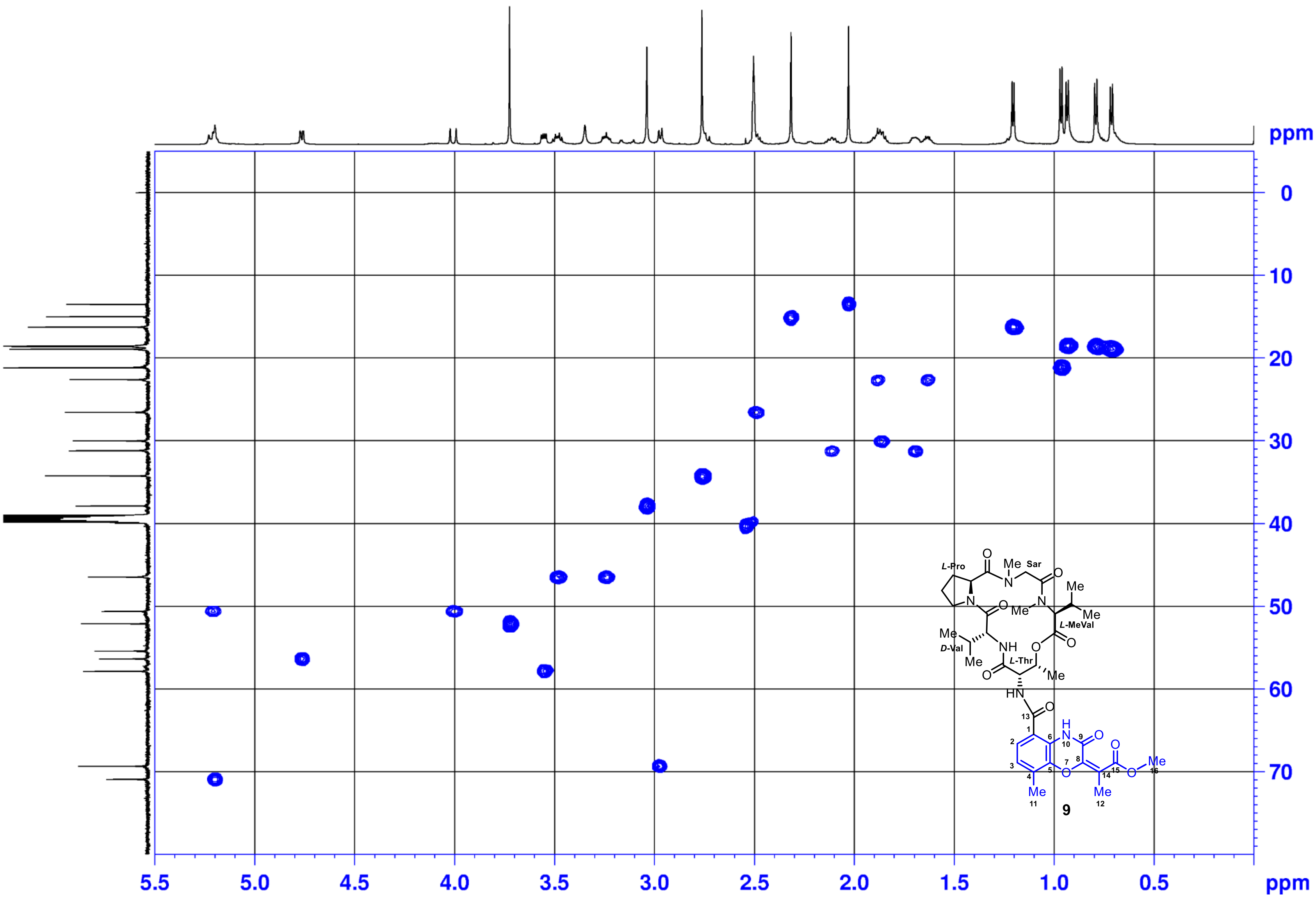

Figure S93-continue

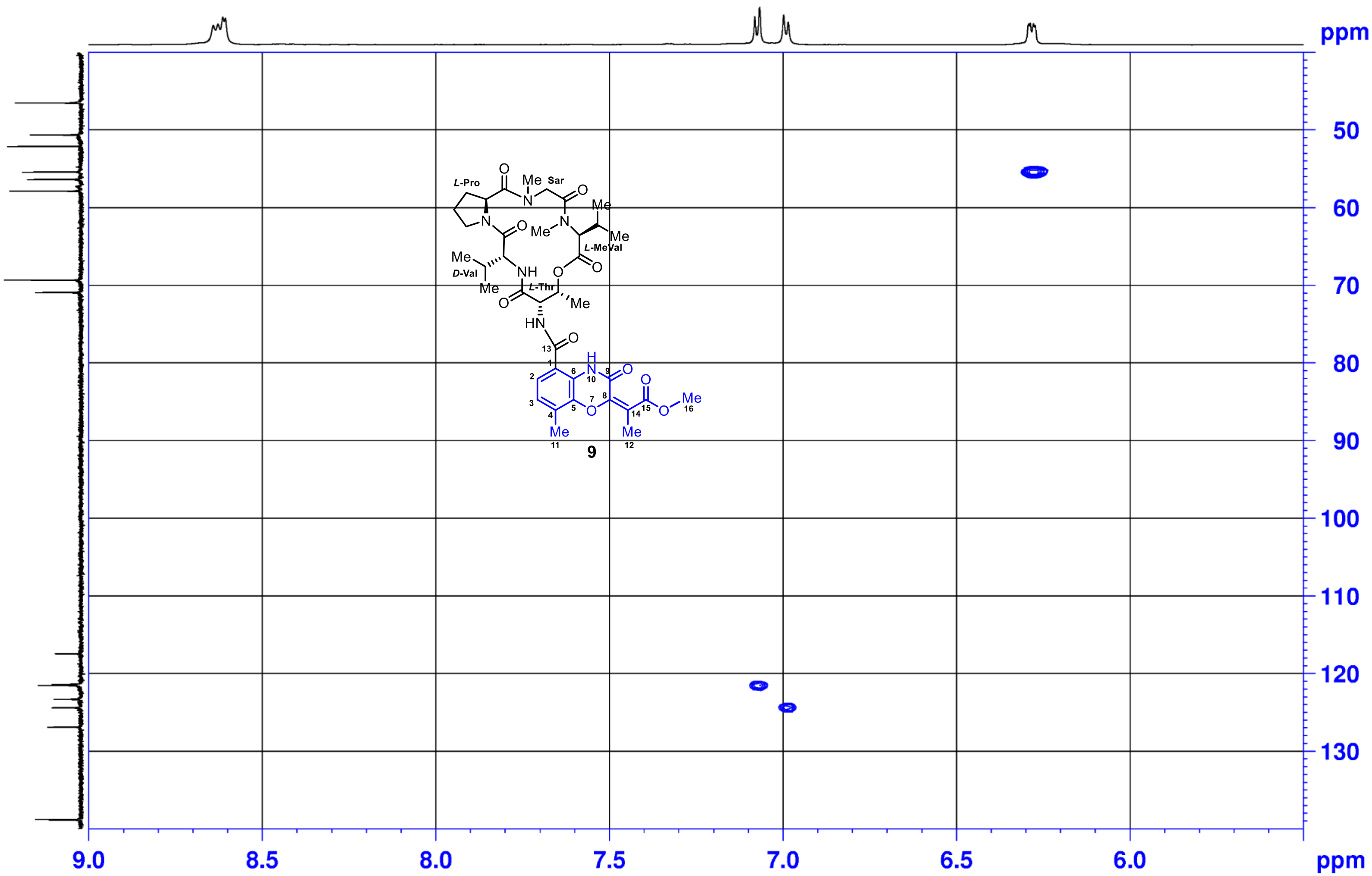

Figure S94. HMBC (600 MHz) spectrum of compound **9** in DMSO-d_6

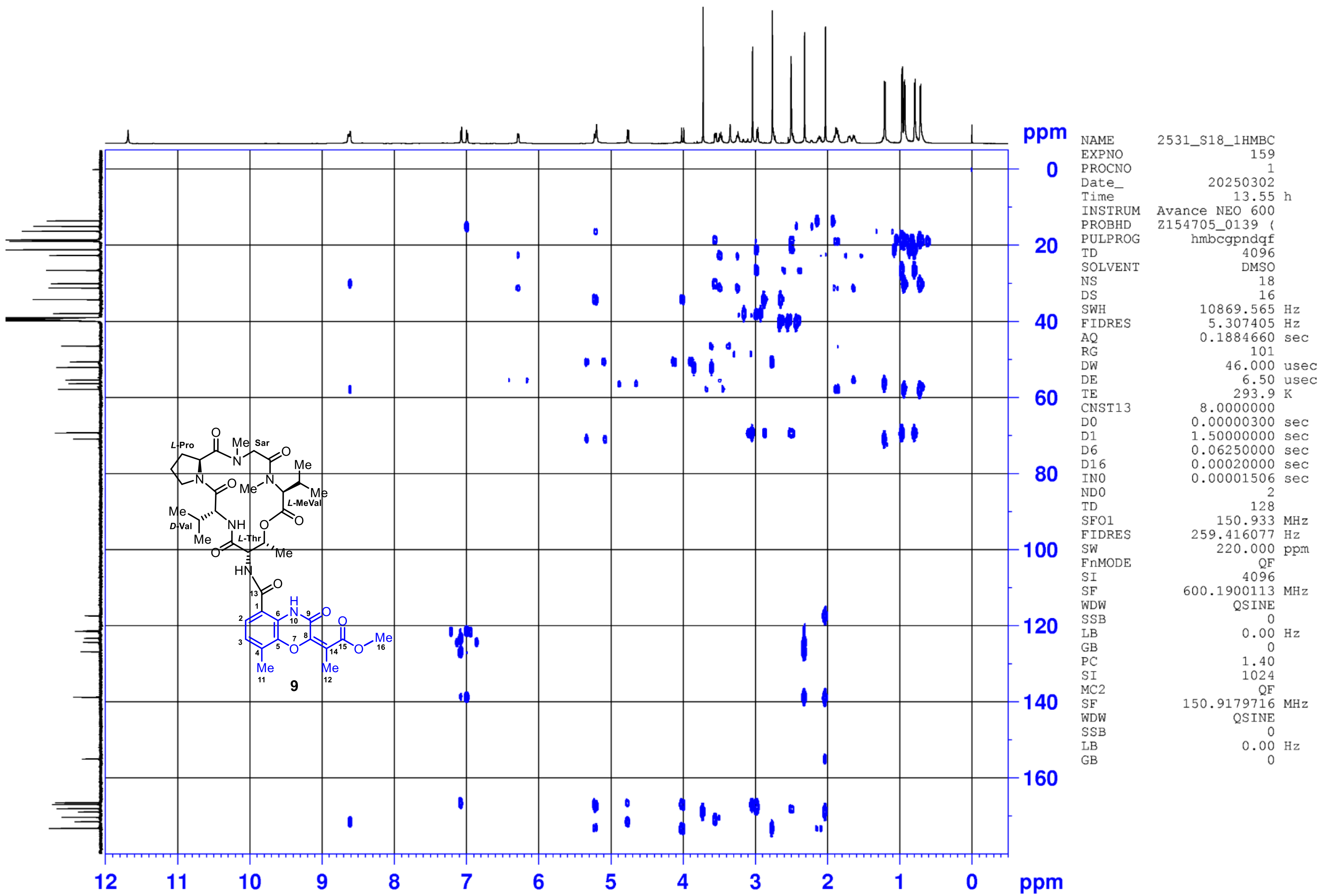

Figure S94-continue

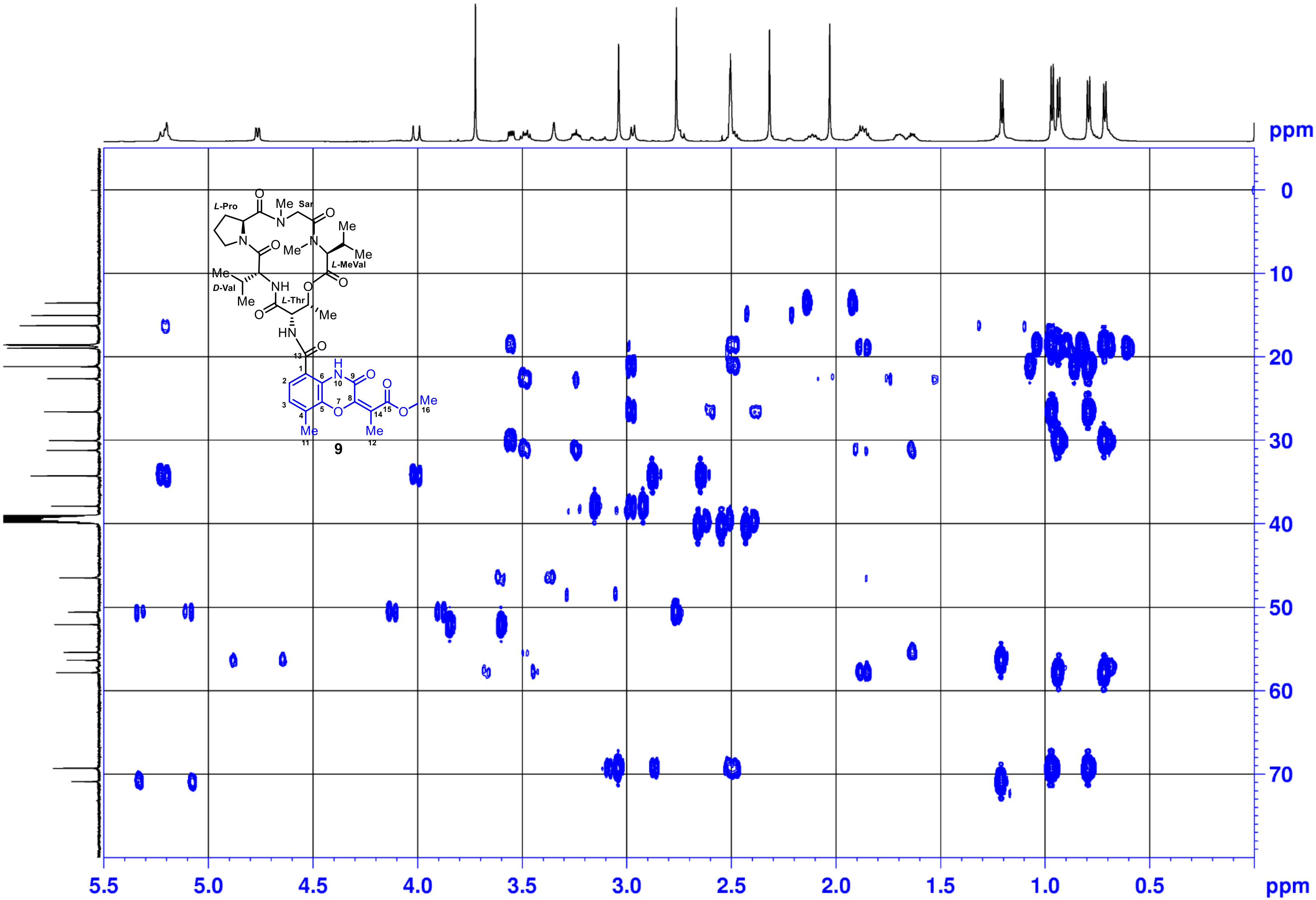

Figure S94-continue

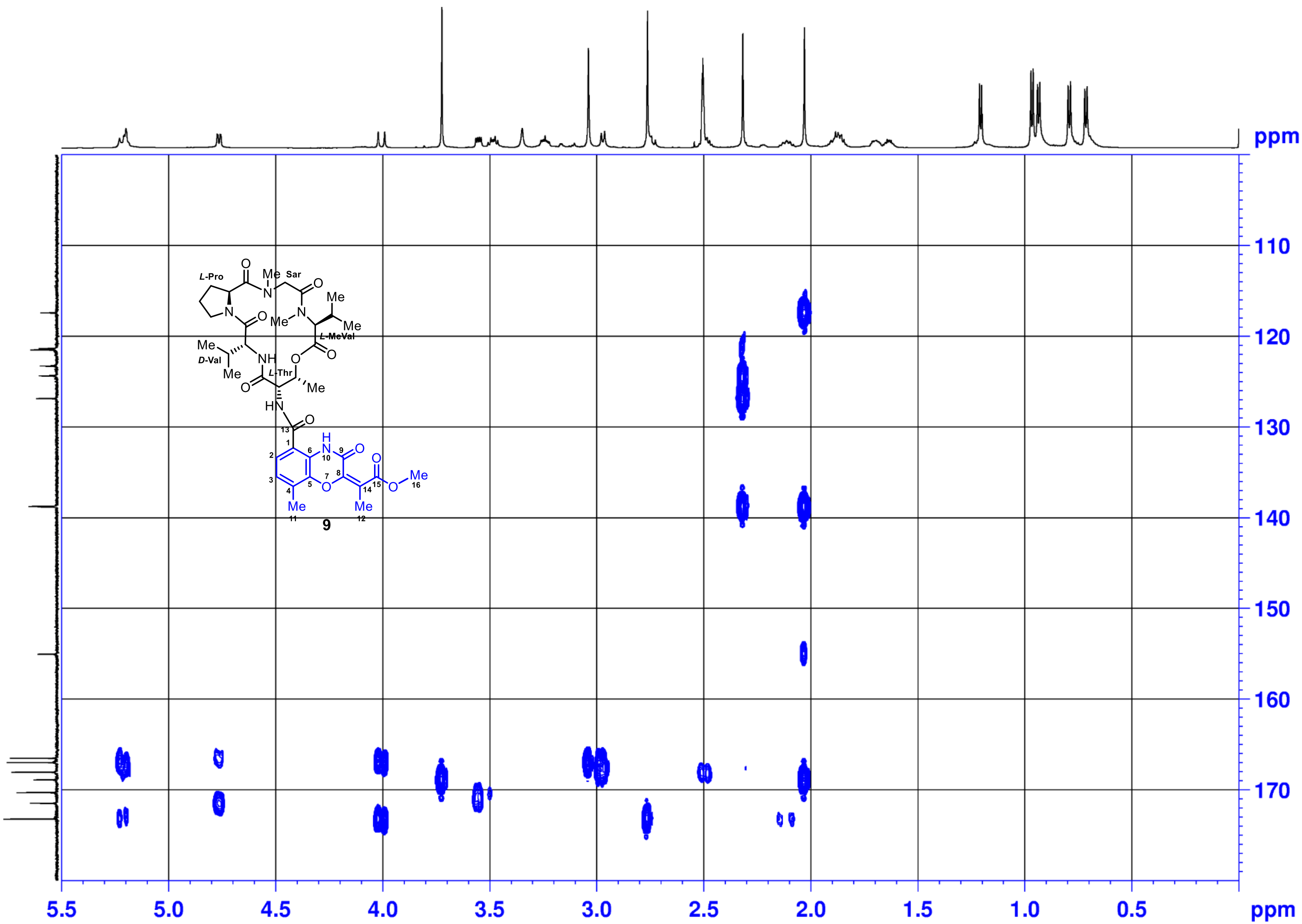

Figure S94-continue

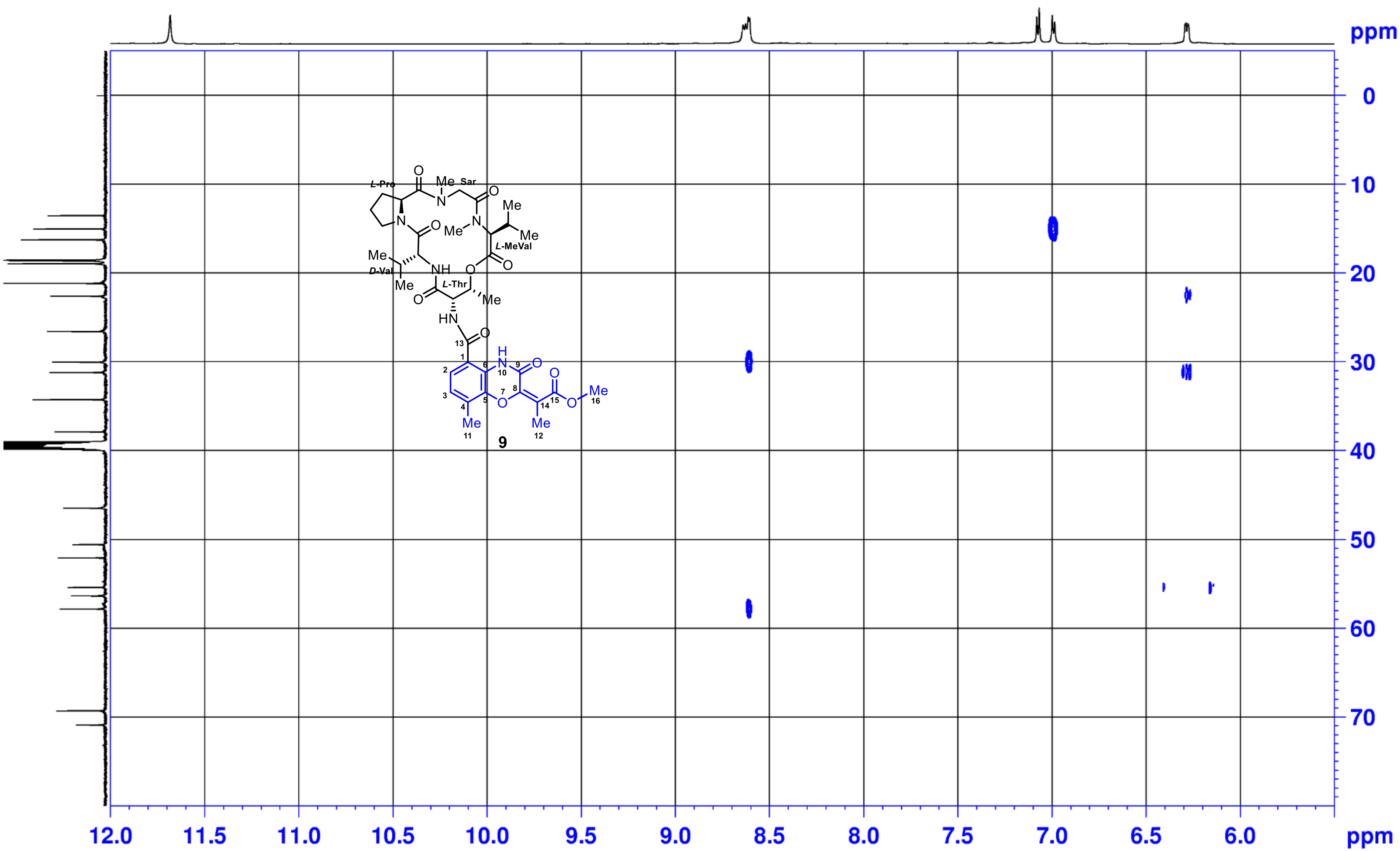

Figure S94-continue

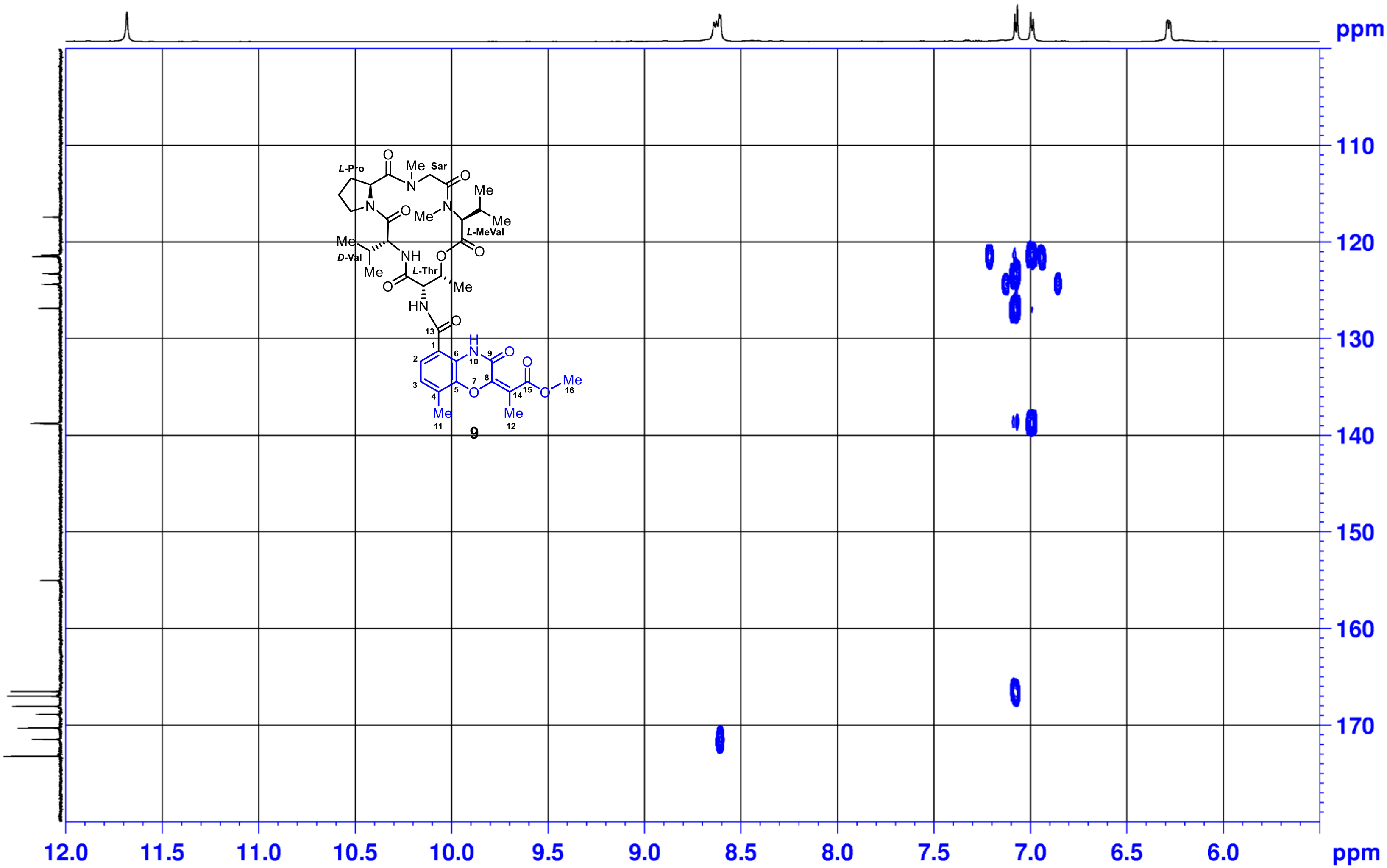

Figure S95. ROESY (600 MHz) spectrum of compound **9** in DMSO-d_6

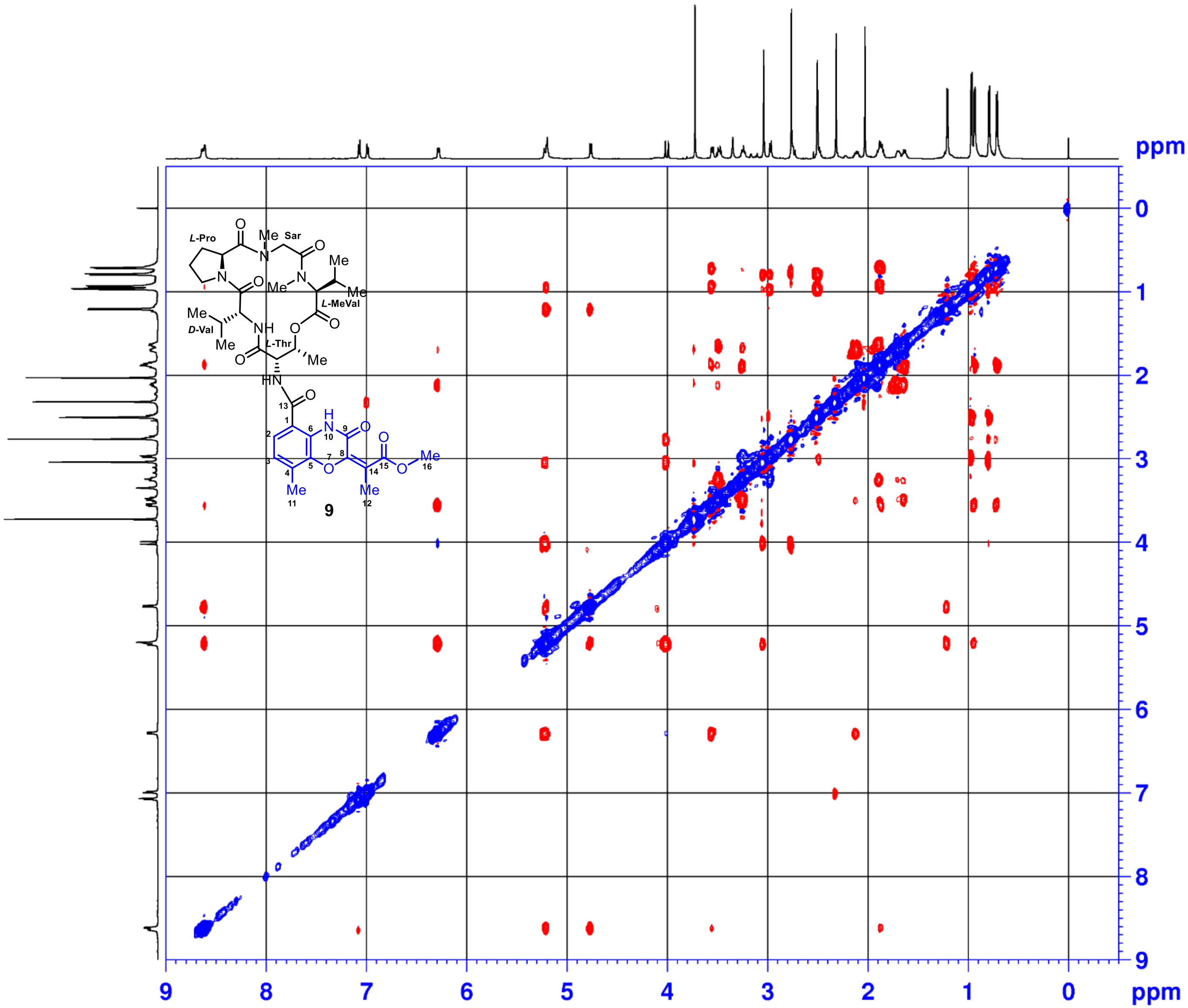

NAME	S_18_1_ROE_1
EXPNO	10
PROCNO	1
Date_	20250427
Time	3.11 h
INSTRUM	Avance NEO 600
PROBHD	Z154705_0139 (
PULPROG	roesyphpp.2
TD	2048
SOLVENT	DMSO
NS	12
DS	32
SWH	13888.889 Hz
FIDRES	13.563368 Hz
AQ	0.0737780 sec
RG	101
DW	36.000 usec
DE	6.50 usec
TE	292.9 K
D0	0.00002650 sec
D1	2.00000000 sec
D11	0.03000000 sec
D12	0.00002000 sec
IN0	0.00007373 sec
L4	556
P15	200000.00 usec
ND0	1
TD	256
SFO1	600.1928 MHz
FIDRES	52.985771 Hz
SW	22.600 ppm
FnMODE	States-TPPI
SI	1024
SF	600.1899929 MHz
WDW	QSINE
SSB	2
LB	0.00 Hz
GB	0
PC	1.00
SI	1024
MC2	States-TPPI
SF	600.1899931 MHz
WDW	QSINE
SSB	2
LB	0.00 Hz
GB	0

Figure S95-continue

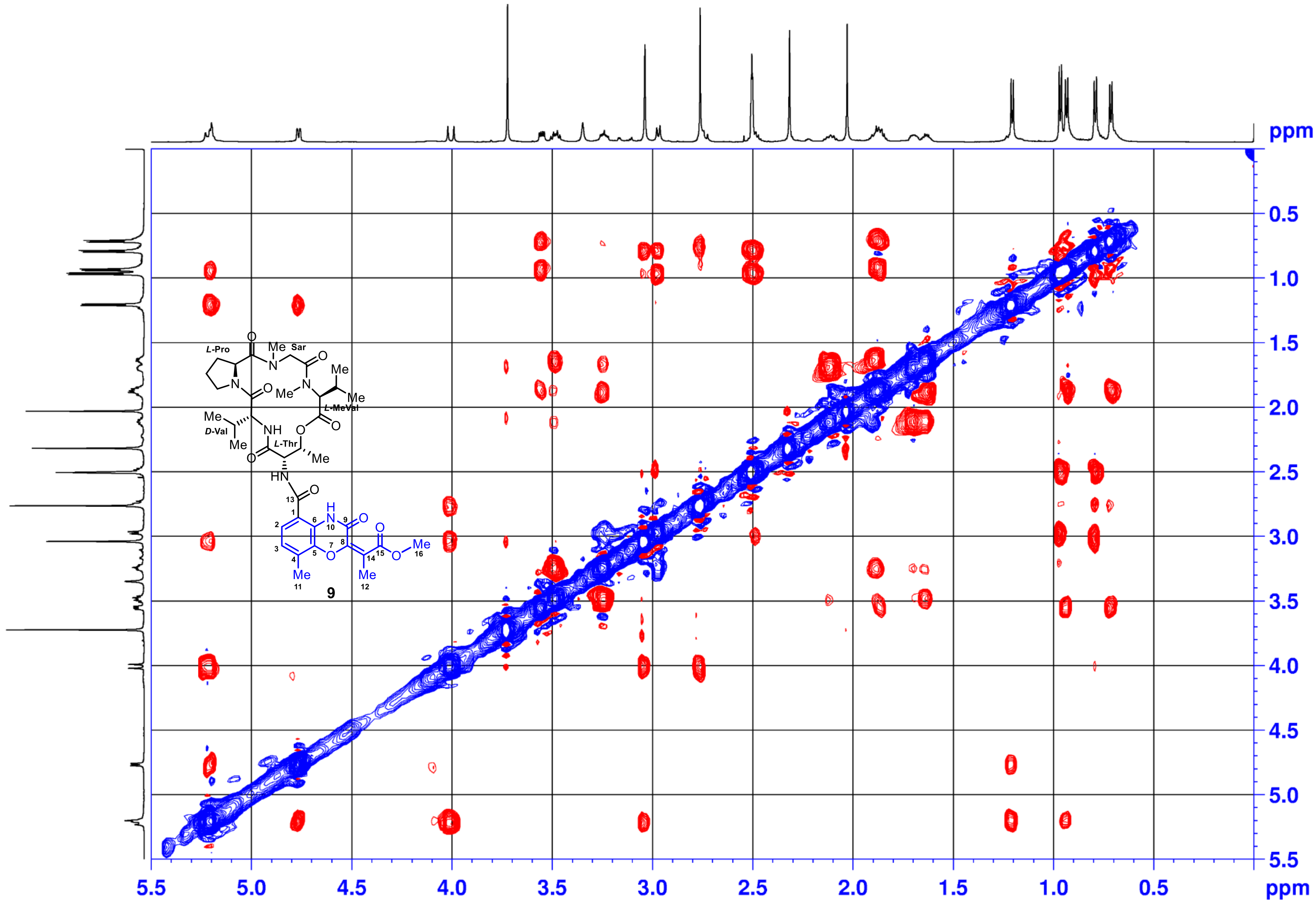

Figure S95-continue

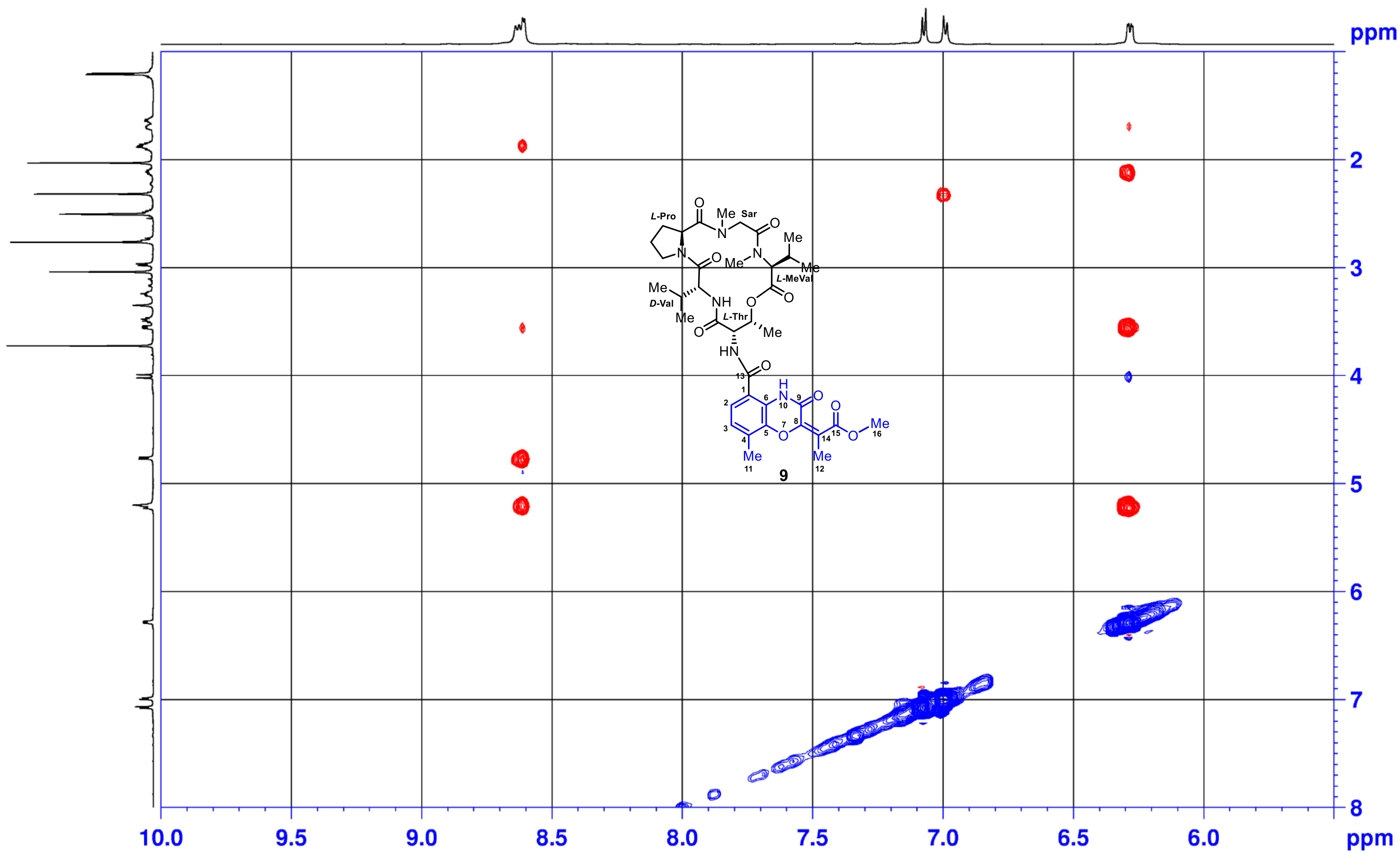

Figure S96. Marfey's analysis of compound **9** and standard amino acids

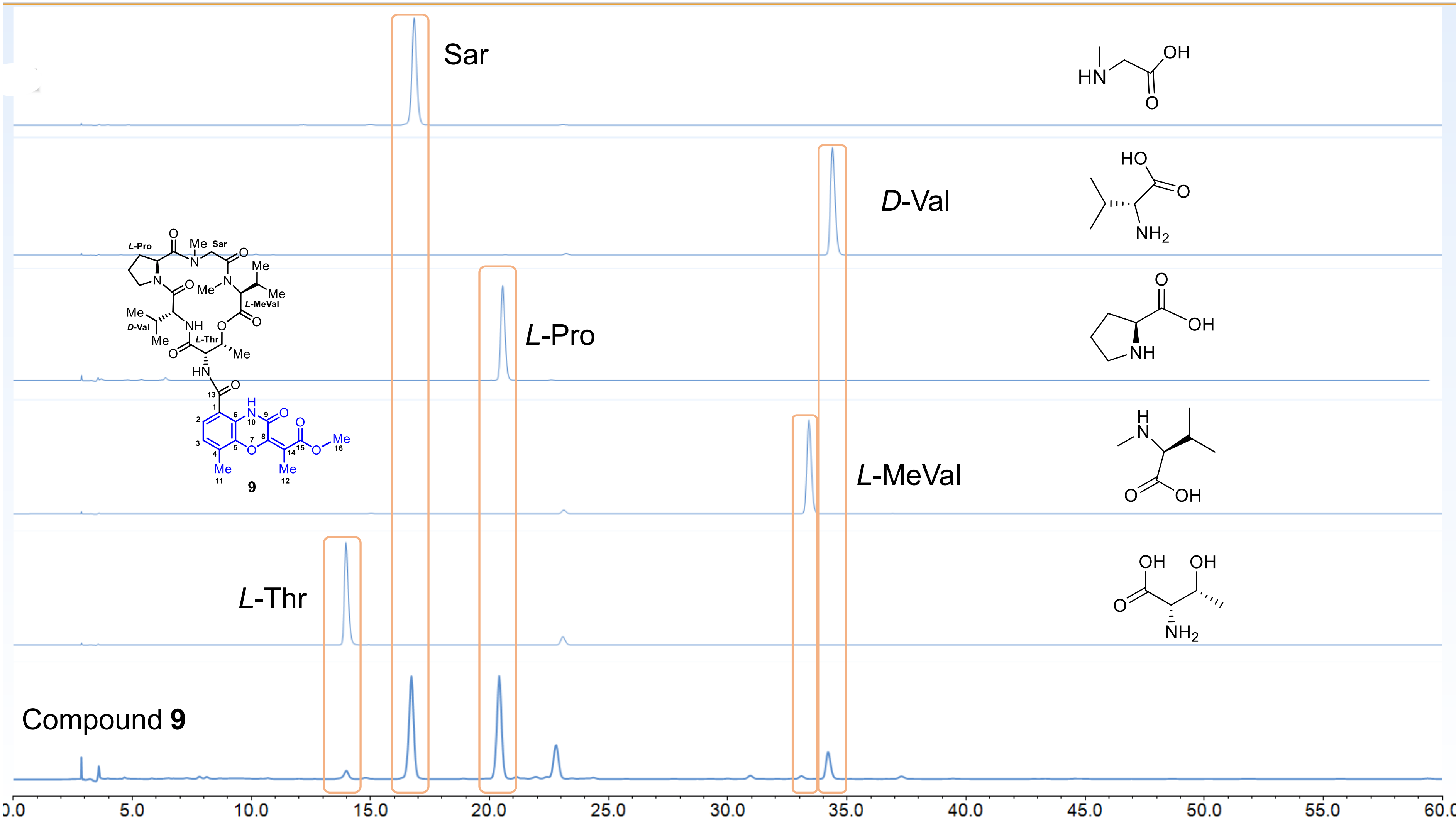

Figure S97. UV spectrum of compound **10**

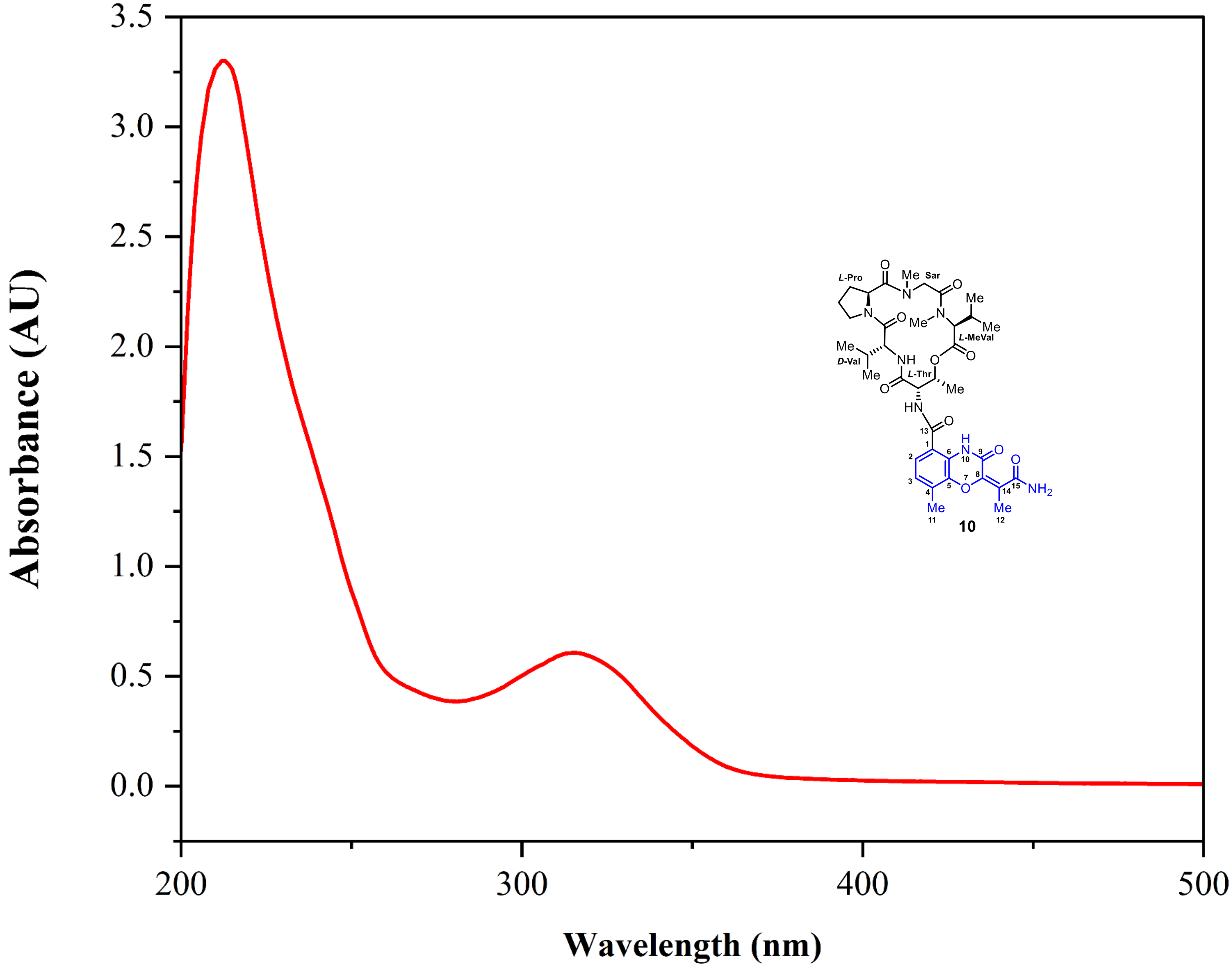

Figure S98. HR-ESIMS for compound **10**

Mass Spectrum SmartFormula Report

Analysis Info

Analysis Name	D:\Data\MS\data\202505\panghe_S-4-0_pos_44_01_19983.d	Acquisition Date	5/29/2025 5:58:22 PM
Method	LC_Direct Infusion_pos_100-1000mz.m	Operator	SCSIO
Sample Name	panghe_S-4-0_pos	Instrument	maXis 255552.00029
Comment			

Acquisition Parameter

Source Type	ESI	Ion Polarity	Positive	Set Nebulizer	0.4 Bar
Focus	Active	Set Capillary	4500 V	Set Dry Heater	180 °C
Scan Begin	100 m/z	Set End Plate Offset	-500 V	Set Dry Gas	4.0 l/min
Scan End	2000 m/z	Set Charging Voltage	0 V	Set Divert Valve	Waste
		Set Corona	0 nA	Set APCI Heater	0 °C

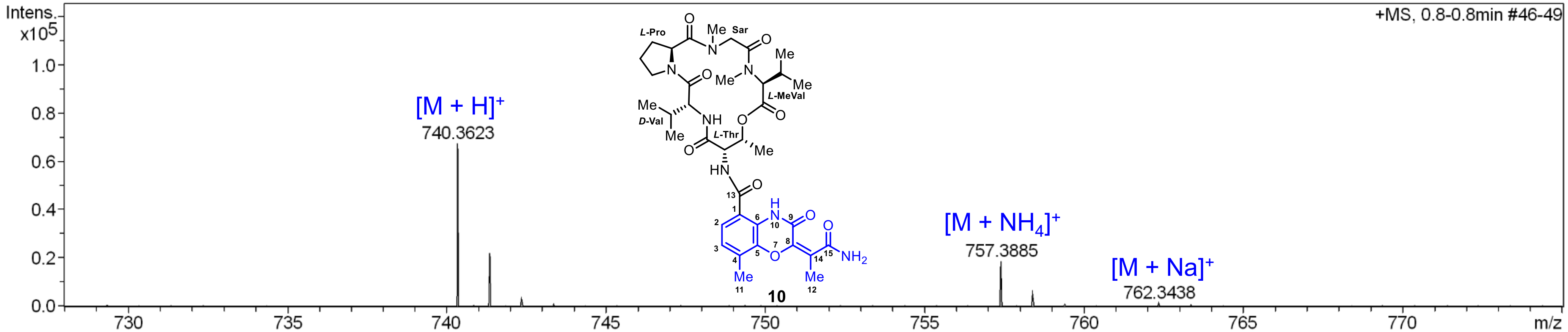

Meas. m/z	#	Ion Formula	Score	m/z	err [ppm]	err [mDa]	mSigma	rdb	e⁻ Conf	N-Rule
740.3623	1	C36H50N7O10	100.00	740.3614	1.3	1.0	57.1	15.5	even	ok
757.3885	1	C36H53N8O10	100.00	757.3879	-0.8	-0.6	91.6	14.5	even	ok
762.3438	1	C36H49N7NaO10	100.00	762.3433	-0.6	-0.5	46.9	15.5	even	ok

Figure S99. ^1H NMR (600 MHz) spectrum of compound **10** in DMSO-d_6

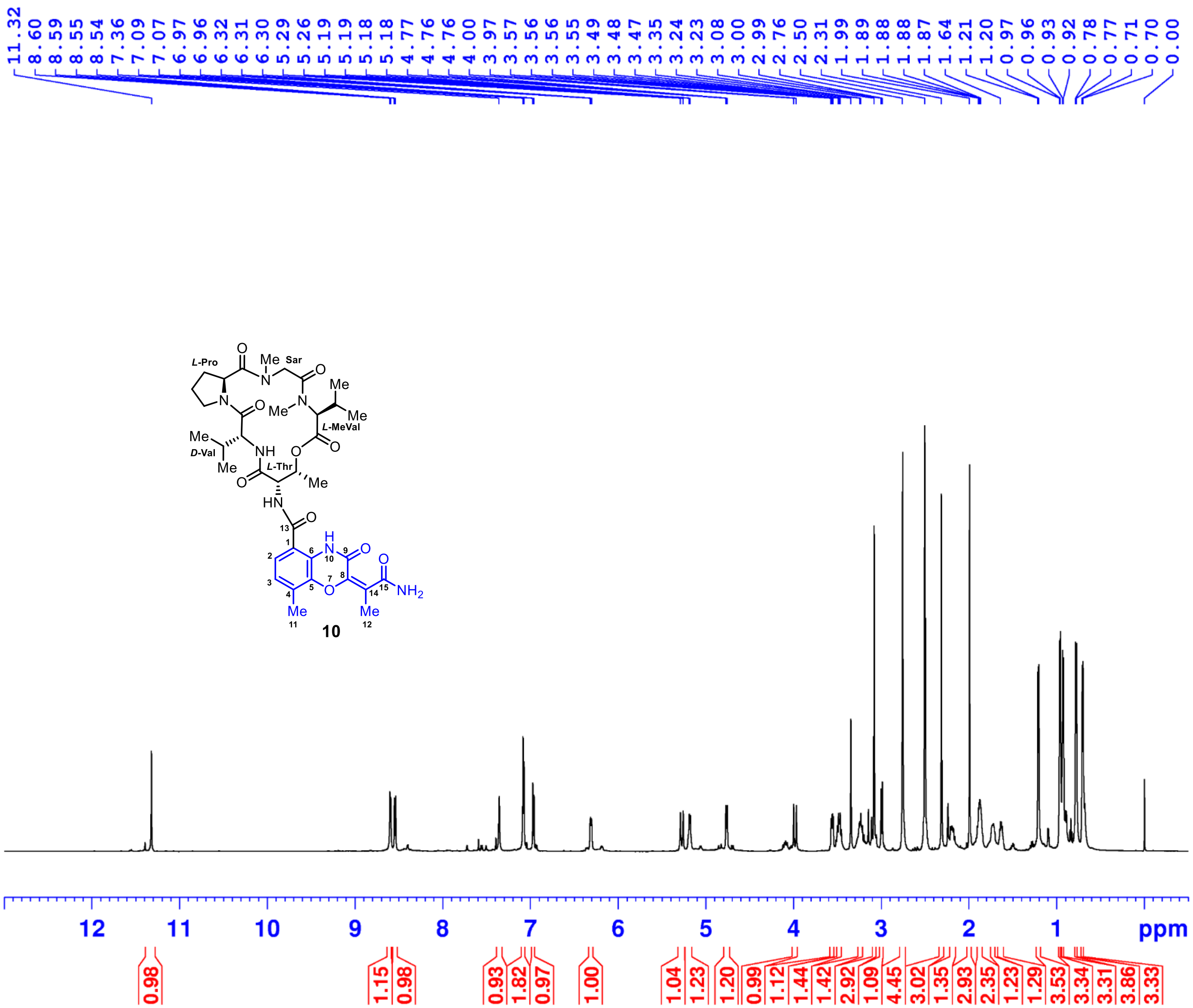

NAME	S_4_H	
EXPNO	21	
PROCNO	1	
Date_	20250316	
Time	7.31	h
INSTRUM	Avance NEO 600	
PROBHD	Z154705_0139 (	
PULPROG	zg30	
TD	65536	
SOLVENT	DMSO	
NS	4	
DS	2	
SWH	11904.762	Hz
FIDRES	0.363304	Hz
AQ	2.7525620	sec
RG	45.2	
DW	42.000	usec
DE	8.79	usec
TE	293.0	K
D1	1.00000000	sec
TD0	1	
SFO1	600.1937062	MHz
NUC1	1H	
P0	3.33	usec
P1	10.00	usec
SI	65536	
SF	600.1900026	MHz
WDW	EM	
SSB	0	
LB	0.30	Hz
GB	0	
PC	1.00	

Figure S99-continue

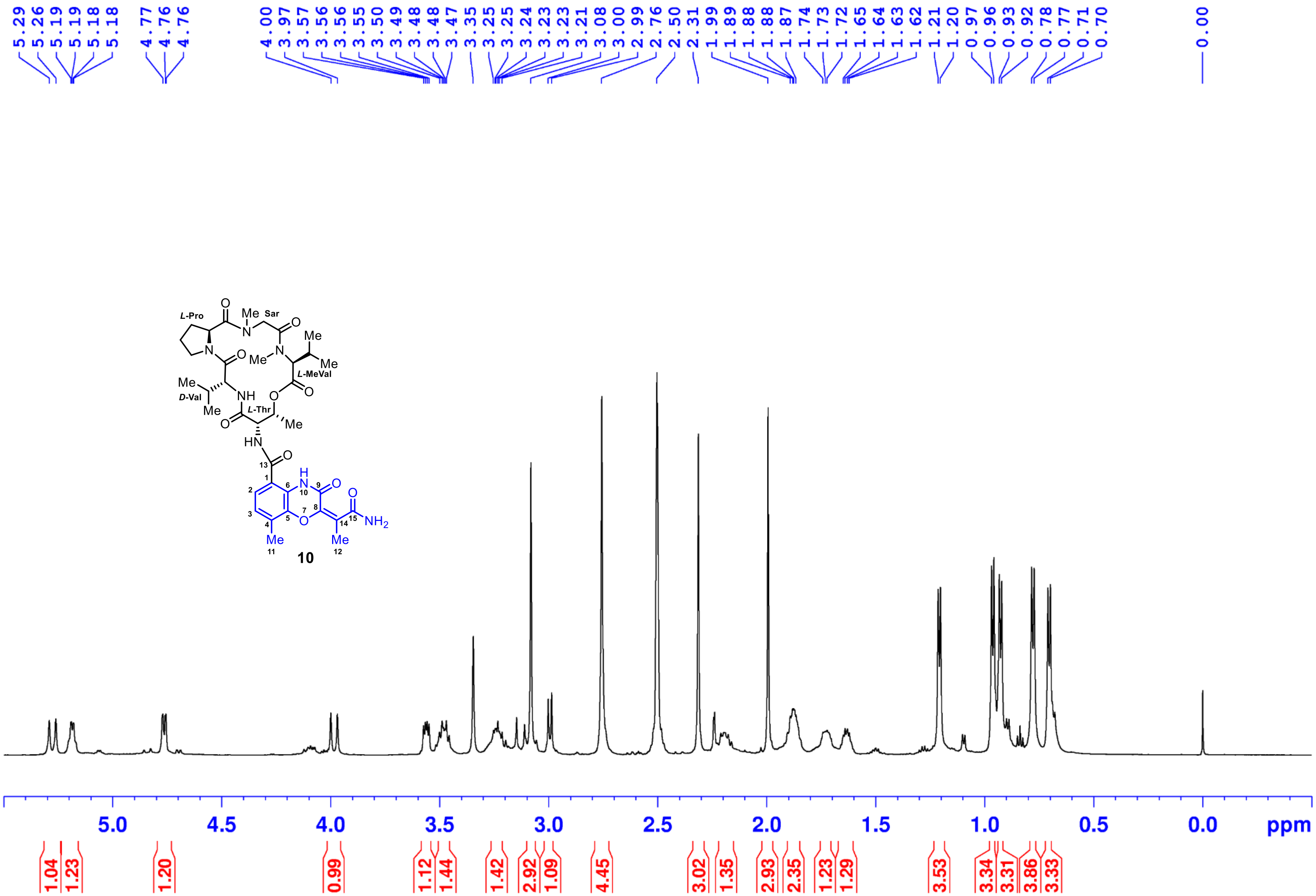

Figure S99-continue

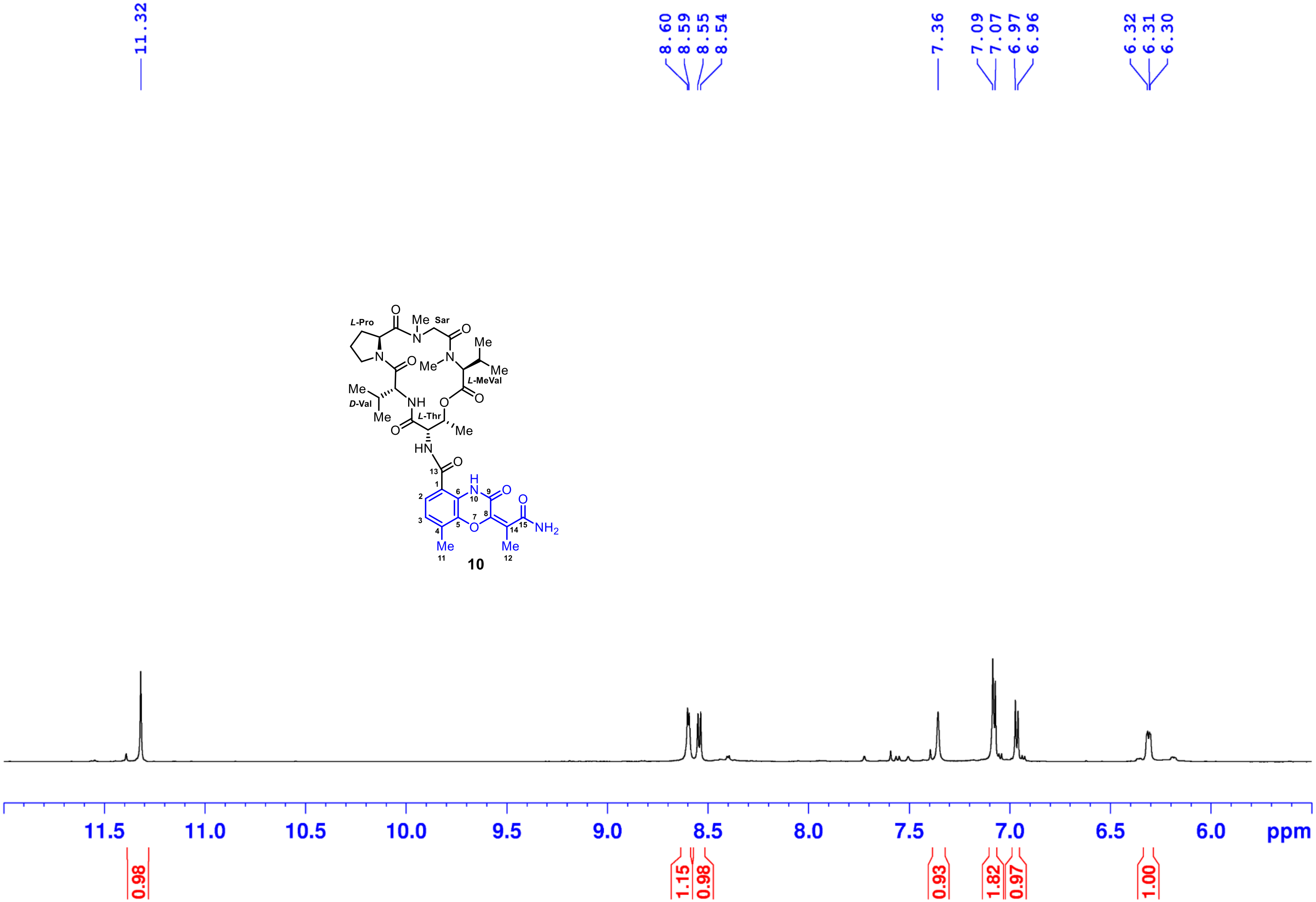

10

Figure S100. ^{13}C NMR (150 MHz) spectrum of compound **10** in DMSO-d_6

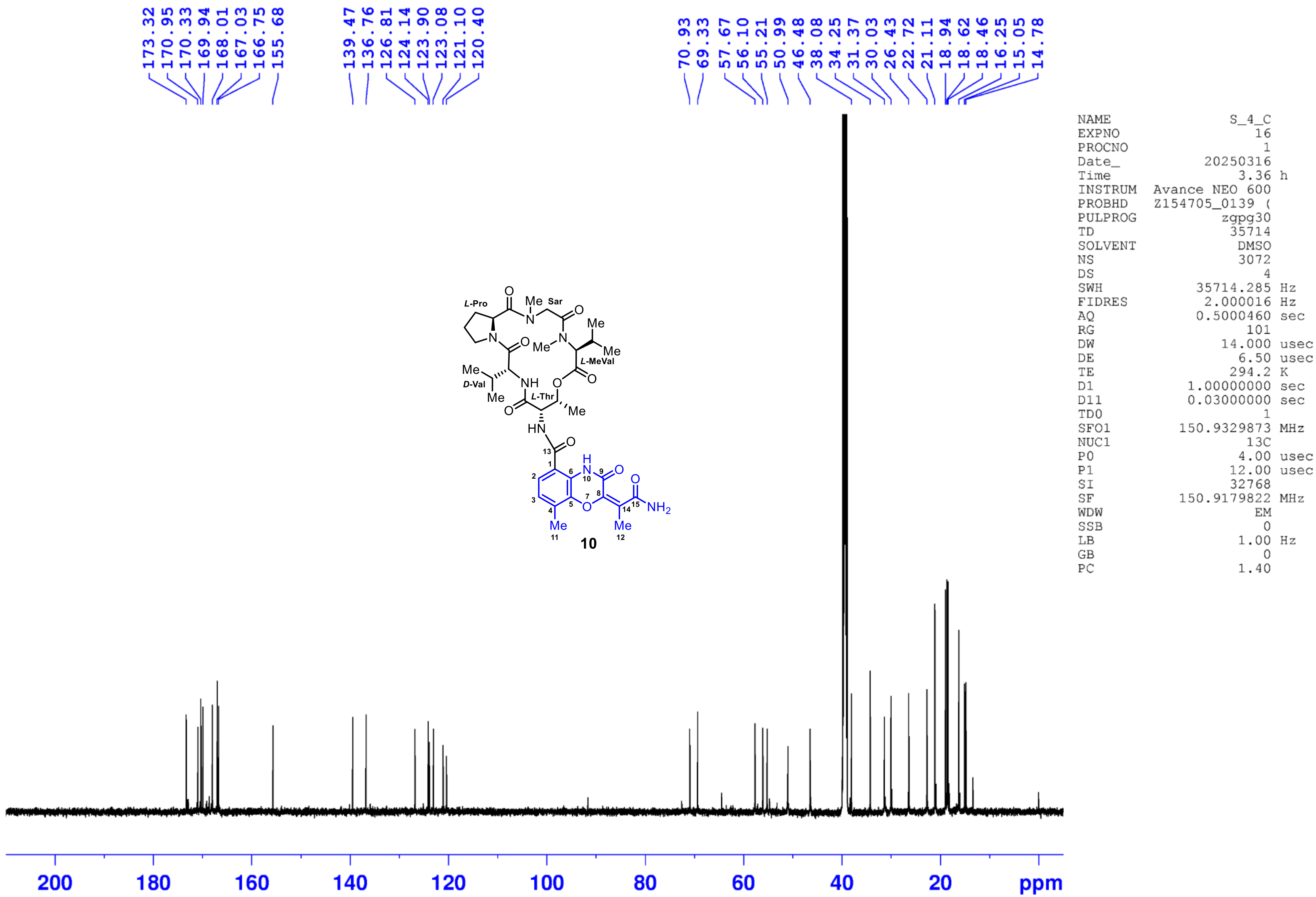

Figure S100-continue

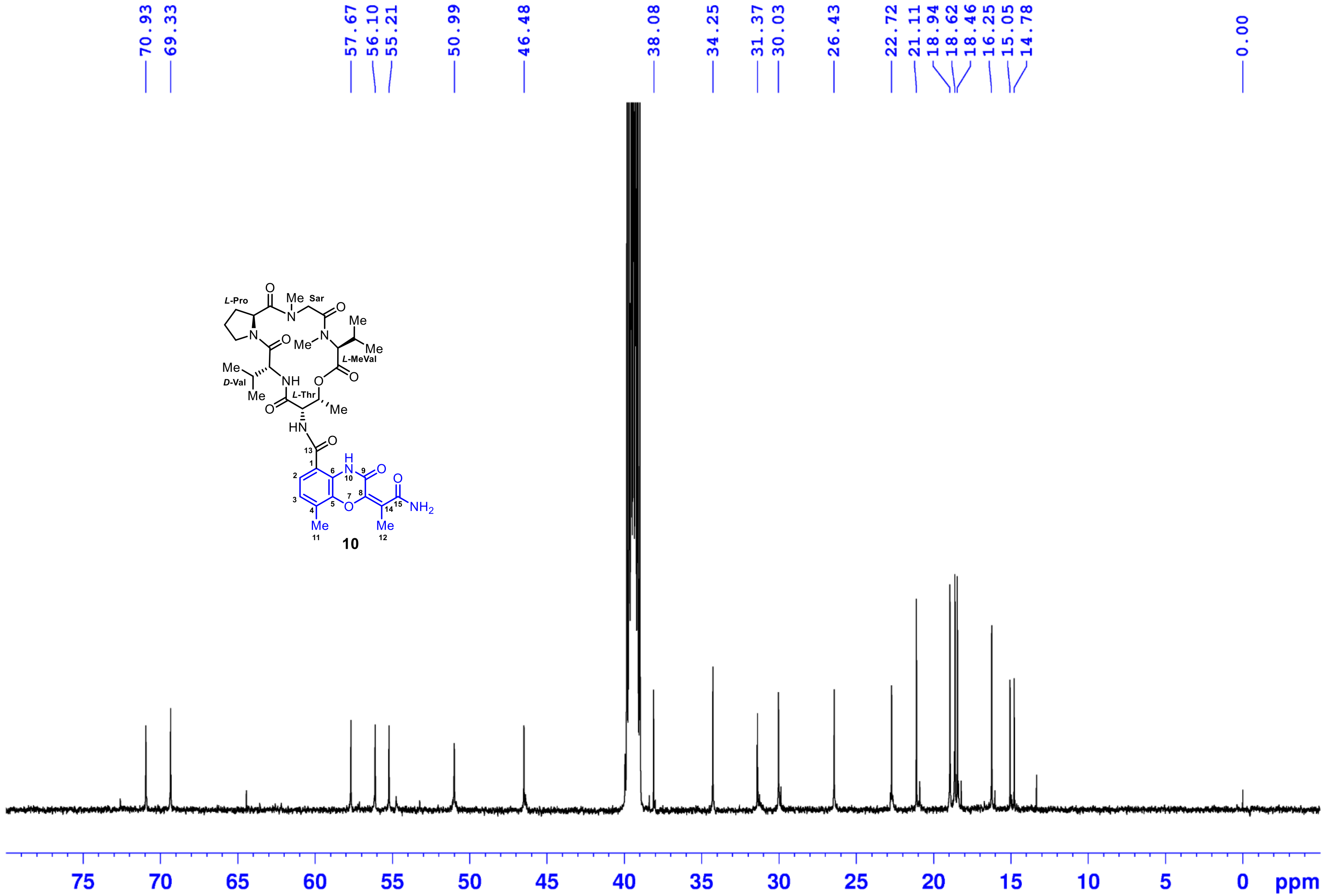

Figure S100-continue

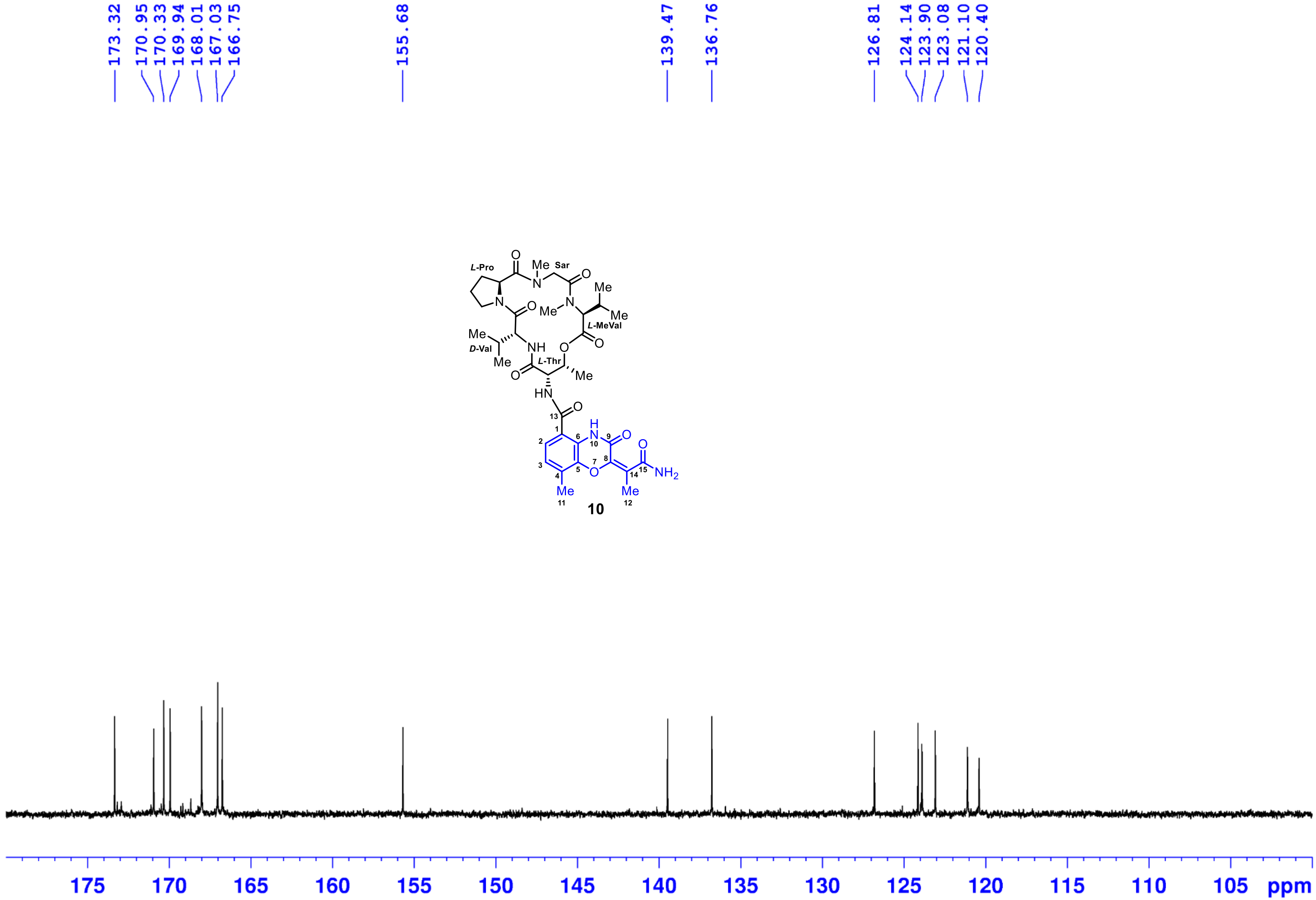

10

Figure S101. DEPT135 (150 MHz) spectrum of compound **10** in DMSO-d_6

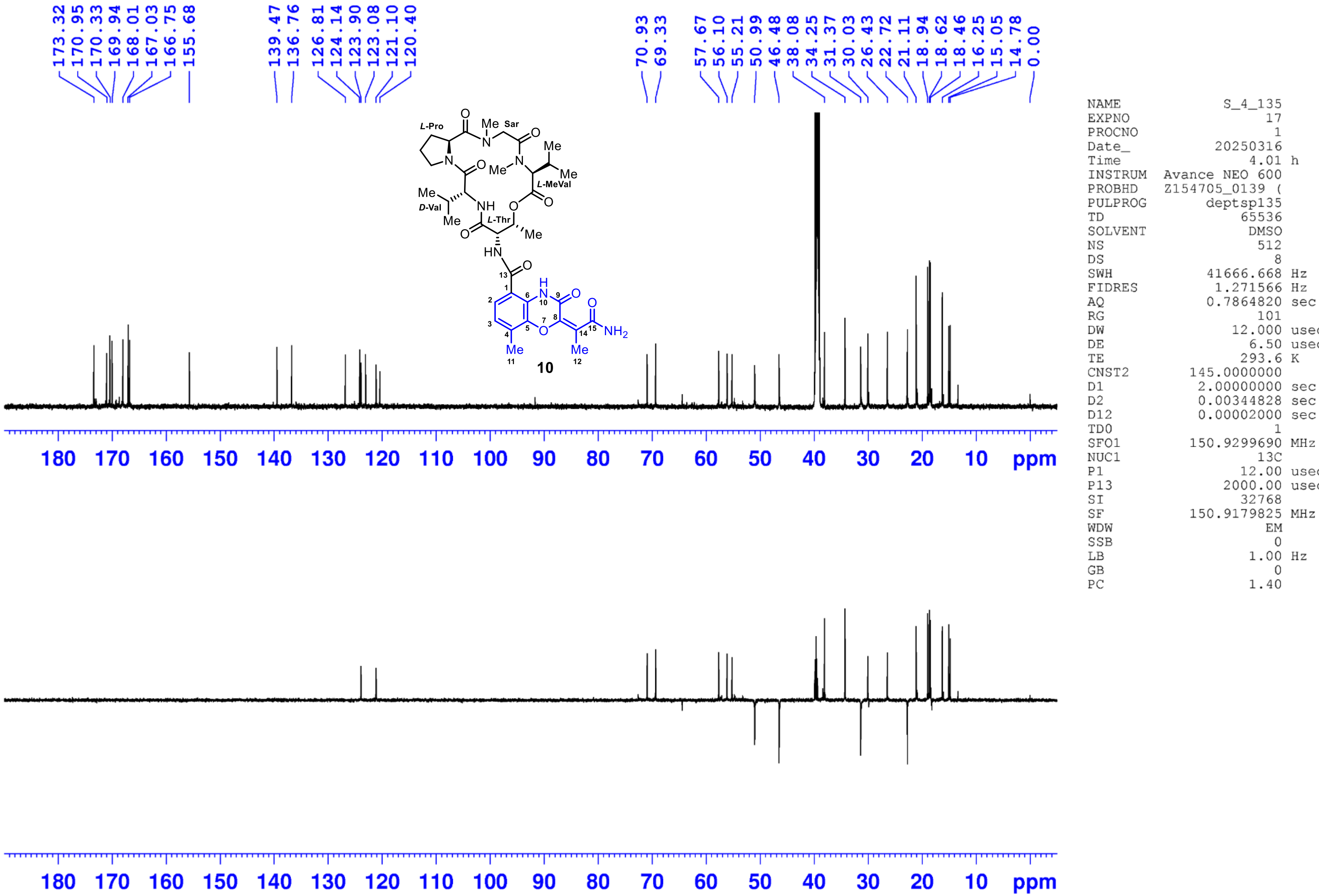

Figure S101-continue

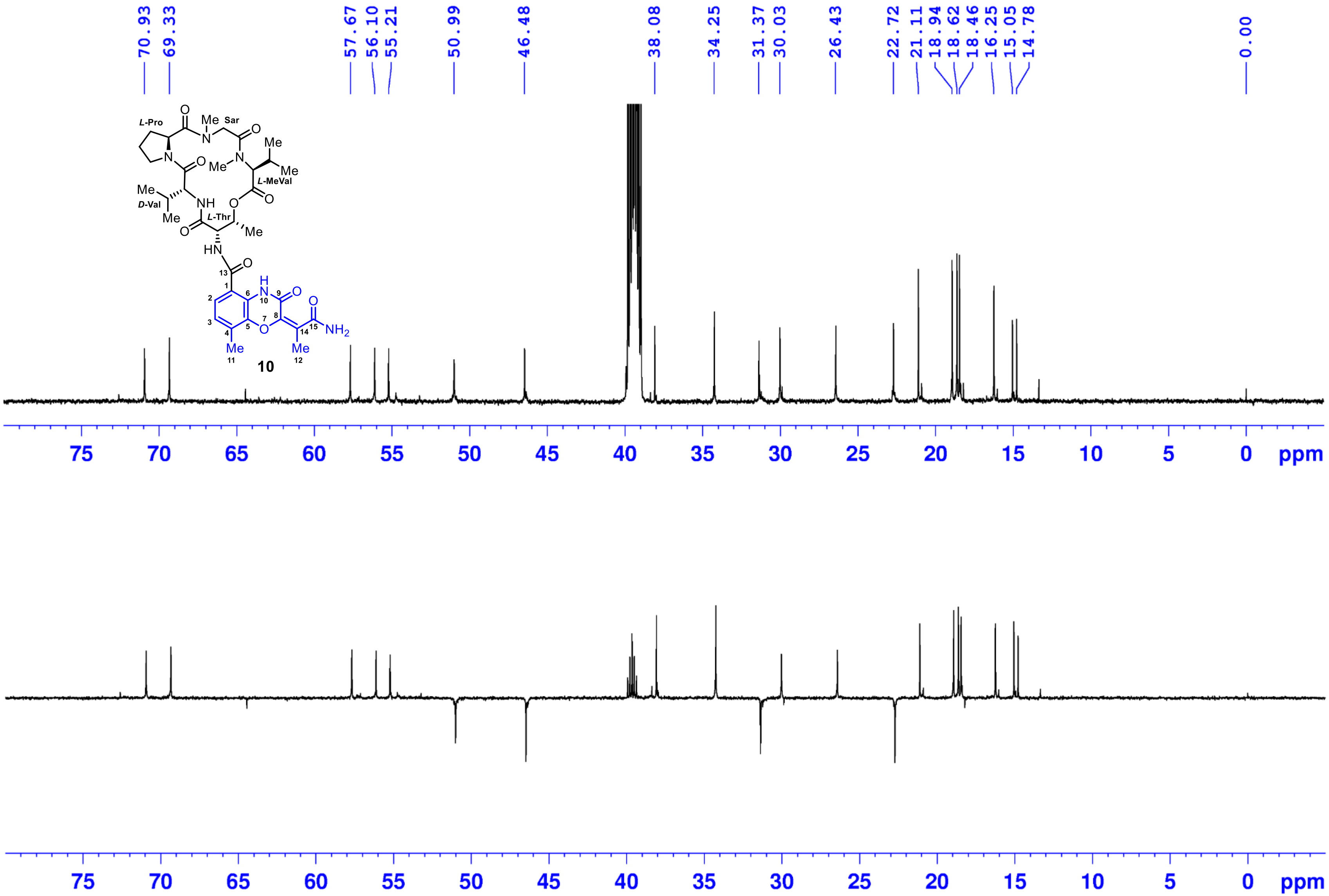

Figure S101-continue

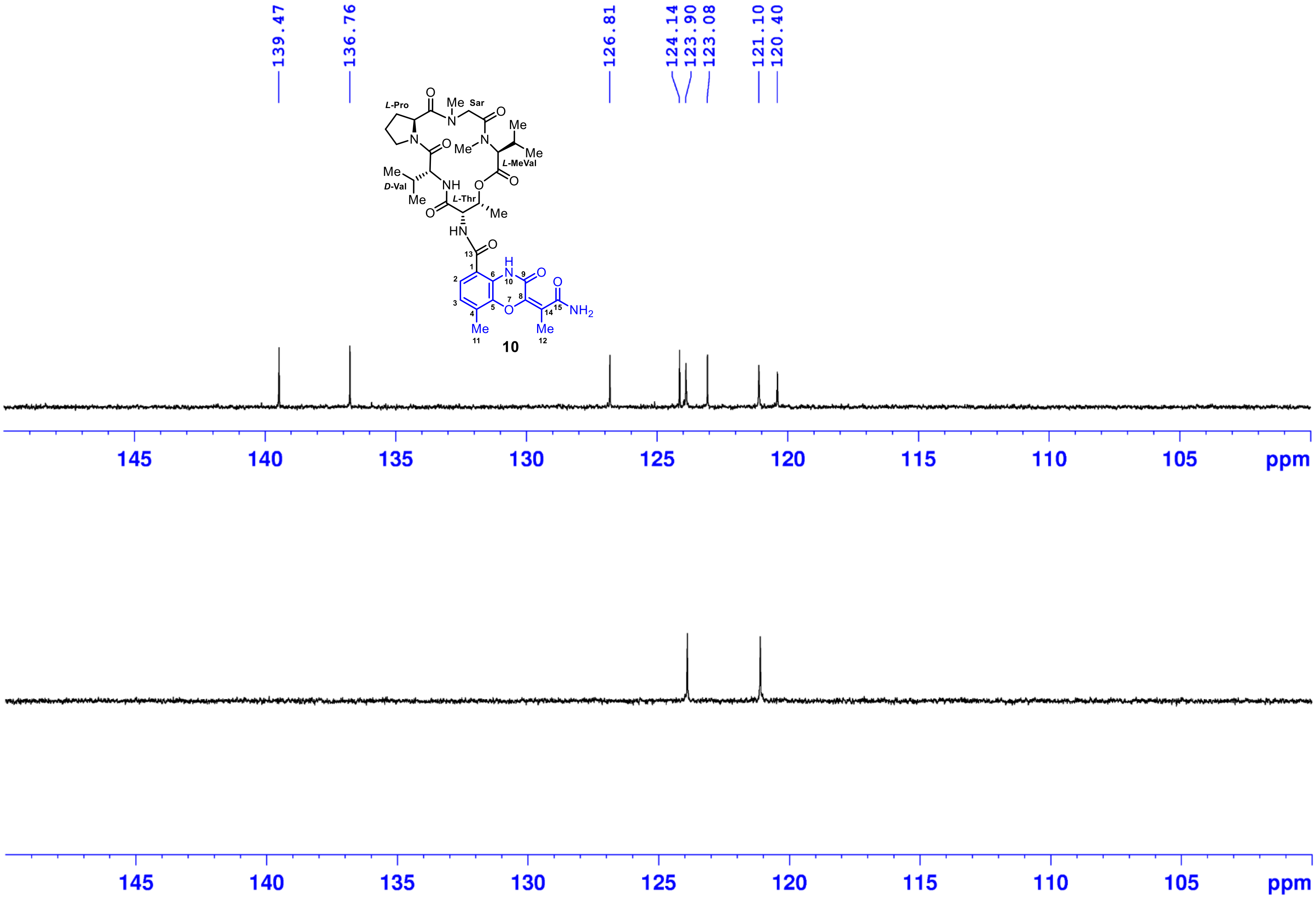

Figure S102. ^{1}H–^{1}H COSY (600 MHz) spectrum of compound **10** in DMSO-d_6

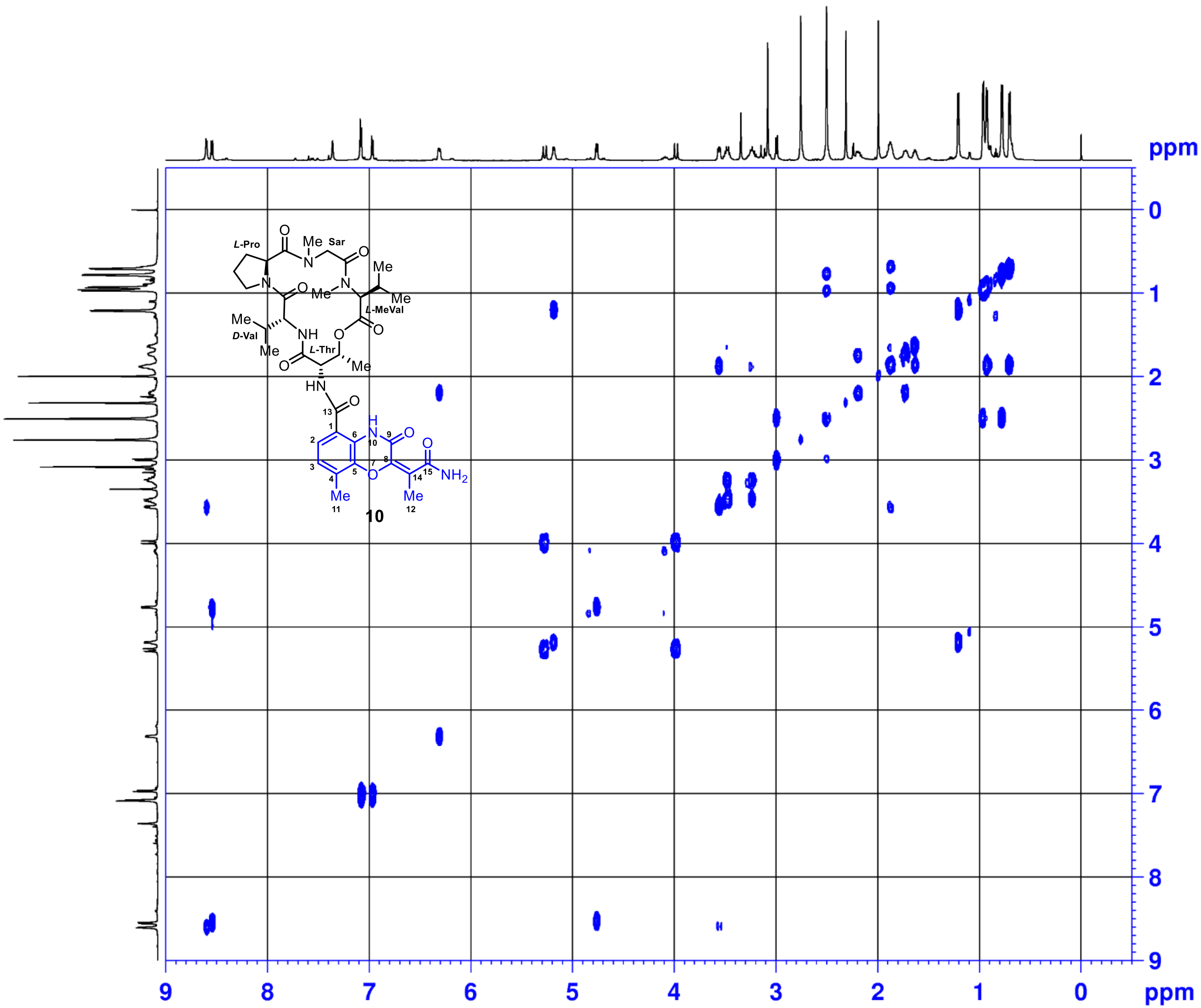

NAME	S_4_COSY
EXPNO	18
PROCNO	1
Date_	20250316
Time	4.56 h
INSTRUM	Avance NEO 600
PROBHD	Z154705_0139 (
PULPROG	cosygpmfqf
TD	2048
SOLVENT	DMSO
NS	12
DS	16
SWH	10869.565 Hz
FIDRES	10.614810 Hz
AQ	0.0942580 sec
RG	101
DW	46.000 usec
DE	6.50 usec
TE	293.2 K
D0	0.00000300 sec
D1	2.00000000 sec
D13	0.00000400 sec
D16	0.00020000 sec
IN0	0.00009200 sec
ND0	1
TD	128
SFO1	600.1936 MHz
FIDRES	84.918480 Hz
SW	18.110 ppm
FnMODE	QF
SI	1024
SF	600.1900000 MHz
WDW	SINE
SSB	0
LB	0.00 Hz
GB	0
PC	1.40
SI	1024
MC2	QF
SF	600.1900000 MHz
WDW	SINE
SSB	0
LB	0.00 Hz
GB	0

Figure S102-continue

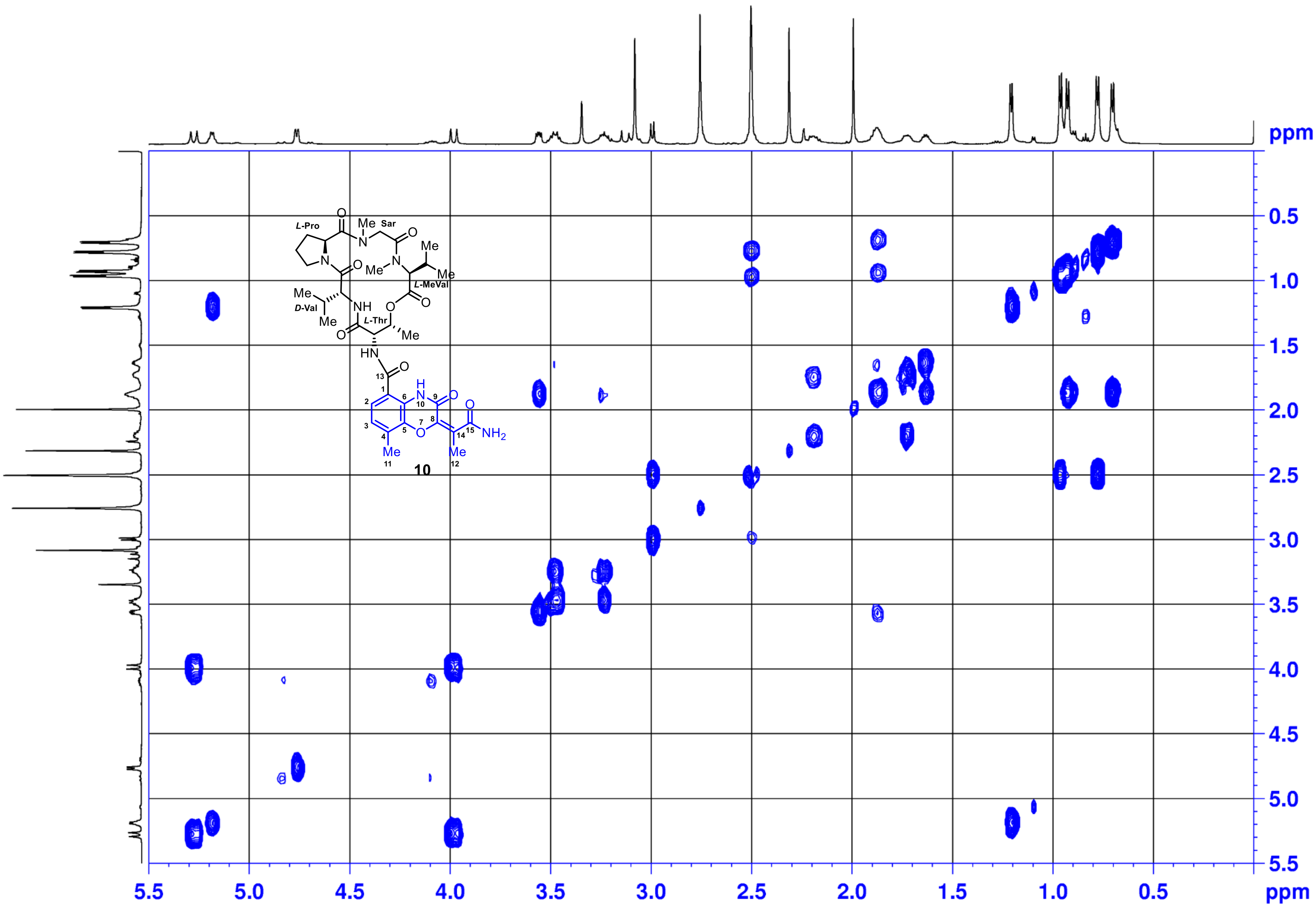

Figure S102-continue

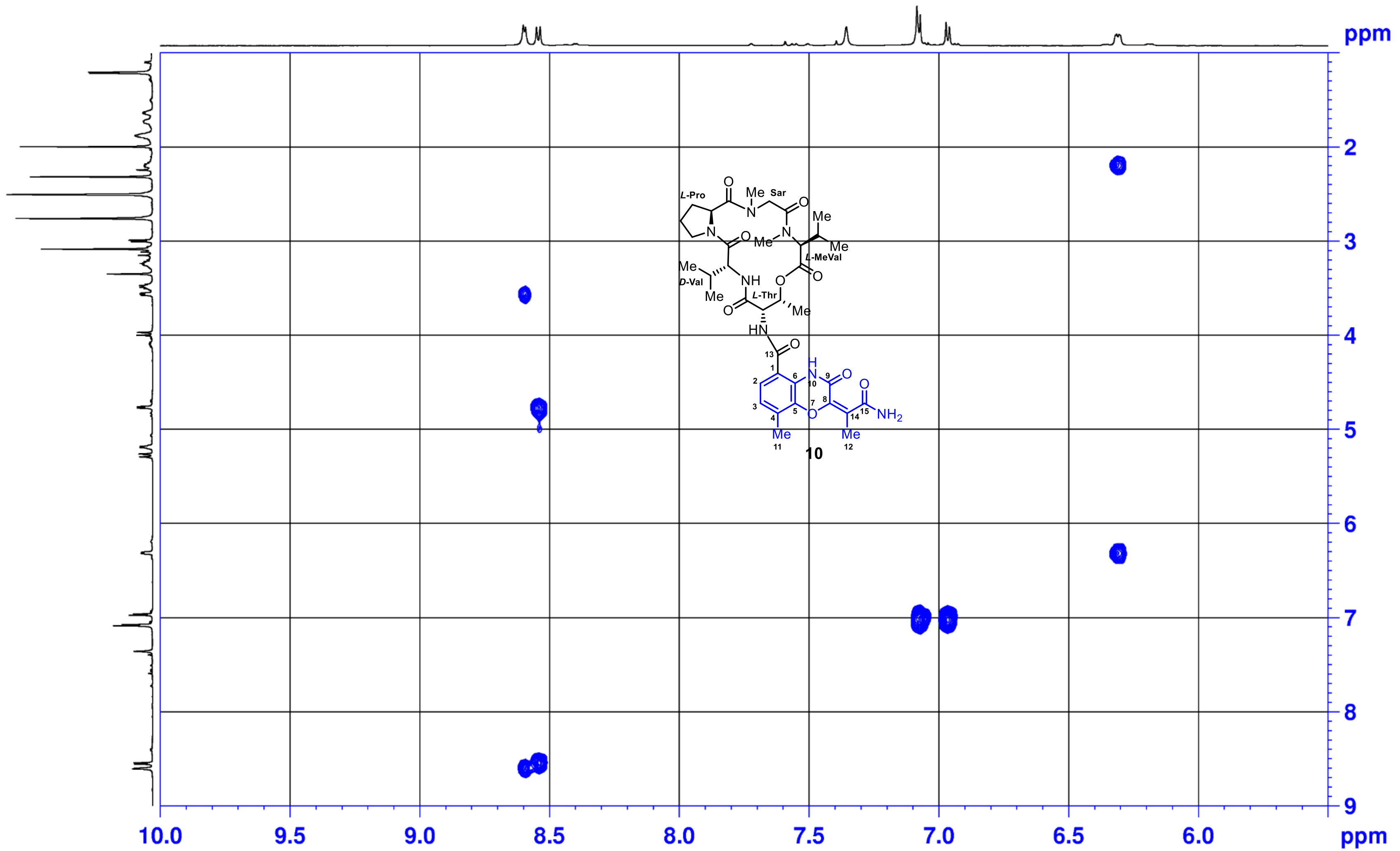

Figure S103. HSQC (600 MHz) spectrum of compound **10** in DMSO-d_6

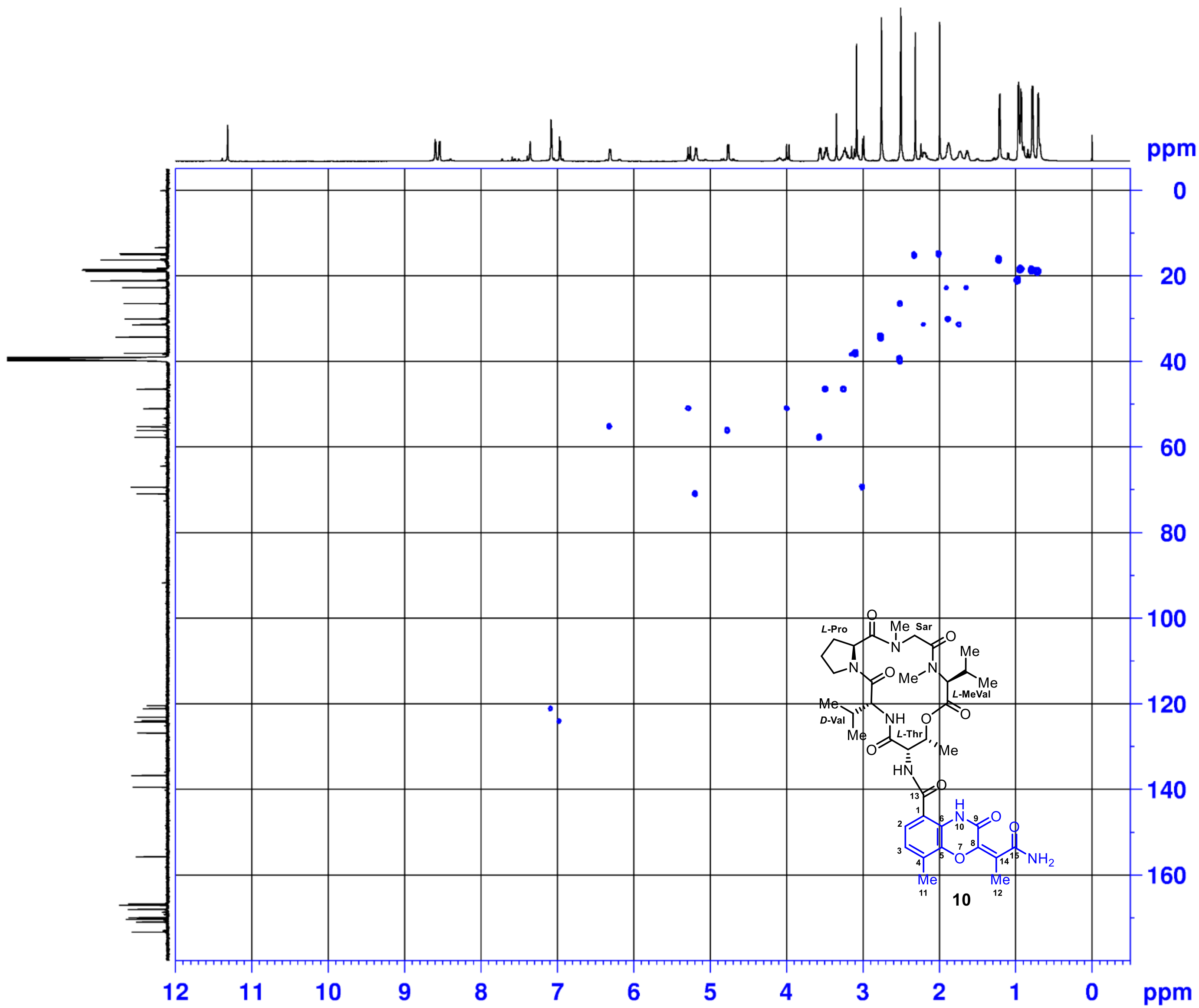

NAME	S_4_HSQC
EXPNO	23
PROCNO	1
Date_	20250316
Time	8.52 h
INSTRUM	Avance NEO 600
PROBHD	Z154705_0139 (
PULPROG	hsqcetgp
TD	1024
SOLVENT	DMSO
NS	12
DS	16
SWH	10869.565 Hz
FIDRES	21.229620 Hz
AQ	0.0471540 sec
RG	101
DW	46.000 usec
DE	6.50 usec
TE	293.1 K
CNST2	145.0000000
D0	0.00000300 sec
D1	1.50000000 sec
D4	0.00172414 sec
D11	0.03000000 sec
D16	0.00020000 sec
IN0	0.00001142 sec
ND0	2
TD	256
SFO1	150.9292 MHz
FIDRES	170.974503 Hz
SW	290.000 ppm
FnMODE	Echo-Antiecho
SI	1024
SF	600.1899971 MHz
WDW	QSINE
SSB	2
LB	0.00 Hz
GB	0
PC	1.40
SI	1024
MC2	echo-antiecho
SF	150.9179735 MHz
WDW	QSINE
SSB	2
LB	0.00 Hz
GB	0

Figure S103-continue

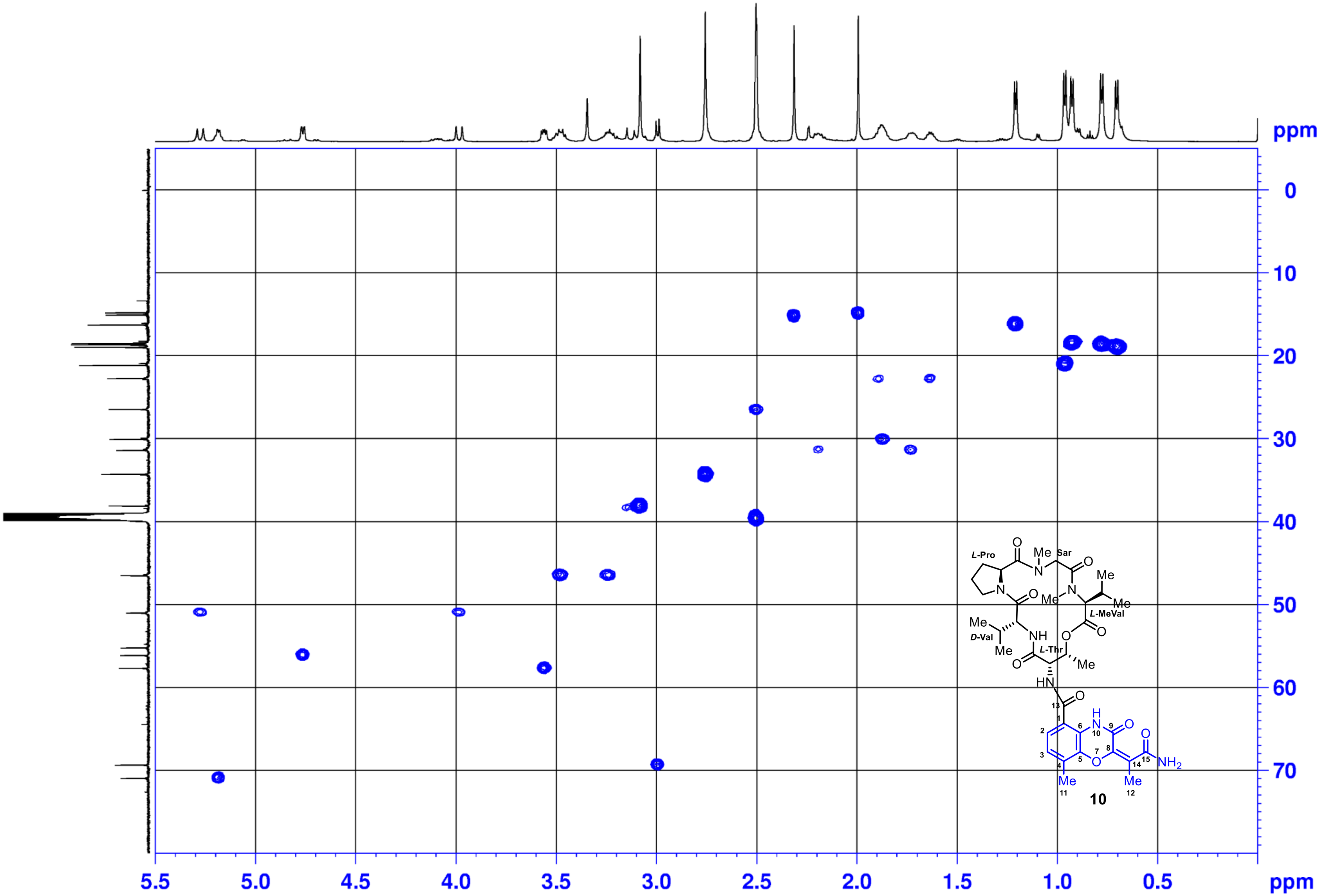

Figure S103-continue

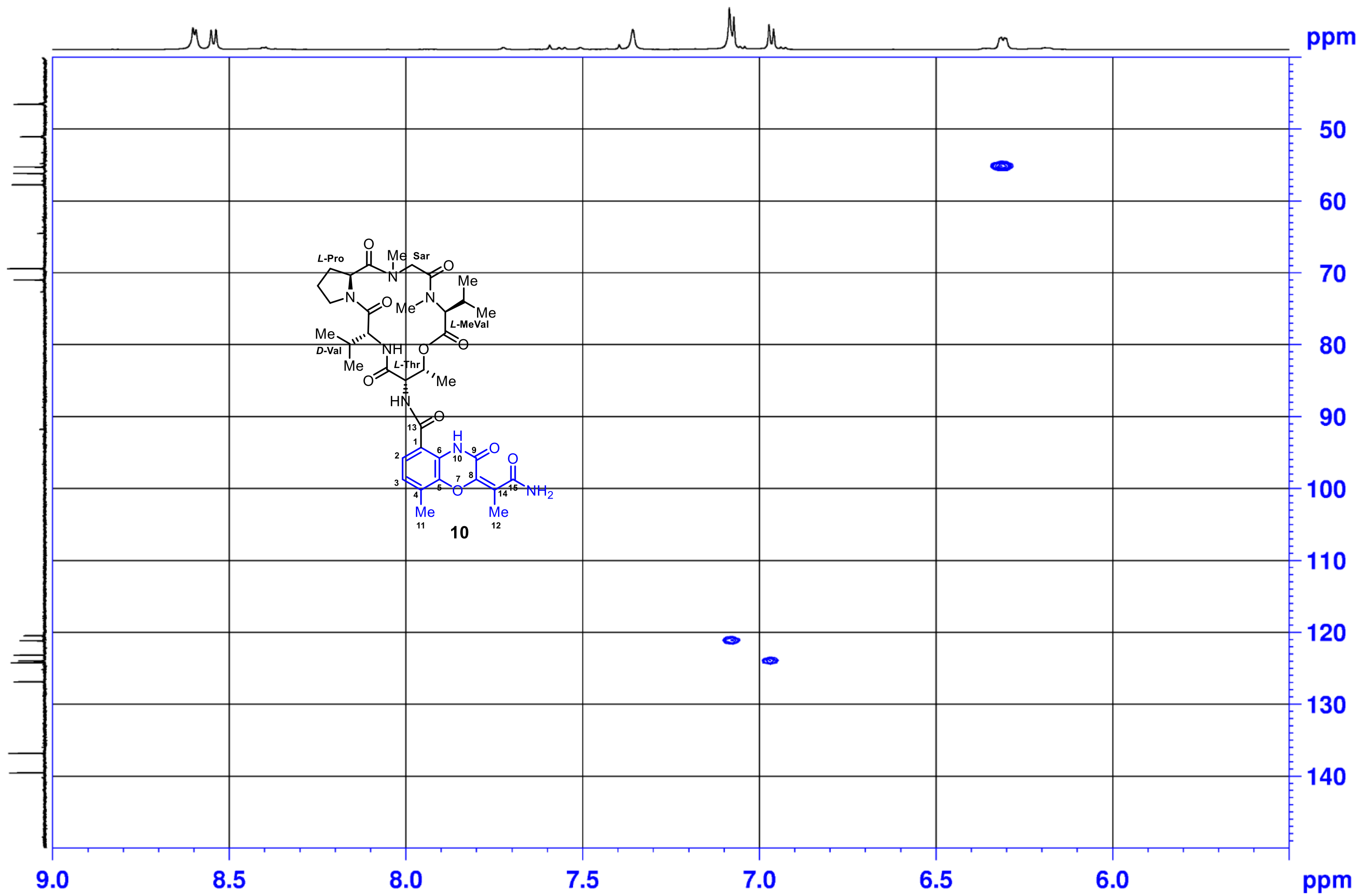

Figure S104. HMBC (600 MHz) spectrum of compound **10** in DMSO-d_6

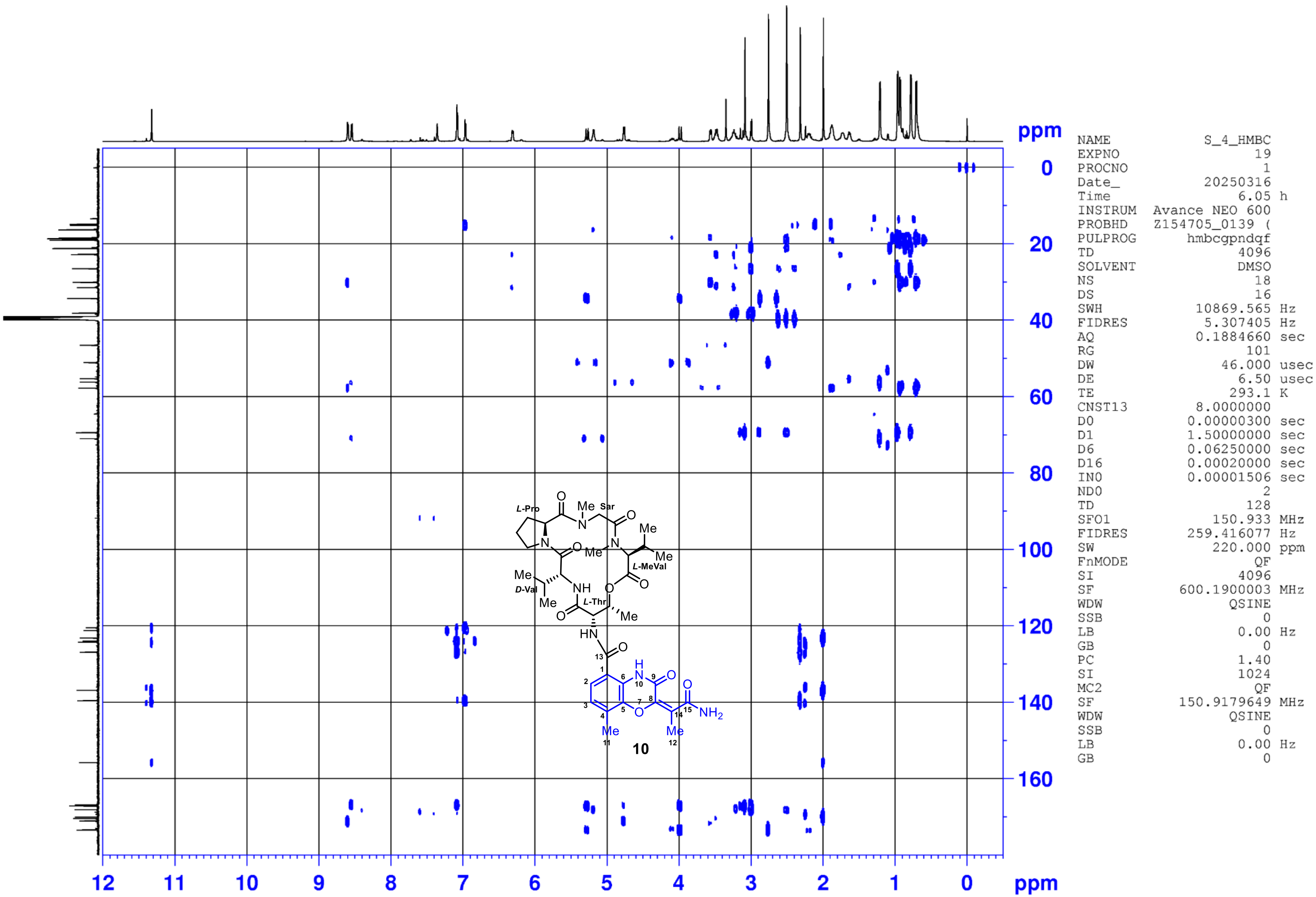

Figure S104-continue

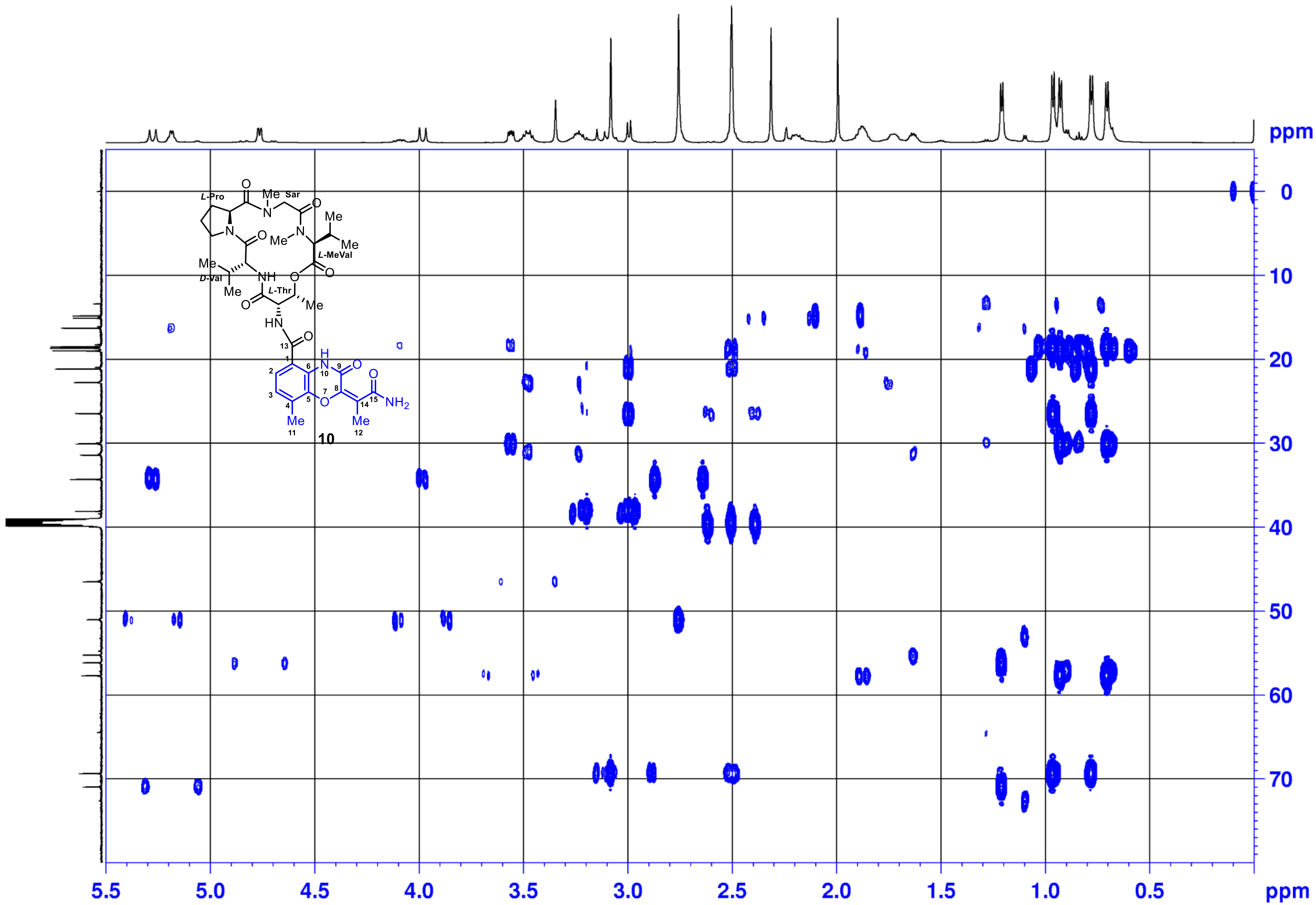

Figure S104-continue

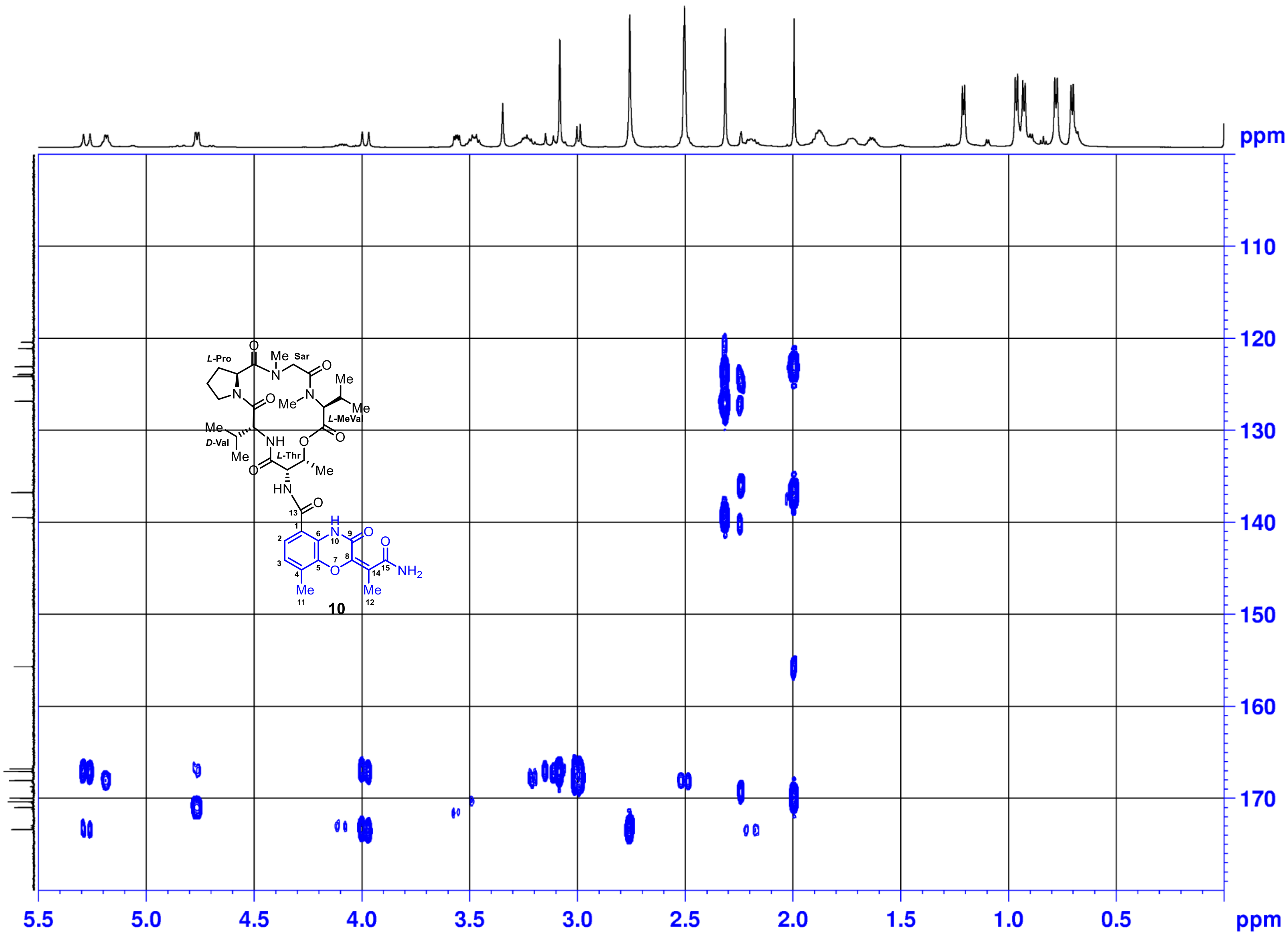

Figure S104-continue

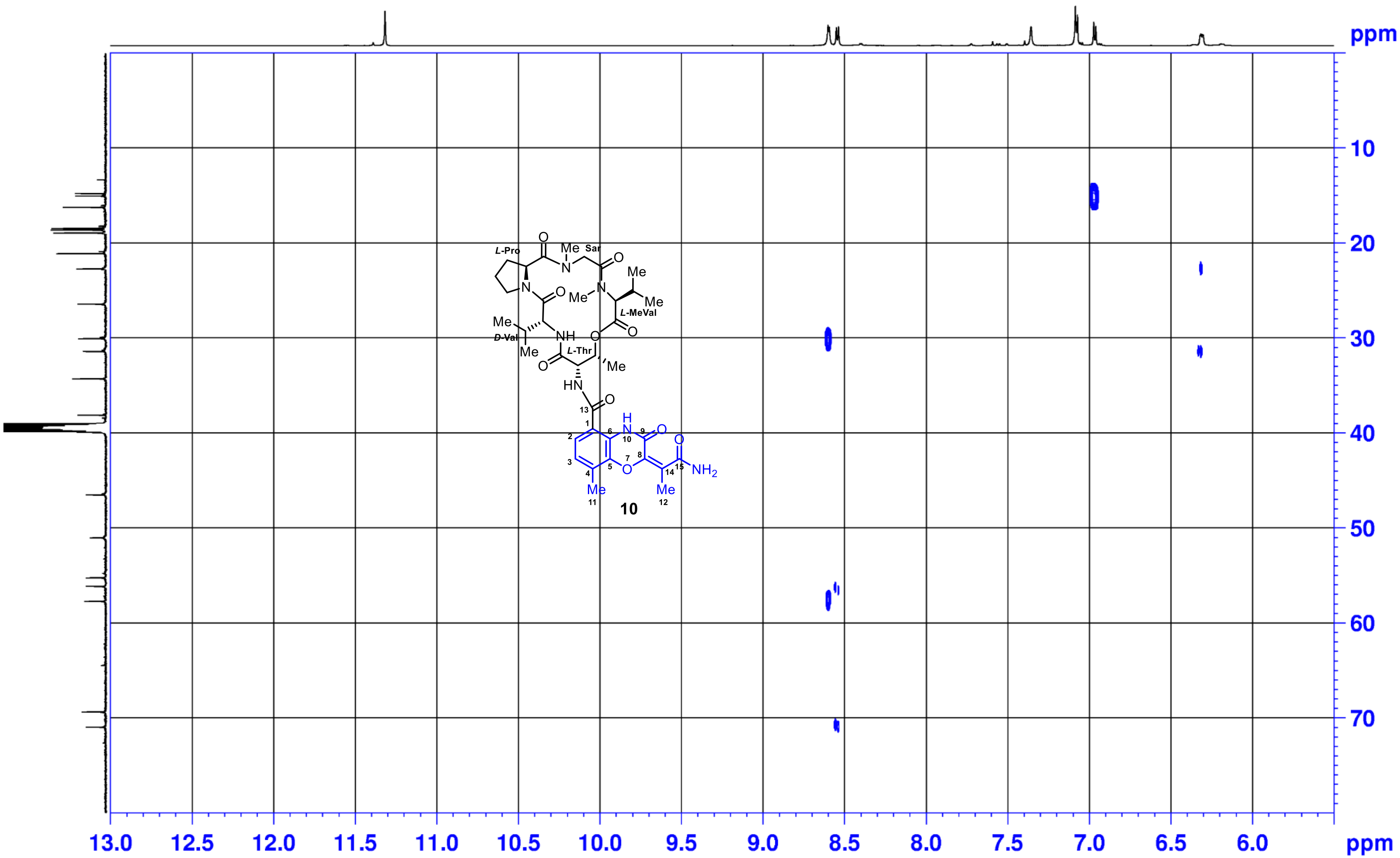

Figure S104-continue

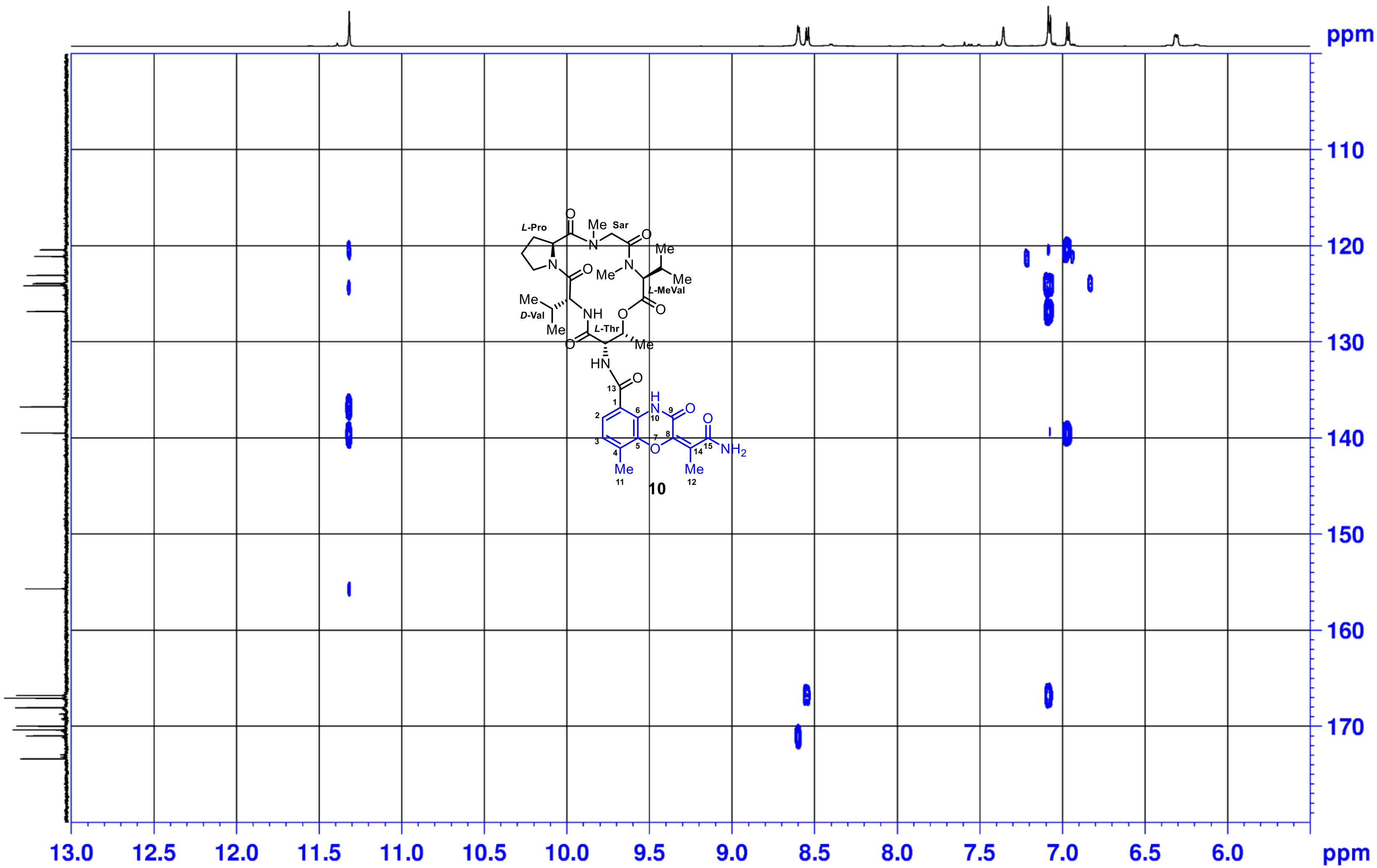

Figure S105. ROESY (600 MHz) spectrum of compound **10** in DMSO-d_6

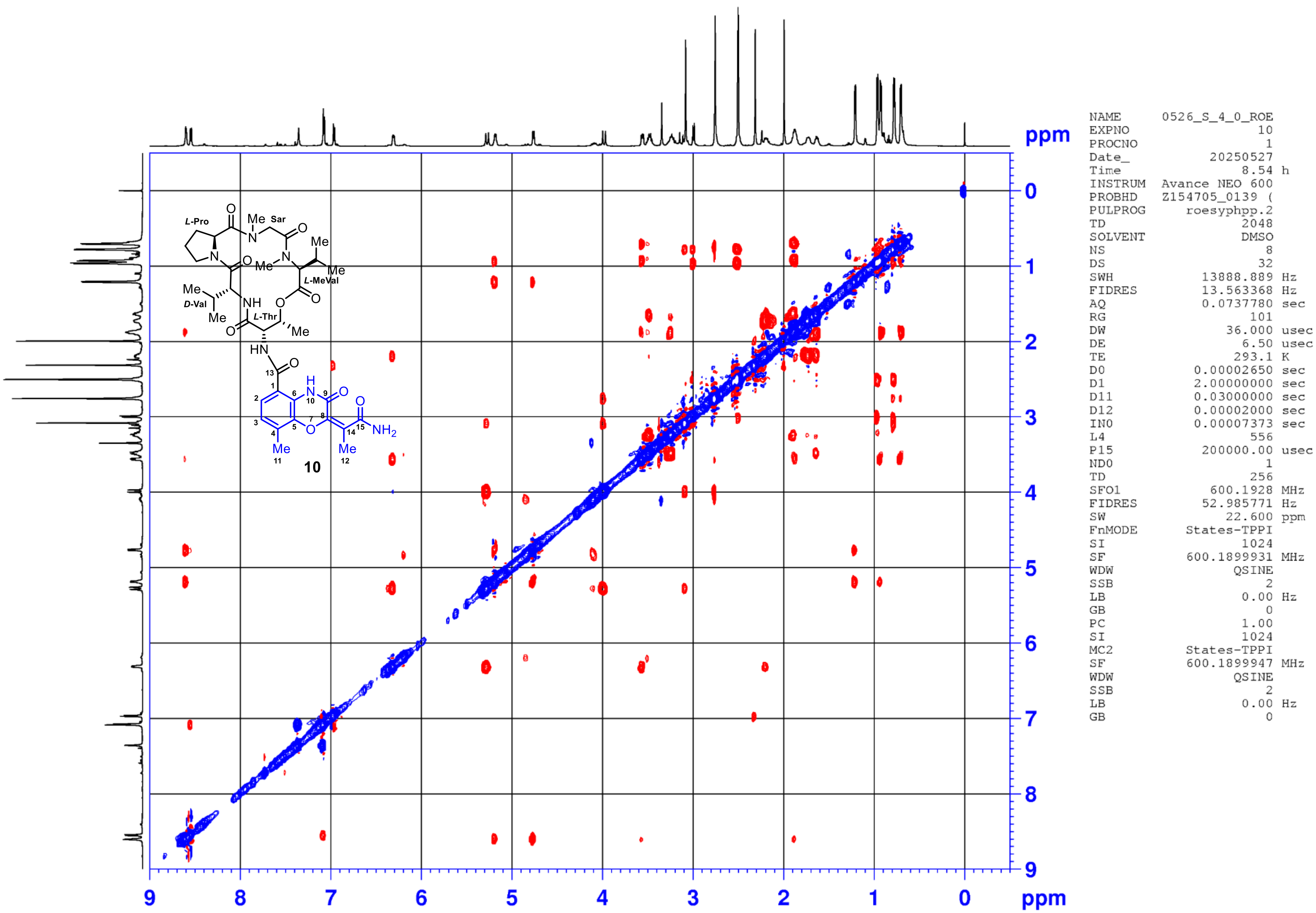

Figure S105-continue

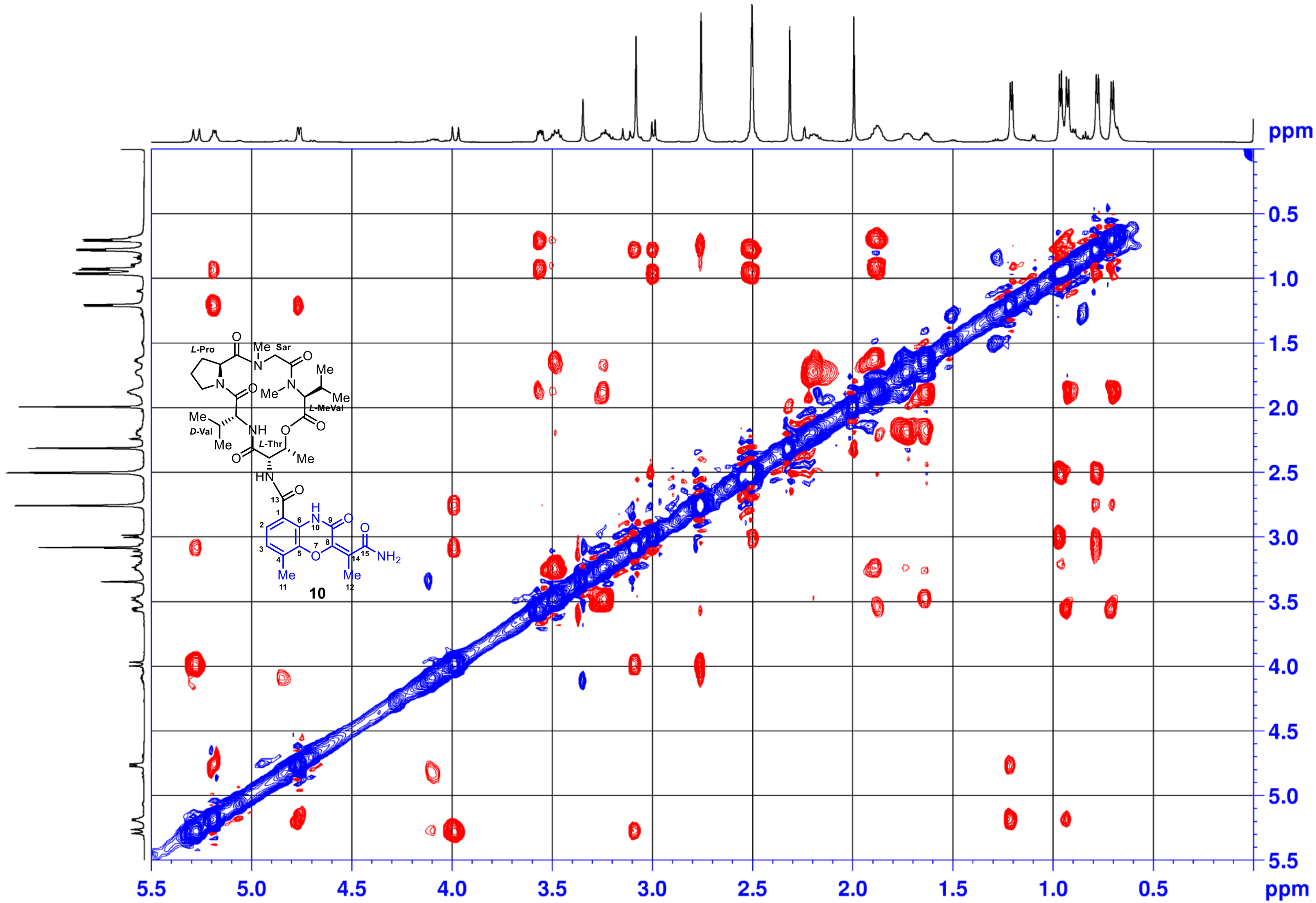

Figure S105-continue

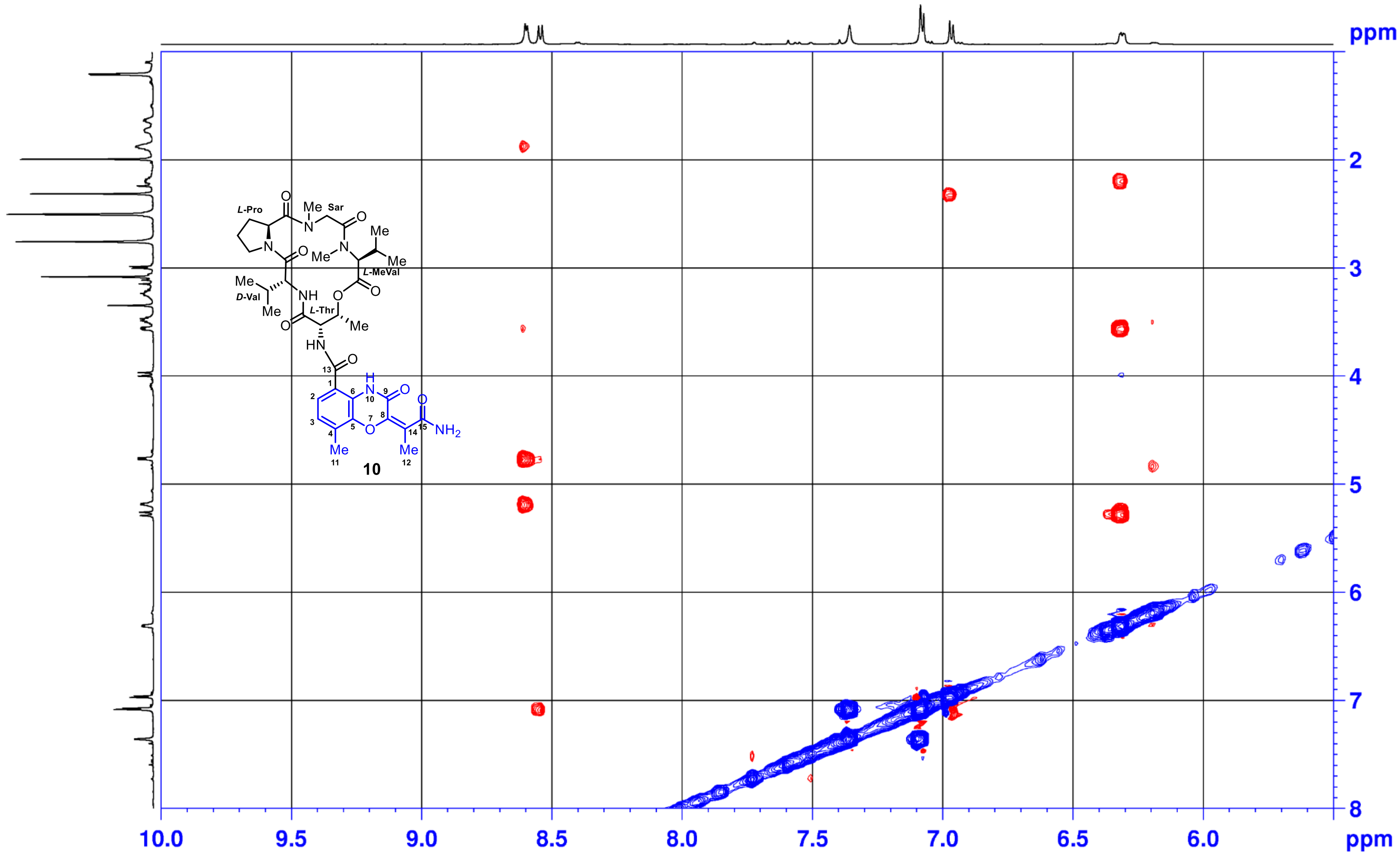

Figure S106. Marfey's analysis of compound **10** and standard amino acids

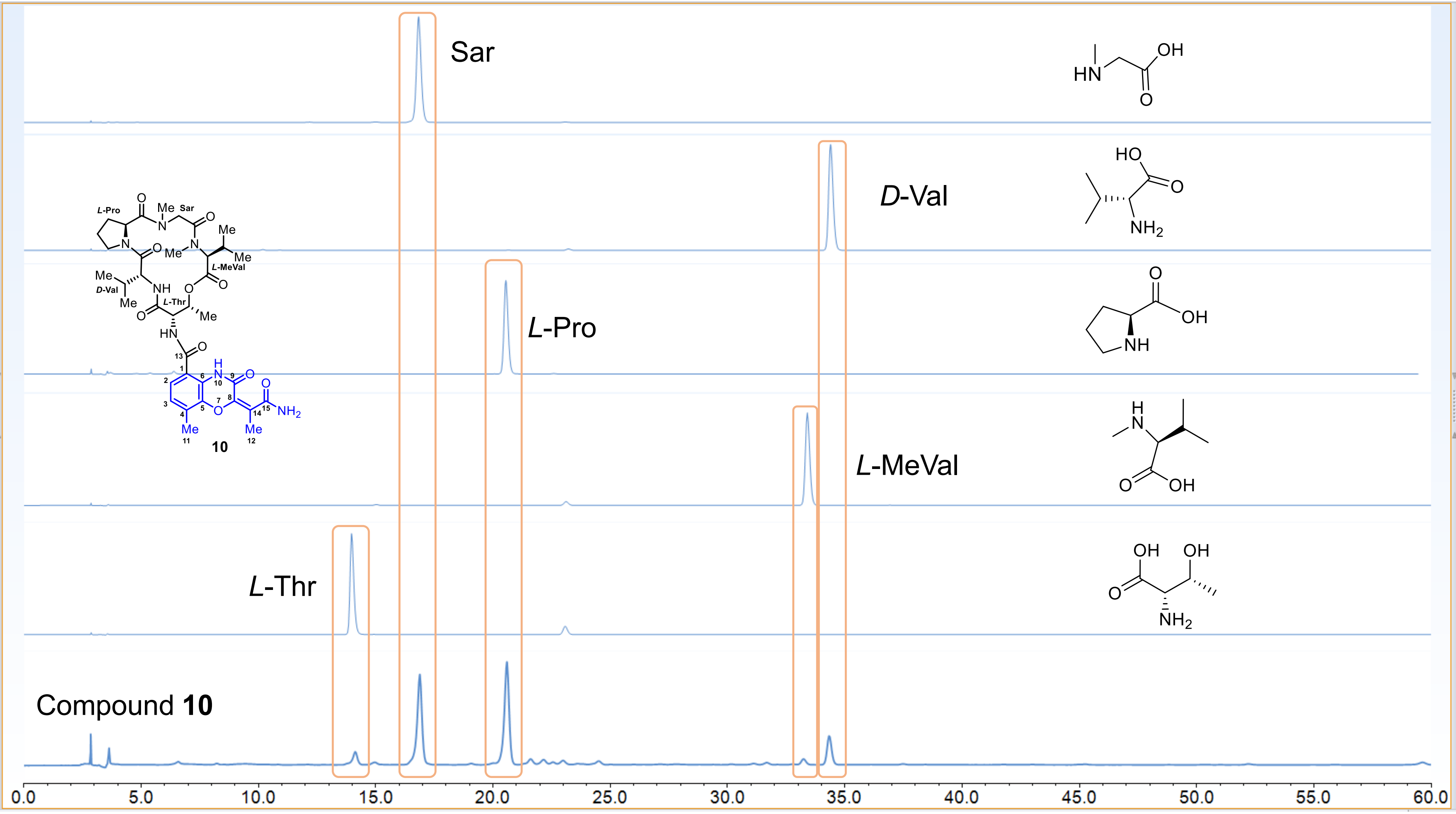

Figure S107. UV spectrum of compound **11**

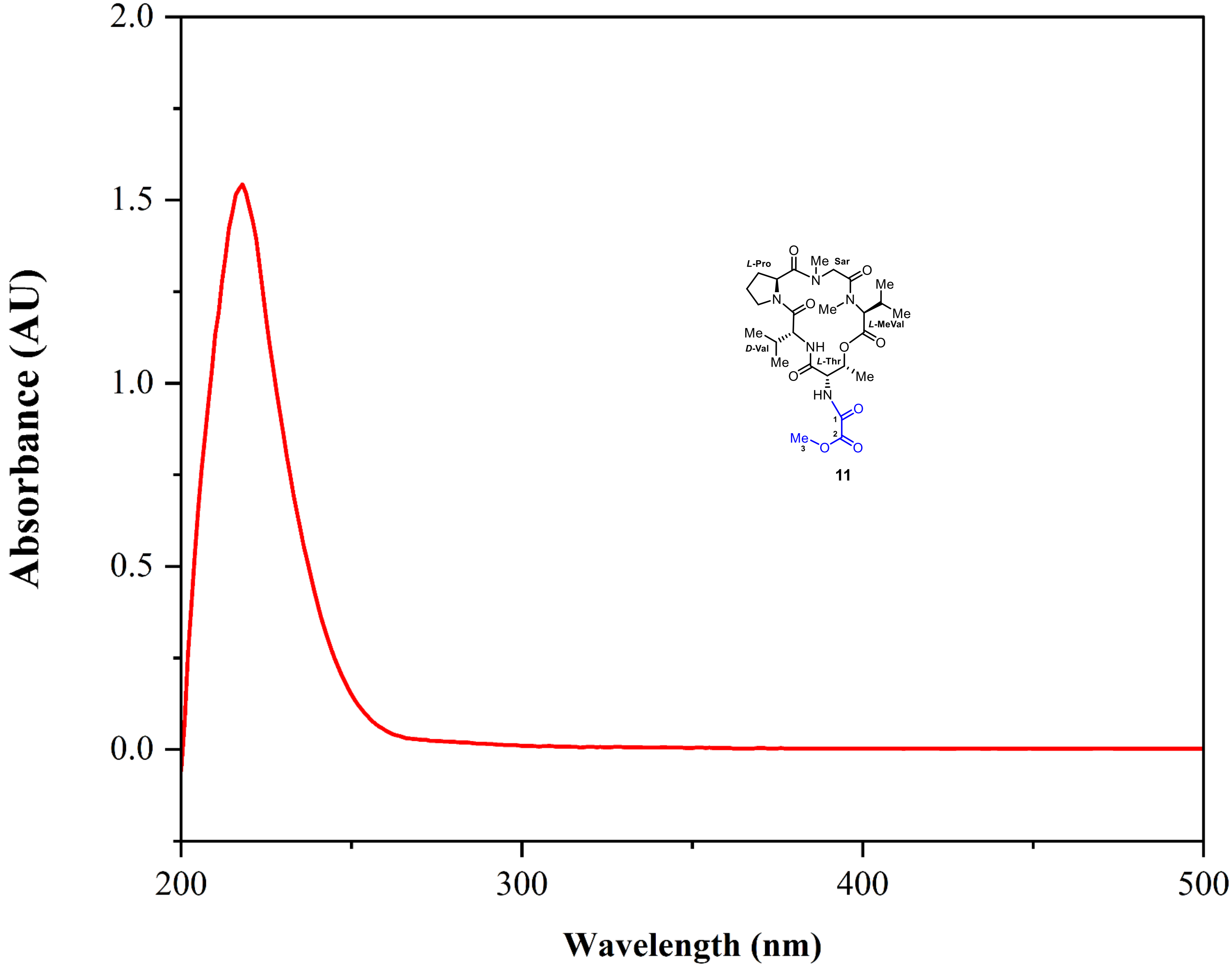

Figure S108. HR-ESIMS for compound **11**

Compound Spectrum SmartFormula Report

Analysis Info

Analysis	D:\Data\SCSIO Metabonomics data\MS\202508\panghe_S-2-4_pos_P1-F-6_806.d	Acquisition Date	2025-08-19 11:25:15
Method	Tune_pos_middle_avg_100-2000_calibration_1.5m.m	Operator	SCSIO User
Sample	panghe_S-2-4_pos	Instrument	timsTOF Pro 2 1875087.11040
Comment			

Acquisition Parameter

Source Type	ESI	Ion Polarity	Positive	Set Nebulizer	0.3 Bar
Scan Begin	100 m/z	Set Capillary	3500 V	Set Dry Heater	200 °C
Scan End	2000 m/z	Set Multipole RF	500.0 Vpp	Set Dry Gas	3.5 l/min
		Set Collision Cell RF	2000.0 Vpp	ICC active	Off
		IMS Active	Off	ICC Target	2000000 cts.
		IMS Collision Cell In	300.0 V		

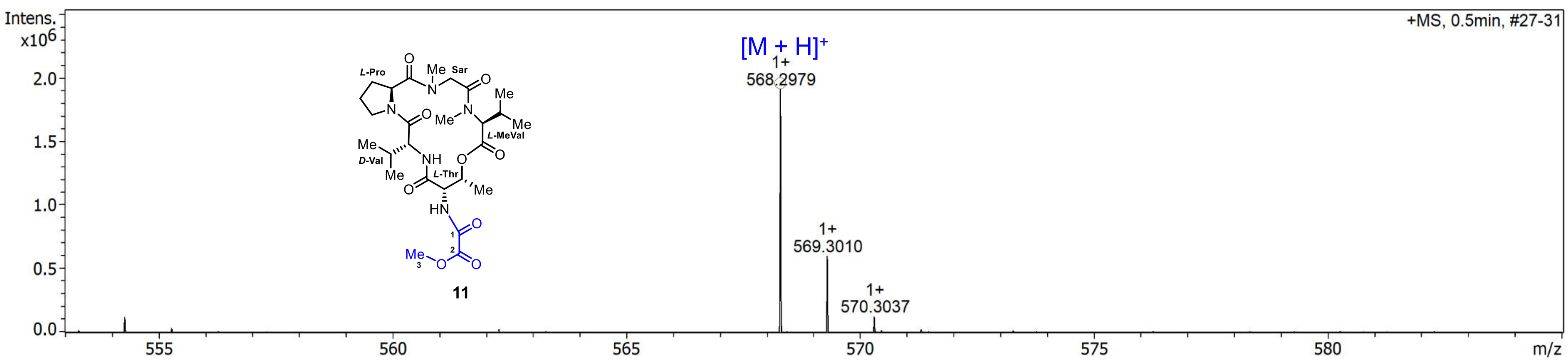

Meas. m/z	#	Ion Formula	Score	m/z	err [mDa]	err [ppm]	mSigma	rdb (neutral)	e⁻ Conf	Adduct
568.297913	1	C26H42N5O9	100.00	568.297704	-0.2	-0.4	2.9	9.0	even	M+H
1135.588138	1	C52H83N10O18	100.00	1135.588132	-0.0	-0.0	20.8	17.0	even	M+H
1152.614982	1	C52H86N11O18	100.00	1152.614681	-0.3	-0.3	29.3	16.0	even	M+H
1157.570233	1	C52H82N10NaO18	100.00	1157.570077	-0.2	-0.1	27.9	17.0	even	M+H
1157.570233	2	C54H81N10O18	23.96	1157.572482	2.2	1.9	34.0	20.0	even	M+H

Figure S109. ^{1}H NMR (600 MHz) spectrum of compound **11** in DMSO-d_6

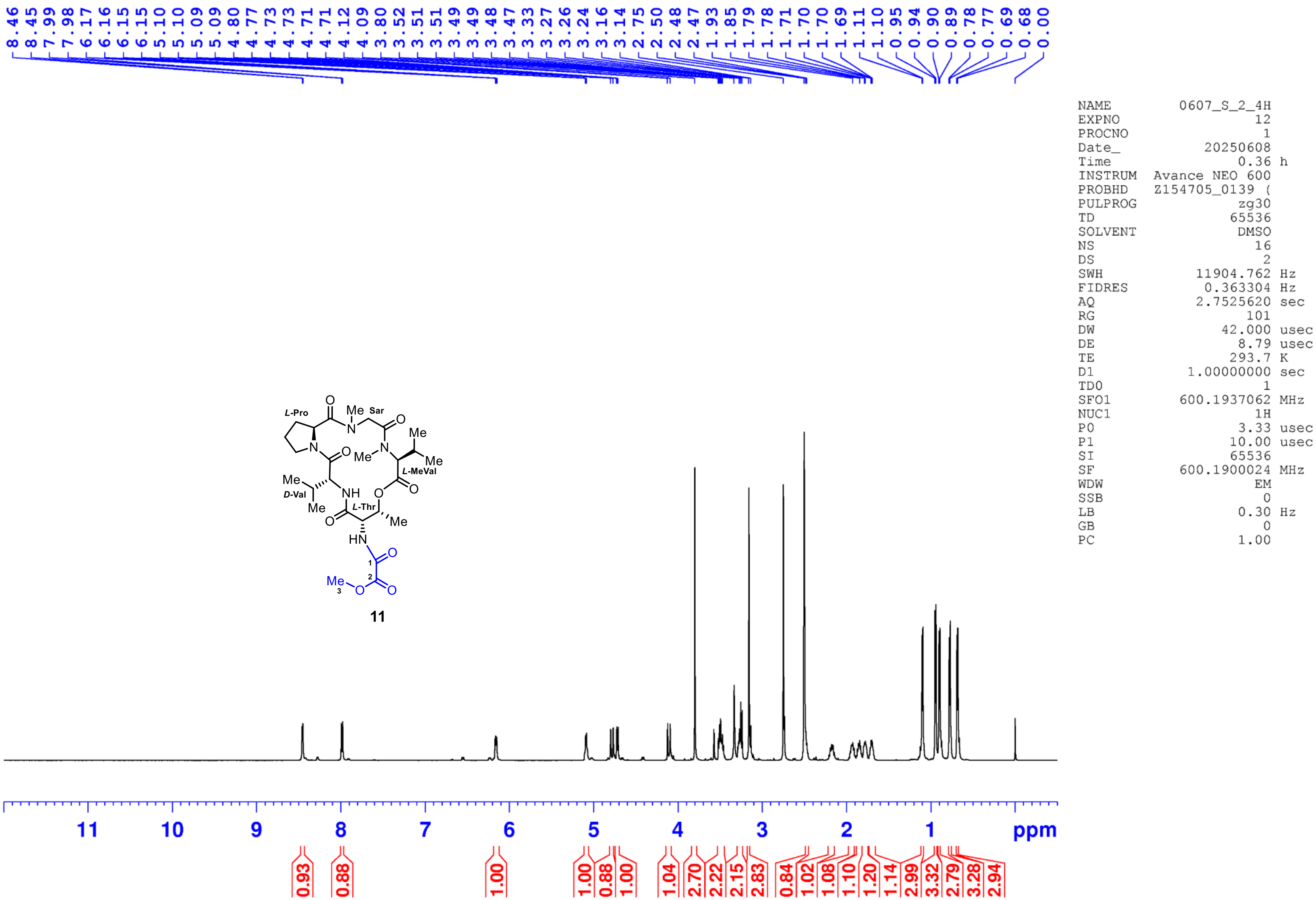

Figure S109-continue

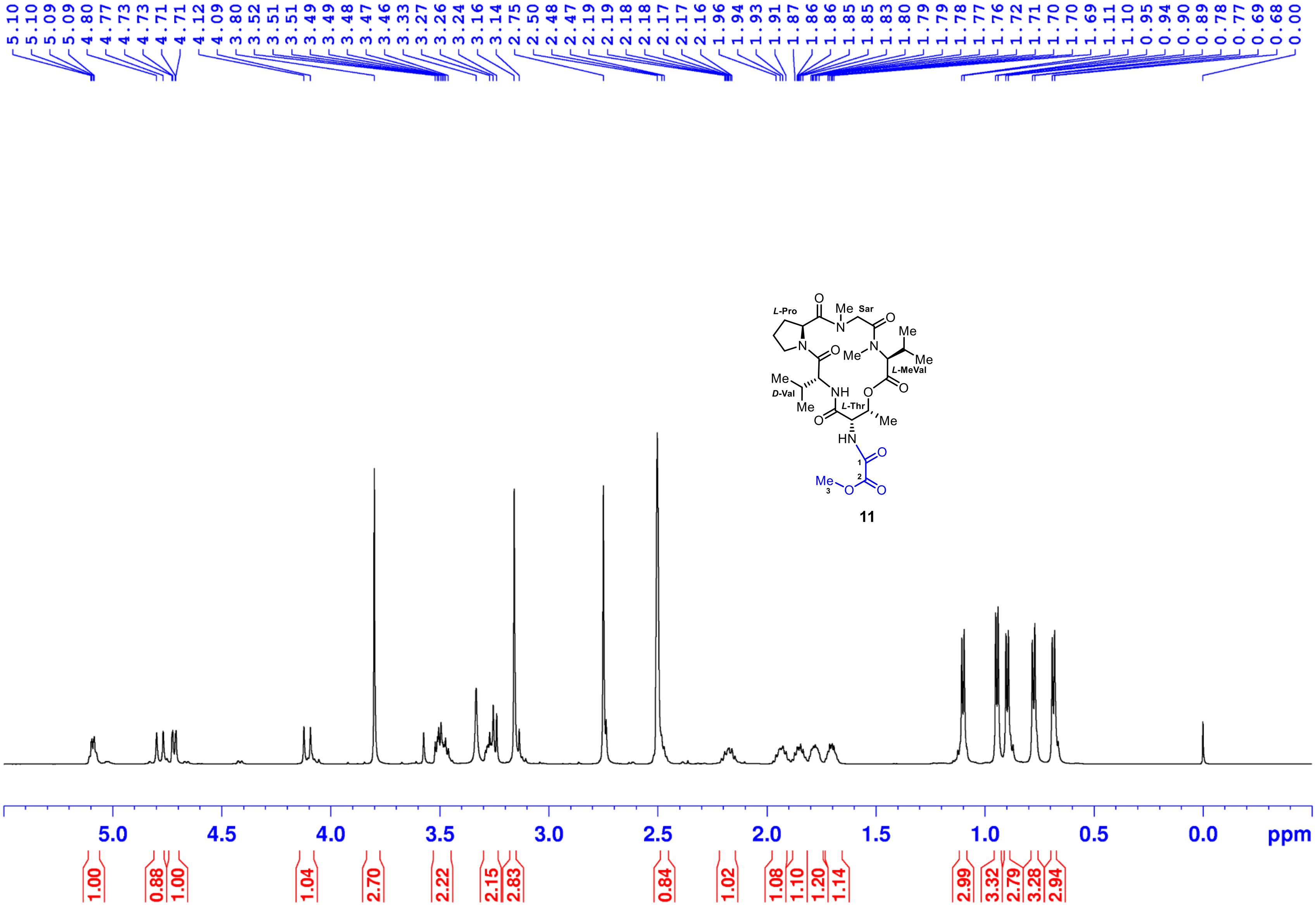

Figure S109-continue

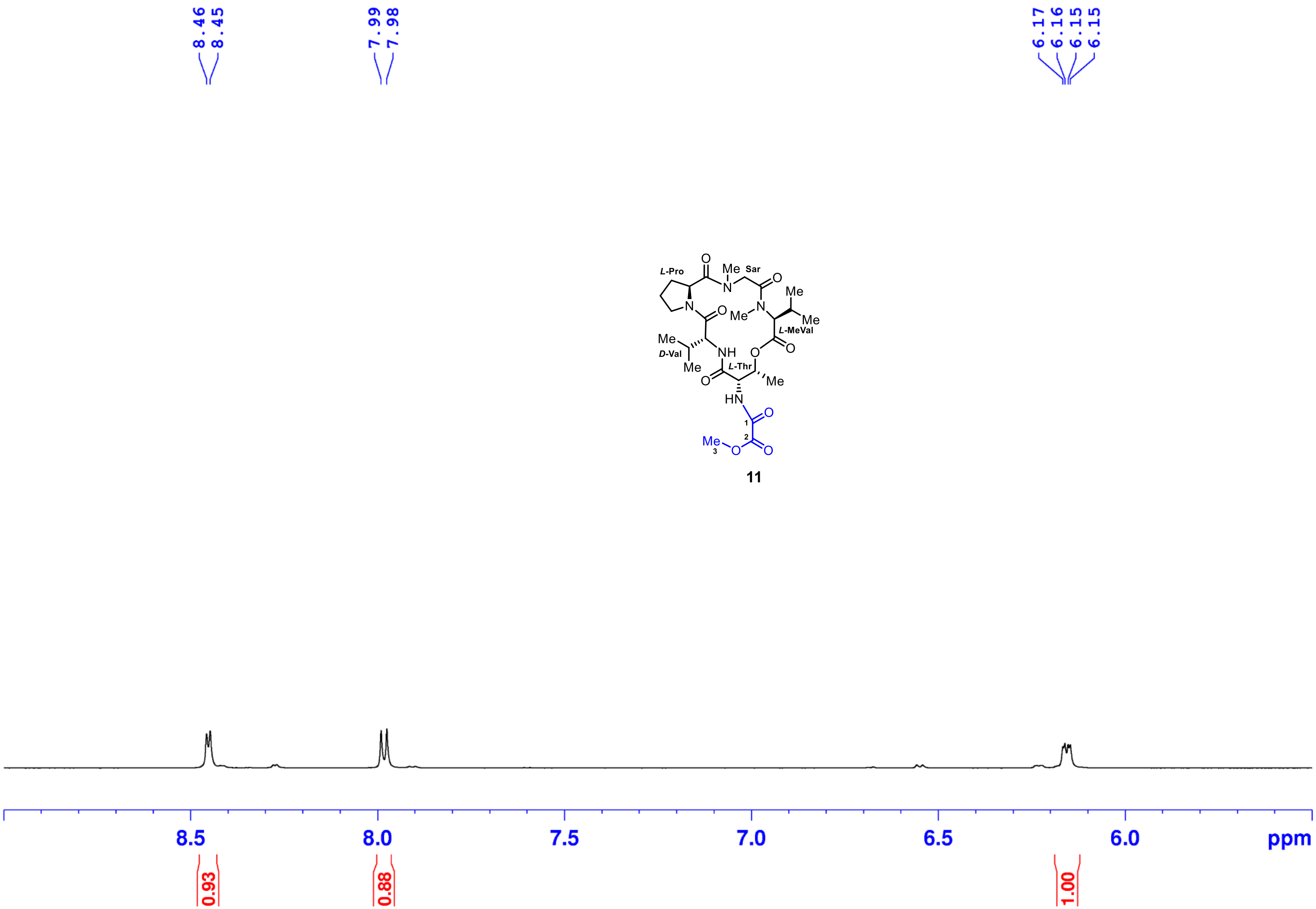

Figure S110. ^{13}C NMR (150 MHz) spectrum of compound **11** in DMSO-d_6

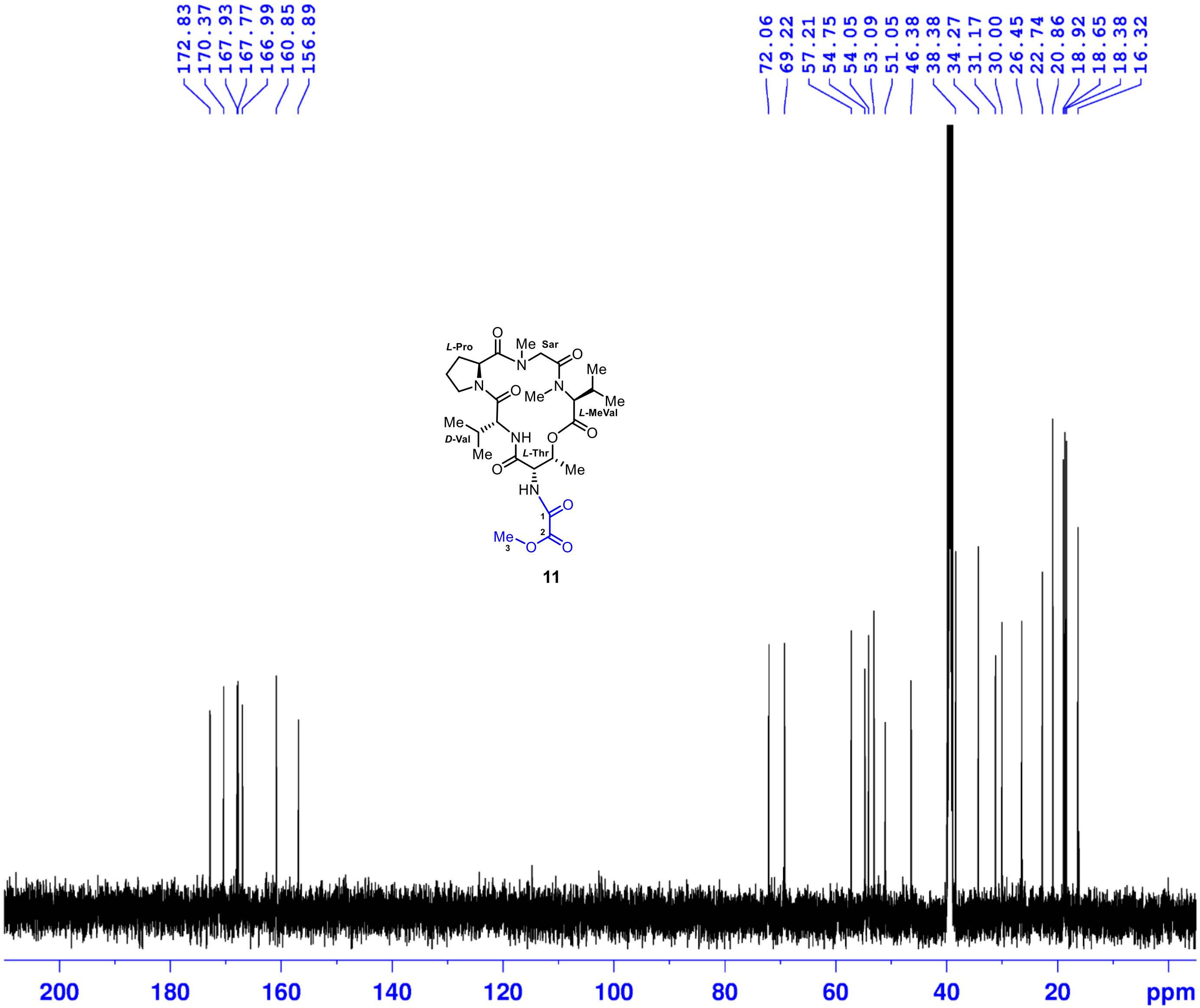

NAME	0607_S_2_4	
EXPNO	10	
PROCNO	1	
Date_	20250608	
Time	0.34	h
INSTRUM	Avance NEO 600	
PROBHD	Z154705_0139 (	
PULPROG	zgpg30	
TD	35714	
SOLVENT	DMSO	
NS	512	
DS	4	
SWH	35714.285	Hz
FIDRES	2.000016	Hz
AQ	0.5000460	sec
RG	101	
DW	14.000	usec
DE	6.50	usec
TE	294.5	K
D1	1.00000000	sec
D11	0.03000000	sec
TD0	1	
SFO1	150.9329873	MHz
NUC1	13C	
P0	4.00	usec
P1	12.00	usec
SI	32768	
SF	150.9179828	MHz
WDW	EM	
SSB	0	
LB	1.00	Hz
GB	0	
PC	1.40	

Figure S110-continue

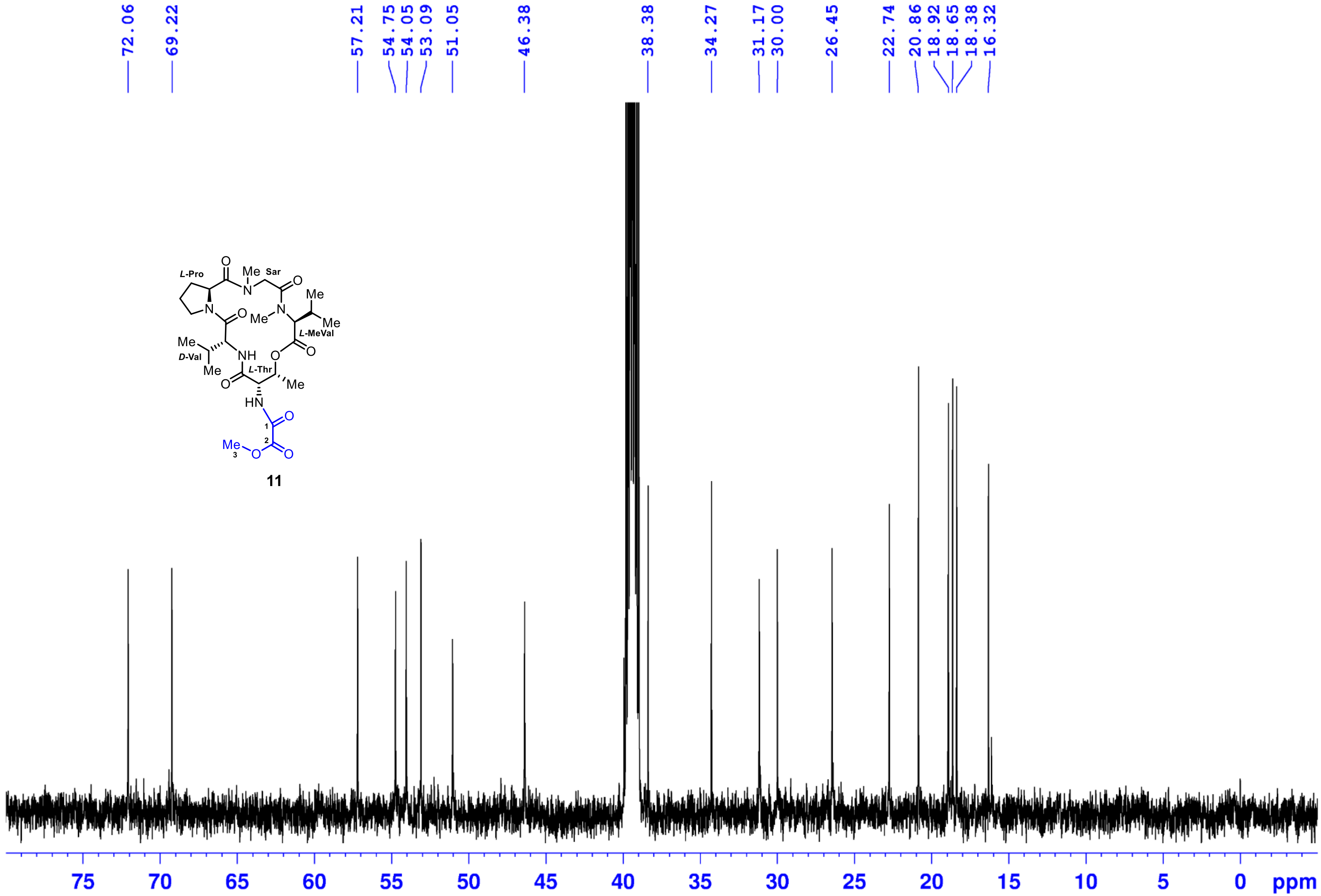

Figure S110-continue

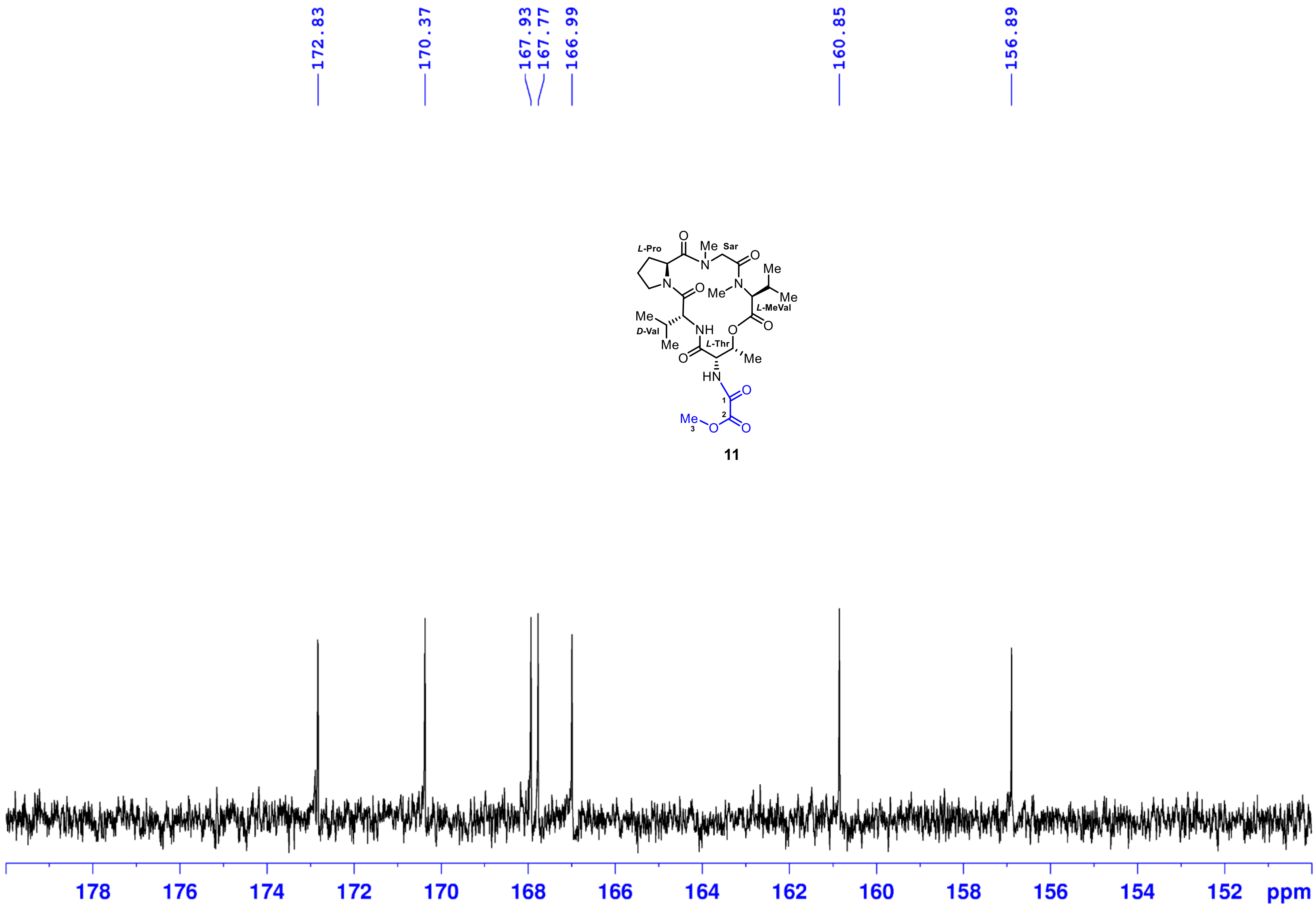

Figure S111. DEPT135 (150 MHz) spectrum of compound **11** in DMSO-d_6

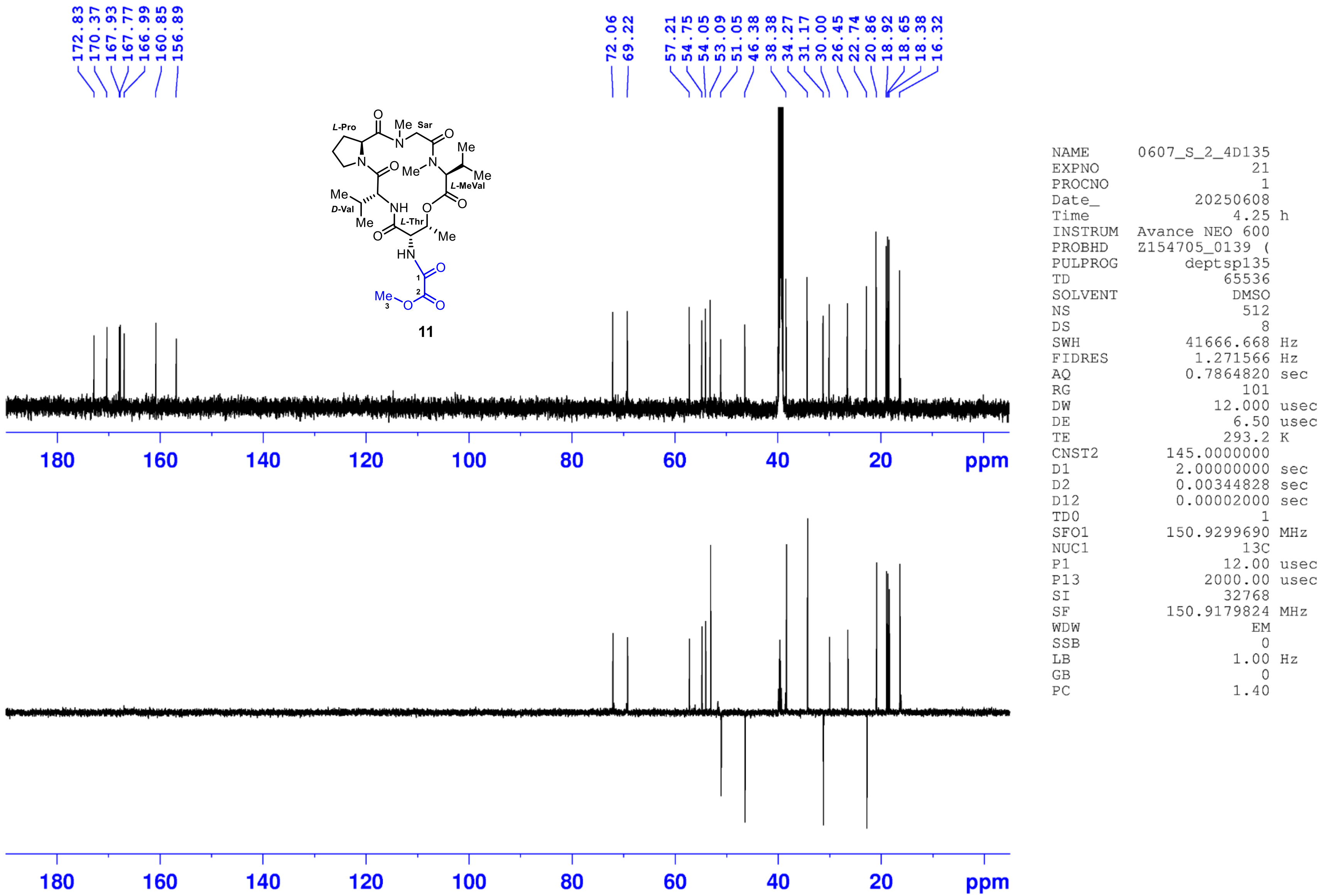

Figure S111-continue

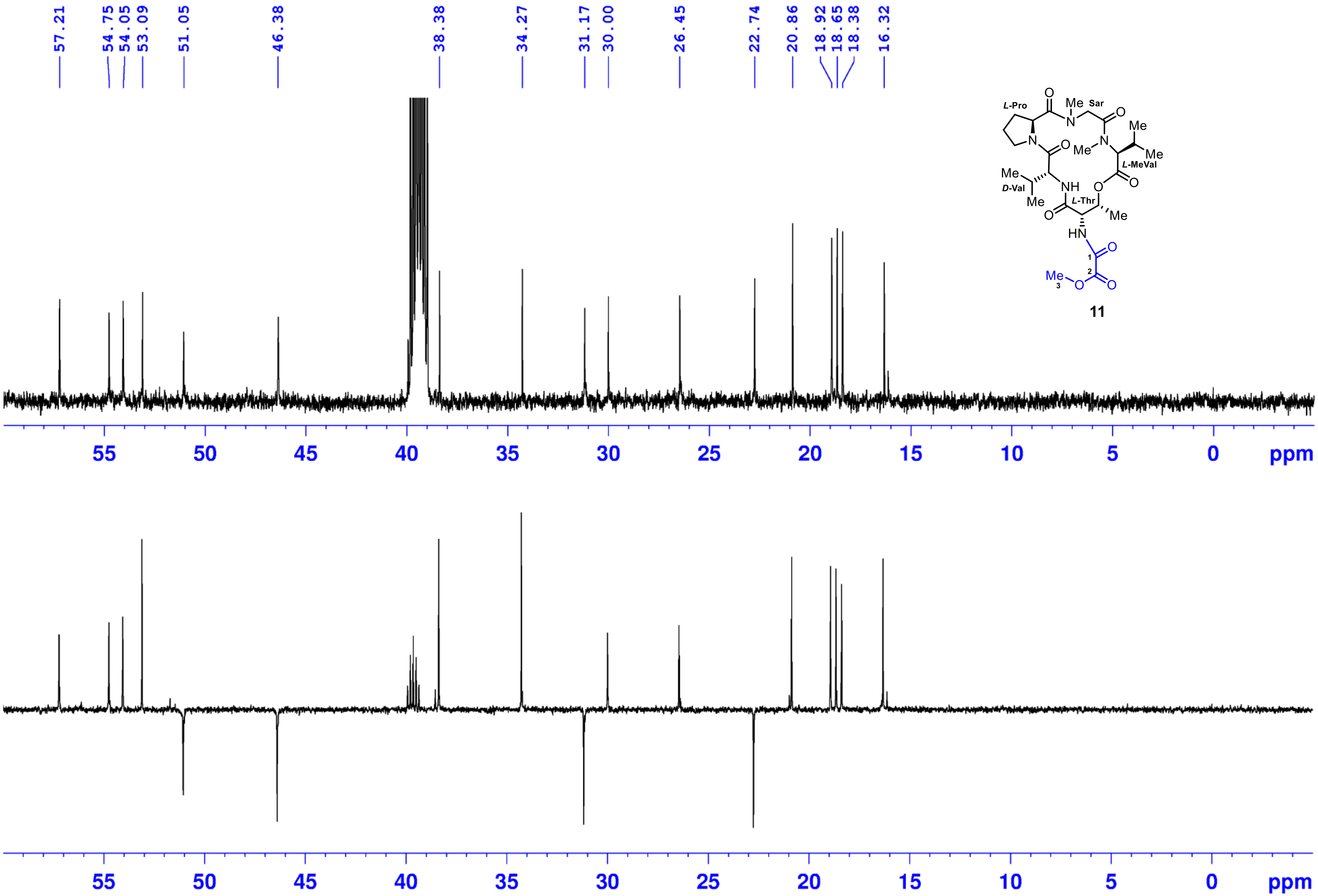

Figure S111-continue

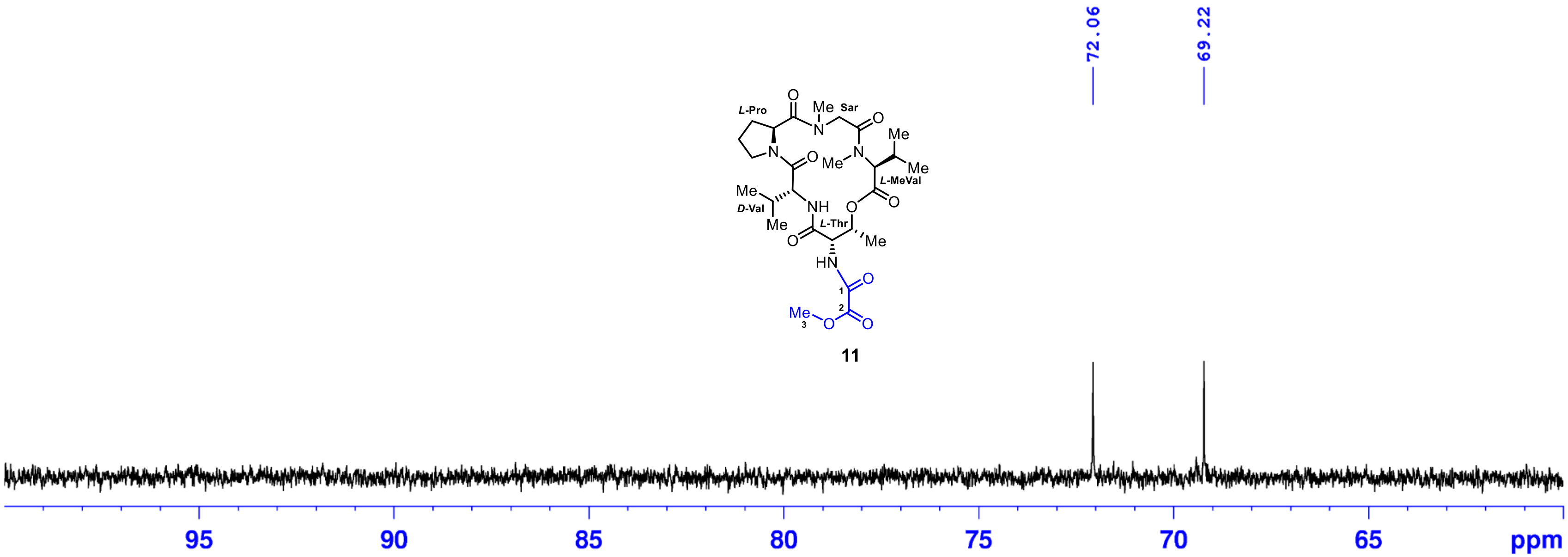

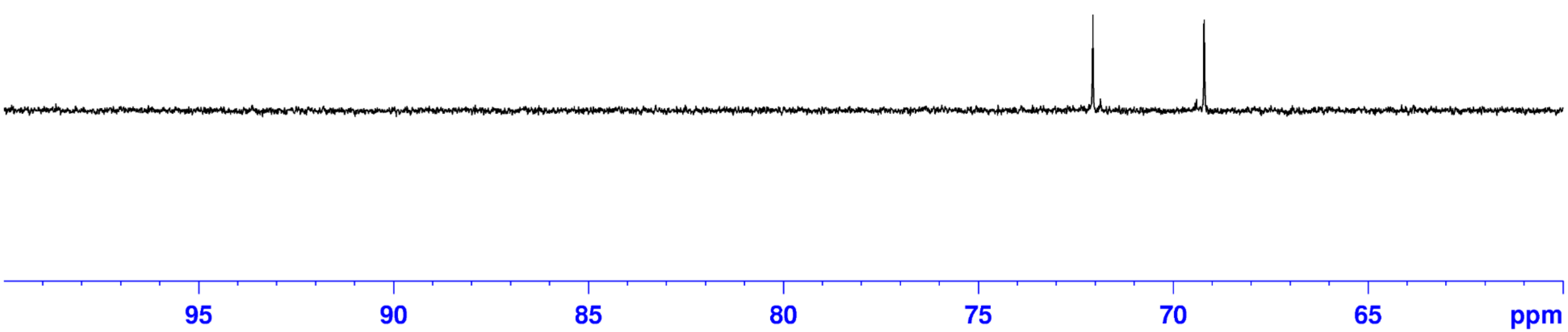

Figure S112. ^{1}H–^{1}H COSY (600 MHz) spectrum of compound **11** in DMSO-d_6

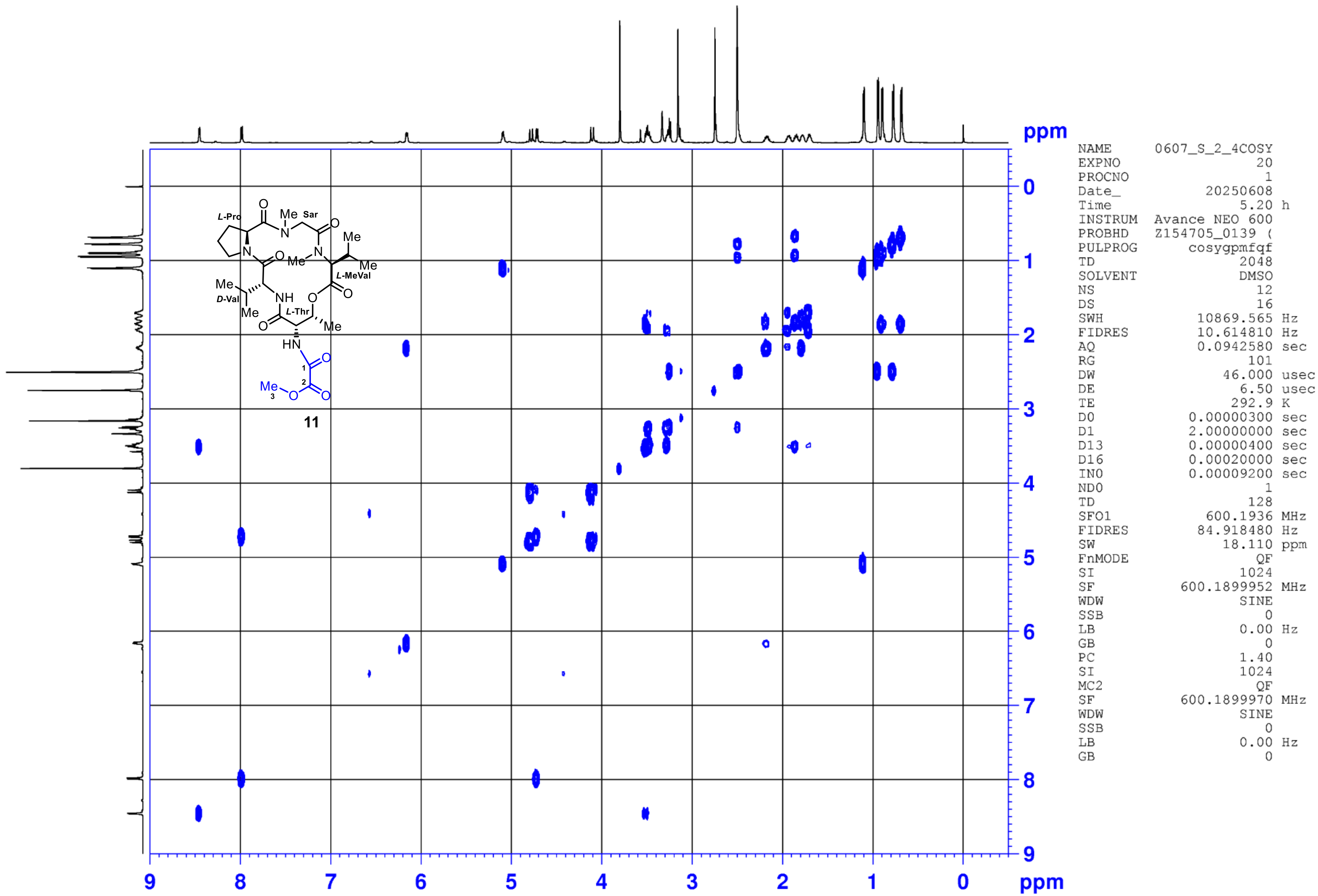

Figure S112-continue

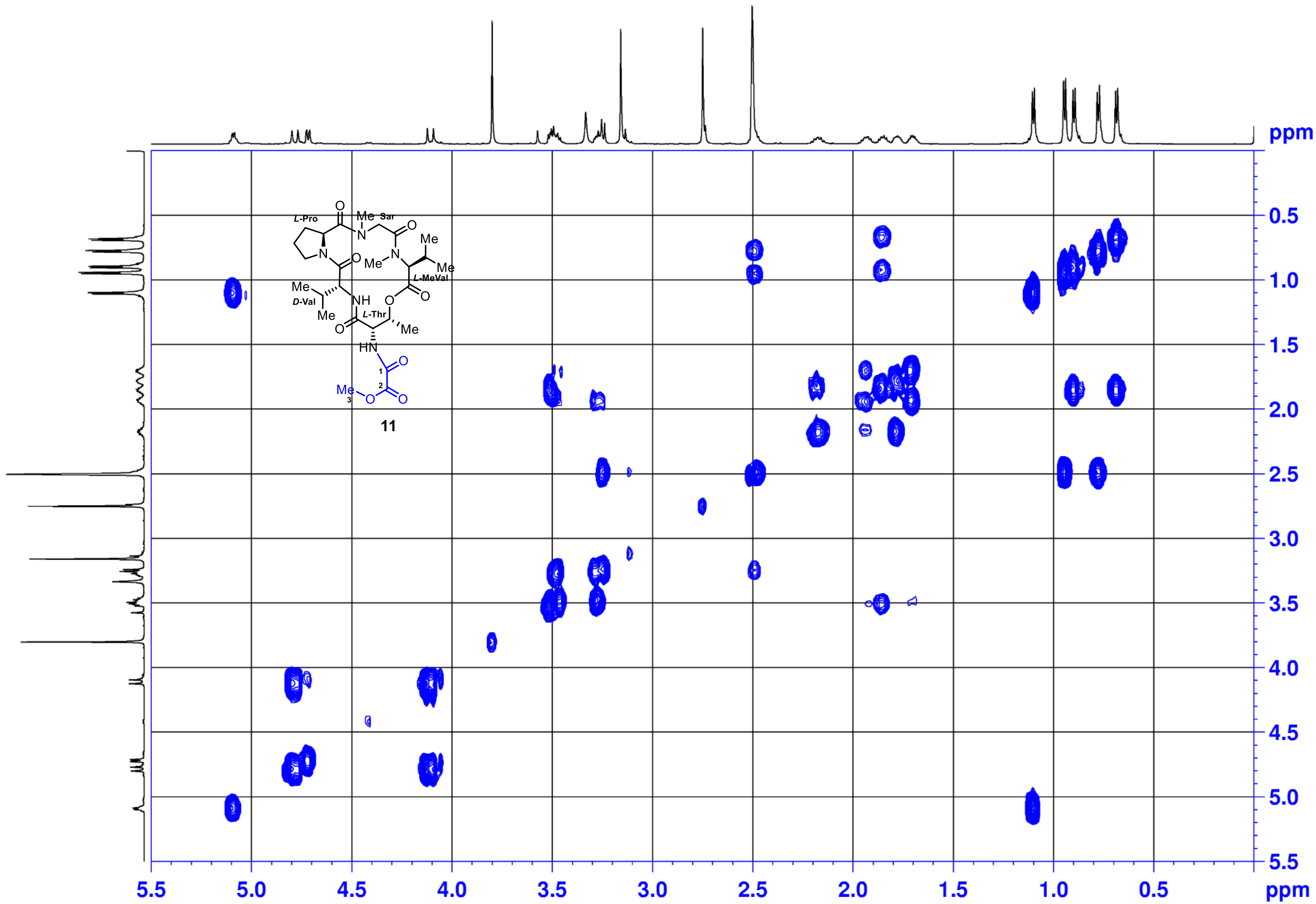

Figure S112-continue

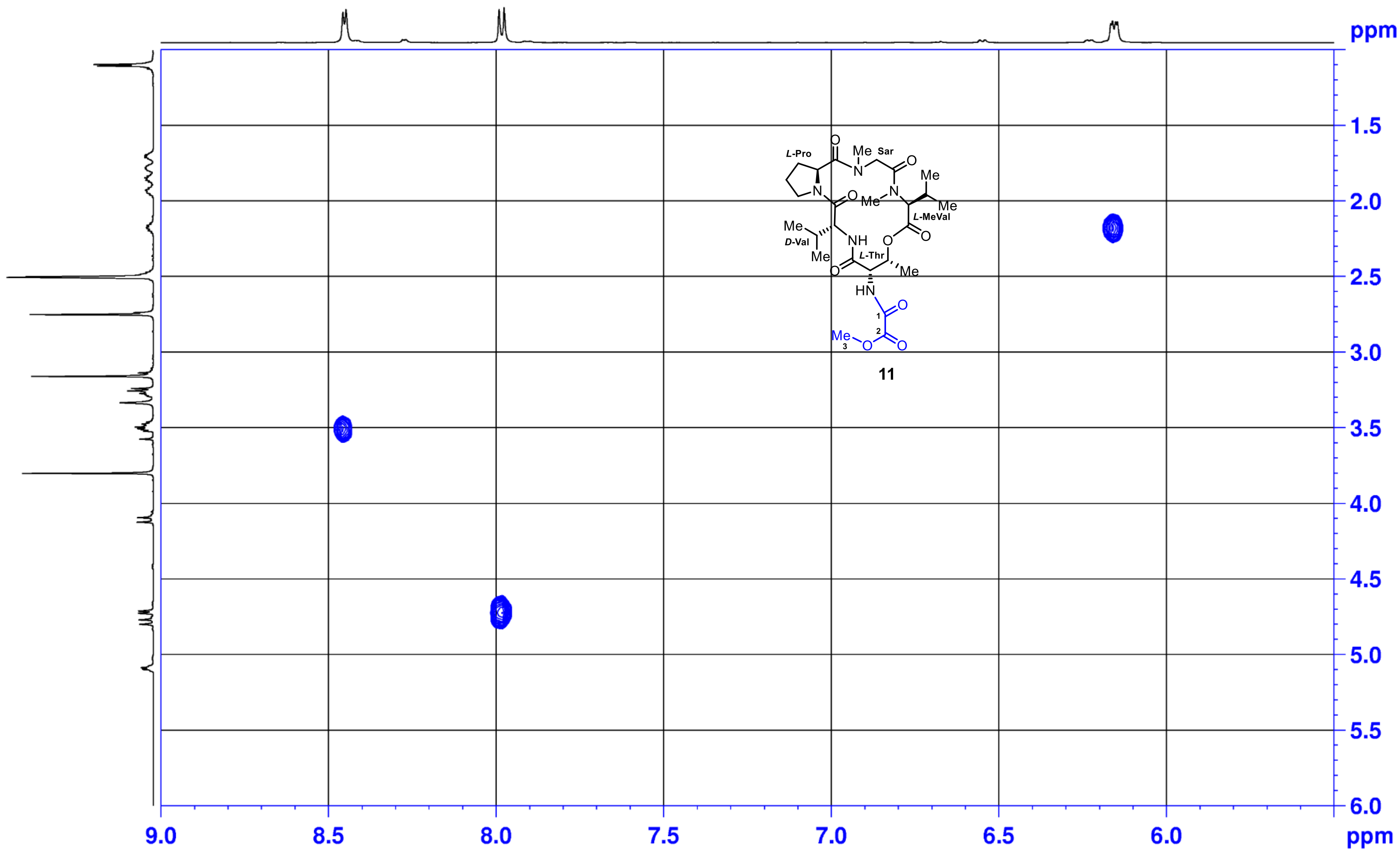

Figure S113. HSQC (600 MHz) spectrum of compound **11** in DMSO-d_6

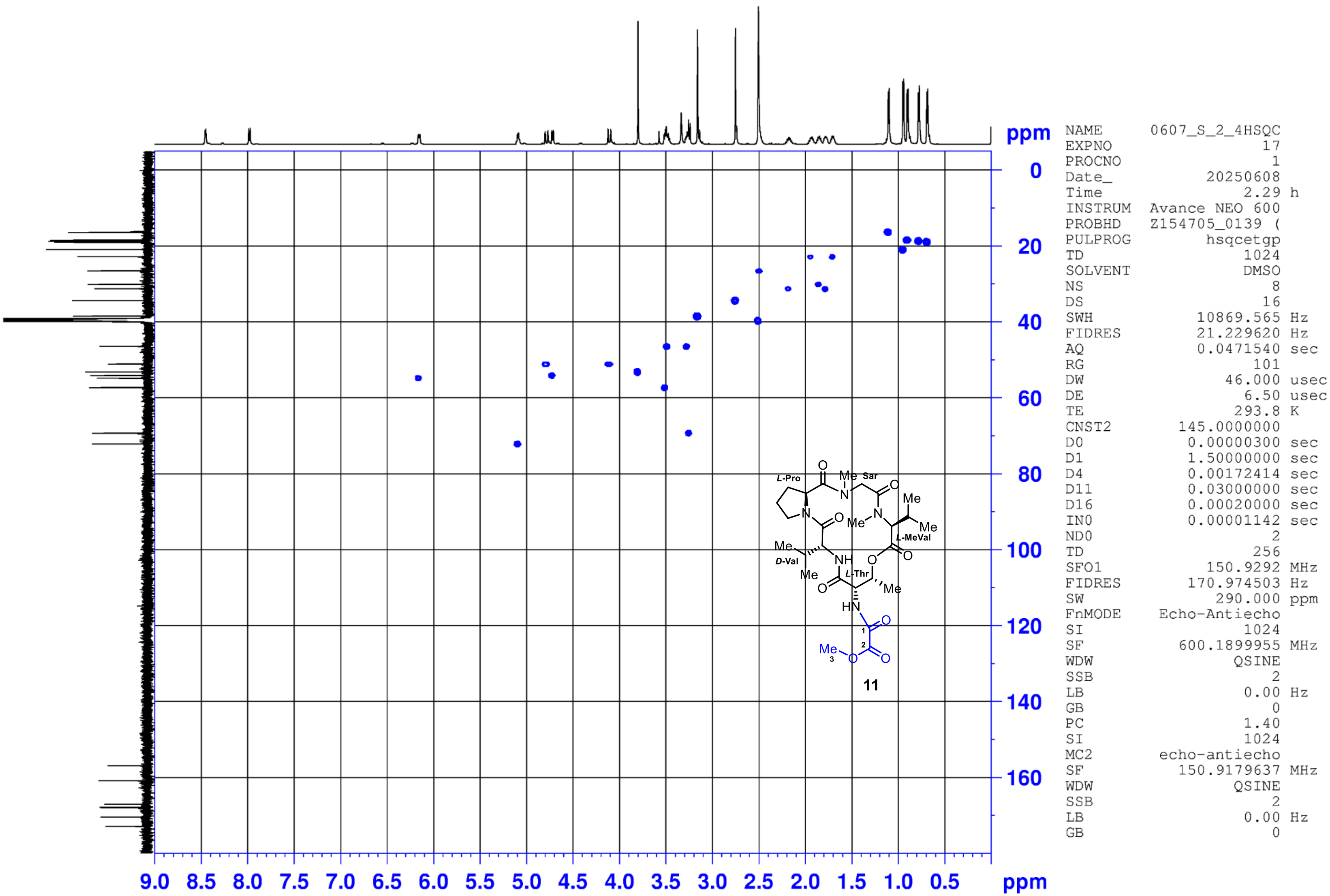

Figure S113-continue

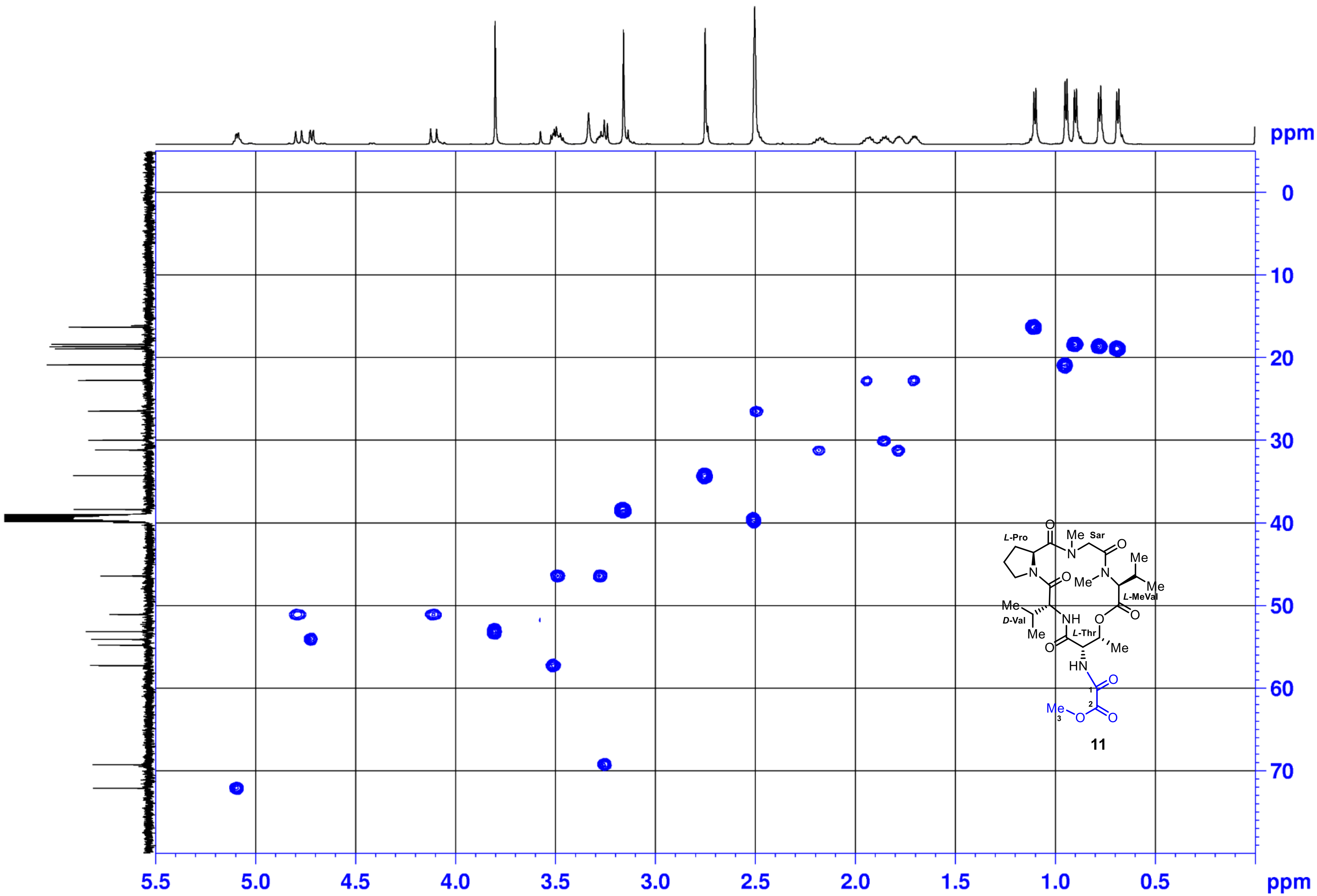

Figure S113-continue

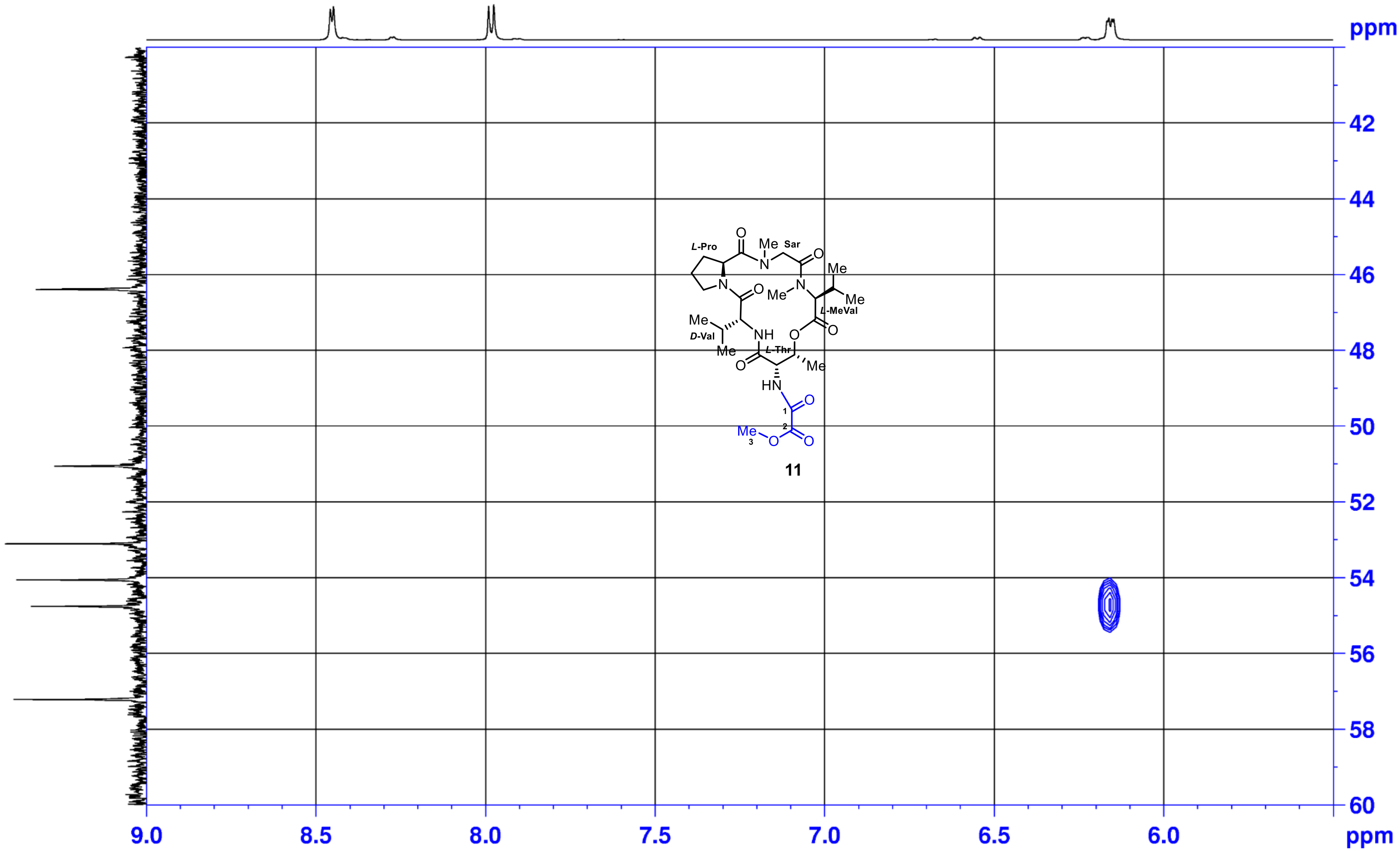

Figure S114. HMBC (600 MHz) spectrum of compound **11** in DMSO-d_6

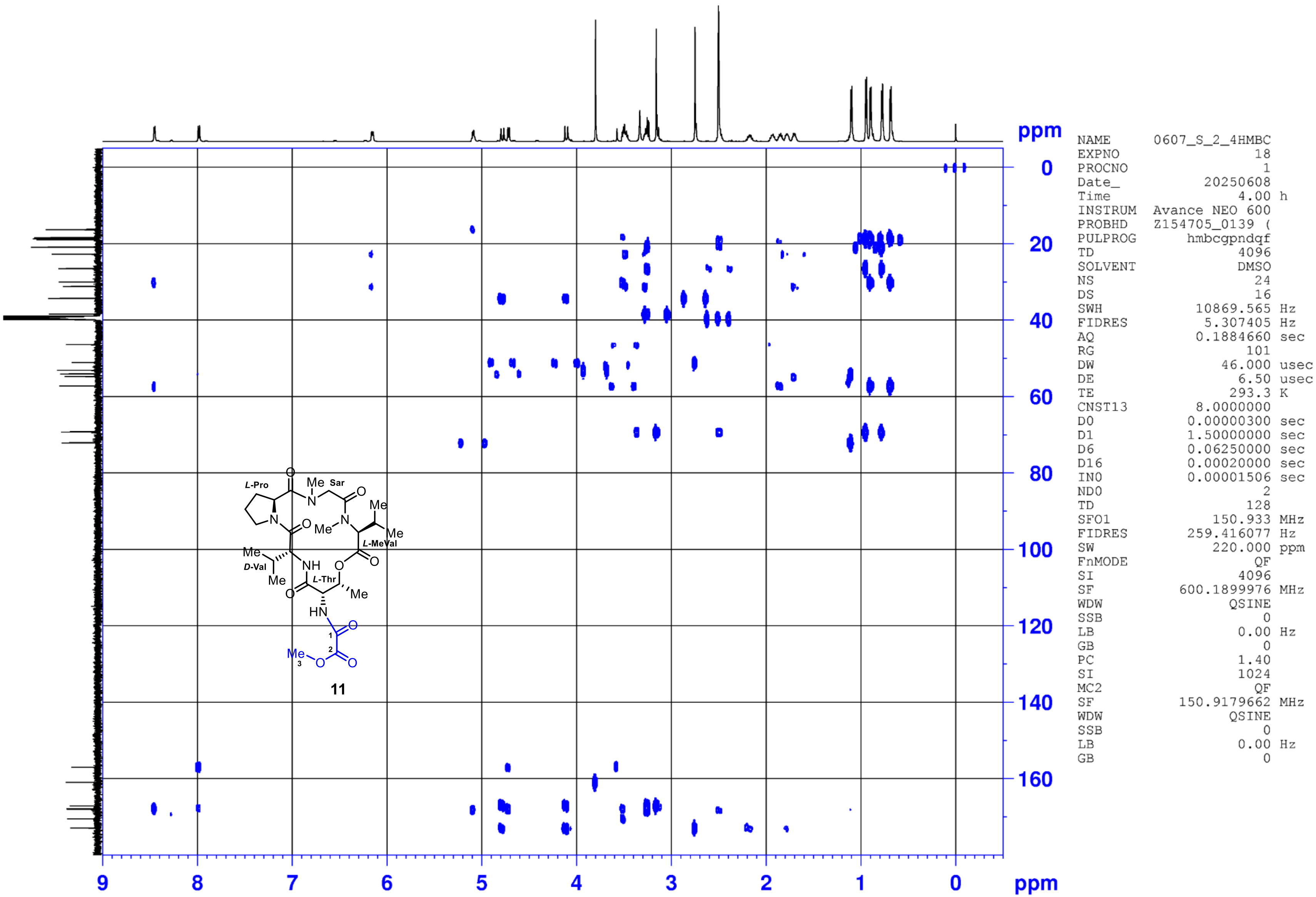

Figure S114-continue

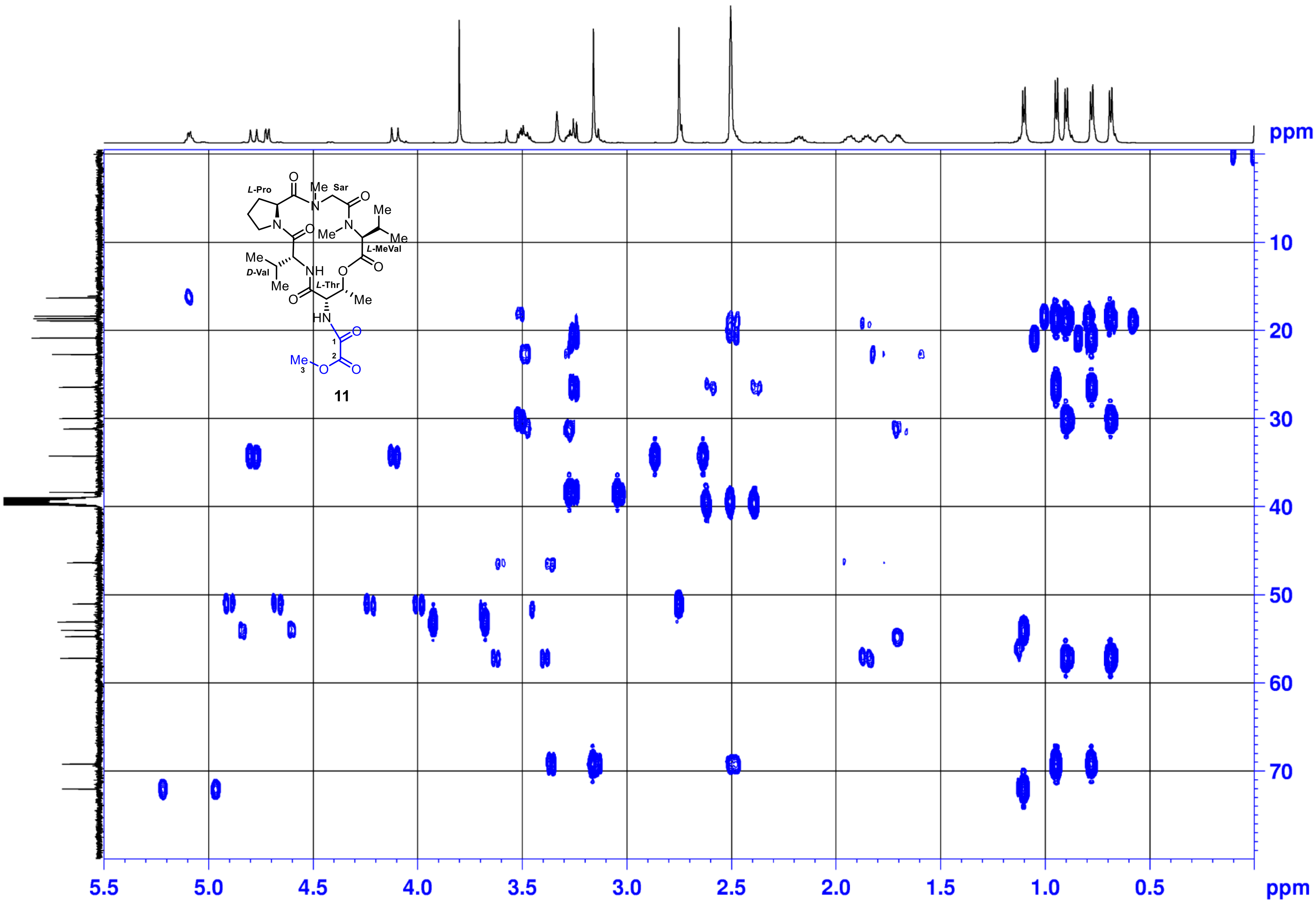

Figure S114-continue

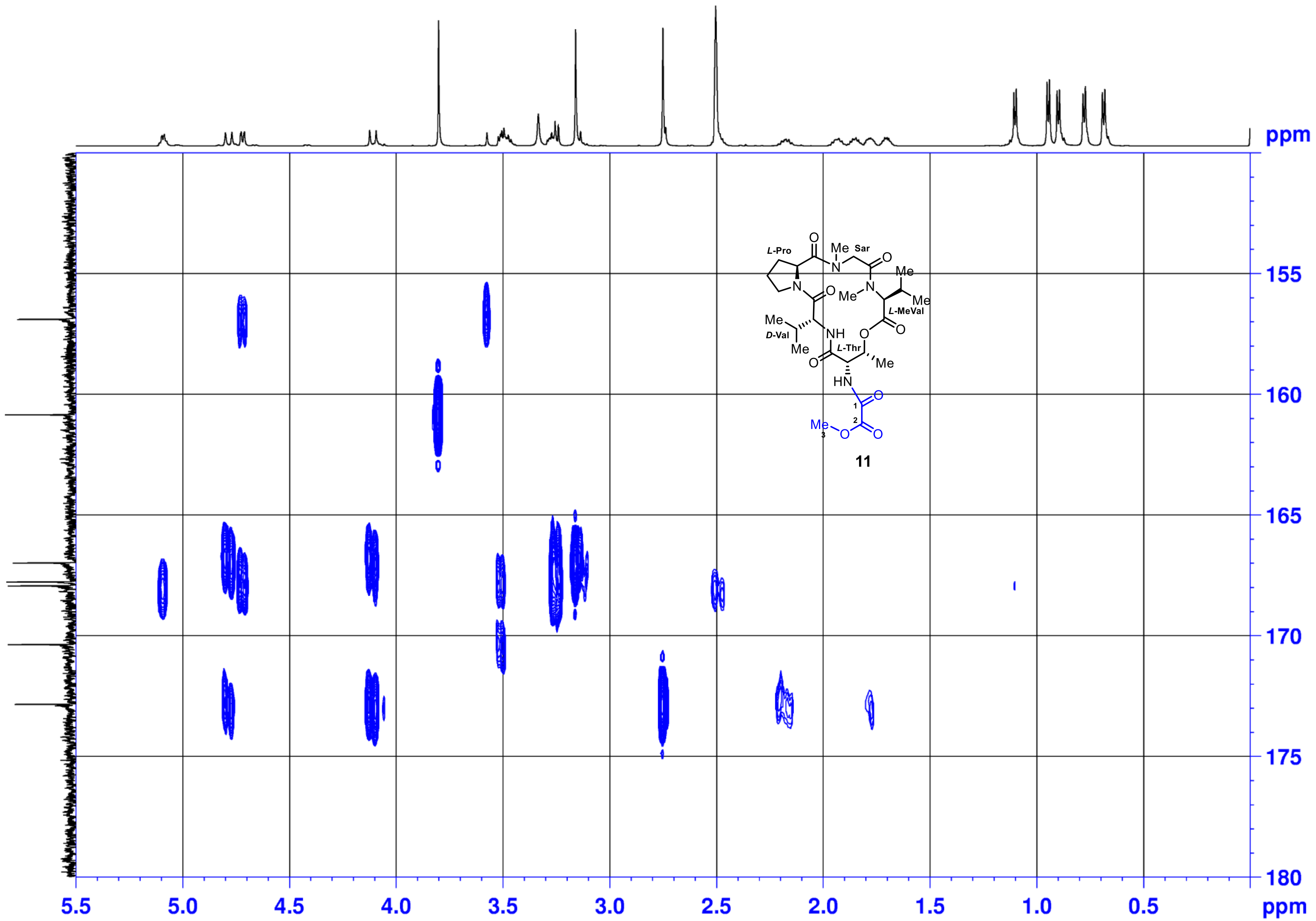

Figure S114-continue

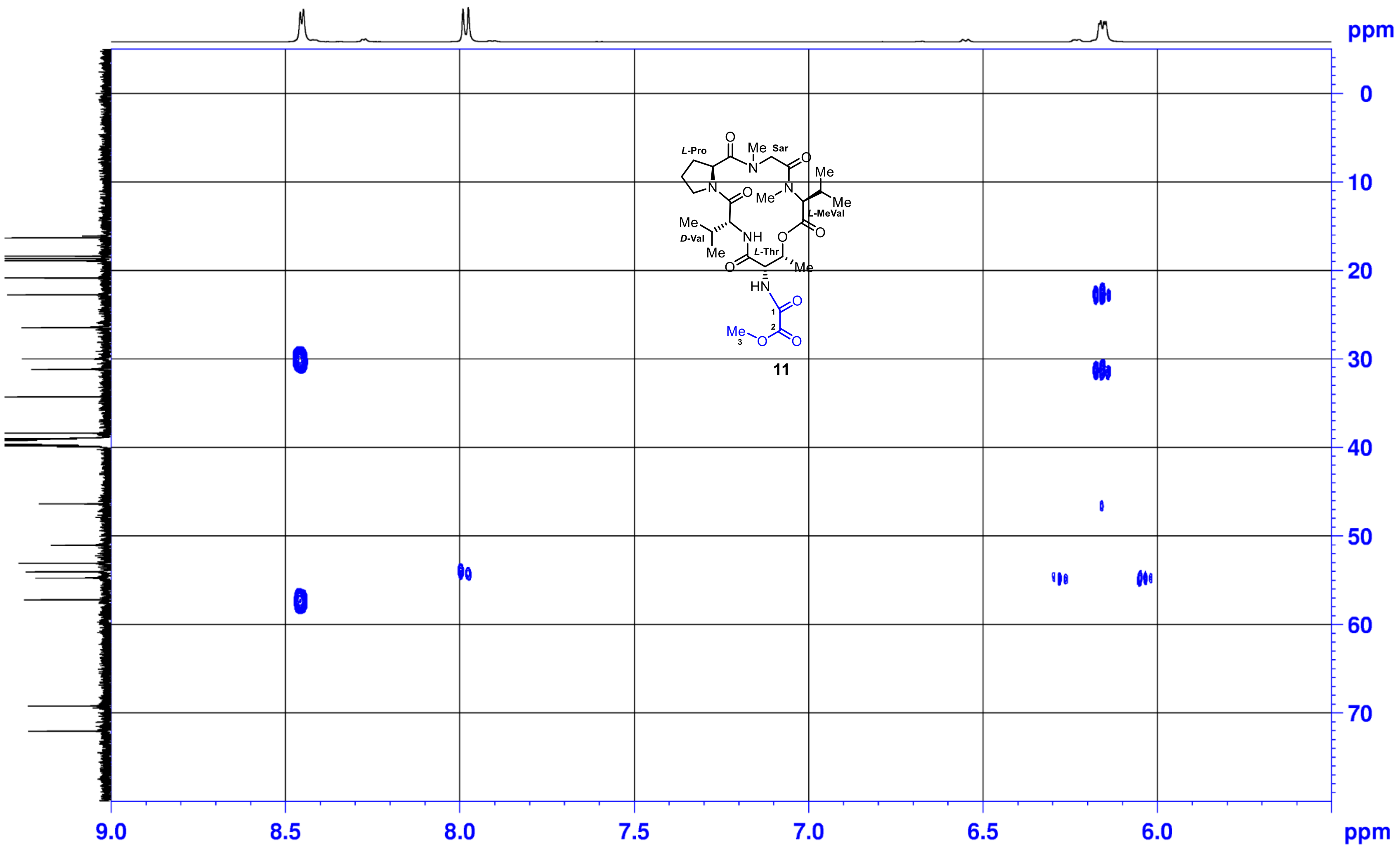

Figure S114-continue

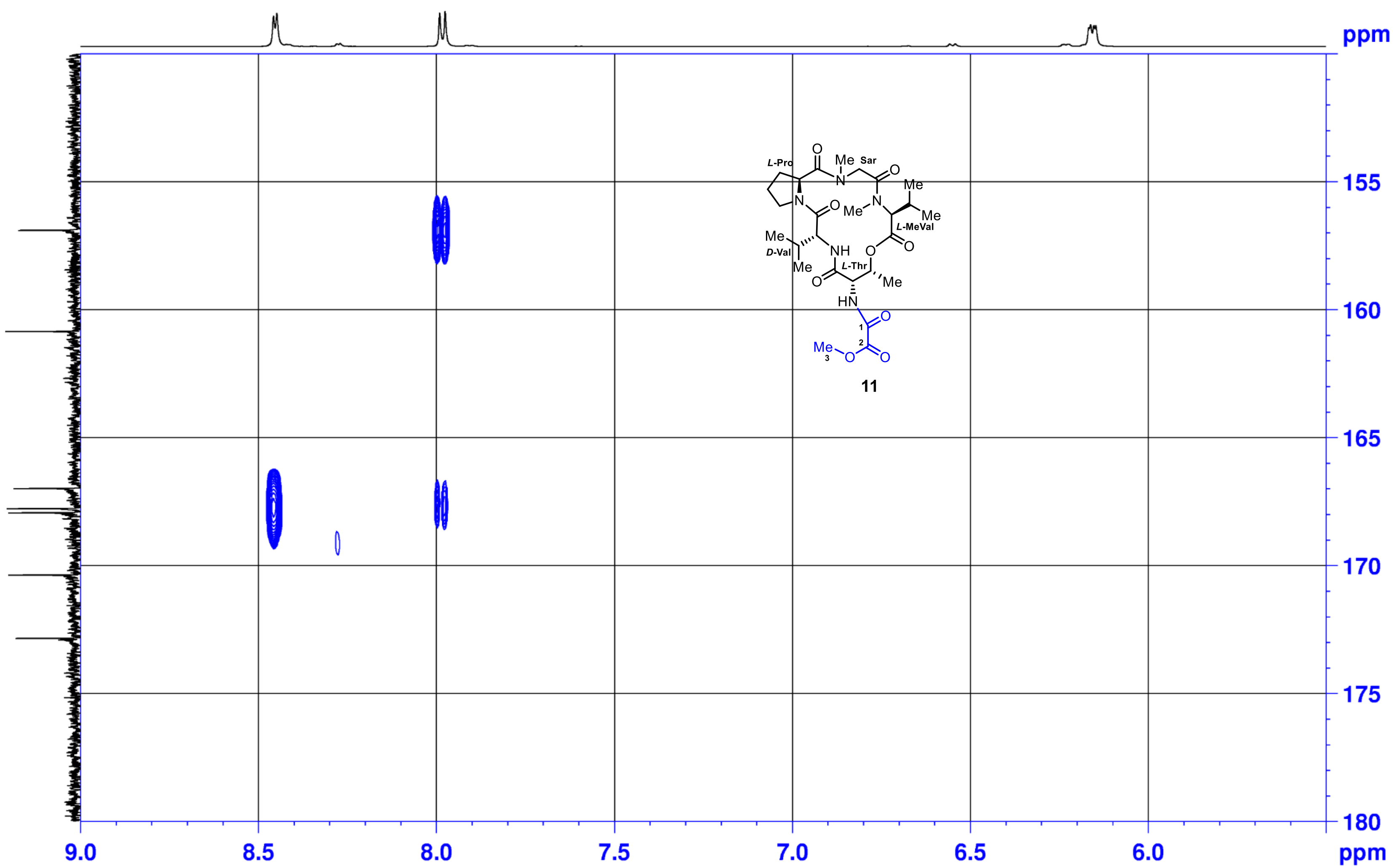

Figure S115. ROESY (600 MHz) spectrum of compound **11** in DMSO-d_6

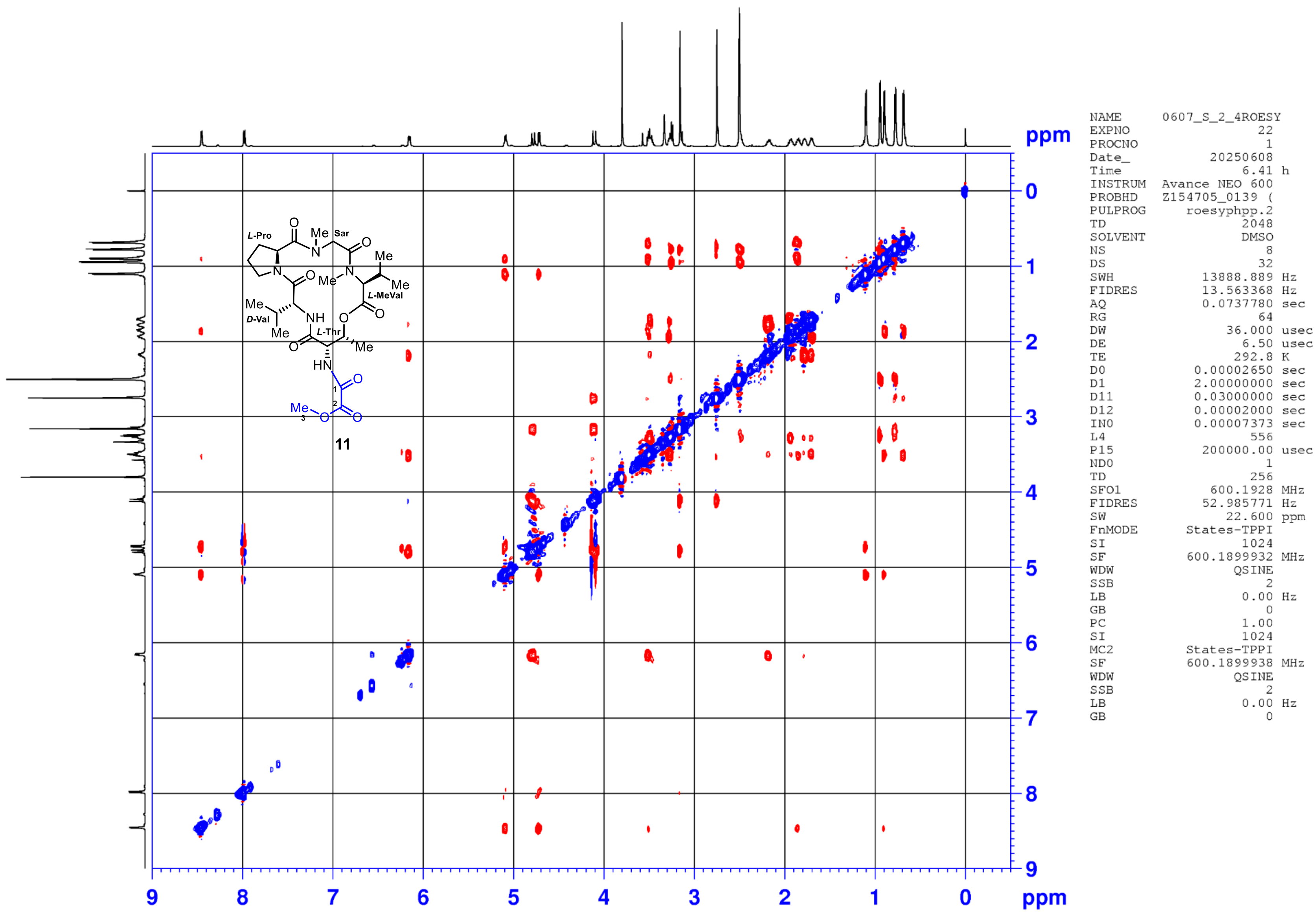

Figure S115-continue

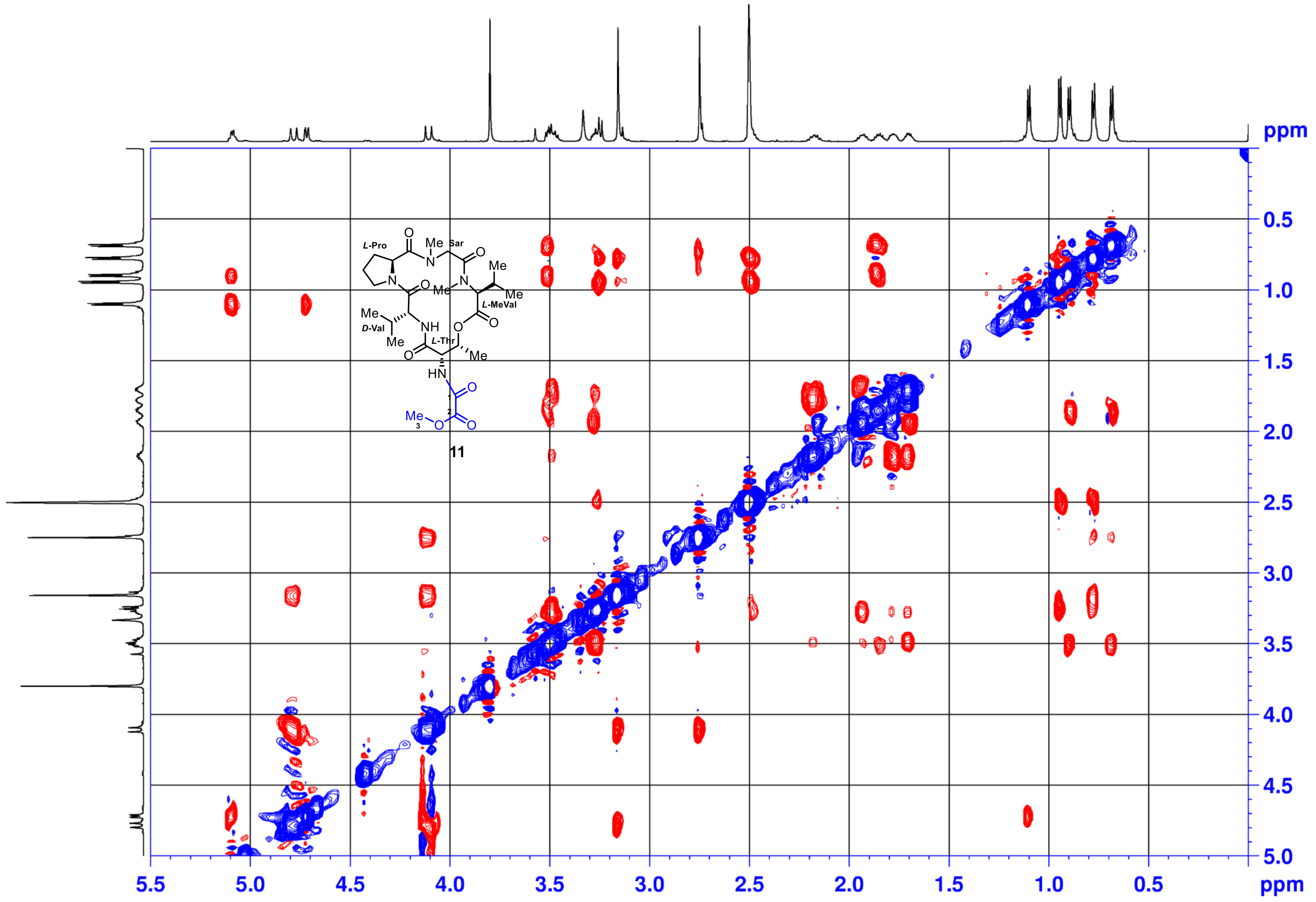

Figure S115-continue

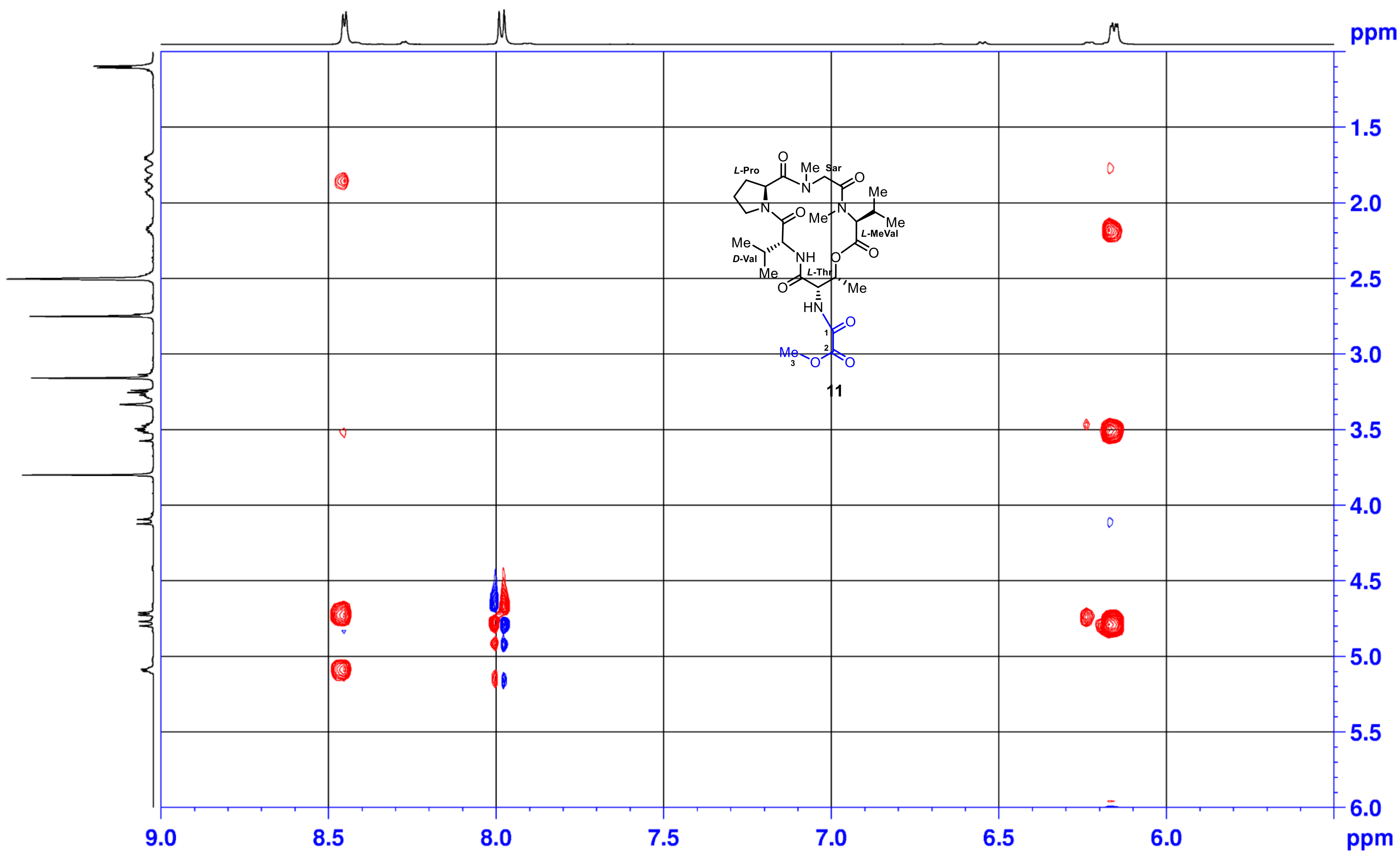

Figure S116. Marfey's analysis of compound **11** and standard amino acids

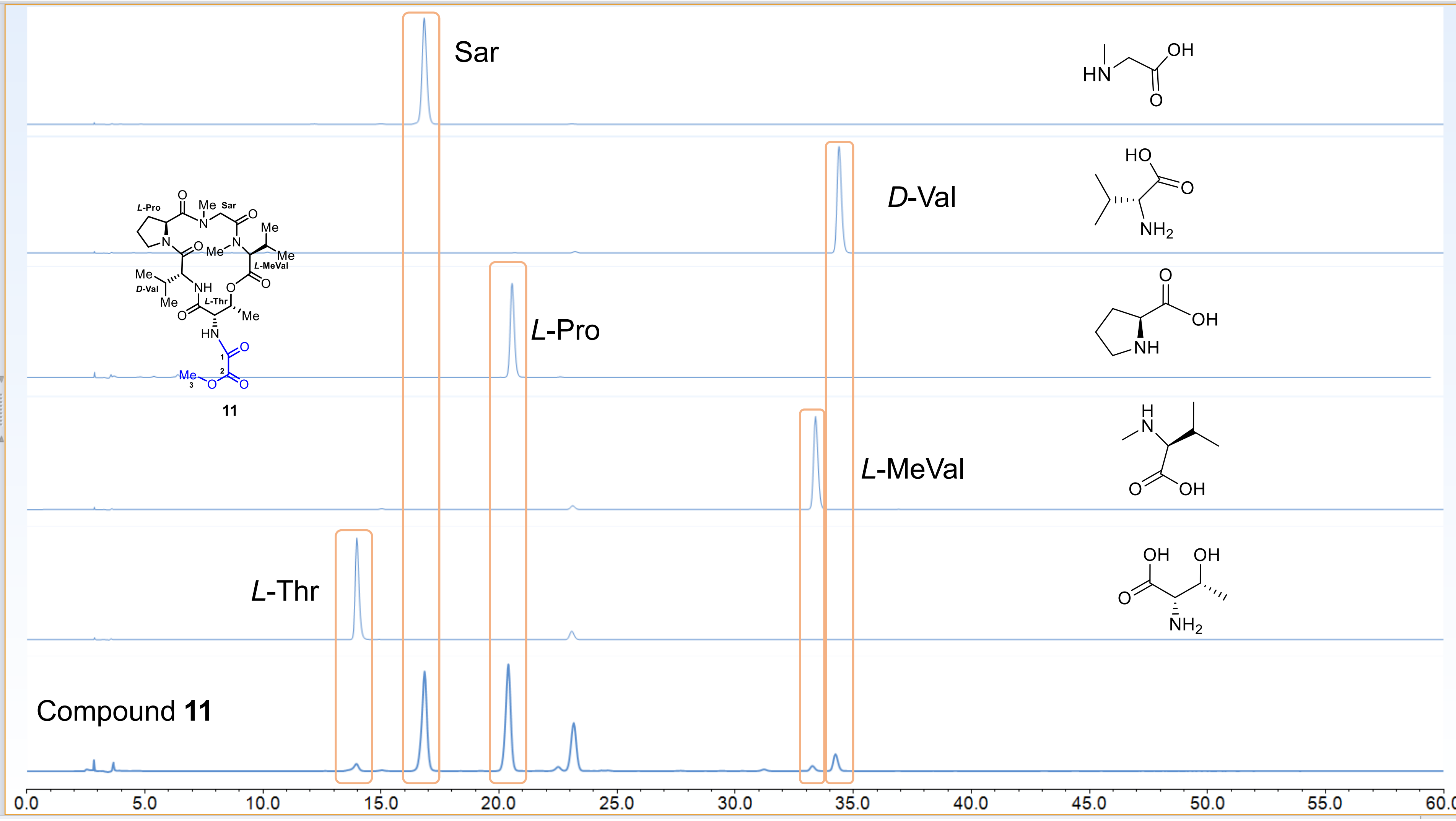

Figure S117. UV spectrum of compound **12**

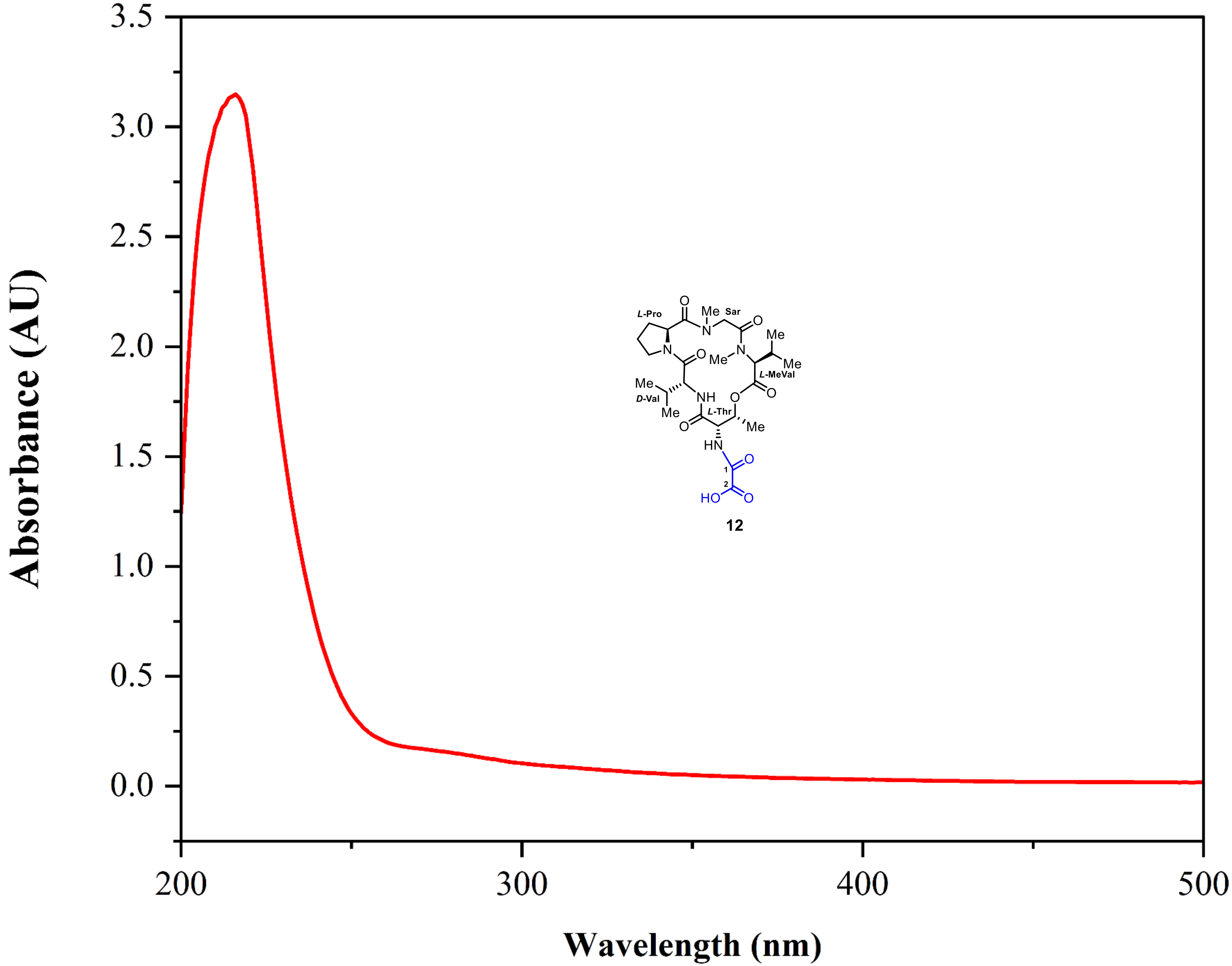

Figure S118. HR-ESIMS for compound **12**

Mass Spectrum SmartFormula Report

Analysis Info

Analysis Name	D:\Data\MS\data\202505\panghe_S-1-0_pos_46_01_19985.d	Acquisition Date	5/29/2025 6:05:46 PM	
Method	LC_Direct Infusion_pos_100-1000mz.m	Operator	SCSIO	
Sample Name	panghe_S-1-0_pos	Instrument	maXis	255552.00029
Comment				

Acquisition Parameter

Source Type	ESI	Ion Polarity	Positive	Set Nebulizer	0.4 Bar
Focus	Active	Set Capillary	4500 V	Set Dry Heater	180 °C
Scan Begin	100 m/z	Set End Plate Offset	-500 V	Set Dry Gas	4.0 l/min
Scan End	2000 m/z	Set Charging Voltage	0 V	Set Divert Valve	Waste
		Set Corona	0 nA	Set APCI Heater	0 °C

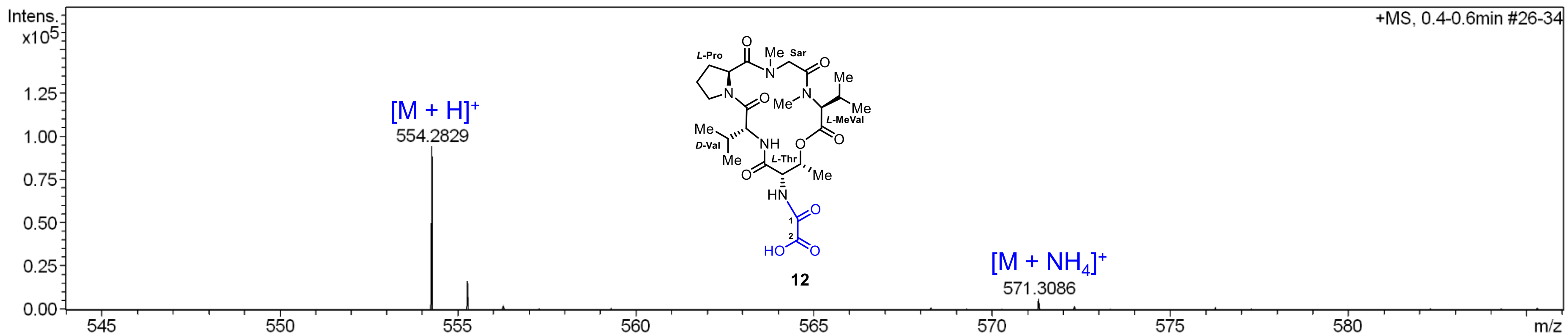

Meas. m/z	#	Ion Formula	Score	m/z	err [ppm]	err [mDa]	mSigma	rdb	e⁻ Conf	N-Rule
554.2829	1	C25H40N5O9	100.00	554.2821	-1.5	-0.9	65.6	8.5	even	ok
571.3086	1	C25H43N6O9	100.00	571.3086	-0.1	-0.0	37.1	7.5	even	ok
576.2637	1	C25H39N5NaO9	100.00	576.2640	0.6	0.3	36.0	8.5	even	ok

Figure S119. ^{1}H NMR (600 MHz) spectrum of compound **12** in DMSO-d_6

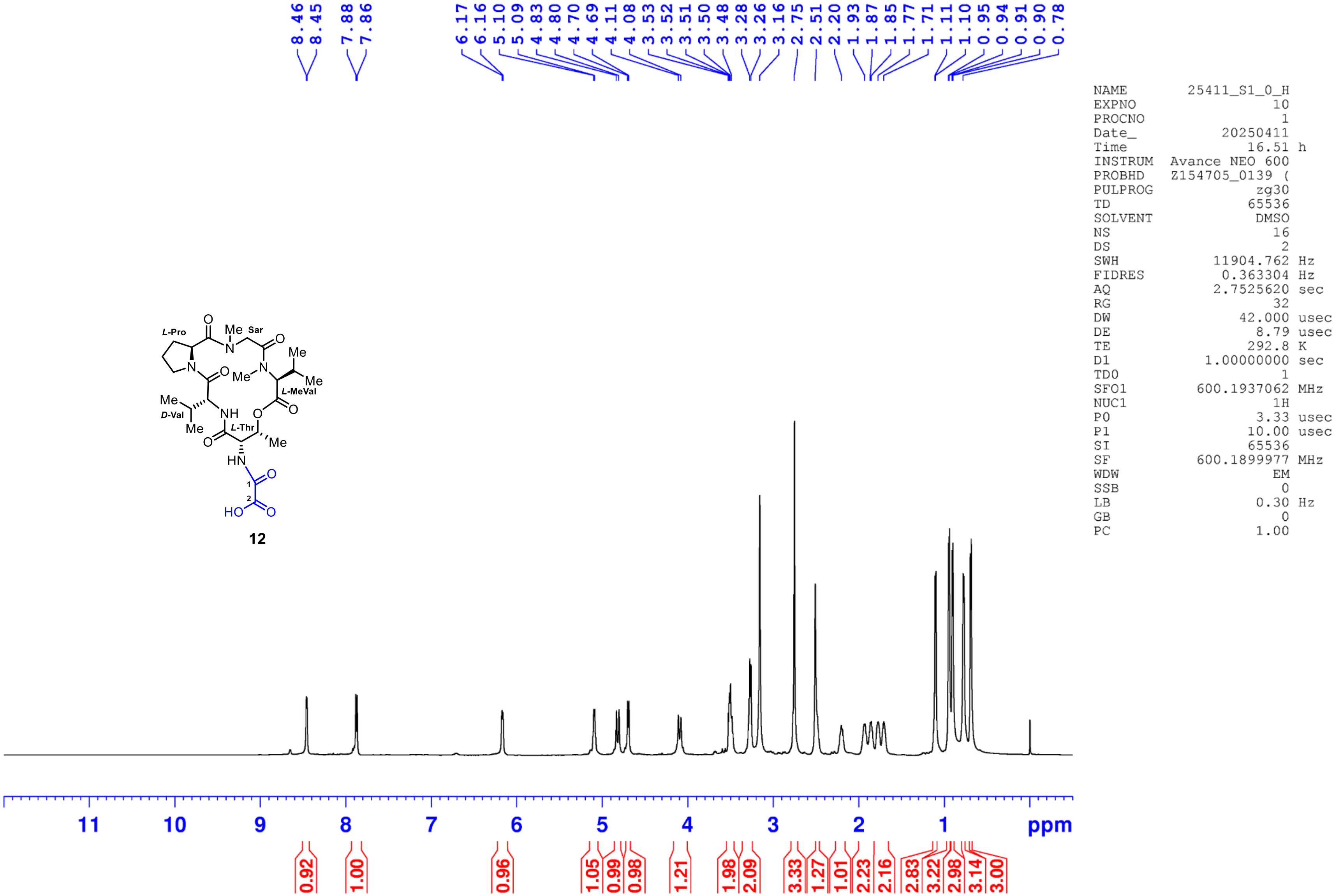

Figure S119-continue

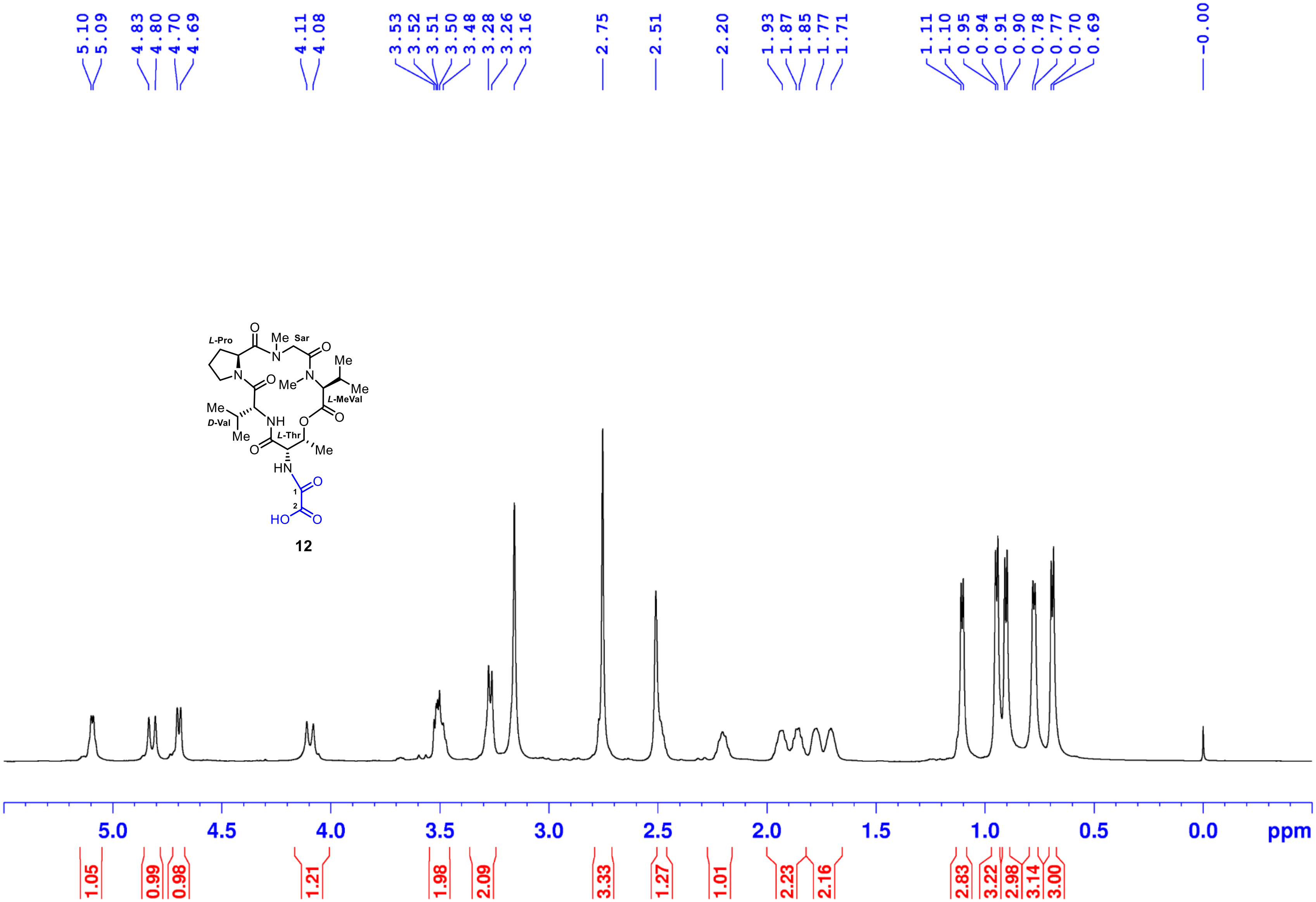

Figure S119-continue

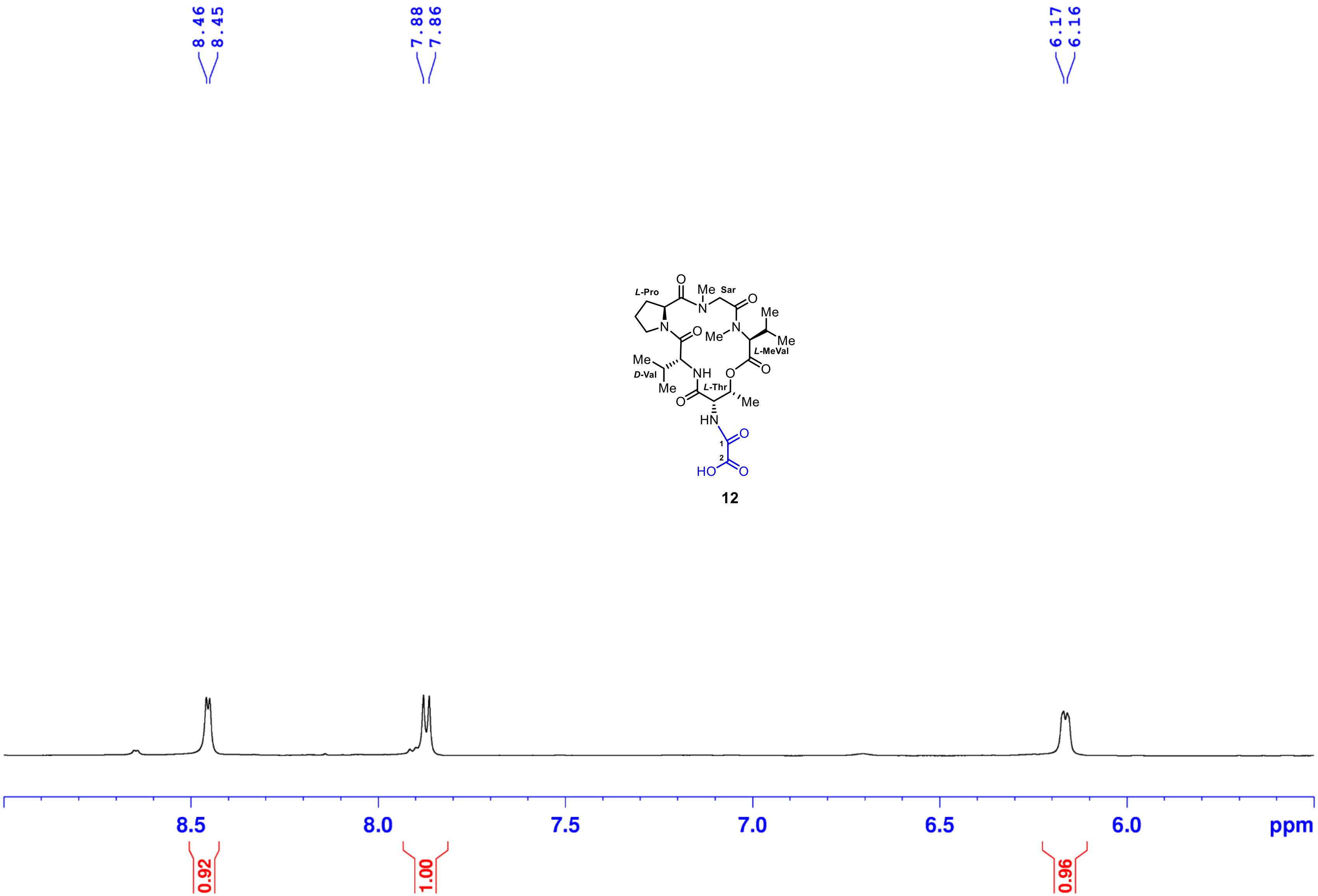

Figure S120. ^{13}C NMR (150 MHz) spectrum of compound **12** in DMSO-d_6

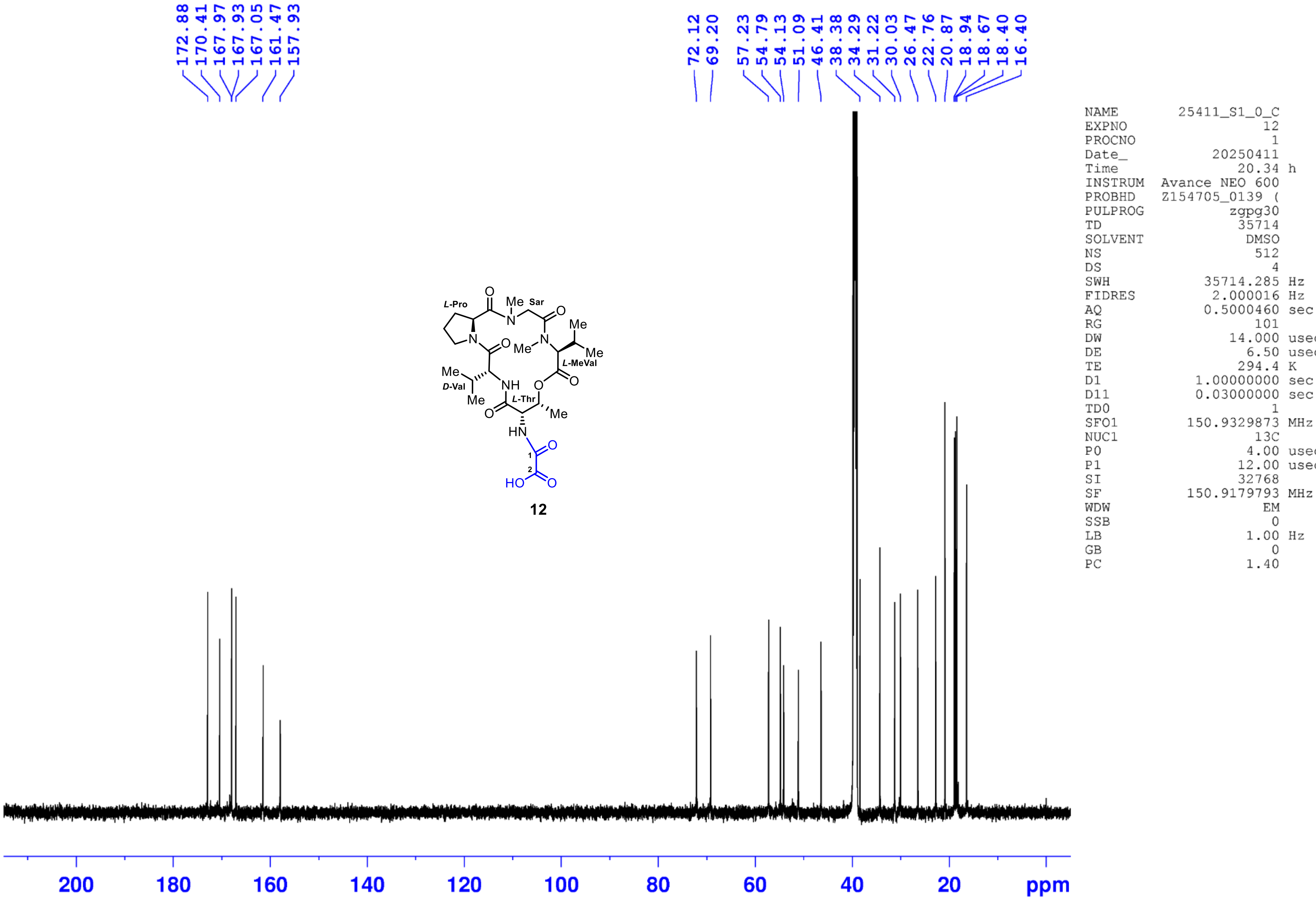

Figure S120-continue

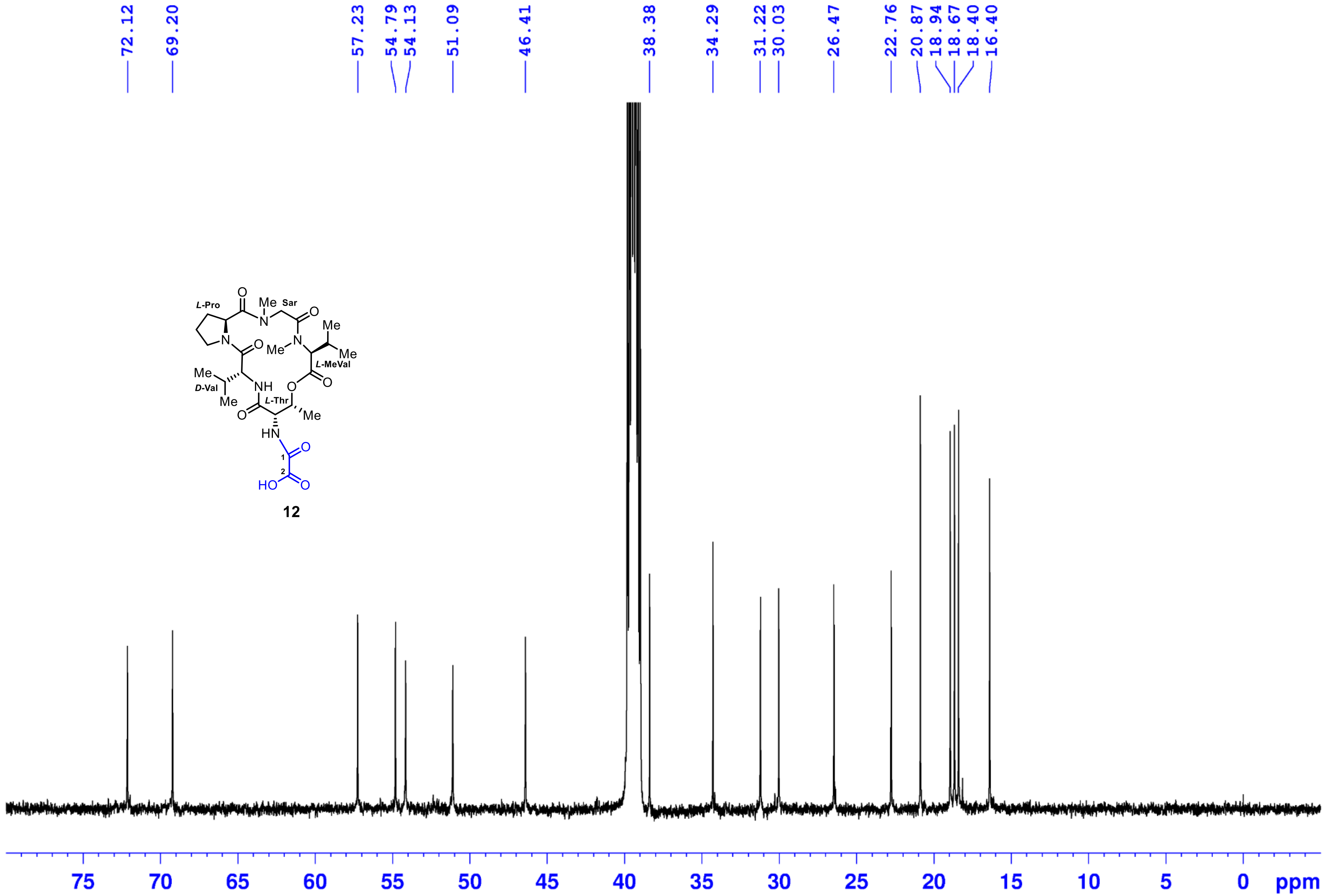

Figure S120-continue

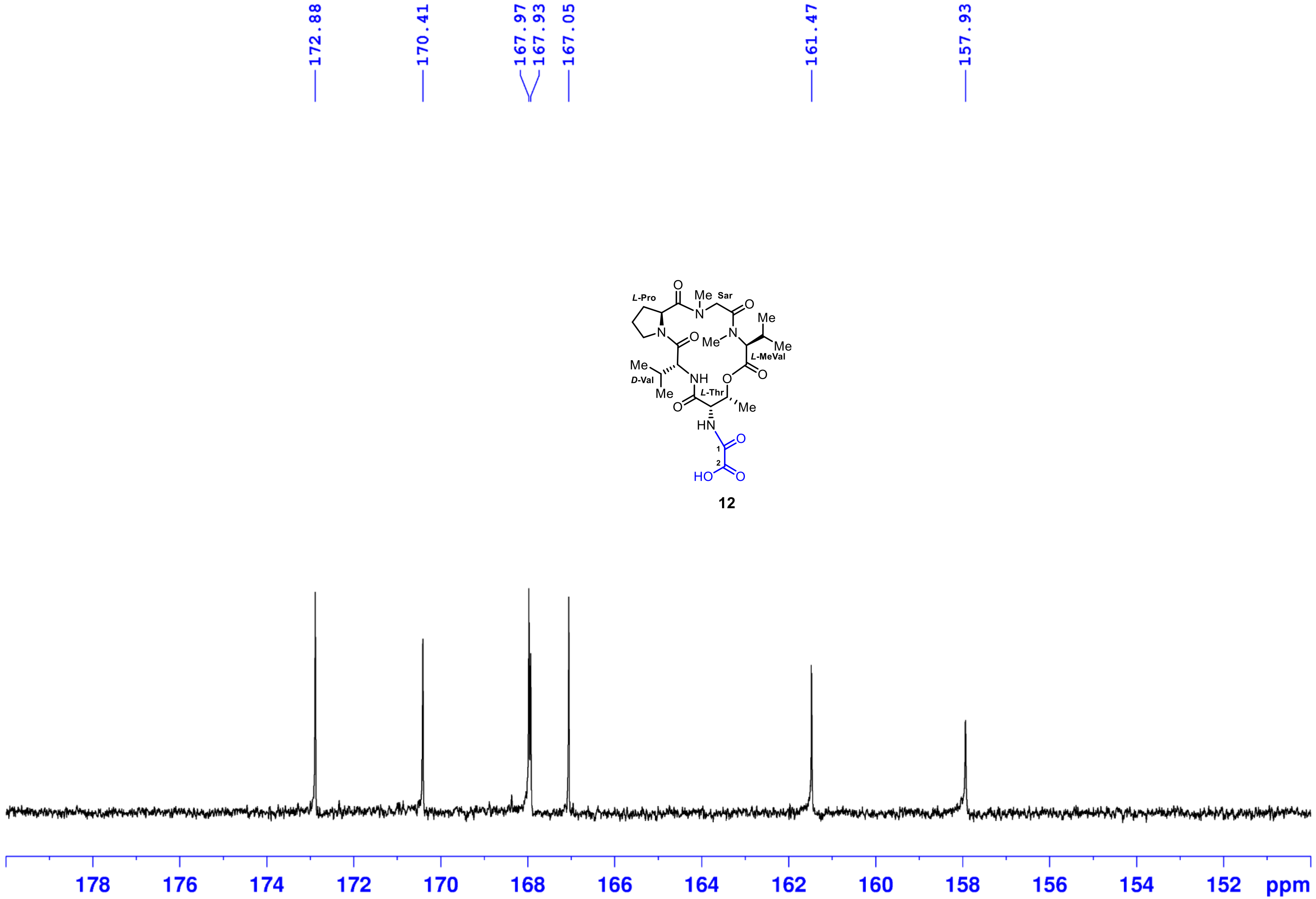

Figure S121. DEPT135 (150 MHz) spectrum of compound **12** in DMSO-d_6

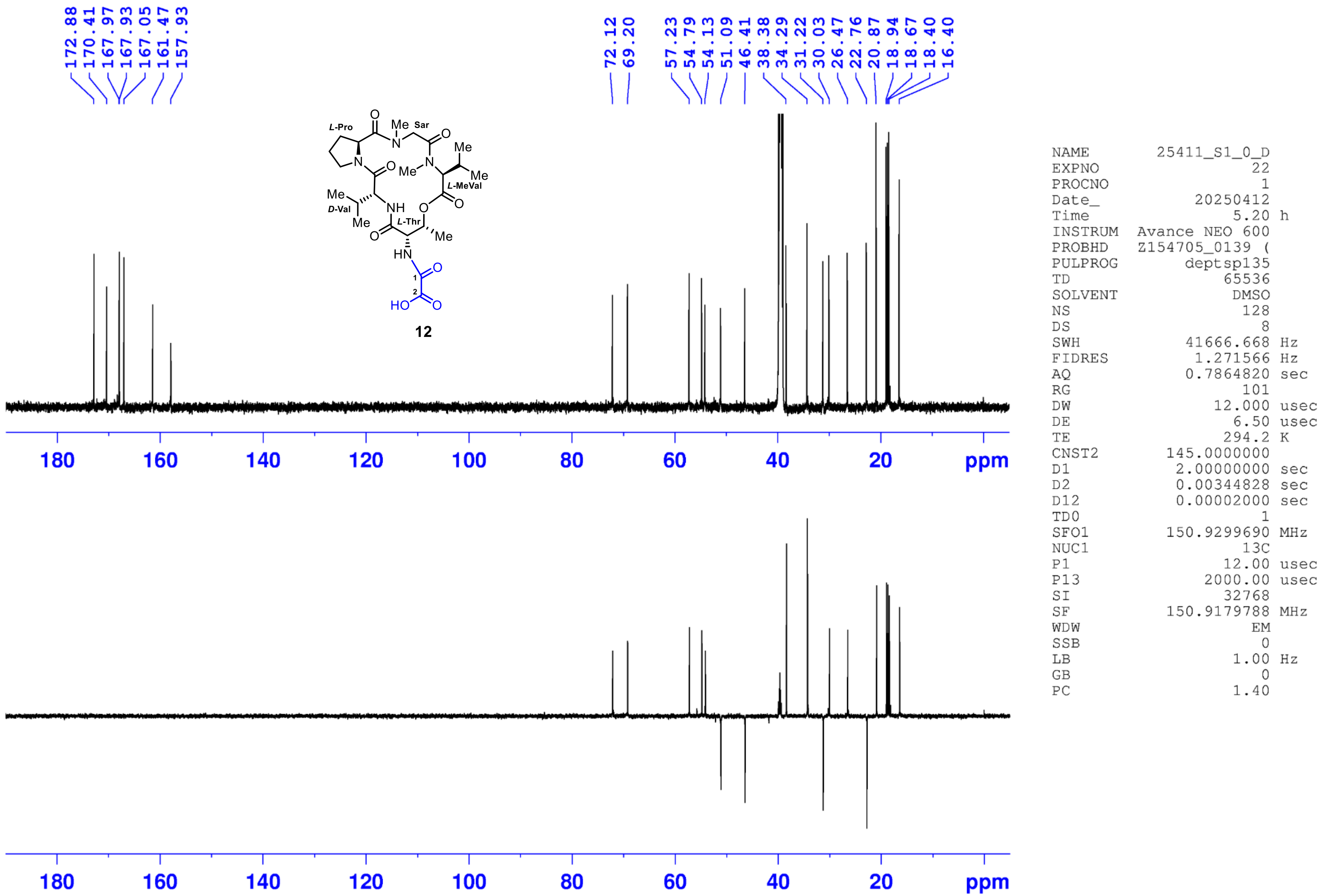

Figure S121-continue

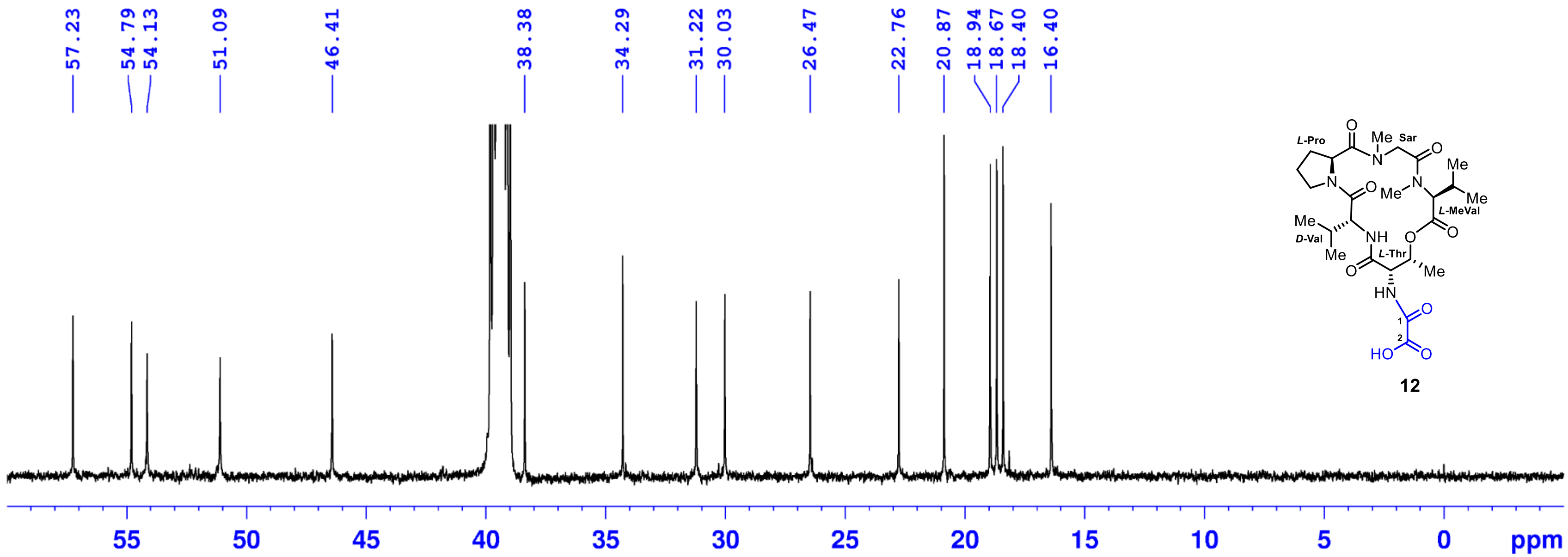

12

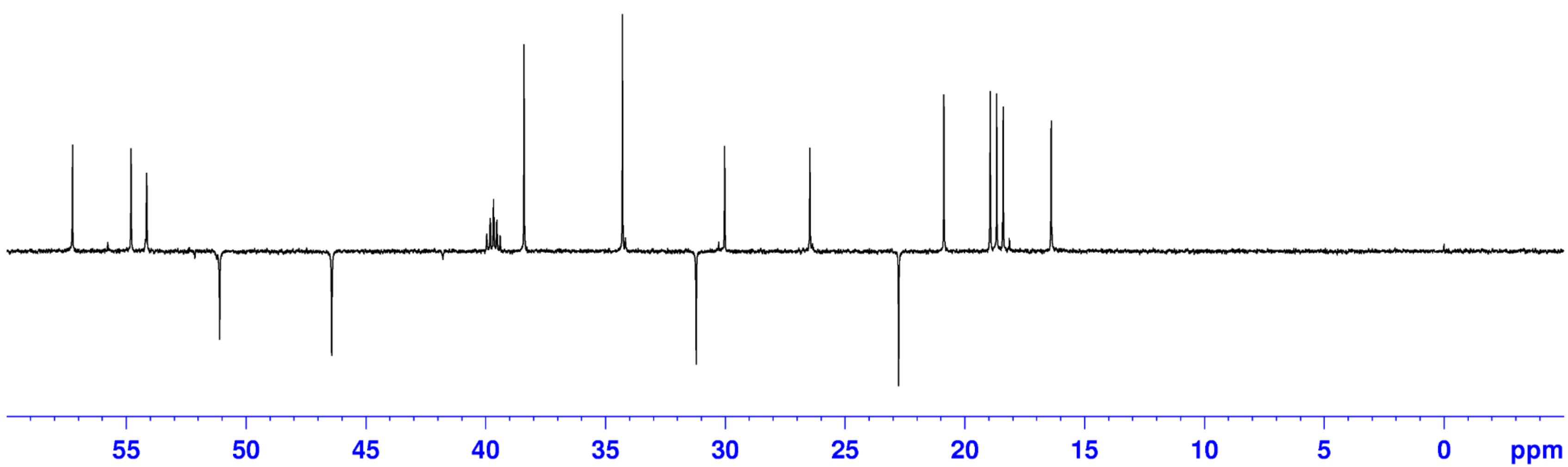

Figure S121-continue

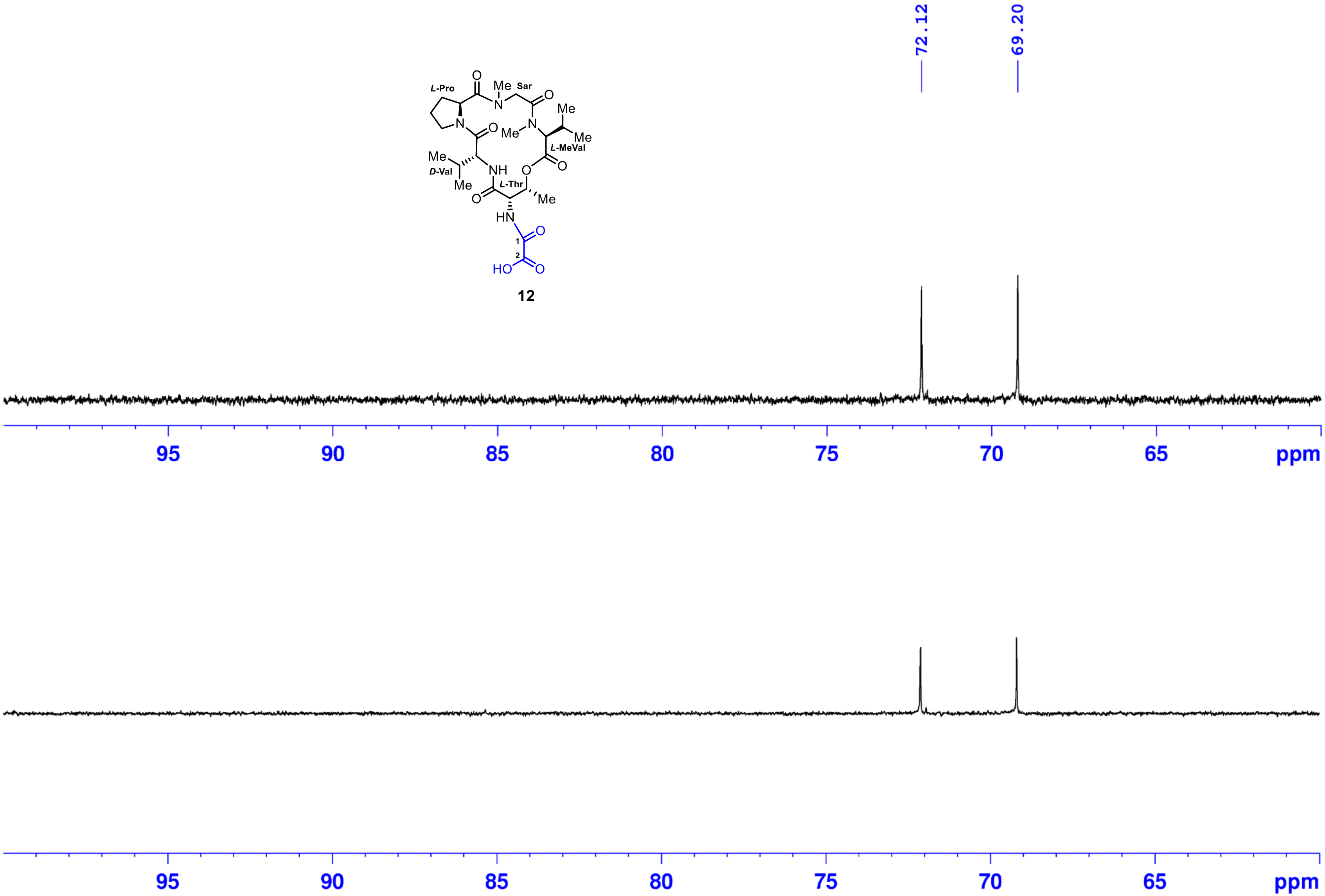

Figure S122. ^{1}H–^{1}H COSY (600 MHz) spectrum of compound **12** in DMSO-d_6

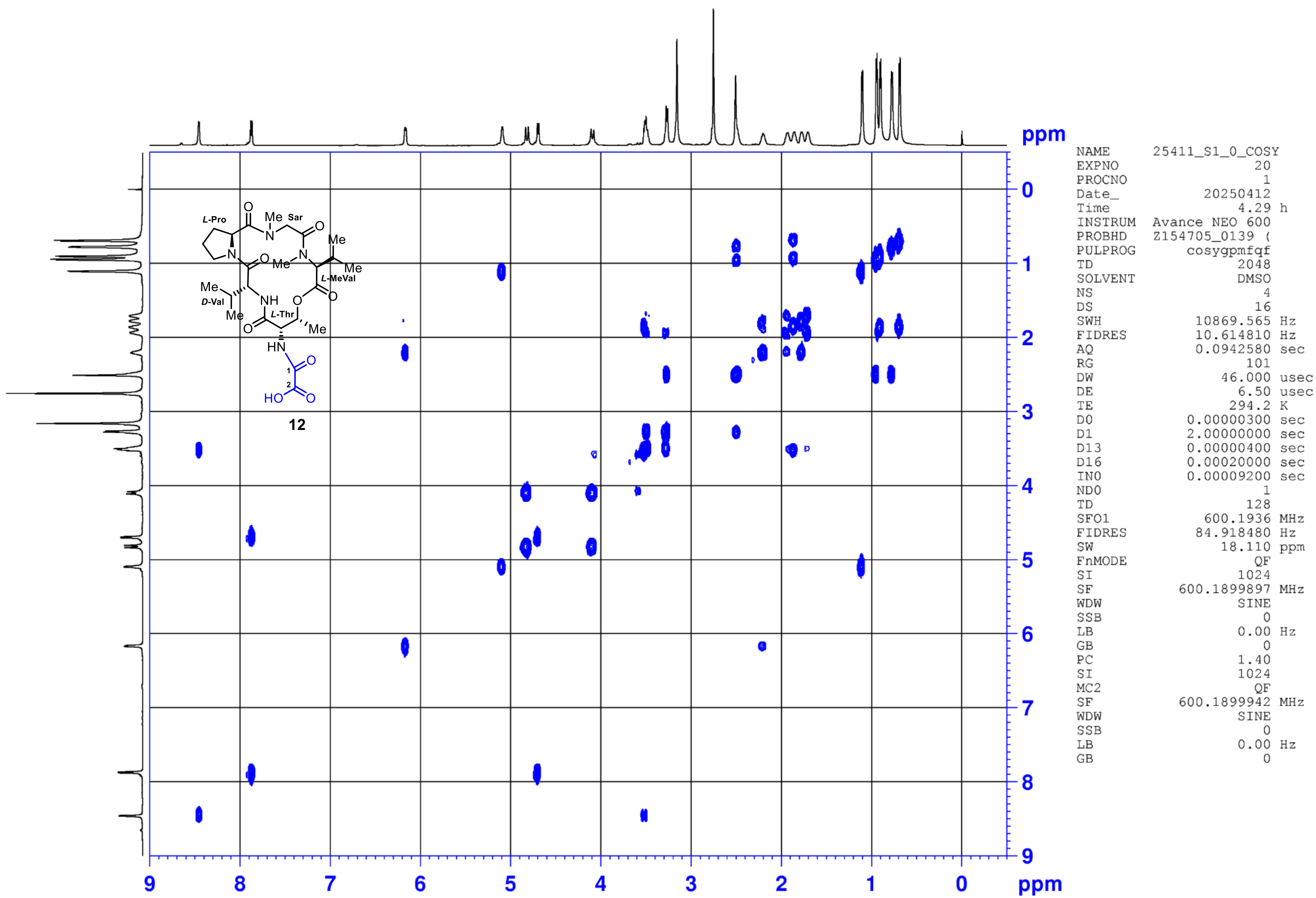

Figure S122-continue

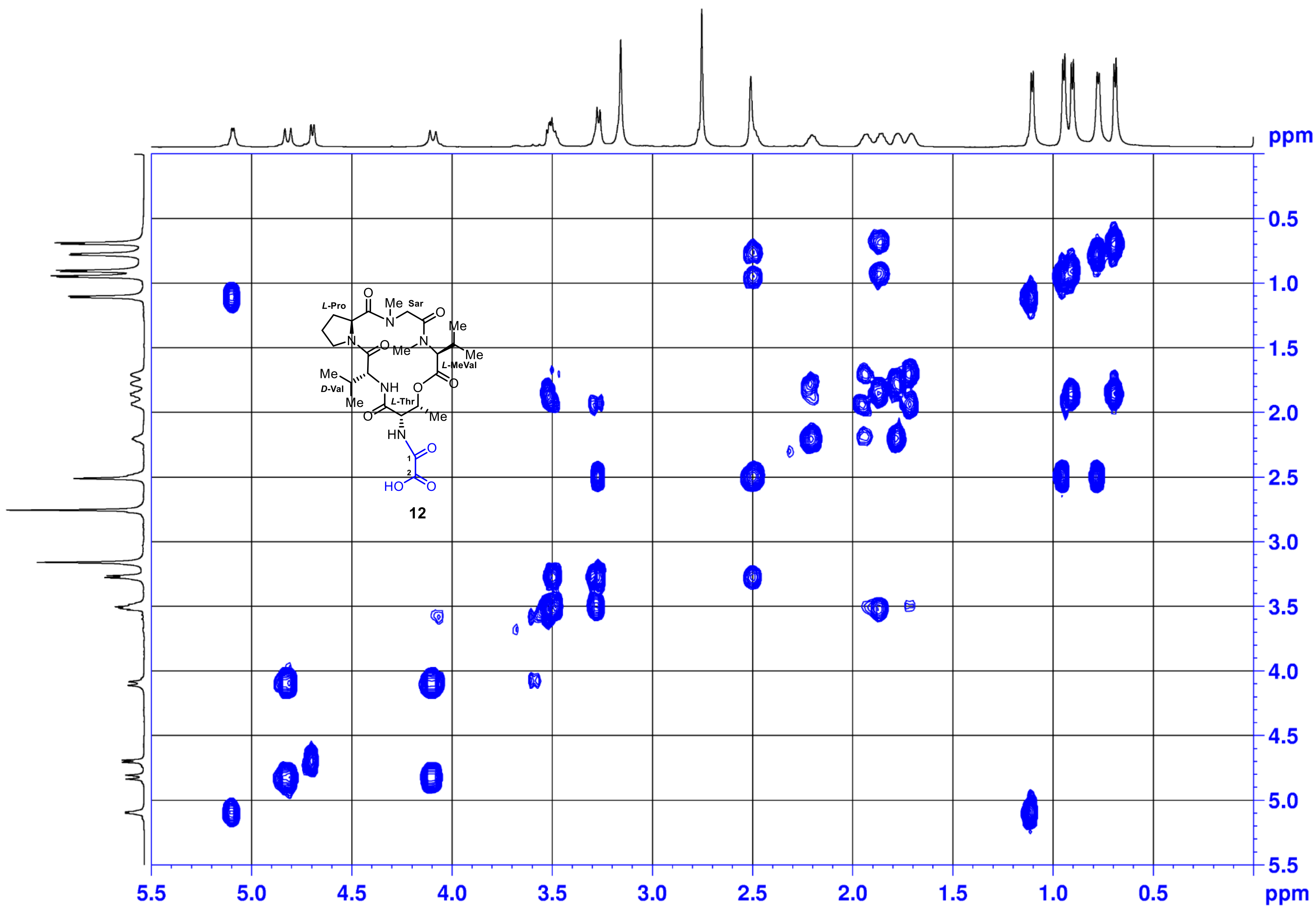

Figure S122-continue

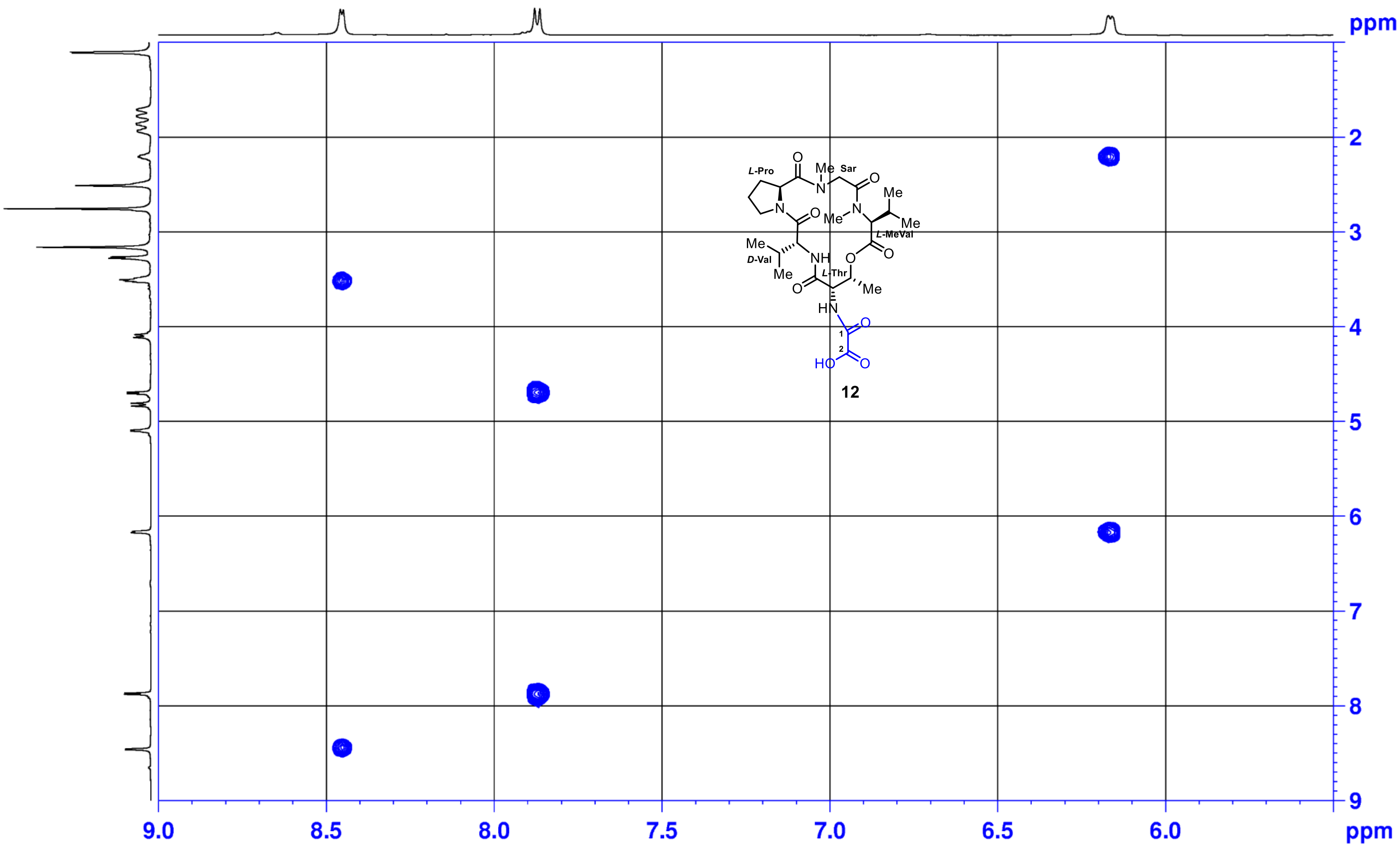

Figure S123. HSQC (600 MHz) spectrum of compound **12** in DMSO-d_6

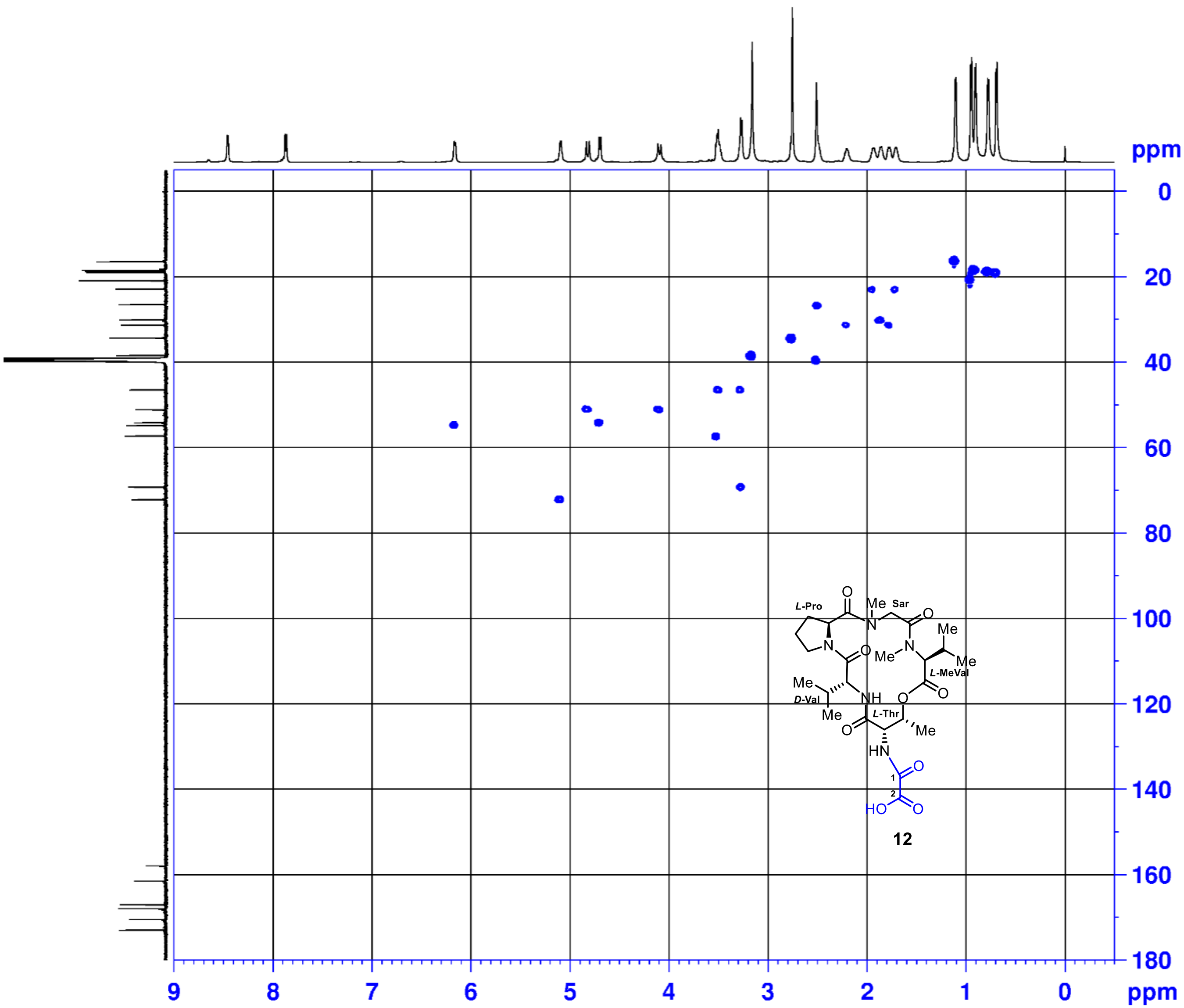

NAME	25411_S1_0_HSQC
EXPNO	18
PROCNO	1
Date_	20250412
Time	3.39 h
INSTRUM	Avance NEO 600
PROBHD	Z154705_0139 (
PULPROG	hsqcetgp
TD	1024
SOLVENT	DMSO
NS	4
DS	16
SWH	10869.565 Hz
FIDRES	21.229620 Hz
AQ	0.0471540 sec
RG	101
DW	46.000 usec
DE	6.50 usec
TE	294.7 K
CNST2	145.0000000
D0	0.00000300 sec
D1	1.50000000 sec
D4	0.00172414 sec
D11	0.03000000 sec
D16	0.00020000 sec
IN0	0.00001142 sec
ND0	2
TD	256
SFO1	150.9292 MHz
FIDRES	170.974503 Hz
SW	290.000 ppm
FnMODE	Echo-Antiecho
SI	1024
SF	600.1899907 MHz
WDW	QSINE
SSB	2
LB	0.00 Hz
GB	0
PC	1.40
SI	1024
MC2	echo-antiecho
SF	150.9179583 MHz
WDW	QSINE
SSB	2
LB	0.00 Hz
GB	0

Figure S123-continue

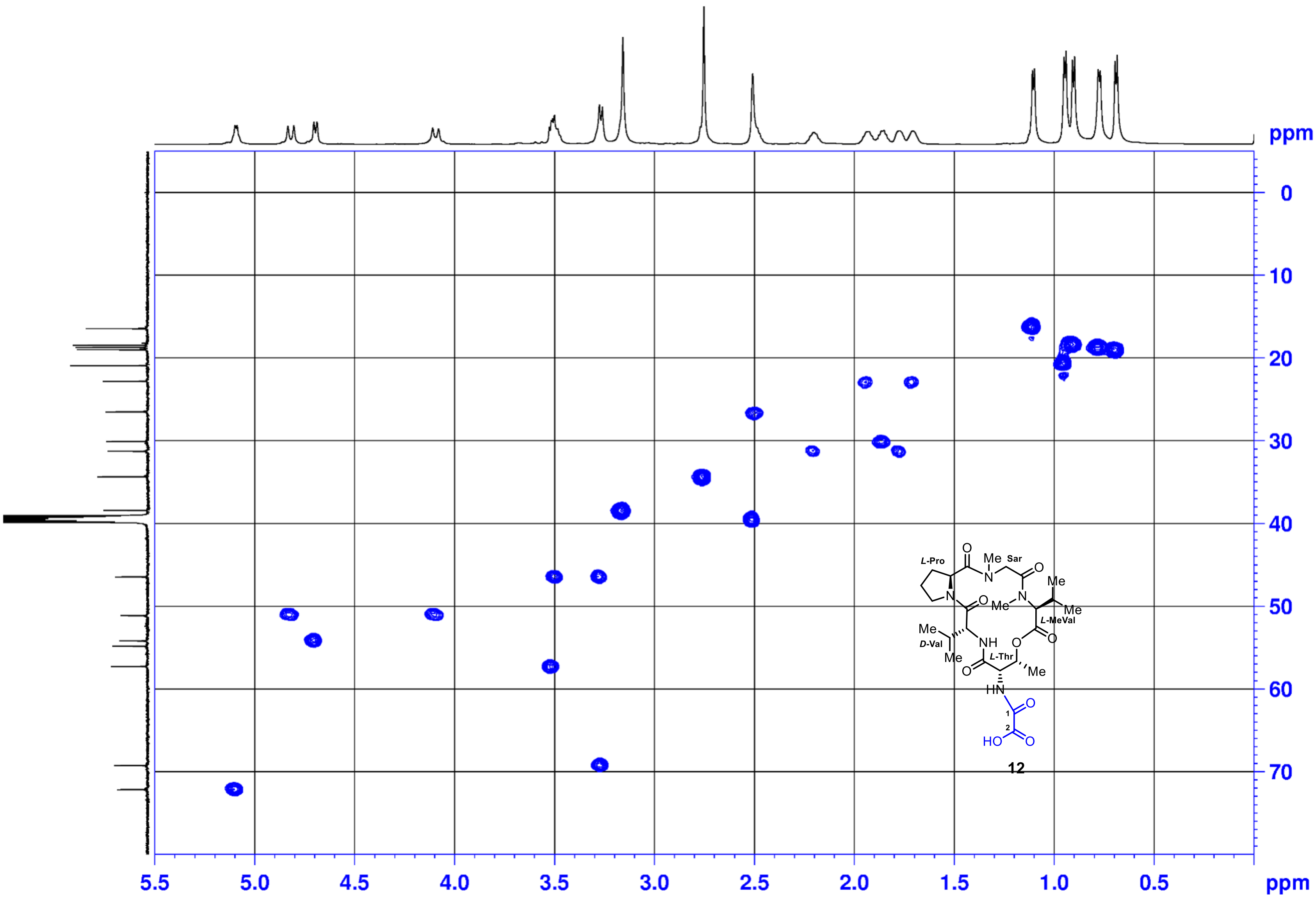

Figure S123-continue

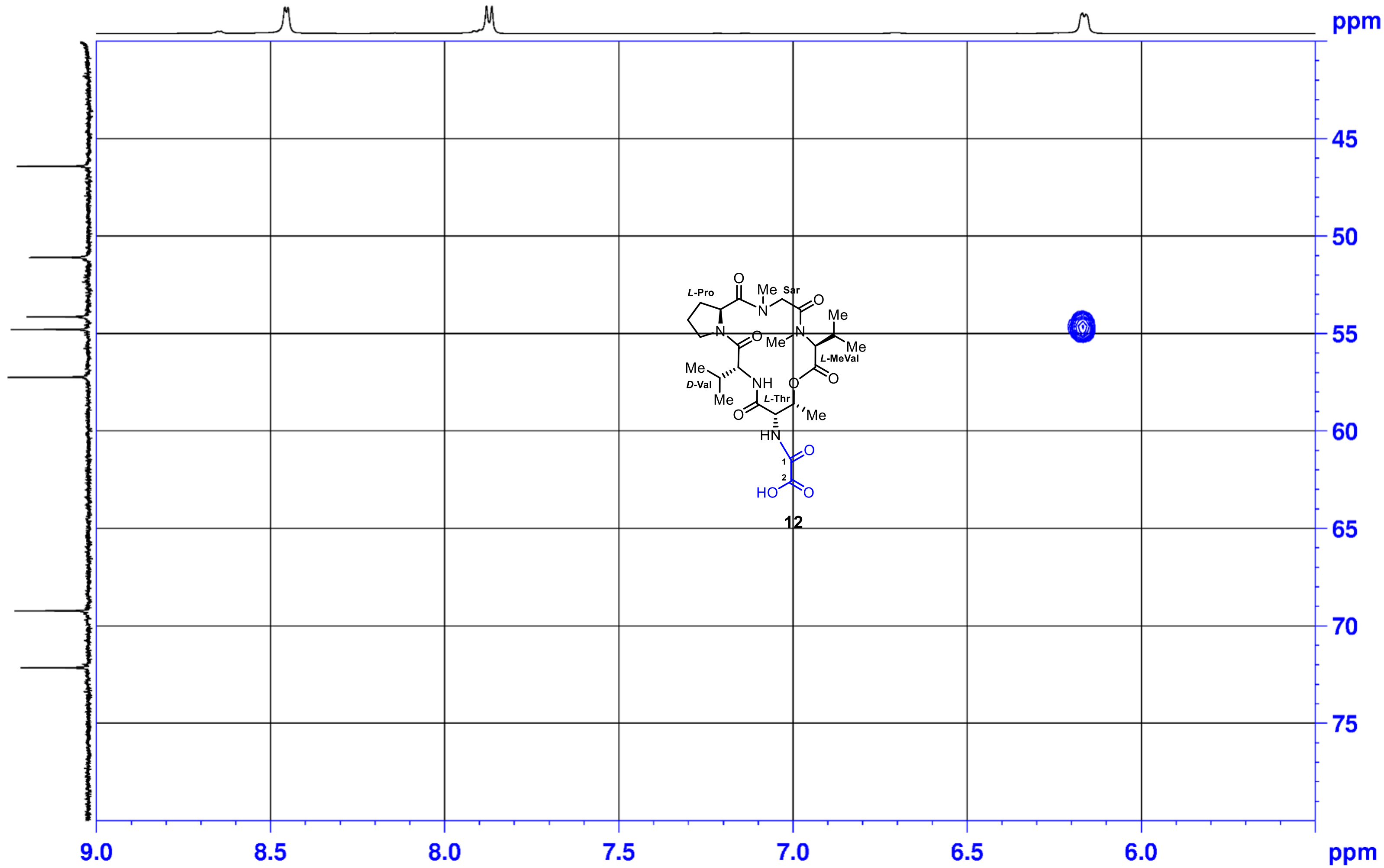

Figure S124. HMBC (600 MHz) spectrum of compound **12** in DMSO-d_6

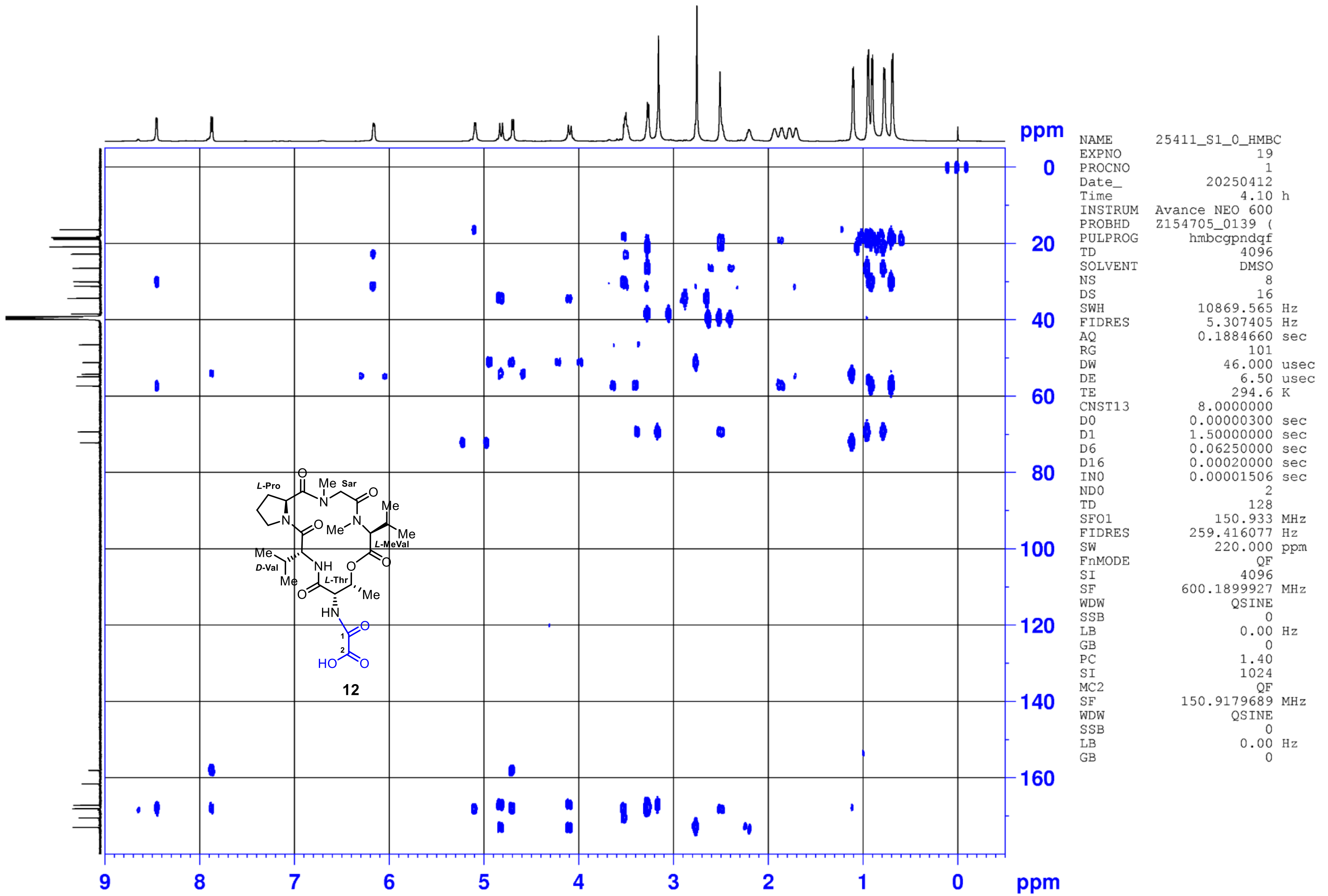

Figure S124-continue

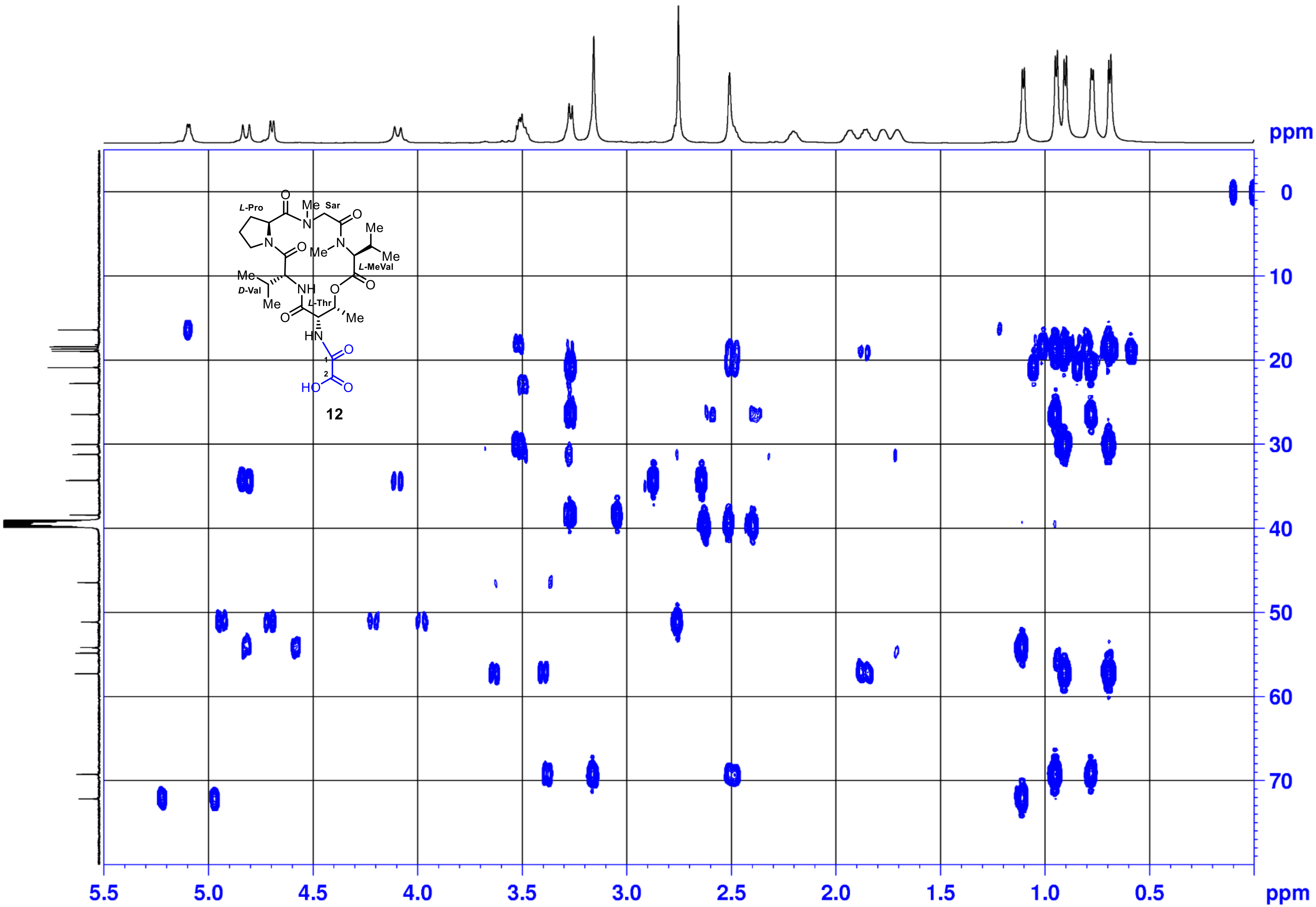

Figure S124-continue

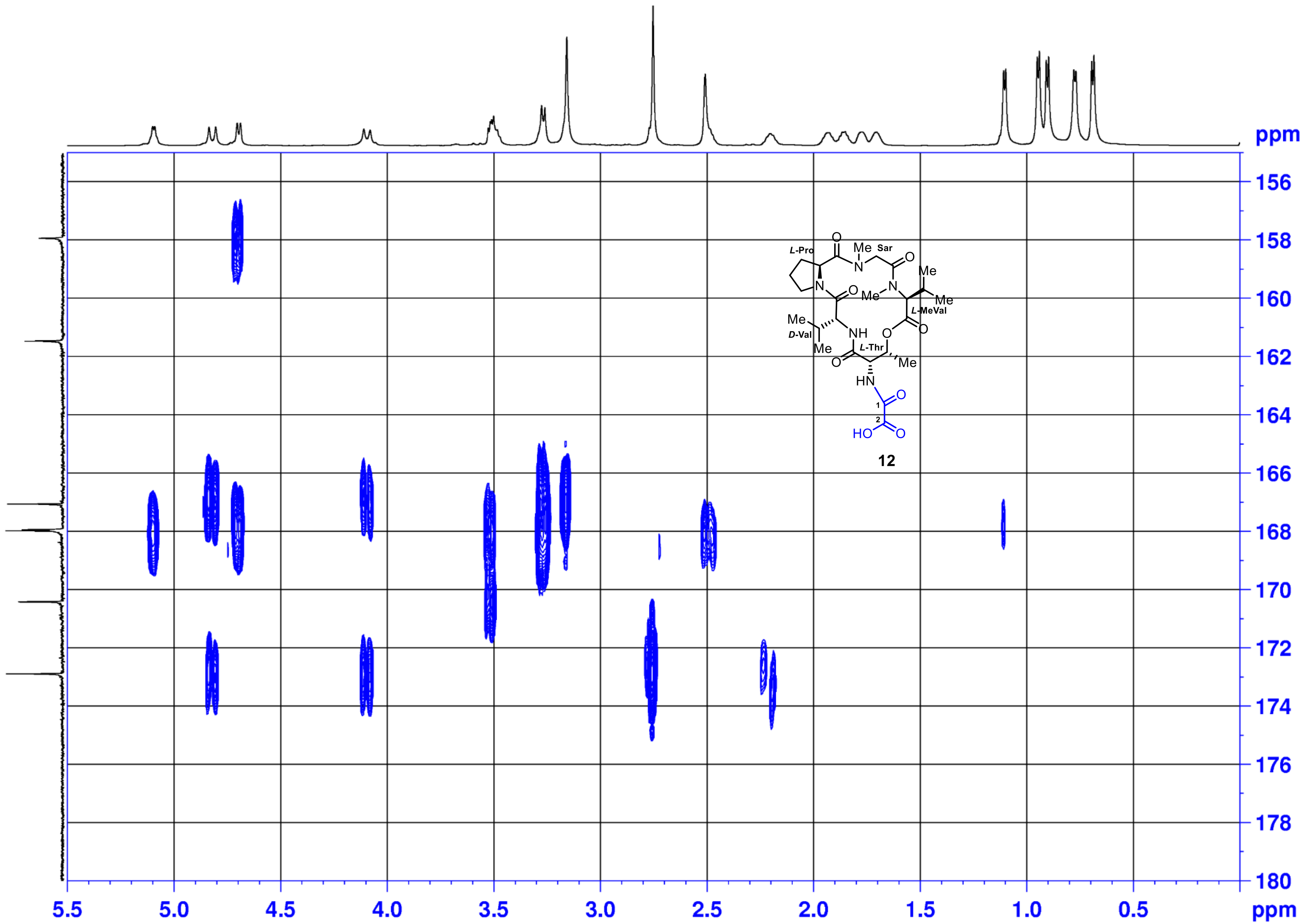

Figure S124-continue

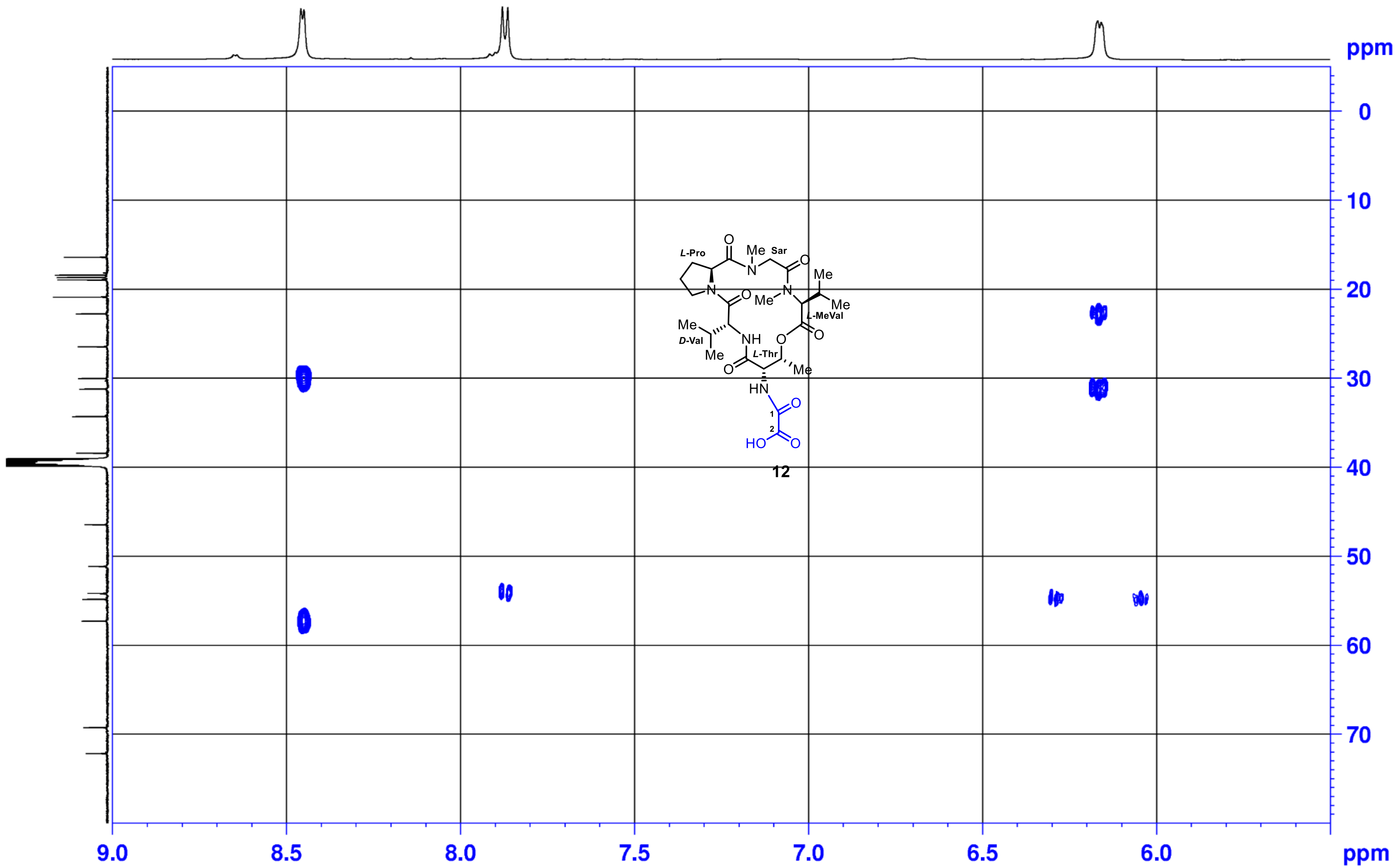

Figure S124-continue

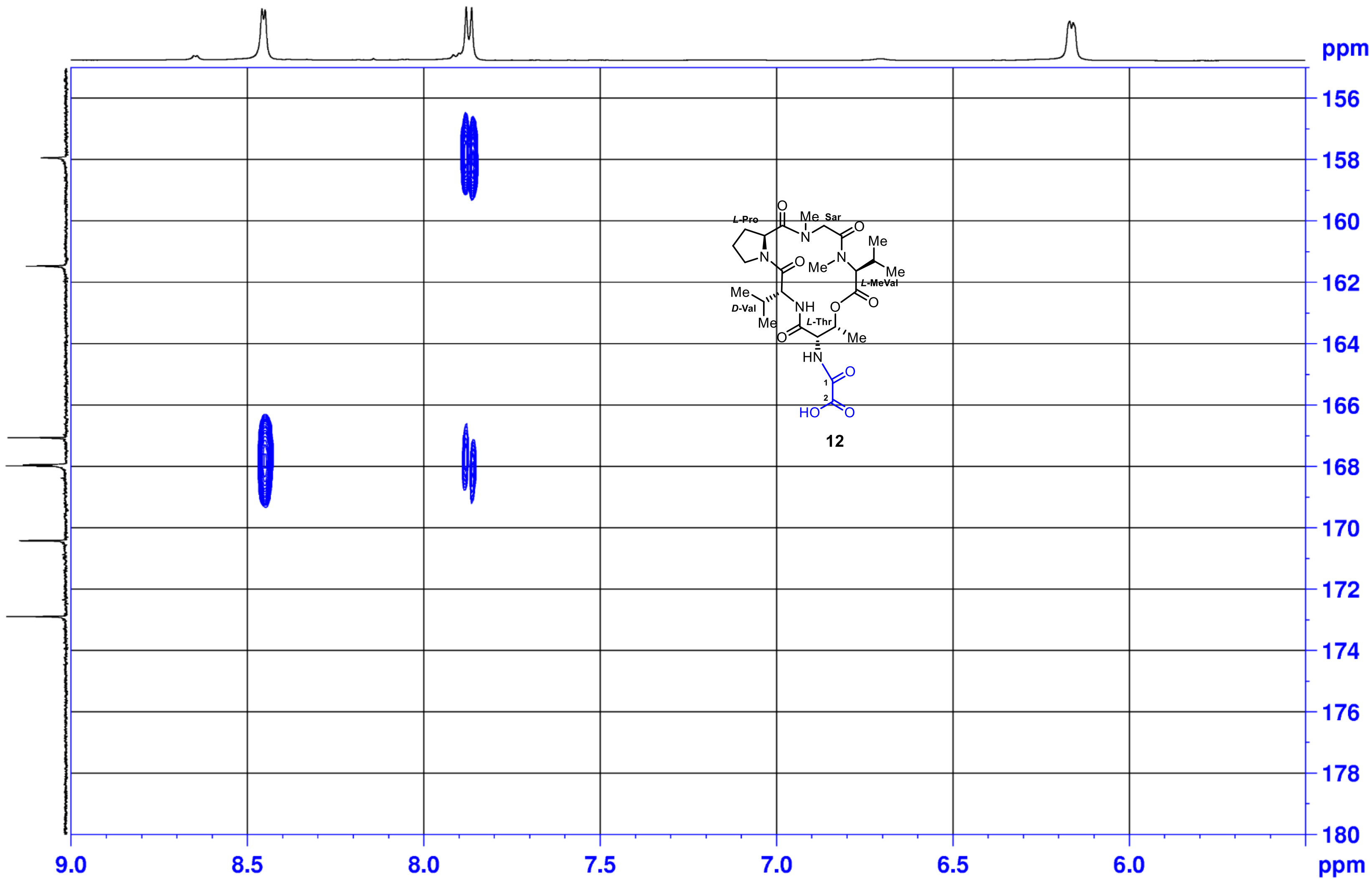

Figure S125. ROESY (600 MHz) spectrum of compound **12** in DMSO-d_6

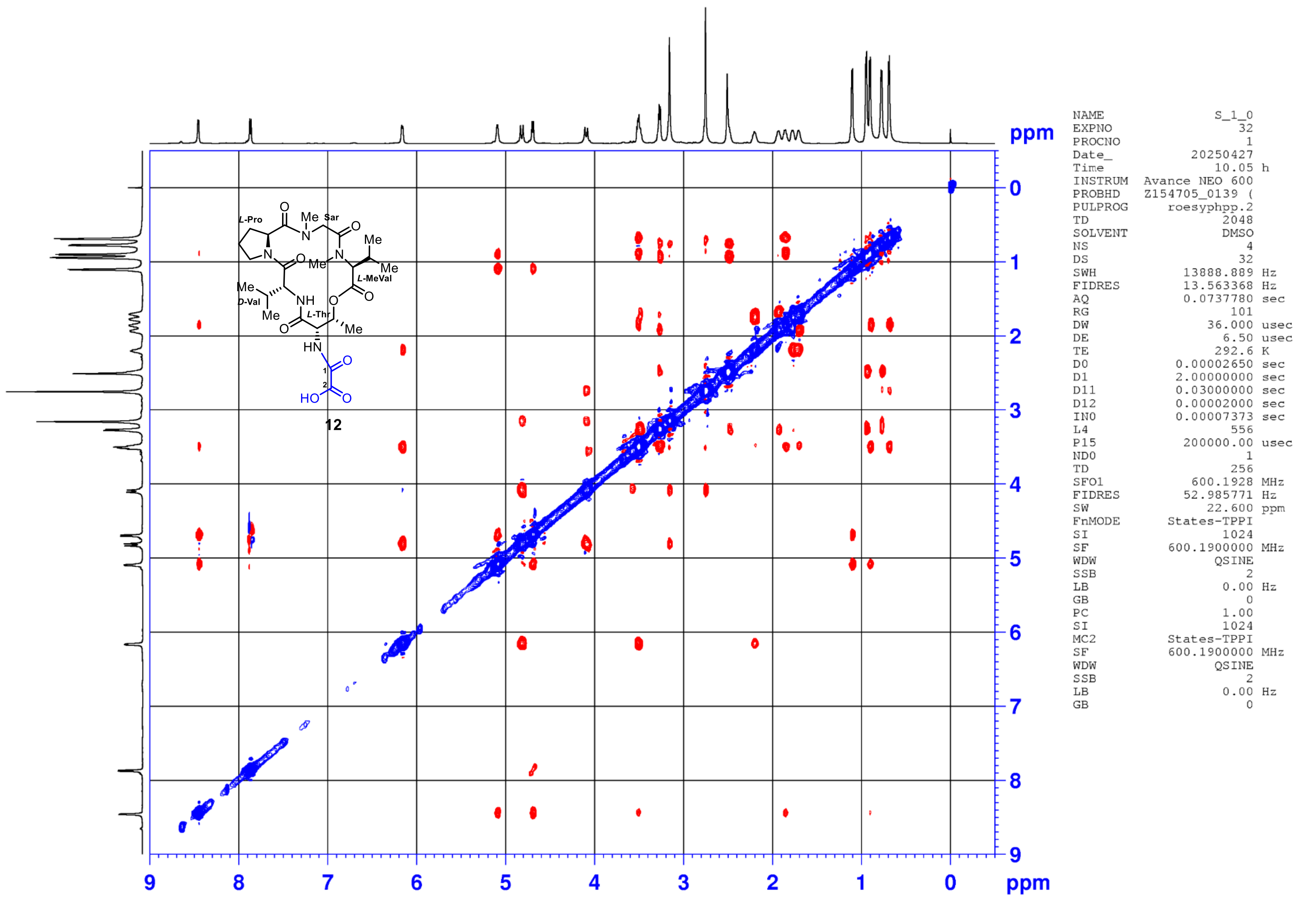

Figure S125-continue

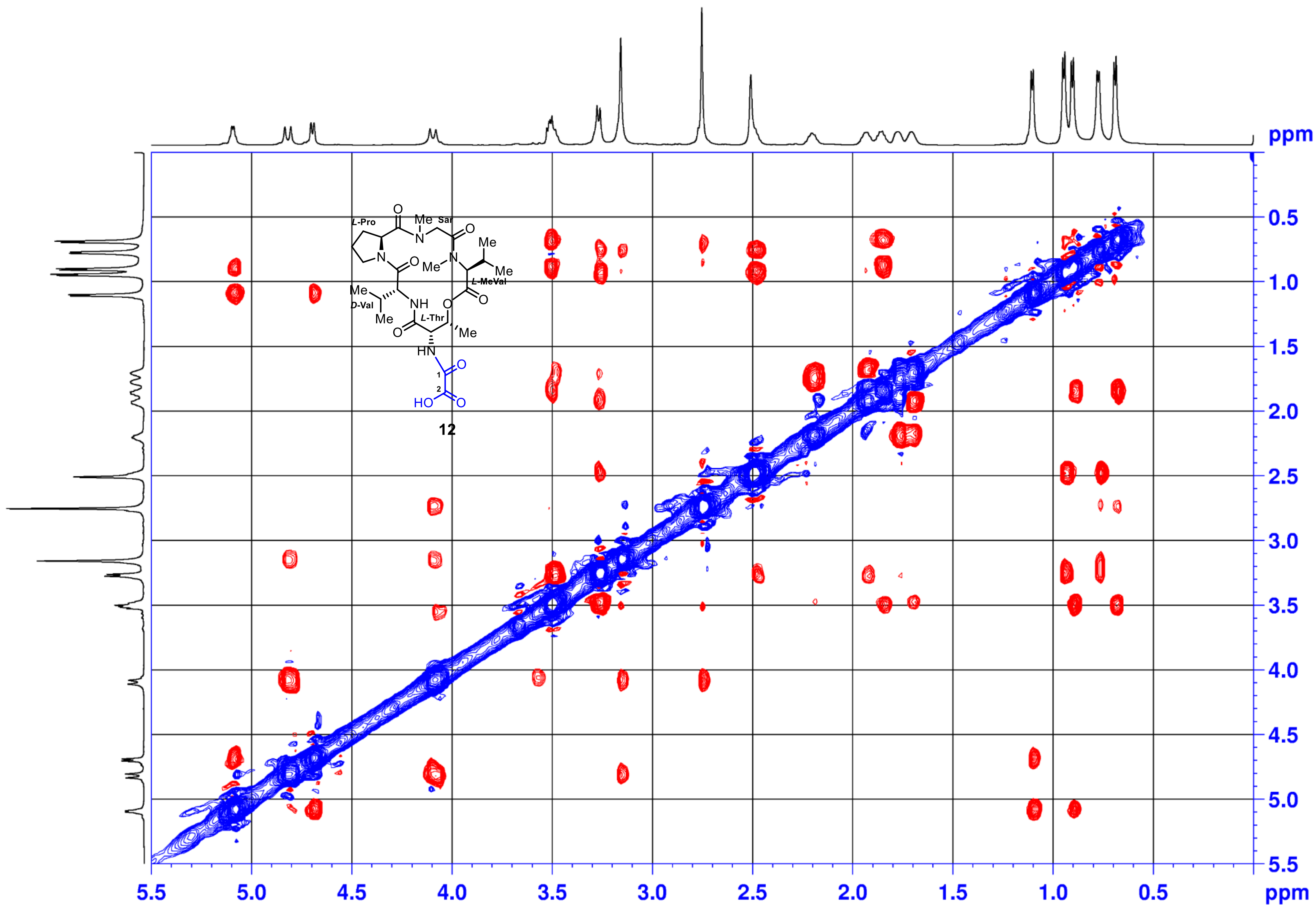

Figure S125-continue

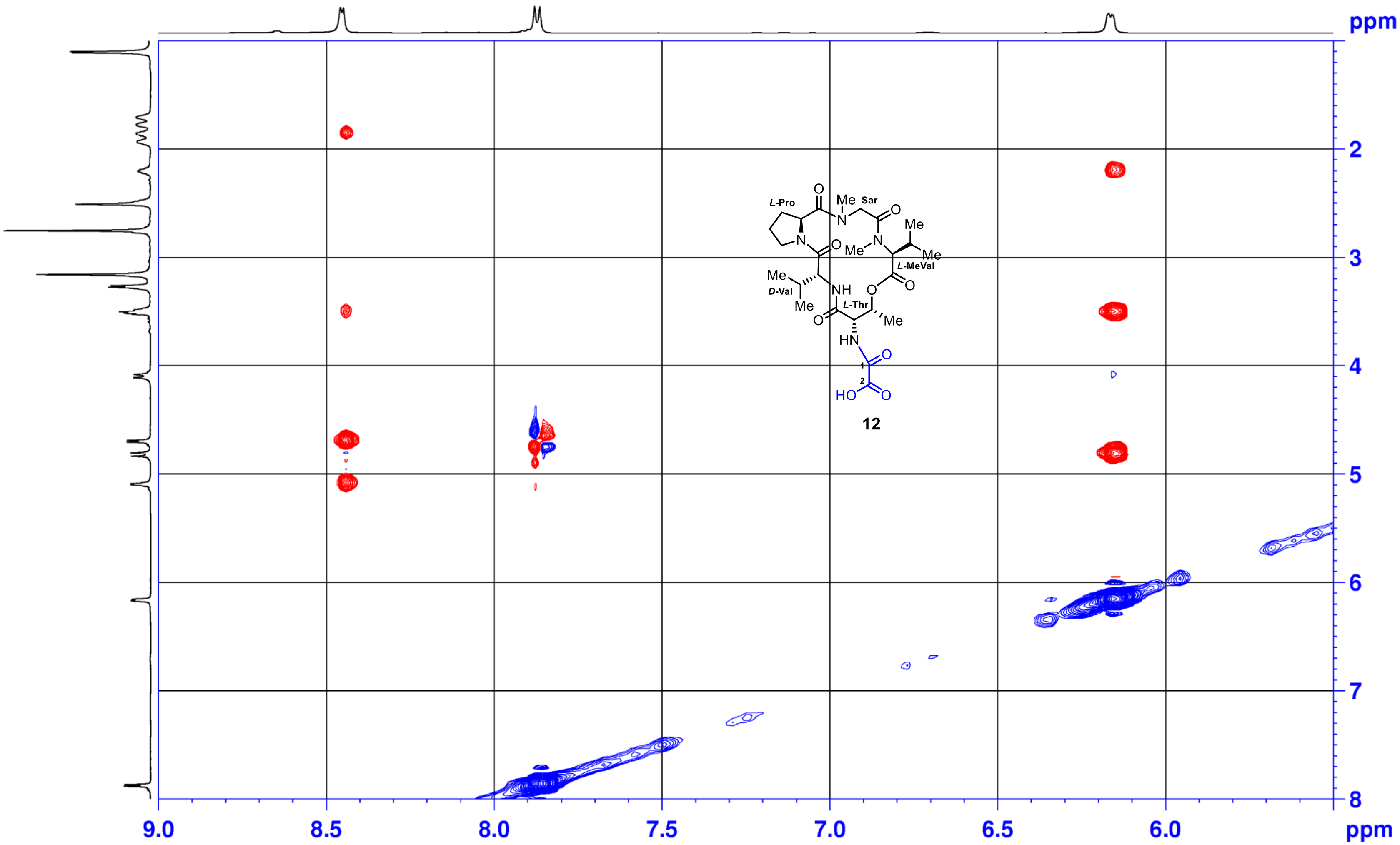

Figure S126. Marfey's analysis of compound **12** and standard amino acids

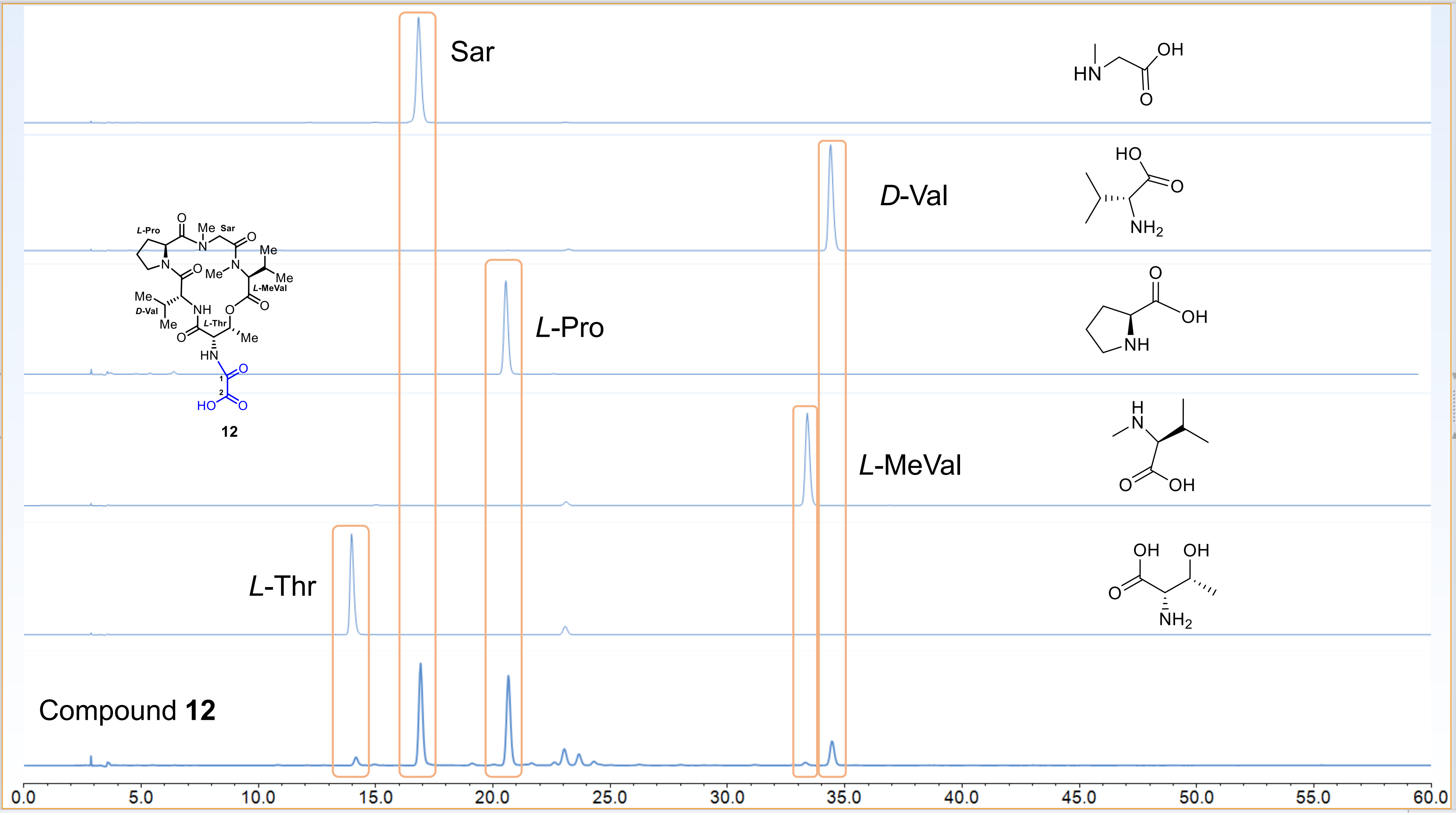

Figure S127. UV spectrum of compound **13**

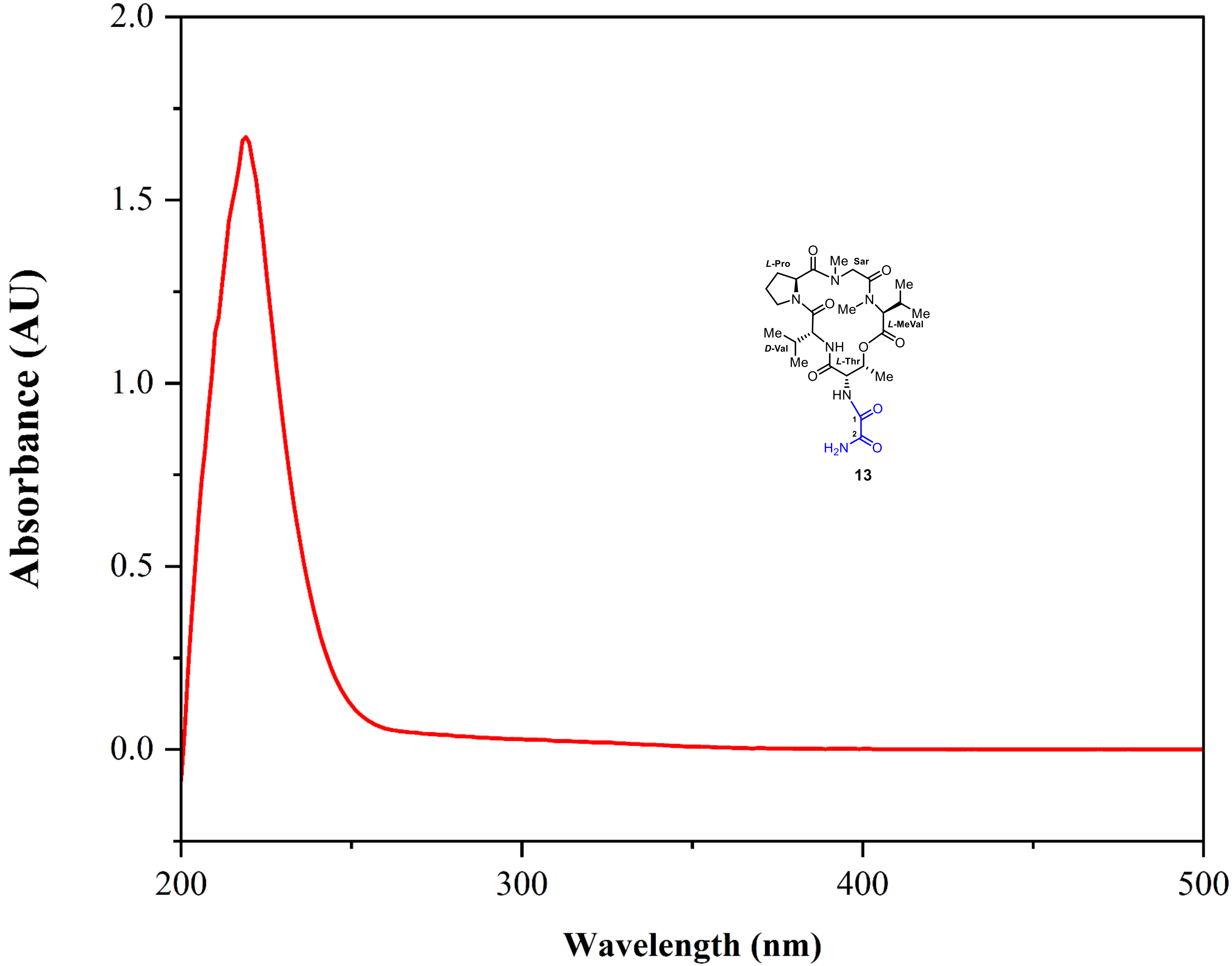

Figure S128. HR-ESIMS for compound **13**

Compound Spectrum SmartFormula Report

Analysis Info

Analysis	D:\Data\SCSIO Metabonomics data\MS\202508\panghe_S-2-2_pos_P1-F-5_805.d	Acquisition Date	2025-08-19 11:23:04
Method	Tune_pos_middle_avg_100-2000_calibration_1.5m.m	Operator	SCSIO User
Sample	panghe_S-2-2_pos	Instrument	timsTOF Pro 2 1875087.11040
Comment			

Acquisition Parameter

Source Type	ESI	Ion Polarity	Positive	Set Nebulizer	0.3 Bar
Scan Begin	100 m/z	Set Capillary	3500 V	Set Dry Heater	200 °C
Scan End	2000 m/z	Set Multipole RF	500.0 Vpp	Set Dry Gas	3.5 l/min
		Set Collision Cell RF	2000.0 Vpp	ICC active	Off
		IMS Active	Off	ICC Target	2000000 cts.
		IMS Collision Cell In	300.0 V		

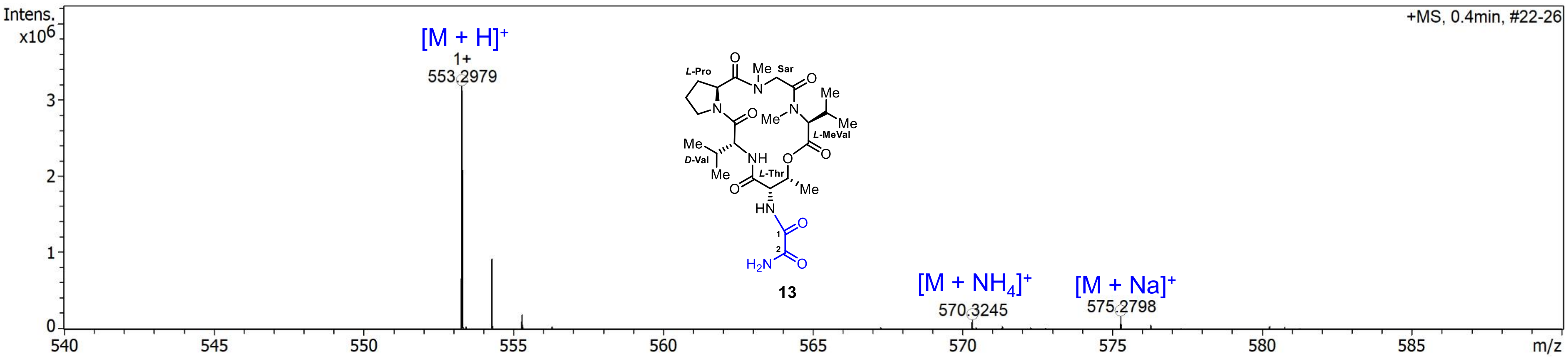

Meas. m/z	#	Ion Formula	Score	m/z	err [mDa]	err [ppm]	mSigma	rdb (neutral)	e⁻ Conf	Adduct
553.297883	1	C25H41N6O8	100.00	553.298039	0.2	0.3	6.0	9.0	even	M+H
570.324500	1	C25H44N7O8	100.00	570.324588	0.1	0.2	4.8	8.0	even	M+H
575.279826	1	C25H40N6NaO8	100.00	575.279983	0.2	0.3	8.6	9.0	even	M+H
1105.588150	1	C50H81N12O16	100.00	1105.588801	0.7	0.6	6.5	17.0	even	M+H
1122.614875	1	C50H84N13O16	100.00	1122.615350	0.5	0.4	9.0	16.0	even	M+H
1127.569967	1	C50H80N12NaO16	100.00	1127.570745	0.8	0.7	27.3	17.0	even	M+H

Figure S129. ^{1}H NMR (600 MHz) spectrum of compound **13** in DMSO-d_6

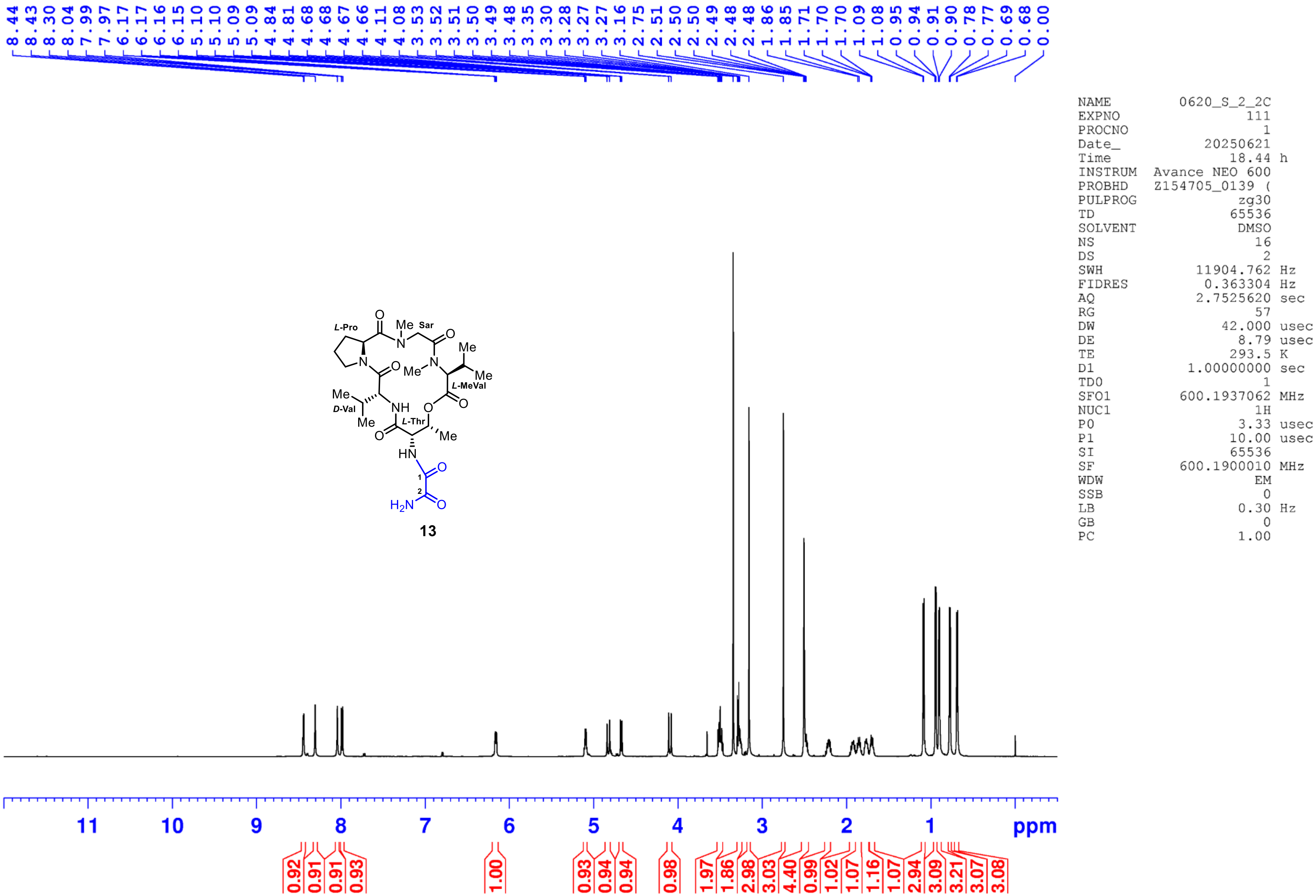

Figure S129-continue

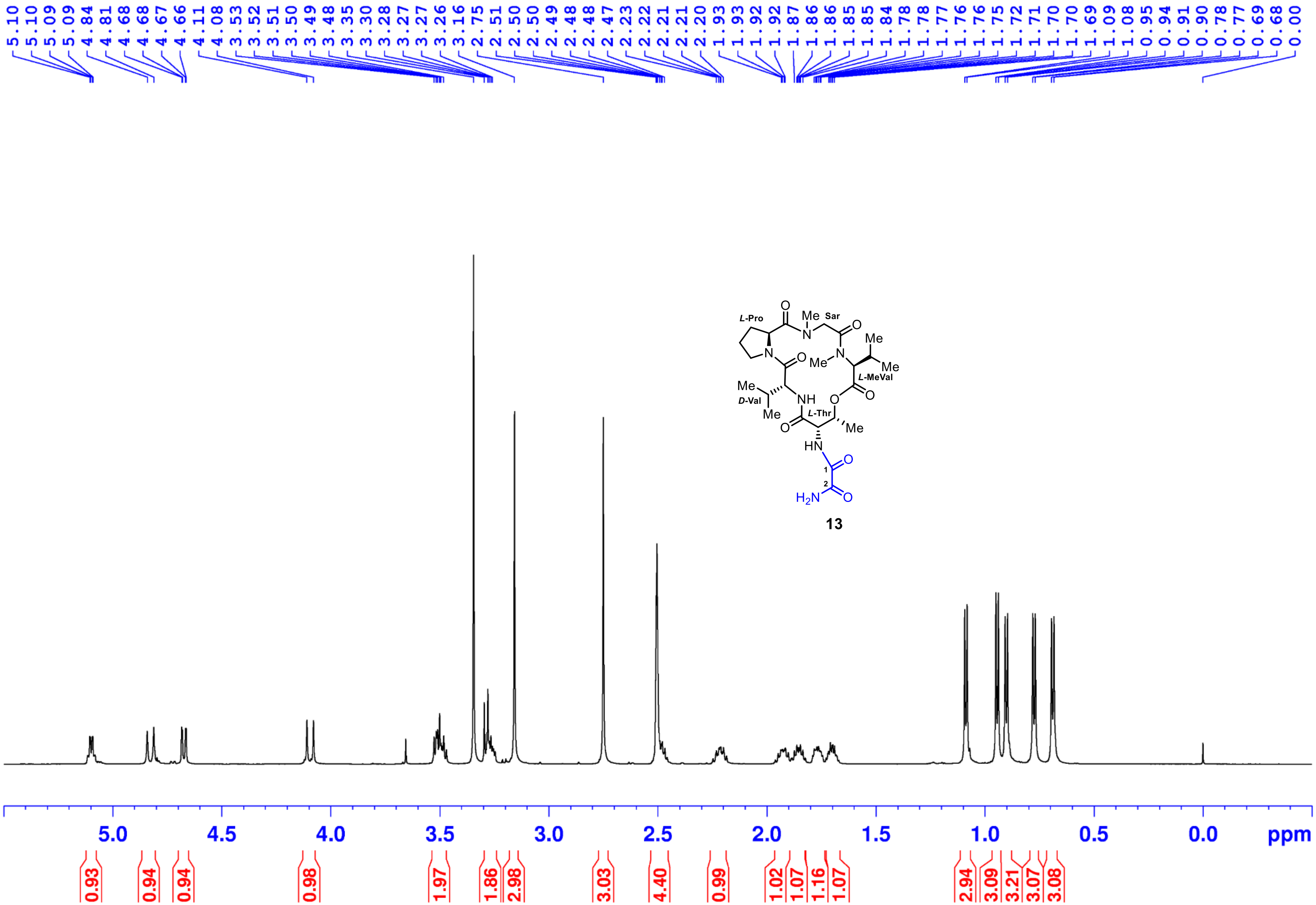

Figure S129-continue

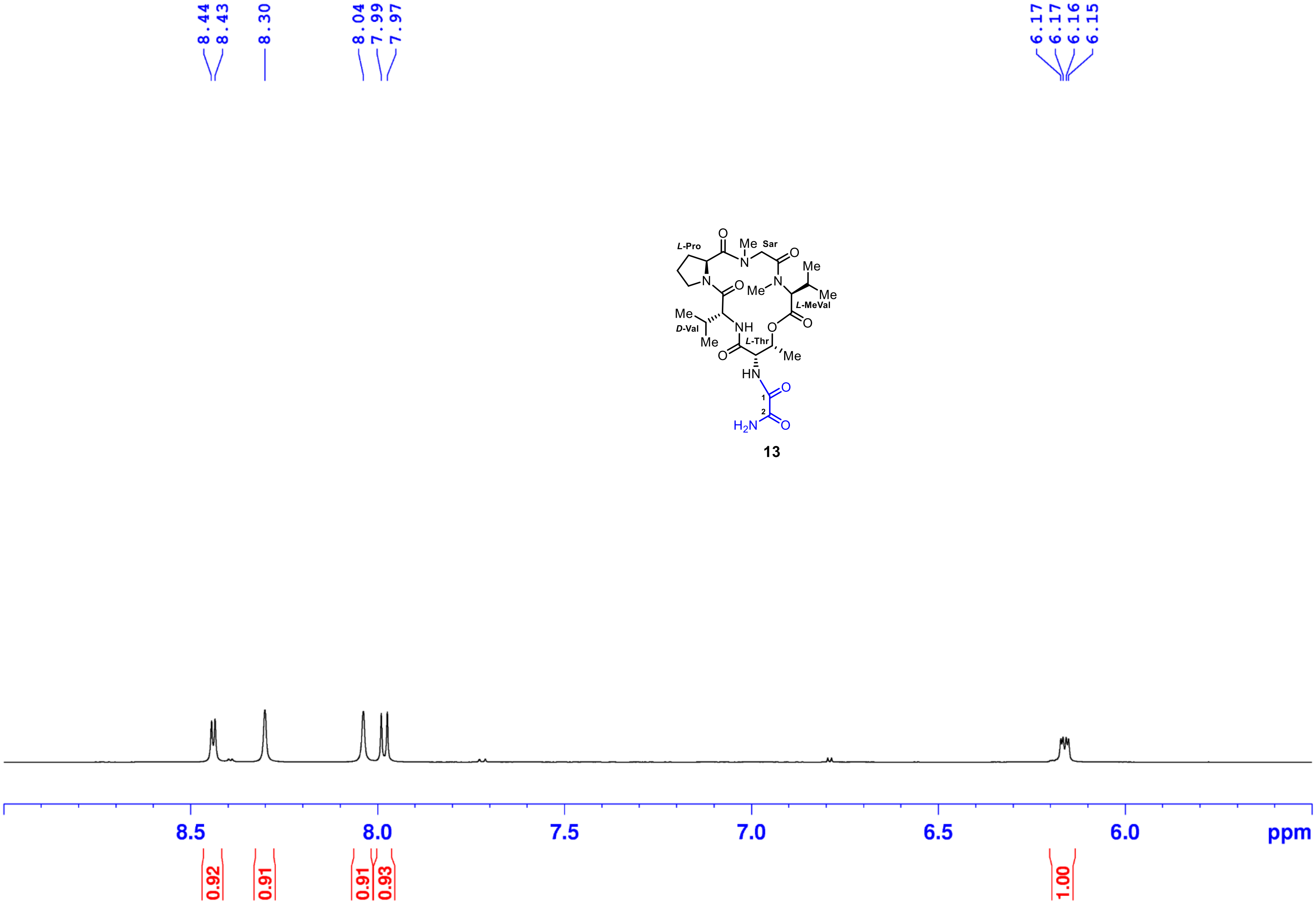

Figure S130. ^{13}C NMR (150 MHz) spectrum of compound **13** in DMSO-d_6

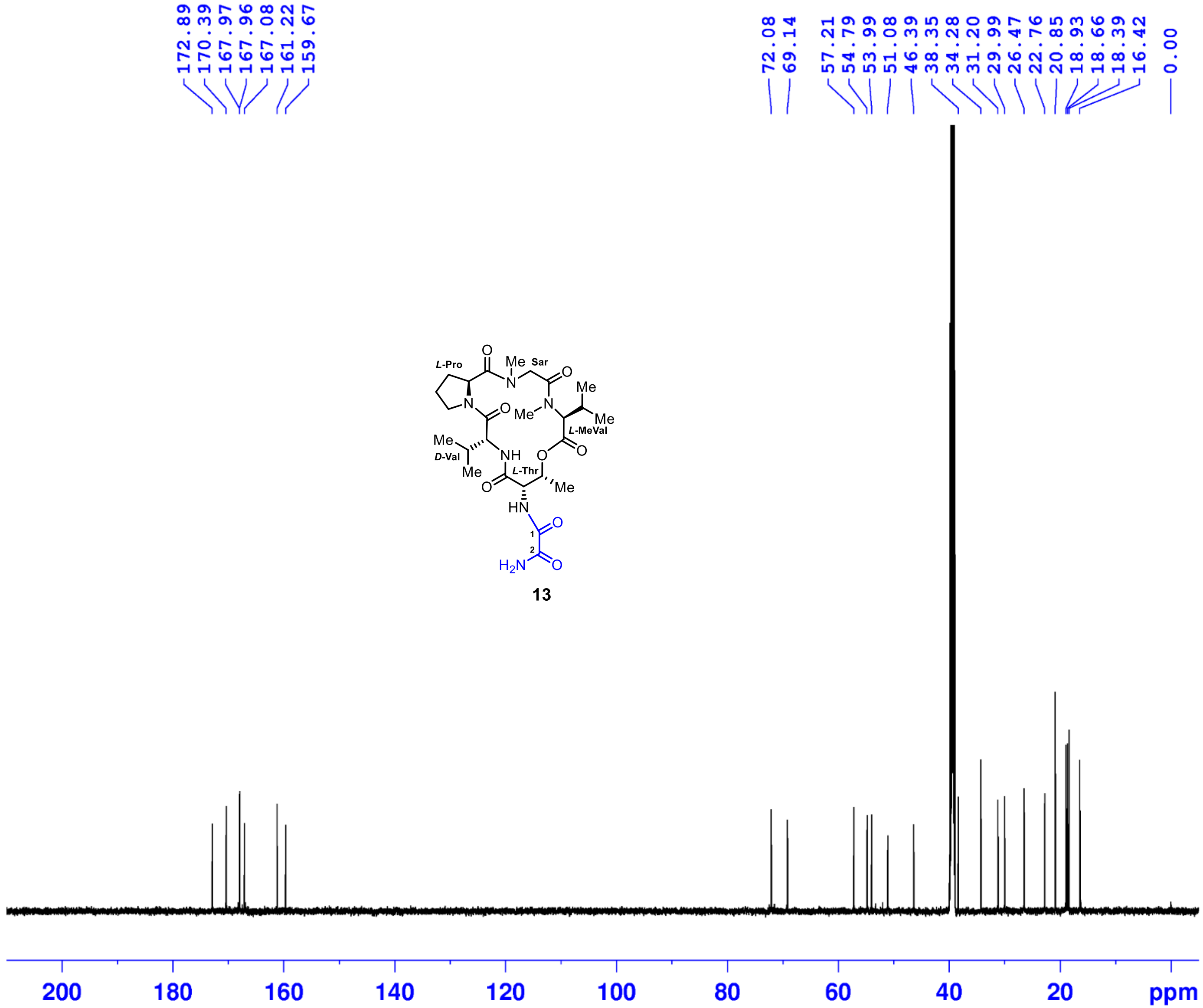

NAME	0620_S_2_2C	
EXPNO	113	
PROCNO	1	
Date_	20250622	
Time	2.38	h
INSTRUM	Avance NEO 600	
PROBHD	Z154705_0139 (	
PULPROG	zgpg30	
TD	65536	
SOLVENT	DMSO	
NS	2048	
DS	4	
SWH	35714.285	Hz
FIDRES	1.089913	Hz
AQ	0.9175540	sec
RG	101	
DW	14.000	usec
DE	6.50	usec
TE	294.6	K
D1	2.00000000	sec
D11	0.03000000	sec
TD0	1	
SFO1	150.9329873	MHz
NUC1	13C	
P0	4.00	usec
P1	12.00	usec
SI	32768	
SF	150.9179821	MHz
WDW	EM	
SSB	0	
LB	1.00	Hz
GB	0	
PC	1.40	

Figure S130-continue

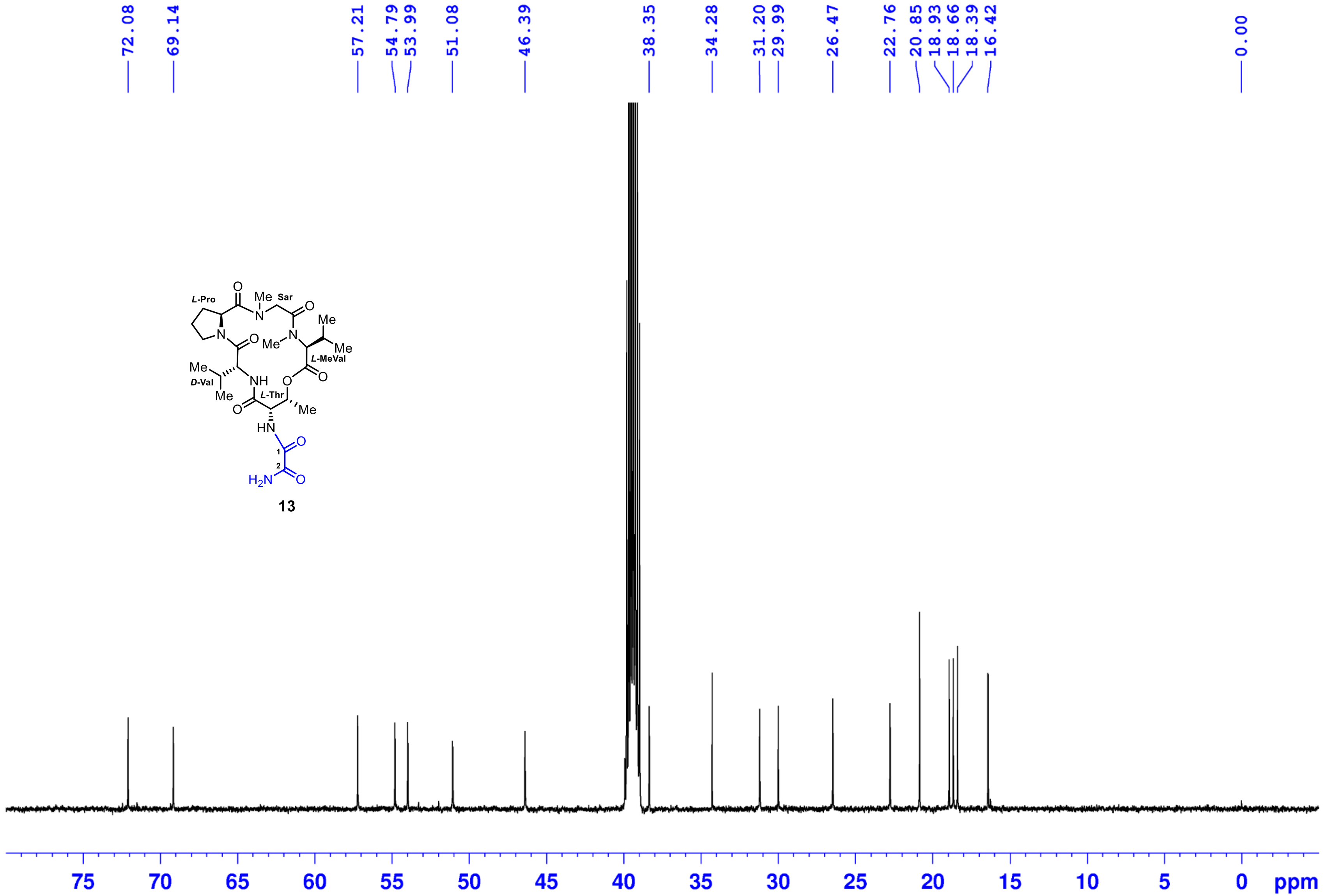

Figure S130-continue

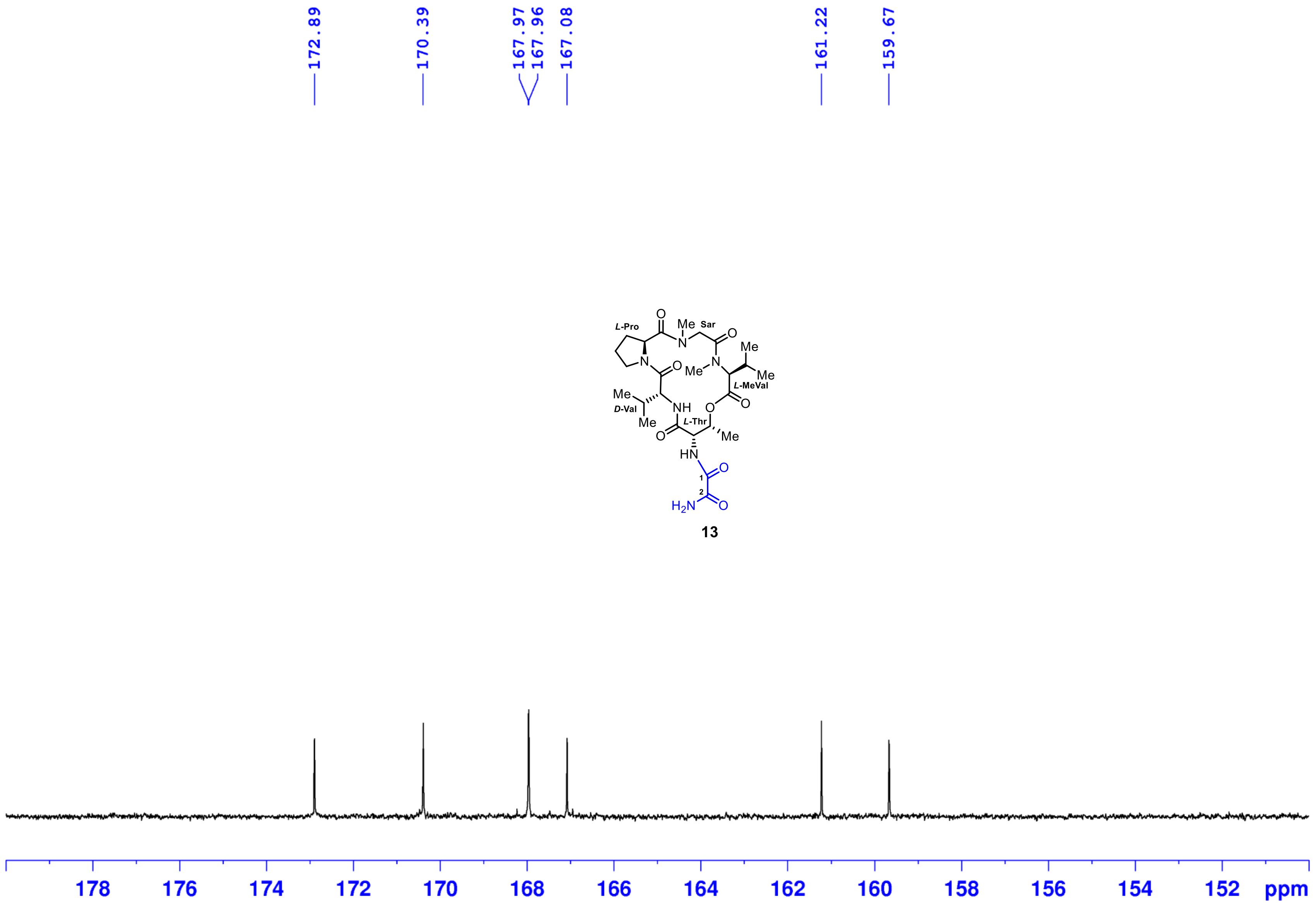

13

Figure S131. DEPT135 (150 MHz) spectrum of compound **13** in DMSO-d_6

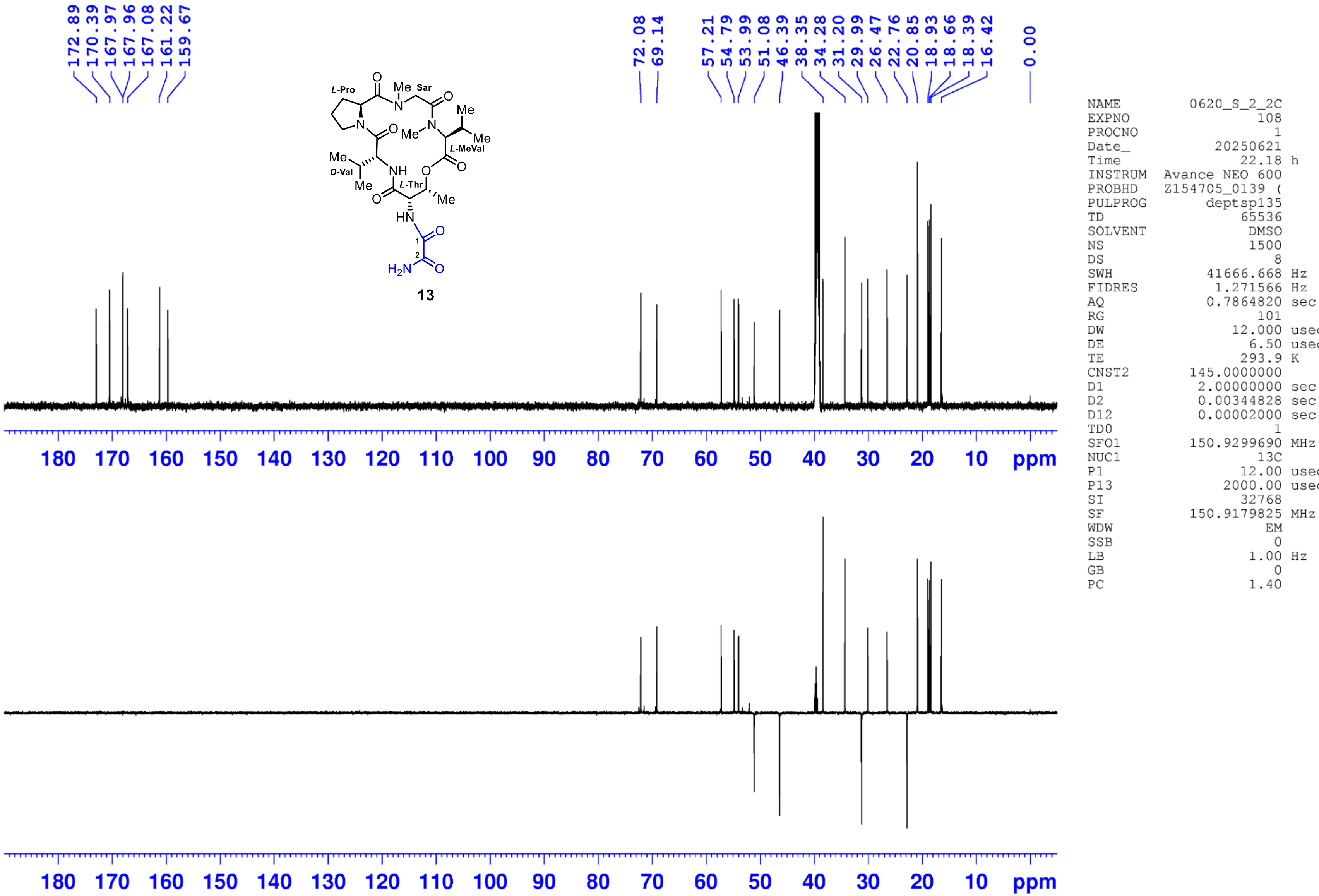

Figure S131-continue

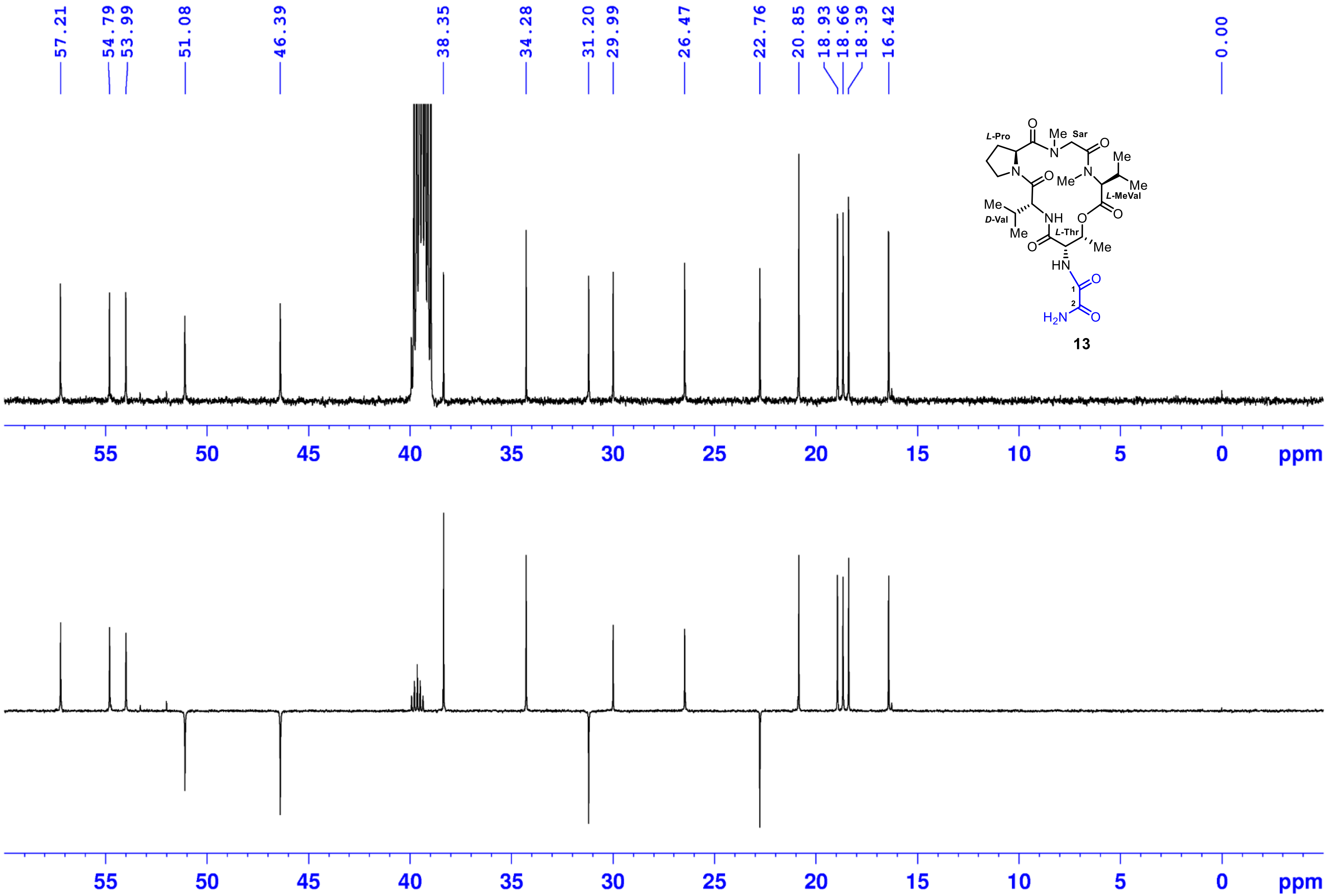

Figure S131-continue

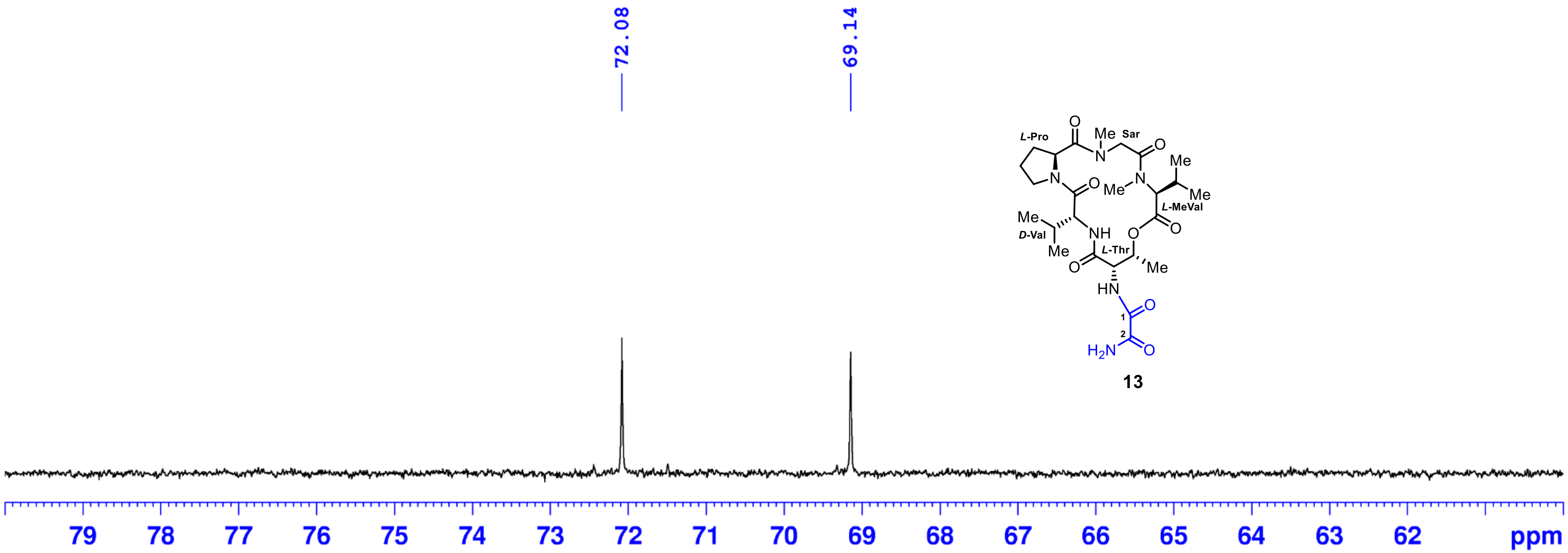

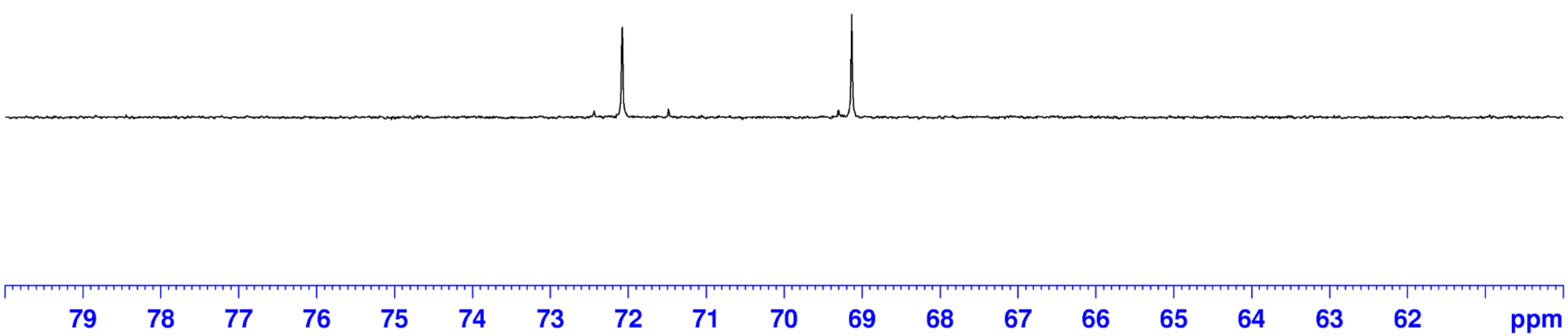

Figure S132. ^{1}H–^{1}H COSY (600 MHz) spectrum of compound **13** in DMSO-d_6

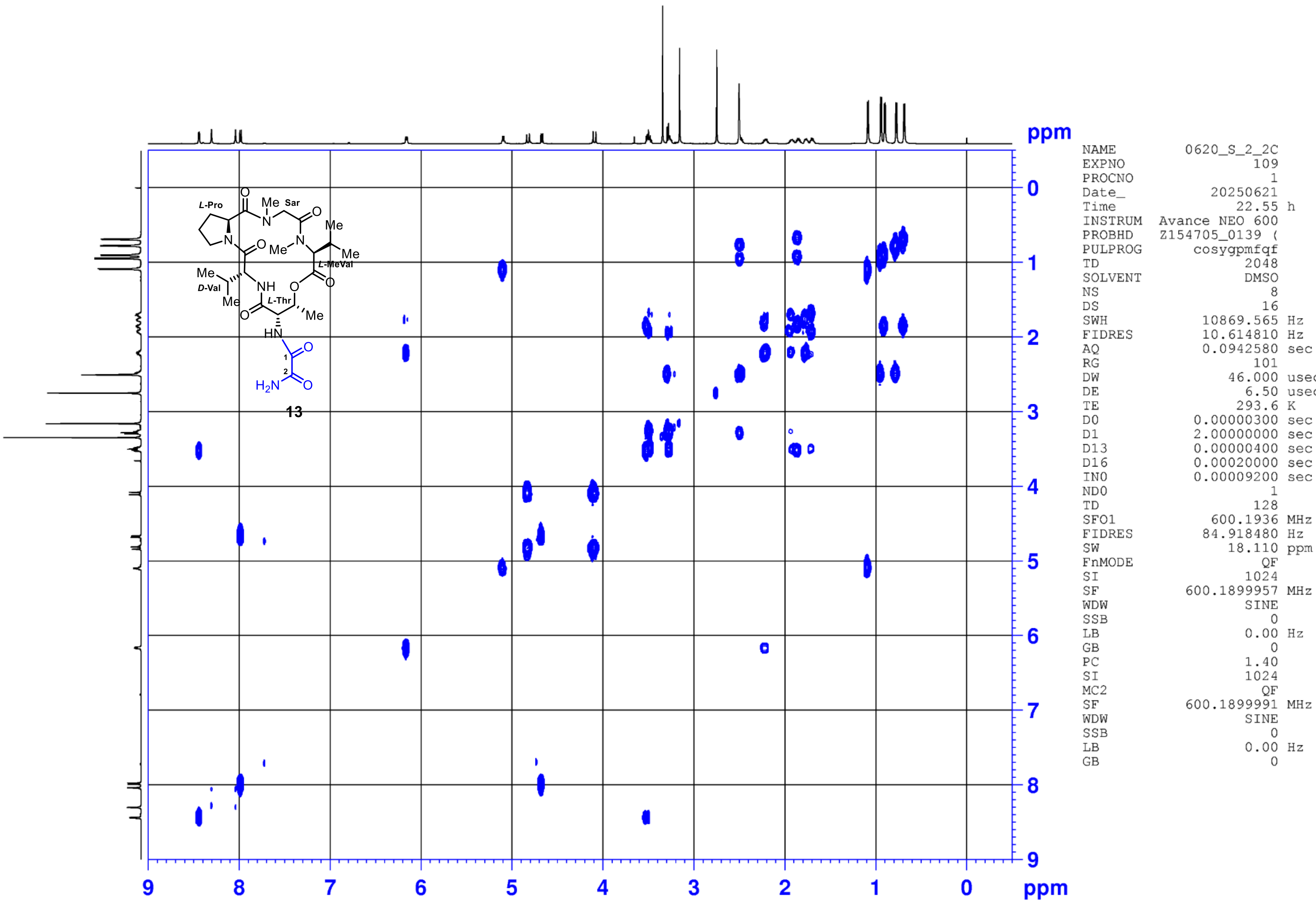

Figure S132-continue

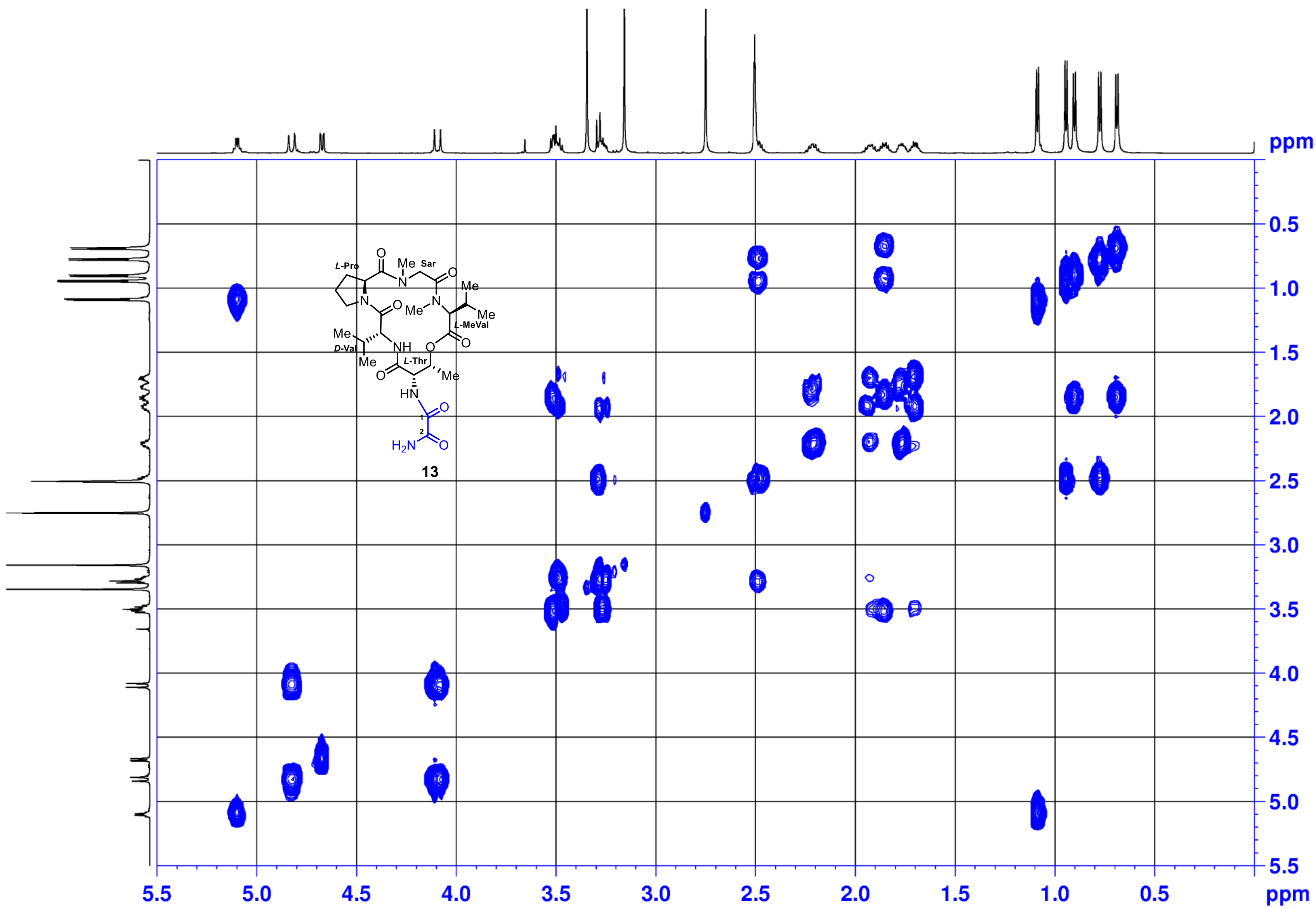

Figure S132-continue

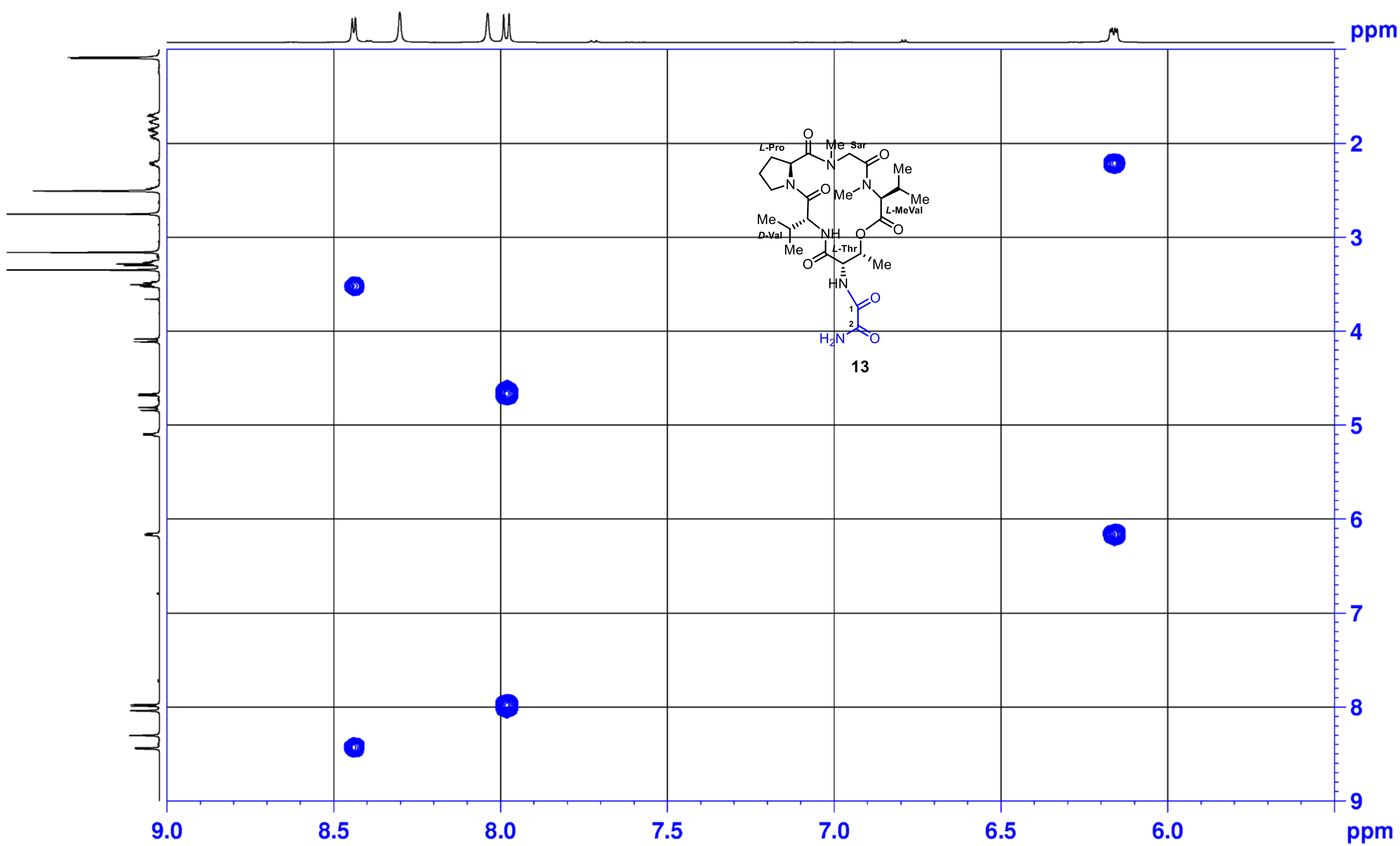

Figure S133. HSQC (600 MHz) spectrum of compound **13** in DMSO-d_6

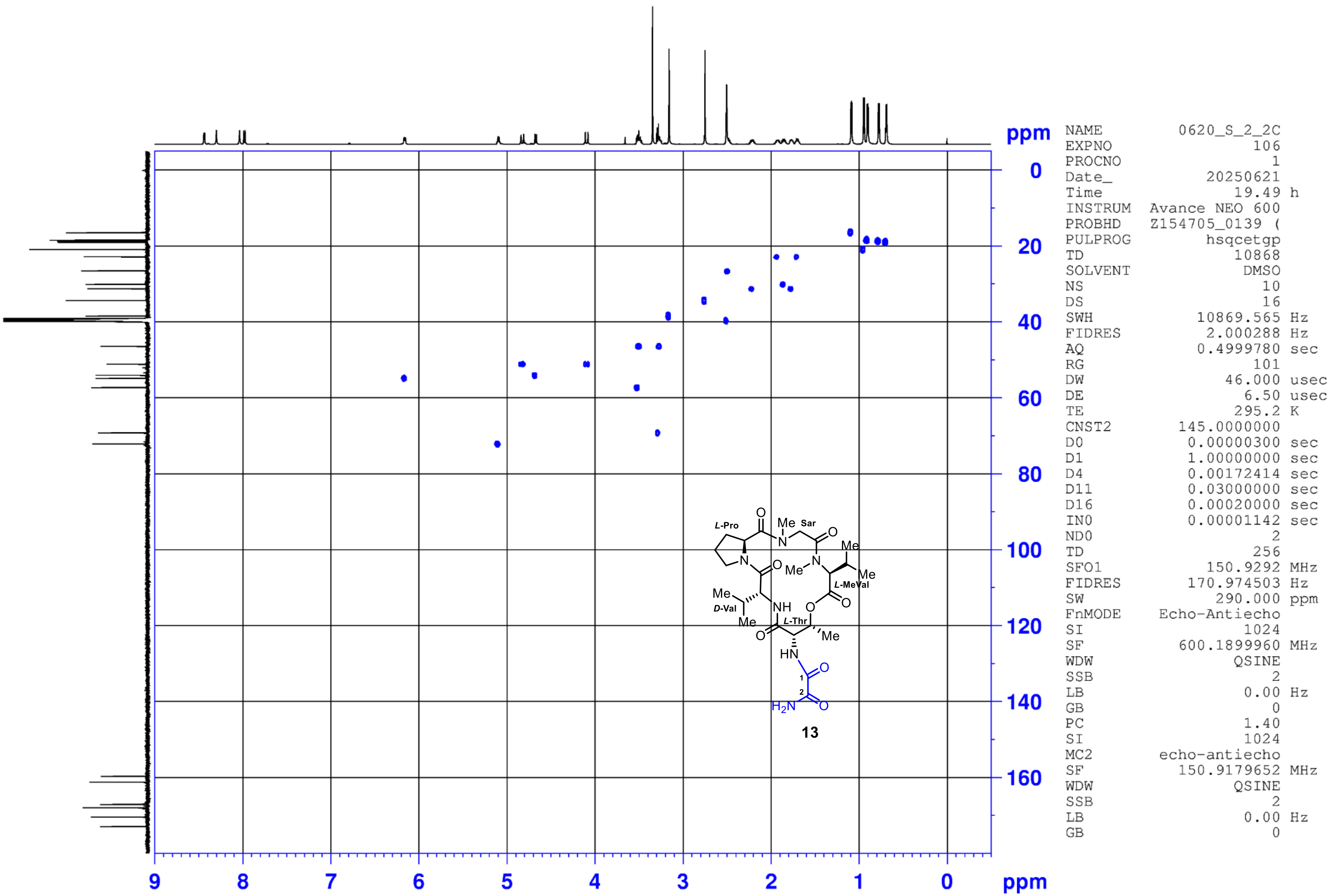

Figure S133-continue

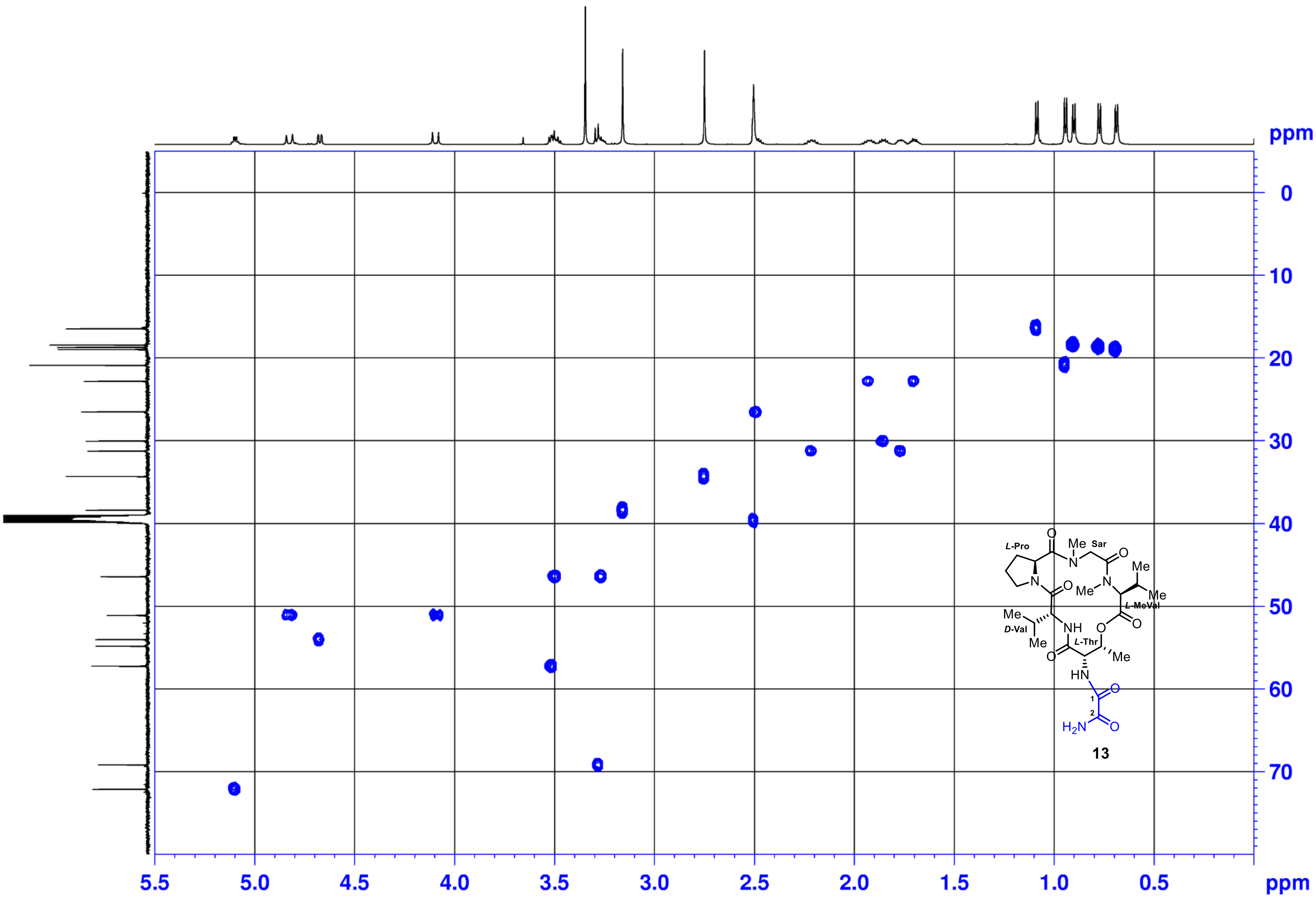

Figure S133-continue

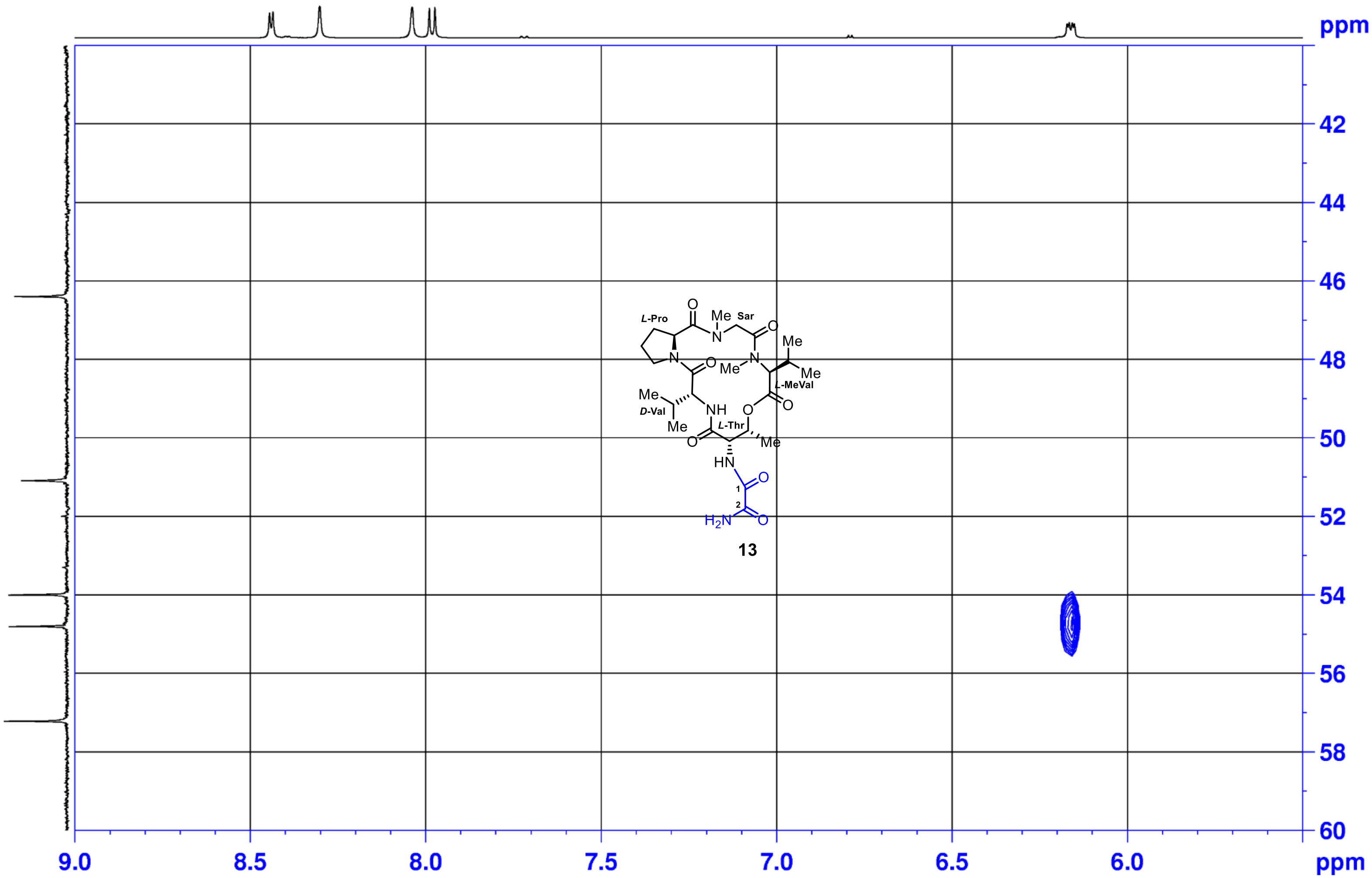

Figure S134. HMBC (600 MHz) spectrum of compound **13** in DMSO-d_6

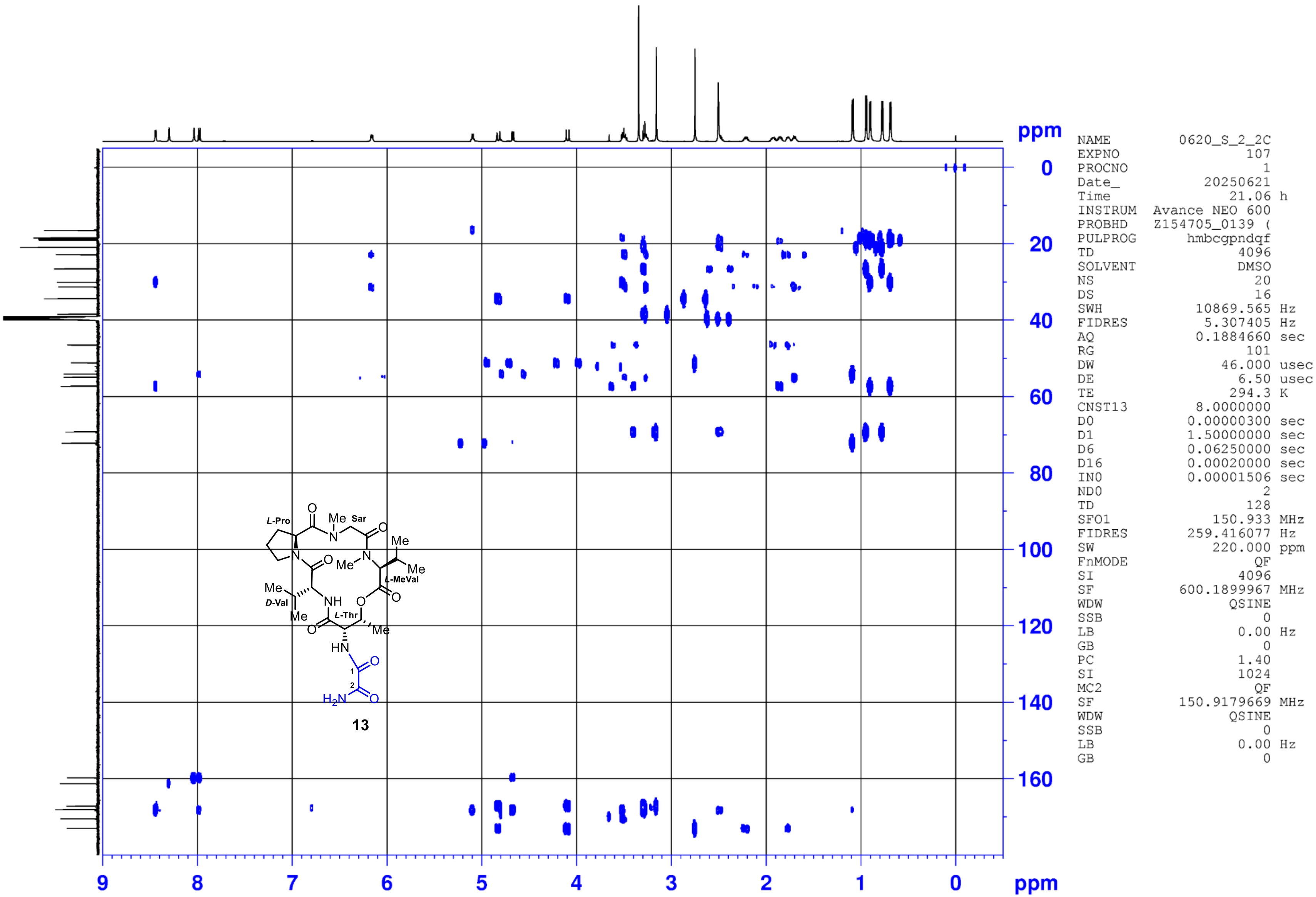

Figure S134-continue

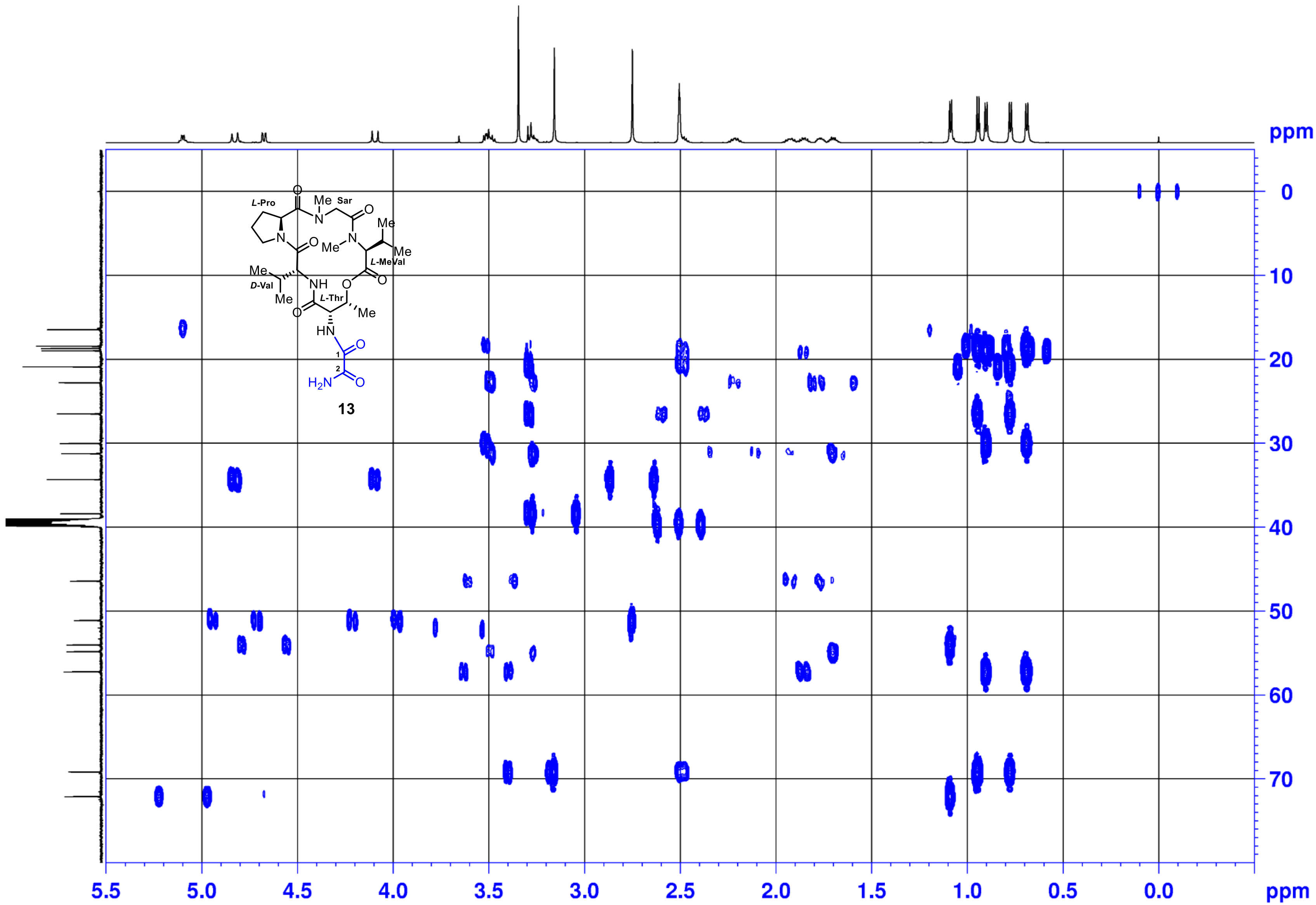

Figure S134-continue

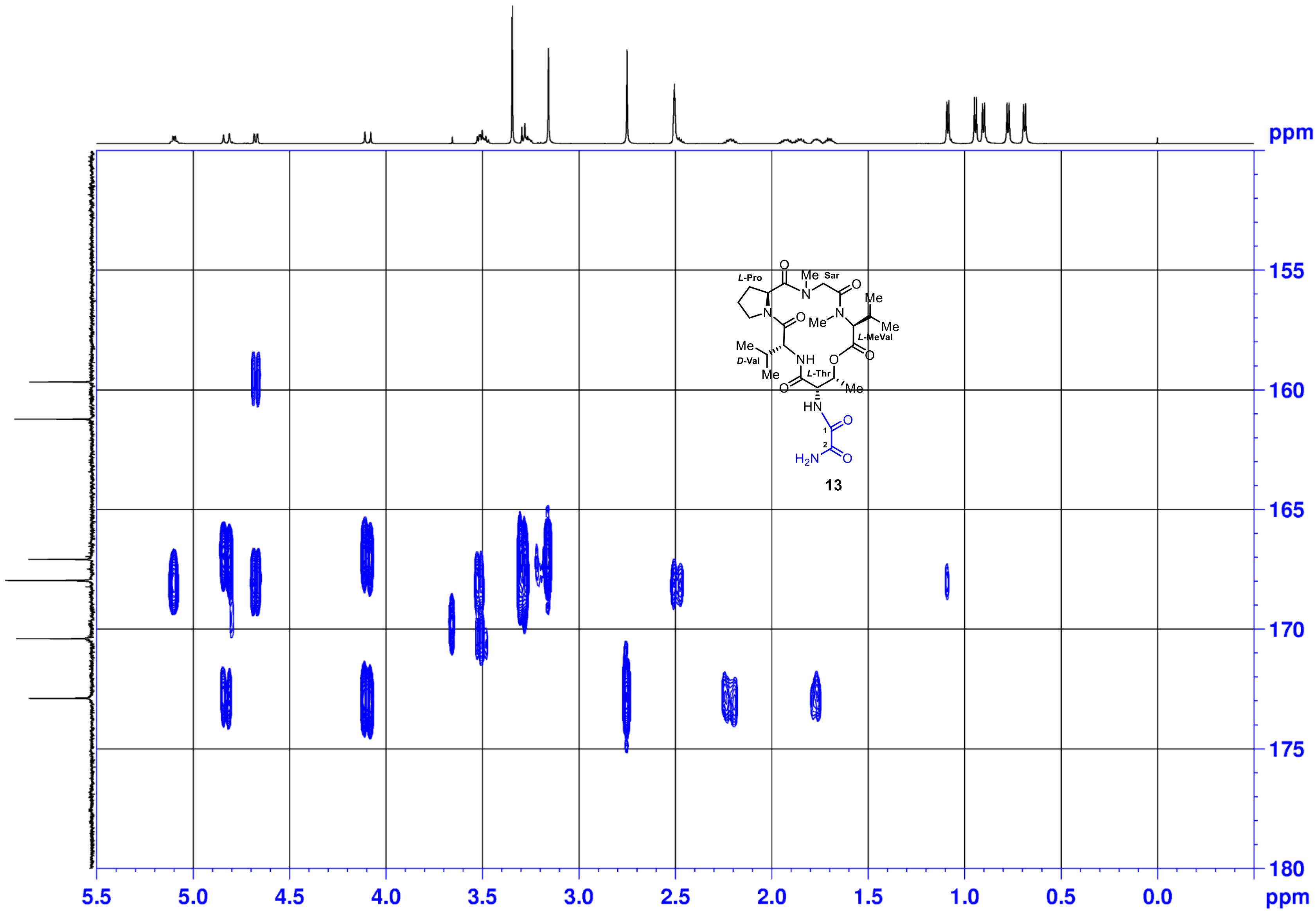

Figure S134-continue

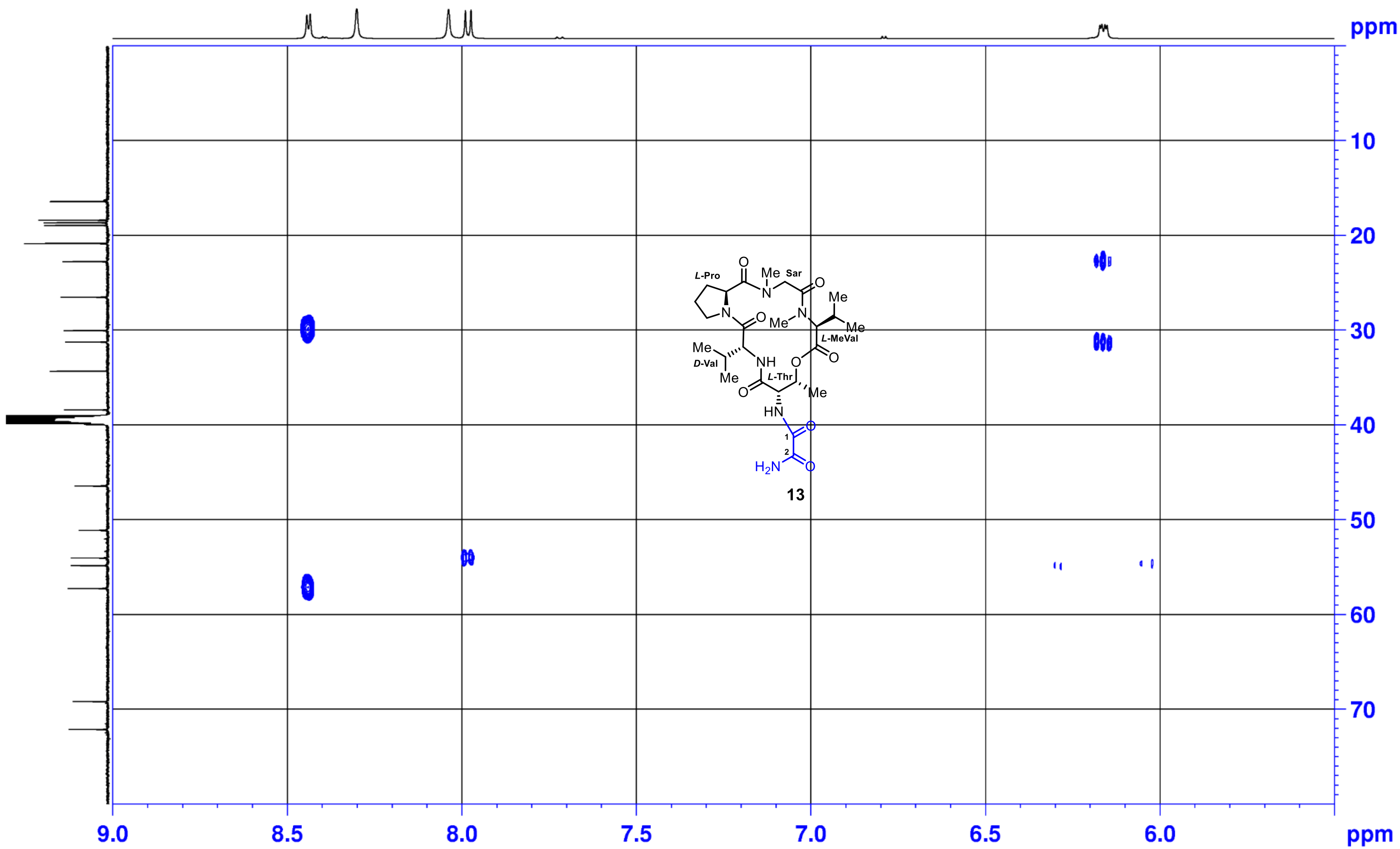

Figure S134-continue

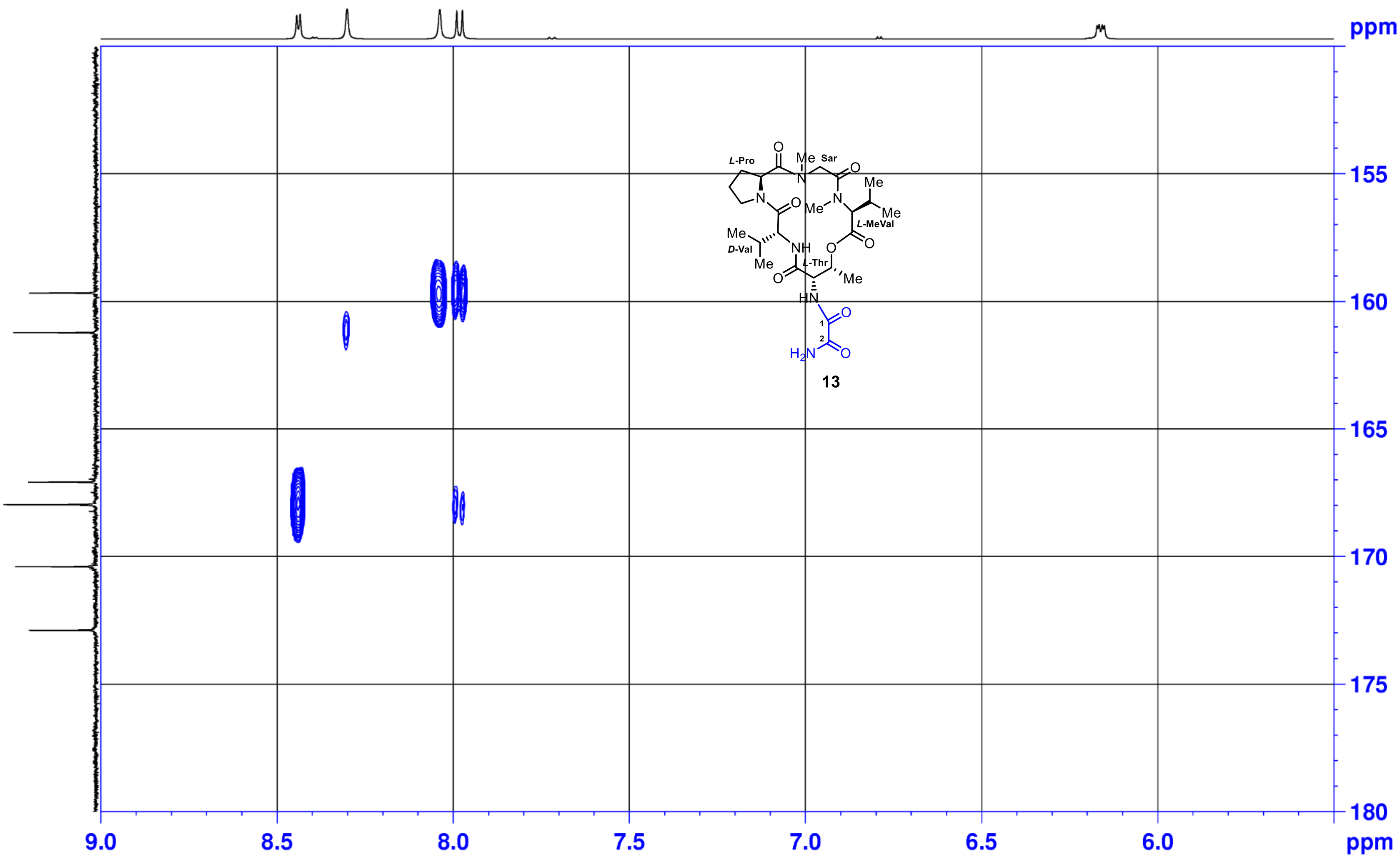

Figure S135. ROESY (600 MHz) spectrum of compound **13** in DMSO-d_6

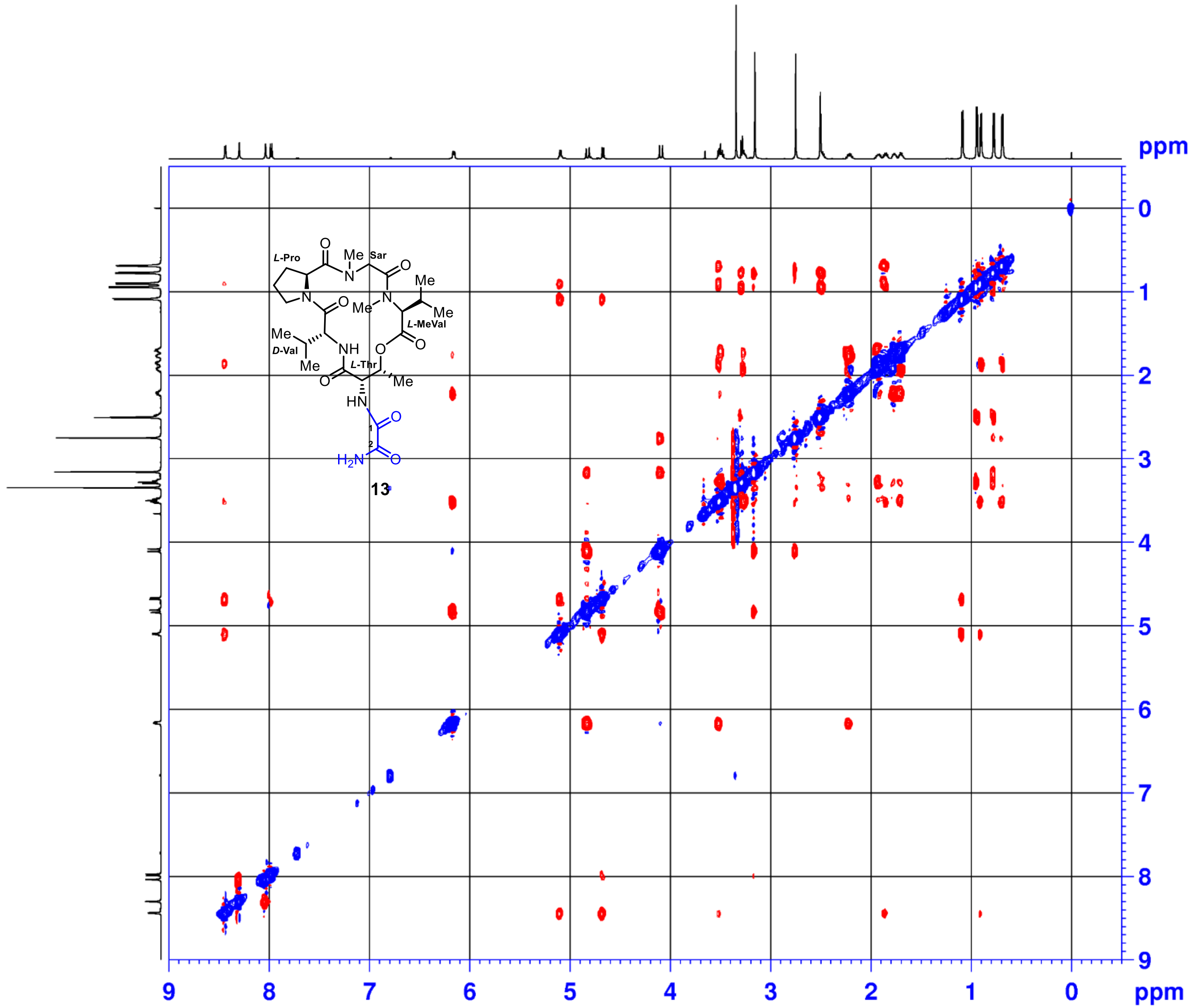

NAME	0620_S_2_2C	
EXPNO	110	
PROCNO	1	
Date_	20250622	
Time	0.55	h
INSTRUM	Avance NEO 600	
PROBHD	Z154705_0139 (	
PULPROG	roesyphpp.2	
TD	2048	
SOLVENT	DMSO	
NS	12	
DS	32	
SWH	13888.889	Hz
FIDRES	13.563368	Hz
AQ	0.0737780	sec
RG	101	
DW	36.000	usec
DE	6.50	usec
TE	293.8	K
D0	0.00002650	sec
D1	2.00000000	sec
D11	0.03000000	sec
D12	0.00002000	sec
IN0	0.00007373	sec
L4	556	
P15	200000.00	usec
ND0	1	
TD	256	
SFO1	600.1928	MHz
FIDRES	52.985771	Hz
SW	22.600	ppm
FnMODE	States-TPPI	
SI	1024	
SF	600.1899930	MHz
WDW	QSINE	
SSB	2	
LB	0.00	Hz
GB	0	
PC	1.00	
SI	1024	
MC2	States-TPPI	
SF	600.1899923	MHz
WDW	QSINE	
SSB	2	
LB	0.00	Hz
GB	0	

Figure S135-continue

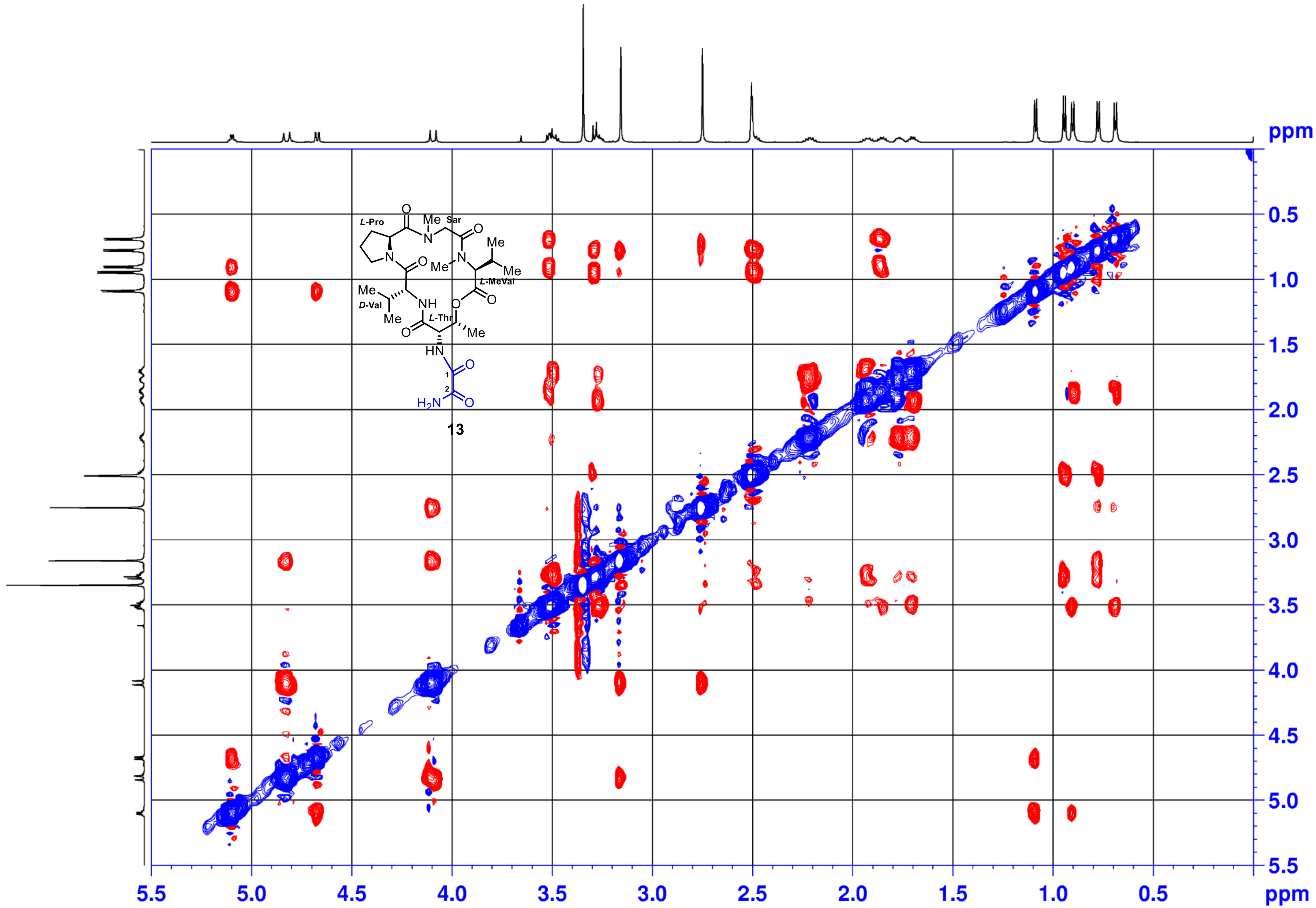

Figure S135-continue

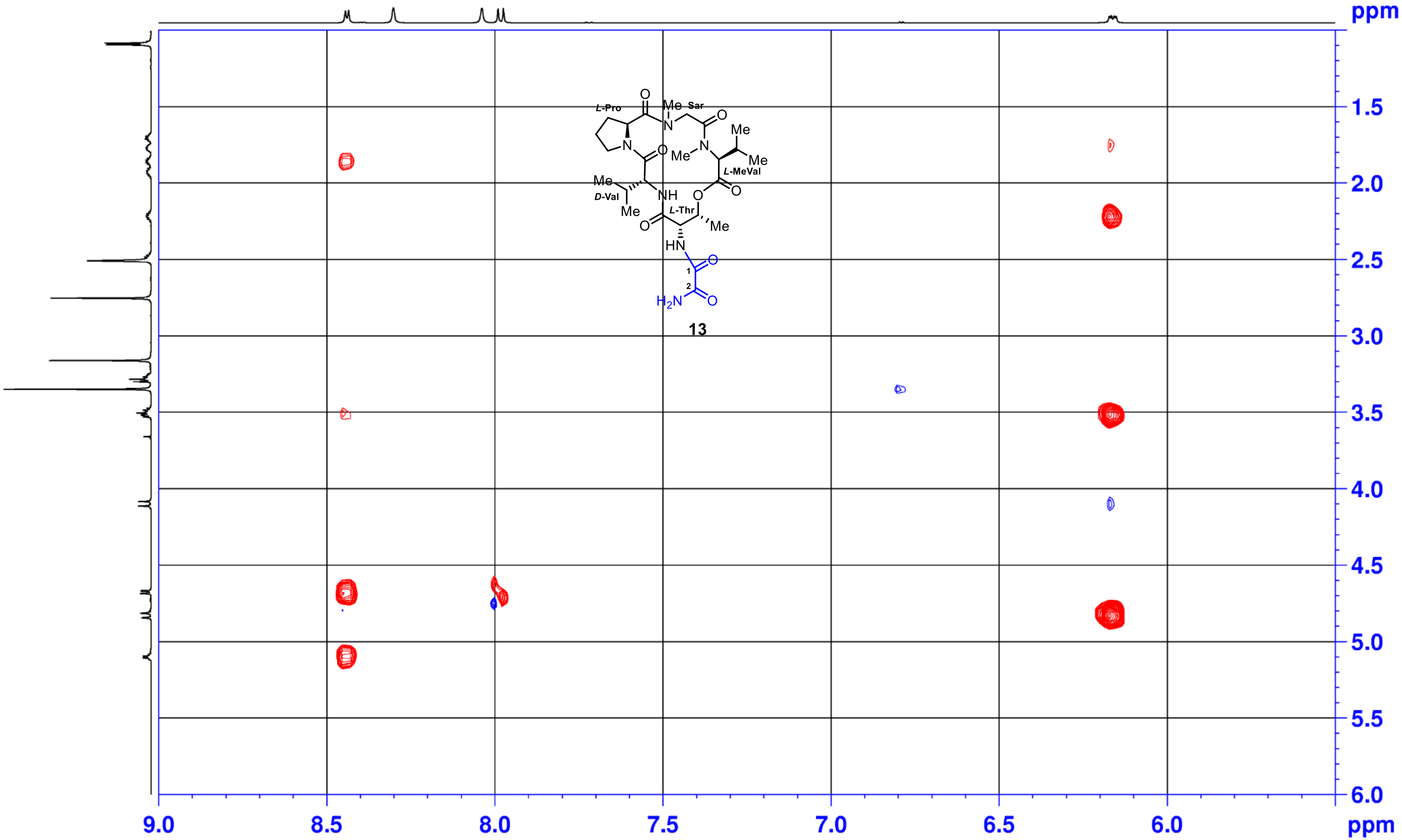

Figure S136. Marfey's analysis of compound **13** and standard amino acids

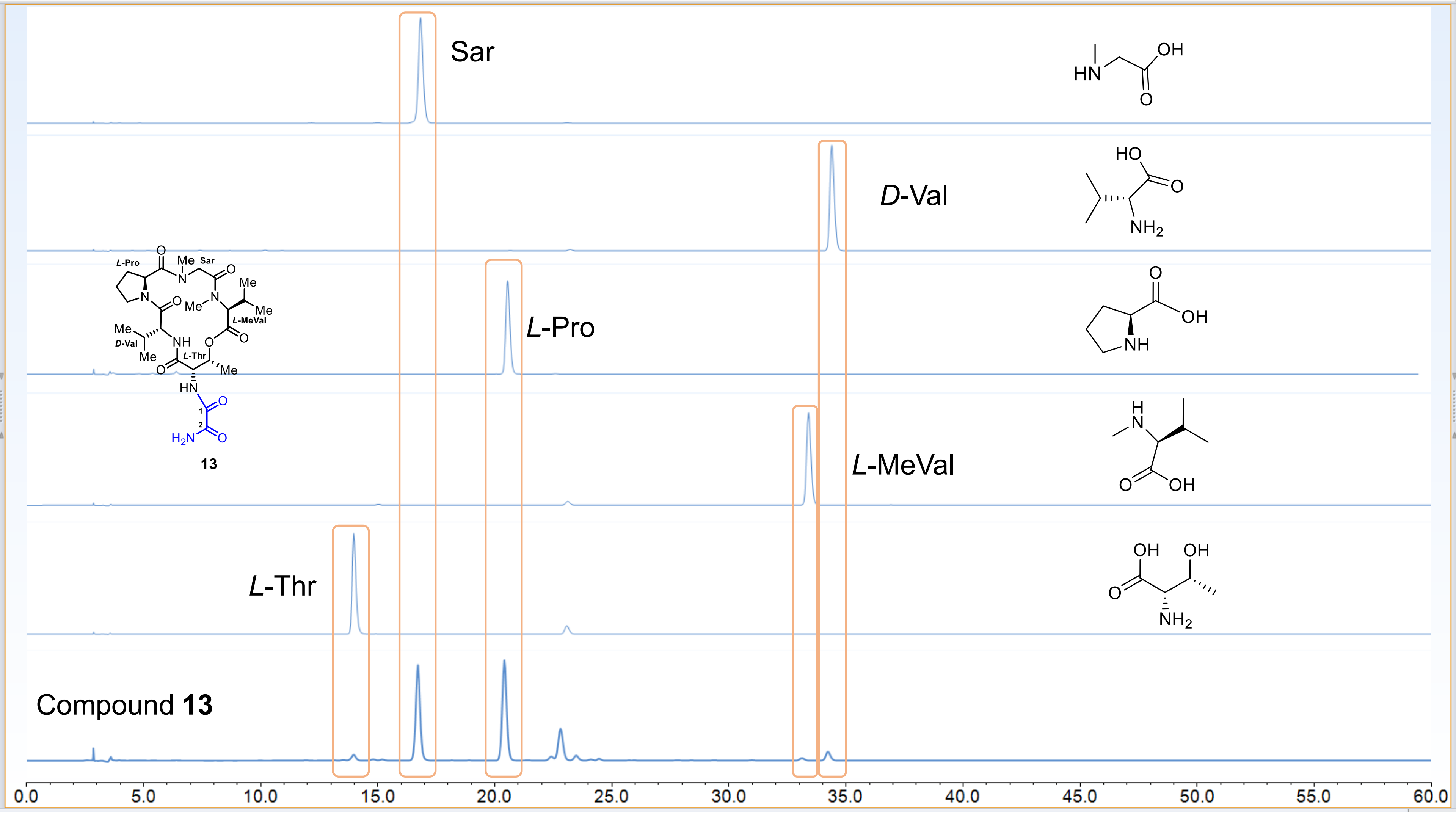

Figure S137. UV spectrum of compound **14**

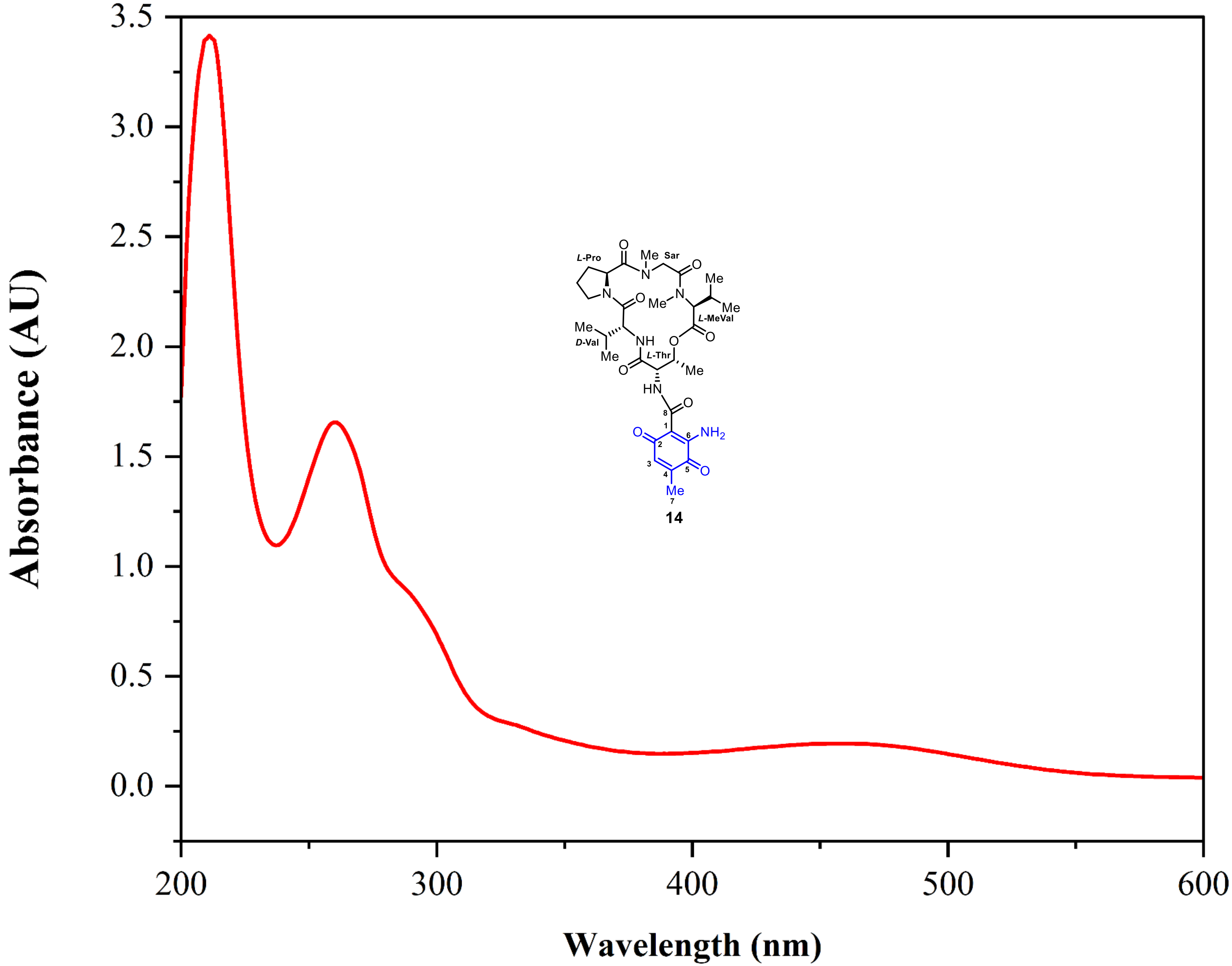

Figure S138. HR-ESIMS for compound **14**

Compound Spectrum SmartFormula Report

Analysis Info

Analysis	D:\Data\SCSIO Metabonomics data\MS\202508\panghe_S-4-2_pos_P1-F-2_801.d
Method	Tune_pos_middle_avg_100-2000_calibration_1.5m.m
Sample	panghe_S-4-2_pos
Comment	
Acquisition Date	2025-08-19 11:09:11
Operator	SCSIO User
Instrument	timsTOF Pro 2 1875087.11040

Acquisition Parameter

Source Type	ESI	Ion Polarity	Positive	Set Nebulizer	0.3 Bar
Scan Begin	100 m/z	Set Capillary	3500 V	Set Dry Heater	200 °C
Scan End	2000 m/z	Set Multipole RF	500.0 Vpp	Set Dry Gas	3.5 l/min
		Set Collision Cell RF	2000.0 Vpp	ICC active	Off
		IMS Active	Off	ICC Target	2000000 cts.
		IMS Collision Cell In	300.0 V		

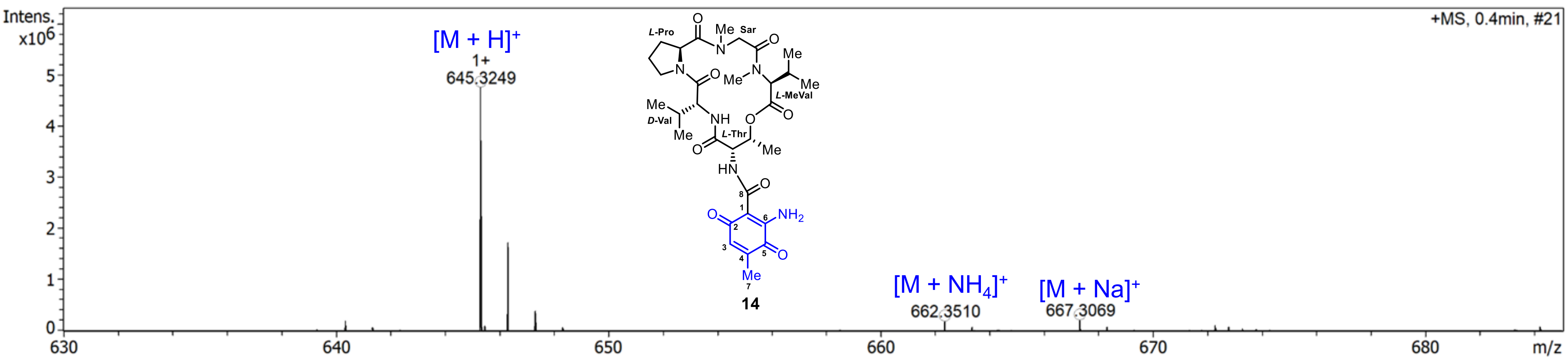

Meas. m/z	#	Ion Formula	Score	m/z	err [mDa]	err [ppm]	mSigma	rdb (neutral)	e⁻ Conf	Adduct
645.324936	1	C31H45N6O9	100.00	645.324253	-0.7	-1.1	1.1	13.0	even	M+H
662.351040	1	C31H48N7O9	100.00	662.350803	-0.2	-0.4	8.2	12.0	even	M+H
667.306890	1	C31H44N6NaO9	100.00	667.306198	-0.7	-1.0	10.8	13.0	even	M+H
1289.641971	1	C62H89N12O18	100.00	1289.641230	-0.7	-0.6	4.5	25.0	even	M+H
1306.668318	1	C62H92N13O18	100.00	1306.667780	-0.5	-0.4	3.2	24.0	even	M+H
1311.623736	1	C62H88N12NaO18	100.00	1311.623175	-0.6	-0.4	13.2	25.0	even	M+H

Figure S139. ^{1}H NMR (600 MHz) spectrum of compound **14** in DMSO-d_6

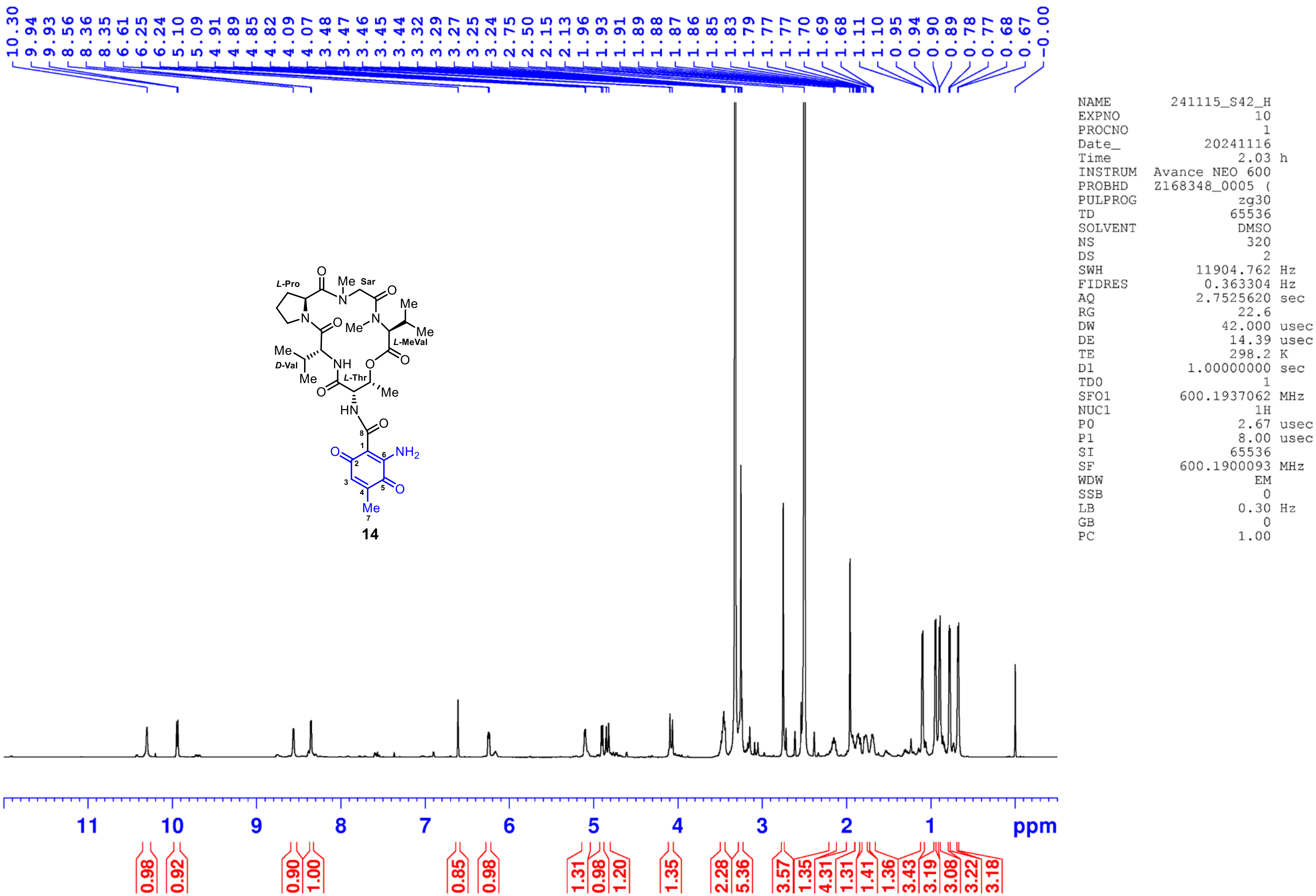

Figure S139-continue

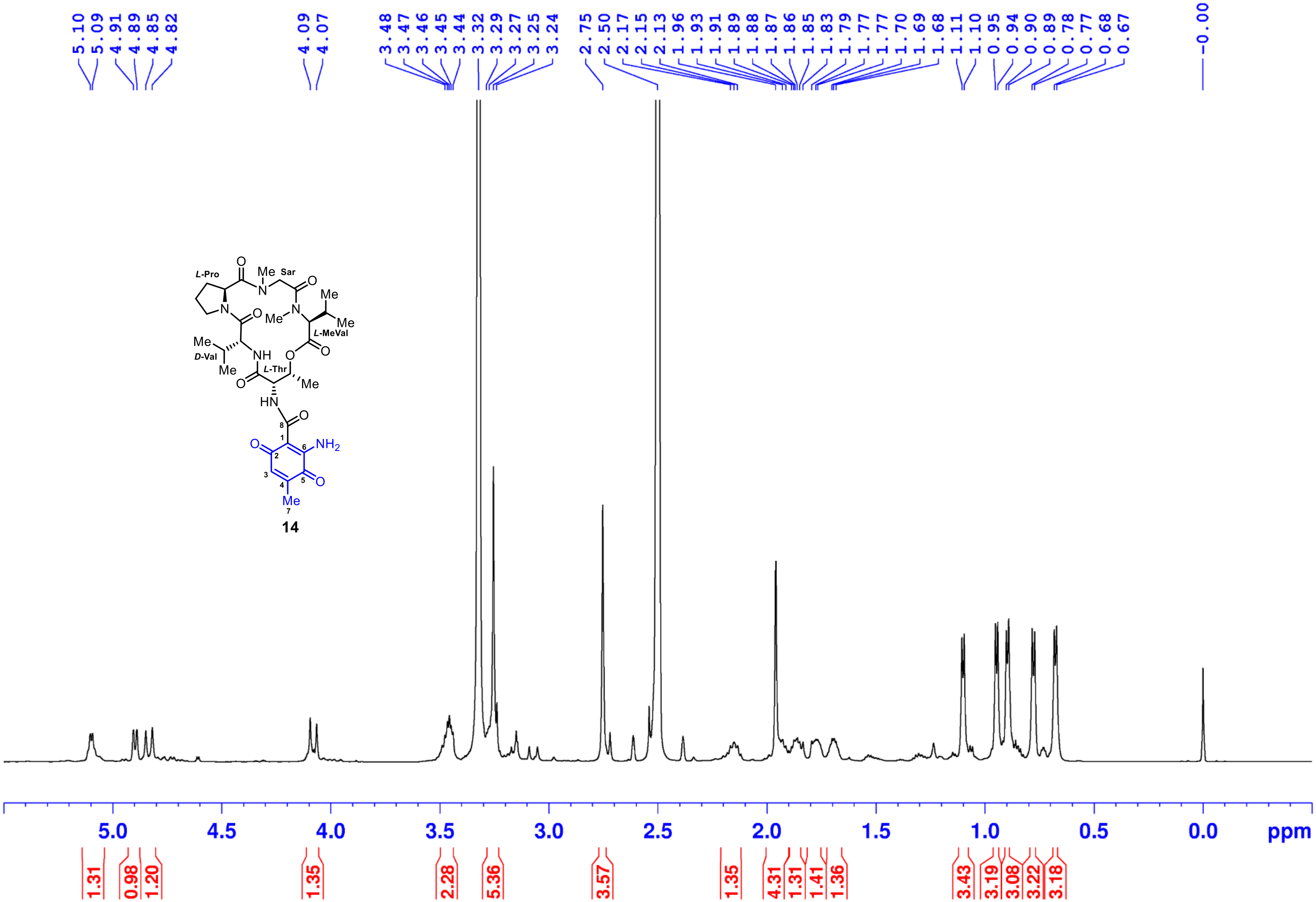

Figure S139-continue

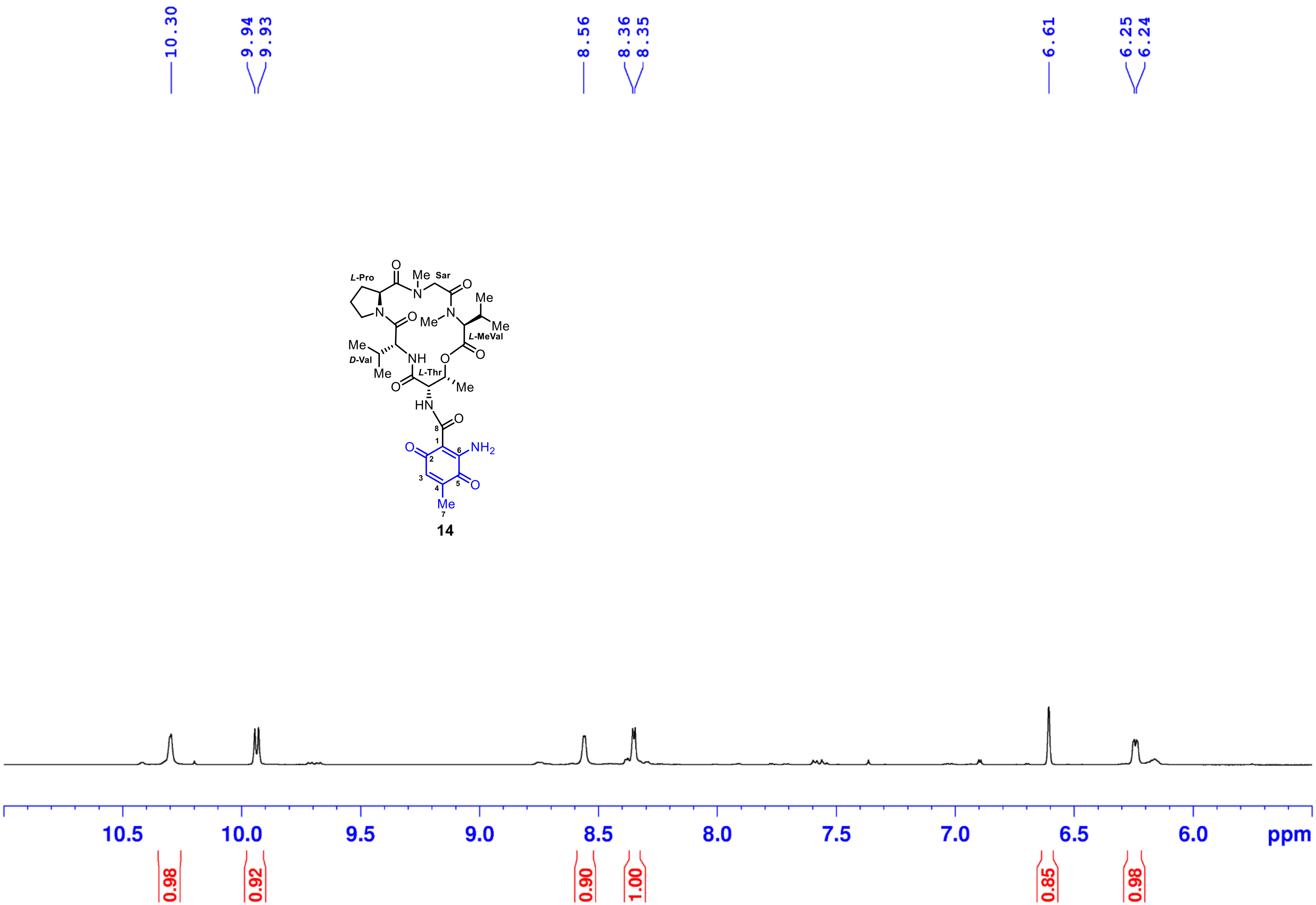

Figure S140. ^{13}C NMR (150 MHz) spectrum of compound **14** in DMSO-d_6

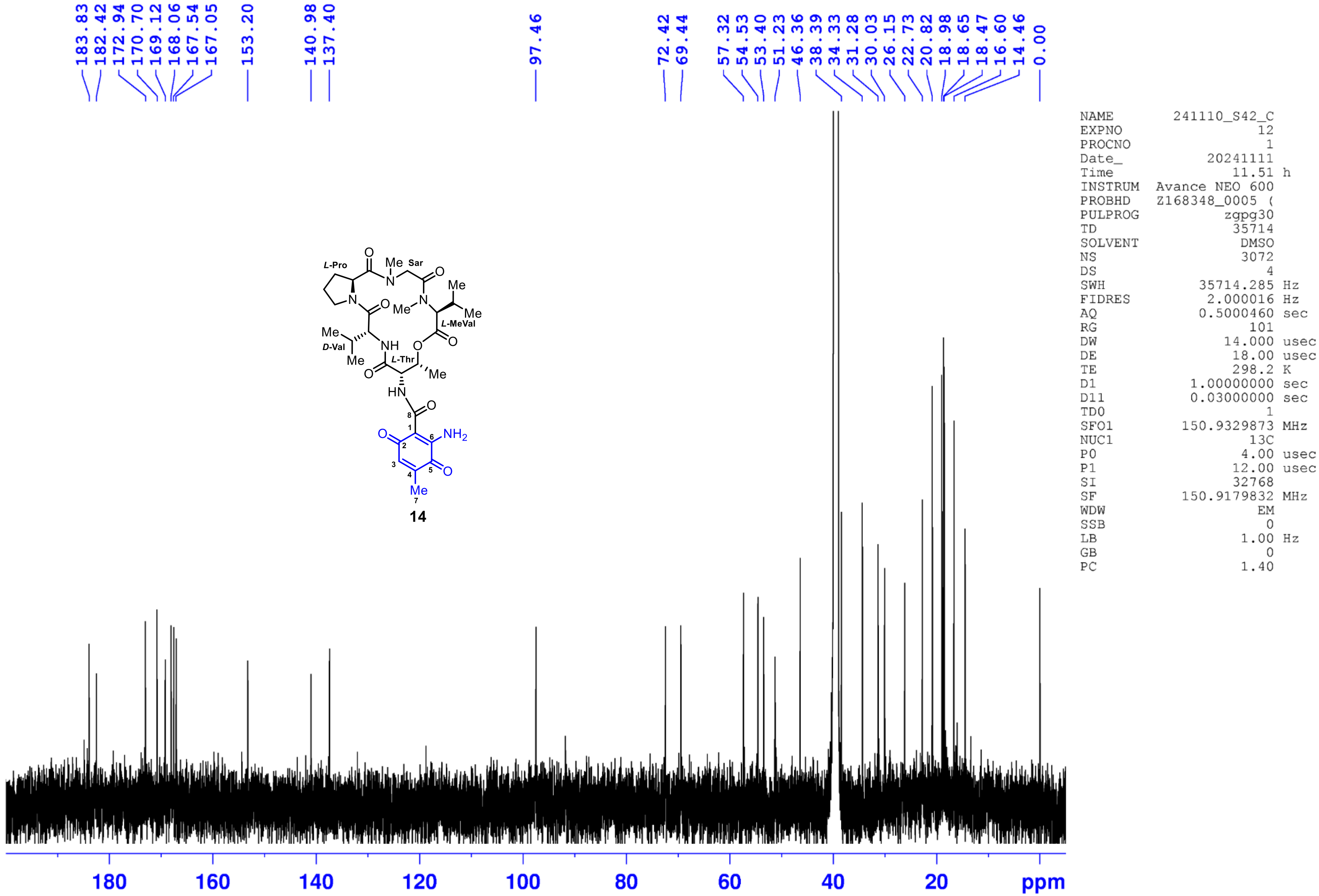

Figure S140-continue

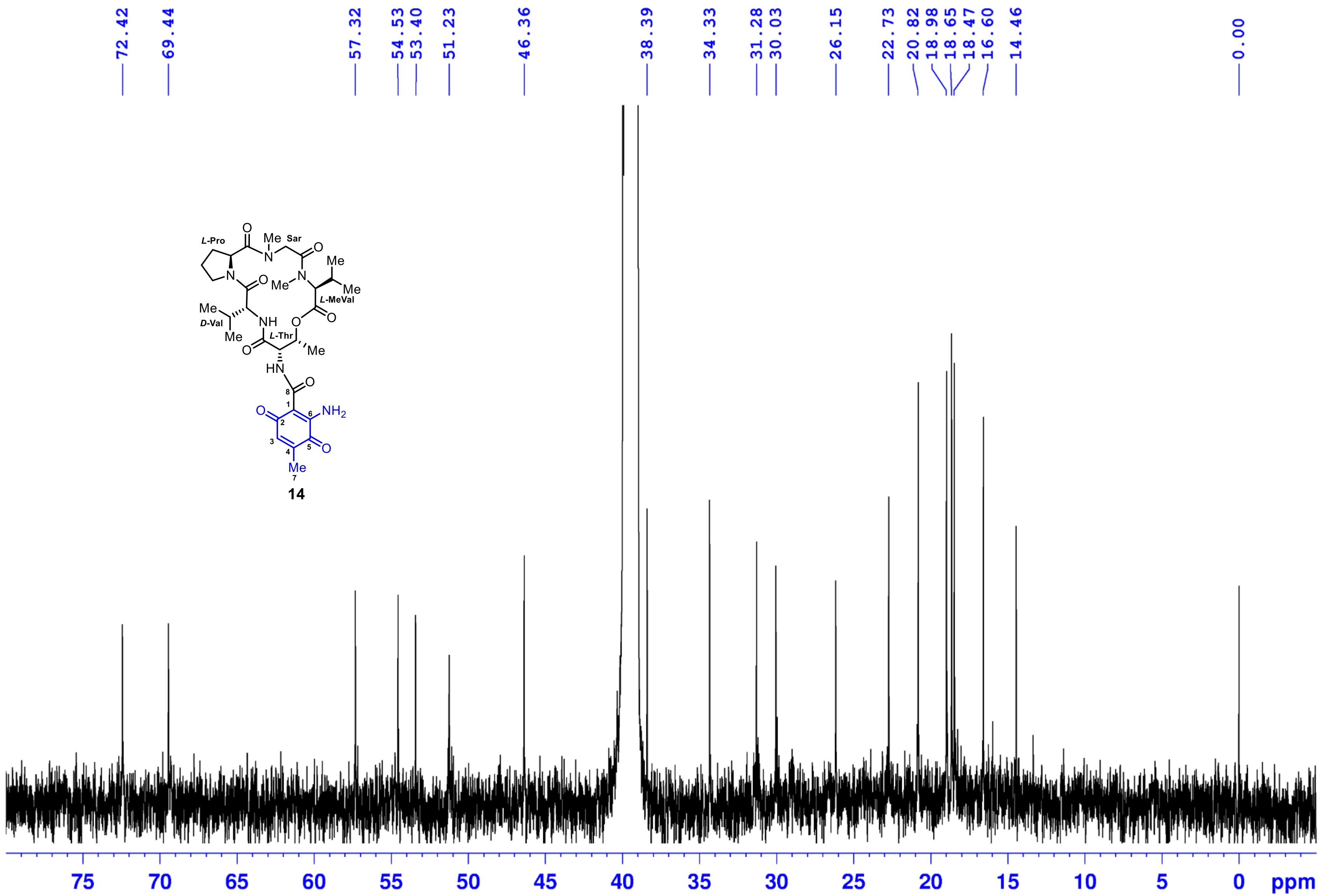

Figure S140-continue

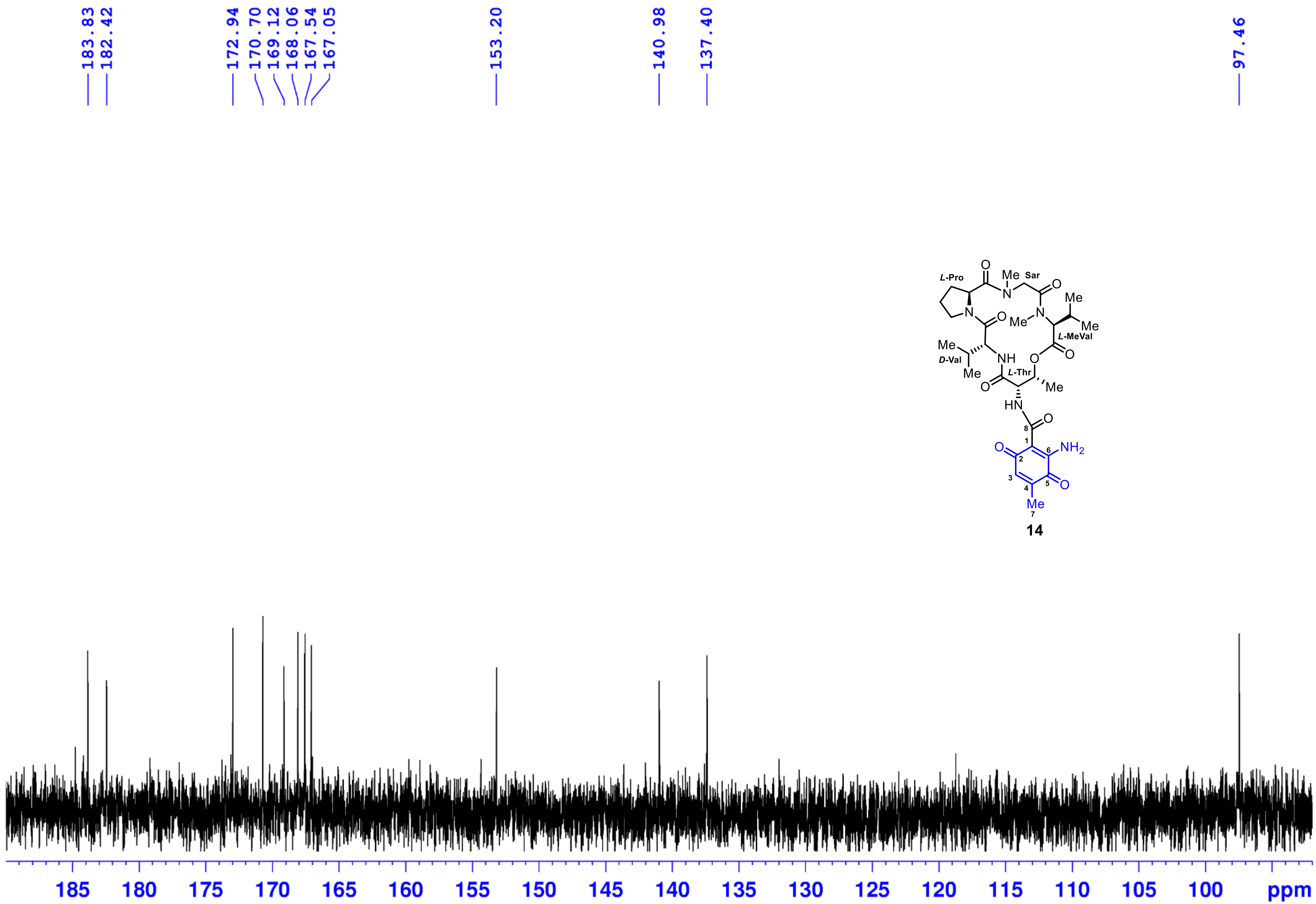

Figure S141. DEPT135 (150 MHz) spectrum of compound **14** in DMSO-d_6

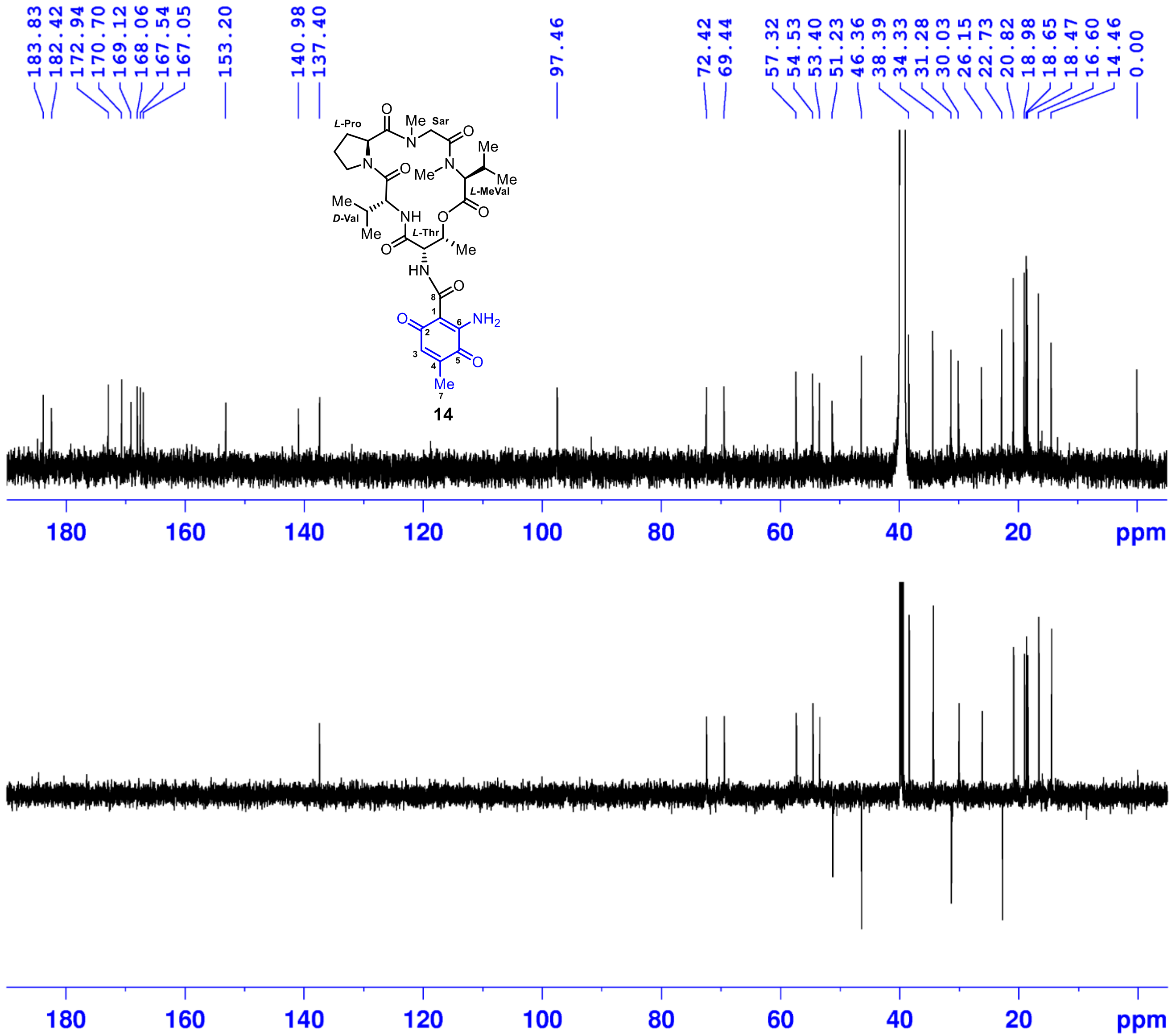

NAME	241115_S42_135C
EXPNO	14
PROCNO	1
Date_	20241116
Time	3.45 h
INSTRUM	Avance NEO 600
PROBHD	Z168348_0005 (
PULPROG	deptsp135
TD	65536
SOLVENT	DMSO
NS	1024
DS	8
SWH	41666.668 Hz
FIDRES	1.271566 Hz
AQ	0.7864820 sec
RG	101
DW	12.000 usec
DE	18.00 usec
TE	298.2 K
CNST2	145.0000000
D1	2.00000000 sec
D2	0.00344828 sec
D12	0.00002000 sec
TD0	1
SFO1	150.9299690 MHz
NUC1	13C
P1	12.00 usec
P13	2000.00 usec
SI	32768
SF	150.9179842 MHz
WDW	EM
SSB	0
LB	1.00 Hz
GB	0
PC	1.40

Figure S141-continue

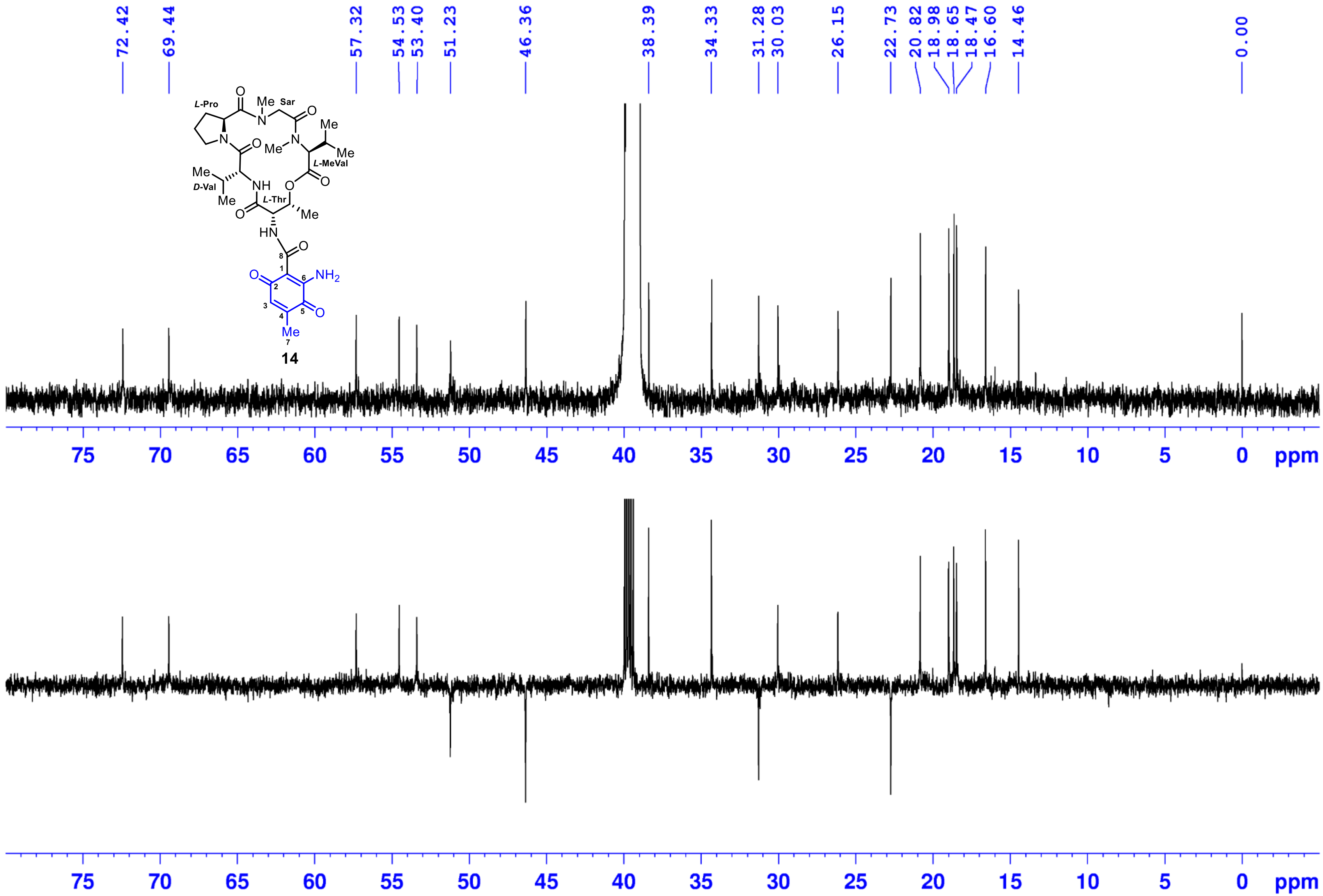

Figure S141-continue

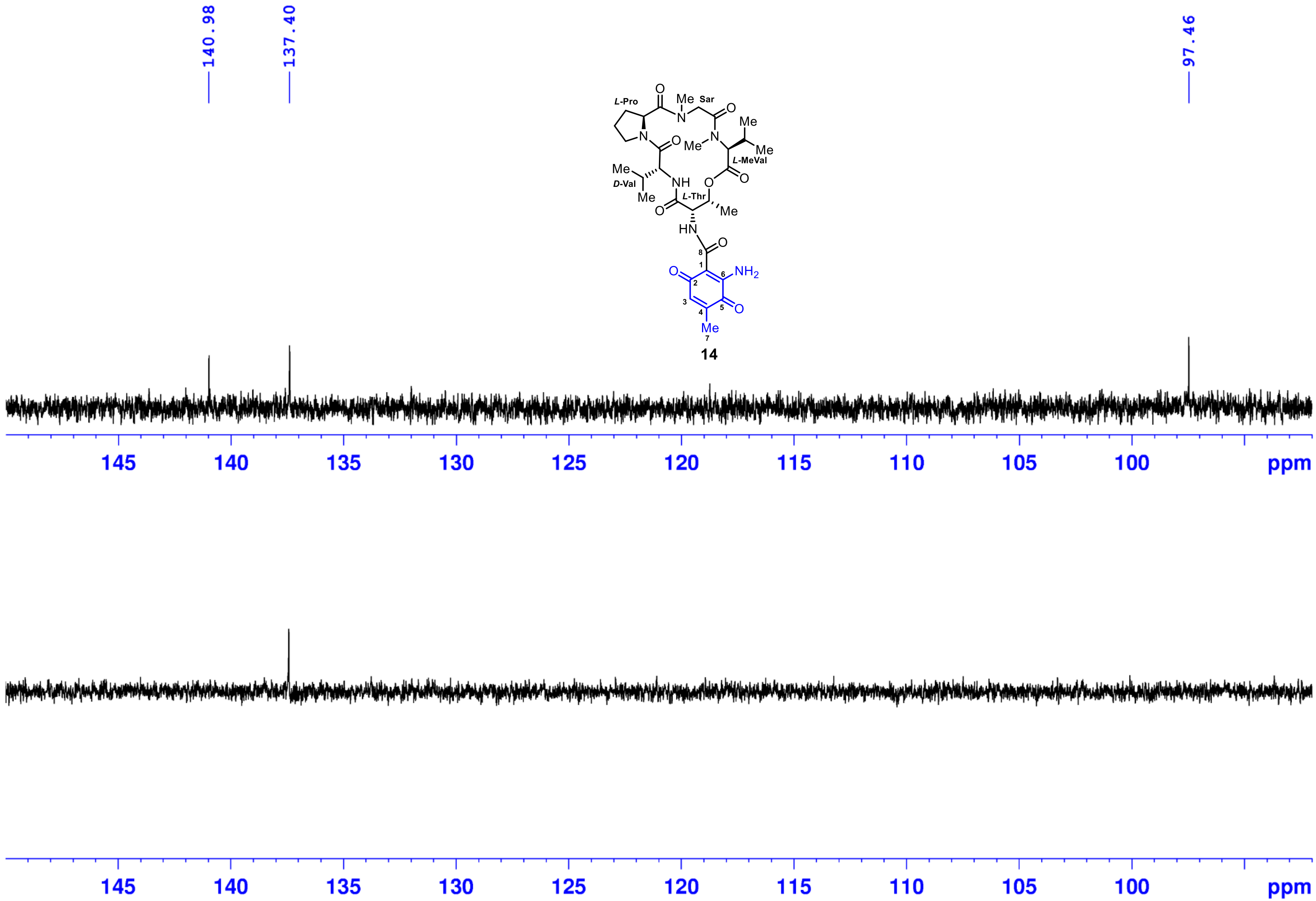

Figure S142. ^{1}H–^{1}H COSY (600 MHz) spectrum of compound **14** in DMSO-d_6

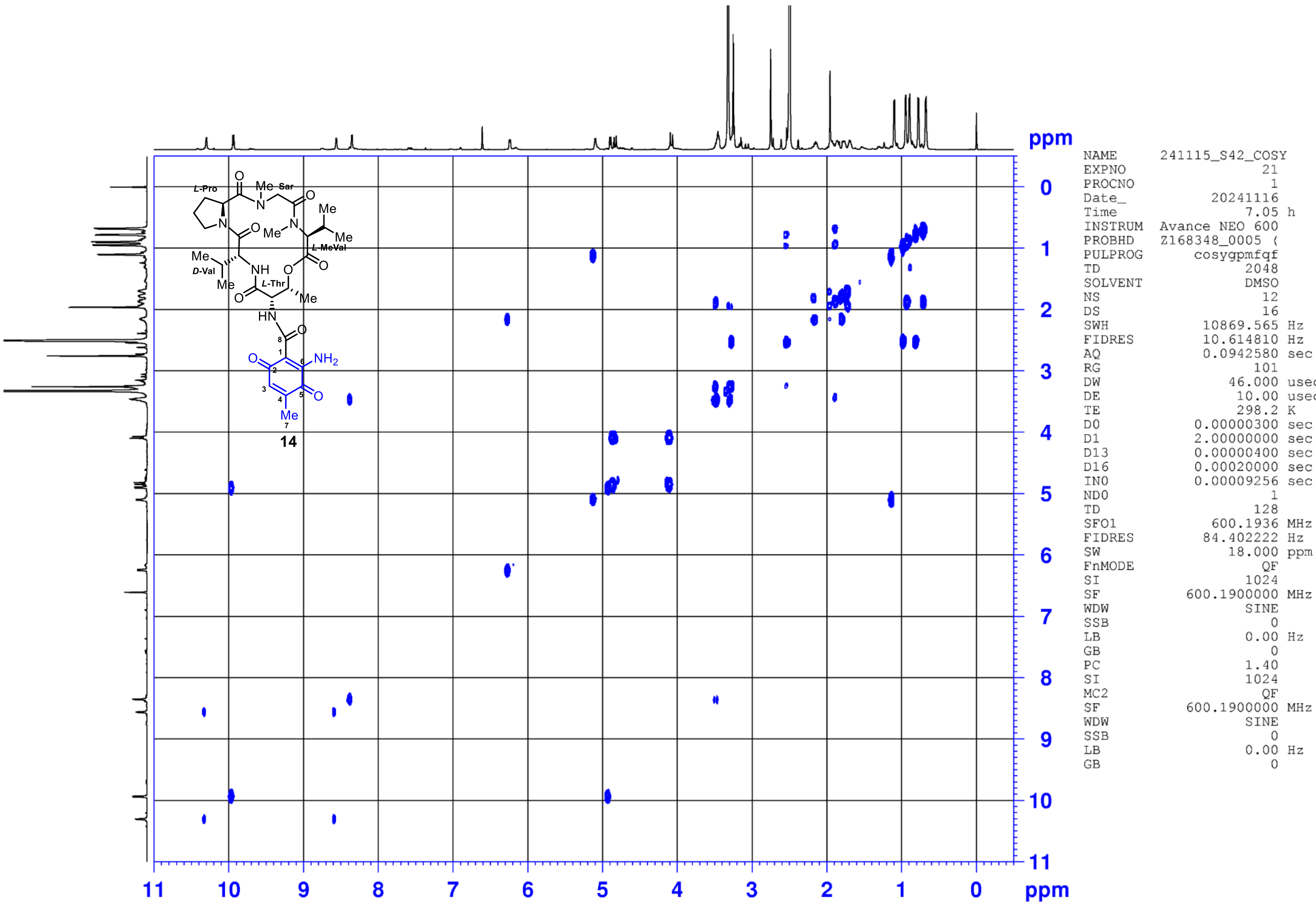

Figure S142-continue

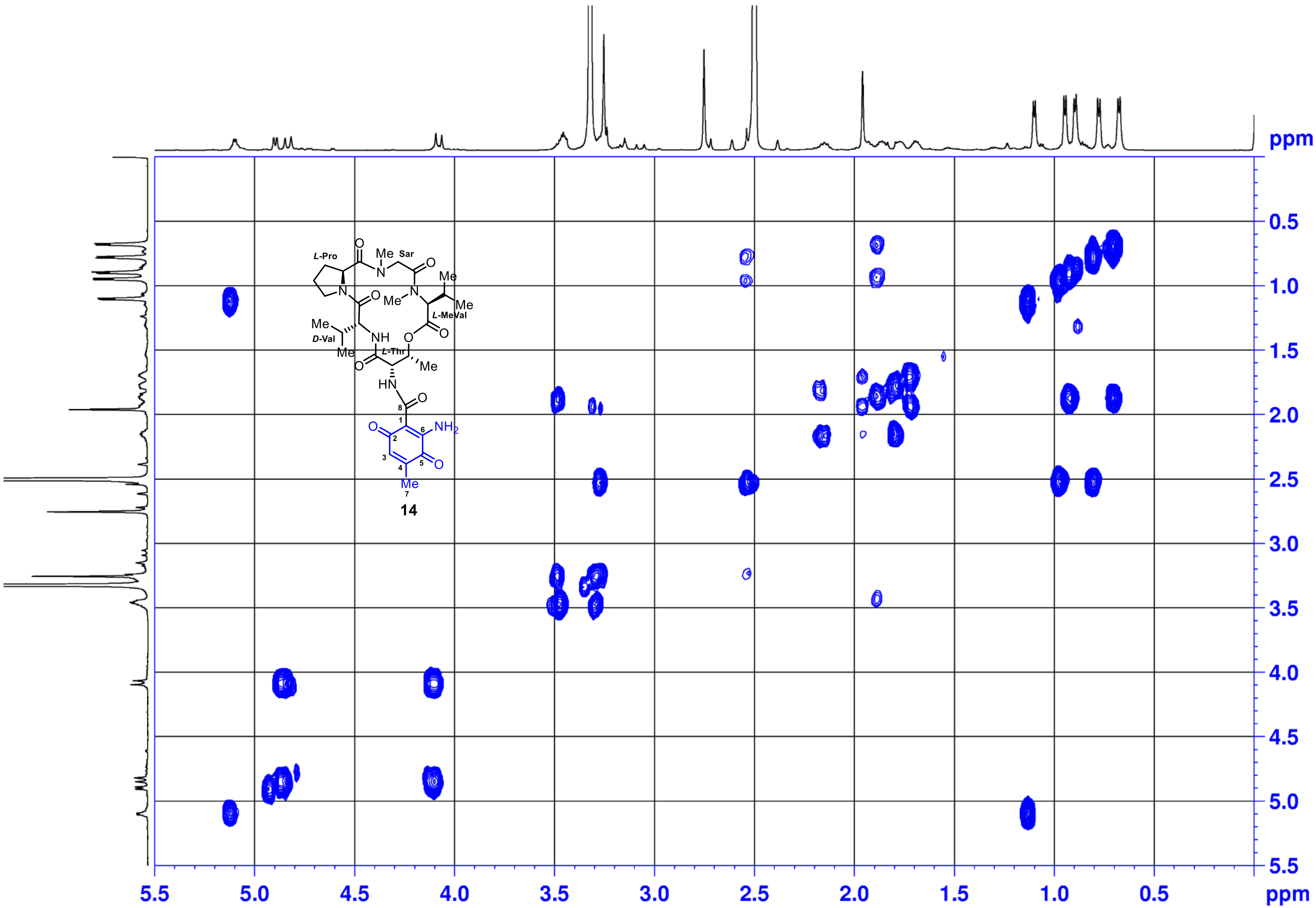

Figure S142-continue

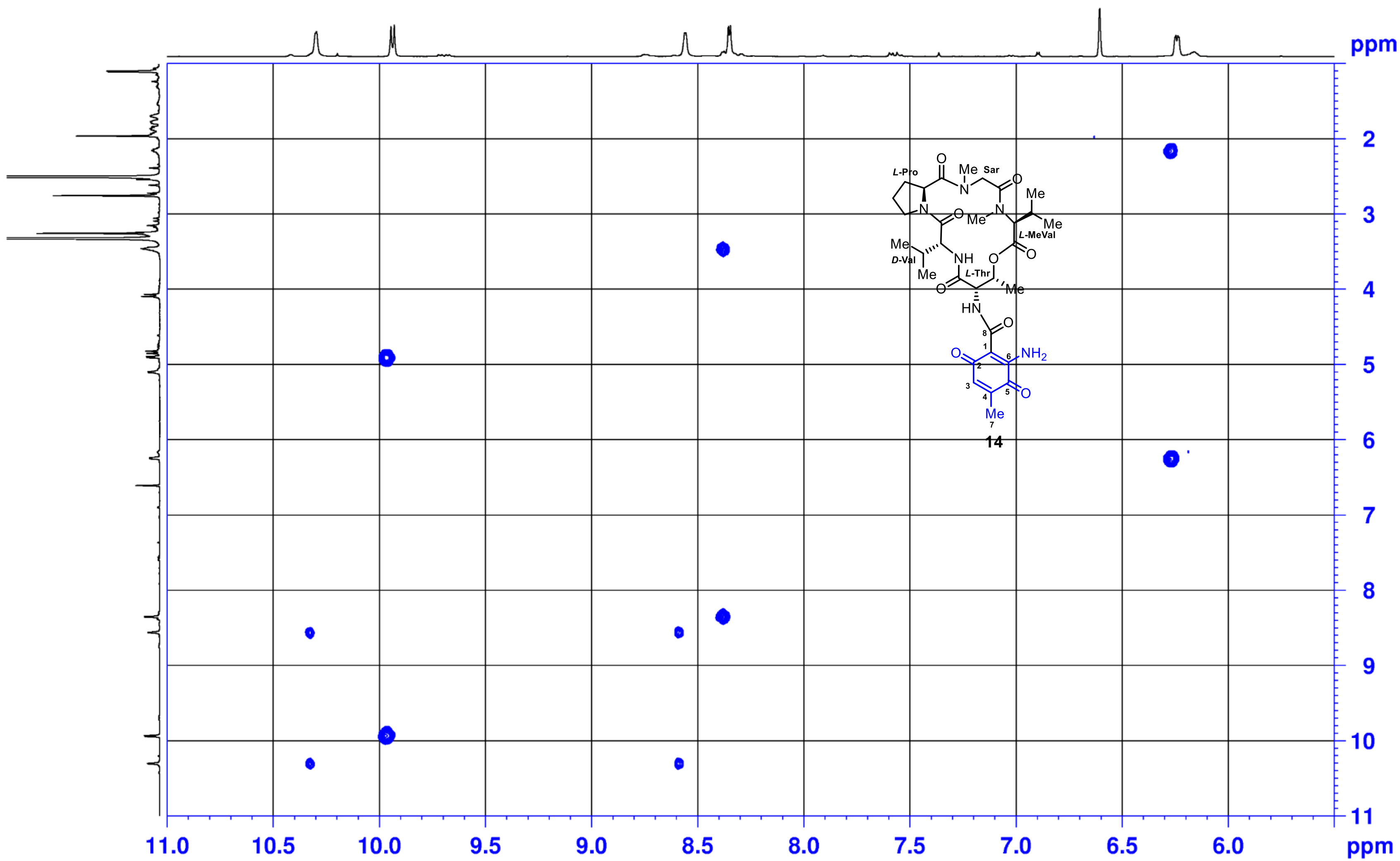

Figure S143. HSQC (600 MHz) spectrum of compound **14** in DMSO-d_6

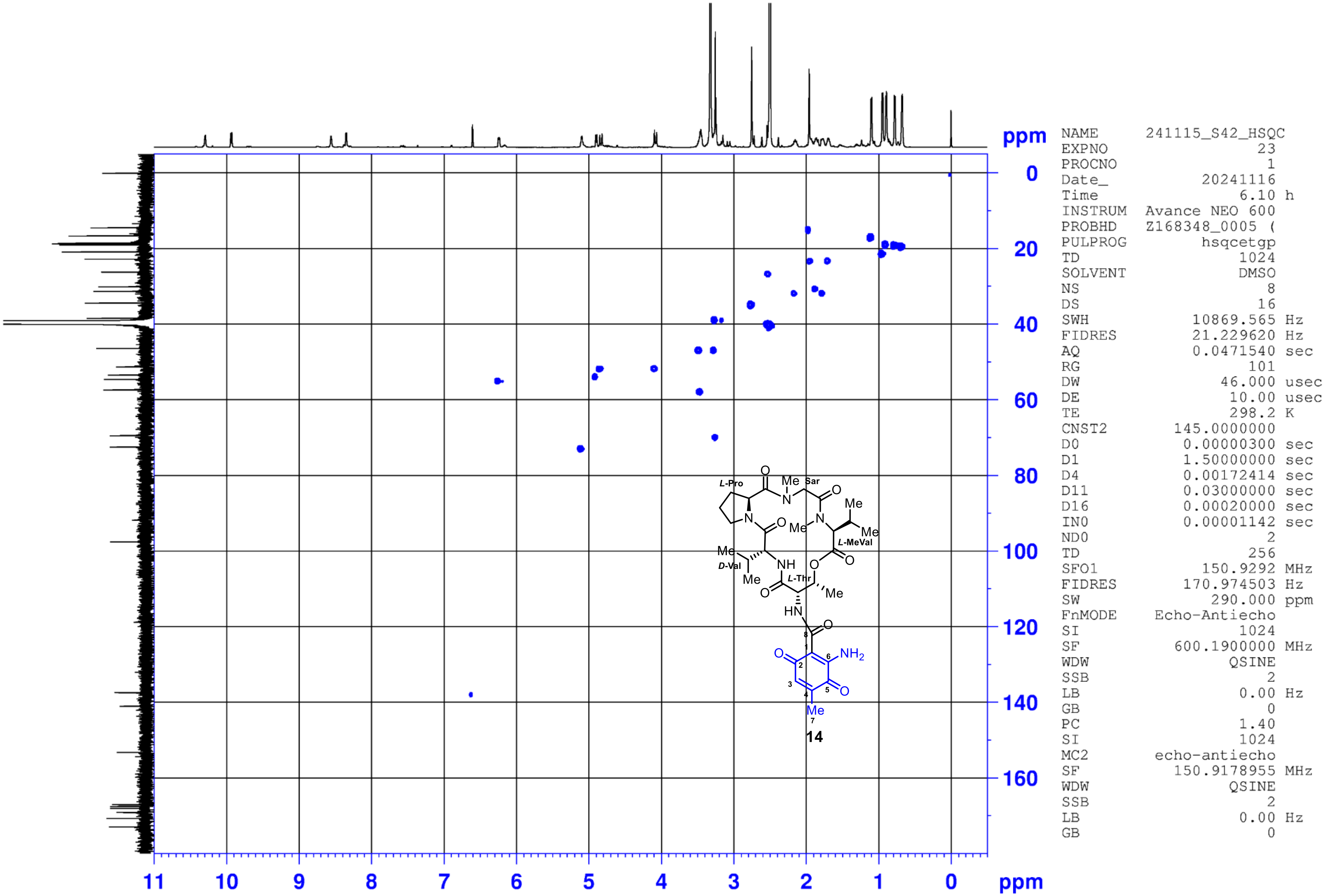

Figure S143-continue

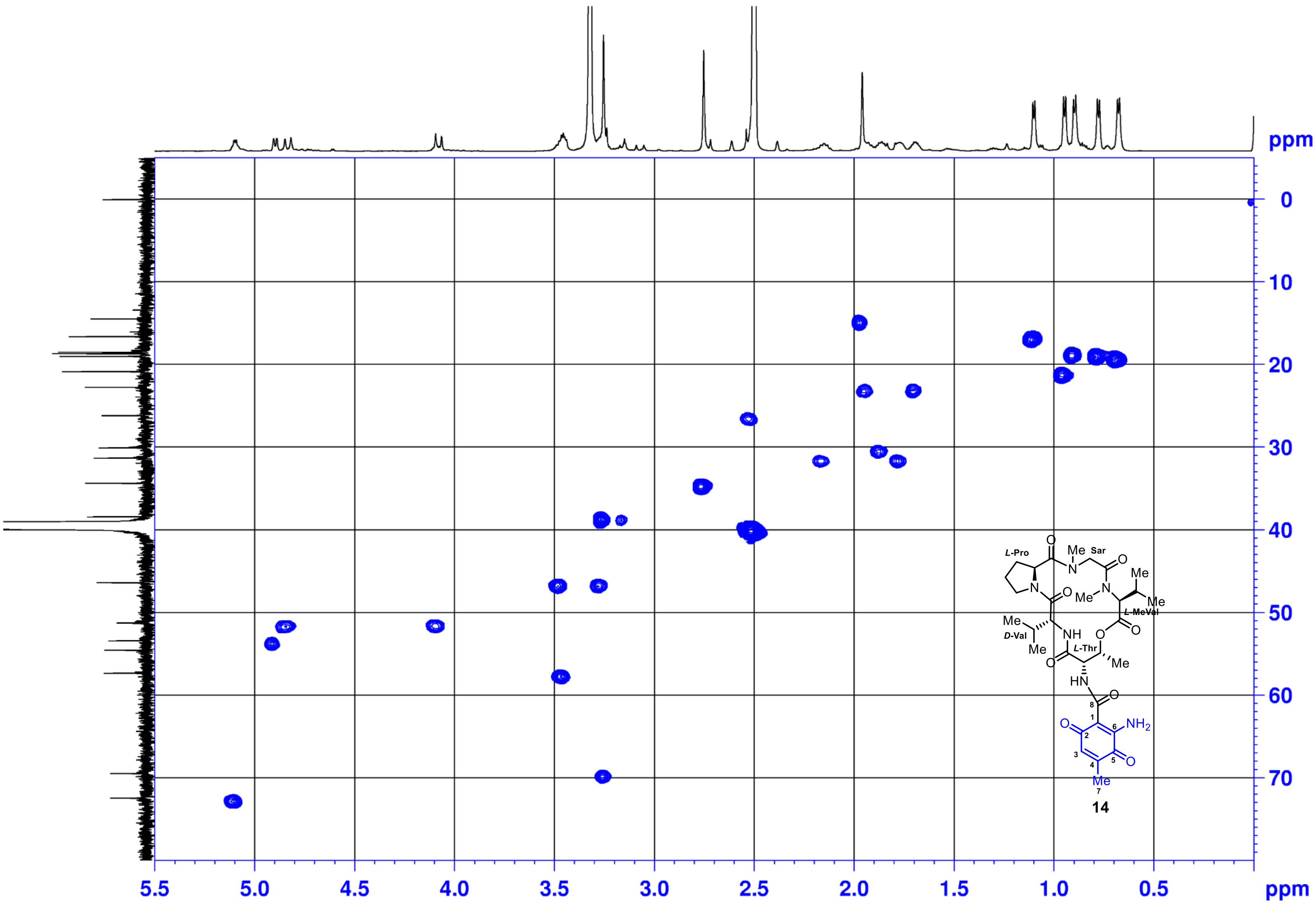

Figure S143-continue

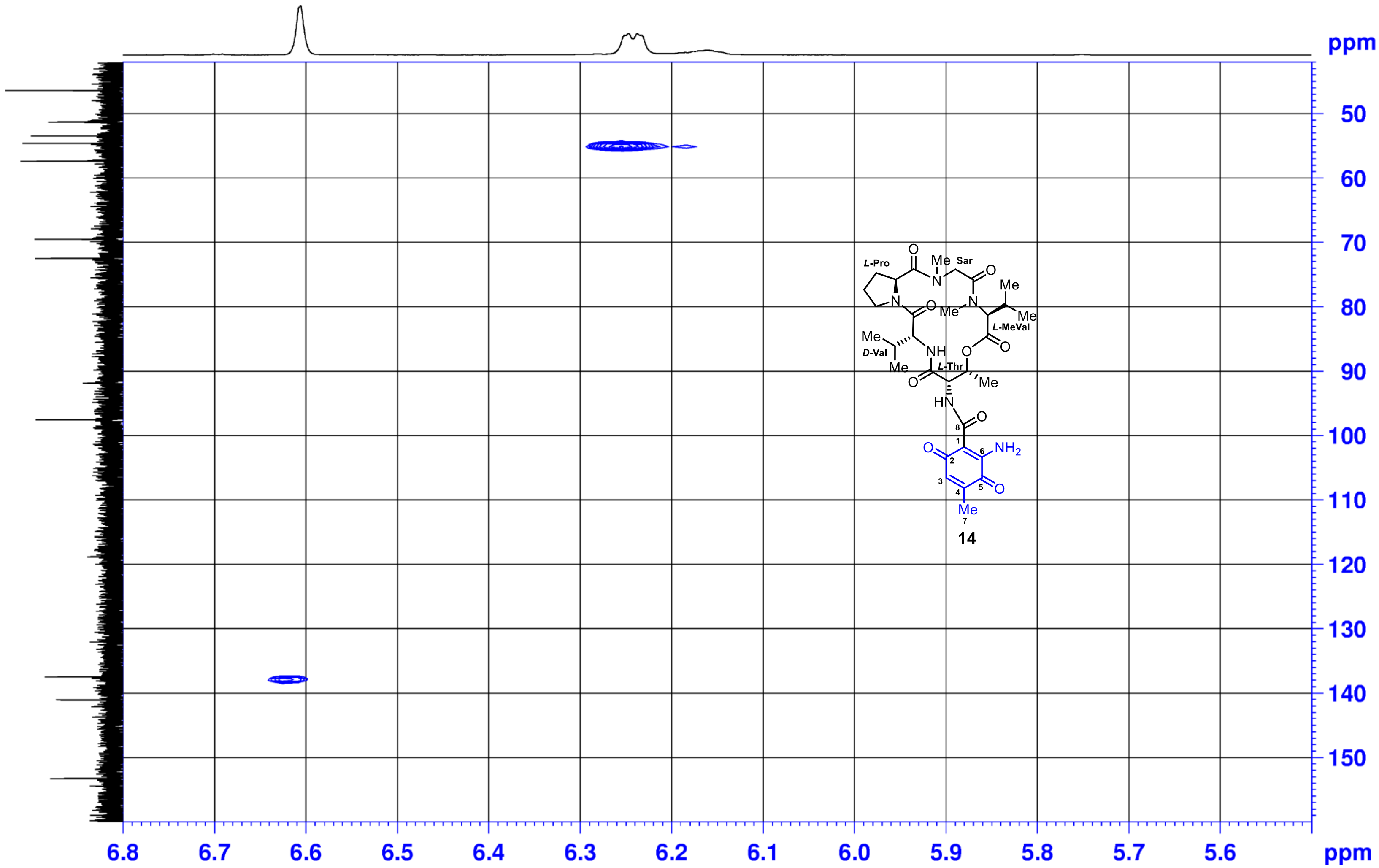

Figure S144. HMBC (600 MHz) spectrum of compound **14** in DMSO-d_6

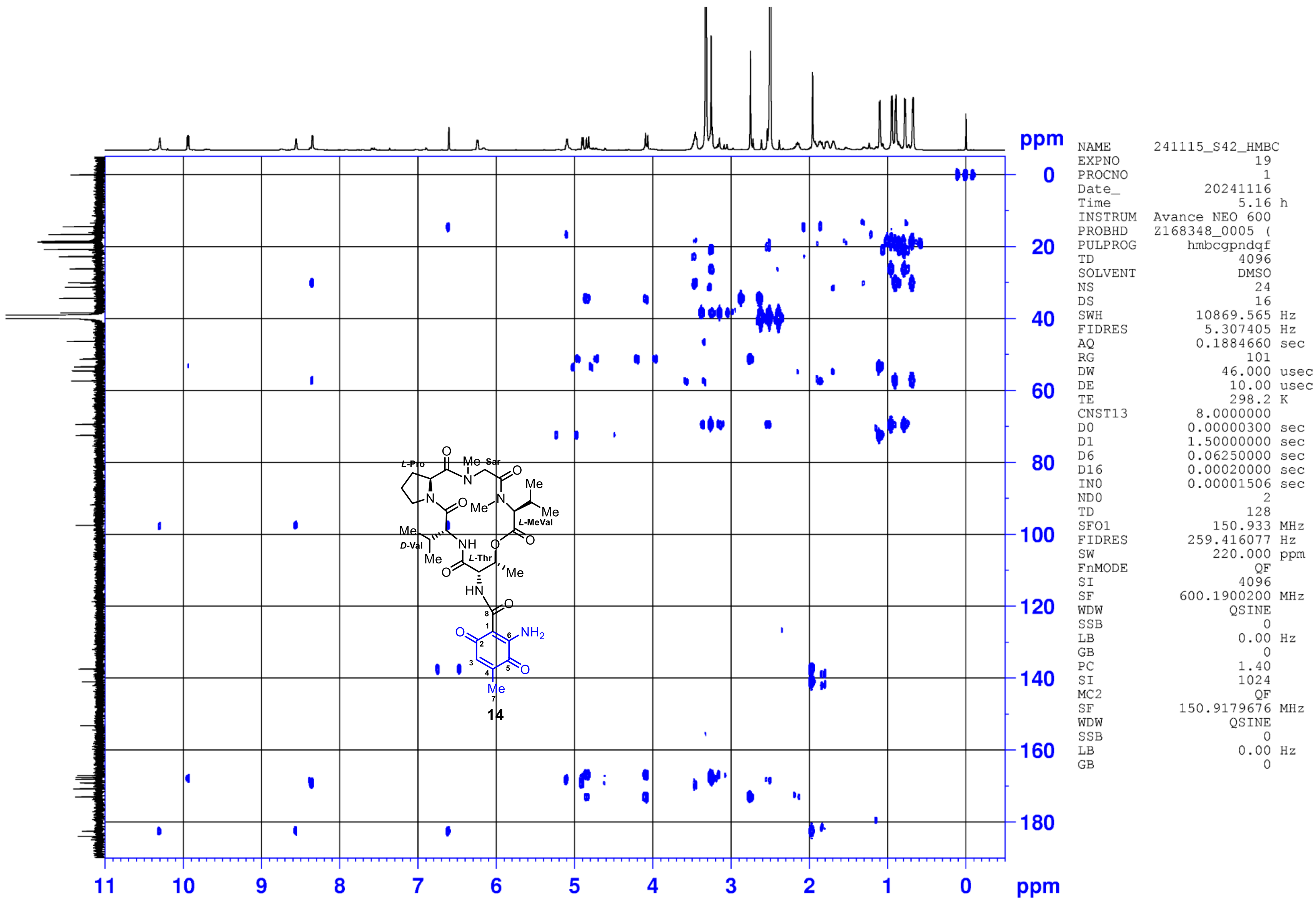

Figure S144-continue

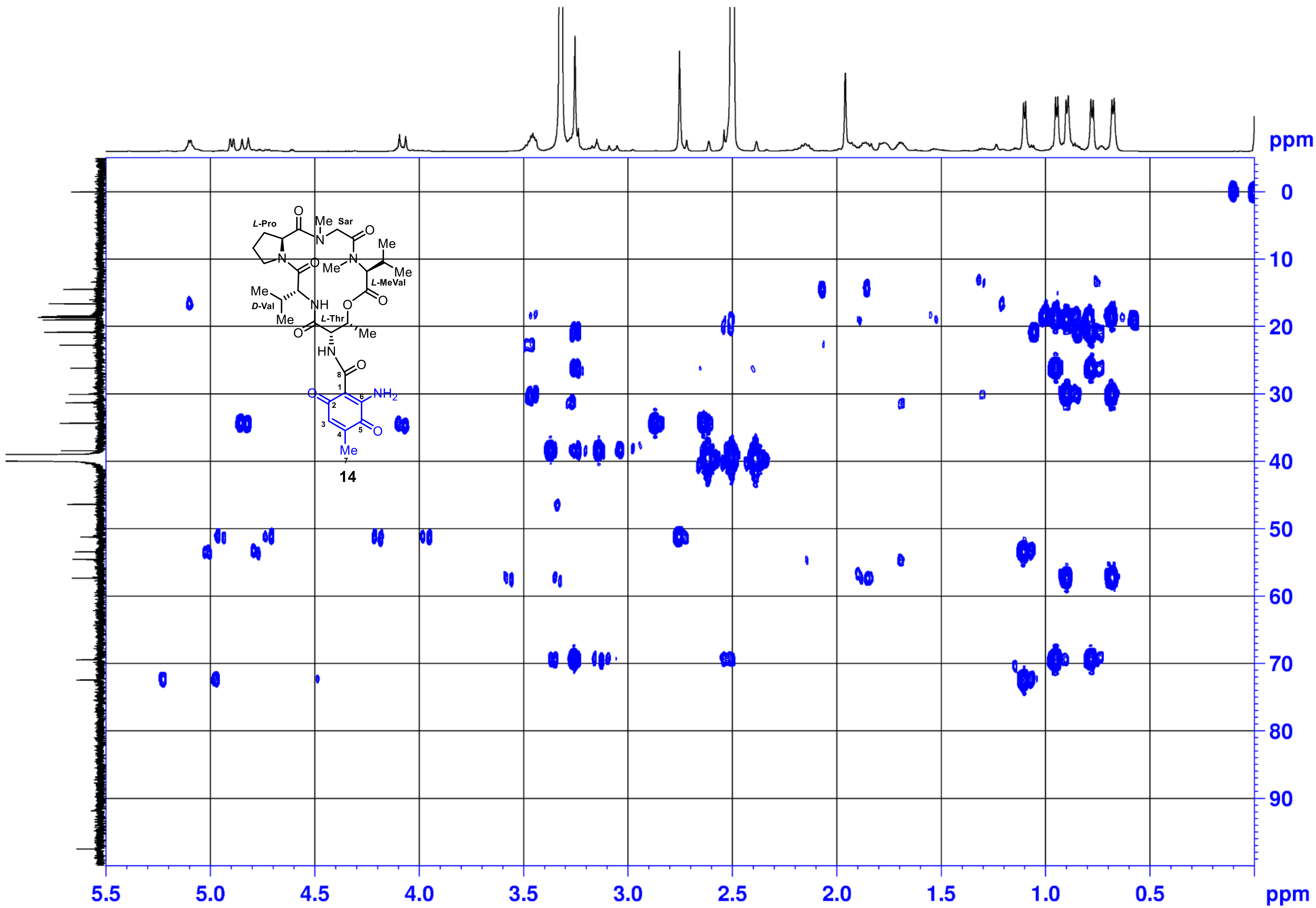

Figure S144-continue

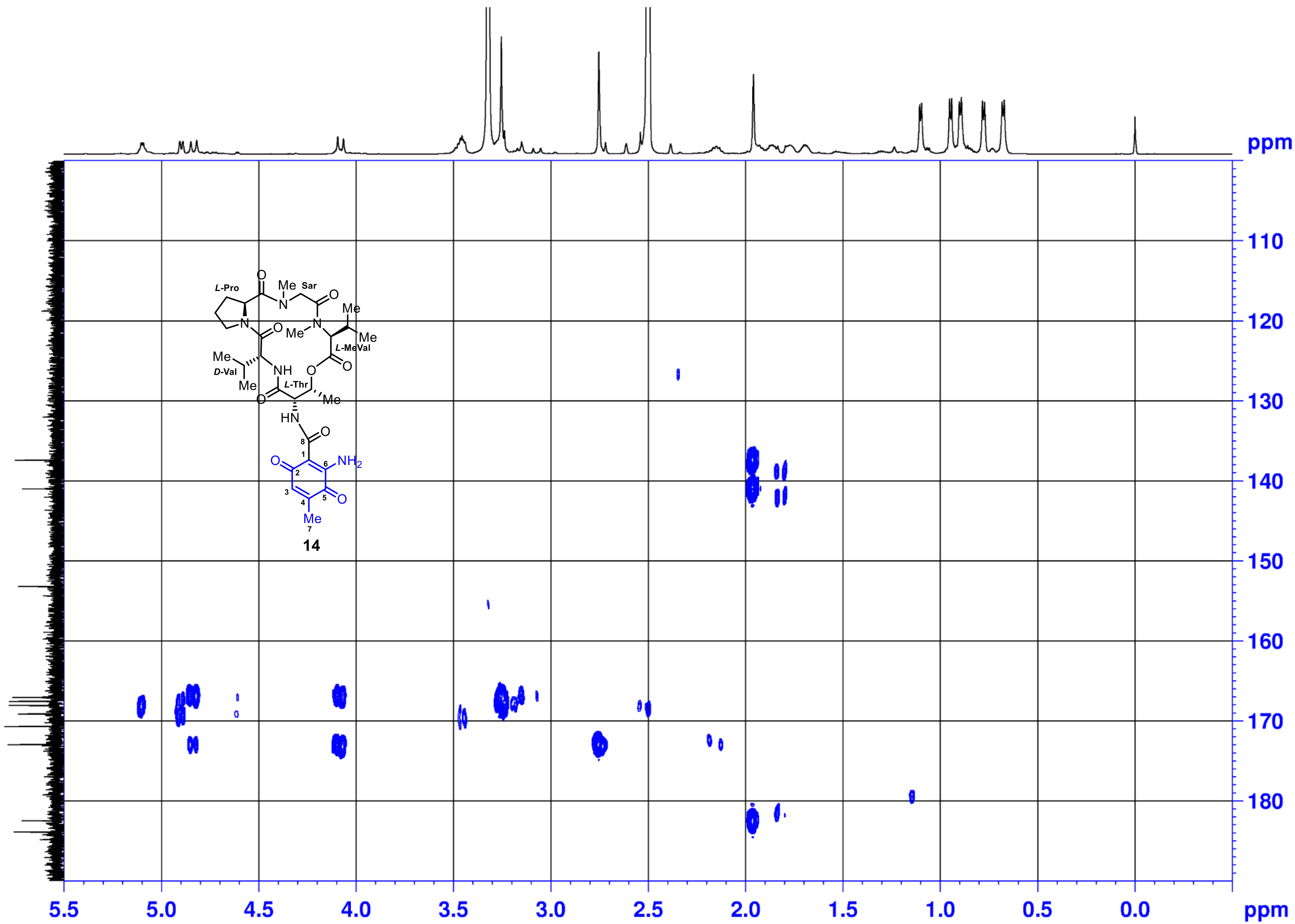

Figure S144-continue

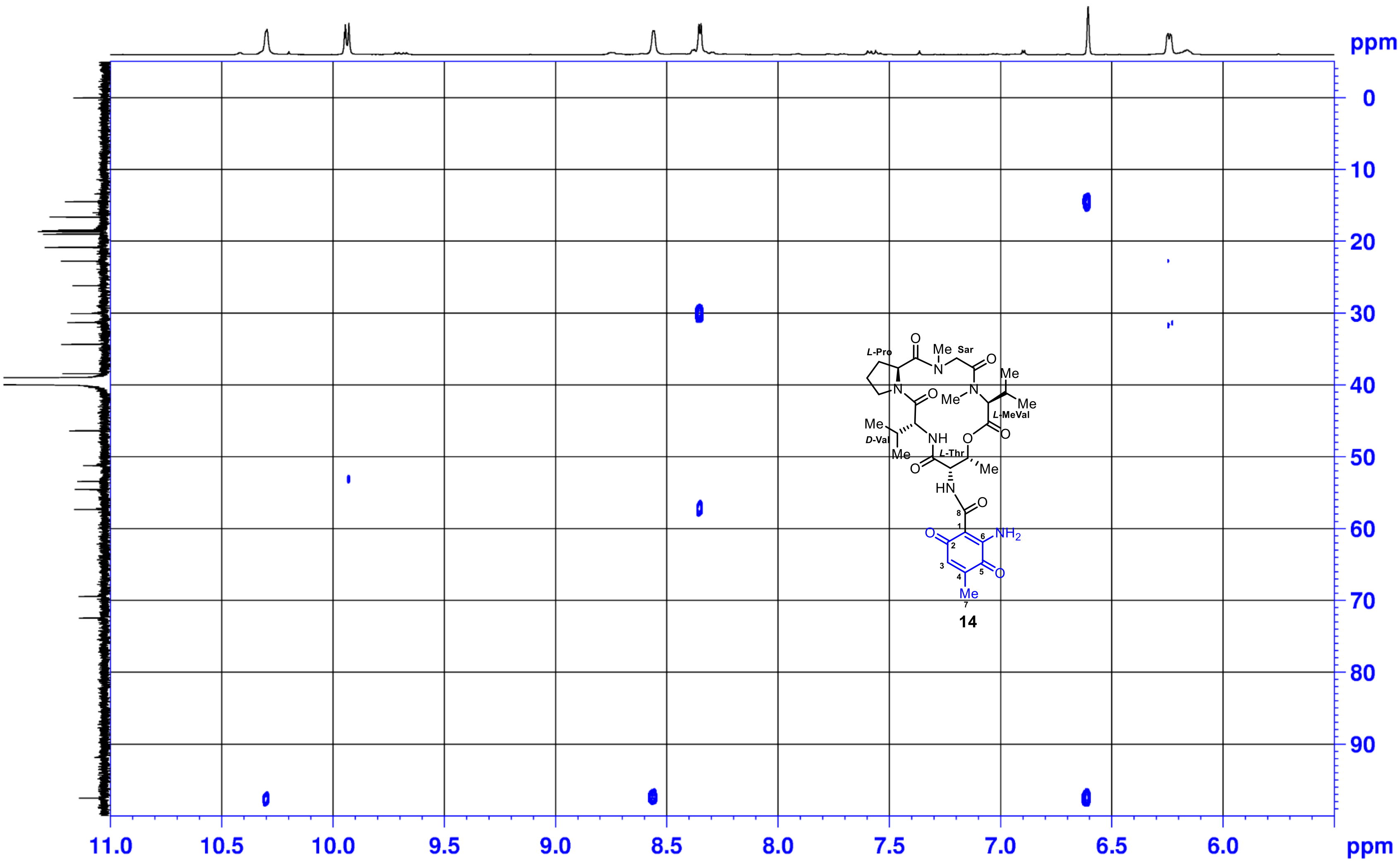

Figure S144-continue

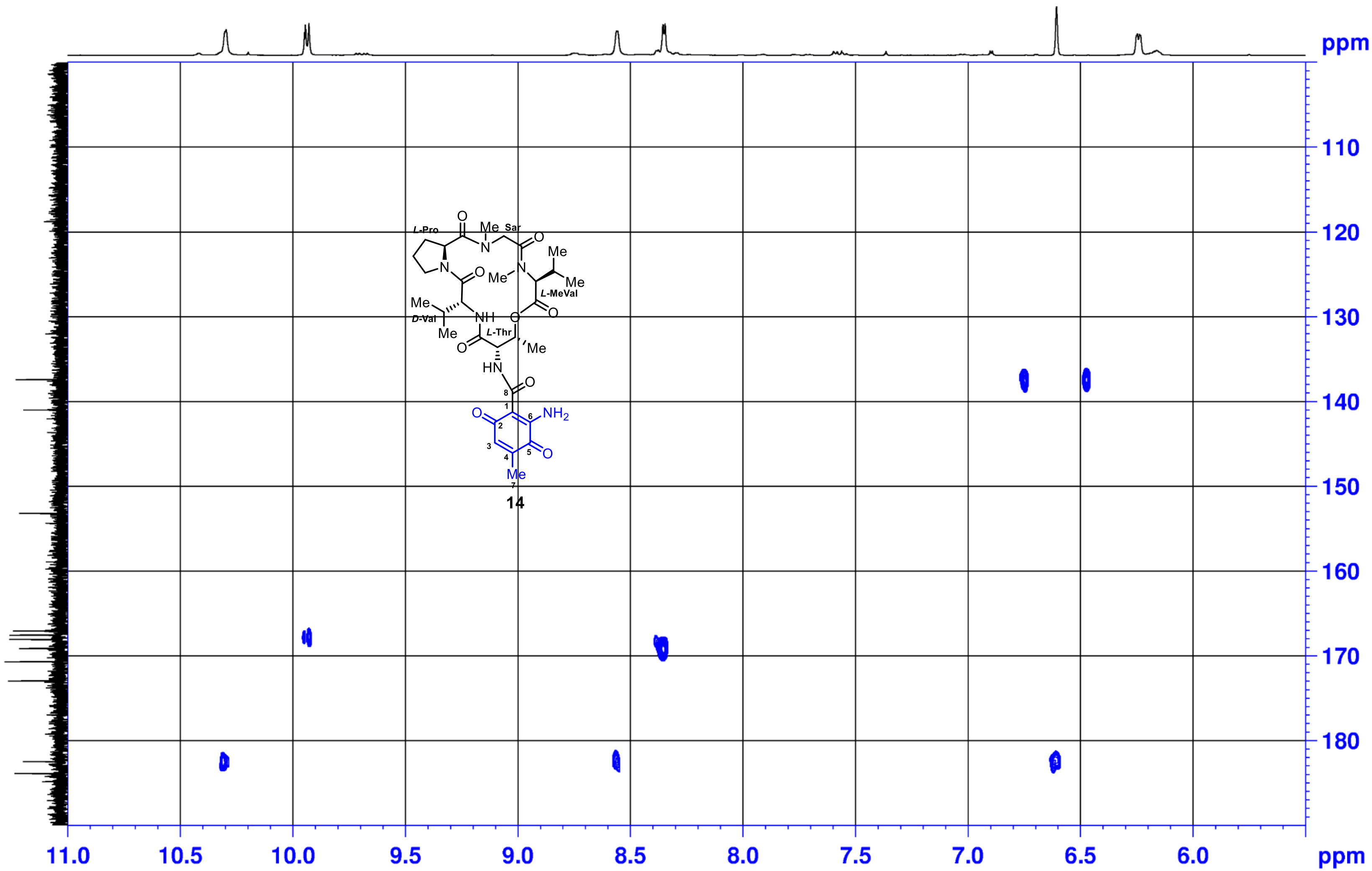

Figure S145. Marfey's analysis of compound **14** and standard amino acids

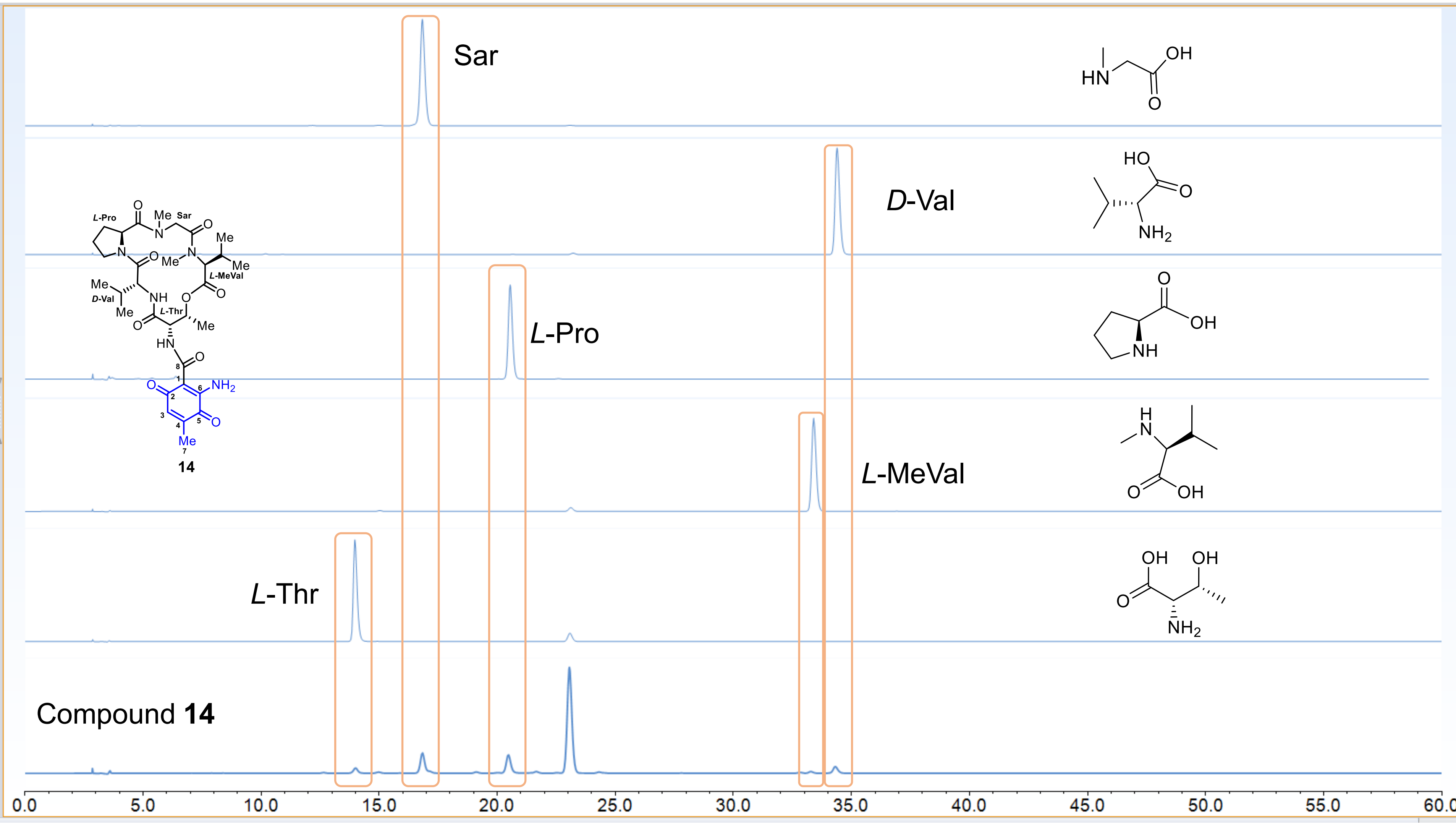

Figure S146. UV spectrum of compound **15**

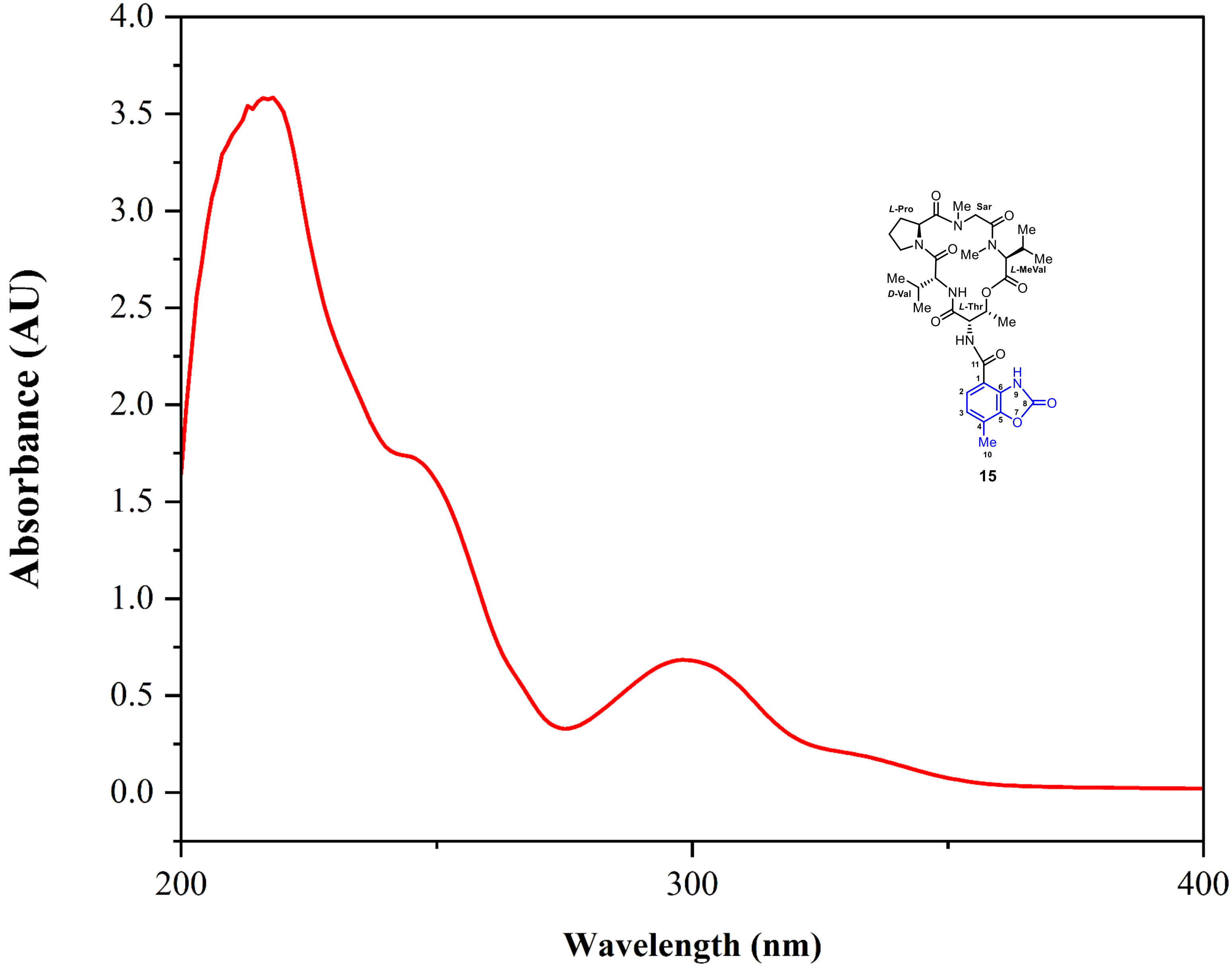

Figure S147. HR-ESIMS for compound **15**

Mass Spectrum SmartFormula Report

Analysis Info

Analysis Name	D:\Data\MS\data\202505\panghe_S-9-1_pos_47_01_19986.d	Acquisition Date	5/29/2025 6:09:27 PM
Method	LC_Direct Infusion_pos_100-1000mz.m	Operator	SCSIO
Sample Name	panghe_S-9-1_pos	Instrument	maXis 255552.00029
Comment			

Acquisition Parameter

Source Type	ESI	Ion Polarity	Positive	Set Nebulizer	0.4 Bar
Focus	Active	Set Capillary	4500 V	Set Dry Heater	180 °C
Scan Begin	100 m/z	Set End Plate Offset	-500 V	Set Dry Gas	4.0 l/min
Scan End	2000 m/z	Set Charging Voltage	0 V	Set Divert Valve	Waste
		Set Corona	0 nA	Set APCI Heater	0 °C

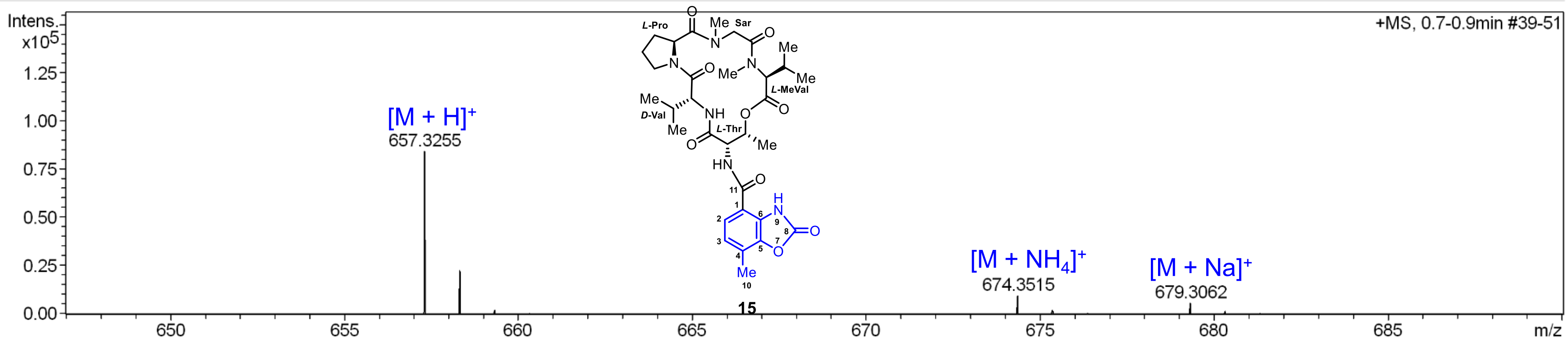

Meas. m/z	#	Ion Formula	Score	m/z	err [ppm]	err [mDa]	mSigma	rdb	e⁻ Conf	N-Rule
657.3255	1	C32H45N6O9	100.00	657.3243	1.9	1.3	62.2	13.5	even	ok
674.3515	1	C32H48N7O9	100.00	674.3508	1.1	0.7	78.7	12.5	even	ok
679.3062	1	C32H44N6NaO9	100.00	679.3062	0.0	0.0	58.5	13.5	even	ok

Figure S148. ^{1}H NMR (600 MHz) spectrum of compound **15** in DMSO-d_6

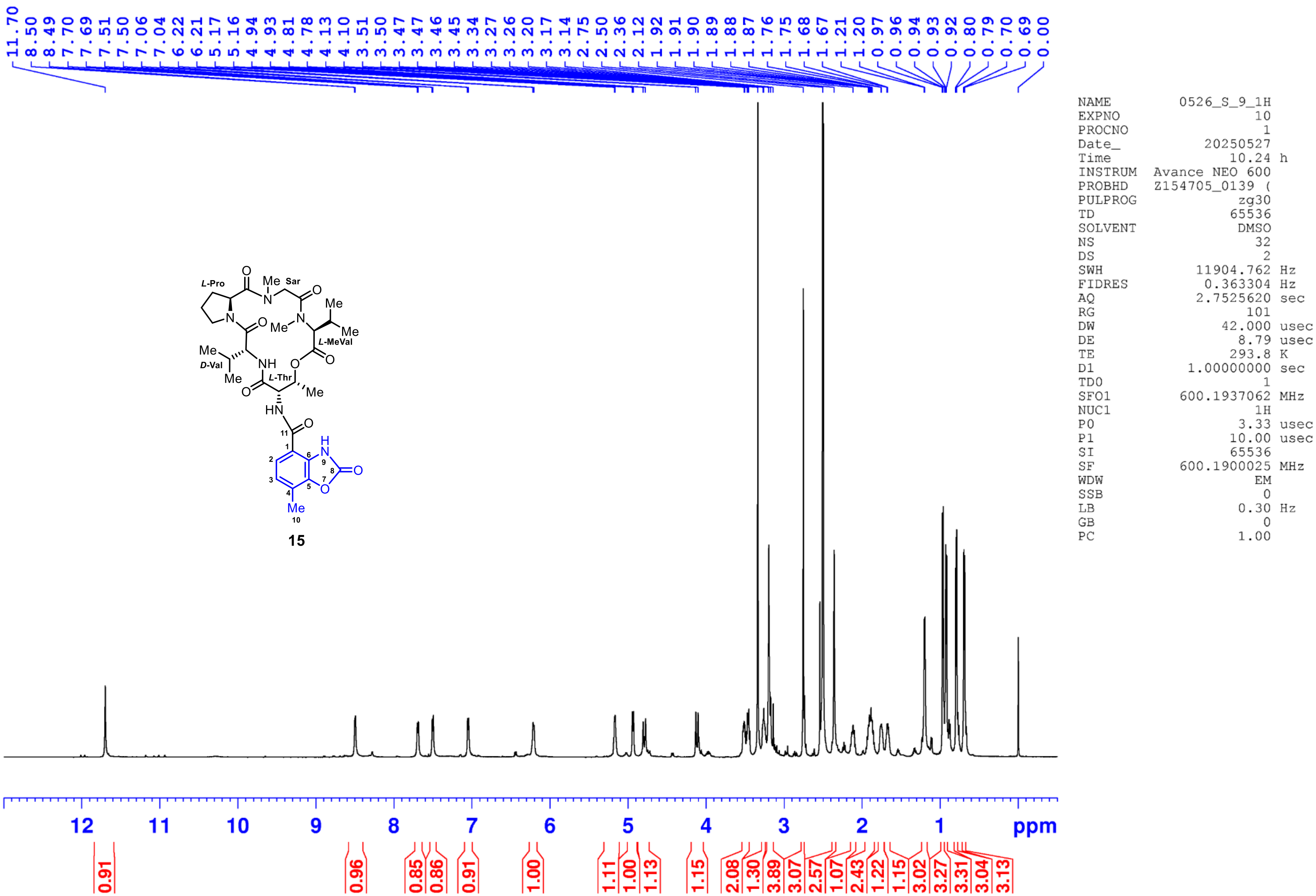

Figure S148-continue

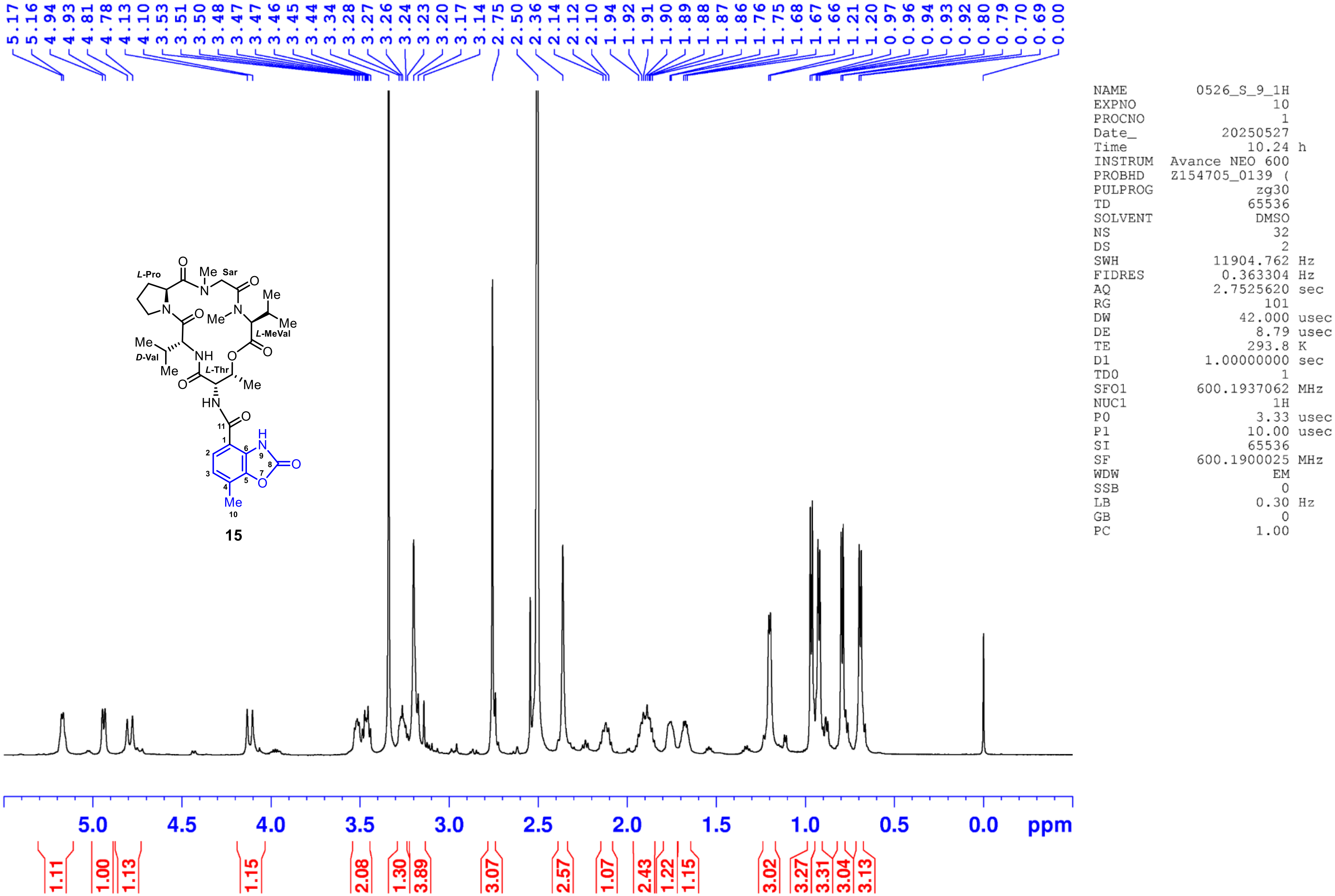

Figure S148-continue

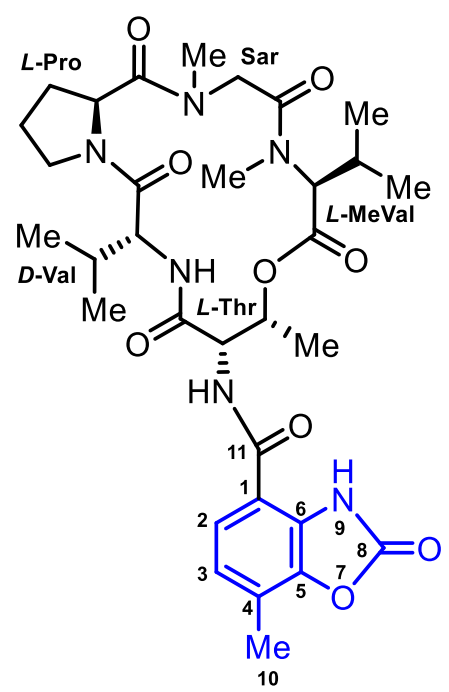

15

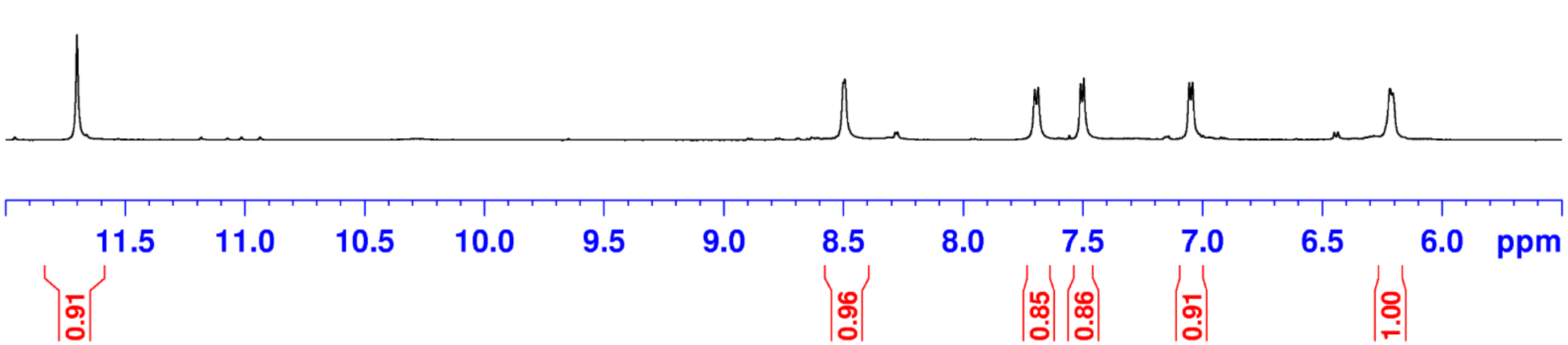

NAME	0526_S_9_1H	
EXPNO	10	
PROCNO	1	
Date_	20250527	
Time	10.24	h
INSTRUM	Avance NEO 600	
PROBHD	Z154705_0139 (	
PULPROG	zg30	
TD	65536	
SOLVENT	DMSO	
NS	32	
DS	2	
SWH	11904.762	Hz
FIDRES	0.363304	Hz
AQ	2.7525620	sec
RG	101	
DW	42.000	usec
DE	8.79	usec
TE	293.8	K
D1	1.00000000	sec
TD0	1	
SFO1	600.1937062	MHz
NUC1	1H	
P0	3.33	usec
P1	10.00	usec
SI	65536	
SF	600.1900025	MHz
WDW	EM	
SSB	0	
LB	0.30	Hz
GB	0	
PC	1.00	

Figure S149. ^{13}C NMR (150 MHz) spectrum of compound **15** in DMSO-d_6

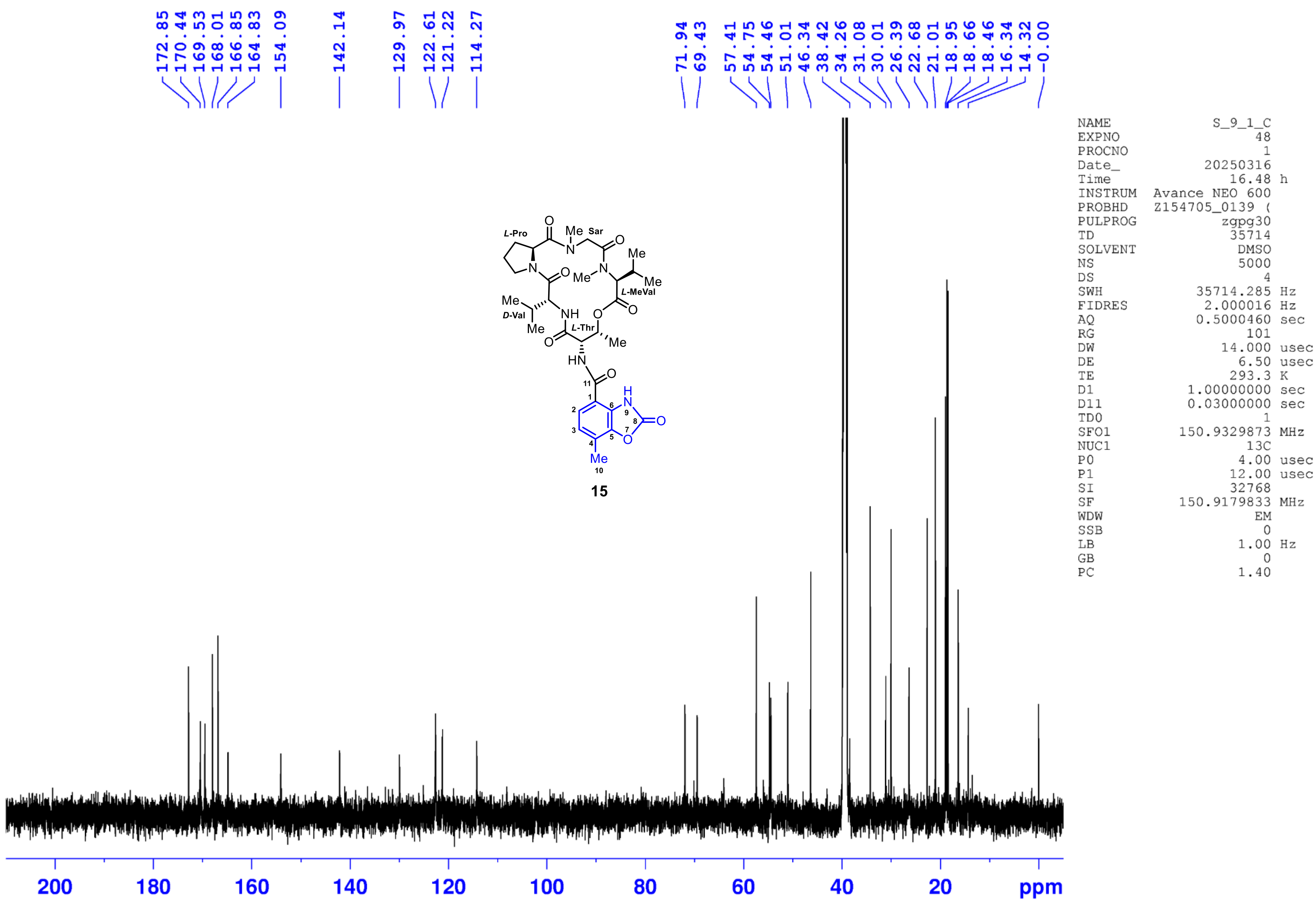

Figure S149-continue

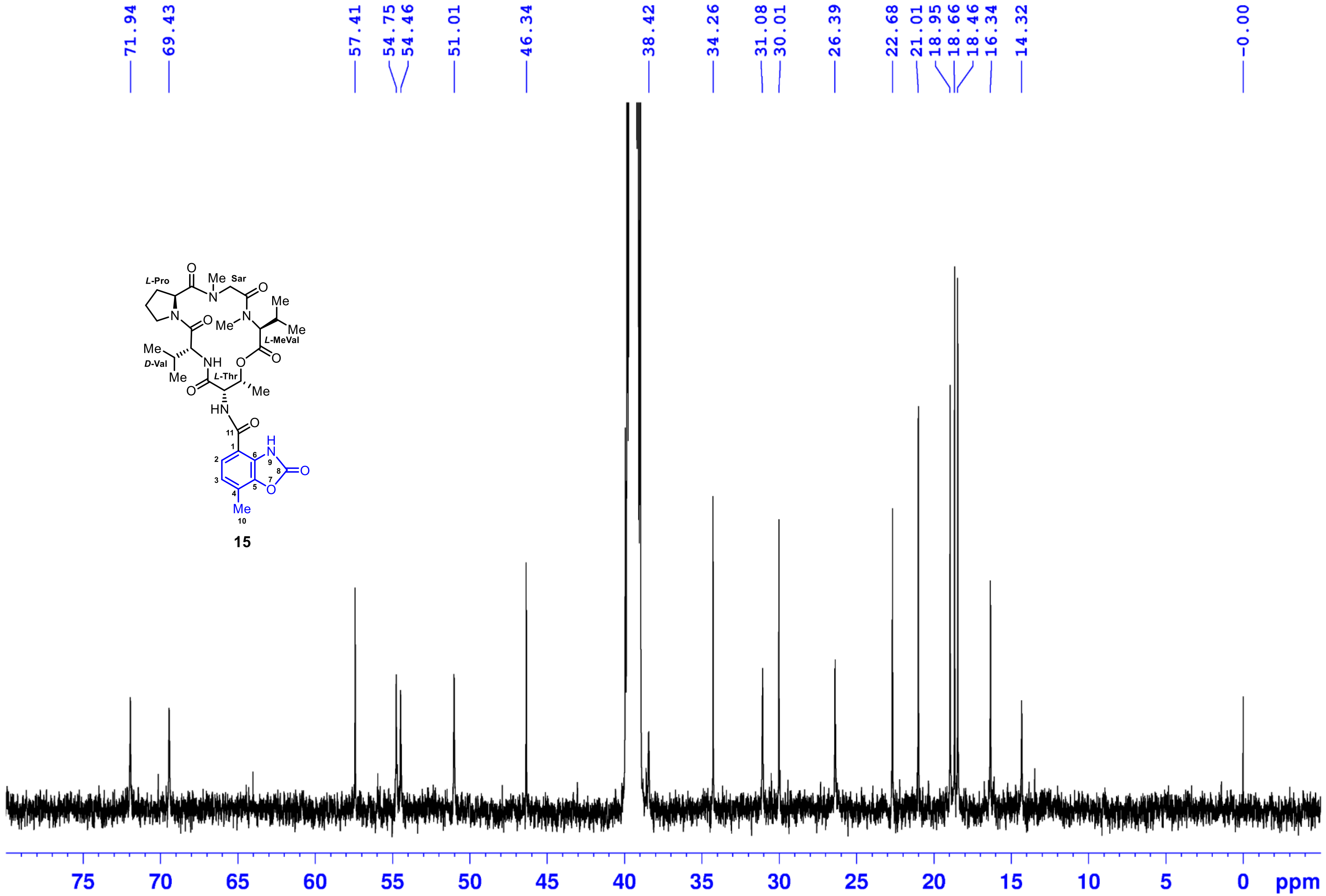

Figure S149-continue

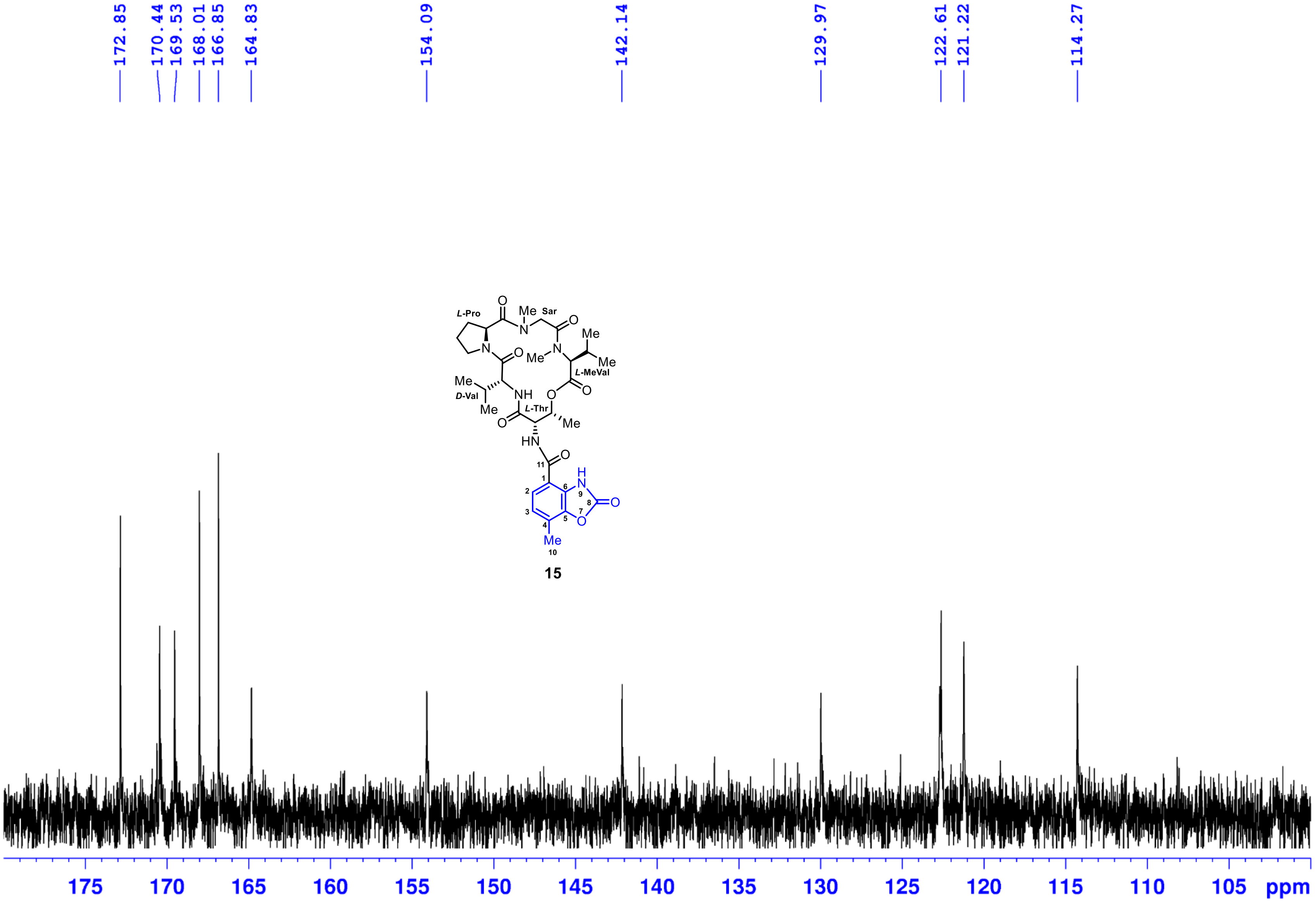

Figure S150. DEPT135 (150 MHz) spectrum of compound **15** in DMSO-d_6

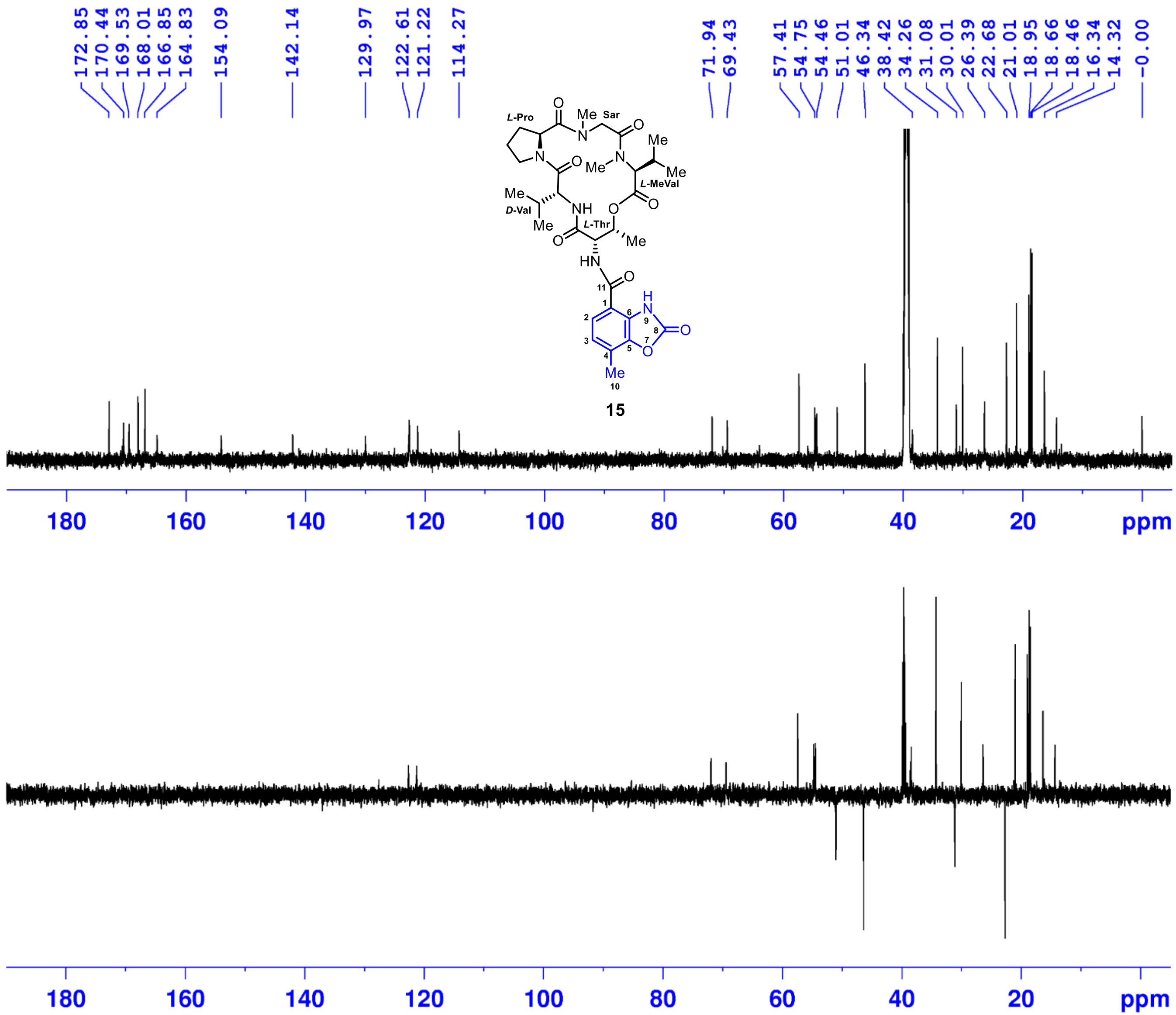

NAME	S_9_1_D135
EXPNO	49
PROCNO	1
Date_	20250316
Time	17.13 h
INSTRUM	Avance NEO 600
PROBHD	Z154705_0139 (
PULPROG	deptsp135
TD	65536
SOLVENT	DMSO
NS	512
DS	8
SWH	41666.668 Hz
FIDRES	1.271566 Hz
AQ	0.7864820 sec
RG	101
DW	12.000 usec
DE	6.50 usec
TE	292.7 K
CNST2	145.0000000
D1	2.00000000 sec
D2	0.00344828 sec
D12	0.00002000 sec
TD0	1
SFO1	150.9299690 MHz
NUC1	13C
P1	12.00 usec
P13	2000.00 usec
SI	32768
SF	150.9179820 MHz
WDW	EM
SSB	0
LB	1.00 Hz
GB	0
PC	1.40

Figure S150-continue

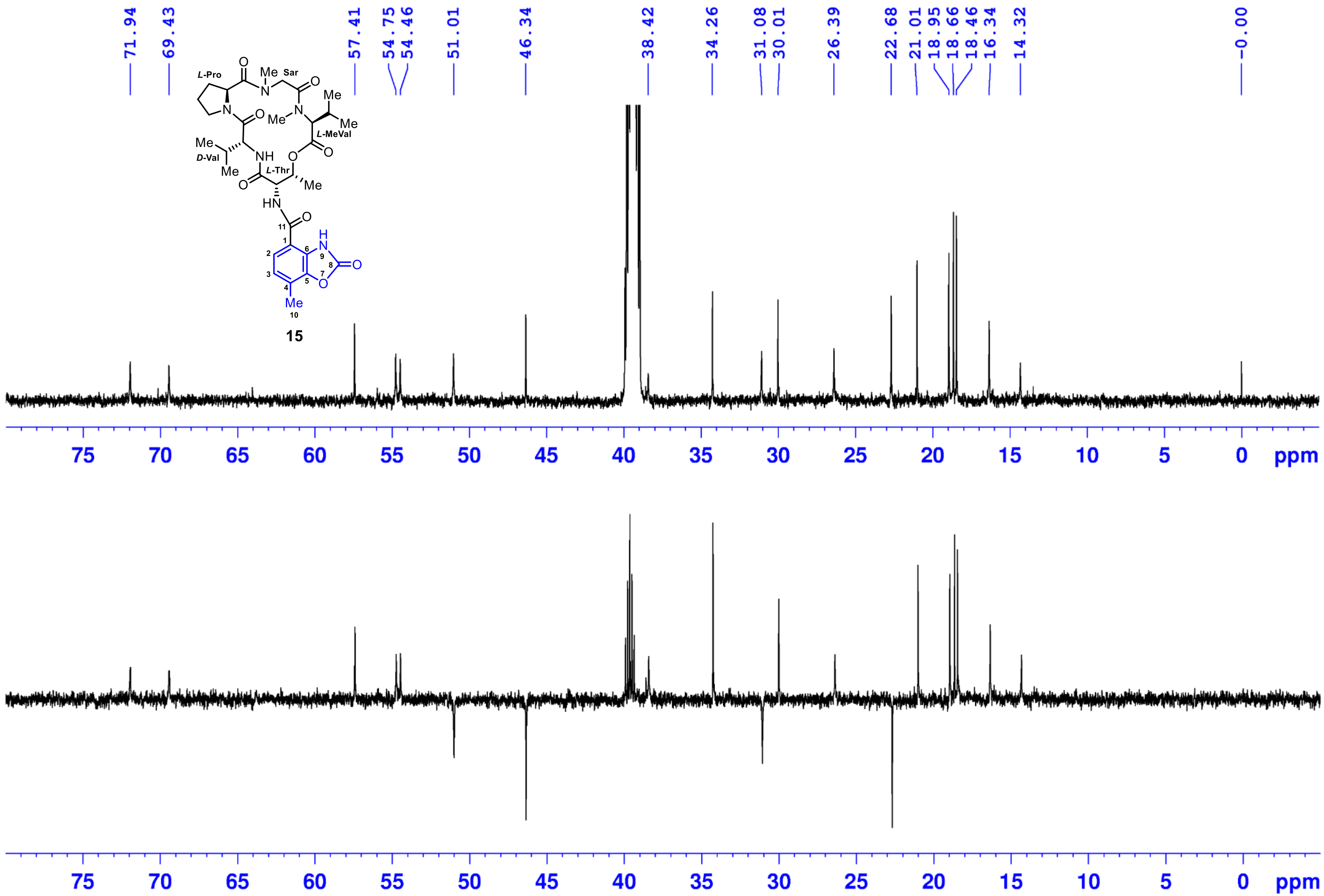

Figure S150-continue

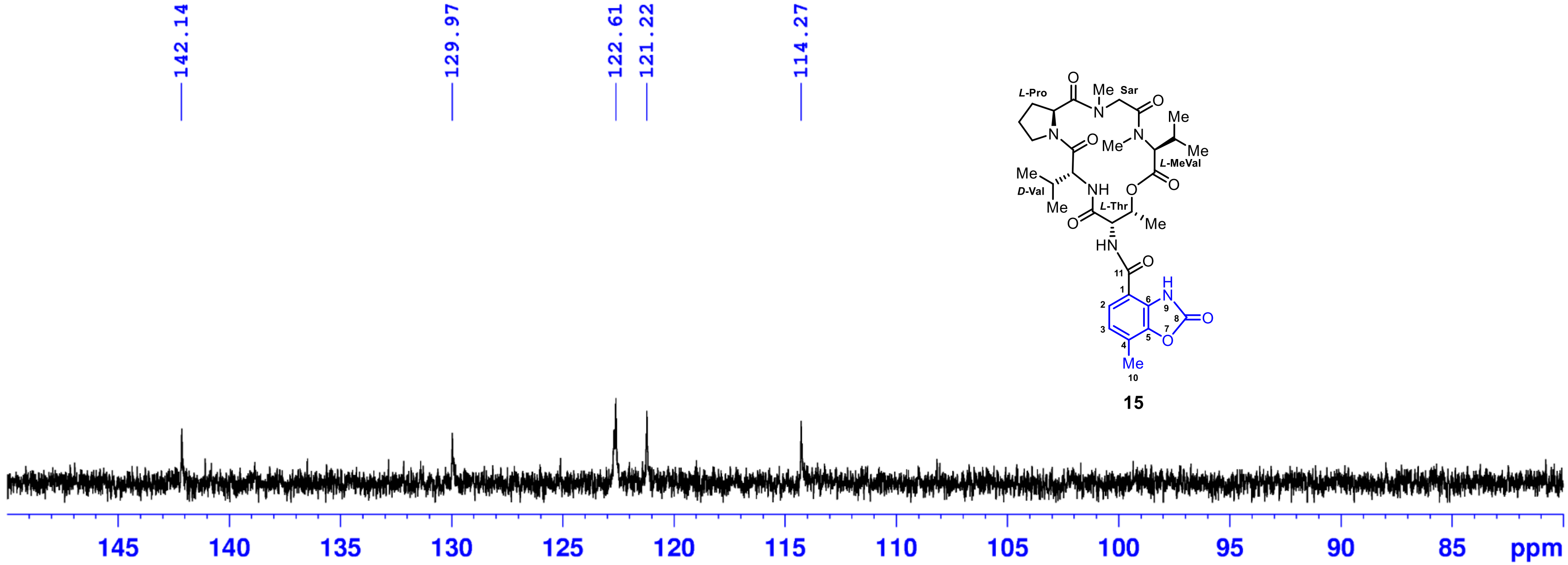

15

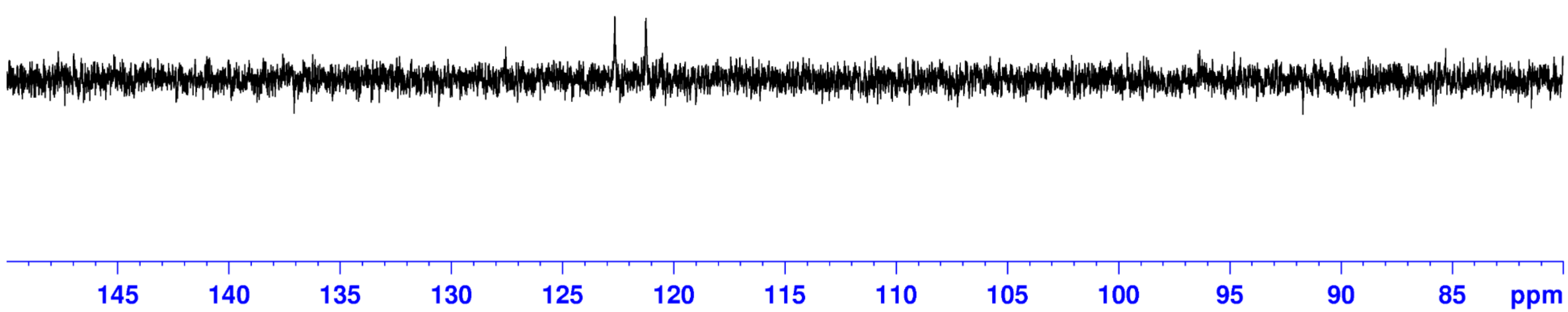

Figure S151. ^{1}H–^{1}H COSY (600 MHz) spectrum of compound **15** in DMSO-d_6

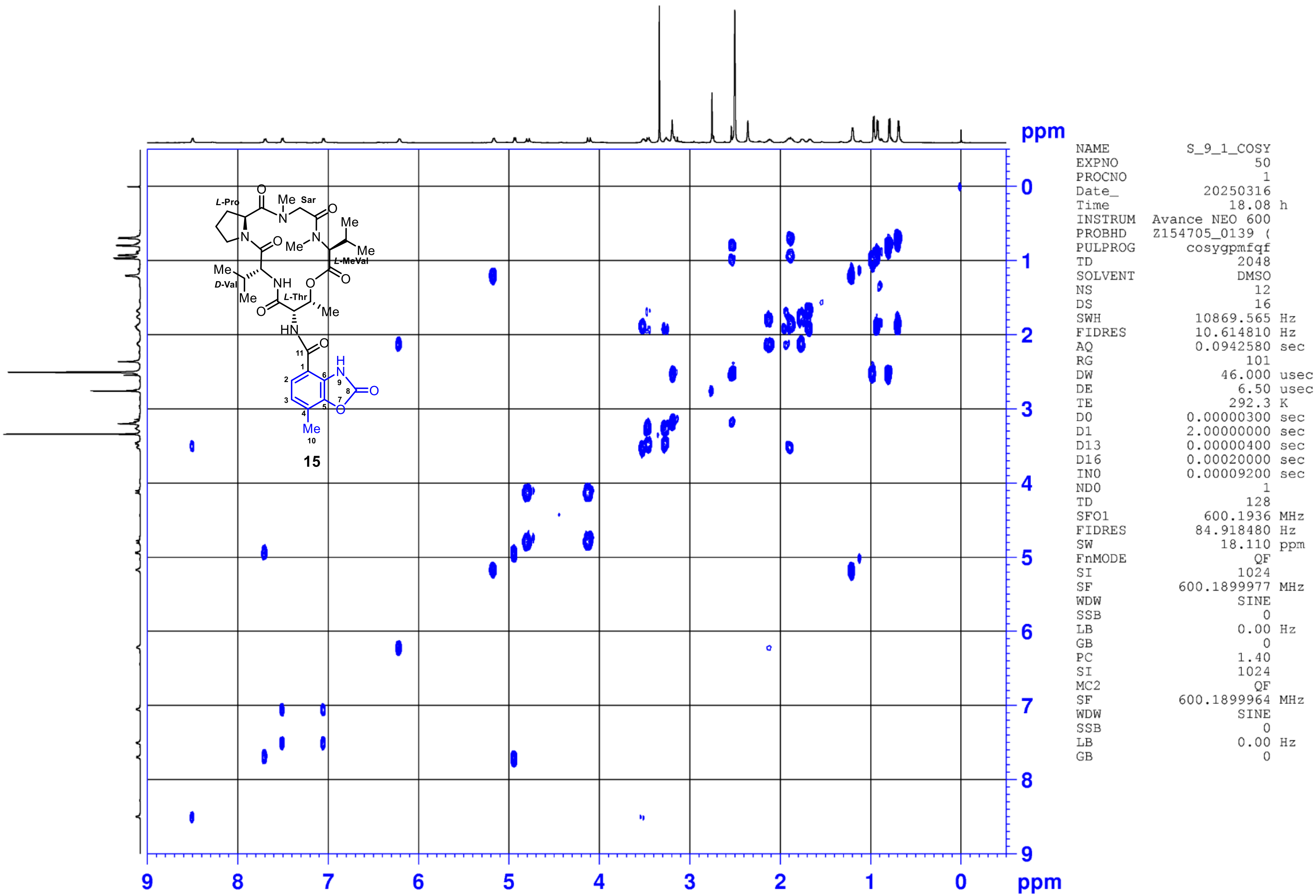

Figure S151-continue

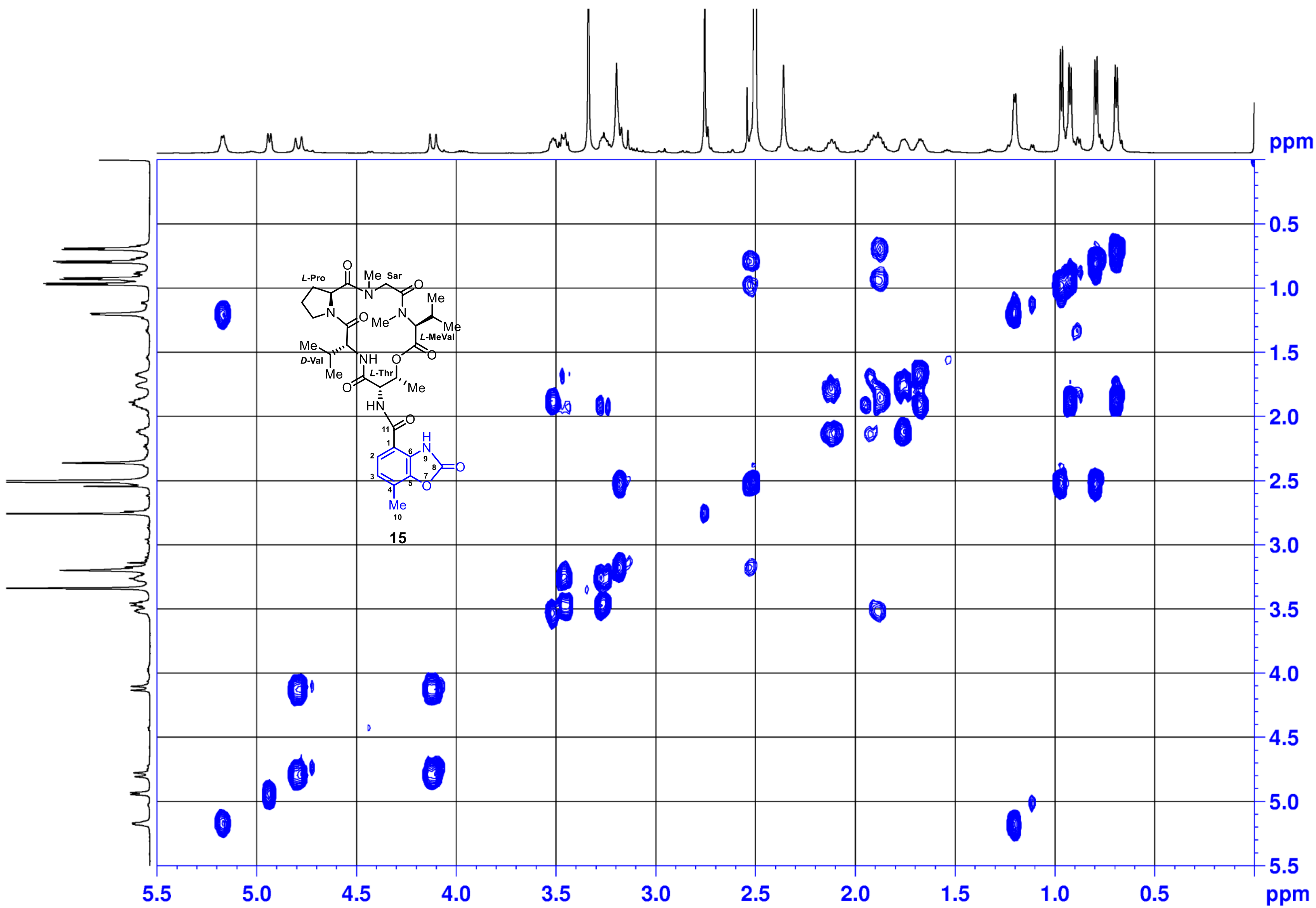

Figure S151-continue

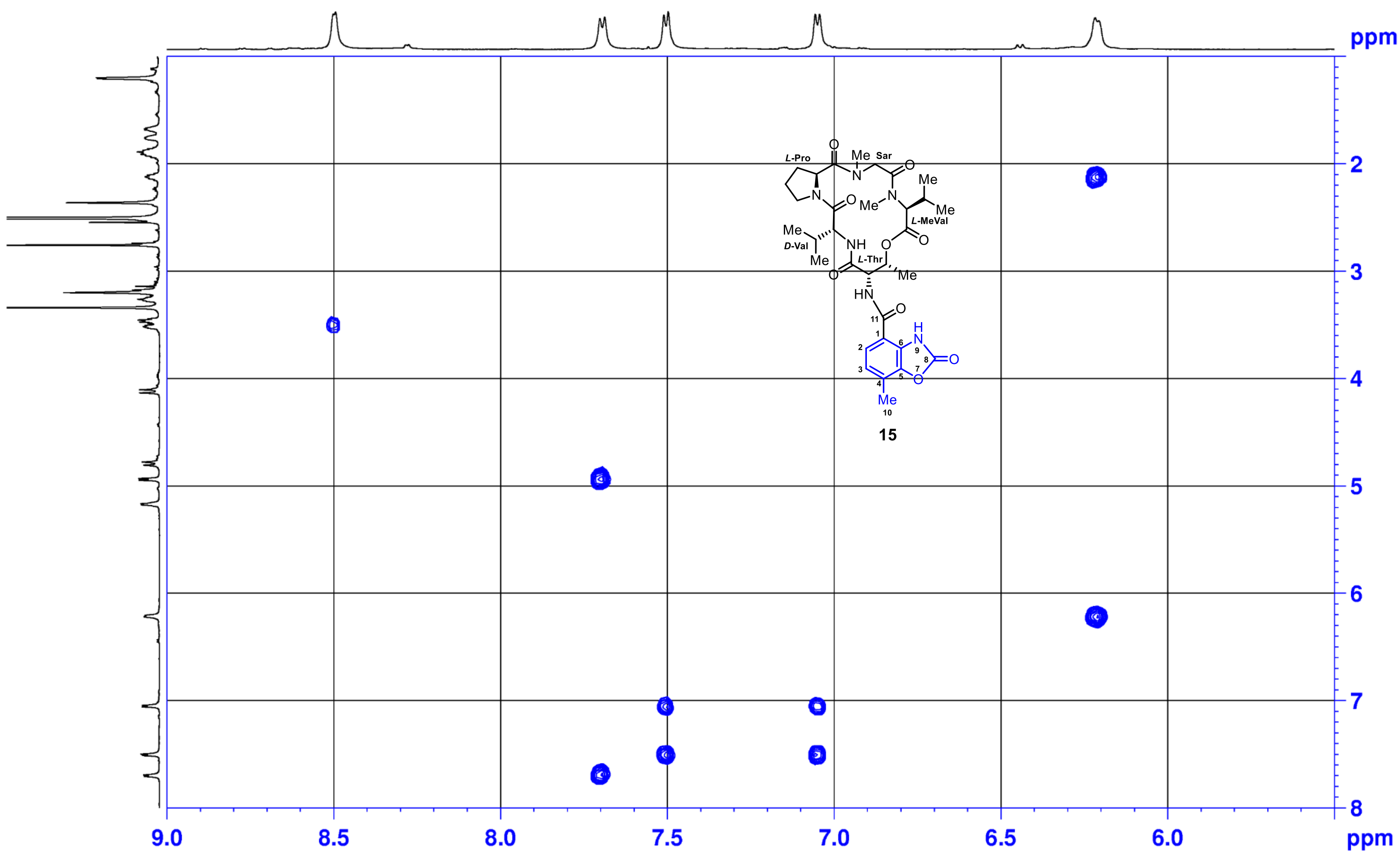

Figure S152. HSQC (600 MHz) spectrum of compound **15** in DMSO-d_6

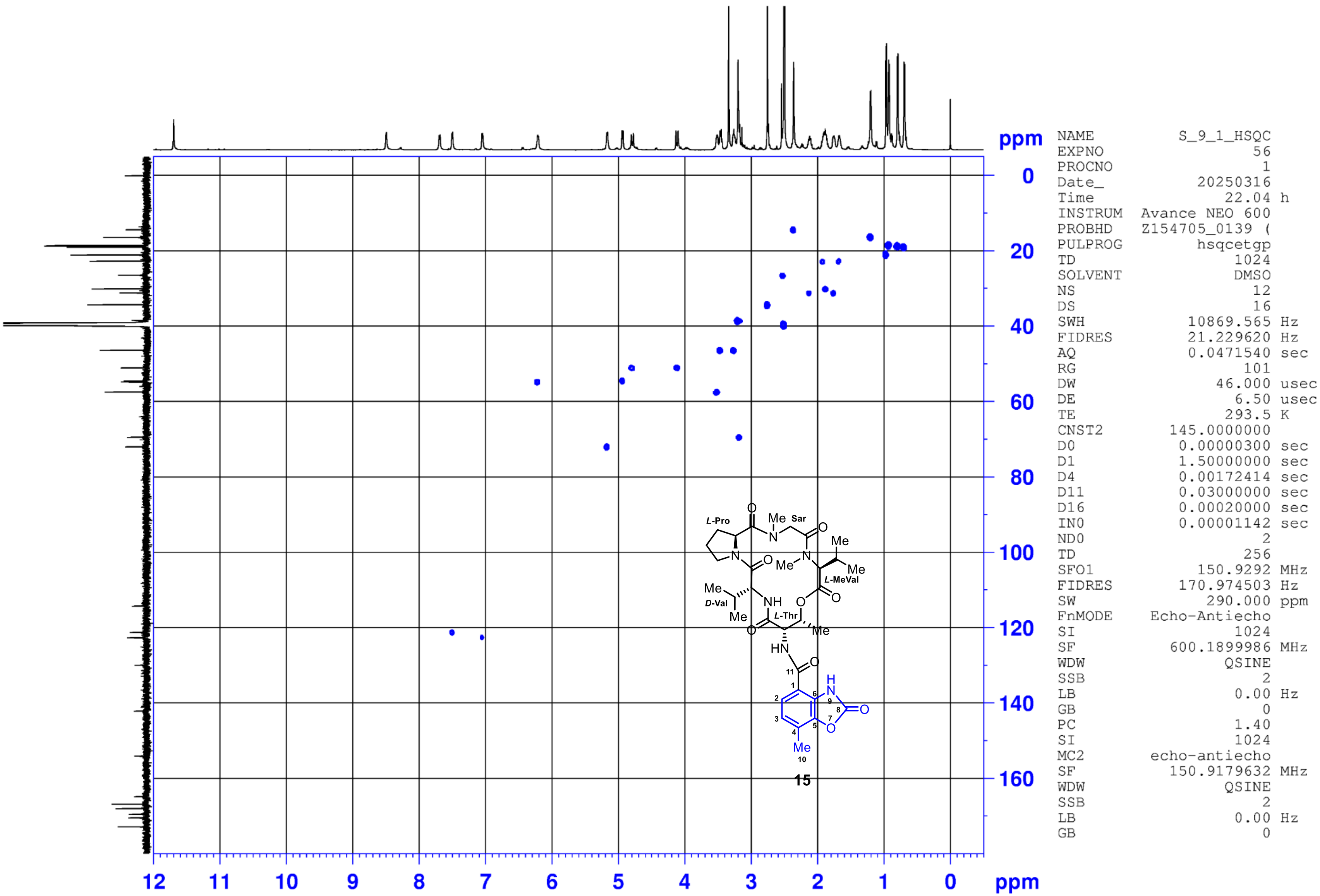

Figure S152-continue

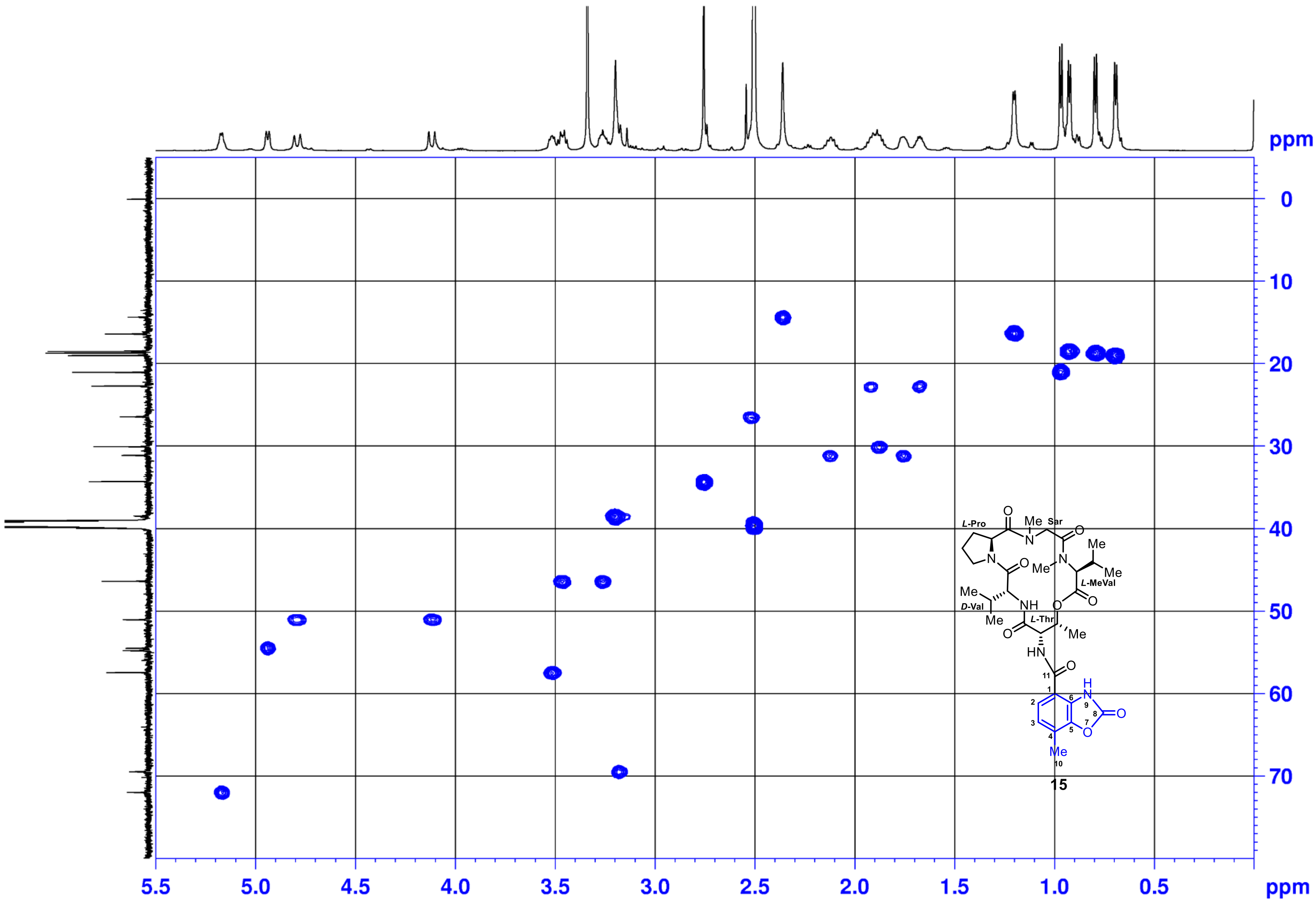

Figure S152-continue

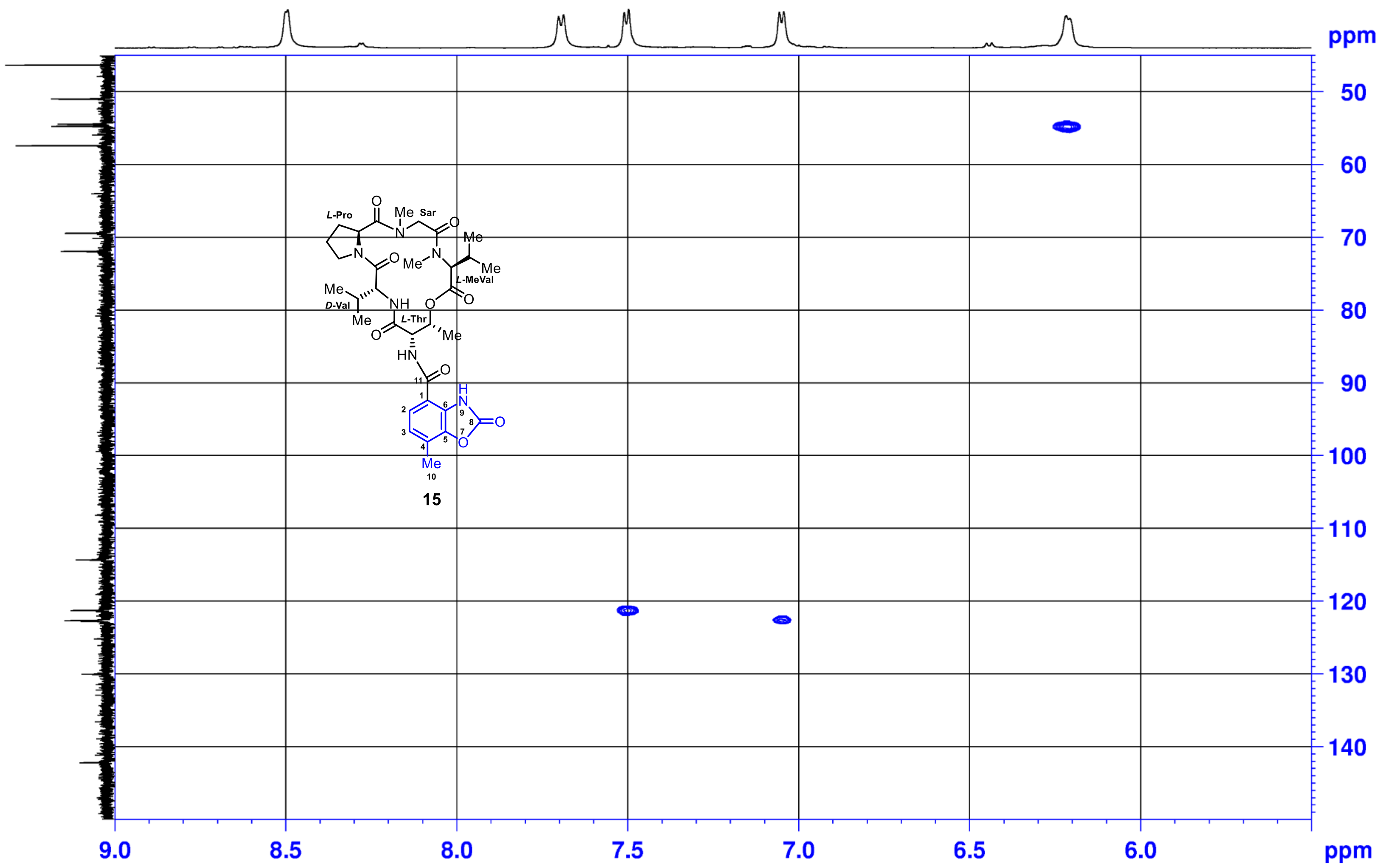

Figure S153. HMBC (600 MHz) spectrum of compound **15** in DMSO-d_6

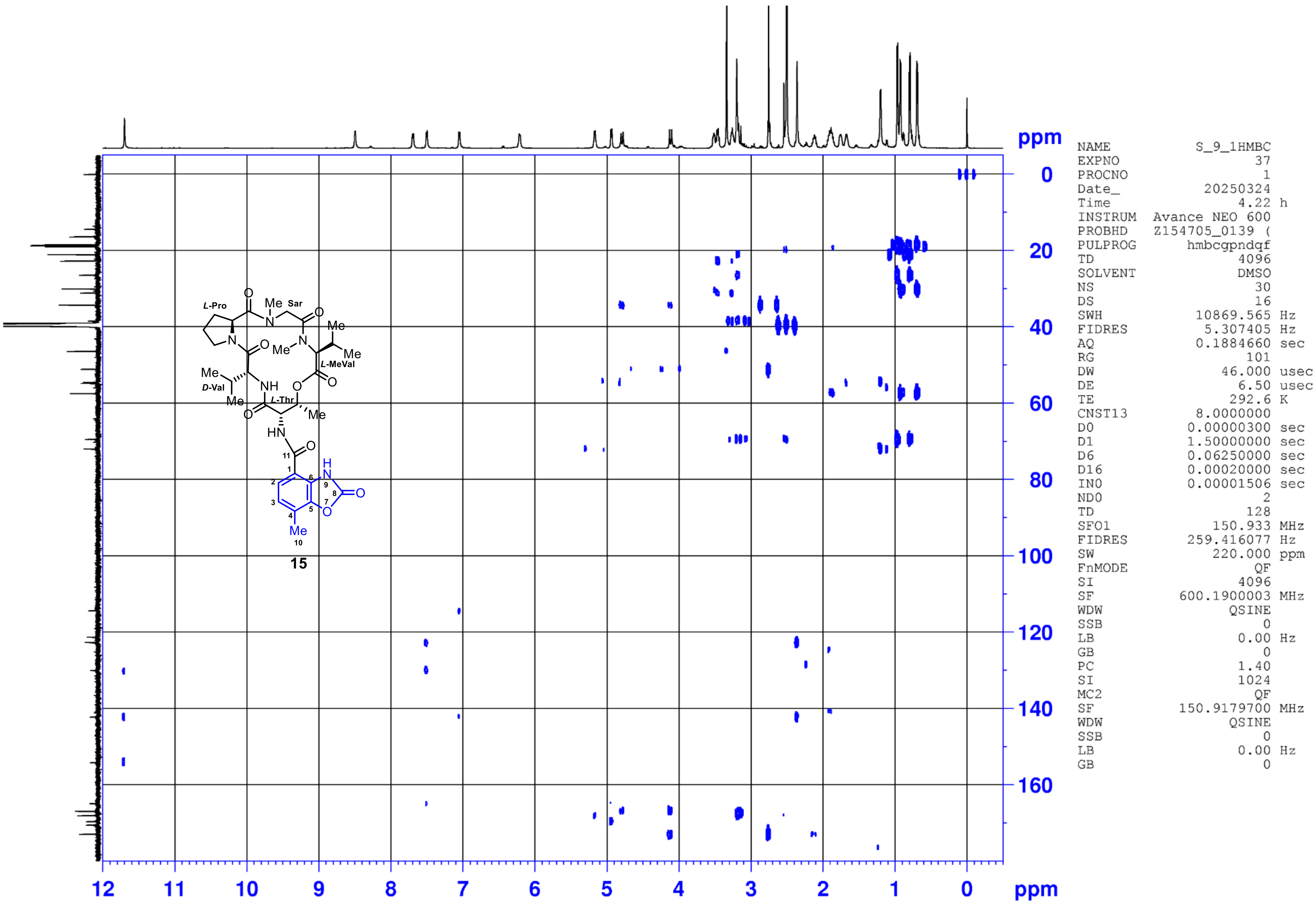

Figure S153-continue

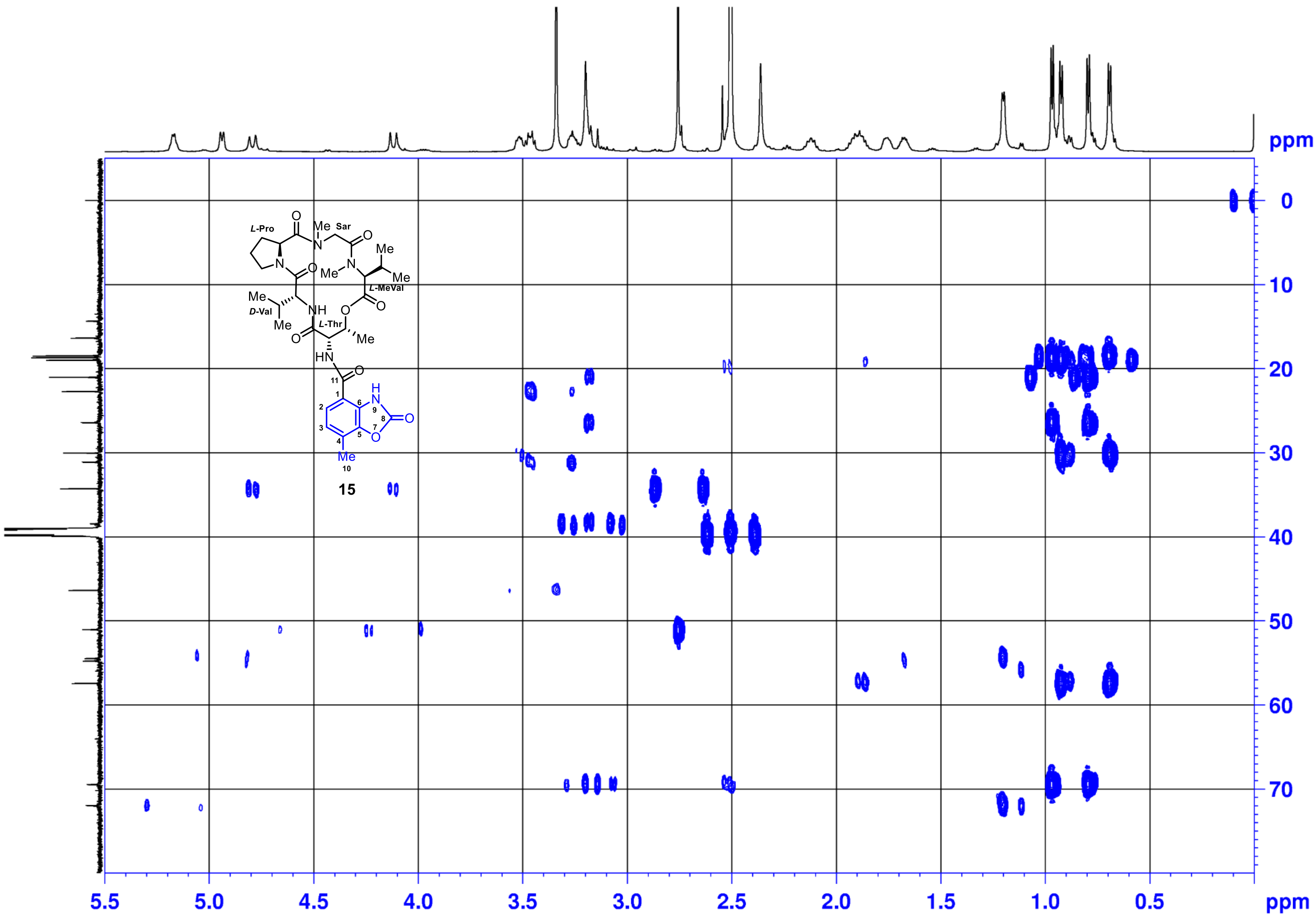

Figure S153-continue

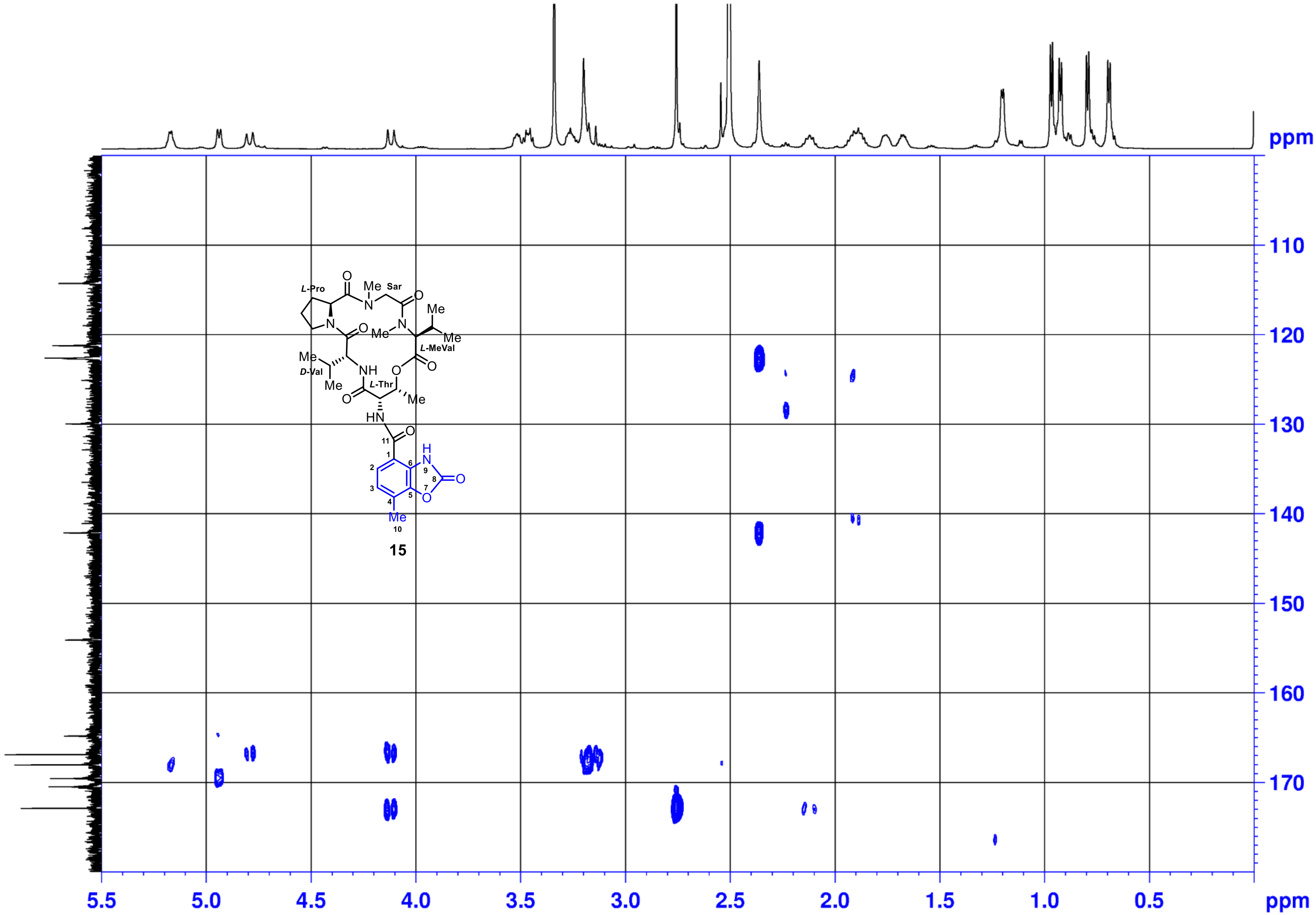

Figure S153-continue

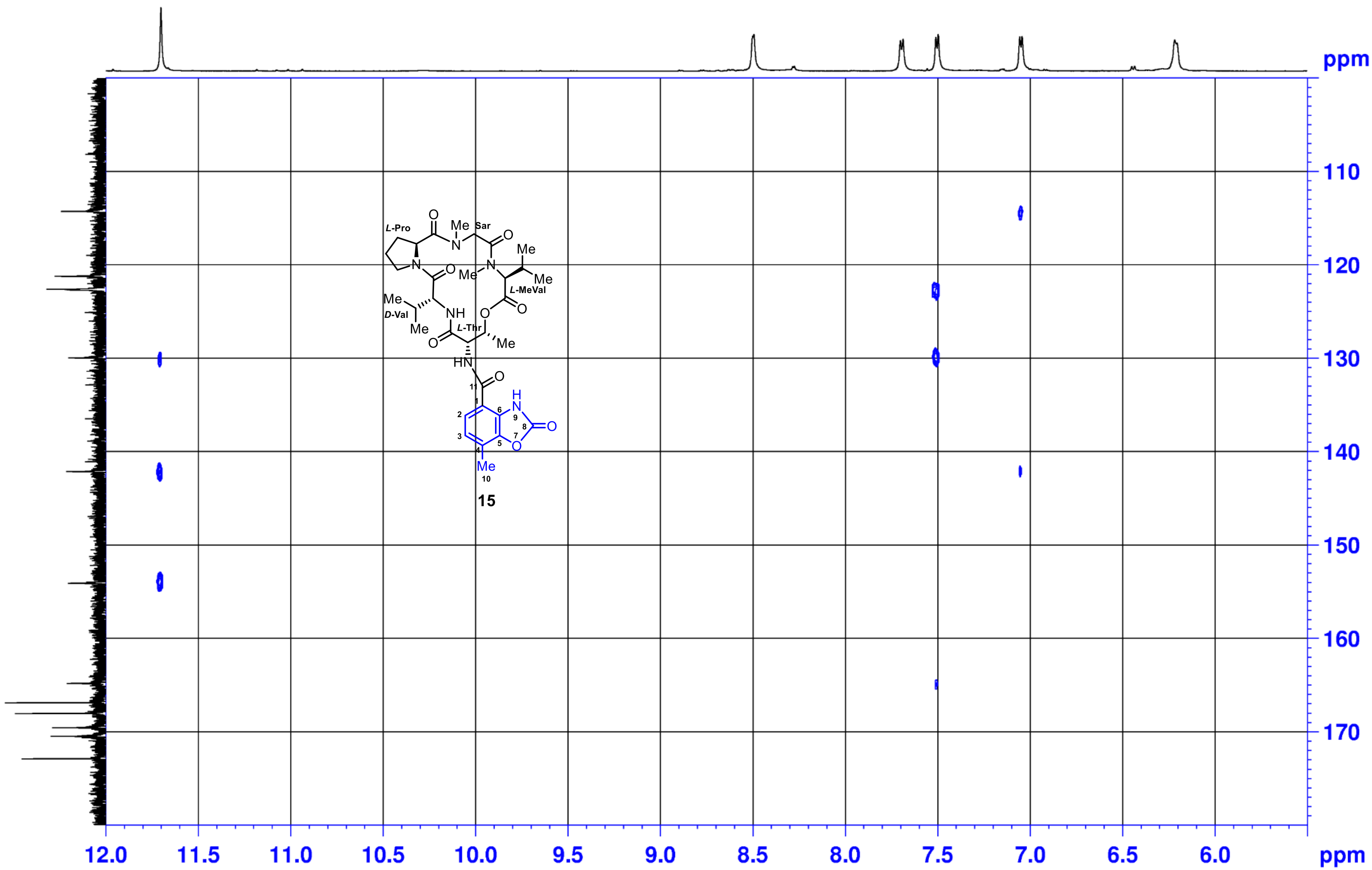

Figure S154. ROESY (600 MHz) spectrum of compound **15** in DMSO-d_6

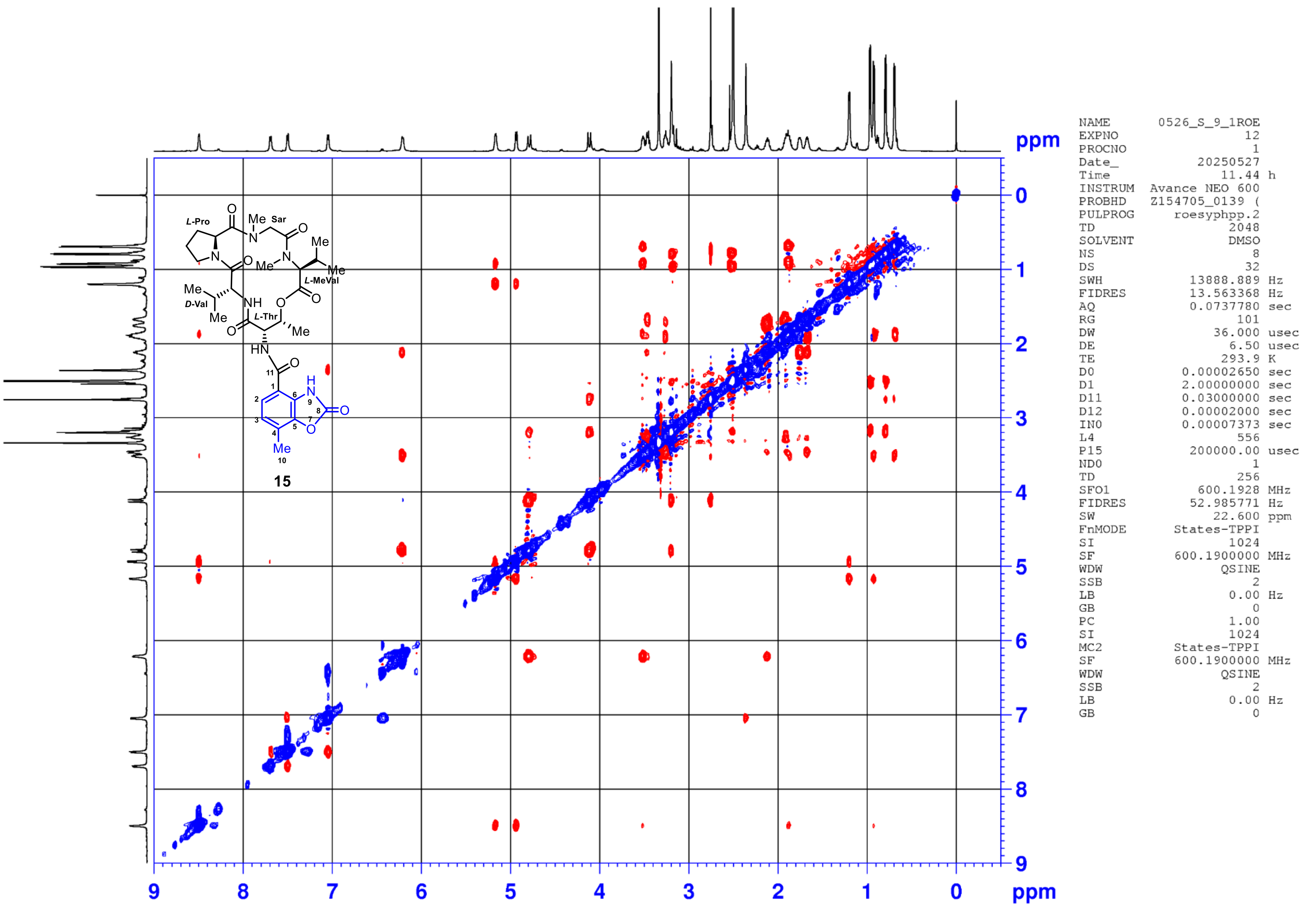

Figure S154-continue

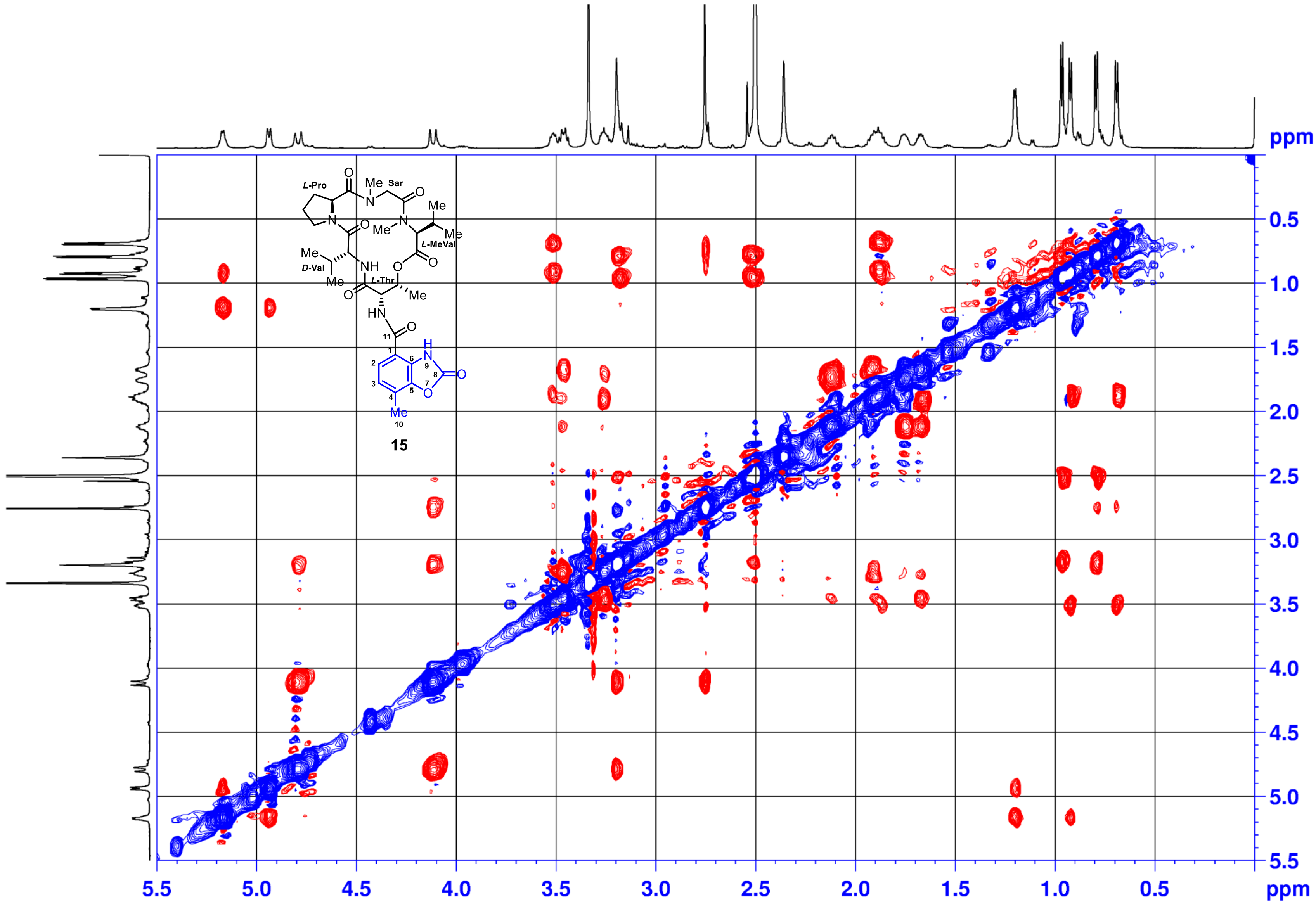

Figure S154-continue

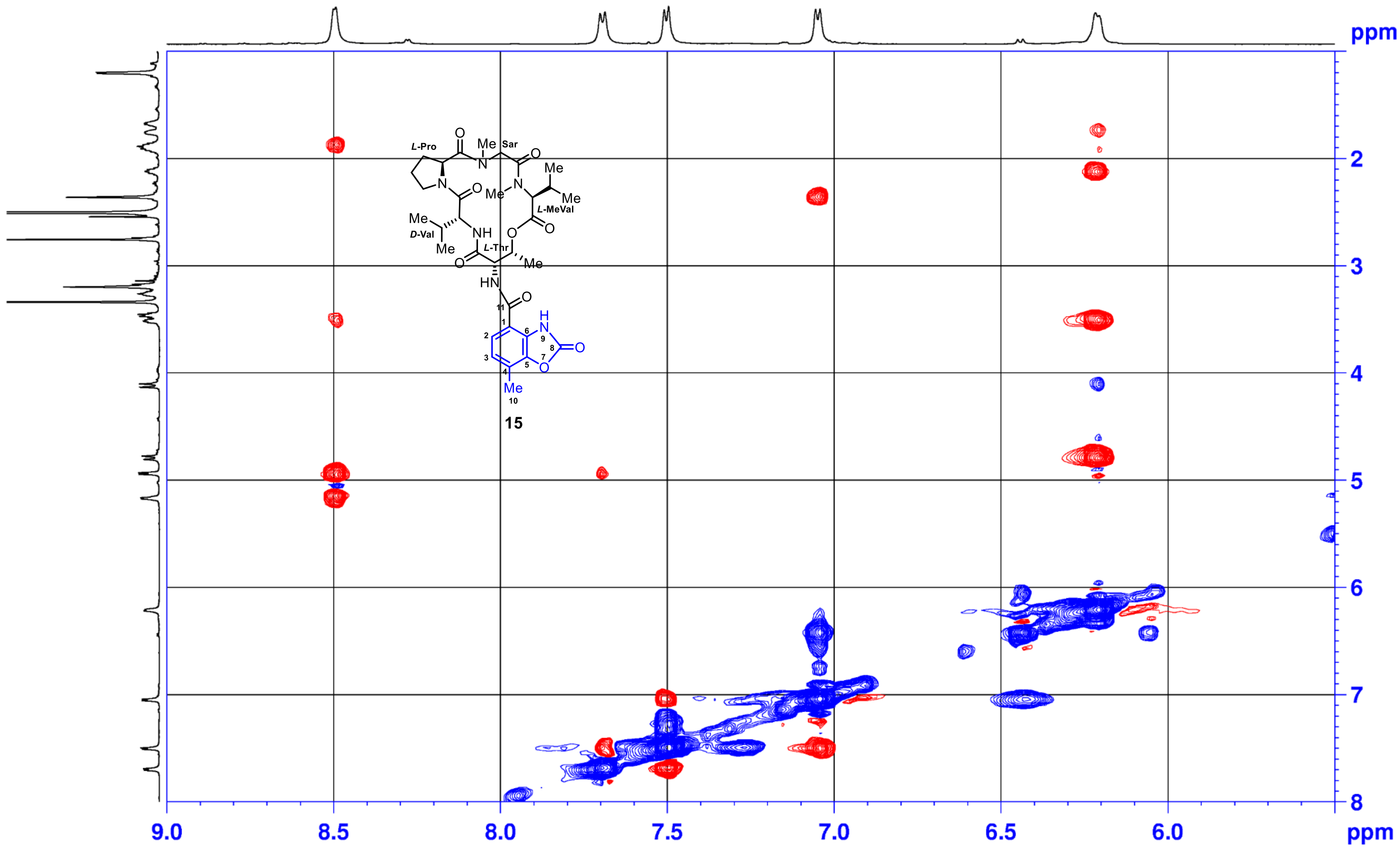

Figure S155. Marfey's analysis of compound **15** and standard amino acids

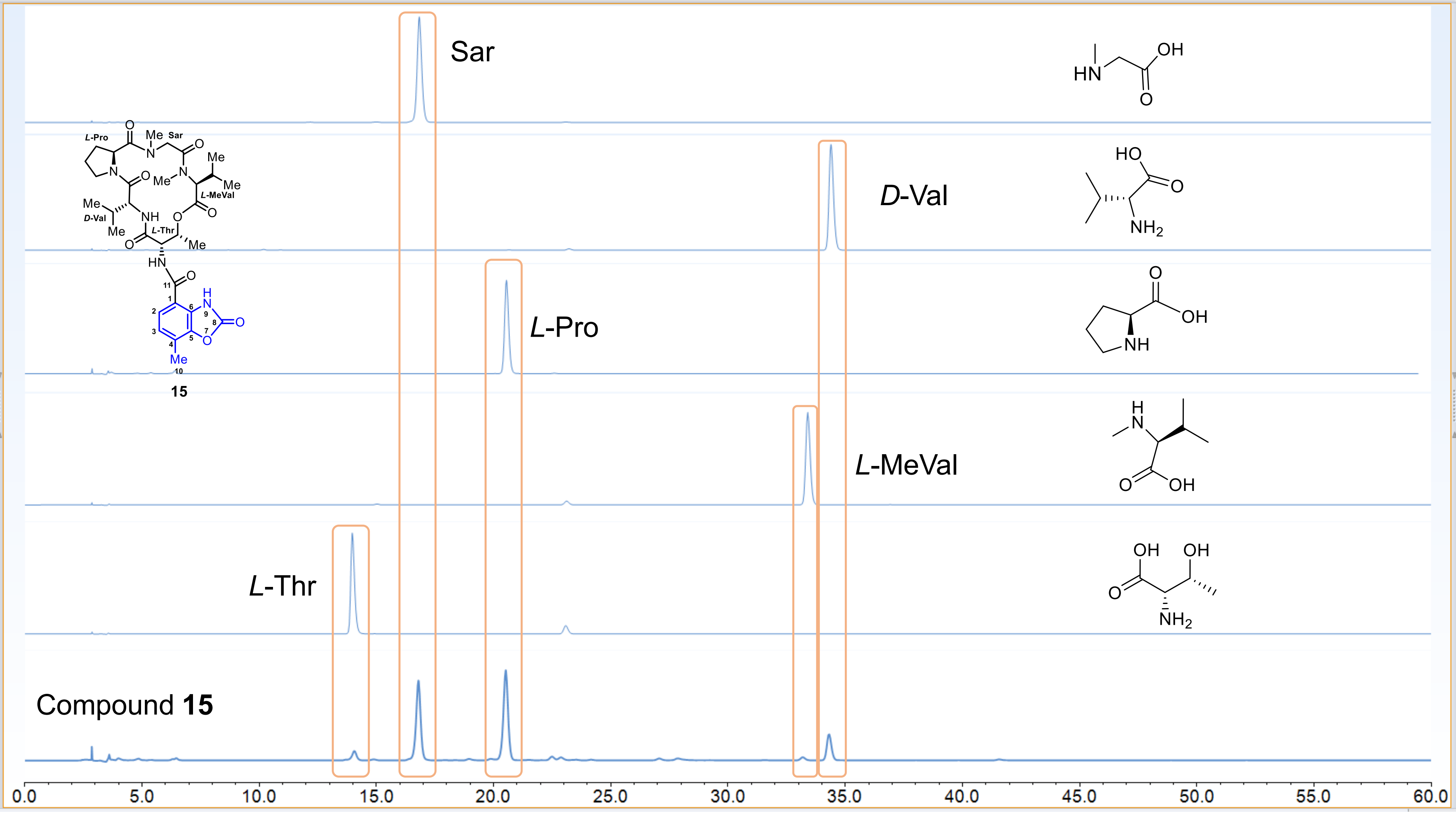

Figure S156. IC50 values of compounds **1–15** in MDA-MB-231 and 4T1 TNBC Cells

Compound	MDA-MB-231	4T1	Compound	MDA-MB-231	4T1
**1**	28.00 nM	47.43 nM	**9**	4.34 μM	7.92 μM
**2**	30.13 nM	57.81 nM	**10**	>20 μM	>20 μM
**3**	32.54 nM	147.51 nM	**11**	>20 μM	>20 μM
**4**	11.89 nM	50.64 nM	**12**	>20 μM	>20 μM
**5**	103.11 nM	289.12 nM	**13**	>20 μM	>20 μM
**6**	2.51 μM	3.59 μM	**14**	>20 μM	>20 μM
**7**	>20 μM	>20 μM	**15**	>20 μM	>20 μM
**8**	>20 μM	>20 μM			
